# Genome-wide cloning and sequence analysis of leucine-rich repeat receptor-like protein kinase genes in *Arabidopsis thaliana*

**DOI:** 10.1186/1471-2164-11-19

**Published:** 2010-01-11

**Authors:** Xiaoping Gou, Kai He, Hui Yang, Tong Yuan, Honghui Lin, Steven D Clouse, Jia Li

**Affiliations:** 1 Department of Botany and Microbiology, University of Oklahoma, Norman, OK 73019, USA; 2 College of Life Sciences, Lanzhou University, Lanzhou 730000, PR China; 3 College of Life Sciences, Sichuan University, Chengdu 610064, PR China; 4 Department of Horticultural Science, North Carolina State University, Raleigh, NC 27695, USA

## Abstract

**Background:**

Transmembrane receptor kinases play critical roles in both animal and plant signaling pathways regulating growth, development, differentiation, cell death, and pathogenic defense responses. In *Arabidopsis thaliana*, there are at least 223 Leucine-rich repeat receptor-like kinases (LRR-RLKs), representing one of the largest protein families. Although functional roles for a handful of LRR-RLKs have been revealed, the functions of the majority of members in this protein family have not been elucidated.

**Results:**

As a resource for the in-depth analysis of this important protein family, the complementary DNA sequences (cDNAs) of 194 LRR-RLKs were cloned into the Gateway^R ^donor vector pDONR/Zeo^R ^and analyzed by DNA sequencing. Among them, 157 clones showed sequences identical to the predictions in the Arabidopsis sequence resource, TAIR8. The other 37 cDNAs showed gene structures distinct from the predictions of TAIR8, which was mainly caused by alternative splicing of pre-mRNA. Most of the genes have been further cloned into Gateway^R ^destination vectors with GFP or FLAG epitope tags and have been transformed into Arabidopsis for *in planta *functional analysis. All clones from this study have been submitted to the Arabidopsis Biological Resource Center (ABRC) at Ohio State University for full accessibility by the Arabidopsis research community.

**Conclusions:**

Most of the Arabidopsis LRR-RLK genes have been isolated and the sequence analysis showed a number of alternatively spliced variants. The generated resources, including cDNA entry clones, expression constructs and transgenic plants, will facilitate further functional analysis of the members of this important gene family.

## Background

Multi-cellular organisms such as plants and animals use cell surface receptors to sense and transduce chemical signals for cell-to-cell communications. One of the most important groups of cell surface receptors, the receptor-like protein kinases (RLKs), has unique structural features that make them particularly suitable for cell-to-cell signaling. A typical RLK contains an extracellular receptor domain to perceive a specific signal, a single-pass transmembrane domain to anchor the protein within the membrane, and a cytoplasmic kinase domain to transduce the signal downstream *via *autophosphorylation followed by further phosphorylation of specific substrates. Plant receptor kinases were originally named "receptor-like" protein kinases since ligands for these receptors were largely unknown at the time when the first RLK was identified in maize [1]. Since then, a small number of RLKs have been functionally characterized in plants and a few specific ligands have been identified. They play essential roles in plant growth, development, pathogen resistance and cell death [2-8].

In the model plant Arabidopsis, both transmembrane RLKs and receptor-like cytoplasmic kinases (RLCKs, which lack extracellular domains) belong to a large, monophyletic gene superfamily of at least 610 members, representing nearly 2.5% of the protein coding sequences within the entire genome [9,10]. About two thirds of genes in this superfamily encode proteins with a typical N-terminal signal peptide and a hydrophobic transmembrane domain, which are consistent structural features of transmembrane RLKs. Based on their structural and sequence similarities, the RLKs are further grouped into more than 10 subfamilies. Leucine-rich repeat (LRR)-RLKs represent the largest subfamily in the Arabidopsis genome with at least 223 members [10].

Despite the identification of a large number of LRR-RLKs in Arabidopsis, biological functions have been defined for only about 30 proteins (Additional file 1: Table S1), which play crucial roles in a variety of different physiological processes. For instance, ERECTA (ER) regulates organ shape and inflorescence architecture [11]; CLAVATA1 (CLV1) determines the balance between undifferentiated and differentiated shoot and floral meristem cells [12]; BRASSINOSTEROID-INSENSITIVE 1 (BRI1) and BRI1-ASSOCIATED RECEPTOR KINASE 1 (BAK1) are a pair of RLKs involved in brassinosteroid (BR) signaling [13-15]; HAESA controls floral organ abscission [16]; FLAGELLIN-SENSITIVE 2 (FLS2) contributes to plant defense/pathogen-recognition [17]; VASCULAR HIGHWAY 1 (VH1) influences leaf cell patterning [18]; and EXCESS MICROSPOCYTES 1 (EMS1), SOMATIC EMBRYOGENESIS RECEPTOR KINASE 1 (SERK1) and SERK2 play important roles in microsporogenesis and male sterility [19-21]. Other LRR-RLKs of known function include RECEPTOR-LIKE PROTEIN KINASE 1 (RPK1), involved in abscisic acid early signaling [22,23]; TOAD2 and its redundant homologue RPK1, both required in Arabidopsis embryonic pattern formation [24]; PXY, responsible for maintaining vascular tissue polarity [25]; and GASSHO1 (GSO1) and GASSHO2 (GSO2) which are essential for the normal development of epidermal surface of Arabidopsis embryos [26]. Recently, two LRR-RLKs, BIR1 and SOBIR1, were identified to regulate cell death and innate immunity in Arabidopsis [27]. Interestingly, several RLKs were found to possess dual or multiple roles during plant growth and development. For example, ERECTA is involved in both plant development and pathogen defense responses [28]. BAK1 and BAK1-LIKE 1 (BKK1) regulate BR-dependent cell growth, and play an important role in cell-death control under various biotic and abiotic stresses. When plants are attacked by bacterial pathogens, BAK1 also can be recruited to the FLS2 complex and regulates the innate immunity response [29-32].

Reverse genetics has been used as a routine and effective approach to dissect the biological functions of genes. Isolated complementary DNA (cDNA) sequences are valuable resources in many processes in determining the functions of their corresponding genes. For example, the cDNA sequences can be used for ectopic expression, complementary experiments for gene knock out lines, site-directed mutagenesis, dominant negative analysis, gene silencing and RNA interference, subcellular localization of epitope-tagged fusion proteins, and protein-protein interaction analysis. Epitope-tagged fusion proteins can also facilitate the proteomic studies of interesting genes. For example, *in vivo *phosphorylation sites of BRI1 and BAK1 were identified by immunoprecipitation of epitope-tagged BRI1/BAK1 from Arabidopsis followed by liquid chromatography-tandem mass spectrometry (LC/MS/MS) and the functions of the identified phosphorylation sites were determined *in planta *[33,34].

In this paper, the full-length cDNA cloning of the entire Arabidopsis *LRR-RLK *subfamily genes is reported. A total of 194 cDNA sequences have been successfully amplified by RT-PCR and cloned into a Gateway^R ^donor vector pDONR/Zeo^R^. Sequence analysis indicated that 157 cDNAs are identical to the predicted or earlier submitted cDNA sequences in The Arabidopsis Information Resource (TAIR) database, whereas 37 other genes showed altered cDNA sequences distinct from those presented in the database, which is likely due to alternative splicing of pre-mRNA. One hundred eighty cDNA sequences with 100% sequence accuracy were further transferred, by *in vitro *DNA recombination, into two different destination vectors with either FLAG or GFP as the C-terminal fusion tags. Preliminary results indicated that most of the gene products can be detected by Western hybridization analysis using anti-FLAG or anti-GFP antibodies. The results and resources generated by this study will be useful tools for future functional analyses of LRR-RLKs.

## Results

### Construction of Gateway^R^-compatible binary vectors for plant transformation

**Figure 1:**
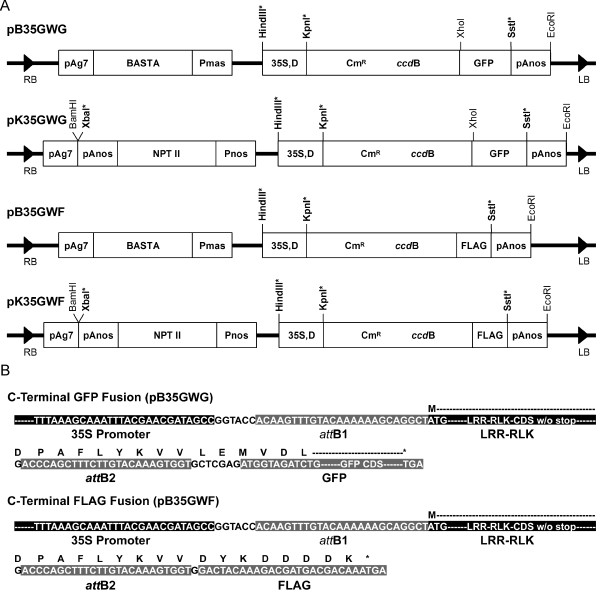
**Gene transformation constructs generated in this study**. Four Gateway^R^-compatible cloning vectors developed specifically in this study. All the four vectors were derived from pBIB vectors [60] by inserting the Gateway^R ^module and BASTA resistance gene. Gateway^R^-mediated addition of GFP and FLAG epitope tags to the C-terminal ends of target sequences in vectors pB35GWG and pB35GWF. The *att*B sites are from the recombination between *att*L and *att*R sites. The target *LRR-RLK *sequence without stop codon is inserted between the *att*B1 and *att*B2 sites. To make the sequence in-frame with the epitope tags, one extra G is attached to the end of the C-terminus of the target sequence. Amino acids are indicated with a single-letter code. Additional amino acids from *att*B sites and linking sequences in destination vectors are added to the final protein.

To facilitate future functional analyses of all LRR-RLKs, we generated 4 different Gateway^R^-compatible binary vectors for high through-put cloning of *LRR-RLKs *(Figure 1A). The four vectors contain a Gateway^R ^cassette for DNA recombination with plasmid DNA of entry clones to produce final expression constructs. GFP or FLAG sequences were integrated at the 3' terminus of the Gateway^R ^cassette for the production of epitope-tagged fusion proteins that will facilitate subsequent immunoprecipitation and coimmunoprecipitation analyses.

The first vector, named pB35GWG, contains a BASTA resistance gene for selecting transgenic plants and a C-terminal GFP tag. The second vector, designated pK35GWG, uses a kanamycin resistance gene for selecting transgenic plants, also with a C-terminal GFP tag. The third vector, termed pB35GWF, uses the BASTA gene for transgenic selection and FLAG as the C-terminal fusion tag. The fourth vector, labeled pK35GWF, contains a kanamycin resistance gene as the selectable marker and again has a C-terminal FLAG tag. All vectors use the CaMV 35 S promoter with dual enhancers to drive expression of the gene of interest. Detailed sequence information of the junction region of the Gateway^R ^cassette and the GFP or FLAG eptiope tags is also shown (Figure 1B).

**Figure 2:**
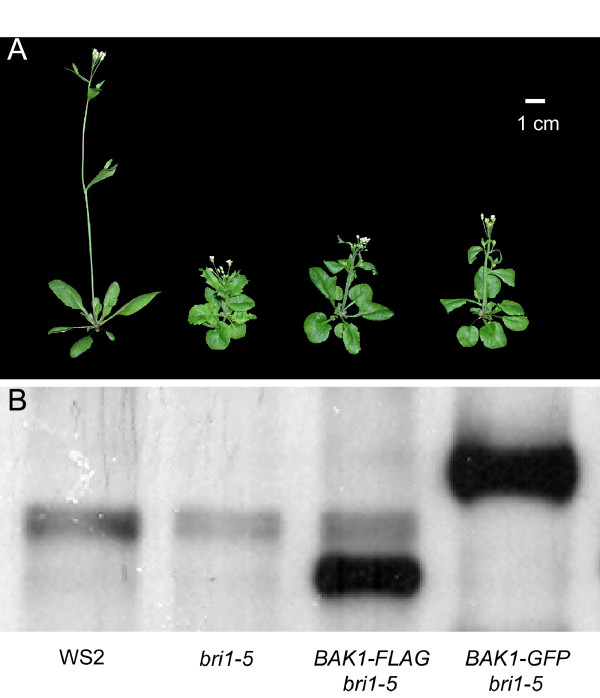
**Functional examination of the generated Gateway**^R^**destination vectors**. Full-length cDNA sequence of *BAK1 *was PCR amplified from Arabidopsis Col-0 plants and introduced into two destination vectors, pB35GWG and pB35GWF, by *in vitro *DNA recombination mediated by BP clonase and LR clonase. Expression constructs harboring *BAK1 *cDNA were transformed into the *bri1-5 *mutant, a weak allele of *bri1*. Overexpression of *BAK1 *using the vectors can suppress the *bri1-5 *mutant phenotype, indicating that the vectors are functional. Expression of *BAK1 *was confirmed by Western hybridization in transgenic plants. Anti-FLAG and anti-GFP antibodies were mixed to detect the signals on one membrane.

To examine whether the newly-constructed Gateway^R^-compatible vectors are reliable in generating *LRR-RLK *overexpressed transgenic plants, a functionally characterized gene, *BAK1*, was used for the test. Previous studies have shown that *BAK1 *is involved in the BR signal transduction pathway [14,15,34]. Overexpession of *BAK1 *can suppress the dwarf phenotype of the *bri1 *weak allele, *bri1-5*, to wildtype [14,15,34]. To clone *BAK1 *into the destination vectors, *att*B1 and *att*B2 flanked *BAK1 *was PCR-amplified and gel purified as described in experimental procedures. After BP and LR clonase reactions, *BAK1 *was transferred into the destination vectors and introduced into *bri1-5 *mutant plants. Obtained transgenic plants showed a typical *bri1-5 *suppression phenotype (Figure 2A). Western hybridization analysis using anti-FLAG or anti-GFP antibodies also indicated that both *BAK1-FLAG *and *BAK1-GFP *were truly overexpressed in the transgenic plants (Figure 2B). The results suggest that the generated destination vectors are fully functional and can be used for cloning and overexpression of all *LRR-RLKs *in Arabidopsis plants for future functional analyses.

### Gateway^R ^cloning of *LRR-RLKs*

A three-step protocol was used to efficiently produce *att*B1- and *att*B2-flanked *LRR-RLK *ORF fragments (Additional file 2A): (a) the reverse transcriptase reaction to generate single-stranded cDNA; (b) the first round of PCR with gene-specific primers to amplify target ORF flanked with partial *att*B1 and *att*B2 adaptor sequences; and (c) the second round of PCR with universal *att*B1 and *att*B2 adaptor primers to integrate complete *att*B1 and *att*B2 sites into the ORF amplicons. Two hundred twenty three predicted *LRR-RLKs *distributed on all five chromosomes of the Arabidopsis genome with ORF sizes ranging from 339 bp -3,759 bp are presented in TAIR8. The coding sequences of 221 *LRR-RLKs *are larger than 1,500 bp. Superscript III was used to produce long cDNAs with full-length ORFs and a proof-reading polymerase (AccuPfx) was employed to amplify the predicted ORFs with high fidelity. Two rounds of PCR can produce enough DNA for Gateway^R ^cloning even for some genes with relatively low expression. PCR products were obtained for 208 of the 223 predicted *LRR-RLKs *genes, while 15 genes were never amplified by RT-PCR (Additional file 2B). All PCR products were agarose gel purified and introduced into pDONR/Zeo^R ^to produce the entry clones. Plasmid DNA from entry clones was then used for LR clonase-mediated *in vitro *DNA recombination with appropriate destination vectors to yield FLAG and GFP epitope tagged constructs.

### Sequence analysis of the isolated *LRR-RLKs*

**Figure 3:**
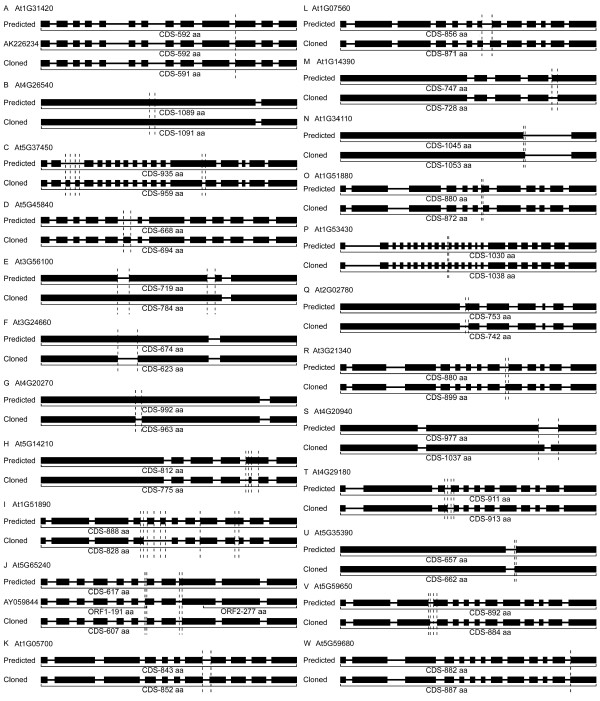
**Experimentally derived *LRR-RLK *cDNAs displaying different coding sequences and containing one continuous ORF**. Black boxes indicate exons, and lines between exons represent introns. Vertical dotted lines indicate the differences between predicted and isolated sequences. Size of produced amino acid sequence is indicated under each molecule. The accession numbers for each sequence can be found in Additional file 1: Table S7.

**Figure 4:**
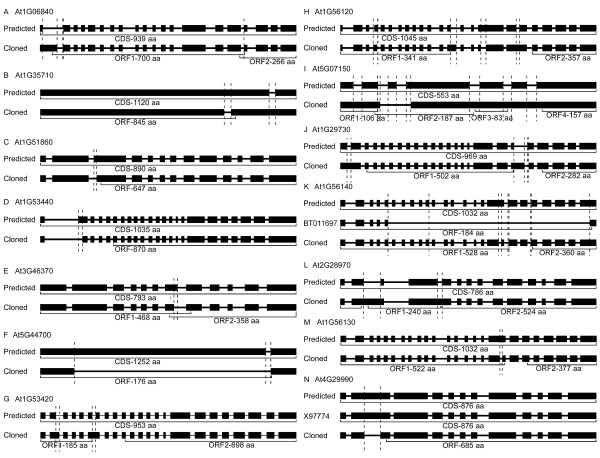
**Experimentally derived *LRR-RLK *cDNAs showing different coding sequences but not containing one continuous ORF**. Black boxes indicate exons, and lines between exons represent introns. Vertical dotted lines indicate the differences between predicted and isolated sequences. Size of produced amino acid sequence is indicated under each molecule. The accession numbers for each sequence can be found in Additional file 1: Table S8.

A total of 194 cDNA sequences were successfully cloned into the donor vector and are summarized in Table S2 in Additional file 1. Among them, 157 (80.9%) of the clones contain cDNA sequences identical to those predicted in TAIR8 (Additional file 1: Table S3). The other 37 isolated sequences (19.1%) display gene structures that are different from their corresponding predictions in TAIR8 (Additional file 1: Table S4, S5). Based on their structural differences, they can be divided into two groups: (1) one complete ORF exists from the predicted start codon to the predicted stop codon despite the coding sequences being different from that predicted (Figure 3); (2) no continuous ORF exists from the predicted start codon to the predicted stop codon because of the different coding sequences (Figure 4). The other 29 *LRR-RLKs *(Additional file 1: Table S6) were not isolated successfully because of possible wrong annotation, specific and/or low expression, and bactericidal effect.

The first group includes 23 genes (Figure 3, Additional file 1: Table S4). The detailed sequence differences are summarized in Table S7 and the alignments among isolated cDNAs, predicted ORFs and the corresponding genomic DNA sequences are shown in Additional file 3. The isolated sequence of At1g31420 is 3 bp shorter than the prediction. One clone [GenBank:AK226234] with the same sequence as the prediction was found in database, indicating that this gene has transcripts with alternative splicing (Figure 3A). The isolated sequence of At4g26540 is 6 bp longer than the prediction, and both Cs at position 1,412 and 1,484 in the isolated sequence are not found in the Arabidopsis genome (Figure 3B). The isolated sequence of At5g37450 displays two unpredicted exons and shows one unpredicted intron in predicted sequence (Figure 3C). An unpredicted exon is found in the isolated sequence of At5g45840 (Figure 3D). Two predicted introns in gene At3g56100 are eliminated in the isolated sequence and have become a part of the first exon (Figure 3E). The first predicted exons of At3g24660 and At4g20270 have one unpredicted extra intron each (Figure 3F, G). The predicted 5th exon of At5g14210 contains one extra unpredicted intron, and the intron/exon boundary is also different from that predicted (Figure 3H). Two predicted exons disappear and one unpredicted intron is shown in the predicted 10th exon of At1g51890, and a different intron/exon boundary is also observed (Figure 3I). The isolated sequence of At5g65240 is 30 bp shorter than the prediction because of the different intron/exon boundaries; and a RIKEN clone [GenBank:AY059844] without a continuous ORF from the predicted start codon to the predicted stop codon is available in the database (Figure 3J). Isolated sequences of the other 13 genes, At1g05700, At1g07560, At1g14390, At1g34110, At1g51880, At1g53430, At2g02780, At3g21340, At4g20940, At4g29180, At5g35390, At5g59650 and At5g59680, show different intron/exon boundaries compared with the predicted sequences, resulting in different mRNA sequences (Figure 3K-W).

The second group contains 14 genes (Figure 4, Additional file 1: Table S5). The detailed sequence differences are summarized in Table S8 in Additional file 1 and the alignments among isolated cDNAs, predicted ORFs and the corresponding genomic DNA sequences are shown in Additional file 4. Unlike the genes in the first group, the isolated sequences in this group do not display continuous ORFs from the predicted start codon to the predicted stop codon that were used to design the forward and reverse PCR primers for Gateway^R ^cloning. The isolated sequences of genes At1g06840, At1g35710, At1g51860, At1g53440, At3g46370 and At5g44700 exhibit different intron/exon boundaries compared to the predicted ORF sequences (Figure 4A-F). Different intron/exon boundaries are also found in the isolated ORF sequences of At1g53420, At1g56120, At5g07150, At1g29730 and At1g56140, with other structural differences (Figure 4G-K). The predicted 6th intron disappears in the isolated ORF sequence of At1g53420 (Figure 4G). The predicted intron 17, exon 17 and exon 18 are merged into exon 18 and the predicted exon 16 is split into exon 16 and exon 17 in the isolated sequence of At1g56120 (Figure 4H). The third predicted exon does not exist in the experimentally derived sequence of At5g07150, and the other six predicted exons are merged into two exons (Figure 4I). The first two exons and the first intron in the prediction of At1g29730 merge into the first exon in the isolated sequence (Figure 4J). The predicted exon 17 is split into exon 17 and exon 18 in the isolated sequence of At1g56140. In database, a previously isolated sequence [GenBank:BT011697] is different from both the prediction of At1g56140 in TAIR8 and the sequence from this report, losing the sequence from exon 6 to exon 23 and part of exon 24, resulting in a much smaller protein with 184 aa compared to the predicted protein of 1,032 aa (Figure 4K). Two predicted introns in At2g28970 do not exist in the isolated sequence (Figure 4L). At1g56130 displays an unpredicted intron (Figure 4M). One extra unpredicted intron is shown in At4g29990, and the isolated sequence is different from both the existing sequence [GenBank:X97774] and the TAIR prediction (Figure 4N).

**Figure 5:**
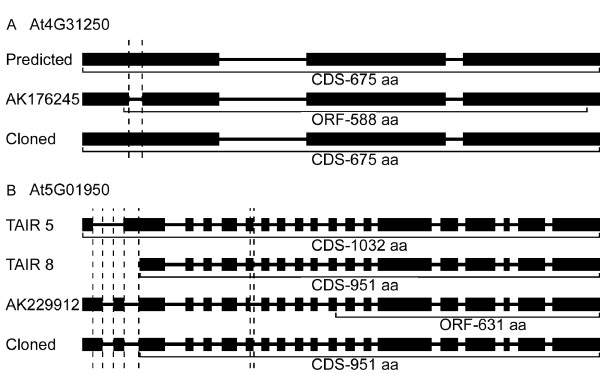
**Two *LRR-RLKs *producing alternatively spliced transcripts**. At4g31250, the isolated sequence in this report is the same as the TAIR8 prediction with an ORF of 675 aa. A previously reported sequence, AK176245, shows an unpredicted intron, producing a shorter protein of 588 aa with delayed start codon and early stop codon. The GenBank accession number for the isolated sequence is FJ708761. At5g01950, the first two exons in TAIR5 prediction are shown as three exons in both experimental sequences. AK229912 displays a different intron/exon boundary with a shorter exon 7, resulting in a shorter protein of 631 aa. The GenBank accession number for the isolated sequence is FJ708768.

Although the isolated sequences of genes At4g31250 and At5g01950 are the same as the current predictions in TAIR8, the previously reported coding sequences of them are different (Figure 5A, 5B). The predicted exon 1 of At4g31250 is split into exon 1 and exon 2 in sequence AK176245 [GenBank:AK176245] (Figure 5A). Gene At5g01950 has a new annotation in TAIR8. The isolated sequence contains the same ORF as the current prediction, but the first two predicted exons in TAIR5 are arranged as three exons. The existing sequence AK229912 [GenBank: AK229912] shows a different intron/exon boundary between exon 7 and intron 7, resulting in a smaller ORF of 631 amino acids (Figure 5B).

### Detection of alternative splicing of *LRR-RLKs*

**Figure 6:**
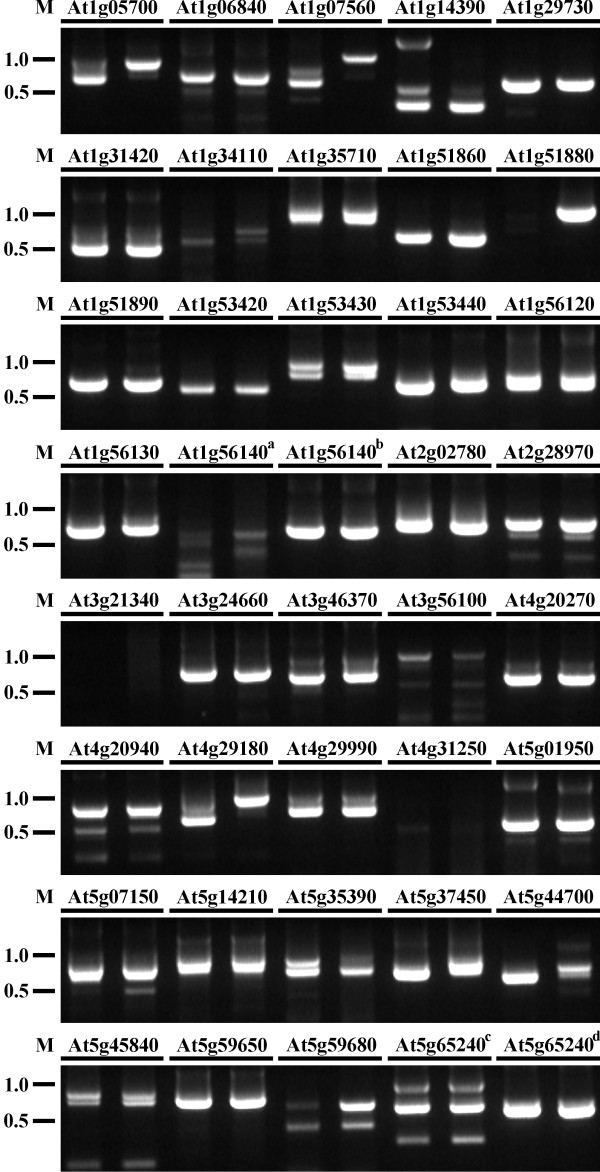
**Confirmation of alternative splicing of *LRR-RLKs *by RT-PCR**. Total RNA was extracted from inflorescence and leaf of Arabidopsis, respectively. The reverse transcribed single-stranded cDNA was used as template for nested PCR with variant-specific primers to confirm potentially alternatively spliced variants emerged from the predicted mRNA sequences and previous reports. The nested PCR products were separated on a 1.5% (w/v) agarose gel. The AGI numbers for examined genes are shown on the top of the lanes. For each gene, the left lane shows the result from inflorescence and the right lane shows the result from leaf. a, Previously reported variant [GenBank:BT011697] of At1g56140; b, Predicted sequence [GenBank:NM_104492] of At1g56140; c, Predicted sequence [GenBank:NM_125922] of At5g65240; d, Previously reported variant [GenBank:AY059844] of At5g65240. M, molecular weight markers (kb).

Potentially alternatively spliced variants of 38 *LRR-RLKs *were examined by RT-PCR with variant-specific primers according to the predicted mRNA sequences and previous reports (Figure 6). Isolated cDNA sequences from this study were not examined because they were identified by RT-PCR during the cloning procedure. Isolated cDNA of At4g26540 in this report showed a structure with slight difference from the prediction in database, which made it difficult to examine the sequence difference with variant-specific primers. This gene was not included in the RT-PCR experiment. From inflorescence, 34 variants of 33 *LRR-RLKs *were confirmed by RT-PCR with expected size of products (Figure 6). No RT-PCR products were obtained from At1g34110, At1g51880, At3g21340, At3g56100 and At4g31250. The previously reported cDNA sequence [GenBank: BT011697] of At1g56140 was not amplified from this study, but the predicted variant sequence [GenBank: NM_104492] of it was confirmed by RT-PCR. Both the previously reported cDNA sequence [GenBank: AY059844] and the predicted mRNA sequence [GenBank: NM_125922] of At5g65240 were confirmed in this study. From leaf, RT-PCR fragments of 35 variants of 34 *LRR-RLKs *were obtained (Figure 6). No RT-PCR products with expected sizes were obtained for the same genes as in inflorescence except At1g51880 that produced a larger fragment than predicted. No RT-PCR product of previously reported cDNA [GenBank: AK176245] of At4g31250 was recovered. Together, a total of 34 *LRR-RLKs *were confirmed with alternative splicing of pre-mRNA, including four previously reported cDNA variants of At1g31420 [GenBank: AK226234], At4g29990 [GenBank: X97774], At5g01950 [GenBank: AK229912] and At5g65240 [GenBank: AY059844].

### LRR-RLKs phylogenetic analysis

Sequence analyses of isolated *LRR-RLKs *reported in this paper demonstrate that some of them encode protein sequences distinct from the predictions. This sequence variation and the improved annotation of Arabidopsis genome makes it necessary to examine the previously created phylogenies of this superfamily. The previous report suggested 15 subfamilies because the sequences clearly fell into distinct clades [10]. Studies in this report based on the alignment of the full-length amino acid sequences result in a similar phylogenetic tree to the previous report [10] with minor adjustments (Additional file 5). (1) At1g74360, a member of the previously assigned subfamily LRR X, fell into the LRR VII subfamily; (2) two members (At1g35710 and At4g08850) of the previously assigned subfamily LRR XII, one previously ungrouped gene (At2g25790), and one member (At5g51350) of the subfamily LRR XIV fell into the LRR XI subfamily.

### Epitope-tagged proteins of LRR-RLKs in transgenic Arabidopsis plants

**Figure 7:**
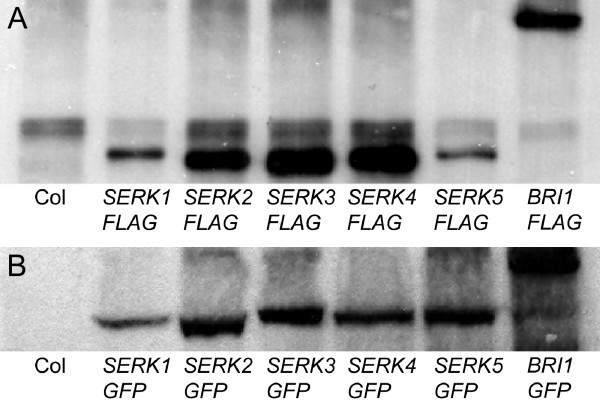
**Detection of epitope-tagged recombinant LRR-RLK proteins in Arabidopsis with Western hybridization**. Full-length cDNAs of *SERK1*, *SERK*2, *SERK3, SERK4, SERK5 *and *BRI1 *were recombined into pB35GWF and pK35GWG. FLAG-tagged fusion proteins, prepared by immunoprecipitation of total membrane protein, and total proteins from leaves of transgenic plants expressing GFP-tagged fusion proteins were subjected to sodium dodecyl sulfate-polyacrylamide gel electrophoresis (SDS-PAGE). Proteins were transferred to nitrocellulose membrane and epitope-tagged proteins were detected using either (A) anti-FLAG or (B) anti-GFP antibody (diluted 1:3,000) followed by anti-mouse immunoglobulin G (IgG)-horseradish peroxidase (HRP) secondary antibody (diluted 1:10,000).

The expression of *LRR-RLKs *cloned in the destination vectors pB35GWF and pK35GWG and transformed into Arabidopsis ecotype 'Columbia-0 (Col-0)' was verified by Western hybridization analysis with αFLAG and αGFP antibodies respectively (Figure 7A, B). Immunoprecipitated membrane protein was prepared and separated by SDS-PAGE for the detection of FLAG-tagged fusion proteins while total protein could be used directly to detect signals of GFP-tagged fusion proteins. The FLAG- or GFP-tagged LRR-RLKs could be detected in most of the examined transgenic lines usually as one distinct and specific protein band.

## Discussion

### Experimentally derived sequences help to verify and expand the predicted genome annotation

The TAIR annotation release TAIR8 (April, 2008) contains 33,282 genes, including 27,235 putative protein coding genes. Among all the putative protein coding genes, 2,289 genes have not been experimentally supported by identified transcripts. Among the 223 predicted *LRR-RLK *genes, 30 of them have no EST support. EST support for 12 of them is now provided (Additional file 1: Table S9). A total of 94 *LRR-RLK *genes have no isolated full length coding sequence in the existing database. From this study, 70 new *LRR-RLK *cDNAs with full length coding sequence were provided (Additional file 1: Table S10). The resources generated in this study will provide useful tools for future functional analyses of this important protein family. At the same time, phylogenetic analysis can guide researchers to create double, even higher level, mutants to overcome functional redundancy of genes in one subfamily. For example, elegant genetics studies in subfamily LRR II revealed redundant functions of *SERK *genes in brassinosteroid signal transduction [14,15,30,35], male sporogenesis [21,35], pathogen response [29,31,35] and cell death [30,32]. Phylogenetic analysis in this study indicated that several subfamilies, such as LRR XII whose members fell into two different subfamilies based on the phylogeny of full length amino acid sequence, could be rearranged to aim future functional analysis of their gene members.

The sequence data generated from this project will also greatly improve genome annotation [36]. In a previous study, 5,000 full-length gene transcripts from Arabidopsis were used to re-annotate its genome. The results indicated that the gene structures of approximately 35% of the examined genes could be improved according to the isolated full-length cDNA sequences [37]. When examing existing EST and full-length cDNA sequences for all of the predicted *LRR-RLKs*, one full-length cDNA, clone RAFL25-47-F19 [GenBank:AK221400], was identified that covered two predicted loci, At1g51830 and At1g51840, with a full and complete ORF of 886 aa. The loci should be merged into one according to this data. As described above, from this study a total of 37 genes were identified with different variant transcripts compared to the predictions (Additional file 1: Tables S7, S8). All the data are useful for the improvement of Arabidopsis *LRR-RLK *annotation.

### Gene functions and alternatively spliced transcripts

The TAIR8 release showed that 4,330 of the annotated 27,235 protein coding genes (15.9%) have alternatively spliced transcripts. In this report, sequence analyses show that a total of 37 *LRR-RLK *genes have different sequences from the TAIR8 predictions. This includes two possibilities: (1) the prediction was not correct; (2) both the predicted and the isolated sequences exist in plant, which suggests some *LRR-RLK *genes have alternatively spliced transcripts, possibly in the same tissue, or in different tissues, or under different growth conditions.

The sequence analysis of isolated *LRR-RLKs *in this report revealed different forms of the CDS compared to TAIR8 predictions or the existing sequences in the database, including alternative intron donor and/or acceptor sites (for example, At1g05700, At4g20940, At5g44700), unpredicted introns (At3g24660, At4g20270, At1g56130, At4g29990), unpredicted exons (At5g45840), unspliced introns (At3g56100, At2g28970) and different combinations of the aforementioned changes. They form a continuous ORF or several discontinuous ORFs. The presence of the observed alternative splicing was further confirmed by RT-PCR (Figure 6). It is already known that alternative splicing can significantly increase the complexity of the transcriptome and proteome by synthesizing multiple transcripts and proteins from one gene. Several previous reports showed that approximately 20% of Arabidopsis genes are alternatively spliced and some alternatively spliced transcripts have different functions [38-42]. Serine/arginine-rich (SR) proteins form a conserved family of splicing regulators in eukaryotes. The pre-mRNAs of Arabidopsis SR genes are extensively alternatively spliced, and about 95 transcripts are produced from 15 genes. The transcriptome complexity of SR genes is increased by six-fold. Abiotic stresses regulate the alternative splicing of the pre-mRNAs of SR genes to produce different isoforms of SR proteins that are likely to have altered functions in pre-mRNA splicing [41]. Six mRNA variants were generated by alternative splicing in the pre-mRNA of a homologue of SR protein, atSR45a. The transcript abundance and the splicing patterns of atSR45a were altered under various types of stress [42]. The U1 small nuclear ribonucleoprotein particle (U1 snRNP) 70K protein (U1-70K) interacts with splicing factors and is involved in basic and alternative splicing of pre-mRNA [43-47]. In Arabidopsis, two distinct transcripts are produced by alternative splicing of the pre-mRNA of the U1 snRNA 70K gene. Only the short transcript encodes a full-length functional U1-70K, whereas the long transcript codes for a truncated U1-70K [48]. COP1 is a negative regulator of Arabidopsis light-dependent development. COP1b is generated by alternative splicing, resulting in a 60-amino acid deletion in the WD-40 repeat domain relative to the full-length COP1, which functions as a dominant negative regulator of COP1 function [38]. The maize MIK gene codes for a GCK-like MAP4K that can be activated by interaction with maize atypical receptor kinase (MARK) [49]. Four different mature mRNAs of MIK are generated by alternative splicing, and the resulting polypeptides display different kinase activity and are differentially activated by interaction with the MARK receptor [50]. Recent studies further demonstrated that alternative splicing affected regions frequently code for intrinsically disordered regions of the corresponding protein products and the association of alternative splicing and intrinsic disorder results in various isoforms to increase the functional and regulatory diversity of the gene [51-54].

LRR-RLKs are critical proteins involved in many aspects of plant growth, development and stress responses. It is noticed that six genes (At1g05700, At1g07560, At1g51880, At4g29180, At5g37450 and At5g44700) produce RT-PCR fragments with different sizes in inflorescence and leaf (Figure 6), which indicates that different forms of LRR-RLK protein may be required for distinct tissue development and function. Some of the alternatively spliced transcripts of LRR-RLKs will generate truncated versions of the predicted proteins. The truncated proteins may be involved in the functional regulation of these genes in different developmental stages and different growth conditions/stresses. Future functional analyses of the alternatively spliced LRR-RLKs, revealed from this study, would eventually elucidate the biological meaning of the process.

### Gene function and phosphorylation sites analysis

Clones reported in this paper are not only a resource for gene annotation, but also will be very useful for gene function analysis. Genes in entry clones can be transferred freely to any Gateway^R^-compatible destination vectors and introduced into Arabidopsis. They can be used for overexpression in Arabidopsis to dissect the resulting phenotypes that will indicate the possible related pathways and functions of the target genes. They can be used to generate different epitope-tagged fusion proteins. For example, as described above, GFP- and FLAG-tagged fusion protein can be produced in transgenic plants with different antibiotic resistances. The subcellular localizations of the interesting genes can be determined with the help of confocal microscopy. The homodimerization or heterodimerization between LRR-RLKs can be detected and confirmed by coexpression *in planta *and coimmunoprecipitation analysis. The transgenic plants can also be used to isolate protein complexes for each LRR-RLKs, which can help to dissect the complicated signaling pathways that the genes participate in. The cloned genes could be mutated directly by site-directed mutagenesis in entry clones to create kinase-inactive copies. Overexpression of kinase-inactive genes in Arabidopsis will be useful to dissect the functions of the genes by dominant negative effects, especially in the case that functional redundancy is a problem during analysis of them.

In order to clearly understand LRR-RLK function, it is necessary to characterize cytoplasmic kinase domain phosphorylation and examine the role of receptor oligomerization in initiating signaling pathways. The primary goal of this study was to generate resources for our current Arabidopsis 2010 project that is focused on mapping LRR-RLK phosphorylation sites, assessing the functions of the identified sites in plant growth and development, and examining the *in vivo *interactions of numerous LRR-RLKs. A prototype for this approach has been developed for the BRI1 and BAK1, two LRR-RLKs involved in BR signaling. For example, immunoprecipitated BRI1-FLAG protein was analyzed by liquid chromatography-tandem mass spectrometry (LC/MS/MS) and multiple *in vivo *phosphorylated Ser and Thr residues of BRI1 were identified. T-1049 and S-1044 are highly conserved activation loop residues that were shown to be essential for kinase function *in vitro *and BRI1 signaling *in planta *[33]. The interaction of BRI1 and BAK1 was studied in detail both *in vitro *and *in vivo*, and a novel mechanism of sequential transphosphorylation was developed, which helps explain the role of the BAK1 co-receptor in regulating BR signaling through BRI1 [34]. This approach, utilizing the resources developed here, is being expanded to examine the mechanisms of action of numerous LRR-RLKs across this important family of regulatory proteins.

## Conclusions

This study generated four Gateway^R^-compatible destination vectors for plant transformation and they were proved functional by overexpressing *BAK1 *to suppress *bri1-5 *mutant plant phenotype. Complementary DNA sequences of 194 Arabidopsis *LRR-RLKs *were cloned into the Gateway^R ^donor vector pDONR/Zeo^R ^and analyzed by DNA sequencing. A total of 37 isolated *LRR-RLKs *showed distinct sequences from the database prediction or previously reported sequences. Alternative RNA splicing was observed in some of them, which was thought involved in the regulation of gene functions and plant development. Experimental evidences for the annotation of these *LRR-RLKs *were provided in is study. The generated cDNA clones, expression constructs and transgenic plants are useful resources for scientific communities and will accelerate the research in this field.

## Methods

### Primer design and reverse transcriptase PCR reaction for *LRR-RLK *cloning

Coding sequences of all the predicted *LRR-RLK *genes [10] were retrieved from the database (TAIR5 release). Primer pairs for all the genes were designed according to the predicted ORF sequences. The forward primer contained partial *att*B1 sequence (5'-AAAAAGCAGGCT-3'), the start codon and 18-28 gene-specific nucleotides thereafter to yield a sequence with a T_m _value higher than 55°C. The reverse primer contained partial *att*B2 sequence (5'-AGAAAGCTGGGT-3') and 18-28 nucleotides of 3' gene specific sequence without the stop codon. To make the cloned sequence in frame with FLAG and GFP sequences in the vectors, one extra C was added before the gene specific sequence in the reverse primer.

Total RNA was extracted from whole plants, inflorescences and roots of Arabidopsis using RNeasy Plant Mini Kit (Qiagen, Valencia, CA). Messenger RNA (mRNA) was isolated from the total RNA by Oligotex mRNA Mini Kit (Qiagen). Either total RNA or mRNA was reverse transcribed into single-stranded cDNA with Superscript III reverse transcriptase (Invitrogen, Carlsbad, CA) in a 40 μl volume. Two rounds of PCR reactions were performed to generate *att*B-flanked PCR products. The first round of PCR with gene specific primers was processed with the following program: 95°C for 2 min; 30 cycles of 95°C for 15 s, 55°C for 30 s, 68°C for 4 min; 72°C for 10 min. After the first round of PCR reaction was completed, the second round of PCR was performed using *att*B1 and *att*B2 adaptors as universal primers containing *att*B1 and *att*B2 recombinational cloning sites (*att*B1 adaptor: 5'-GGGGACAAGTTTGTACAAAAAAGCAGGCT-3'; *att*B2 adaptor: 5'-GGGGACCACTTTGTACAAGAAAGCTGGGT-3') to incorporate complete *att*B1 and *att*B2 sequences into the final PCR products.

### Gel purification and *in vitro *DNA cloning

PCR products of all LRR-RLKs were subjected to agarose gel electrophoresis in 1 × TAE buffer. DNA products were purified from the DNA containing gel slices using GENECLEAN^® ^Turbo kit (Qbiogene, Irvine, CA) and PureLink™ Gel Extraction Kit (Invitrogen). Purified PCR products were eluted into 50 μl ddH_2_O. Gateway^R ^BP clonase-directed *in vitro *DNA cloning (Invitrogen) was performed between purified DNA and plasmid DNA of the Gateway^R ^donor vector pDONR/Zeo^R ^in a 5 μl volume at room temperature for approximately 16 h. The BP clonase reactions were transformed into *E.coli *DH5α competent cells and incubated overnight at 37°C for selecting positive entry clones on Luria Bertani (LB) agar plates containing 50 μg/ml zeocin (Invitrogen). Positive entry clones were picked for further analysis by colony PCR with M13 forward (5'- TGTAAAACGACGGCCAGT-3') and M13 reverse (5'- CAGGAAACAGCTATGACC-3') primers. Entry clones with positive PCR signal and correct molecular size were inoculated into 2.5 ml LB broth containing 50 μg/ml zeocin and incubated overnight at 37°C. Plasmid DNA of entry clones were isolated and analyzed by restriction enzymatic digestion. Clones with appropriate insert sizes were selected for further analyses by DNA sequencing.

After sequence verification, plasmid DNA of each entry clone was recombined into the destination vectors pB35GWF, pB35GWG, pK35GWF and pK35GWG (see below) with the help of LR clonase (Invitrogen). The LR reactions were transformed into *E.coli *DH5α competent cells and incubated overnight at 37°C for selecting positive expression clones on LB agar plates containing 50 μg/ml kanamycin. The recombinants were inoculated into 2.5 ml LB broth containing 50 μg/ml kanamycin and incubated overnight at 37°C. Plasmid DNA of each expression clone was isolated and further analyzed by restriction enzymatic digestion.

### DNA sequence analysis

All coding sequences in entry clones were sent to High-Throughput Sequencing Solutions (The University of Washington, http://www.htseq.org) for sequence analysis with M13 forward primer, M13 reverse primer and gene specific primers. Sequences from the same clone were manually assembled into contigs with the help of Seqtools http://www.seqtools.dk/. Sequences from contigs were compared by BlastN to the Arabidopsis AGI CDS dataset to examine the sequence identity and whether the sequences were from mRNA of target genes. The sequences of contigs were also compared to the AGI whole genome dataset by BlastN. The sequences of assembled contigs, the corresponding CDS sequences and genomic sequences were aligned and analyzed by Spidy http://www.ncbi.nlm.nih.gov/IEB/Research/Ostell/Spidey and GeneDoc http://www.nrbsc.org/gfx/genedoc/index.html to identify introns and exons in target genes and view the detailed sequence differences.

### Determination of *LRR-RLK *variants with reverse transcriptase PCR

Total RNA of inflorescence (including flowers and siliques) and leaf was prepared from four week old Arabidopsis Col-0 plants grown in soil with RNeasy plant mini kit (Qiagen). On column DNase I digestion of the total RNA was performed during the RNA purification process according to the manufacture's instruction to eliminate the genomic DNA contamination. Ten micrograms of total RNA were reverse transcribed to cDNA with PowerScript reverse transcriptase (Clontech, Mountain View, CA) in a 40 μl volume according to the manufacture's instruction. Same amount of cDNA equivalent to 100 ng total RNA was used to perform the primary PCR reactions for 38 *LRR-RLKs *that show potential alternative splicing of pre-mRNA. The nested PCR reactions were conducted to increase the sensitivity and specificity of the investigation of alternative splicing with variant-specific primers. The variant-specific primers were carefully designed, for example, flanking the alternatively spliced sequence if available, to further eliminate the possible genomic DNA contamination. The cDNA sequences generated from this study were not examined by RT-PCR. The used primers were listed in Table S11 in Additional file 1.

### Sequence alignment and phylogenetic analysis

Full-length cDNA sequences of six previously reported *LRR-RLKs *(At1g51830 [GenBank:AK221400], At1g75640 [GenBank:AK226809], At3g24240 [GenBank:AJ550163], At4g29990 [GenBank:X97774], At4g39270 [GenBank:AY099851], At5g67200 [GenBank:BT003370]) were retrieved from GenBank. The predicted mRNA sequences of 37 *LRR-RLKs *without experimentally produced complete coding sequences were retrieved from TAIR. The mRNA sequences of all the other 180 genes were from this study. The corresponding protein sequences were then imported into MEGA 4 [55] for multiple sequence alignment by ClustalW [56] and phylogenetic analysis by using the Neighbor-joining [57] and bootstrap [58] methods. The weighing matrix used for ClustalW alignment was BLOSUM with the penalty of gap opening 10 and gap extension 0.2. The bootstrap consensus tree was inferred from 1,000 replicates.

### Construction of Gateway^R^-compatible binary vectors

The mannopine synthase (mas) promoter (Pmas) and the coding region of glufosinate resistance (BAR) gene were PCR-amplified from pSKI015 [59] and the resulting PCR products were purified and cloned into *Hind*III/*Bam*HI digested pBlueScriptSK(+) (Stratagene, La Jolla, CA). Synonymous mutations were introduced into the BAR sequence by site-directed mutagenesis to eliminate all regularly used restriction sites including *Eco*RI, *Xho*I, *Sac*I, and *Kpn*I, resulting in pBlueScriptSK(+)-BAR. All site-directed mutagenesis reactions were carried out with PfuUltra™ High-Fidelity DNA Polymerase (Stratagene). After treatment with 10 units of *Dpn*I for 1 h at 37°C, 2 μl PCR products were transformed into *E.coli *DH5α competent cells for selecting positive colonies on LB agar plates containing 100 μg/ml ampicillin. PCR products of positive colonies were digested with *Eco*RI, *Xho*I, *Sac*I and *Kpn*I to select those with mutations. After sequence verification, the plasmid DNA of pBlueScriptSK(+)-BAR was used as template to amplify the Pmas and BAR region flanked with *Hind*III and *Bgl*II sites. PCR products were digested by *Hind*III and *Bgl*II, and cloned into *Hind*III/*Bam*HI digested pBIB-HYG-35S [60]. The resulting vector was named pBIB-BASTA-35S and the *Bam*HI restriction site in this vector was eliminated after *Bam*HI/*Bgl*II ligation. The T-DNA region of the vector was sequenced, and the resistance of transgenic plants to herbicide was confirmed by spraying with Finale (AgrEvo, Montvale, NJ).

Gateway^R^-FLAG fragments and Gateway^R^-GFP fragments were amplified from pEarleyGate 302 and pEarleyGate 103 [61] respectively by AccuPrime™ Pfx DNA Polymerase (Invitrogen). The digested and purified fragments were cloned into the *Kpn*I/*Sac*I sites of pBIB-KAN-35S [60] and pBIB-BASTA-35S to produce Gateway^R^-compatible binary vectors pK35GWF, pK35GWG, pB35GWF and pB35GWG. The T-DNA regions of the binary vectors were confirmed by DNA sequencing.

### Plant materials, growth conditions, transformation and selection

Arabidopsis Col-0 plants were grown at 22°C in a long-day condition (16 h of light and 8 h of dark) in the greenhouse. The floral dip method [62] was used to transform wild-type Arabidopsis and *bri1-5 *mutant plants [63]. *Agrobacterium tumefaciens *strain GV3101 containing each target construct was grown at 30°C for 30 h to the stationary phase. Cells were then harvested by centrifugation and resuspended in two volumes of water with 5% (w/v) sucrose and 0.03% (v/v) Silwet L-77 (Lehle Seeds, Round Rock, TX). Healthy and vigorously growing inflorescences of Arabidopsis were immersed in the above *A. tumefaciens *suspension for 30 sec for gene transformation. After treatment, plants were kept in covered flats for 1 day. All the seeds subjected to screening were treated at 4°C for 3 d before being sown on soil or agar plates. Seeds from plants dipped with constructs containing the glufosinate resistance (BAR) gene were sown directly on soil and sprayed with 1.5:1,000 (v/v) commercially available Finale (AgrEvo) in water to screen for transgenic plants with herbicide resistance. Seeds from plants dipped with constructs containing neomycin phosphotransferase II (NPTII) were grown on ½ Murashige and Skoog medium (MS) plates [64] with 50 μg/ml kanamycin, 0.6% (w/v) agar and 1% (w/v) sucrose to obtain transgenic plants with kanamycin resistance. After about 10 days on agar plates, the selected kanamycin resistant individuals were transplanted to soil.

### Western hybridization analyses

Transgenic plants harboring GFP fusion proteins were harvested after 3 weeks of growth in soil. Total proteins from leaves were prepared for Western hybridization. Membrane proteins were extracted from 11 d seedlings grown in shaking liquid culture and subjected to immunoprecipitation of FLAG-tagged fusion proteins as previously described [14,33]. Protein samples were separated on 7.5% (w/v) SDS-PAGE gel. Western hybridization analyses with GFP or FLAG antibodies were performed as previously described [14,33].

Sequence data from this study can be found in the GenBank database under accession numbers: FJ708625-FJ708818.

## Authors' contributions

JL supervised the project in which the experiments were carried out. XG, JL and SDC designed the experiments. XG and JL made the Gateway^R^-compatible destination vectors for Arabidopsis transformation. XG performed the molecular cloning and sequence analysis of *LRR-RLKs*. XG, KH, HY and TY extracted plasmid DNA and made glycerol stocks. Arabidopsis transformation and Western hybridization analyses were performed by XG, KH and HY. HL partially participated in the experiments. XG, JL and SDC wrote the manuscript. All authors read and approved the final manuscript.

## Supplementary Material

Additional file 1 **Supplemental tables and related references**. Additional file 1 contains Tables S1-S11 and references cited in Table S1. Supplemental Table S1. Arabidopsis LRR-RLKs with known functions. Supplemental Table S2. Summary of isolated *LRR-RLKs*. Supplemental Table S3. Isolated *LRR-RLKs *with the same structure as predicted in TAIR8. Supplemental Table S4. Isolated *LRR-RLKs *with different coding sequences and one continuous ORF. Supplemental Table S5. Isolated *LRR-RLKs *with different coding sequences and no continuous ORF.Supplemental Table S6. Uncloned *LRR-RLKs*.Supplemental Table S7. Detailed sequence information of isolated *LRR-RLKs *with one continuous ORF showing sequence differences. Supplemental Table S8. Detailed sequence information of isolated *LRR-RLKs *without continuous ORF. Supplemental Table S9. Isolated *LRR-RLKs *without EST sequence in TAIR database. Supplemental Table S10. Isolated *LRR-RLKs *without full-length coding sequence in TAIR database.Supplemental Table S11. Primers used to detect alternative splicing of *LRR-RLKs*. Click here for file

Additional file 2 **Cloning strategy and results**. (a) Target *LRR-RLK *sequences without stop codons are RT-PCR amplified, agarose gel purified and recombined with the pDONR/Zeo^R ^vector by BP clonase to create *pENTR-LRR-RLK *entry clones. Final expression constructs are created by performing LR clonase-mediated DNA recombination between the *pENTR-LRR-RLK *clones and the destination vectors that contain GFP or FLAG epitope tags. (a) The cloning results of the predicted *LRR-RLKs *in Arabidopsis. Click here for file

Additional file 3 **Sequence alignments of isolated LRR-RLKs displaying different coding sequences and containing one continuous ORF**. Corresponding genomic DNA sequences, predicted mRNA sequences, previously reported cDNA sequences (if available), and isolated cDNA sequences obtained from this report for each *LRR-RLK *were aligned. Sequences with differences are indicated with red boxes. Click here for file

Additional file 4 **Sequence alignments of isolated LRR-RLKs showing different coding sequences but not containing one continuous ORF**. Corresponding genomic DNA sequences, predicted mRNA sequences, previously reported cDNA sequences (if available), and isolated cDNA sequences obtained from this report for each *LRR-RLK *were aligned. Sequences with differences are indicated with red boxes. Click here for file

Additional file 5 **LRR-RLKs phylogeny based on the full-length amino acid sequences**. The previously assigned LRR subfamily names are shown on the right in black. Click here for file
