# Supplementary material for: Genome-wide cloning and sequence analysis of leucine-rich repeat receptor-like protein kinase genes in Arabidopsis thaliana
Source: BMC Genomics. 2010 Jan 11;11:19. doi: 10.1186/1471-2164-11-19 (PMC2817689; doi:10.1186/1471-2164-11-19)
Supplement: Additional file 1 — Supplemental tables and related references. Additional file 1 contains Tables S1-S11 and references cited in Table S1. Supplemental Table S1. Arabidopsis LRR-RLKs with known functions. Supplemental Table S2. Summary of isolated LRR-RLKs. Supplemental Table S3. Isolated LRR-RLKs with the same structure as predicted in TAIR8. Supplemental Table S4. Isolated LRR-RLKs with different coding sequences and one continuous ORF. Supplemental Table S5. Isolated LRR-RLKs with different coding sequences and no continuous ORF.Supplemental Table S6. Uncloned LRR-RLKs.Supplemental Table S7. Detailed sequence information of isolated LRR-RLKs with one continuous ORF showing sequence differences. Supplemental Table S8. Detailed sequence information of isolated LRR-RLKs without continuous ORF. Supplemental Table S9. Isolated LRR-RLKs without EST sequence in TAIR database. Supplemental Table S10. Isolated LRR-RLKs without full-length coding sequence in TAIR database.Supplemental Table S11. Primers used to detect alternative splicing of LRR-RLKs. [file 1471-2164-11-19-S1.DOC]

**Table S1 Arabidopsis LRR-RLKs with known functions**

| **Subfamily** | **Gene** | **Symbol** | **Functions** | **Reference** |
| --- | --- | --- | --- | --- |
| LRR I | At4g29990 | LRRPK | Light signal transduction | 1 |
| LRR II | At4g33430 | BAK1/AtSERK3 | BR signaling/Pathogen response/Cell death | 2-6 |
| LRR II | At2g13790 | BKK1/AtSERK4 | BR signaling/Pathogen response/Cell death | 2 |
| LRR II | At1g71830 | AtSERK1 | BR signaling/ Male Sporogenesis | 7-9 |
| LRR II | At1g34210 | AtSERK2 | Male Sporogenesis | 7, 8 |
| LRR II | At5g16000 | NIK1 | Antiviral defense response | 10 |
| LRR II | At3g25560 | NIK2 | Antiviral defense response | 10 |
| LRR II | At1g60800 | NIK3 | Antiviral defense response | 10 |
| LRR V | At3g13065 | SRF4 | Leaf size control | 11 |
| LRR V | At1g11130 | Scrambled/SRF9/SUB/Strubbelig | Root epidermis patterning/Organ development/Positional signaling/cell morphogenesis | 11-14 |
| LRR X | At4g39400 | BRI1 | Brassinosteroid receptor | 15 |
| LRR X | At1g55610 | BRL1 | Brassinosteroid receptor/Vascular differentiation | 16, 17 |
| LRR X | At2g01950 | BRL2/VH1 | Vascular differentiation | 18 |
| LRR X | At3g13380 | BRL3 | Brassinosteroid receptor/Vascular differentiation | 16, 17 |
| LRR X | At1G69270 | RPK1/TOAD1 | Abscisic acid signaling/embryonic pattern formation | 19-22 |
| LRR X | At3g02130 | RPK2/TOAD2 | Anther development/embryonic pattern formation | 20, 21, 23 |
| LRR X | At5g07280 | EMS1/EXS | Anther development | 24, 25 |
| LRR X | At5g48380 | BIR1 | Cell death and innate immunity | 26 |
| LRR XI | At4g20140 | GSO1 | Epidermal surface formation during embryogenesis | 27 |
| LRR XI | At5g44700 | GSO2 | Epidermal surface formation during embryogenesis | 27 |
| LRR XI | At1g75820 | CLV1 | Meristem differentiation and maintenance | 28 |
| LRR XI | At5g65700 | BAM1 | Meristem differentiation/Anther development | 29, 30 |
| LRR XI | At3g49670 | BAM2 | Meristem differentiation/Anther development | 29, 30 |
| LRR XI | At4g20270 | BAM3 | Meristem differentiation/Anther development | 29 |
| LRR XI | At2g31880 | SOBIR1 | Cell death and innate immunity | 26 |
| LRR XI | At4g28490 | HAESA | floral organ abscission | 31 |
| LRR XI | At3g19700 | IKU2 | Seed size | 32 |
| LRR XI | At5g61480 | PXY/TDR | Procambium polar cell division/ vascular stem cell fate | 33, 34 |
| LRR XII | At5g46330 | FLS2 | Pathogen response | 35 |
| LRR XII | At5g20480 | EFR | Pathogen response | 36 |
| LRR XIII | At1g31420 | FEI1 | Cell Wall Biosynthesis | 37 |
| LRR XIII | At2g35620 | FEI2 | Cell Wall Biosynthesis | 37 |
| LRR XIII | At2g26330 | ERECTA | Organ growth/ Stomatal patterning and differentiation | 38, 39 |
| LRR XIII | At5g62230 | ERL1 | Stomatal patterning and differentiation | 38, 39 |
| LRR XIII | At5g07180 | ERL2 | Stomatal patterning and differentiation | 38, 39 |

**Table S2 Summary of isolated *LRR-RLKs***

| **Categories** | **Number of genes** |
| --- | --- |
| All isolated *LRR-RLKs* | 194 |
| With the same structure as predicted | 157 |
| Variants with the predicted start and stop codons | 23 |
| Variants with different start and stop codons | 14 |
| No EST sequence in TAIR8 database | 12 |
| No full-length cDNA in TAIR8 database | 70 |
| Full-length cDNA available from ABRC | 50 |
| Full-length cDNA not available from ABRC | 74 |

**Table S3 Isolated *LRR-RLKs* with the same structure as predicted in TAIR8**

| At1g07550 At1g07650 At1g08590 At1g09970 At1g11130 At1g12460 At1g17750 At1g24650 At1g25320 At1g27190 At1g28440 At1g29720 At1g29750 At1g34210 At1g34420 At1g48480 At1g50610 At1g51800 At1g51810 At1g51820 At1g53730 At1g55610 At1g56145 At1g60630 At1g60800 At1g62950 At1g63430 At1g66150 At1g66830 At1g67510 At1g67720 At1g68400 At1g69270 At1g69990 At1g71830 At1g72180 At1g72300 At1g72460 At1g73080 At1g74360 At1g75820 At1g78980 At1g79620 At2g01210 At2g01820 At2g01950 At2g02220 At2g07040 At2g13790 At2g13800 At2g14510 At2g16250 At2g19190 At2g20850 At2g23300 At2g23950 At2g24230 At2g25790 At2g26330 At2g26730 At2g27060 At2g28960 At2g28990 At2g31880 At2g33170 At2g35620 At2g36570 At2g37050 At2g41820 At2g42290 At2g45340 At3g02130 At3g02880 At3g03770 At3g08680 At3g13065 At3g13380 At3g14350 At3g14840 At3g17840 At3g20190 At3g23750 At3g25560 At3g28040 At3g28450 At3g42880 At3g46330 At3g47090 At3g47570 At3g47580 At3g49670 At3g50230 At3g51740 At3g56370 At3g57830 At4g03390 At4g08850 At4g18640 At4g20140 At4g20450 At4g20790 At4g22130 At4g22730 At4g23740 At4g28490 At4g28650 At4g29450 At4g30520 At4g31250 At4g33430 At4g34220 At4g36180 At4g37250 At4g39400 At5g01890 At5g01950 At5g05160 At5g06820 At5g07180 At5g07280 At5g10020 At5g10290 At5g16000 At5g16590 At5g16900 At5g20480 At5g20690 At5g24100 At5g25930 At5g41180 At5g43020 At5g45780 At5g45800 At5g46330 At5g48380 At5g48740 At5g48940 At5g49660 At5g49760 At5g49770 At5g51350 At5g51560 At5g53320 At5g53890 At5g56040 At5g58150 At5g58300 At5g59670 At5g61480 At5g62230 At5g62710 At5g63410 At5g63710 At5g63930 At5g65700 At5g65710 At5g67280 |
| --- |

**Table S4 Isolated *LRR-RLKs* with different coding sequences and one continuous ORF**

| At1g05700 At1g07560 At1g14390 At1g31420 At1g34110 At1g51880 At1g51890 At1g53430 At2g02780 At3g21340 At3g24660 At3g56100 At4g20270 At4g20940 At4g26540 At4g29180 At5g14210 At5g35390 At5g37450 At5g45840 At5g59650 At5g59680 At5g65240 |
| --- |

**Table S5 Isolated *LRR-RLK*s with different coding sequences and no continuous ORF**

| At1g06840 At1g29730 At1g35710 At1g51860 At1g53420 At1g53440 At1g56120 At1g56130 At1g56140 At2g28970 At3g46370 At4g29990 At5g07150 At5g44700 |
| --- |

**Table S6 Uncloned *LRR-RLK*s**

| At1g17230 At1g29740 At1g49100 At1g51790 At1g51830 At1g51850 At1g51870 At1g51910 At1g64210 At1g75640 At2g04300 At2g14440 At2g15300 At2g19210 At2g19230 At2g24130 At3g19700 At3g24240 At3g46340 At3g46350 At3g46400 At3g46420 At3g47110 At3g53590 At4g39270 At5g39390 At5g49780 At5g59660 At5g67200 |
| --- |

**Table S7 Detailed sequence information of isolated *LRR-RLKs* with one continuous ORF showing sequence differences**

| AGI | Old Acc.^a^ | New Acc^b^. | ORF in bp^c^ | | Differences^d^ |
| --- | --- | --- | --- | --- | --- |
|  |  |  | Old | New |  |
| At1g05700 | NM_100450 | FJ708625 | 2,529 | 2,556 | 5 bp and 22 bp added at 1,620 to the 7^th^ and 8^th^ exons respectively |
| At1g07560 | NM_100630 | FJ708628 | 2,568 | 2,613 | 12 bp and 33 bp added at 1,461 to the 7^th^ and 8^th^ exons respectively |
| At1g14390 | NM_101306 | FJ708634 | 2,241 | 2,184 | 57 bp deleted at 1,810 from the 5^th^ exon |
| At1g31420 | NM_102881  AK226234 | FJ708643 | 1,776  1,775 | 1,773 | 3 bp deleted at 1,178 from the 11th exon; AK226234 lost 1 bp at 1,708, resulting in truncated ORF |
| At1g34110 | NM_103134 | FJ708644 | 3,135 | 3,159 | 24 bp added at 2,762 to the 1^st^ exon |
| At1g51880 | NM_104068 | FJ708654 | 2,640 | 2,616 | 24 bp deleted at 1,389 to the 7^th^ exon |
| At1g51890 | NM_104069 | FJ708655 | 2,664 | 2,484 | 49 bp added at 1,184 to the 4^th^ exon; the 5^th^ and 6^th^ exons lost with 97 bp deletion at 1,184 and 72 bp deletion at 1,281, respectively; 6 bp added at 1,568 to the 9^th^ exon; 66 bp deleted at 1,938, separating the 10^th^ exon to 2 exons |
| At1g53430 | NM_104221 | FJ708657 | 3,090 | 3,114 | 24 bp added at 862 to the 11^th^ exon |
| At2g02780 | NM_126333 | FJ708689 | 2,259 | 2,226 | 33 bp deleted at 1,262 from the 2^nd^ exon |
| At3g21340 | NM_113029 | FJ708725 | 2,640 | 2,697 | 57 bp added at 1,658 to the 9^th^ exon |
| At3g24660 | NM_113377 | FJ708727 | 2,022 | 1,869 | 153 bp deleted at 638, splitting the first exon to 2 exons |
| At3g56100 | NM_115468 | FJ708740 | 2,157 | 2,352 | 111 bp added at 733 and 83 bp added at 1,473, merging the first 3 exons to one exon; T at 827 is not found in the isolated cDNA sequence and A at 1,562 in the isolated sequence is not found in the Arabidopsis genome |
| At4g20270 | NM_118146 | FJ708747 | 2,976 | 2,889 | 87 bp deleted at 1,142, splitting the first exon to 2 exons |
| At4g20940 | NM_118212 | FJ708750 | 2,931 | 3,111 | 83 bp added at 2,445 to the 2^nd^ exon and 97 bp added at 2,445 to the 3^rd^ exon, spanning the 2^nd^ intron |
| At4g26540 | NM_118787 | FJ708754 | 3,267 | 3,273 | 3 bp added at 1,412 and 1,484 respectively. Both C at 1,412 and 1,484 in the isolated sequence are not found in the Arabidopsis genome |
| At4g29180 | NM_119062 | FJ708757 | 2,733 | 2,739 | 46 bp added at 1,205 to the 3^rd^ exon and 40 bp deleted at 1,205 from the 4^th^ exon |
| At5g14210 | NM_121425 | FJ708776 | 2,436 | 2,325 | 34 bp deleted at 1,966 and 77 bp deleted at 2,027 from the 5^th^ exon |
| At5g35390 | NM_122930 | FJ708784 | 1,971 | 1,986 | 15 bp added at 1,332 to the 2^nd^ exon |
| At5g37450 | NM_123104 | FJ708785 | 2,805 | 2,877 | 144 bp added at 247 to form two new exons; 72 bp deleted at 1,499, splitting the 12^th^ exon |
| At5g45840 | NM_123952 | FJ708791 | 2,004 | 2,085 | 81 bp added at 603 to form a new exon |
| At5g59650 | NM_125357 | FJ708806 | 2,676 | 2,652 | 22 bp added at 1,077 to the 3^rd^ exon; 46 bp deleted at 1,077 from the 4^th^ exon |
| At5g59680 | NM_125360 | FJ708808 | 2,646 | 2,661 | 15 bp added at 2,256 to the last exon |
| At5g65240 | NM_125922  AY059844 | FJ708815 | 1,851  1,837 | 1,821 | In AY059844 and the isolated sequence, 30 bp deleted at 774 from the 9^th^ exon; 16 bp added in AY059844 at 561 to the 7^th^ exon |

1. Accession numbers for database sequences of predicted and previously submitted cDNA clones.
2. Accession numbers for experimentally produced cDNA sequences in this report.
3. Nucleotide positions start from the A of the start codon and the count for all ORFs doesn’t include the stop codon.
4. New sequences compared with old sequences. The positions of insertion and deletion are shown according to the old sequence.

**Table S8 Detailed sequence information of isolated *LRR-RLKs* without continuous ORF**

| AGI | Old Acc.^a^ | New Acc^b^. | ORF in bp^c^ | | Differences^d^ |
| --- | --- | --- | --- | --- | --- |
|  |  |  | Old | New |  |
| At1g06840 | NM_100561 | FJ708626 | 2,817 | 3,137 | 309 bp added at 75 to exon 1; 4 bp added at 75 to exon 2; 7 bp added at 2,041 to exon 17 |
| At1g29730 | NM_102713 | FJ708641 | 2,907 | 3,202 | 98 bp added at 131, merging the first 2 exons; 229 bp added at 1,850 to exon 19; 29 bp deleted at 1,850 from exon 20; 3 bp deleted at 2,180 from exon 22 |
| At1g35710 | NM_103273 | FJ708647 | 3,360 | 3,349 | 95 bp deleted at 2,473, splitting exon 1 to two exons; 84 bp added at 3,077 merging the two exons |
| At1g51860 | NM_104066 | FJ708653 | 2,670 | 2,626 | 44 bp deleted at 624 from exon 3 |
| At1g53420 | NM_104220 | FJ708656 | 2,859 | 3,000 | 66 bp added at 204 to exon 2; 75 bp added at 490, merging exon 6 and exon 7 |
| At1g53440 | NM_104222 | FJ708658 | 3,105 | 3,029 | 76 bp added at 101 to exon 2 |
| At1g56120 | NM_104490 | FJ708661 | 3,135 | 3,228 | 26 bp added at 310 to exon 4; 20 bp deleted at 356 from exon 4; 128 bp added at 1,017 to exon 13; 129 bp deleted at 1,190 from exon 16; 88 bp added at 1,895, merging exon 17 and exon 18 |
| At1g56130 | NM_104491 | FJ708662 | 3,096 | 3,025 | 71 bp deleted at 1,526 from exon 17 |
| At1g56140 | NM_104492  BT011697 | FJ708663 | 3,096  668 | 3,042 | 3 bp added at 814 to exon 10; 72 bp deleted at 1,521 from exon 17; 5 bp deleted at 1,648 from exon 18; 20 bp added at 2,006 to exon 20  BT011697 missed sequences from exon 6 to exon 23 and part of exon 5 and exon 24 |
| At2g28970 | NM_128456 | FJ708705 | 2,358 | 2,731 | 289 bp added at 264, merging exon 2 and exon 3; 84 bp added at 794, merging exon 4 and exon 5 |
| At3g46370 | NM_114504 | FJ708733 | 2,379 | 2,330 | 49 deleted at 1,330 from exon 7 |
| At4g29990 | NM_119145  X97774 | FJ708759 | 2,628  2,628 | 2,387 | 241 bp deleted at 257, splitting exon 2 to two exons  X97774 showed the same sequence as the prediction |
| At5g07150 | NM_120797 | FJ708771 | 1,659 | 1,900 | 78 bp added at 110, merging exon 1 and exon 2; 20 bp added at 240 to exon 2; the entire exon 3 (72 bp) deleted at 240; 37 bp deleted from exon 4; 90 bp added at 841, 76 bp added at 1,033, 86 bp added at 1,160, respectively, merging exon 4, exon 5, exon 6 and exon 7 |
| At5g44700 | NM_123837 | FJ708788 | 3,756 | 887 | 2,868 bp deleted at 515 from exon 1; 1 bp deleted at 515 from exon 2 |

1. Accession numbers for database sequences of predicted and previously submitted cDNA clones.
2. Accession numbers for experimentally produced cDNA sequences in this report.
3. Nucleotide positions start from the A of the start codon and the count for all ORFs doesn’t include the stop codon.
4. New sequences compared with old sequences. The positions of insertion and deletion are shown according to the old sequence.

**Table S9 Isolated *LRR-RLKs* without EST sequence in TAIR database**

| At1g05700 At1g07560 At1g51880 At1g69990 At1g72460 At2g14510  At3g46370 At4g20450 At4g20790 At5g16900 At5g20690 At5g37450 |
| --- |

**Table S10 Isolated *LRR-RLKs* without full-length coding sequence in TAIR database**

| At1g05700 At1g06840 At1g07560 At1g07650 At1g14390 At1g17750 At1g24650 At1g29730 At1g34110 At1g34420 At1g35710 At1g51820 At1g51860 At1g51880 At1g51890 At1g53420 At1g53430 At1g53440 At1g55610 At1g56120 At1g56130 At1g56140 At1g56145 At1g62950 At1g67510 At1g69990 At1g72460 At1g75820 At2g01820 At2g02220 At2g02780 At2g14510 At2g16250 At2g23300 At2g28960 At2g28970 At2g28990 At2g35620 At3g08680 At3g21340 At3g23750 At3g28040 At3g46370 At3g47090 At3g50230 At3g56100 At4g18640 At4g20450 At4g20790 At4g20940 At4g28650 At4g29180 At4g39400 At5g05160 At5g07150 At5g14210 At5g16900 At5g20690 At5g35390 At5g37450 At5g43020 At5g44700 At5g45780 At5g45840 At5g49770 At5g59650 At5g59680 At5g61480 At5g63930 At5g65710 |
| --- |

Table S11 Primers used to detect alternative splicing of *LRR-RLKs*

| Genes | Primers | Primers for primary PCR | Primers for nested PCR |
| --- | --- | --- | --- |
| At1g05700 | 1g05700NestAsF, GGTTCAGTTCCATCCGAGTTAT  1g05700PB2, ATAGTTCTTGTTACTCTCTTCTCTT  1g05700oldAsF, AAACCGGAGAAACAATCCAATG  1g05700AsR, CATCTCCAACAGAACAACTCCA | 1g05700NestAsF  1g05700PB2 | 1g05700oldAsF  1g05700AsR |
| At1g06840 | 1g06840PB1, ATGGTTTTGACGGAAGAAGGTGGT  1g06840NestAsR, GGTTCGGTATGCTGCTAAGATC  1g06840oldAsF, CGCTCTTCCGTCGGGCTTTG | 1g06840PB1  1g06840NestAsR | 1g06840oldAsF  1g06840NestAsR |
| At1g07560 | 1g07560NestAsF, GGTGGAGTACCTGAATTTCTAGC 1g07560PB2, ACGTGCCTTGGGGTTCACATCT  1g07560AsR, GCTTAGCTTGTAATTGATCGTCC  1g07560oldAsF, CAAAGTGAAAAATGGTTCATGC | 1g07560NestAsF  1g07560PB2 | 1g07560oldAsF  1g07560AsR |
| At1g14390 | 1g14390NestAsF, AGGTGTTGAAATGGCCTCAGAG  1g14390oldAsF, TAACCTTCTGGGGGAATTTCAG  1g14390PB2, TAGTTCTGAACCACCAAGCCCGAG | 1g14390NestAsF  1g14390PB2 | 1g14390oldAsF  1g14390PB2 |
| At1g29730 | 1g29730PB1, ATGTCTGCAGCTTACAATCTCATGAT  1g29730oldAsF, GCACCCAGATGAAGTGGAAG  1g29730AsR, CTCTAAGGCCACTCGCATAGAG | 1g29730PB1  1g29730AsR | 1g29730oldAsF  1g29730AsR |
| At1g31420 | 1g31420AsR, TGGACTCGGAGACACACACTGT  1g31420NestAsF, GGATGATGGCAAAGTCTTTGCA  1g31420oldAsF, GCCTTGATGAAGCACTTCATGTAG  1g31420PB2, ATCAGAGCTGGAATCATAAAATTCGCT | 1g31420NestAsF  1g31420PB2 | 1g31420oldAsF  1g31420AsR |
| At1g34110 | 1g34110NestAsF, AGATTGCGATTGGAGCTGCTCA  1g34110oldAsF, CCATGTCTCGTGTTGCTGAATAC  1g34110PB2, TGAAGAAGAAGGCTTGATAAGAGGCT | 1g34110NestAsF  1g34110PB2 | 1g34110oldAsF  1g34110PB2 |
| At1g35710 | 1g35710NestAsF, TGGCAACCTTGTTGTGTGGA  1g35710oldAsF, CATATTCAGCGTTGATGGCAA  1g35710PB2, AGAAAATGTAGTGGAGATTGACAACA | 1g35710NestAsF  1g35710PB2 | 1g35710oldAsF  1g35710PB2 |
| At1g51860 | 1g51860NestAsR, CTGAACCAACGTAGACCACCAT  1g51860oldAsR, ACGGTCATGTATGTCCTCATCA  1g51860PB1, ATGAAATCTCTTCACTGGTTTTTG | 1g51860PB1  1g51860NestAsR | 1g51860PB1  1g51860oldAsR |
| At1g51880 | 1g51880NestAsF, CCTAAGTCTTATCAGTGGGAAGGT  1g51880oldAsF, AAAGCTTAATGTTTTCATTTGCAG  1g51880AsR, ATGGACCATAGGAGGCGTACAT  1g51880PB2, TCTGGCTCCAGGGGAAAATTCGGA | 1g51880NestAsF  1g51880PB2 | 1g51880oldAsF  1g51880AsR |
| At1g51890 | 1g51890NestAsF, CCTCTTATCAACGGCCTTGAGA  1g51890oldAsF, CTATTAGGAACTTGAGTGGAAGCA  1g51890AsR, GCTAAGTTGTCTCCATCATCACA  1g51890PB2, CCTAGCTAAAGGGGAAAAATCAGA | 1g51890NestAsF  1g51890PB2 | 1g51890oldAsF  1g51890AsR |
| At1g53420 | 1g53420PB1, ATGTCGTTAAATCGGTTTCTCTTCAC  1g53420NestAsR, AGTGTTAGGGATGGCTCCACTT  1g53420OldAsR, GTTATTCGAGCTAAGAATCATTTGT | 1g53420PB1  1g53420NestAsR | 1g53420PB1  1g53420OldAsR |
| At1g53430 | 1g53430NestAsF, GGAAACTGGACTCTGCTGGAGA  1g53430NestAsR, AACTCCACCTGCTCTCTCTGCT  1g53430oldAsF, AAAATGAAACGATTGGGACCTA  1g53430AsR, TTCCCTTGGACATAAATGTCGA | 1g53430NestAsF  1g53430NestAsR | 1g53430oldAsF  1g53430AsR |
| At1g53440 | 1g53440PB1, ATGGGTTTCTTTTTCTCGACCCGGA  1g53440oldAsF, CGAAAGAACTTCTTGTTTGGAC  1g53440NestAsR, AGGAGAAGTTGGTCCACGCA | 1g53440PB1  1g53440NestAsR | 1g53440oldAsF  1g53440NestAsR |
| At1g56120 | 1g56120NestAsF, CTTCATGGGTCAGCTTACCAGA  1g56120oldAsF, GACTTTCGCTATTGACTGGTGT  1g56120NestAsR, ATGGTGGCCTATTAGCCACAGT | 1g56120NestAsF  1g56120NestAsR | 1g56120oldAsF  1g56120NestAsR |

| At1g56130 | 1g56130NestAsF, ATGGGCAGCTAGTAGTGTAGGA  1g56130oldAsF, GGAGGATATACAGTAACCCTTCAG  1g56130NestAsR, ACAAATTGTCCCTTCCCTTGA | 1g56130NestAsF  1g56130NestAsR | 1g56130oldAsF  1g56130NestAsR |
| --- | --- | --- | --- |
| At1g56140 | 1g56140NestAsF, GAGATGGGCAGCCAGTAGTG  1g56140NestAsR, ACAAATTGTCCCTTCCCTTGTC  1g56140oldAsR, GGCTGTGGTGTCATCAAATCTG  1g56140preAsF, GGAGGCTATACCGTCACACTTC  1g56140PB1, ATGCTCAGGCTATGGCGGTATCTG  1g56140PB2, TCTTCCCTCATTCATCTGGGCTC | For BT011697:  1g56140PB1  1g56140PB2  For NM_104492:  1g56140NestAsF  1g56140NestAsR | For BT011697:  1g56140PB1  1g56140oldAsR  For NM_104492:  1g56140preAsF  1g56140NestAsR |
| At2g02780 | 2g02780NestAsF, GAAGTGAACAACAGCAACAATG  2g02780oldAsF, CCATTAACAGGATCTTTCTGAGTG  2g02780AsR, GCAGCAACTACTTTCCCTGTGA  2g02780PB2, TTCATATATTGCTTTCATGGATGAT | 2g02780NestAsF  2g02780PB2 | 2g02780oldAsF  2g02780AsR |
| At2g28970 | 2g28970PB1, ATGATGAGCCATCTTTTGTTGGCCA  2g28970oldAsF, AGGAAAGCGTAATTGCTCACTG  2g28970NestAsR, TCTTGAGGGAGACATGGATCTC | 2g28970PB1  2g28970NestAsR | 2g28970oldAsF  2g28970NestAsR |
| At3g21340 | 3g21340NestAsF, TGCGTTTGTGGTTGTTCTTGGA  3g21340oldAsF, AGACATCAAACAGTCAAGAGTCG  3g21340AsR, CACAAGTTGGTTTGTGATGATCT  3g21340PB2, ACGAGCATCAGGGGTAGCTCCA | 3g21340NestAsF  3g21340PB2 | 3g21340oldAsF  3g21340AsR |
| At3g24660 | 3g24660NestAsF, GAGCTTGGTTACACTTCTTCTCTC  3g24660oldAsF, AGGTCTTGTTCCTGAGGGTTTA  3g24660AsR, TCGCTATTCCAAGTGCAATCTT  3g24660PB2, AAATGGAGTTTCGGCGTCGCTC | 3g24660NestAsF  3g24660PB2 | 3g24660oldAsF  3g24660AsR |
| At3g46370 | 3g46370NestAsF, TGACAGGAGTAGTGCCCGAATT  3g46370oldAsF, GATTCTCATTTTTCTGTTCAGAAAG  3g46370NestAsR, ACTCATCTCAGCCAACCGACA | 3g46370NestAsF  3g46370NestAsR | 3g46370oldAsF  3g46370NestAsR |
| At3g56100 | 3g56100NestAsF, TGATGGACCAATGGCGTTTACT  3g56100oldAsF, CCAAAAGTCAAAAAGAGAGAGAAG  3g56100PB2, TTGACTTGTGGAAGCAGAAGCTT | 3g56100NestAsF  3g56100PB2 | 3g56100oldAsF  3g56100PB2 |
| At4g20270 | 4g20270NestAsF, TTCAACAGACTACACGGCGAGA  4g20270NestAsR, TCGGGTTGTTCTTTCTCATTCT  4g20270oldAsF, ACAACTTCTTGTTCGGTCCTCT  4g20270AsR, TTCCAAGGAATGACGTGTTGTT | 4g20270NestAsF  4g20270NestAsR | 4g20270oldAsF  4g20270AsR |
| At4g20940 | 4g20940NestAsF, TCTTACAGGGCAACGCTGGA  4g20940oldAsF, GTGACTCTCCGAGGAGCTGTT  4g20940PB2, AATAGAAGAAAGATCTTCGTAAATGGT | 4g20940NestAsF  4g20940PB2 | 4g20940oldAsF  4g20940PB2 |
| At4g29180 | 4g29180NestAsF, TCTGTTCAGAAGACCGCAGAGT  4g29180NestAsR, TCCAACCCTTGTGCAGAGTCT  4g29180oldAsF, TTGCTGATGAGCTTGTTTCTGT  4g29180AsR, ACCTGGGATGATGATGATGATG | 4g29180NestAsF  4g29180NestAsR | 4g29180oldAsF  4g29180AsR |
| At4g29990 | 4g29990NestAsF, TCTCTTGAAAGGCAGTTCCAA  4g29990oldAsF, CACCTCCATCAGACCATATTCATG  4g29990AsR, AGAATTGTCCACTGGAACACATG | 4g29990NestAsF  4g29990AsR | 4g29990oldAsF  4g29990AsR |
| At4g31250 | 4g31250PB1, ATGACCCGTGATGACAAATTCCCGA  4g31250oldAsF, TGTTCAAACGGCTCCGCGGTCT  4g31250AsR, TGAGCACTACGACGGCGAGGAT | 4g31250PB1  4g31250AsR | 4g31250oldAsF  4g31250AsR |
| At5g01950 | 5g01950NestAsF, ATGGTGTTCCCTCAAAGACTCT  5g01950oldAsF, ACCGGCCAGATTCCAGCTGCTA  5g01950AsR, GATATGCTTCCATTAGTGCAGATC | 5g01950NestAsF  5g01950AsR | 5g01950oldAsF  5g01950AsR |
| At5g07150 | 5g07150PB1, ATGAGTTCCGATCAACGGTGGAGA 5g07150oldAsF, CAAGGAACACTTGCACCTGA  5g07150NestAsR, CTGAGGATGTGGAGCCTATGA | 5g07150PB1  5g07150NestAsR | 5g07150oldAsF  5g07150NestAsR |
| At5g14210 | 5g14210NestAsF, ACTCACACACCAGTCTCTCCTCT  5g14210oldAsR, GGATTAGATTCTCATAAATGCCAT  5g14210PB2, CGACGACGTATCAGATTTGCGTT | 5g14210NestAsF  5g14210PB2 | 5g14210NestAsF  5g14210oldAsR |
| At5g35390 | 5g35390NestAsF, GGTCGTGAAGAGGTTCAAGCA  5g35390oldAsF, GGCTATTAATCTTCATAGAAAACCA  5g35390PB2, TGCAAAGCTGATACTCTCGCATGA | 5g35390NestAsF  5g35390PB2 | 5g35390oldAsF  5g35390PB2 |
| At5g37450 | 5g37450NestAsF, GAGGTTCAACGTATTGTGGACT  5g37450PB2, GCGCGGTGCGATGGAGGGAAT  5g37450oldAsF, TCTTCTACTCATCTTTGTGCATCA  5g37450AsR, CAGCTTCTGTGTGCAGATACAG | 5g37450NestAsF  5g37450PB2 | 5g37450oldAsF  5g37450AsR |
| At5g44700 | 5g44700NestAsF, GACACTTGGAACAATCAGACATAG  5g44700oldAsF, AGCACTTGGGTTGGCTCAAG  5g44700PB2, TTTATCGGTATCAGTTTGCATCTC | 5g44700NestAsF  5g44700PB2 | 5g44700oldAsF  5g44700PB2 |
| At5g45840 | 5g45840NestAsF, GCTGATGTCCTCGACTGTGTCA  5g45840PB2, TGTAGCTTCAGAGGATAAGATCTCA  5g45840oldAsF, AACTTGGATACTGGGTGCGA  5g45840AsR, TATCAATCCTTCTGCGGTAAGT | 5g45840NestAsF  5g45840PB2 | 5g45840oldAsF  5g45840AsR |
| At5g59650 | 5g59650NestAsF, CTCCAGTGACATGCAAAGGAGA  5g59650NestAsR, CTAGTGCAGCCTCCAGAGCTA  5g59650oldAsF, CACCTGCTAATTCTCACATCCT  5g59650AsR, CCATGACAAACAACACCGAATC | 5g59650NestAsF  5g59650NestAsR | 5g59650oldAsF  5g59650AsR |
| At5g59680 | 5g59680NestAsF, AAGGAGGTAAACCCATCGTCA  5g59680oldAsF, TACCTTGATCCAGATCGGTTG  5g59680PB2, TCTTGCTCTGGGAATCATCTCA | 5g59680NestAsF  5g59680PB2 | 5g59680oldAsF  5g59680PB2 |
| At5g65240 | 5g65240NestAsF, TGGTTCTATCCCGGATTCACTT  5g65240PB2, TCTTCCACCAGATAATTCAATAGCA  5g65240PreAsF, CTTTAAAAAAGGTTTGATTTCAGG 5g65240AsR1, CATTGTTCCTCGGACCTGAGT  5g65240oldAsF, AAATCCCAAAATACAATCTTTCAA  5g65240AsR, ATTCTGCATGAAAGGATACACCA | 5g65240NestAsF  5g65240PB2 | For NM_125922:  5g65240PreAsF  5g65240AsR1  For AY059844:  5g65240oldAsF  5g65240AsR |

**Supplemental references:**

1. Deeken R, Kaldenhoff R: **Light-repressible receptor protein kinase: a novel photo-regulated gene from *Arabidopsis thaliana*.** *Planta* 1997, **202:**479-486.

2. He K, Gou X, Yuan T, Lin H, Asami T, Yoshida S, Russell SD, Li J: **BAK1 and BKK1 regulate brassinosteroid-dependent growth and brassinosteroid-independent cell-death pathways.** *Curr Biol* 2007, **17:**1109-1115.

3. Heese A, Hann DR, Gimenez-Ibanez S, Jones AM, He K, Li J, Schroeder JI, Peck SC, Rathjen JP: **The receptor-like kinase SERK3/BAK1 is a central regulator of innate immunity in plants.** *P Natl Acad Sci USA* 2007, **104:**12217-12222.

4. Kemmerling B, Schwedt A, Rodriguez P, Mazzotta S, Frank M, Qamar SA, Mengiste T, Betsuyaku S, Parker JE, Mussig C, Thomma BP, Albrecht C, de Vries SC, Hirt H, Nurnberger T: **The BRI1-Associated Kinase 1, BAK1, has a brassinolide-independent role in plant cell-death control.** *Curr Biol* 2007, **17:**1116-1122.

5. Li J, Wen J, Lease KA, Doke JT, Tax FE, Walker JC: **BAK1, an Arabidopsis LRR receptor-like protein kinase, interacts with BRI1 and modulates brassinosteroid signaling.** *Cell* 2002, **110:**213-222.

6. Nam KH, Li J: **BRI1/BAK1, a receptor kinase pair mediating brassinosteroid signaling.** *Cell* 2002, **110:**203-212.

7. Albrecht C, Russinova E, Hecht V, Baaijens E, de Vries S: **The *Arabidopsis thaliana* SOMATIC EMBRYOGENESIS RECEPTOR-LIKE KINASES1 and 2 control male sporogenesis.** *Plant Cell* 2005, **17:**3337-3349.

8. Colcombet J, Boisson-Dernier A, Ros-Palau R, Vera CE, Schroeder JI: **Arabidopsis SOMATIC EMBRYOGENESIS RECEPTOR KINASES1 and 2 are essential for tapetum development and microspore maturation.** *Plant Cell* 2005, **17:**3350-3361.

9. Karlova R, Boeren S, Russinova E, Aker J, Vervoort J, de Vries S: **The Arabidopsis SOMATIC EMBRYOGENESIS RECEPTOR-LIKE KINASE1 protein complex includes BRASSINOSTEROID-INSENSITIVE1.** *Plant Cell* 2006, **18:**626-638.

10. Fontes EP, Santos AA, Luz DF, Waclawovsky AJ, Chory J: **The geminivirus nuclear shuttle protein is a virulence factor that suppresses transmembrane receptor kinase activity.** *Gene Dev* 2004, **18:**2545-2556.

11. Eyuboglu B, Pfister K, Haberer G, Chevalier D, Fuchs A, Mayer KF, Schneitz K: **Molecular characterisation of the STRUBBELIG-RECEPTOR FAMILY of genes encoding putative leucine-rich repeat receptor-like kinases in *Arabidopsis thaliana*.** *BMC Plant Biol* 2007, **7:**16.

12. Chevalier D, Batoux M, Fulton L, Pfister K, Yadav RK, Schellenberg M, Schneitz K: **STRUBBELIG defines a receptor kinase-mediated signaling pathway regulating organ development in Arabidopsis.** *P Natl Acad Sci USA* 2005, **102:**9074-9079.

13. Kwak SH, Shen R, Schiefelbein J: **Positional signaling mediated by a receptor-like kinase in Arabidopsis.** *Science* 2005, **307:**1111-1113.

14. Yadav RK, Fulton L, Batoux M, Schneitz K: **The Arabidopsis receptor-like kinase STRUBBELIG mediates inter-cell-layer signaling during floral development.** *Dev Biol* 2008, **323:**261-270.

15. Li J, Chory J: **A putative leucine-rich repeat receptor kinase involved in brassinosteroid signal transduction.** *Cell* 1997, **90:**929-938.

16. Cano-Delgado A, Yin Y, Yu C, Vafeados D, Mora-Garcia S, Cheng JC, Nam KH, Li J, Chory J: **BRL1 and BRL3 are novel brassinosteroid receptors that function in vascular differentiation in Arabidopsis.** *Development* 2004, **131:**5341-5351.

17. Zhou A, Wang H, Walker JC, Li J: **BRL1, a leucine-rich repeat receptor-like protein kinase, is functionally redundant with BRI1 in regulating Arabidopsis brassinosteroid signaling.** *Plant J* 2004, **40:**399-409.

18. Clay NK, Nelson T: **VH1, a provascular cell-specific receptor kinase that influences leaf cell patterns in Arabidopsis.** *Plant Cell* 2002, **14:**2707-2722.

19. Hong SW, Jon JH, Kwak JM, Nam HG: **Identification of a receptor-like protein kinase gene rapidly induced by abscisic acid, dehydration, high salt, and cold treatments in *Arabidopsis thaliana*.** *Plant Physiol* 1997, **113:**1203-1212.

20. Nodine MD, Tax FE: **Two receptor-like kinases required together for the establishment of Arabidopsis cotyledon primordia.** *Dev Biol* 2008, **314:**161-170.

21. Nodine MD, Yadegari R, Tax FE: **RPK1 and TOAD2 are two receptor-like kinases redundantly required for Arabidopsis embryonic pattern formation.** *Dev Cell* 2007, **12:**943-956.

22. Osakabe Y, Maruyama K, Seki M, Satou M, Shinozaki K, Yamaguchi-Shinozaki K: **Leucine-rich repeat receptor-like kinase1 is a key membrane-bound regulator of abscisic acid early signaling in Arabidopsis.** *Plant Cell* 2005, **17:**1105-1119.

23. Mizuno S, Osakabe Y, Maruyama K, Ito T, Osakabe K, Sato T, Shinozaki K, Yamaguchi-Shinozaki K: **Receptor-like protein kinase 2 (RPK 2) is a novel factor controlling anther development in *Arabidopsis thaliana*.** *Plant J* 2007, **50:**751-766.

24. Jia G, Liu X, Owen HA, Zhao D: **Signaling of cell fate determination by the TPD1 small protein and EMS1 receptor kinase.** *P Natl Acad Sci USA* 2008, **105:**2220-2225.

25. Zhao DZ, Wang GF, Speal B, Ma H: **The excess microsporocytes1 gene encodes a putative leucine-rich repeat receptor protein kinase that controls somatic and reproductive cell fates in the Arabidopsis anther.** *Gene Dev* 2002, **16:**2021-2031.

26. Gao M, Wang X, Wang D, Xu F, Ding X, Zhang Z, Bi D, Cheng YT, Chen S, Li X *et al*: **Regulation of cell death and innate immunity by two receptor-like kinases in Arabidopsis**. *Cell Host Microbe* 2009, **6**(1):34-44.

27. Tsuwamoto R, Fukuoka H, Takahata Y: ***GASSHO1* and *GASSHO2* encoding a putative leucine-rich repeat transmembrane-type receptor kinase are essential for the normal development of the epidermal surface in Arabidopsis embryos.** *Plant J* 2008, **54:**30-42.

28. Clark SE, Williams RW, Meyerowitz EM: **The *CLAVATA1* gene encodes a putative receptor kinase that controls shoot and floral meristem size in Arabidopsis.** *Cell* 1997, **89:**575-585.

29. DeYoung BJ, Bickle KL, Schrage KJ, Muskett P, Patel K, Clark SE: **The CLAVATA1-related BAM1, BAM2 and BAM3 receptor kinase-like proteins are required for meristem function in Arabidopsis.** *Plant J* 2006, **45:**1-16.

30. Hord CL, Chen C, Deyoung BJ, Clark SE, Ma H: **The BAM1/BAM2 receptor-like kinases are important regulators of Arabidopsis early anther development.** *Plant Cell* 2006, **18:**1667-1680.

31. Jinn TL, Stone JM, Walker JC: **HAESA, an Arabidopsis leucine-rich repeat receptor kinase, controls floral organ abscission.** *Gene Dev* 2000, **14:**108-117.

32. Luo M, Dennis ES, Berger F, Peacock WJ, Chaudhury A: **MINISEED3 (MINI3), a WRKY family gene, and HAIKU2 (IKU2), a leucine-rich repeat (LRR) KINASE gene, are regulators of seed size in Arabidopsis.** *P Natl Acad Sci USA* 2005, **102:**17531-17536.

33. Fisher K, Turner S: **PXY, a receptor-like kinase essential for maintaining polarity during plant vascular-tissue development.** *Curr Biol* 2007, **17:**1061-1066.

34. Hirakawa Y, Shinohara H, Kondo Y, Inoue A, Nakanomyo I, Ogawa M, Sawa S, Ohashi-Ito K, Matsubayashi Y, Fukuda H: **Non-cell-autonomous control of vascular stem cell fate by a CLE peptide/receptor system.** *P Natl Acad Sci USA* 2008, **105:**15208-15213.

35. Gomez-Gomez L, Boller T: **FLS2: an LRR receptor-like kinase involved in the perception of the bacterial elicitor flagellin in Arabidopsis.** *Mol Cell* 2000, **5:**1003-1011.

36. Zipfel C, Kunze G, Chinchilla D, Caniard A, Jones JD, Boller T, Felix G: **Perception of the bacterial PAMP EF-Tu by the receptor EFR restricts Agrobacterium-mediated transformation.** *Cell* 2006, **125:**749-760.

37. Xu SL, Rahman A, Baskin TI, Kieber JJ: **Two leucine-rich repeat receptor kinases mediate signaling, linking cell wall biosynthesis and ACC synthase in Arabidopsis.** *Plant Cell* 2008, **20:**3065-3079.

38. Shpak ED, McAbee JM, Pillitteri LJ, Torii KU: **Stomatal patterning and differentiation by synergistic interactions of receptor kinases.** *Science* 2005, **309:**290-293.

39. Torii KU, Mitsukawa N, Oosumi T, Matsuura Y, Yokoyama R, Whittier RF, Komeda Y: **The Arabidopsis *ERECTA* gene encodes a putative receptor protein kinase with extracellular leucine-rich repeats.** *Plant Cell* 1996, **8:**735-746.
