# Supplementary figures and images for: Genome-wide cloning and sequence analysis of leucine-rich repeat receptor-like protein kinase genes in Arabidopsis thaliana
Source: BMC Genomics. 2010 Jan 11;11:19. doi: 10.1186/1471-2164-11-19 (PMC2817689; doi:10.1186/1471-2164-11-19)

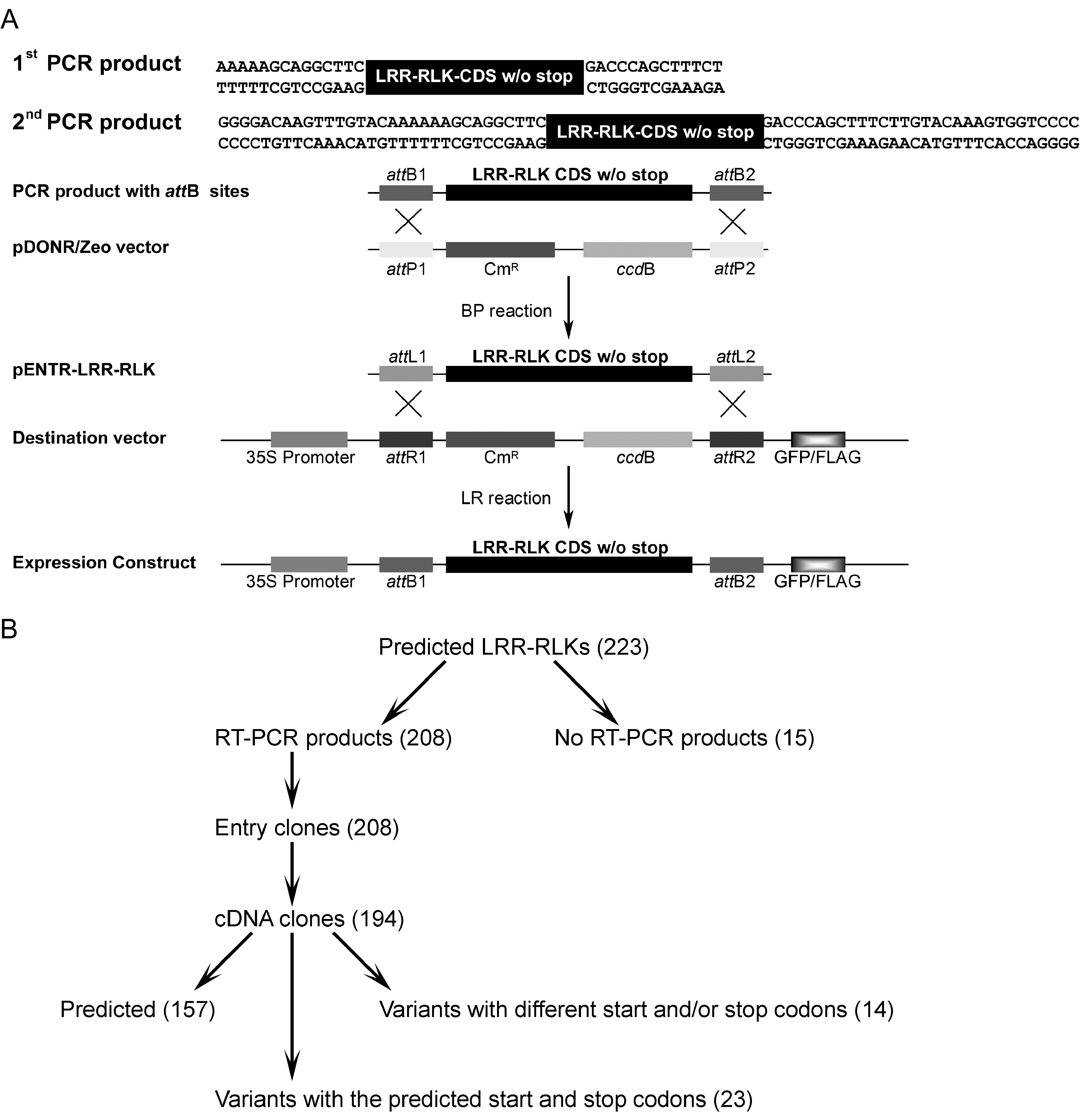

Supplement: Additional file 2 — Cloning strategy and results. (a) Target LRR-RLK sequences without stop codons are RT-PCR amplified, agarose gel purified and recombined with the pDONR/ZeoR vector by BP clonase to create pENTR-LRR-RLK entry clones. Final expression constructs are created by performing LR clonase-mediated DNA recombination between the pENTR-LRR-RLK clones and the destination vectors that contain GFP or FLAG epitope tags. (a) The cloning results of the predicted LRR-RLKs in Arabidopsis. [file 1471-2164-11-19-S2.TIFF]

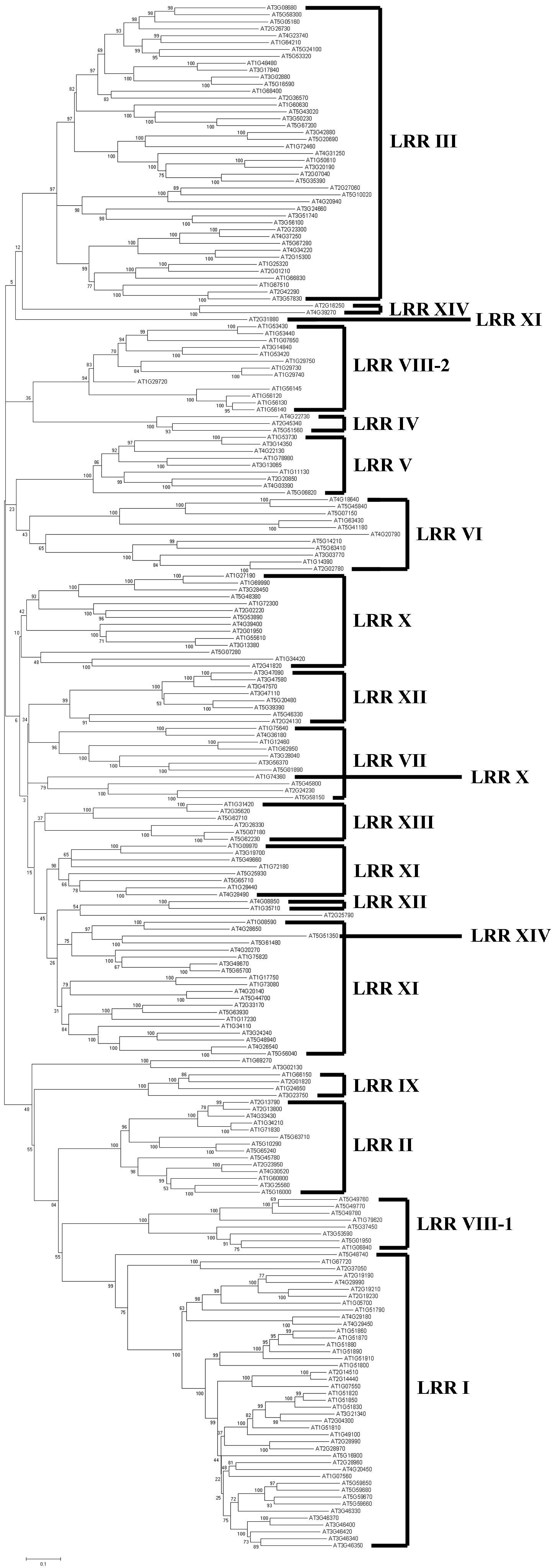

Supplement: Additional file 5 — LRR-RLKs phylogeny based on the full-length amino acid sequences. The previously assigned LRR subfamily names are shown on the right in black. [file 1471-2164-11-19-S5.TIFF]
