# Supplementary material for: Genome-wide cloning and sequence analysis of leucine-rich repeat receptor-like protein kinase genes in Arabidopsis thaliana
Source: BMC Genomics. 2010 Jan 11;11:19. doi: 10.1186/1471-2164-11-19 (PMC2817689; doi:10.1186/1471-2164-11-19)
Supplement: Additional file 3 — Sequence alignments of isolated LRR-RLKs displaying different coding sequences and containing one continuous ORF. Corresponding genomic DNA sequences, predicted mRNA sequences, previously reported cDNA sequences (if available), and isolated cDNA sequences obtained from this report for each LRR-RLK were aligned. Sequences with differences are indicated with red boxes. [file 1471-2164-11-19-S3.RTF]

At1g05700
                                                                                                                                                               
                     *        20         *        40         *        60         *        80         *       100         *       120         *       140       
Genomic   : ATGGAAGAGTTTCGTTTTCTCTATTTGATATATTCAGCAGCATTTGCATTGTGTCTCGTCGTTTCTGTTCTTGCTCAAGACCAATCAGGTTTTCTTAACTGATTCATTTATCTTTCAAACTATTTTCTAGTGAAGAAAGT :  140
NM_100450 : ATGGAAGAGTTTCGTTTTCTCTATTTGATATATTCAGCAGCATTTGCATTGTGTCTCGTCGTTTCTGTTCTTGCTCAAGACCAATCAGGTTT------------------------------------------------ :   92
FJ708625  : ATGGAAGAGTTTCGTTTTCTCTATTTGATATATTCAGCAGCATTTGCATTGTGTCTCGTCGTTTCTGTTCTTGCTCAAGACCAATCAGGTTT------------------------------------------------ :   92
                                                                                                                                                               
                     *       160         *       180         *       200         *       220         *       240         *       260         *       280       
Genomic   : CTAATCCTTTATTCTTTTCATAACTTTAACTCGTTGCGTGATCACAGGTTTCATAAGCATCGATTGTGGAATTCCAAGTGGATCATCGTACAAAGACGACACAACAGGTATAAACTACGTCTCAGATTCATCCTTTGTCG :  280
NM_100450 : ---------------------------------------------------CATAAGCATCGATTGTGGAATTCCAAGTGGATCATCGTACAAAGACGACACAACAGGTATAAACTACGTCTCAGATTCATCCTTTGTCG :  181
FJ708625  : ---------------------------------------------------CATAAGCATCGATTGTGGAATTCCAAGTGGATCATCGTACAAAGACGACACAACAGGTATAAACTACGTCTCAGATTCATCCTTTGTCG :  181
                                                                                                                                                               
                     *       300         *       320         *       340         *       360         *       380         *       400         *       420       
Genomic   : AAACCGGAGTCTCCAAATCGATCCCTTTCACAGCTCAAAGACAGCTTCAAAACCTGAGGAGCTTTCCCGAAGGTTCAAGAAACTGCTATACATTGATTCCAATACAAGGGAAGGGCAAGAAATATCTCATAAGAGCTAGT :  420
NM_100450 : AAACCGGAGTCTCCAAATCGATCCCTTTCACAGCTCAAAGACAGCTTCAAAACCTGAGGAGCTTTCCCGAAGGTTCAAGAAACTGCTATACATTGATTCCAATACAAGGGAAGGGCAAGAAATATCTCATAAGAGCTAGT :  321
FJ708625  : AAACCGGAGTCTCCAAATCGATCCCTTTCACAGCTCAAAGACAGCTTCAAAACCTGAGGAGCTTTCCCGAAGGTTCAAGAAACTGCTATACATTGATTCCAATACAAGGGAAGGGCAAGAAATATCTCATAAGAGCTAGT :  321
                                                                                                                                                               
                     *       440         *       460         *       480         *       500         *       520         *       540         *       560       
Genomic   : TTCATGTACGGTAACTACGATGGAGAAAATGGTTCACCGGAGTTTGATCTGTTTCTTGGTGGCAATATTTGGGATACCGTCTTGCTCAGCAATGGGTCGAGCATTGTTTCCAAAGAAGTCGTTTACTTGAGCCAATCTGA :  560
NM_100450 : TTCATGTACGGTAACTACGATGGAGAAAATGGTTCACCGGAGTTTGATCTGTTTCTTGGTGGCAATATTTGGGATACCGTCTTGCTCAGCAATGGGTCGAGCATTGTTTCCAAAGAAGTCGTTTACTTGAGCCAATCTGA :  461
FJ708625  : TTCATGTACGGTAACTACGATGGAGAAAATGGTTCACCGGAGTTTGATCTGTTTCTTGGTGGCAATATTTGGGATACCGTCTTGCTCAGCAATGGGTCGAGCATTGTTTCCAAAGAAGTCGTTTACTTGAGCCAATCTGA :  461
                                                                                                                                                               
                     *       580         *       600         *       620         *       640         *       660         *       680         *       700       
Genomic   : GAACATATTTGTGTGTTTGGGAAACAAAGGCAAAGGAACTCCATTTATCTCGACTTTAGAGCTTAGGTTTCTCGGGAACGACAATACGACATATGATTCTCCAAACGGTGCTCTCTTCTTCTCTAGACGCTGGGACTTAC :  700
NM_100450 : GAACATATTTGTGTGTTTGGGAAACAAAGGCAAAGGAACTCCATTTATCTCGACTTTAGAGCTTAGGTTTCTCGGGAACGACAATACGACATATGATTCTCCAAACGGTGCTCTCTTCTTCTCTAGACGCTGGGACTTAC :  601
FJ708625  : GAACATATTTGTGTGTTTGGGAAACAAAGGCAAAGGAACTCCATTTATCTCGACTTTAGAGCTTAGGTTTCTCGGGAACGACAATACGACATATGATTCTCCAAACGGTGCTCTCTTCTTCTCTAGACGCTGGGACTTAC :  601
                                                                                                                                                               
                     *       720         *       740         *       760         *       780         *       800         *       820         *       840       
Genomic   : GCTCTCTCATGGGTTCACCTGTTAGGTAAAGCTCTGTTTCTTGCTCTGTTTCGTGCTCTGTTTCATACTCTGTTTGATGCTCTGTTTCGTTAGCTCAAAGTTTACTTTTTACCAGTTGAAACTTGAAATCTGAAGCTTTT :  840
NM_100450 : GCTCTCTCATGGGTTCACCTGTTAG------------------------------------------------------------------------------------------------------------------- :  626
FJ708625  : GCTCTCTCATGGGTTCACCTGTTAG------------------------------------------------------------------------------------------------------------------- :  626
                                                                                                                                                               
                     *       860         *       880         *       900         *       920         *       940         *       960         *       980       
Genomic   : TTGTGGATCAGATATGATGATGATGTGTACGACAGAATCTGGATACCTCGCAACTTTGGCTATTGTAGAGAGATCAATACCTCACTCCCCGTGACCTCAGACAATAATAGTTATAGTCTTTCAAGTTTGGTGATGAGCAC :  980
NM_100450 : -----------ATATGATGATGATGTGTACGACAGAATCTGGATACCTCGCAACTTTGGCTATTGTAGAGAGATCAATACCTCACTCCCCGTGACCTCAGACAATAATAGTTATAGTCTTTCAAGTTTGGTGATGAGCAC :  755
FJ708625  : -----------ATATGATGATGATGTGTACGACAGAATCTGGATACCTCGCAACTTTGGCTATTGTAGAGAGATCAATACCTCACTCCCCGTGACCTCAGACAATAATAGTTATAGTCTTTCAAGTTTGGTGATGAGCAC :  755
                                                                                                                                                               
                     *      1000         *      1020         *      1040         *      1060         *      1080         *      1100         *      1120       
Genomic   : AGCGATGACTCCAATAAACACAACAAGACCCATCACAATGACTTTGGAAAACAGTGATCCAAATGTCAGGTACTTTGTCTACATGCACTTCGCAGAGGTTGAAGATCTTAGTCTCAAACCGAACCAGACAAGAGAGTTCG : 1120
NM_100450 : AGCGATGACTCCAATAAACACAACAAGACCCATCACAATGACTTTGGAAAACAGTGATCCAAATGTCAGGTACTTTGTCTACATGCACTTCGCAGAGGTTGAAGATCTTAGTCTCAAACCGAACCAGACAAGAGAGTTCG :  895
FJ708625  : AGCGATGACTCCAATAAACACAACAAGACCCATCACAATGACTTTGGAAAACAGTGATCCAAATGTCAGGTACTTTGTCTACATGCACTTCGCAGAGGTTGAAGATCTTAGTCTCAAACCGAACCAGACAAGAGAGTTCG :  895
                                                                                                                                                               
                     *      1140         *      1160         *      1180         *      1200         *      1220         *      1240         *      1260       
Genomic   : ACATCAGCATTAACGGAGTGACAGTTGCTGCTGGATTCAGCCCCAAGTATCTTCAGACAAACACGTTTTTTCTAAACCCTGAGAGCCAATCGAAAATTGCGTTTTCACTCGTTCGAACCCCAAAGTCCACTCTTCCGCCA : 1260
NM_100450 : ACATCAGCATTAACGGAGTGACAGTTGCTGCTGGATTCAGCCCCAAGTATCTTCAGACAAACACGTTTTTTCTAAACCCTGAGAGCCAATCGAAAATTGCGTTTTCACTCGTTCGAACCCCAAAGTCCACTCTTCCGCCA : 1035
FJ708625  : ACATCAGCATTAACGGAGTGACAGTTGCTGCTGGATTCAGCCCCAAGTATCTTCAGACAAACACGTTTTTTCTAAACCCTGAGAGCCAATCGAAAATTGCGTTTTCACTCGTTCGAACCCCAAAGTCCACTCTTCCGCCA : 1035
                                                                                                                                                               
                     *      1280         *      1300         *      1320         *      1340         *      1360         *      1380         *      1400       
Genomic   : ATTGTTAACGCTCTAGAGATCTACGTCGCAAATAGTTTCTCGCAGTCTCTCACTAACCAAGAGGATGGTCTGTGCTAGTATAACATGACTAGCAAAGTTCAATCTTTTTTCTCAGTTTGATCAATGCATTCAATTGAAAC : 1400
NM_100450 : ATTGTTAACGCTCTAGAGATCTACGTCGCAAATAGTTTCTCGCAGTCTCTCACTAACCAAGAGGATGGT----------------------------------------------------------------------- : 1104
FJ708625  : ATTGTTAACGCTCTAGAGATCTACGTCGCAAATAGTTTCTCGCAGTCTCTCACTAACCAAGAGGATGGT----------------------------------------------------------------------- : 1104
                                                                                                                                                               
                     *      1420         *      1440         *      1460         *      1480         *      1500         *      1520         *      1540       
Genomic   : TTACAGGTGACGCGGTTACGAGTCTAAAGACGAGTTATAAAGTGAAGAAGAACTGGCACGGAGATCCTTGTTTGCCTAATGACTACATTTGGGAGGGCCTTAATTGCAGTTATGATAGTCTTACTCCTCCAAGAATCACA : 1540
NM_100450 : --------GACGCGGTTACGAGTCTAAAGACGAGTTATAAAGTGAAGAAGAACTGGCACGGAGATCCTTGTTTGCCTAATGACTACATTTGGGAGGGCCTTAATTGCAGTTATGATAGTCTTACTCCTCCAAGAATCACA : 1236
FJ708625  : --------GACGCGGTTACGAGTCTAAAGACGAGTTATAAAGTGAAGAAGAACTGGCACGGAGATCCTTGTTTGCCTAATGACTACATTTGGGAGGGCCTTAATTGCAGTTATGATAGTCTTACTCCTCCAAGAATCACA : 1236
                                                                                                                                                               
                     *      1560         *      1580         *      1600         *      1620         *      1640         *      1660         *      1680       
Genomic   : TCACTGTAAGTGTAGTCTAATTTTAATCGCTTCTTGATCAGTCGGTGGATTTTCTATATTGAGTAGGAAAAAAATTTACACTATTGGAGGCAGGAACTTATCATCAAGCGGGTTAACGGGTCATATATCTTCCTCCTTCT : 1680
NM_100450 : TCACTG----------------------------------------------------------------------------------------AACTTATCATCAAGCGGGTTAACGGGTCATATATCTTCCTCCTTCT : 1288
FJ708625  : TCACTG----------------------------------------------------------------------------------------AACTTATCATCAAGCGGGTTAACGGGTCATATATCTTCCTCCTTCT : 1288
                                                                                                                                                               
                     *      1700         *      1720         *      1740         *      1760         *      1780         *      1800         *      1820       
Genomic   : CTAACCTCACAATGATTCAAGAGCTGTGAGTTTACCTAATTCAAGATTCTGATGATACCTATAATTTTGTATTTTGTTAAAGAGTTTTTTCTGACTTGAACAAGACAACTCTATTATCAGAGACTTATCAAACAATGGCT : 1820
NM_100450 : CTAACCTCACAATGATTCAAGAGCT-----------------------------------------------------------------------------------------------AGACTTATCAAACAATGGCT : 1333
FJ708625  : CTAACCTCACAATGATTCAAGAGCT-----------------------------------------------------------------------------------------------AGACTTATCAAACAATGGCT : 1333
                                                                                                                                                               
                     *      1840         *      1860         *      1880         *      1900         *      1920         *      1940         *      1960       
Genomic   : TAACCGGAGACATTCCGGAGTTCCTCTCAAAACTGAAGTTCTTGAGGGTTTTGTAAGTTCTGGACATATCTTAACAAATGAGATCGATAATTATGGACTCTTAACTCTTAACAACATCTGCAGAAATCTAGAAAATAACA : 1960
NM_100450 : TAACCGGAGACATTCCGGAGTTCCTCTCAAAACTGAAGTTCTTGAGGGTTTT-----------------------------------------------------------------------AAATCTAGAAAATAACA : 1402
FJ708625  : TAACCGGAGACATTCCGGAGTTCCTCTCAAAACTGAAGTTCTTGAGGGTTTT-----------------------------------------------------------------------AAATCTAGAAAATAACA : 1402
                                                                                                                                                               
                     *      1980         *      2000         *      2020         *      2040         *      2060         *      2080         *      2100       
Genomic   : CTCTTACCGGTTCAGTTCCATCCGAGTTATTAGAAAGATCAAACACCGGATCATTCTCGCTAAGGCTAGGAGAAAATCCAGGACTCTGTACTGAGATTTCTTGCAGAAAAAGCAACAGCAAAAAACTCGTTATCCCGCTT : 2100
NM_100450 : CTCTTACCGGTTCAGTTCCATCCGAGTTATTAGAAAGATCAAACACCGGATCATTCTCGCTAAGGCTAGGAGAAAATCCAGGACTCTGTACTGAGATTTCTTGCAGAAAAAGCAACAGCAAAAAACTCGTTATCCCGCTT : 1542
FJ708625  : CTCTTACCGGTTCAGTTCCATCCGAGTTATTAGAAAGATCAAACACCGGATCATTCTCGCTAAGGCTAGGAGAAAATCCAGGACTCTGTACTGAGATTTCTTGCAGAAAAAGCAACAGCAAAAAACTCGTTATCCCGCTT : 1542
                                                                                                                                                               
                     *      2120         *      2140         *      2160         *      2180         *      2200         *      2220         *      2240       
Genomic   : GTCGCATCATTTGCAGCATTGTTCATCCTCTTGTTATTGTCTGGTGTGTTTTGGAGAATCAGAAACCGGAGAAACAAGTCCGGTACAATTACAAATCTATCTCTATATCTTTCCTTTTACATTCATATCTATCTGTTAAT : 2240
NM_100450 : GTCGCATCATTTGCAGCATTGTTCATCCTCTTGTTATTGTCTGGTGTGTTTTGGAGAATCAGAAACCGGAGAAACAA--------------------------------------------------------------- : 1619
FJ708625  : GTCGCATCATTTGCAGCATTGTTCATCCTCTTGTTATTGTCTGGTGTGTTTTGGAGAATCAGAAACCGGAGAAACAAGTCCG---------------------------------------------------------- : 1624
                                                                                                                                                               
                     *      2260         *      2280         *      2300         *      2320         *      2340         *      2360         *      2380       
Genomic   : CACATTACCAATTGTTAAAACTTTCTTTTGCAAAGTAAACTCCGCACCTCAAACCAGTCCAATGGCGAAATCGGAGAACAAATTACTGTTTACCTTTGCAGACGTTATAAAGATGACAAATAACTTCGGTCAAGTCCTCG : 2380
NM_100450 : ---------------------------------------------------------TCCAATGGCGAAATCGGAGAACAAATTACTGTTTACCTTTGCAGACGTTATAAAGATGACAAATAACTTCGGTCAAGTCCTCG : 1702
FJ708625  : -----------------------------------TAAACTCCGCACCTCAAACCAGTCCAATGGCGAAATCGGAGAACAAATTACTGTTTACCTTTGCAGACGTTATAAAGATGACAAATAACTTCGGTCAAGTCCTCG : 1729
      
                                                                                                                                                               
                     *      2400         *      2420         *      2440         *      2460         *      2480         *      2500         *      2520       
Genomic   : GCAAAGGAGGGTTTGGAACAGTCTACCATGGCTTCTATGACAACCTTCAAGTAGCTGTTAAACTTTTATCAGAAACATCAGCTCAAGGATTCAAAGAGTTTCGCTCTGAGGTATTGTAGTAGAACTTCAGATATGACAAA : 2520
NM_100450 : GCAAAGGAGGGTTTGGAACAGTCTACCATGGCTTCTATGACAACCTTCAAGTAGCTGTTAAACTTTTATCAGAAACATCAGCTCAAGGATTCAAAGAGTTTCGCTCTGAGGT---------------------------- : 1814
FJ708625  : GCAAAGGAGGGTTTGGAACAGTCTACCATGGCTTCTATGACAACCTTCAAGTAGCTGTTAAACTTTTATCAGAAACATCAGCTCAAGGATTCAAAGAGTTTCGCTCTGAGGT---------------------------- : 1841
                                                                                                                                                               
                     *      2540         *      2560         *      2580         *      2600         *      2620         *      2640         *      2660       
Genomic   : TGATTCCAAAAAGATATACAAATCATAACCACCTAAATACATGTAGGTCGAAGTTCTTGTGAGGGTTCATCATGTTAACCTCACTGCGCTGATCGGTTATTTCCATGAAGGTGACCAAATGGGATTGATCTATGAGTTCA : 2660
NM_100450 : ------------------------------------------------CGAAGTTCTTGTGAGGGTTCATCATGTTAACCTCACTGCGCTGATCGGTTATTTCCATGAAGGTGACCAAATGGGATTGATCTATGAGTTCA : 1906
FJ708625  : ------------------------------------------------CGAAGTTCTTGTGAGGGTTCATCATGTTAACCTCACTGCGCTGATCGGTTATTTCCATGAAGGTGACCAAATGGGATTGATCTATGAGTTCA : 1933
                                                                                                                                                               
                     *      2680         *      2700         *      2720         *      2740         *      2760         *      2780         *      2800       
Genomic   : TGGCTAACGGCAACATGGCGGATCATCTCGCGGGAAAGTATCAGCACACATTGAGCTGGAGACAAAGGCTTCAGATTGCACTTGATGCAGCACAAGGTATGATGAAACAAAGTGTCGATCTTTTATATACTAGGTTATTA : 2800
NM_100450 : TGGCTAACGGCAACATGGCGGATCATCTCGCGGGAAAGTATCAGCACACATTGAGCTGGAGACAAAGGCTTCAGATTGCACTTGATGCAGCACAAGG------------------------------------------- : 2003
FJ708625  : TGGCTAACGGCAACATGGCGGATCATCTCGCGGGAAAGTATCAGCACACATTGAGCTGGAGACAAAGGCTTCAGATTGCACTTGATGCAGCACAAGG------------------------------------------- : 2030
                                                                                                                                                               
                     *      2820         *      2840         *      2860         *      2880         *      2900         *      2920         *      2940       
Genomic   : ATTAATCCAAAAGTTTAAATCTTTAACGAAAAATCTATGAAGGGCTTGAGTATCTTCACTGTGGATGCAAACCTCCAATAGTCCACAGAGATGTGAAGACTTCAAACATATTGTTGAACGAGAAAAACAGAGCAAAGCTT : 2940
NM_100450 : -------------------------------------------GCTTGAGTATCTTCACTGTGGATGCAAACCTCCAATAGTCCACAGAGATGTGAAGACTTCAAACATATTGTTGAACGAGAAAAACAGAGCAAAGCTT : 2100
FJ708625  : -------------------------------------------GCTTGAGTATCTTCACTGTGGATGCAAACCTCCAATAGTCCACAGAGATGTGAAGACTTCAAACATATTGTTGAACGAGAAAAACAGAGCAAAGCTT : 2127
                                                                                                                                                               
                     *      2960         *      2980         *      3000         *      3020         *      3040         *      3060         *      3080       
Genomic   : GCAGATTTCGGACTCTCACGGAGTTTTCATACAGAGAGTCGTTCTCACGTATCCACCCTAGTCGCTGGAACTCCCGGATATCTCGATCCGCTGTCAGTTTTCTCTCTTATGTCTGCCATATTTGCCAAACATGGAATCTT : 3080
NM_100450 : GCAGATTTCGGACTCTCACGGAGTTTTCATACAGAGAGTCGTTCTCACGTATCCACCCTAGTCGCTGGAACTCCCGGATATCTCGATCCGCT------------------------------------------------ : 2192
FJ708625  : GCAGATTTCGGACTCTCACGGAGTTTTCATACAGAGAGTCGTTCTCACGTATCCACCCTAGTCGCTGGAACTCCCGGATATCTCGATCCGCT------------------------------------------------ : 2219
                                                                                                                                                               
                     *      3100         *      3120         *      3140         *      3160         *      3180         *      3200         *      3220       
Genomic   : TTTTTAGTGATTGTATGCTCCGTTTTGCAGATGCTTTGAGACAAATGGGCTAAACGAGAAGAGTGACATTTACAGCTTTGGAGTTGTTCTGTTGGAGATGATTACAGGCAAAACCGTGATCAAAGAGTCACAAACGAAAC : 3220
NM_100450 : ------------------------------ATGCTTTGAGACAAATGGGCTAAACGAGAAGAGTGACATTTACAGCTTTGGAGTTGTTCTGTTGGAGATGATTACAGGCAAAACCGTGATCAAAGAGTCACAAACGAAAC : 2302
FJ708625  : ------------------------------ATGCTTTGAGACAAATGGGCTAAACGAGAAGAGTGACATTTACAGCTTTGGAGTTGTTCTGTTGGAGATGATTACAGGCAAAACCGTGATCAAAGAGTCACAAACGAAAC : 2329
                                                                                                                                                               
                     *      3240         *      3260         *      3280         *      3300         *      3320         *      3340         *      3360       
Genomic   : GTGTTCATGTGAGTGATTGGGTGATATCGATCTTGAGGTCTACGAATGATGTGAACAATGTTATAGATTCAAAGATGGCGAAAGATTTTGATGTAAACTCTGTATGGAAAGTTGTGGAGCTTGCATTATCCTCTGTTTCG : 3360
NM_100450 : GTGTTCATGTGAGTGATTGGGTGATATCGATCTTGAGGTCTACGAATGATGTGAACAATGTTATAGATTCAAAGATGGCGAAAGATTTTGATGTAAACTCTGTATGGAAAGTTGTGGAGCTTGCATTATCCTCTGTTTCG : 2442
FJ708625  : GTGTTCATGTGAGTGATTGGGTGATATCGATCTTGAGGTCTACGAATGATGTGAACAATGTTATAGATTCAAAGATGGCGAAAGATTTTGATGTAAACTCTGTATGGAAAGTTGTGGAGCTTGCATTATCCTCTGTTTCG : 2469
                                                                                                                                                               
                     *      3380         *      3400         *      3420         *      3440              
Genomic   : CAGAACGTCTCTGATAGGCCAAACATGCCACATATTGTTAGAGGATTGAATGAGTGTCTTCAAAGAGAAGAGAGTAACAAGAACTAT : 3447
NM_100450 : CAGAACGTCTCTGATAGGCCAAACATGCCACATATTGTTAGAGGATTGAATGAGTGTCTTCAAAGAGAAGAGAGTAACAAGAACTAT : 2529
FJ708625  : CAGAACGTCTCTGATAGGCCAAACATGCCACATATTGTTAGAGGATTGAATGAGTGTCTTCAAAGAGAAGAGAGTAACAAGAACTAT : 2556


At1g07560
                                                                                                                                                               
                     *        20         *        40         *        60         *        80         *       100         *       120         *       140       
Genomic   : ATGAAAAACCTCCGTGGACTTCTATTGGCCTTCCTAGTTTTGTCTTTAGGCATTTCCGATTTTCTTCGAGCACAAGATCAACAAGGTATAACTTTTTTTGTTTCAGGTTTTCTTCTTTTAACTTTGACTAAACATGTTTC :  140
NM_100630 : ATGAAAAACCTCCGTGGACTTCTATTGGCCTTCCTAGTTTTGTCTTTAGGCATTTCCGATTTTCTTCGAGCACAAGATCAACAA-------------------------------------------------------- :   84
FJ708628  : ATGAAAAACCTCCGTGGACTTCTATTGGCCTTCCTAGTTTTGTCTTTAGGCATTTCCGATTTTCTTCGAGCACAAGATCAACAA-------------------------------------------------------- :   84
                                                                                                                                                               
                     *       160         *       180         *       200         *       220         *       240         *       260         *       280       
Genomic   : CTATCACAATTTAGTCATTAATTCAGTTTCCAAACTTATTTTTTTACCAAATTATTTCTCTGTTTTTTTTCTTTTGCCTTTATCAGGATTCATCAGTTTGGATTGCGGGTTGCAAGCCGATGAGTCTCCTTATACCGAAC :  280
NM_100630 : -------------------------------------------------------------------------------------GGATTCATCAGTTTGGATTGCGGGTTGCAAGCCGATGAGTCTCCTTATACCGAAC :  139
FJ708628  : -------------------------------------------------------------------------------------GGATTCATCAGTTTGGATTGCGGGTTGCAAGCCGATGAGTCTCCTTATACCGAAC :  139
                                                                                                                                                               
                     *       300         *       320         *       340         *       360         *       380         *       400         *       420       
Genomic   : CATTAACAAAGTTAACATTCACATCAGACGCCGATTTCATCAAAAGTGGAAAAAGCGGCAAGATTCAAAATGTTCCGGGGATGGAGTATATAAAGCCATACACGGTTTTGAGATATTTTCCAGATGGAGTACGAAACTGT :  420
NM_100630 : CATTAACAAAGTTAACATTCACATCAGACGCCGATTTCATCAAAAGTGGAAAAAGCGGCAAGATTCAAAATGTTCCGGGGATGGAGTATATAAAGCCATACACGGTTTTGAGATATTTTCCAGATGGAGTACGAAACTGT :  279
FJ708628  : CATTAACAAAGTTAACATTCACATCAGACGCCGATTTCATCAAAAGTGGAAAAAGCGGCAAGATTCAAAATGTTCCGGGGATGGAGTATATAAAGCCATACACGGTTTTGAGATATTTTCCAGATGGAGTACGAAACTGT :  279
                                                                                                                                                               
                     *       440         *       460         *       480         *       500         *       520         *       540         *       560       
Genomic   : TATACTCTTATCGTCATACAAGGCACAAACTATCTGATCGTAGCCATGTTTACTTATGGAAACTACGATAATCTTAACACACACCCAAAGTTCGACCTTTATCTTGGTCCAAATATATGGACCACGGTCGATTTGCAAAG :  560
NM_100630 : TATACTCTTATCGTCATACAAGGCACAAACTATCTGATCGTAGCCATGTTTACTTATGGAAACTACGATAATCTTAACACACACCCAAAGTTCGACCTTTATCTTGGTCCAAATATATGGACCACGGTCGATTTGCAAAG :  419
FJ708628  : TATACTCTTATCGTCATACAAGGCACAAACTATCTGATCGTAGCCATGTTTACTTATGGAAACTACGATAATCTTAACACACACCCAAAGTTCGACCTTTATCTTGGTCCAAATATATGGACCACGGTCGATTTGCAAAG :  419
                                                                                                                                                               
                     *       580         *       600         *       620         *       640         *       660         *       680         *       700       
Genomic   : AAATGTAAACGGAACAAGGGCGGAAATCATTCACATACCAAGGTCAACTTCTTTGCAGATTTGTCTTGTTAAGACGGGAACAACTACGCCGCTAATATCAGCTTTAGAACTACGACCATTGAGAAATAATACTTATATTC :  700
NM_100630 : AAATGTAAACGGAACAAGGGCGGAAATCATTCACATACCAAGGTCAACTTCTTTGCAGATTTGTCTTGTTAAGACGGGAACAACTACGCCGCTAATATCAGCTTTAGAACTACGACCATTGAGAAATAATACTTATATTC :  559
FJ708628  : AAATGTAAACGGAACAAGGGCGGAAATCATTCACATACCAAGGTCAACTTCTTTGCAGATTTGTCTTGTTAAGACGGGAACAACTACGCCGCTAATATCAGCTTTAGAACTACGACCATTGAGAAATAATACTTATATTC :  559
                                                                                                                                                               
                     *       720         *       740         *       760         *       780         *       800         *       820         *       840       
Genomic   : CTCAATCTGGTTCCCTCAAGACATTGTTCCGCGTCCATTTGACTGATTCCAAAGAAACTGTTCGGTAAGCCAACTCAAAACCAATTGGTAATGAATAGATTGGTTTCCACTGGATACACTGTTCGGTAATACTTTCCATC :  840
NM_100630 : CTCAATCTGGTTCCCTCAAGACATTGTTCCGCGTCCATTTGACTGATTCCAAAGAAACTGTTCGGTA------------------------------------------------------------------------- :  626
FJ708628  : CTCAATCTGGTTCCCTCAAGACATTGTTCCGCGTCCATTTGACTGATTCCAAAGAAACTGTTCGGTA------------------------------------------------------------------------- :  626
                                                                                                                                                               
                     *       860         *       880         *       900         *       920         *       940         *       960         *       980       
Genomic   : CTTTCTCTTCAGGTATCCAGAAGATGTCCATGATCGTCTTTGGAGTCCATTTTTCATGCCGGAATGGAGACTGTTAAGAACAAGTCTCACGGTCAACACTTCTGATGATAACGGCTATGACATACCGGAAGACGTAGTGG :  980
NM_100630 : ---------------TCCAGAAGATGTCCATGATCGTCTTTGGAGTCCATTTTTCATGCCGGAATGGAGACTGTTAAGAACAAGTCTCACGGTCAACACTTCTGATGATAACGGCTATGACATACCGGAAGACGTAGTGG :  751
FJ708628  : ---------------TCCAGAAGATGTCCATGATCGTCTTTGGAGTCCATTTTTCATGCCGGAATGGAGACTGTTAAGAACAAGTCTCACGGTCAACACTTCTGATGATAACGGCTATGACATACCGGAAGACGTAGTGG :  751
                                                                                                                                                               
                     *      1000         *      1020         *      1040         *      1060         *      1080         *      1100         *      1120       
Genomic   : TTACGGCAGCCACACCTGCAAATGTGAGTTCACCATTAACCATTTCTTGGAATTTGGAAACACCTGACGACTTAGTTTATGCATACCTCCACGTCGCTGAGATCCAATCCTTAAGGGAAAATGATACCAGGGAATTCAAC : 1120
NM_100630 : TTACGGCAGCCACACCTGCAAATGTGAGTTCACCATTAACCATTTCTTGGAATTTGGAAACACCTGACGACTTAGTTTATGCATACCTCCACGTCGCTGAGATCCAATCCTTAAGGGAAAATGATACCAGGGAATTCAAC :  891
FJ708628  : TTACGGCAGCCACACCTGCAAATGTGAGTTCACCATTAACCATTTCTTGGAATTTGGAAACACCTGACGACTTAGTTTATGCATACCTCCACGTCGCTGAGATCCAATCCTTAAGGGAAAATGATACCAGGGAATTCAAC :  891
                                                                                                                                                               
                     *      1140         *      1160         *      1180         *      1200         *      1220         *      1240         *      1260       
Genomic   : ATTAGCGCAGGCCAAGATGTTAACTATGGACCTGTTAGTCCTGACGAGTTTCTAGTGGGTACTCTATTCAATACATCACCAGTTAAATGTGAAGGAGGGACATGCCATTTGCAACTAATAAAAACTCCAAAATCAACTCT : 1260
NM_100630 : ATTAGCGCAGGCCAAGATGTTAACTATGGACCTGTTAGTCCTGACGAGTTTCTAGTGGGTACTCTATTCAATACATCACCAGTTAAATGTGAAGGAGGGACATGCCATTTGCAACTAATAAAAACTCCAAAATCAACTCT : 1031
FJ708628  : ATTAGCGCAGGCCAAGATGTTAACTATGGACCTGTTAGTCCTGACGAGTTTCTAGTGGGTACTCTATTCAATACATCACCAGTTAAATGTGAAGGAGGGACATGCCATTTGCAACTAATAAAAACTCCAAAATCAACTCT : 1031
                                                                                                                                                               
                     *      1280         *      1300         *      1320         *      1340         *      1360         *      1380         *      1400       
Genomic   : CCCTCCCCTCCTCAATGCTATTGAGGCTTTTATAACAGTGGAGTTTCCGCAGTCTGAAACTAATGCAAATGACGGTATGTCAGCAAAATCTGAGTATATATATACTTTTTTGTATATACTTAAGTGTTTTCATTCTGTTA : 1400
NM_100630 : CCCTCCCCTCCTCAATGCTATTGAGGCTTTTATAACAGTGGAGTTTCCGCAGTCTGAAACTAATGCAAATGACG------------------------------------------------------------------ : 1105
FJ708628  : CCCTCCCCTCCTCAATGCTATTGAGGCTTTTATAACAGTGGAGTTTCCGCAGTCTGAAACTAATGCAAATGACG------------------------------------------------------------------ : 1105
                                                                                                                                                               
                     *      1420         *      1440         *      1460         *      1480         *      1500         *      1520         *      1540       
Genomic   : TTGTGTGTTAGTCCTTGCTATCAAGAGCATAGAAACAAGCTATGGATTAAGTAGAATTAGTTGGCAAGGAGATCCATGTGTTCCTCAACAATTATTGTGGGATGGTCTTACCTGCGAGTACACCAATATGTCTACACCAC : 1540
NM_100630 : -----------TCCTTGCTATCAAGAGCATAGAAACAAGCTATGGATTAAGTAGAATTAGTTGGCAAGGAGATCCATGTGTTCCTCAACAATTATTGTGGGATGGTCTTACCTGCGAGTACACCAATATGTCTACACCAC : 1234
FJ708628  : -----------TCCTTGCTATCAAGAGCATAGAAACAAGCTATGGATTAAGTAGAATTAGTTGGCAAGGAGATCCATGTGTTCCTCAACAATTATTGTGGGATGGTCTTACCTGCGAGTACACCAATATGTCTACACCAC : 1234
                                                                                                                                                               
                     *      1560         *      1580         *      1600         *      1620         *      1640         *      1660         *      1680       
Genomic   : CAAGAATCCATTCCTTGTACGTCCATGGCTATTCGTAACATTAAAACAGTTTTTTGTTAAGCTCTGCATTTCGTAACATCTTACACTTTAAATGTGTGTTATGATCTTTAATTACCACCAGAGACTTGTCCTCAAGTGAA : 1680
NM_100630 : CAAGAATCCATTCCTT---------------------------------------------------------------------------------------------------------AGACTTGTCCTCAAGTGAA : 1269
FJ708628  : CAAGAATCCATTCCTT---------------------------------------------------------------------------------------------------------AGACTTGTCCTCAAGTGAA : 1269
                                                                                                                                                               
                     *      1700         *      1720         *      1740         *      1760         *      1780         *      1800         *      1820       
Genomic   : CTAACTGGAATTATAGTTCCTGAGATCCAAAATCTTACGGAGCTTAAAAAATTGTAAGTATGAACACATTGCCAATTTTTTATCAAGGTCAAACACATACTCTGTTTTACCTGAATACTCTGTTTTACTTGAATACTCTG : 1820
NM_100630 : CTAACTGGAATTATAGTTCCTGAGATCCAAAATCTTACGGAGCTTAAAAAATTG-------------------------------------------------------------------------------------- : 1323
FJ708628  : CTAACTGGAATTATAGTTCCTGAGATCCAAAATCTTACGGAGCTTAAAAAATTG-------------------------------------------------------------------------------------- : 1323
                                                                                                                                                               
                     *      1840         *      1860         *      1880         *      1900         *      1920         *      1940         *      1960       
Genomic   : TTTATCAAGGCCAAACATATAATCAATTAATTCATTTTTGCATATGATAGGGACTTTTCAAATAATAATCTAACAGGTGGAGTACCTGAATTTCTAGCCAAAATGAAATCATTGTTAGTCATGTAAGTTACTTGGTCACT : 1960
NM_100630 : ---------------------------------------------------GACTTTTCAAATAATAATCTAACAGGTGGAGTACCTGAATTTCTAGCCAAAATGAAATCATTGTTAGTCAT------------------ : 1394
FJ708628  : ---------------------------------------------------GACTTTTCAAATAATAATCTAACAGGTGGAGTACCTGAATTTCTAGCCAAAATGAAATCATTGTTAGTCAT------------------ : 1394
                                                                                                                                                               
                     *      1980         *      2000         *      2020         *      2040         *      2060         *      2080         *      2100       
Genomic   : CATATGAATCACATTGCCAATTTATTATCCGAGTCTGTTGATGTTTATTTTTTACCTAGGCAGAAACTTAAGCGGAAACAACCTTAGTGGATCAGTCCCTCAAGCTCTTCTCAACAAAGTGAAAAATGGGTTGAAGTTGA : 2100
NM_100630 : ---------------------------------------------------------------AAACTTAAGCGGAAACAACCTTAGTGGATCAGTCCCTCAAGCTCTTCTCAACAAAGTGAAAAATGG----------- : 1460
FJ708628  : ---------------------------------------------------------------AAACTTAAGCGGAAACAACCTTAGTGGATCAGTCCCTCAAGCTCTTCTCAACAAAGTGAAAAATGGGTTGAAGTTGA : 1471
                                                                                                                                                               
                     *      2120         *      2140         *      2160         *      2180         *      2200         *      2220         *      2240       
Genomic   : AGTAAGATCTTCTCAACTATATTTAGTCTTCAATAGTTGATGGTCATTGAAATATGATATCTTAATTTGCAGTATGTCTTCACTAACACTTGATTAGCTTGTTTACAATACTAGCATTCAGGGAAATCCAAACCTATGTT : 2240
NM_100630 : -------------------------------------------------------------------------------------------------------------------------------------------- :    -
FJ708628  : A-----------------------------------------------------------------------------------------------------------------CATTCAGGGAAATCCAAACCTATGTT : 1498
                                                                                                                                                               
                     *      2260         *      2280         *      2300         *      2320         *      2340         *      2360         *      2380       
Genomic   : TTTCCAGTTCATGCAACAAAAAGAAAAATAGCATCATGTTACCAGTTGTTGCATCACTTGCTTCTTTGGCTGCTATAATAGCAATGATCGCTCTGCTTTTTGTTTGTATTAAAAGACGCTCATCAAGCAGAAAAGGTATC : 2380
NM_100630 : -------TTCATGCAACAAAAAGAAAAATAGCATCATGTTACCAGTTGTTGCATCACTTGCTTCTTTGGCTGCTATAATAGCAATGATCGCTCTGCTTTTTGTTTGTATTAAAAGACGCTCATCAAGCAGAAAAGGT--- : 1590
FJ708628  : TTTCCAGTTCATGCAACAAAAAGAAAAATAGCATCATGTTACCAGTTGTTGCATCACTTGCTTCTTTGGCTGCTATAATAGCAATGATCGCTCTGCTTTTTGTTTGTATTAAAAGACGCTCATCAAGCAGAAAAGGT--- : 1635
      
                                                                                                                                                         
                     *      2400         *      2420         *      2440         *      2460         *      2480         *      2500         *      2520       
Genomic   : TGTATCAAATTCATCAAACCAGAAATATTATTGTATGCATACATAATTATAAAGTGATTTCTAATTATTTGTGTATGGTTCTAGGTCCATCACCAAGCCAACAATCAATAGAAACAATAAAGAAAAGATATACATATGCC : 2520
NM_100630 : --------------------------------------------------------------------------------------CCATCACCAAGCCAACAATCAATAGAAACAATAAAGAAAAGATATACATATGCC : 1644
FJ708628  : --------------------------------------------------------------------------------------CCATCACCAAGCCAACAATCAATAGAAACAATAAAGAAAAGATATACATATGCC : 1689
                                                                                                                                                               
                     *      2540         *      2560         *      2580         *      2600         *      2620         *      2640         *      2660       
Genomic   : GAAGTTCTTGCCATGACAAAGAAATTTGAAAGGGTTTTGGGTAAAGGAGGATTTGGAATGGTCTATCATGGTTATATAAATGGTACTGAAGAAGTAGCGGTCAAGCTACTCTCTCCATCATCAGCTCAAGGCTATAAAGA : 2660
NM_100630 : GAAGTTCTTGCCATGACAAAGAAATTTGAAAGGGTTTTGGGTAAAGGAGGATTTGGAATGGTCTATCATGGTTATATAAATGGTACTGAAGAAGTAGCGGTCAAGCTACTCTCTCCATCATCAGCTCAAGGCTATAAAGA : 1784
FJ708628  : GAAGTTCTTGCCATGACAAAGAAATTTGAAAGGGTTTTGGGTAAAGGAGGATTTGGAATGGTCTATCATGGTTATATAAATGGTACTGAAGAAGTAGCGGTCAAGCTACTCTCTCCATCATCAGCTCAAGGCTATAAAGA : 1829
                                                                                                                                                               
                     *      2680         *      2700         *      2720         *      2740         *      2760         *      2780         *      2800       
Genomic   : GTTCAAAACAGAGGTTTCTTTGGCACCCTTATGAATTTTTATACATTTTTGTAAGGCTAAGTTGACGTATATTATATATTGTACATATTTGGATAGGTAGAACTTCTTCTAAGGGTTTACCACACAAATCTGGTAAGTCT : 2800
NM_100630 : GTTCAAAACAGAGGT-----------------------------------------------------------------------------------AGAACTTCTTCTAAGGGTTTACCACACAAATCTGGTAAGTCT : 1841
FJ708628  : GTTCAAAACAGAGGT-----------------------------------------------------------------------------------AGAACTTCTTCTAAGGGTTTACCACACAAATCTGGTAAGTCT : 1886
                                                                                                                                                                                                                                                                                                                              
                     *      2820         *      2840         *      2860         *      2880         *      2900         *      2920         *      2940       
Genomic   : TGTTGGATACTGTGATGAAAAAGATCATCTGGCCCTCATCTACCAGTACATGGTCAATGGCGATTTGAAAAAACATTTTTCAGGTAACACATGTATATTCTCATGCATATATCATGACAGAAATGCATCGTGTTGATATG : 2940
NM_100630 : TGTTGGATACTGTGATGAAAAAGATCATCTGGCCCTCATCTACCAGTACATGGTCAATGGCGATTTGAAAAAACATTTTTCAGGTA------------------------------------------------------ : 1927
FJ708628  : TGTTGGATACTGTGATGAAAAAGATCATCTGGCCCTCATCTACCAGTACATGGTCAATGGCGATTTGAAAAAACATTTTTCAGGTA------------------------------------------------------ : 1972
                                                                                                                                                               
                     *      2960         *      2980         *      3000         *      3020         *      3040         *      3060         *      3080       
Genomic   : TGACATTCATGTCTATGCTAATACATGCAGGTAGCTCCATCATTAGCTGGGTTGATAGACTAAACATAGCTGTTGATGCAGCGTCAGGTTCGTCATTTTCATTTCCTTTTAACACCACTTGATCAAACTTTCTATTGTTT : 3080
NM_100630 : ---------------------------------GCTCCATCATTAGCTGGGTTGATAGACTAAACATAGCTGTTGATGCAGCGTCAGGTT-------------------------------------------------- : 1984
FJ708628  : ---------------------------------GCTCCATCATTAGCTGGGTTGATAGACTAAACATAGCTGTTGATGCAGCGTCAGGTT-------------------------------------------------- : 2029
                                                                                                                                                               
                     *      3100         *      3120         *      3140         *      3160         *      3180         *      3200         *      3220       
Genomic   : TGTTAAACACCAAAGAACTTTGGAGACAGGTTTGGAGTACTTGCATATTGGTTGCAAACCGCTGATAGTTCATAGAGATGTAAAAAGTTCTAACATACTATTGGACGATCAATTACAAGCTAAGCTTGCAGATTTTGGGC : 3220
NM_100630 : --------------------------------TGGAGTACTTGCATATTGGTTGCAAACCGCTGATAGTTCATAGAGATGTAAAAAGTTCTAACATACTATTGGACGATCAATTACAAGCTAAGCTTGCAGATTTTGGGC : 2092
FJ708628  : --------------------------------TGGAGTACTTGCATATTGGTTGCAAACCGCTGATAGTTCATAGAGATGTAAAAAGTTCTAACATACTATTGGACGATCAATTACAAGCTAAGCTTGCAGATTTTGGGC : 2137
                                                                                                                                                               
                     *      3240         *      3260         *      3280         *      3300         *      3320         *      3340         *      3360       
Genomic   : TTTCTAGATCTTTTCCCATTGGGGATGAATCTCATGTGTCTACACTTGTTGCTGGAACATTTGGATACCTTGATCATGAGTAAGAATTTTAGCTCTTTCTGAAAACTATCTTCTCTACCGGTTAAAACAAAAGAATCATA : 3360
NM_100630 : TTTCTAGATCTTTTCCCATTGGGGATGAATCTCATGTGTCTACACTTGTTGCTGGAACATTTGGATACCTTGATCATGA------------------------------------------------------------- : 2171
FJ708628  : TTTCTAGATCTTTTCCCATTGGGGATGAATCTCATGTGTCTACACTTGTTGCTGGAACATTTGGATACCTTGATCATGA------------------------------------------------------------- : 2216
                                                                                                                                                               
                     *      3380         *      3400         *      3420         *      3440         *      3460         *      3480         *      3500       
Genomic   : ATCATTTCGTCTTGCAGATACTACCAAACTAATAGGTTGTCTGAGAAGAGTGATGTCTACAGTTTTGGTGTTGTACTGCTAGAAATTATTACAAACAAACCGGTGATCGATCATAACCGCGACATGCCTCACATAGCAGA : 3500
NM_100630 : -----------------ATACTACCAAACTAATAGGTTGTCTGAGAAGAGTGATGTCTACAGTTTTGGTGTTGTACTGCTAGAAATTATTACAAACAAACCGGTGATCGATCATAACCGCGACATGCCTCACATAGCAGA : 2294
FJ708628  : -----------------ATACTACCAAACTAATAGGTTGTCTGAGAAGAGTGATGTCTACAGTTTTGGTGTTGTACTGCTAGAAATTATTACAAACAAACCGGTGATCGATCATAACCGCGACATGCCTCACATAGCAGA : 2339
                                                                                                                                                               
                     *      3520         *      3540         *      3560         *      3580         *      3600         *      3620         *      3640       
Genomic   : ATGGGTGAAACTTATGCTTACTAGAGGAGACATTAGCAATATTATGGATCCTAAACTTCAAGGGGTCTATGACTCTGGTTCTGCATGGAAAGCTCTTGAATTAGCAATGACATGTGTGAATCCTTCTTCCTTGAAAAGAC : 3640
NM_100630 : ATGGGTGAAACTTATGCTTACTAGAGGAGACATTAGCAATATTATGGATCCTAAACTTCAAGGGGTCTATGACTCTGGTTCTGCATGGAAAGCTCTTGAATTAGCAATGACATGTGTGAATCCTTCTTCCTTGAAAAGAC : 2434
FJ708628  : ATGGGTGAAACTTATGCTTACTAGAGGAGACATTAGCAATATTATGGATCCTAAACTTCAAGGGGTCTATGACTCTGGTTCTGCATGGAAAGCTCTTGAATTAGCAATGACATGTGTGAATCCTTCTTCCTTGAAAAGAC : 2479
                                                                                                                                                               
                     *      3660         *      3680         *      3700         *      3720         *      3740         *      3760         *           
Genomic   : CCAACATGTCCCATGTTGTTCATGAGCTGAAGGAGTGTCTGGTATCTGAAAACAACAGGACAAGAGACATAGACACATCGAGATCCATGGACATTAACTTAAGCTTTGGCACAGATGTGAACCCCAAGGCACGT : 3774
NM_100630 : CCAACATGTCCCATGTTGTTCATGAGCTGAAGGAGTGTCTGGTATCTGAAAACAACAGGACAAGAGACATAGACACATCGAGATCCATGGACATTAACTTAAGCTTTGGCACAGATGTGAACCCCAAGGCACGT : 2568
FJ708628  : CCAACATGTCCCATGTTGTTCATGAGCTGAAGGAGTGTCTGGTATCTGAAAACAACAGGACAAGAGACATAGACACATCGAGATCCATGGACATTAACTTAAGCTTTGGCACAGATGTGAACCCCAAGGCACGT : 2613


At1g14390
                                                                                                                                                               
                     *        20         *        40         *        60         *        80         *       100         *       120         *       140       
Genomic   : ATGCATAGTTCCTCTAAAAGCCAGGCCTTTTCTCTCACTTTTCTTCTCTTTCTTTTTCTTCTGCCATCAGTCTCAGAATCACAGCTAATATCAAGTGAATCAAGAACTCTATTAGAAATCCAAAAGCATTTACAATATCC :  140
NM_101306 : ATGCATAGTTCCTCTAAAAGCCAGGCCTTTTCTCTCACTTTTCTTCTCTTTCTTTTTCTTCTGCCATCAGTCTCAGAATCACAGCTAATATCAAGTGAATCAAGAACTCTATTAGAAATCCAAAAGCATTTACAATATCC :  140
FJ708634  : ATGCATAGTTCCTCTAAAAGCCAGGCCTTTTCTCTCACTTTTCTTCTCTTTCTTTTTCTTCTGCCATCAGTCTCAGAATCACAGCTAATATCAAGTGAATCAAGAACTCTATTAGAAATCCAAAAGCATTTACAATATCC :  140
                                                                                                                                                               
                     *       160         *       180         *       200         *       220         *       240         *       260         *       280       
Genomic   : ACCAACTCTTCGATCATGGAGCAACTGGACCAATTTCTGCTACCTCCCTTCTTCTCCTTCTTTCAAAATCCTCTGTTTCAATGGTCATGTAACTGAATTAACCGTCACGGGGAACAGAACCGTTAAGCTCCCCGGAAGAT :  280
NM_101306 : ACCAACTCTTCGATCATGGAGCAACTGGACCAATTTCTGCTACCTCCCTTCTTCTCCTTCTTTCAAAATCCTCTGTTTCAATGGTCATGTAACTGAATTAACCGTCACGGGGAACAGAACCGTTAAGCTCCCCGGAAGAT :  280
FJ708634  : ACCAACTCTTCGATCATGGAGCAACTGGACCAATTTCTGCTACCTCCCTTCTTCTCCTTCTTTCAAAATCCTCTGTTTCAATGGTCATGTAACTGAATTAACCGTCACGGGGAACAGAACCGTTAAGCTCCCCGGAAGAT :  280
                                                                                                                                                               
                     *       300         *       320         *       340         *       360         *       380         *       400         *       420       
Genomic   : TCTCAAGCGACTCACTATTCACTGTTCTTACAAAACTCTCAAACTTAAAGACATTGTCTCTGGTCTCTCTTGGCATCTCTGGTCCTCTCCCTTCACAGATCATCAGGTTATCTTCATCTCTTCAATCTCTGAATCTCAGT :  420
NM_101306 : TCTCAAGCGACTCACTATTCACTGTTCTTACAAAACTCTCAAACTTAAAGACATTGTCTCTGGTCTCTCTTGGCATCTCTGGTCCTCTCCCTTCACAGATCATCAGGTTATCTTCATCTCTTCAATCTCTGAATCTCAGT :  420
FJ708634  : TCTCAAGCGACTCACTATTCACTGTTCTTACAAAACTCTCAAACTTAAAGACATTGTCTCTGGTCTCTCTTGGCATCTCTGGTCCTCTCCCTTCACAGATCATCAGGTTATCTTCATCTCTTCAATCTCTGAATCTCAGT :  420
                                                                                                                                                               
                     *       440         *       460         *       480         *       500         *       520         *       540         *       560       
Genomic   : TCTAATTTCATCTCTGGGAATATTCCTAAAGAGATCTCGTCGTTGAAGAATCTGAGAAGTCTTGTTCTAGCGAACAATTTGTTTAACGGAAGTGTTCCTGATCTCAGAGGATTGTCGAATCTTCAAGAGCTGAATTTAGG :  560
NM_101306 : TCTAATTTCATCTCTGGGAATATTCCTAAAGAGATCTCGTCGTTGAAGAATCTGAGAAGTCTTGTTCTAGCGAACAATTTGTTTAACGGAAGTGTTCCTGATCTCAGAGGATTGTCGAATCTTCAAGAGCTGAATTTAGG :  560
FJ708634  : TCTAATTTCATCTCTGGGAATATTCCTAAAGAGATCTCGTCGTTGAAGAATCTGAGAAGTCTTGTTCTAGCGAACAATTTGTTTAACGGAAGTGTTCCTGATCTCAGAGGATTGTCGAATCTTCAAGAGCTGAATTTAGG :  560
                                                                                                                                                               
                     *       580         *       600         *       620         *       640         *       660         *       680         *       700       
Genomic   : CGGTAATAAACTCGGTCCTGAAGTTGTTCCTTCACTTGCAAGTAACTTGATTACTATTTCATTGAAGAACAACTCTTTTGGATCAAAGATTCCAGAACAGATCAAGAAGCTGAATAAGCTTCAGAGCTTAGATCTTTCCT :  700
NM_101306 : CGGTAATAAACTCGGTCCTGAAGTTGTTCCTTCACTTGCAAGTAACTTGATTACTATTTCATTGAAGAACAACTCTTTTGGATCAAAGATTCCAGAACAGATCAAGAAGCTGAATAAGCTTCAGAGCTTAGATCTTTCCT :  700
FJ708634  : CGGTAATAAACTCGGTCCCGAAGTTGTTCCTTCACTTGCAAGTAACTTGATTACTATTTCATTGAAGAACAACTCTTTTGGATCAAAGATTCCAGAACAGATCAAGAAGCTGAATAAGCTTCAGAGCTTAGATCTTTCCT :  700
                                                                                                                                                               
                     *       720         *       740         *       760         *       780         *       800         *       820         *       840       
Genomic   : CCAACAAGTTCACTGGCTCAATCCCAAGATTCCTGCTTTCGCTTCCTTCGCTTCAGAATCTAAGTTTAGCACAAAACTTGCTGAGTGGATCACTTCCAAATTCATCGTTGTGCAACTCCAAGCTTAGAATTTTAGATGTT :  840
NM_101306 : CCAACAAGTTCACTGGCTCAATCCCAAGATTCCTGCTTTCGCTTCCTTCGCTTCAGAATCTAAGTTTAGCACAAAACTTGCTGAGTGGATCACTTCCAAATTCATCGTTGTGCAACTCCAAGCTTAGAATTTTAGATGTT :  840
FJ708634  : CCAACAAGTTCACTGGCTCAATCCCAAGATTCCTGCTTTCGCTTCCTTCGCTTCAGAATCTAAGTTTAGCACAAAACTTGCTGAGTGGATCACTTCCAAATTCATCGTTGTGCAACTCCAAGCTTAGAATTTTAGATGTT :  840                                                                                                                                                               
                                                                                                                                                               
                     *       860         *       880         *       900         *       920         *       940         *       960         *       980       
Genomic   : TCTCGGAATCTTCTGACTGGGAAGCTTCCATCTTGCTTCTCTTCCAAGAAACAGACAGTACTGCTCTTCACATTCAATTGCTTGTCTATAAATGGTTCTCCTTCTGCAAAGTATCAGCGTCCAGTTACCTTCTGTGAAAA :  980
NM_101306 : TCTCGGAATCTTCTGACTGGGAAGCTTCCATCTTGCTTCTCTTCCAAGAAACAGACAGTACTGCTCTTCACATTCAATTGCTTGTCTATAAATGGTTCTCCTTCTGCAAAGTATCAGCGTCCAGTTACCTTCTGTGAAAA :  980
FJ708634  : TCTCGGAATCTTCTGACTGGGAAGCTTCCATCTTGCTTCTCTTCCAAGAAACAGACAGTACTGCTCTTCACATTCAATTGCTTGTCTATAAATGGTTCTCCTTCTGCAAAGTATCAGCGTCCAGTTACCTTCTGTGAAAA :  980
                                                                                                                                                               
                     *      1000         *      1020         *      1040         *      1060         *      1080         *      1100         *      1120       
Genomic   : CGAAGCAAAGCAAGCAGTAGCTGCTGTGAAGTCTGACACTAAAGATAAAGAAAGGAAAGAAGAAGATACAGGAATCGAACTTGGATTGGTGATTGGCATTATCATCGGTGTGATCCTCGTTTCAGCGGTTTTAGCTGGCT : 1120
NM_101306 : CGAAGCAAAGCAAGCAGTAGCTGCTGTGAAGTCTGACACTAAAGATAAAGAAAGGAAAGAAGAAGATACAGGAATCGAACTTGGATTGGTGATTGGCATTATCATCGGTGTGATCCTCGTTTCAGCGGTTTTAGCTGGCT : 1120
FJ708634  : CGAAGCAAAGCAAGCAGTAGCTGCTGTGAAGTCTGACACTAAAGATAAAGAAAGGAAAGAAGAAGATACAGGAATCGAACTTGGATTGGTGATTGGCATTATCATCGGTGTGATCCTCGTTTCAGCGGTTTTAGCTGGCT : 1120                                                                                                                                                               
                                                                                                                                                               
                     *      1140         *      1160         *      1180         *      1200         *      1220         *      1240         *      1260       
Genomic   : TGGTTTTGGTTAGGATGAGAAAATCAAGATCAAAAGAAGAGCCTTTGGAAGCAAACAACGTCGATCAGGTCACTGTTTGCAGCAACACCACCAGGTCCACCACATCAAAGACAGTACCAGATCTAAGTAGGCTATTACTT : 1260
NM_101306 : TGGTTTTGGTTAGGATGAGAAAATCAAGATCAAAAGAAGAGCCTTTGGAAGCAAACAACGTCGATCAGGTCACTGTTTGCAGCAACACCACCAGGTCCACCACATCAAAGACAGTACCAGATCTAAG------------- : 1247
FJ708634  : TGGTTTTGGTTAGGATGAGAAAATCAAGATCAAAAGAAGAGCCTTTGGAAGCAAACAACGTCGATCAGGTCACTGTTTGCAGCAACACCACCAGGTCCACCACATCAAAGACAGTACCAGATCTAAG------------- : 1247                                                                                                                                                               
                                                                                                                                                               
                     *      1280         *      1300         *      1320         *      1340         *      1360         *      1380         *      1400       
Genomic   : TGAAATCTTGAATCAAGTTTTCACACCCATTAACCTAACTAGCTTGATTTTTTCAAATGCAGGACGTGTACCGCAAACCATGAGATCCGCGGTGATTGGACTATCACCATACCGAGTTTTCTCTTTGGAGGAACTGGAAG : 1400
NM_101306 : ---------------------------------------------------------------ACGTGTACCGCAAACCATGAGATCCGCGGTGATTGGACTATCACCATACCGAGTTTTCTCTTTGGAGGAACTGGAAG : 1324
FJ708634  : ---------------------------------------------------------------ACGTGTACCGCAAACCATGAGATCCGCGGTGATTGGACTATCACCATACCGAGTTTTCTCTTTGGAGGAACTGGAAG : 1324
                                                                                                                                                               
                     *      1420         *      1440         *      1460         *      1480         *      1500         *      1520         *      1540       
Genomic   : AAGCAACCAACAATTTTGATGCAGAGAATCTCTGTGGAGAACAGGTAAAGGAAAATGTAAATAAGACATGATTTAAAATTGATCCGAGTGTCTAACTAATCGATATATCTAATTTCAAAACAGCTGTACAAAGGTTGTCT : 1540
NM_101306 : AAGCAACCAACAATTTTGATGCAGAGAATCTCTGTGGAGAACAG-------------------------------------------------------------------------------CTGTACAAAGGTTGTCT : 1385
FJ708634  : AAGCAACCAACAATTTTGATGCAGAGAATCTCTGTGGAGAACAG-------------------------------------------------------------------------------CTGTACAAAGGTTGTCT : 1385
                                                                                                                                                               
                     *      1560         *      1580         *      1600         *      1620         *      1640         *      1660         *      1680       
Genomic   : TAGAGAAGGCATAGCAGTGACAGTGAGGTGTATTAAGCTGAAACAGAAGAATTCAACACAGAATTTAGCTCAACAAATGGAAGTTTTATCAAAGCTAAGGCATATGCATTTGGTCAGTGTTCTTGGACACTGCATTGGTA : 1680
NM_101306 : TAGAGAAGGCATAGCAGTGACAGTGAGGTGTATTAAGCTGAAACAGAAGAATTCAACACAGAATTTAGCTCAACAAATGGAAGTTTTATCAAAGCTAAGGCATATGCATTTGGTCAGTGTTCTTGGACACTGCATTGGTA : 1525
FJ708634  : TAGAGAAGGCATAGCAGTGACAGTGAGGTGTATTAAGCTGAAACAGAAGAATTCAACACAGAATTTAGCTCAACAAATGGAAGTTTTATCAAAGCTAAGGCATATGCATTTGGTCAGTGTTCTTGGACACTGCATTGGTA : 1525
                                                                                                                                                               
                     *      1700         *      1720         *      1740         *      1760         *      1780         *      1800         *      1820       
Genomic   : CTTATCAAGATCATCATCCTTATGCCGGAAGCACTATTTTCATTGTCCAAGAATATATCTCTAACGGGTCTTTGAGGGATTACCTCACCGGTAATACTTGAAAAAGATTATCGATTTCAAGAACTTATATTTTTGTTGGG : 1820
NM_101306 : CTTATCAAGATCATCATCCTTATGCCGGAAGCACTATTTTCATTGTCCAAGAATATATCTCTAACGGGTCTTTGAGGGATTACCTCACCG-------------------------------------------------- : 1615
FJ708634  : CTTATCAAGATCATCATCCTTATGCCGGAAGCACTATTTTCATTGTCCAAGAATATATCTCTAACGGGTCTTTGAGGGATTACCTCACCG-------------------------------------------------- : 1615
                                                                                                                                                               
                     *      1840         *      1860         *      1880         *      1900         *      1920         *      1940         *      1960       
Genomic   : AAACTAATTATTTGGAAATGTTTTTGGCAGATTGGAGAAAGAAAGAGGTGTTGAAATGGCCTCAGAGAATGTCGATAGCGATTGGAGTTGCTCGAGGAATACAGTTCTTGCACACAGGAGTGGCACCAGGAATATTTGGG : 1960
NM_101306 : ------------------------------ATTGGAGAAAGAAAGAGGTGTTGAAATGGCCTCAGAGAATGTCGATAGCGATTGGAGTTGCTCGAGGAATACAGTTCTTGCACACAGGAGTGGCACCAGGAATATTTGGG : 1725
FJ708634  : ------------------------------ATTGGAGAAAGAAAGAGGTGTTGAAATGGCCTCAGAGAATGTCGATAGCGATTGGAGTTGCTCGAGGAATACAGTTCTTGCACACAGGAGTGGCACCAGGAATATTTGGG : 1725
                                                                                                                                                               
                     *      1980         *      2000         *      2020         *      2040         *      2060         *      2080         *      2100       
Genomic   : AACAATTTGGAAATAGAGAATGTTTTACTTGATGAAACACTCACTGTAAAACTCAGTGGTTATAGTATTCCTTTACCATCCAAGGTATATAAACAAACCAGATATAATATAATCAGGATATAGCTTCTAATCTTCAGTTT : 2100
NM_101306 : AACAATTTGGAAATAGAGAATGTTTTACTTGATGAAACACTCACTGTAAAACTCAGTGGTTATAGTATTCCTTTACCATCCAAG---------------------------------------CTTCTAATCTTCAGTTT : 1826
FJ708634  : AACAATTTGGAAATAGAGAATGTTTTACTTGATGAAACACTCACTGTAAAACTCAGTGGTTATAGTATTCCTTTACCATCCAAG-------------------------------------------------------- : 1809
                                                                                                                                                               
                     *      2120         *      2140         *      2160         *      2180         *      2200         *      2220         *      2240       
Genomic   : AACCTCTCATGAAATATATAACCTTCTGGGGGAATTTCAGGTTGGAGCGGAGAGCCCTAGTAATGAAGATGGAGAGAAAGAAGATGTGTACCAGTTTGGAGTGATACTAATTCAGATCATCACAGGCAAAGTAATAGCAG : 2240
NM_101306 : AACCTCTCATGAAATATATAACCTTCTGGGGGAATTTCAGGTTGGAGCGGAGAGCCCTAGTAATGAAGATGGAGAGAAAGAAGATGTGTACCAGTTTGGAGTGATACTAATTCAGATCATCACAGGCAAAGTAATAGCAG : 1966
FJ708634  : ----------------------------------------GTTGGAGCGGAGAGCCCTAGTAATGAAGATGGAGAGAAAGAAGATGTGTACCAGTTTGGAGTGATACTAATTCAGATCATCACAGGCAAAGTAATAGCAG : 1909
                                                                                                                                                               
                     *      2260         *      2280         *      2300         *      2320         *      2340         *      2360         *      2380       
Genomic   : CTGCATCTTCAGAGTTGGGGAGCTTGAAGCTTCAGCTGGAGAACAGTTTGAGAGACGAACCATCGGTGTTGCGTAGCTTGGCAGATCCGTGTGTGAGAGGAACATATGCATACGAGTCATTGAGAACCACAGTAGAGTTT : 2380
NM_101306 : CTGCATCTTCAGAGTTGGGGAGCTTGAAGCTTCAGCTGGAGAACAGTTTGAGAGACGAACCATCGGTGTTGCGTAGCTTGGCAGATCCGTGTGTGAGAGGAACATATGCATACGAGTCATTGAGAACCACAGTAGAGTTT : 2106
FJ708634  : CTGCATCTTCAGAGTTGGGGAGCTTGAAGCTTCAGCTGGAGAACAGTTTGAGAGACGAACCATCGGTGTTGCGTAGCTTGGCAGATCCGTGTGTGAGAGGAACATATGCATACGAGTCATTGAGAACCACAGTAGAGTTT : 2049
                                                                                                                                                               
                                                                                                                                          
                     *      2400         *      2420         *      2440         *      2460         *      2480         *      2500         *            
Genomic   : GCCATTAACTGTCTTTGTGAAGATCAGAGGAAGCGGCCATCGATAGAGGATGTTGTATGGAATCTGCAGTACACAATTCAAGTGCAACAAGGATGGACAAGCAGTGAGAACCTCGGGCTTGGTGGTTCAGAACTA : 2515
NM_101306 : GCCATTAACTGTCTTTGTGAAGATCAGAGGAAGCGGCCATCGATAGAGGATGTTGTATGGAATCTGCAGTACACAATTCAAGTGCAACAAGGATGGACAAGCAGTGAGAACCTCGGGCTTGGTGGTTCAGAACTA : 2241
FJ708634  : GCCATTAACTGTCTTTGTGAAGATCAGAGGAAGCGGCCATCGATAGAGGATGTTGTATGGAATCTGCAGTACACAATTCAAGTGCAACAAGGATGGACAAGCAGTGAGAACCTCGGGCTTGGTGGTTCAGAACTA : 2184


At1g31420
                                                                                                                                                               
                     *        20         *        40         *        60         *        80         *       100         *       120         *       140       
Genomic   : ATGATGGGCATCTGTGAGATGAAAAGCTGCTGCTCATGGCTTCTTTTGATCTCGTTGCTTTGCTCACTATCTAATGAAAGTCAAGCGATTAGTCCTGACGGTACGTCGATATGAATCAACTATTACTATTCATAGCGTCC :  140
NM_102881 : ATGATGGGCATCTGTGAGATGAAAAGCTGCTGCTCATGGCTTCTTTTGATCTCGTTGCTTTGCTCACTATCTAATGAAAGTCAAGCGATTAGTCCTGACGG--------------------------------------- :  101
AK226234  : ATGATGGGCATCTGTGAGATGAAAAGCTGCTGCTCATGGCTTCTTTTGATCTCGTTGCTTTGCTCACTATCTAATGAAAGTCAAGCGATTAGTCCTGACGG--------------------------------------- :  101
FJ708643  : ATGATGGGCATCTGTGAGATGAAAAGCTGCTGCTCATGGCTTCTTTTGATCTCGTTGCTTTGCTCACTATCTAATGAAAGTCAAGCGATTAGTCCTGACGG--------------------------------------- :  101
                                                                                                                                                               
                     *       160         *       180         *       200         *       220         *       240         *       260         *       280       
Genomic   : TGGACCTTTTATTGAGCTTTAATCCTTTATTGCTTGTTTCCATTCTTCAGGCGAGGCGCTTCTGAGCTTCAGAAATGCAGTTACTAGATCAGATAGTTTCATCCATCAGTGGAGACCGGAAGATCCAGATCCATGTAACT :  280
NM_102881 : ---------------------------------------------------CGAGGCGCTTCTGAGCTTCAGAAATGCAGTTACTAGATCAGATAGTTTCATCCATCAGTGGAGACCGGAAGATCCAGATCCATGTAACT :  190
AK226234  : ---------------------------------------------------CGAGGCGCTTCTGAGCTTCAGAAATGCAGTTACTAGATCAGATAGTTTCATCCATCAGTGGAGACCGGAAGATCCAGATCCATGTAACT :  190
FJ708643  : ---------------------------------------------------CGAGGCGCTTCTGAGCTTCAGAAATGCAGTTACTAGATCAGATAGTTTCATCCATCAGTGGAGACCGGAAGATCCAGATCCATGTAACT :  190
                                                                                                                                                               
                     *       300         *       320         *       340         *       360         *       380         *       400         *       420       
Genomic   : GGAACGGAGTGACATGTGATGCAAAAACGAAAAGAGTTATAACCTTGTAAGTTTTCAAACTTGTCAAGGTTTACTGGTTTTCTCTTCAGTATTTGTGCTCCCTAATACTCTTGTTTCGCAGGAATCTTACTTATCACAAA :  420
NM_102881 : GGAACGGAGTGACATGTGATGCAAAAACGAAAAGAGTTATAACCTTG---------------------------------------------------------------------------AATCTTACTTATCACAAA :  255
AK226234  : GGAACGGAGTGACATGTGATGCAAAAACGAAAAGAGTTATAACCTTG---------------------------------------------------------------------------AATCTTACTTATCACAAA :  255
FJ708643  : GGAACGGAGTGACATGTGATGCAAAAACGAAAAGAGTTATAACCTTG---------------------------------------------------------------------------AATCTTACTTATCACAAA :  255
                                                                                                                                                               
                     *       440         *       460         *       480         *       500         *       520         *       540         *       560       
Genomic   : ATAATGGGACCTTTACCTCCTGATATTGGAAAGTTGGACCATTTAAGGCTTTTGTATGTGCTCTCTAGCCTTATTCATTTTTATTGCTTAAGAGAGTACTTCGATTCTGATCATCTGTGTGTCATTGCAGAATGCTTCAC :  560
NM_102881 : ATAATGGGACCTTTACCTCCTGATATTGGAAAGTTGGACCATTTAAGGCTTTT-----------------------------------------------------------------------------AATGCTTCAC :  318
AK226234  : ATAATGGGACCTTTACCTCCTGATATTGGAAAGTTGGACCATTTAAGGCTTTT-----------------------------------------------------------------------------AATGCTTCAC :  318
FJ708643  : ATAATGGGACCTTTACCTCCTGATATTGGAAAGTTGGACCATTTAAGGCTTTT-----------------------------------------------------------------------------AATGCTTCAC :  318
                                                                                                                                                               
                     *       580         *       600         *       620         *       640         *       660         *       680         *       700       
Genomic   : AACAATGCTTTATATGGAGCAATACCTACAGCATTGGGAAATTGCACAGCATTGGAGGAAATGTAAGCGCTTTTCCAGGATTTTATACTGATTCCACATGGTTTCTGAGAGACCAATTCCAGTTGCTTATGGTCATCAAA :  700
NM_102881 : AACAATGCTTTATATGGAGCAATACCTACAGCATTGGGAAATTGCACAGCATTGGAGGAAAT------------------------------------------------------------------------------ :  380
AK226234  : AACAATGCTTTATATGGAGCAATACCTACAGCATTGGGAAATTGCACAGCATTGGAGGAAAT------------------------------------------------------------------------------ :  380
FJ708643  : AACAATGCTTTATATGGAGCAATACCTACAGCATTGGGAAATTGCACAGCATTGGAGGAAAT------------------------------------------------------------------------------ :  380
                                                                                                                                                               
                     *       720         *       740         *       760         *       780         *       800         *       820         *       840       
Genomic   : ATTGTCCAACTGGTTTTGCAGTCACTTGCAGAGTAACTACTTCACTGGACCAATCCCAGCTGAAATGGGAGACCTGCCTGGCCTTCAAAAGCTGTATGTTTTTTTCTCTTCCCTATACGTACTTGCAAGTGCATCCTTCT :  840
NM_102881 : ---------------------TCACTTGCAGAGTAACTACTTCACTGGACCAATCCCAGCTGAAATGGGAGACCTGCCTGGCCTTCAAAAGCTG---------------------------------------------- :  453
AK226234  : ---------------------TCACTTGCAGAGTAACTACTTCACTGGACCAATCCCAGCTGAAATGGGAGACCTGCCTGGCCTTCAAAAGCTG---------------------------------------------- :  453
FJ708643  : ---------------------TCACTTGCAGAGTAACTACTTCACTGGACCAATCCCAGCTGAAATGGGAGACCTGCCTGGCCTTCAAAAGCTG---------------------------------------------- :  453
                                                                                                                                                               
                     *       860         *       880         *       900         *       920         *       940         *       960         *       980       
Genomic   : TTTAGTGTATGCAATATCTATTTGTTTTTTGTTTTCATTTACTTCTATCACCATCTAGGATCATGGTAACATATGGTTGACTGCATAATGTCTTCTCATGCACATTATATTCAGAACTAGTTATACCTAAACTCTTGGTG :  980
NM_102881 : -------------------------------------------------------------------------------------------------------------------------------------------- :    -
AK226234  : -------------------------------------------------------------------------------------------------------------------------------------------- :    -
FJ708643  : -------------------------------------------------------------------------------------------------------------------------------------------- :    -
                                                                                                                                                               
                     *      1000         *      1020         *      1040         *      1060         *      1080         *      1100         *      1120       
Genomic   : AGCAATTTGAAAAGCATGATAATGTATTGTTTACTTCAGATTCACATATTGATTGTTCTCTTTTCTCTACTAAAACTGACTTTTGACTTTTTTATTTTATTTTCAAGGGACATGTCAAGCAACACTCTCAGTGGACCTAT : 1120
NM_102881 : ------------------------------------------------------------------------------------------------------------GACATGTCAAGCAACACTCTCAGTGGACCTAT :  485
AK226234  : ------------------------------------------------------------------------------------------------------------GACATGTCAAGCAACACTCTCAGTGGACCTAT :  485
FJ708643  : ------------------------------------------------------------------------------------------------------------GACATGTCAAGCAACACTCTCAGTGGACCTAT :  485
                                                                                                                                                               
                     *      1140         *      1160         *      1180         *      1200         *      1220         *      1240         *      1260       
Genomic   : CCCTGCTTCGCTTGGGCAGTTAAAAAAGCTTAGTAACTTGTGAGCACCTTAATCTCTAACTTCCGCTGTAATTTATCATATTGCAGTCCTGTGAATCTTTCTGTTTCGTTTGTCTTATGAAGCAATGTCTCGAACAACTT : 1260
NM_102881 : CCCTGCTTCGCTTGGGCAGTTAAAAAAGCTTAGTAACTT-----------------------------------------------------------------------------------CAATGTCTCGAACAACTT :  542
AK226234  : CCCTGCTTCGCTTGGGCAGTTAAAAAAGCTTAGTAACTT-----------------------------------------------------------------------------------CAATGTCTCGAACAACTT :  542
FJ708643  : CCCTGCTTCGCTTGGGCAGTTAAAAAAGCTTAGTAACTT-----------------------------------------------------------------------------------CAATGTCTCGAACAACTT :  542
                                                                                                                                                               
                     *      1280         *      1300         *      1320         *      1340         *      1360         *      1380         *      1400       
Genomic   : CCTGGTGGGGCAAATACCTTCAGACGGAGTGCTCTCTGGATTTTCTAAGAACTCGTAAGACAATAAACACCCTTTTCTTCTTGTAGGGATAGAAAAATAAAGCCTCTAAAGTTACATTTTTTTTTTTTTTTTGTCAATCC : 1400
NM_102881 : CCTGGTGGGGCAAATACCTTCAGACGGAGTGCTCTCTGGATTTTCTAAGAACTC-------------------------------------------------------------------------------------- :  596
AK226234  : CCTGGTGGGGCAAATACCTTCAGACGGAGTGCTCTCTGGATTTTCTAAGAACTC-------------------------------------------------------------------------------------- :  596
FJ708643  : CCTGGTGGGGCAAATACCTTCAGACGGAGTGCTCTCTGGATTTTCTAAGAACTC-------------------------------------------------------------------------------------- :  596
                                                                                                                                                               
                     *      1420         *      1440         *      1460         *      1480         *      1500         *      1520         *      1540       
Genomic   : TCTAAAGTTACATTTATACAGCTGATCTTTCATATTTCGCTTATATTCGAGATGATTTTTTGAACCACATAACTATTTAGCTAGATTGTATCAGTGCGACTGAAATTATATGATTCTCAAACTGATGCGTTACTACTACA : 1540
NM_102881 : -------------------------------------------------------------------------------------------------------------------------------------------- :    -
AK226234  : -------------------------------------------------------------------------------------------------------------------------------------------- :    -
FJ708643  : -------------------------------------------------------------------------------------------------------------------------------------------- :    -
                                                                                                                                                               
                     *      1560         *      1580         *      1600         *      1620         *      1640         *      1660         *      1680       
Genomic   : GCTTCATTGGAAATCTTAACTTGTGTGGAAAGCATGTTGATGTCGTGTGCCAAGATGATAGTGGAAACCCTTCATCACATTCTCAATCAGGTAAAAGCAATTATGAATTTCTTGTGATATACAGGTTTTGTTGTGGCTCT : 1680
NM_102881 : -CTTCATTGGAAATCTTAACTTGTGTGGAAAGCATGTTGATGTCGTGTGCCAAGATGATAGTGGAAACCCTTCATCACATTCTCAATCAGGT------------------------------------------------ :  687
AK226234  : -CTTCATTGGAAATCTTAACTTGTGTGGAAAGCATGTTGATGTCGTGTGCCAAGATGATAGTGGAAACCCTTCATCACATTCTCAATCAGGT------------------------------------------------ :  687
FJ708643  : -CTTCATTGGAAATCTTAACTTGTGTGGAAAGCATGTTGATGTCGTGTGCCAAGATGATAGTGGAAACCCTTCATCACATTCTCAATCAGGT------------------------------------------------ :  687
                                                                                                                                                               
                     *      1700         *      1720         *      1740         *      1760         *      1780         *      1800         *      1820       
Genomic   : CTATCAGTAAAGTTATATTTATTTGGTGTCTTATACTTTATAGGTCAAAATCAAAAGAAAAACTCTGGTAAGCTGCTGATTAGCGCATCAGCAACTGTAGGTGCGCTACTCCTAGTTGCACTCATGTGTTTCTGGGGATG : 1820
NM_102881 : ---------------------------------------------CAAAATCAAAAGAAAAACTCTGGTAAGCTGCTGATTAGCGCATCAGCAACTGTAGGTGCGCTACTCCTAGTTGCACTCATGTGTTTCTGGGGATG :  782
AK226234  : ---------------------------------------------CAAAATCAAAAGAAAAACTCTGGTAAGCTGCTGATTAGCGCATCAGCAACTGTAGGTGCGCTACTCCTAGTTGCACTCATGTGTTTCTGGGGATG :  782
FJ708643  : ---------------------------------------------CAAAATCAAAAGAAAAACTCTGGTAAGCTGCTGATTAGCGCATCAGCAACTGTAGGTGCGCTACTCCTAGTTGCACTCATGTGTTTCTGGGGATG :  782
                                                                                                                                                               
                     *      1840         *      1860         *      1880         *      1900         *      1920         *      1940         *      1960       
Genomic   : CTTTCTATATAAAAAGCTCGGTAAAGTCGAGATTAAAAGCCTTGCAAAGGATGTTGGTGGAGGTGAGCCGTATTGATTTTTTTTGTACAGATATTATCTCGTTAAATATTGGGAAATTTCATATTTTTCTCTATCTCCTA : 1960
NM_102881 : CTTTCTATATAAAAAGCTCGGTAAAGTCGAGATTAAAAGCCTTGCAAAGGATGTTGGTGGAGGTG--------------------------------------------------------------------------- :  847
AK226234  : CTTTCTATATAAAAAGCTCGGTAAAGTCGAGATTAAAAGCCTTGCAAAGGATGTTGGTGGAGGTG--------------------------------------------------------------------------- :  847
FJ708643  : CTTTCTATATAAAAAGCTCGGTAAAGTCGAGATTAAAAGCCTTGCAAAGGATGTTGGTGGAGGTG--------------------------------------------------------------------------- :  847
                                                                                                                                                               
             
                                                                                                                                                  
                     *      1980         *      2000         *      2020         *      2040         *      2060         *      2080         *      2100       
Genomic   : AATCTTCATTGTGACTTTTTCAATTCAGGTGCGTCTATTGTGATGTTCCACGGAGATTTACCTTACTCTTCCAAAGATATCATTAAGAAGCTGGAAATGTTGAATGAAGAGCACATAATTGGATGTGGAGGCTTTGGAAC : 2100
NM_102881 : -------------------------------CGTCTATTGTGATGTTCCACGGAGATTTACCTTACTCTTCCAAAGATATCATTAAGAAGCTGGAAATGTTGAATGAAGAGCACATAATTGGATGTGGAGGCTTTGGAAC :  956
AK226234  : -------------------------------CGTCTATTGTGATGTTCCACGGAGATTTACCTTACTCTTCCAAAGATATCATTAAGAAGCTGGAAATGTTGAATGAAGAGCACATAATTGGATGTGGAGGCTTTGGAAC :  956
FJ708643  : -------------------------------CGTCTATTGTGATGTTCCACGGAGATTTACCTTACTCTTCCAAAGATATCATTAAGAAGCTGGAAATGTTGAATGAAGAGCACATAATTGGATGTGGAGGCTTTGGAAC :  956
                                                                                                                                                               
                     *      2120         *      2140         *      2160         *      2180         *      2200         *      2220         *      2240       
Genomic   : GGTGTATAAGCTGGCCATGGATGATGGCAAAGTCTTTGCATTGAAGAGAATTCTAAAGTTAAACGAAGGGTTTGATCGGTTTTTTGAGCGGGAGCTTGAGATTCTTGGGAGCATCAAACACCGATACCTCGTGAATCTAC : 2240
NM_102881 : GGTGTATAAGCTGGCCATGGATGATGGCAAAGTCTTTGCATTGAAGAGAATTCTAAAGTTAAACGAAGGGTTTGATCGGTTTTTTGAGCGGGAGCTTGAGATTCTTGGGAGCATCAAACACCGATACCTCGTGAATCTAC : 1096
AK226234  : GGTGTATAAGCTGGCCATGGATGATGGCAAAGTCTTTGCATTGAAGAGAATTCTAAAGTTAAACGAAGGGTTTGATCGGTTTTTTGAGCGGGAGCTTGAGATTCTTGGGAGCATCAAACACCGATACCTCGTGAATCTAC : 1096
FJ708643  : GGTGTATAAGCTGGCCATGGATGATGGCAAAGTCTTTGCATTGAAGAGAATTCTAAAGTTAAACGAAGGGTTTGATCGGTTTTTTGAGCGGGAGCTTGAGATTCTTGGGAGCATCAAACACCGATACCTCGTGAATCTAC : 1096
                                                                                                                                                               
                     *      2260         *      2280         *      2300         *      2320         *      2340         *      2360         *      2380       
Genomic   : GTGGATATTGCAATTCACCAACATCAAAGCTACTGTTATATGATTACTTGCCCGGTGGTAGCCTTGATGAAGCACTTCATGGTATGATCAATCTGCTCTTTATGGCTCTCTTCCATATTATGAATGATTCACTGAGAAGT : 2380
NM_102881 : GTGGATATTGCAATTCACCAACATCAAAGCTACTGTTATATGATTACTTGCCCGGTGGTAGCCTTGATGAAGCACTTCATG----------------------------------------------------------- : 1177
AK226234  : GTGGATATTGCAATTCACCAACATCAAAGCTACTGTTATATGATTACTTGCCCGGTGGTAGCCTTGATGAAGCACTTCATG----------------------------------------------------------- : 1177
FJ708643  : GTGGATATTGCAATTCACCAACATCAAAGCTACTGTTATATGATTACTTGCCCGGTGGTAGCCTTGATGAAGCACTTCATG----------------------------------------------------------- : 1177
                                                                                                                                                               
                     *      2400         *      2420         *      2440         *      2460         *      2480         *      2500         *      2520       
Genomic   : TTGGTTCTTGAATTAGTAGAGAGAGGCGAGCAATTGGACTGGGATTCACGCGTAAATATCATCATAGGAGCTGCGAAAGGGTTATCATATTTGCATCATGATTGTTCTCCTCGGATTATACATCGTGATATAAAGTCGAG : 2520
NM_102881 : ----------------TAGAGAGAGGCGAGCAATTGGACTGGGATTCACGCGTAAATATCATCATAGGAGCTGCGAAAGGGTTATCATATTTGCATCATGATTGTTCTCCTCGGATTATACATCGTGATATAAAGTCGAG : 1301
AK226234  : ----------------TAGAGAGAGGCGAGCAATTGGACTGGGATTCACGCGTAAATATCATCATAGGAGCTGCGAAAGGGTTATCATATTTGCATCATGATTGTTCTCCTCGGATTATACATCGTGATATAAAGTCGAG : 1301
FJ708643  : -------------------AGAGAGGCGAGCAATTGGACTGGGATTCACGCGTAAATATCATCATAGGAGCTGCGAAAGGGTTATCATATTTGCATCATGATTGTTCTCCTCGGATTATACATCGTGATATAAAGTCGAG : 1298
                                                                                                                                                               
                     *      2540         *      2560         *      2580         *      2600         *      2620         *      2640         *      2660       
Genomic   : CAACATTTTGCTTGATGGCAATCTCGAGGCACGGGTATCGGATTTTGGTCTAGCCAAGTTGTTGGAAGACGAAGAATCTCATATCACAACCATTGTTGCAGGCACATTTGGGTACCTAGCTCCAGGTACATATGCCTTTT : 2660
NM_102881 : CAACATTTTGCTTGATGGCAATCTCGAGGCACGGGTATCGGATTTTGGTCTAGCCAAGTTGTTGGAAGACGAAGAATCTCATATCACAACCATTGTTGCAGGCACATTTGGGTACCTAGCTCCAG--------------- : 1426
AK226234  : CAACATTTTGCTTGATGGCAATCTCGAGGCACGGGTATCGGATTTTGGTCTAGCCAAGTTGTTGGAAGACGAAGAATCTCATATCACAACCATTGTTGCAGGCACATTTGGGTACCTAGCTCCAG--------------- : 1426
FJ708643  : CAACATTTTGCTTGATGGCAATCTCGAGGCACGGGTATCGGATTTTGGTCTAGCCAAGTTGTTGGAAGACGAAGAATCTCATATCACAACCATTGTTGCAGGCACATTTGGGTACCTAGCTCCAG--------------- : 1423
                                                                                                                                                               
                     *      2680         *      2700         *      2720         *      2740         *      2760         *      2780         *      2800       
Genomic   : AAAAGTCTTTACTTACATAAAGATTTGATCTTTCAAATGCTGAGACTTTTCCATTTGACAGAATATATGCAAAGTGGTAGAGCAACTGAGAAAACCGATGTATACAGTTTTGGGGTTTTGGTTCTTGAAGTCTTGAGTGG : 2800
NM_102881 : -------------------------------------------------------------AATATATGCAAAGTGGTAGAGCAACTGAGAAAACCGATGTATACAGTTTTGGGGTTTTGGTTCTTGAAGTCTTGAGTGG : 1505
AK226234  : -------------------------------------------------------------AATATATGCAAAGTGGTAGAGCAACTGAGAAAACCGATGTATACAGTTTTGGGGTTTTGGTTCTTGAAGTCTTGAGTGG : 1505
FJ708643  : -------------------------------------------------------------AATATATGCAAAGTGGTAGAGCAACTGAGAAAACCGATGTATACAGTTTTGGGGTTTTGGTTCTTGAAGTCTTGAGTGG : 1502
                                                                                                                                                               
                     *      2820         *      2840         *      2860         *      2880         *      2900         *      2920         *      2940       
Genomic   : GAAAAGACCAACCGATGCTTCCTTCATCGAGAAAGGCCTTAACGTTGTTGGTTGGGTATGCAAATAAGATTCTTTTTCAATACTGATATTTTGTTACGACAAACTTGACTAAAAAAGCTTTTGACTCATGATTTACAGCT : 2940
NM_102881 : GAAAAGACCAACCGATGCTTCCTTCATCGAGAAAGGCCTTAACGTTGTTGGTTGG-----------------------------------------------------------------------------------CT : 1562
AK226234  : GAAAAGACCAACCGATGCTTCCTTCATCGAGAAAGGCCTTAACGTTGTTGGTTGG-----------------------------------------------------------------------------------CT : 1562
FJ708643  : GAAAAGACCAACCGATGCTTCCTTCATCGAGAAAGGCCTTAACGTTGTTGGTTGG-----------------------------------------------------------------------------------CT : 1559
                                                                                                                                                               
                     *      2960         *      2980         *      3000         *      3020         *      3040         *      3060         *      3080       
Genomic   : AAAGTTCTTAATCAGCGAAAAACGGCCAAGGGATATTGTTGATCCAAACTGTGAAGGAATGCAGATGGAGAGCCTTGACGCTCTACTATCAATAGCAACACAGTGTGTGTCTCCGAGTCCAGAAGAGCGTCCAACGATGC : 3080
NM_102881 : AAAGTTCTTAATCAGCGAAAAACGGCCAAGGGATATTGTTGATCCAAACTGTGAAGGAATGCAGATGGAGAGCCTTGACGCTCTACTATCAATAGCAACACAGTGTGTGTCTCCGAGTCCAGAAGAGCGTCCAACGATGC : 1702
AK226234  : AAAGTTCTTAATCAGCGAAAAACGGCCAAGGGATATTGTTGATCCAAACTGTGAAGGAATGCAGATGGAGAGCCTTGACGCTCTACTATCAATAGCAACACAGTGTGTGTCTCCGAGTCCAGAAGAGCGTCCAACGATGC : 1702
FJ708643  : AAAGTTCTTAATCAGCGAAAAACGGCCAAGGGATATTGTTGATCCAAACTGTGAAGGAATGCAGATGGAGAGCCTTGACGCTCTACTATCAATAGCAACACAGTGTGTGTCTCCGAGTCCAGAAGAGCGTCCAACGATGC : 1699
                                                                                                                                                               
                     *      3100         *      3120         *      3140         *           
Genomic   : ATCGGGTTGTCCAGTTACTTGAATCAGAGGTCATGACTCCTTGTCCCAGCGAATTTTATGATTCCAGCTCTGAT : 3154
NM_102881 : ATCGGGTTGTCCAGTTACTTGAATCAGAGGTCATGACTCCTTGTCCCAGCGAATTTTATGATTCCAGCTCTGAT : 1776
AK226234  : ATCGG-TTGTCCAGTTACTTGAATCAGAGGTCATGACTCCTTGTCCCAGCGAATTTTATGATTCCAGCTCTGAT : 1775
FJ708643  : ATCGGGTTGTCCAGTTACTTGAATCAGAGGTCATGACTCCTTGTCCCAGCGAATTTTATGATTCCAGCTCTGAT : 1773


At1g34110
                                                                                                                                                               
                     *        20         *        40         *        60         *        80         *       100         *       120         *       140       
Genomic   : ATGGCCCAACCCACTCTTTCTCTTTCATCAGATGGCCAAGCTCTTCTCTCTCTCAAGAGACCATCACCATCACTCTTCTCTTCTTGGGACCCACAAGACCAGACACCATGTTCATGGTATGGCATTACATGTTCTGCAGA :  140
NM_103134 : ATGGCCCAACCCACTCTTTCTCTTTCATCAGATGGCCAAGCTCTTCTCTCTCTCAAGAGACCATCACCATCACTCTTCTCTTCTTGGGACCCACAAGACCAGACACCATGTTCATGGTATGGCATTACATGTTCTGCAGA :  140
FJ708644  : ATGGCCCAACCCACTCTTTCTCTTTCATCAGATGGCCAAGCTCTTCTCTCTCTCAAGAGACCATCACCATCACTCTTCTCTTCTTGGGACCCACAAGACCAGACACCATGTTCATGGTATGGCATTACATGTTCTGCAGA :  140
                                                                                                                                                               
                     *       160         *       180         *       200         *       220         *       240         *       260         *       280       
Genomic   : CAACAGAGTCATCTCTGTCTCTATCCCCGACACTTTCCTTAACCTGTCTTCAATCCCTGACCTCTCTTCTCTCTCCTCTCTACAGTTCCTAAACCTCTCCTCCACCAACCTCTCTGGCCCAATCCCTCCTTCATTTGGAA :  280
NM_103134 : CAACAGAGTCATCTCTGTCTCTATCCCCGACACTTTCCTTAACCTGTCTTCAATCCCTGACCTCTCTTCTCTCTCCTCTCTACAGTTCCTAAACCTCTCCTCCACCAACCTCTCTGGCCCAATCCCTCCTTCATTTGGAA :  280
FJ708644  : CAACAGAGTCATCTCTGTCTCTATCCCCGACACTTTCCTTAACCTGTCTTCAATCCCTGACCTCTCTTCTCTCTCCTCTCTACAGTTCCTAAACCTCTCCTCCACCAACCTCTCTGGCCCAATCCCTCCTTCATTTGGAA :  280
                                                                                                                                                               
                     *       300         *       320         *       340         *       360         *       380         *       400         *       420       
Genomic   : AACTTACCCACCTAAGGCTTCTCGACCTTTCTTCAAACTCTCTCTCTGGTCCAATCCCATCAGAGCTTGGTCGCCTCTCTACTCTCCAGTTCCTTATATTGAACGCCAACAAGCTCTCAGGTTCAATCCCTTCACAGATT :  420
NM_103134 : AACTTACCCACCTAAGGCTTCTCGACCTTTCTTCAAACTCTCTCTCTGGTCCAATCCCATCAGAGCTTGGTCGCCTCTCTACTCTCCAGTTCCTTATATTGAACGCCAACAAGCTCTCAGGTTCAATCCCTTCACAGATT :  420
FJ708644  : AACTTACCCACCTAAGGCTTCTCGACCTTTCTTCAAACTCTCTCTCTGGTCCAATCCCATCAGAGCTTGGTCGCCTCTCTACTCTCCAGTTCCTTATATTGAACGCCAACAAGCTCTCAGGTTCAATCCCTTCACAGATT :  420
                                                                                                                                                               
                     *       440         *       460         *       480         *       500         *       520         *       540         *       560       
Genomic   : TCGAATCTATTTGCCTTGCAAGTGCTTTGTCTTCAAGACAATCTGTTGAATGGTTCGATTCCTTCAAGTTTTGGCTCTTTGGTCTCTCTTCAGCAGTTCAGACTTGGTGGTAACACTAATCTCGGAGGTCCTATCCCTGC :  560
NM_103134 : TCGAATCTATTTGCCTTGCAAGTGCTTTGTCTTCAAGACAATCTGTTGAATGGTTCGATTCCTTCAAGTTTTGGCTCTTTGGTCTCTCTTCAGCAGTTCAGACTTGGTGGTAACACTAATCTCGGAGGTCCTATCCCTGC :  560
FJ708644  : TCGAATCTATTTGCCTTGCAAGTGCTTTGTCTTCAAGACAATCTGTTGAATGGTTCGATTCCTTCAAGTTTTGGCTCTTTGGTCTCTCTTCAGCAGTTCAGACTTGGTGGTAACACTAATCTCGGAGGTCCTATCCCTGC :  560
                                                                                                                                                                                                                                                                                                                              
                     *       580         *       600         *       620         *       640         *       660         *       680         *       700       
Genomic   : TCAGCTAGGGTTTCTGAAAAATTTGACAACTCTAGGGTTTGCAGCAAGTGGTTTGTCAGGTTCAATCCCTTCCACATTTGGGAATTTGGTAAATTTGCAAACTTTGGCTCTGTATGACACTGAGATCTCTGGTACCATTC :  700
NM_103134 : TCAGCTAGGGTTTCTGAAAAATTTGACAACTCTAGGGTTTGCAGCAAGTGGTTTGTCAGGTTCAATCCCTTCCACATTTGGGAATTTGGTAAATTTGCAAACTTTGGCTCTGTATGACACTGAGATCTCTGGTACCATTC :  700
FJ708644  : TCAGCTAGGGTTTCTGAAAAATTTGACAACTCTAGGGTTTGCAGCAAGTGGTTTGTCAGGTTCAATCCCTTCCACATTTGGGAATTTGGTAAATTTGCAAACTTTGGCTCTGTATGACACTGAGATCTCTGGTACCATTC :  700
                                                                                                                                                               
                     *       720         *       740         *       760         *       780         *       800         *       820         *       840       
Genomic   : CTCCTCAACTGGGTTTGTGCTCAGAGCTCAGGAACTTGTATTTGCACATGAACAAGCTCACTGGATCAATCCCAAAGGAACTGGGTAAGCTCCAGAAGATCACTAGCTTGCTTCTATGGGGGAATTCTTTATCTGGGGTC :  840
NM_103134 : CTCCTCAACTGGGTTTGTGCTCAGAGCTCAGGAACTTGTATTTGCACATGAACAAGCTCACTGGATCAATCCCAAAGGAACTGGGTAAGCTCCAGAAGATCACTAGCTTGCTTCTATGGGGGAATTCTTTATCTGGGGTC :  840
FJ708644  : CTCCTCAACTGGGTTTGTGCTCAGAGCTCAGGAACTTGTATTTGCACATGAACAAGCTCACTGGATCAATCCCAAAGGAACTGGGTAAGCTCCAGAAGATCACTAGCTTGCTTCTATGGGGGAATTCTTTATCTGGGGTC :  840
                                                                                                                                                               
                     *       860         *       880         *       900         *       920         *       940         *       960         *       980       
Genomic   : ATCCCACCAGAGATTTCCAACTGTTCTTCCCTCGTGGTGTTTGATGTTTCAGCTAACGATCTAACTGGCGATATTCCTGGAGATTTGGGGAAGCTTGTTTGGTTGGAGCAGCTTCAGTTGTCTGATAATATGTTTACCGG :  980
NM_103134 : ATCCCACCAGAGATTTCCAACTGTTCTTCCCTCGTGGTGTTTGATGTTTCAGCTAACGATCTAACTGGCGATATTCCTGGAGATTTGGGGAAGCTTGTTTGGTTGGAGCAGCTTCAGTTGTCTGATAATATGTTTACCGG :  980
FJ708644  : ATCCCACCAGAGATTTCCAACTGTTCTTCCCTCGTGGTGTTTGATGTTTCAGCTAACGATCTAACTGGCGATATTCCTGGAGATTTGGGGAAGCTTGTTTGGTTGGAGCAGCTTCAGTTGTCTGATAATATGTTTACCGG :  980
                                                                                                                                                               
                     *      1000         *      1020         *      1040         *      1060         *      1080         *      1100         *      1120       
Genomic   : TCAGATTCCTTGGGAGTTAAGCAACTGTTCCAGCCTCATTGCTCTTCAGCTGGACAAGAACAAGTTATCAGGCTCCATACCCTCACAAATTGGAAACCTCAAGTCTCTGCAGAGCTTTTTCTTGTGGGAGAACTCAATTT : 1120
NM_103134 : TCAGATTCCTTGGGAGTTAAGCAACTGTTCCAGCCTCATTGCTCTTCAGCTGGACAAGAACAAGTTATCAGGCTCCATACCCTCACAAATTGGAAACCTCAAGTCTCTGCAGAGCTTTTTCTTGTGGGAGAACTCAATTT : 1120
FJ708644  : TCAGATTCCTTGGGAGTTAAGCAACTGTTCCAGCCTCATTGCTCTTCAGCTGGACAAGAACAAGTTATCAGGCTCCATACCCTCACAAATTGGAAACCTCAAGTCTCTGCAGAGCTTTTTCTTGTGGGAGAACTCAATTT : 1120
                                                                                                                                                               
                     *      1140         *      1160         *      1180         *      1200         *      1220         *      1240         *      1260       
Genomic   : CAGGAACAATTCCGTCTTCTTTTGGCAACTGCACAGACCTAGTGGCCCTGGACCTCTCTAGGAACAAGCTAACAGGAAGAATACCAGAAGAGCTCTTCAGTTTAAAGAGGCTCAGCAAACTTCTCCTTCTAGGGAACTCC : 1260
NM_103134 : CAGGAACAATTCCGTCTTCTTTTGGCAACTGCACAGACCTAGTGGCCCTGGACCTCTCTAGGAACAAGCTAACAGGAAGAATACCAGAAGAGCTCTTCAGTTTAAAGAGGCTCAGCAAACTTCTCCTTCTAGGGAACTCC : 1260
FJ708644  : CAGGAACAATTCCGTCTTCTTTTGGCAACTGCACAGACCTAGTGGCCCTGGACCTCTCTAGGAACAAGCTAACAGGAAGAATACCAGAAGAGCTCTTCAGTTTAAAGAGGCTCAGCAAACTTCTCCTTCTAGGGAACTCC : 1260
                                                                                                                                                               
                     *      1280         *      1300         *      1320         *      1340         *      1360         *      1380         *      1400       
Genomic   : TTATCCGGTGGATTACCAAAGAGCGTAGCAAAATGCCAGTCGCTTGTGAGGCTAAGAGTCGGAGAGAACCAACTCTCAGGTCAAATCCCTAAAGAGATAGGCGAGTTACAGAACCTGGTGTTTCTTGATCTCTACATGAA : 1400
NM_103134 : TTATCCGGTGGATTACCAAAGAGCGTAGCAAAATGCCAGTCGCTTGTGAGGCTAAGAGTCGGAGAGAACCAACTCTCAGGTCAAATCCCTAAAGAGATAGGCGAGTTACAGAACCTGGTGTTTCTTGATCTCTACATGAA : 1400
FJ708644  : TTATCCGGTGGATTACCAAAGAGCGTAGCAAAATGCCAGTCGCTTGTGAGGCTAAGAGTCGGAGAGAACCAACTCTCAGGTCAAATCCCTAAAGAGATAGGCGAGTTACAGAACCTGGTGTTTCTTGATCTCTACATGAA : 1400
                                                                                                                                                               
                     *      1420         *      1440         *      1460         *      1480         *      1500         *      1520         *      1540       
Genomic   : CCATTTCTCCGGTGGGCTTCCTTACGAGATCTCTAACATCACTGTCCTTGAGCTCTTAGATGTCCACAACAACTACATCACCGGAGATATTCCTGCTCAGTTGGGGAACCTTGTGAATCTAGAGCAGCTTGATTTGAGCA : 1540
NM_103134 : CCATTTCTCCGGTGGGCTTCCTTACGAGATCTCTAACATCACTGTCCTTGAGCTCTTAGATGTCCACAACAACTACATCACCGGAGATATTCCTGCTCAGTTGGGGAACCTTGTGAATCTAGAGCAGCTTGATTTGAGCA : 1540
FJ708644  : CCATTTCTCCGGTGGGCTTCCTTACGAGATCTCTAACATCACTGTCCTTGAGCTCTTAGATGTCCACAACAACTACATCACCGGAGATATTCCTGCTCAGTTGGGGAACCTTGTGAATCTAGAGCAGCTTGATTTGAGCA : 1540
                                                                                                                                                               
                     *      1560         *      1580         *      1600         *      1620         *      1640         *      1660         *      1680       
Genomic   : GAAACAGCTTCACAGGAAACATACCGCTGAGTTTTGGGAACCTAAGCTACTTAAACAAGCTGATCCTCAACAACAATCTCCTCACTGGTCAAATCCCAAAATCTATCAAGAATCTACAAAAGCTTACACTTCTTGATCTT : 1680
NM_103134 : GAAACAGCTTCACAGGAAACATACCGCTGAGTTTTGGGAACCTAAGCTACTTAAACAAGCTGATCCTCAACAACAATCTCCTCACTGGTCAAATCCCAAAATCTATCAAGAATCTACAAAAGCTTACACTTCTTGATCTT : 1680
FJ708644  : GAAACAGCTTCACAGGAAACATACCGCTGAGTTTTGGGAACCTAAGCTACTTAAACAAGCTGATCCTCAACAACAATCTCCTCACTGGTCAAATCCCAAAATCTATCAAGAATCTACAAAAGCTTACACTTCTTGATCTT : 1680
                                                                                                                                                               
                     *      1700         *      1720         *      1740         *      1760         *      1780         *      1800         *      1820       
Genomic   : AGCTACAACAGCTTATCCGGTGAAATCCCACAAGAACTCGGTCAAGTCACAAGCTTAACTATAAACCTCGACTTAAGCTACAACACCTTCACTGGAAACATCCCGGAAACCTTCTCCGACCTCACACAGCTACAATCACT : 1820
NM_103134 : AGCTACAACAGCTTATCCGGTGAAATCCCACAAGAACTCGGTCAAGTCACAAGCTTAACTATAAACCTCGACTTAAGCTACAACACCTTCACTGGAAACATCCCGGAAACCTTCTCCGACCTCACACAGCTACAATCACT : 1820
FJ708644  : AGCTACAACAGCTTATCCGGTGAAATCCCACAAGAACTCGGTCAAGTCACAAGCTTAACTATAAACCTCGACTTAAGCTACAACACCTTCACTGGAAACATCCCGGAAACCTTCTCCGACCTCACACAGCTACAATCACT : 1820
                                                                                                                                                               
                     *      1840         *      1860         *      1880         *      1900         *      1920         *      1940         *      1960       
Genomic   : CGACCTCTCAAGCAACTCGCTACACGGCGACATCAAAGTCCTCGGTTCACTCACAAGCCTAGCTTCACTAAACATCTCCTGCAACAACTTCTCAGGTCCAATCCCATCAACACCATTCTTCAAAACAATCTCCACAACAT : 1960
NM_103134 : CGACCTCTCAAGCAACTCGCTACACGGCGACATCAAAGTCCTCGGTTCACTCACAAGCCTAGCTTCACTAAACATCTCCTGCAACAACTTCTCAGGTCCAATCCCATCAACACCATTCTTCAAAACAATCTCCACAACAT : 1960
FJ708644  : CGACCTCTCAAGCAACTCGCTACACGGCGACATCAAAGTCCTCGGTTCACTCACAAGCCTAGCTTCACTAAACATCTCCTGCAACAACTTCTCAGGTCCAATCCCATCAACACCATTCTTCAAAACAATCTCCACAACAT : 1960
                                                                                                                                                               
                     *      1980         *      2000         *      2020         *      2040         *      2060         *      2080         *      2100       
Genomic   : CATACCTCCAAAACACAAACCTCTGCCATTCACTAGACGGCATCACTTGCTCATCACACACCGGACAAAACAACGGAGTAAAATCCCCAAAGATCGTAGCTTTAACCGCAGTAATCCTCGCATCAATCACAATCGCAATC : 2100
NM_103134 : CATACCTCCAAAACACAAACCTCTGCCATTCACTAGACGGCATCACTTGCTCATCACACACCGGACAAAACAACGGAGTAAAATCCCCAAAGATCGTAGCTTTAACCGCAGTAATCCTCGCATCAATCACAATCGCAATC : 2100
FJ708644  : CATACCTCCAAAACACAAACCTCTGCCATTCACTAGACGGCATCACTTGCTCATCACACACCGGACAAAACAACGGAGTAAAATCCCCAAAGATCGTAGCTTTAACCGCAGTAATCCTCGCATCAATCACAATCGCAATC : 2100
                                                                                                                                                               
                     *      2120         *      2140         *      2160         *      2180         *      2200         *      2220         *      2240       
Genomic   : CTCGCAGCCTGGCTTCTCATCTTACGAAACAACCACCTCTACAAAACATCTCAAAACTCCTCTTCCTCTCCATCAACAGCAGAAGATTTCTCATACCCGTGGACATTCATACCATTCCAAAAACTCGGTATCACCGTCAA : 2240
NM_103134 : CTCGCAGCCTGGCTTCTCATCTTACGAAACAACCACCTCTACAAAACATCTCAAAACTCCTCTTCCTCTCCATCAACAGCAGAAGATTTCTCATACCCGTGGACATTCATACCATTCCAAAAACTCGGTATCACCGTCAA : 2240
FJ708644  : CTCGCAGCCTGGCTTCTCATCTTACGAAACAACCACCTCTACAAAACATCTCAAAACTCCTCTTCCTCTCCATCAACAGCAGAAGATTTCTCATACCCGTGGACATTCATACCATTCCAAAAACTCGGTATCACCGTCAA : 2240
                                                                                                                                                               
                     *      2260         *      2280         *      2300         *      2320         *      2340         *      2360         *      2380       
Genomic   : CAACATCGTCACTTCCTTAACCGACGAAAACGTAATCGGAAAAGGATGTTCAGGAATCGTATACAAAGCCGAAATCCCCAACGGAGACATCGTAGCAGTGAAGAAACTCTGGAAAACTAAAGACAACAACGAAGAAGGAG : 2380
NM_103134 : CAACATCGTCACTTCCTTAACCGACGAAAACGTAATCGGAAAAGGATGTTCAGGAATCGTATACAAAGCCGAAATCCCCAACGGAGACATCGTAGCAGTGAAGAAACTCTGGAAAACTAAAGACAACAACGAAGAAGGAG : 2380
FJ708644  : CAACATCGTCACTTCCTTAACCGACGAAAACGTAATCGGAAAAGGATGTTCAGGAATCGTATACAAAGCCGAAATCCCCAACGGAGACATCGTAGCAGTGAAGAAACTCTGGAAAACTAAAGACAACAACGAAGAAGGAG : 2380
                                                                                                                                                               
      
                     *      2400         *      2420         *      2440         *      2460         *      2480         *      2500         *      2520       
Genomic   : AATCAACAATAGATTCATTCGCTGCAGAGATTCAGATTCTCGGTAACATCAGACACAGAAACATAGTGAAGCTATTAGGTTACTGTTCGAACAAATCAGTGAAGCTGCTTCTATACAATTACTTCCCTAACGGGAATCTA : 2520
NM_103134 : AATCAACAATAGATTCATTCGCTGCAGAGATTCAGATTCTCGGTAACATCAGACACAGAAACATAGTGAAGCTATTAGGTTACTGTTCGAACAAATCAGTGAAGCTGCTTCTATACAATTACTTCCCTAACGGGAATCTA : 2520
FJ708644  : AATCAACAATAGATTCATTCGCTGCAGAGATTCAGATTCTCGGTAACATCAGACACAGAAACATAGTGAAGCTATTAGGTTACTGTTCGAACAAATCAGTGAAGCTGCTTCTATACAATTACTTCCCTAACGGGAATCTA : 2520
                                                                                                                                                               
                     *      2540         *      2560         *      2580         *      2600         *      2620         *      2640         *      2660       
Genomic   : CAACAGTTGTTGCAAGGAAATCGAAACTTGGATTGGGAAACACGTTACAAGATTGCGATTGGAGCTGCTCAGGGTTTGGCTTATCTTCATCATGATTGTGTTCCGGCGATTTTGCATAGAGATGTCAAGTGTAATAATAT : 2660
NM_103134 : CAACAGTTGTTGCAAGGAAATCGAAACTTGGATTGGGAAACACGTTACAAGATTGCGATTGGAGCTGCTCAGGGTTTGGCTTATCTTCATCATGATTGTGTTCCGGCGATTTTGCATAGAGATGTCAAGTGTAATAATAT : 2660
FJ708644  : CAACAGTTGTTGCAAGGAAATCGAAACTTGGATTGGGAAACACGTTACAAGATTGCGATTGGAGCTGCTCAGGGTTTGGCTTATCTTCATCATGATTGTGTTCCGGCGATTTTGCATAGAGATGTCAAGTGTAATAATAT : 2660
                                                                                                                                                               
                     *      2680         *      2700         *      2720         *      2740         *      2760         *      2780         *      2800       
Genomic   : TCTTCTGGATTCGAAATATGAAGCGATTCTTGCTGATTTTGGACTTGCGAAGCTGATGATGAATTCGCCGAATTATCATAACGCCATGTCTCGTGTTGCTGGTTCTTATGGATACATCGCACCAGGCAAGTTTCTCTCTT : 2800
NM_103134 : TCTTCTGGATTCGAAATATGAAGCGATTCTTGCTGATTTTGGACTTGCGAAGCTGATGATGAATTCGCCGAATTATCATAACGCCATGTCTCGTGTTGCTG--------------------------------------- : 2761
FJ708644  : TCTTCTGGATTCGAAATATGAAGCGATTCTTGCTGATTTTGGACTTGCGAAGCTGATGATGAATTCGCCGAATTATCATAACGCCATGTCTCGTGTTGCTGGTTCTTATGGATACATCGCACCAG--------------- : 2785
                                                                                                                                                               
                     *      2820         *      2840         *      2860         *      2880         *      2900         *      2920         *      2940       
Genomic   : TTTTAACGGGAGGGCCTTTTTGATAAATTTGATAAAGCTCAAGGTTTCATTTGCATTATTATAAGAGATAGGGGGTTTTAGTGTATTTATTAAAATTCAGAGCTTCATATCCATTATTACAAGAGATTGAGAGAAATTTT : 2940
NM_103134 : -------------------------------------------------------------------------------------------------------------------------------------------- :    -
FJ708644  : -------------------------------------------------------------------------------------------------------------------------------------------- :    -
                                                                                                                                                               
                     *      2960         *      2980         *      3000         *      3020         *      3040         *      3060         *      3080       
Genomic   : GAATGAATTTGTTAAAAAAACAAAAGGGTTTCTTGTGCATCATTACAAAATATTAGGATTTTTTTTTAATAATGAATTTGTTAAAATTTGAGGTTTCATTTGCATTATTGGCAAAAGATTGTTTTTTTTTTTATATATAG : 3080
NM_103134 : -------------------------------------------------------------------------------------------------------------------------------------------- :    -
FJ708644  : -------------------------------------------------------------------------------------------------------------------------------------------- :    -
                                                                                                                                                               
                     *      3100         *      3120         *      3140         *      3160         *      3180         *      3200         *      3220       
Genomic   : TGAATTTGTTAAAATCAGAGCTTGATTTTCATTACTACAAGAAATTGGGGGAAATTTGGAATGAATTTGTTTTAAAAAGTGTTACTTGTGCATTATTATAAAATAGTAGTTGTTTTTATTTGAAATTTAAGGTTTCACTT : 3220
NM_103134 : -------------------------------------------------------------------------------------------------------------------------------------------- :    -
FJ708644  : -------------------------------------------------------------------------------------------------------------------------------------------- :    -
                                                                                                                                                               
                     *      3240         *      3260         *      3280         *      3300         *      3320         *      3340         *      3360       
Genomic   : GCATTATTACAAAAGATTGTGTGTTTTTTTGGTAGTGAATTAATAAATTTGTTAAAAGCAGGGTTTTATTCATCTTTACAAAATATAGTTTTTTTTTTAATACTTTGTTAATATCAGGGTTTTATTTGCATTATTACAAA : 3360
NM_103134 : -------------------------------------------------------------------------------------------------------------------------------------------- :    -
FJ708644  : -------------------------------------------------------------------------------------------------------------------------------------------- :    -
                                                                                                                                                               
                     *      3380         *      3400         *      3420         *      3440         *      3460         *      3480         *      3500       
Genomic   : AACAATTGTGAGGTTTGTTAATGTGTTTGTTCAAAATCATGGTTTAATACATAAAAACAAAATACTGGGGCTTGCTTTCTGATAACGTAATTAATAAGAGGTTTTTTGTGTAATTATTGCAGAATACGGATACACGATGA : 3500
NM_103134 : --------------------------------------------------------------------------------------------------------------------------AATACGGATACACGATGA : 2779
FJ708644  : --------------------------------------------------------------------------------------------------------------------------AATACGGATACACGATGA : 2803
                                                                                                                                                               
                     *      3520         *      3540         *      3560         *      3580         *      3600         *      3620         *      3640       
Genomic   : ATATAACAGAGAAGAGTGACGTGTACAGCTACGGCGTCGTTTTATTAGAGATTCTAAGTGGCCGGAGCGCCGTGGAGCCACAAATCGGCGACGGGCTTCACATAGTGGAATGGGTGAAGAAGAAGATGGGAACCTTTGAA : 3640
NM_103134 : ATATAACAGAGAAGAGTGACGTGTACAGCTACGGCGTCGTTTTATTAGAGATTCTAAGTGGCCGGAGCGCCGTGGAGCCACAAATCGGCGACGGGCTTCACATAGTGGAATGGGTGAAGAAGAAGATGGGAACCTTTGAA : 2919
FJ708644  : ATATAACAGAGAAGAGTGACGTGTACAGCTACGGCGTCGTTTTATTAGAGATTCTAAGTGGCCGGAGCGCCGTGGAGCCACAAATCGGCGACGGGCTTCACATAGTGGAATGGGTGAAGAAGAAGATGGGAACCTTTGAA : 2943
                                                                                                                                                               
                     *      3660         *      3680         *      3700         *      3720         *      3740         *      3760         *      3780       
Genomic   : CCAGCTTTATCAGTACTTGACGTGAAGCTTCAGGGATTACCAGATCAGATAGTTCAAGAGATGCTTCAGACATTAGGAATCGCCATGTTCTGTGTGAATCCGTCGCCTGTGGAGAGACCAACGATGAAGGAAGTTGTGAC : 3780
NM_103134 : CCAGCTTTATCAGTACTTGACGTGAAGCTTCAGGGATTACCAGATCAGATAGTTCAAGAGATGCTTCAGACATTAGGAATCGCCATGTTCTGTGTGAATCCGTCGCCTGTGGAGAGACCAACGATGAAGGAAGTTGTGAC : 3059
FJ708644  : CCAGCTTTATCAGTACTTGACGTGAAGCTTCAGGGATTACCAGATCAGATAGTTCAAGAGATGCTTCAGACATTAGGAATCGCCATGTTCTGTGTGAATCCGTCGCCTGTGGAGAGACCAACGATGAAGGAAGTTGTGAC : 3083
                                                                                                                                                               
                     *      3800         *      3820         *      3840         *             
Genomic   : GTTGTTAATGGAGGTTAAGTGTAGTCCTGAAGAATGGGGTAAAACATCTCAGCCTCTTATCAAGCCTTCTTCTTCA : 3856
NM_103134 : GTTGTTAATGGAGGTTAAGTGTAGTCCTGAAGAATGGGGTAAAACATCTCAGCCTCTTATCAAGCCTTCTTCTTCA : 3135
FJ708644  : GTTGTTAATGGAGGTTAAGTGTAGTCCTGAAGAATGGGGTAAAACATCTCAGCCTCTTATCAAGCCTTCTTCTTCA : 3159


At1g51880
                                                                                                                                                               
                     *        20         *        40         *        60         *        80         *       100         *       120         *       140       
Genomic   : ATGAAGTCTATTCATGGGTTTTTGCTCTTCTTGATCACAGCTTATGTCATTTTGGAATCAGTTCAAGCTCAAGATCAATTAGGTTCTTACTTTTTGTTTCTTCGATTACTCTATAGATTAAGAAACCCTTTTGGAATTTG :  140
NM_104068 : ATGAAGTCTATTCATGGGTTTTTGCTCTTCTTGATCACAGCTTATGTCATTTTGGAATCAGTTCAAGCTCAAGATCAATTAGG--------------------------------------------------------- :   83
FJ708654  : ATGAAGTCTATTCATGGGTTTTTGCTCTTCTTGATCACAGCTTATGTCATTTTGGAATCAGTTCAAGCTCAAGATCAATTAGG--------------------------------------------------------- :   83
                                                                                                                                                               
                     *       160         *       180         *       200         *       220         *       240         *       260         *       280       
Genomic   : TTTCTTTAAATTTGTTATTTTGGTGTTGATCGTCAACAGGATTCATCAGTTTGGATTGTGGGTTAGTCCCTAAGAACGCAACTTATACGGAGAAGACGACAAATATAACATACAAATCAGATGCAAATTACATCGATAGT :  280
NM_104068 : ----------------------------------------ATTCATCAGTTTGGATTGTGGGTTAGTCCCTAAGAACGCAACTTATACGGAGAAGACGACAAATATAACATACAAATCAGATGCAAATTACATCGATAGT :  183
FJ708654  : ----------------------------------------ATTCATCAGTTTGGATTGTGGGTTAGTCCCTAAGAACGCAACTTATACGGAGAAGACGACAAATATAACATACAAATCAGATGCAAATTACATCGATAGT :  183
                                                                                                                                                               
                     *       300         *       320         *       340         *       360         *       380         *       400         *       420       
Genomic   : GGATTGGTCGGGAGGATCAGTGCTGAGTACAAAGCGCAGCTTCAGCAACAAACTTGGACCGTAAGAAGCTTCCCTGAGGGTGAAAGAAACTGTTACAACTTCAACCTTACCGCTAAAAGCAGATATCTCATCAGAGCAAC :  420
NM_104068 : GGATTGGTCGGGAGGATCAGTGCTGAGTACAAAGCGCAGCTTCAGCAACAAACTTGGACCGTAAGAAGCTTCCCTGAGGGTGAAAGAAACTGTTACAACTTCAACCTTACCGCTAAAAGCAGATATCTCATCAGAGCAAC :  323
FJ708654  : GGATTGGTCGGGAGGATCAGTGCTGAGTACAAAGCGCAGCTTCAGCAACAAACTTGGACTGTAAGAAGCTTCCCTGAGGGTGAAAGAAACTGTTACAACTTCAACCTTACCGCTAAAAGCAGATATCTCATCAGAGCAAC :  323
                                                                                                                                                               
                     *       440         *       460         *       480         *       500         *       520         *       540         *       560       
Genomic   : CTTTACGTATGGGAATTACGATGGTTTGAGACAAGTCCCCAAGTTTGATATTCACATTGGTCCTAGCAAATGGACTTCTGTTAAGTTGGATGGAGTAGGAAATGGTGCAGTCCTCGAGATGATTCATGTCTTAACACAAG :  560
NM_104068 : CTTTACGTATGGGAATTACGATGGTTTGAGACAAGTCCCCAAGTTTGATATTCACATTGGTCCTAGCAAATGGACTTCTGTTAAGTTGGATGGAGTAGGAAATGGTGCAGTCCTCGAGATGATTCATGTCTTAACACAAG :  463
FJ708654  : CTTTACGTATGGGAATTACGATGGTTTGAGACAAGTCCCCAAGTTTGATATTCACATTGGTCCTAGCAAATGGACTTCTGTTAAGTTGGATGGAGTAGGAAATGGTGCAGTCCTCGAGATGATTCATGTCTTAACACAAG :  463
                                                                                                                                                                                                                                                                                                                              
                     *       580         *       600         *       620         *       640         *       660         *       680         *       700       
Genomic   : ACCGTCTTCAAATTTGTCTTGTTAAGACAGGAAAAGGGATACCATTTATTTCGTCGCTGGAACTTCGTCCGTTGAACAATAATACTTACCTCACGCAAAGTGGATCGTTGATTGGGTTCGCAAGAGTTTTCTTTTCAGCC :  700
NM_104068 : ACCGTCTTCAAATTTGTCTTGTTAAGACAGGAAAAGGGATACCATTTATTTCGTCGCTGGAACTTCGTCCGTTGAACAATAATACTTACCTCACGCAAAGTGGATCGTTGATTGGGTTCGCAAGAGTTTTCTTTTCAGCC :  603
FJ708654  : ACCGTCTTCAAATTTGTCTTGTTAAGACAGGAAAAGGGATACCATTTATTTCGTCGCTGGAACTTCGTCCGTTGAACAATAATACTTACCTCACGCAAAGTGGATCGTTGATTGGGTTCGCAAGAGTTTTCTTTTCAGCC :  603
                                                                                                                                                               
                     *       720         *       740         *       760         *       780         *       800         *       820         *       840       
Genomic   : ACTCCAACATTCATAAGGTAAAGAGGTTTTTTTTTTTTTTTTTTTTTCTTTTGTATTTTTTCTGGTTCGTTTCTGCGTGTAGATGCAAATCATGCAATATCTTGATGTAAAGTGCACCAGATATTGCTAGCTAGTTTGAA :  840
NM_104068 : ACTCCAACATTCATAAGGTA------------------------------------------------------------------------------------------------------------------------ :  623
FJ708654  : ACTCCAACATTCATAAGGTA------------------------------------------------------------------------------------------------------------------------ :  623
                                                                                                                                                               
                     *       860         *       880         *       900         *       920         *       940         *       960         *       980       
Genomic   : AAATTTGGTATTTGCATTCGGATTTTCGGATGCAAAACGTTTTATCCAAATGCAACATGCATATGGAAAAAGCGATTTTAGAAACGAACAACATTAACGGCTAGATCTGGATGTTGCATTTAACGACGGGACTTTCTTTT :  980
NM_104068 : -------------------------------------------------------------------------------------------------------------------------------------------- :    -
FJ708654  : -------------------------------------------------------------------------------------------------------------------------------------------- :    -
                                                                                                                                                               
                     *      1000         *      1020         *      1040         *      1060         *      1080         *      1100         *      1120       
Genomic   : TTTTGTTATTTGTTTTTGAGTTAACAAAAACAAGTCTAGTGGCTTTTCCTCTCATTTCTATAGGTATGATGAGGATATCCATGATCGAGTCTGGGTTCGACAGTTTGGTAATGGATTGAAATCGATAAGCACAGACCTTT : 1120
NM_104068 : ------------------------------------------------------------------TGATGAGGATATCCATGATCGAGTCTGGGTTCGACAGTTTGGTAATGGATTGAAATCGATAAGCACAGACCTTT :  697
FJ708654  : ------------------------------------------------------------------TGATGAGGATATCCATGATCGAGTCTGGGTTCGACAGTTTGGTAATGGATTGAAATCGATAAGCACAGACCTTT :  697
                                                                                                                                                               
                     *      1140         *      1160         *      1180         *      1200         *      1220         *      1240         *      1260       
Genomic   : TGGTCGATACAAGTAACCCCTATGATGTGCCACAAGCTGTGGCAAAGACTGCATGTGTACCTTCAAATGCTAGTCAGCCTCTGATCTTTGATTGGACTCTTGACAACATCACTTCACAATCATATGTATACATGCATTTC : 1260
NM_104068 : TGGTCGATACAAGTAACCCCTATGATGTGCCACAAGCTGTGGCAAAGACTGCATGTGTACCTTCAAATGCTAGTCAGCCTCTGATCTTTGATTGGACTCTTGACAACATCACTTCACAATCATATGTATACATGCATTTC :  837
FJ708654  : TGGTCGATACAAGTAACCCCTATGATGTGCCACAAGCTGTGGCAAAGACTGCATGTGTACCTTCAAATGCTAGTCAGCCTCTGATCTTTGATTGGACTCTTGACAACATCACTTCACAATCATATGTATACATGCATTTC :  837
                                                                                                                                                               
                     *      1280         *      1300         *      1320         *      1340         *      1360         *      1380         *      1400       
Genomic   : GCTGAAATCCAGACTCTTAAGGATAATGACATCAGAGAATTCAACATTACTTATAATGGTGGTCAGAATGTGTACTCCTATTTGAGGCCTGAAAAATTCGAGATATCAACTCTATTCGATTCAAAGCCTTTGAGTTCTCC : 1400
NM_104068 : GCTGAAATCCAGACTCTTAAGGATAATGACATCAGAGAATTCAACATTACTTATAATGGTGGTCAGAATGTGTACTCCTATTTGAGGCCTGAAAAATTCGAGATATCAACTCTATTCGATTCAAAGCCTTTGAGTTCTCC :  977
FJ708654  : GCTGAAATCCAGACTCTTAAGGATAATGACATCAGAGAATTCAACATTACTTATAATGGTGGTCAGAATGTGTACTCCTATTTGAGGCCTGAAAAATTCGAGATATCAACTCTATTCGATTCAAAGCCTTTGAGTTCTCC :  977
                                                                                                                                                               
                     *      1420         *      1440         *      1460         *      1480         *      1500         *      1520         *      1540       
Genomic   : GGATGGCAGTTTCAGTTTGTCTTTCACGAAGACCGGTAACTCAACTCTTCCTCCTCTTATCAACGGCCTCGAGATTTATAAAGTCTTAGACCTTCTAGAACTCGAGACAGATCAAGATGAAGGCAAGTTTAGCATGTTCT : 1540
NM_104068 : GGATGGCAGTTTCAGTTTGTCTTTCACGAAGACCGGTAACTCAACTCTTCCTCCTCTTATCAACGGCCTCGAGATTTATAAAGTCTTAGACCTTCTAGAACTCGAGACAGATCAAGATGAAG------------------ : 1099
FJ708654  : GGATGGCAGTTTCAGTTTGTCTTTCACGAAGACCGGTAACTCAACTCTTCCTCCTCTTATCAACGGCCTCGAGATTTATAAAGTCTTAGACCTTCTAGAACTCGAGACAGATCAAGATGAAG------------------ : 1099
                                                                                                                                                               
                     *      1560         *      1580         *      1600         *      1620         *      1640         *      1660         *      1680       
Genomic   : TAAACCCATTTTTTACCAAAAGTTTCATGTCTTATAACTTTCTTTTGTTTTTCAAATGTGATGATTGATCAGTTTCTGCTATGATAAACATCAAGGCAACGTATGATTTGAGCAAAAAGGTTAGCTGGCAAGGAGATCCA : 1680
NM_104068 : ------------------------------------------------------------------------TTTCTGCTATGATAAACATCAAGGCAACGTATGATTTGAGCAAAAAGGTTAGCTGGCAAGGAGATCCA : 1167
FJ708654  : ------------------------------------------------------------------------TTTCTGCTATGATAAACATCAAGGCAACGTATGATTTGAGCAAAAAGGTTAGCTGGCAAGGAGATCCA : 1167
                                                                                                                                                               
                     *      1700         *      1720         *      1740         *      1760         *      1780         *      1800         *      1820       
Genomic   : TGTGCTCCTAAGTCTTATCAGTGGGAAGGTTTAAACTGCAGTTATCCAAACTCTGATCAACCTCGGATCATATCCTTGTATGTTCTGTTTCTTGATCTCATGGTCTTTCAATTTCAAGGTTTCACATATGCTAACTCTTT : 1820
NM_104068 : TGTGCTCCTAAGTCTTATCAGTGGGAAGGTTTAAACTGCAGTTATCCAAACTCTGATCAACCTCGGATCATATCCTTG-------------------------------------------------------------- : 1245
FJ708654  : TGTGCTCCTAAGTCTTATCAGTGGGAAGGTTTAAACTGCAGTTATCCAAACTCTGATCAACCTCGGATCATATCCTTG-------------------------------------------------------------- : 1245
                                                                                                                                                               
                     *      1840         *      1860         *      1880         *      1900         *      1920         *      1940         *      1960       
Genomic   : TGGATACTTTATTGTTTGACAACATTACATTAGGAACTTGGCAGAGAACAAGTTGACCGGTACCATAACACCTGAAATATCCAAGCTAACACAGTTGATAGAGCTGTAAGTGCCCTGACACACCGATTTTATTGTGGTGA : 1960
NM_104068 : ----------------------------------AACTTGGCAGAGAACAAGTTGACCGGTACCATAACACCTGAAATATCCAAGCTAACACAGTTGATAGAGCT----------------------------------- : 1316
FJ708654  : ----------------------------------AACTTGGCAGAGAACAAGTTGACCGGTACCATAACACCTGAAATATCCAAGCTAACACAGTTGATAGAGCT----------------------------------- : 1316
                                                                                                                                                               
                     *      1980         *      2000         *      2020         *      2040         *      2060         *      2080         *      2100       
Genomic   : GAGAAGTATCTAACTCTTCAAAAACTTTGCTTTCTGTGTCTAATGGACAGAGATTTGTCAAAAAACGATTTATCAGGAGAGATACCGGAGTTTTTTGCAGATATGAAGTTATTAAAACTTATGTAAGCTTCTTTATCAGC : 2100
NM_104068 : --------------------------------------------------AGATTTGTCAAAAAACGATTTATCAGGAGAGATACCGGAGTTTTTTGCAGATATGAAGTTATTAAAACTTAT------------------ : 1388
FJ708654  : --------------------------------------------------AGATTTGTCAAAAAACGATTTATCAGGAGAGATACCGGAGTTTTTTGCAGATATGAAGTTATTAAAACTTAT------------------ : 1388
                                                                                                                                                               
                     *      2120         *      2140         *      2160         *      2180         *      2200         *      2220         *      2240       
Genomic   : ACCCTCTCTCTTCCATTTTTTTTTTTTTTTTTTTTTGGTTCAGAAAGCTTAATGTTTTCATTTGCAGAAACTTAAGTGGAAATCTAGGTCTCAACAGCACAATTCCAGACTCTATTCAACAAAGATTAGATAGCAAATCT : 2240
NM_104068 : -------------------------------------------AAAGCTTAATGTTTTCATTTGCAGAAACTTAAGTGGAAATCTAGGTCTCAACAGCACAATTCCAGACTCTATTCAACAAAGATTAGATAGCAAATCT : 1485
FJ708654  : -------------------------------------------------------------------AAACTTAAGTGGAAATCTAGGTCTCAACAGCACAATTCCAGACTCTATTCAACAAAGATTAGATAGCAAATCT : 1461
                                                                                                                                                               
                     *      2260         *      2280         *      2300         *      2320         *      2340         *      2360         *      2380       
Genomic   : CTAATACTAATGTAAGATGTTTATATTTCACTTGATTCAATCTCAAAATTTCTGAGTAATGAAAACAATATTAAACTTCATATCTCTTTATTTCTTGTTTCCGGTTTCCCAACACTTTAATGGTTATAGTTTGAGTAAGA : 2380
NM_104068 : CTAATACTAAT----------------------------------------------------------------------------------------------------------------------TTTGAGTAAGA : 1507
FJ708654  : CTAATACTAAT----------------------------------------------------------------------------------------------------------------------TTTGAGTAAGA : 1483
                                                                                                                                                               
                     
                     *      2400         *      2420         *      2440         *      2460         *      2480         *      2500         *      2520       
Genomic   : CTGTTACTAAAACTGTCACCCTGAAAGGCAAAAGTAAAAAGGTTCCAATGATTCCTATCGTAGCGTCAGTGGCTGGTGTGTTCGCTCTGCTAGTTATCTTGGCCATCTTTTTCGTCGTTAGAAGGAAAAACGGGGAAAGT : 2520
NM_104068 : CTGTTACTAAAACTGTCACCCTGAAAGGCAAAAGTAAAAAGGTTCCAATGATTCCTATCGTAGCGTCAGTGGCTGGTGTGTTCGCTCTGCTAGTTATCTTGGCCATCTTTTTCGTCGTTAGAAGGAAAAACGGGGAAAGT : 1647
FJ708654  : CTGTTACTAAAACTGTCACCCTGAAAGGCAAAAGTAAAAAGGTTCCAATGATTCCTATCGTAGCGTCAGTGGCTGGTGTGTTCGCTCTGCTAGTTATCTTGGCCATCTTTTTCGTCGTTAGAAGGAAAAACGGGGAAAGT : 1623
                                                                                                                                                               
                     *      2540         *      2560         *      2580         *      2600         *      2620         *      2640         *      2660       
Genomic   : AATAAGGGTACGAATCCATCAATCATAACAAAGGAACGCAGGATCACGTATCCCGAGGTACTAAAGATGACTAATAACTTTGAGAGAGTTCTTGGTAAAGGAGGATTTGGAACAGTGTACCATGGAAACTTGGAGGATAC : 2660
NM_104068 : AATAAGGGTACGAATCCATCAATCATAACAAAGGAACGCAGGATCACGTATCCCGAGGTACTAAAGATGACTAATAACTTTGAGAGAGTTCTTGGTAAAGGAGGATTTGGAACAGTGTACCATGGAAACTTGGAGGATAC : 1787
FJ708654  : AATAAGGGTACGAATCCATCAATCATAACAAAGGAACGCAGGATCACGTATCCCGAGGTACTAAAGATGACTAATAACTTTGAGAGAGTTCTTGGTAAAGGAGGATTTGGAACAGTGTACCATGGAAACTTGGAGGATAC : 1763
                                                                                                                                                               
                     *      2680         *      2700         *      2720         *      2740         *      2760         *      2780         *      2800       
Genomic   : TCAAGTAGCTGTGAAAATGCTCTCTCATTCGTCAGCTCAAGGGTATAAAGAGTTCAAAGCAGAGGTACTTTAACAAGAAATGGATTGGATCGGTTAAGTCCGTTTTATTTTAAAACATTACTTTCTCAATCGTGAAGTTG : 2800
NM_104068 : TCAAGTAGCTGTGAAAATGCTCTCTCATTCGTCAGCTCAAGGGTATAAAGAGTTCAAAGCAGAGGT-------------------------------------------------------------------------- : 1853
FJ708654  : TCAAGTAGCTGTGAAAATGCTCTCTCATTCGTCAGCTCAAGGGTATAAAGAGTTCAAAGCAGAGGT-------------------------------------------------------------------------- : 1829
                                                                                                                                                               
                     *      2820         *      2840         *      2860         *      2880         *      2900         *      2920         *      2940       
Genomic   : TCATTTTATTATATTTTTTAGGTTGAACTTCTTTTAAGAGTTCACCATAGAAATTTGGTGGGACTTGTGGGTTACTGTGATGATGGAGATAACTTGGCTTTGATTTATGAATACATGGCTAATGGAGATCTCAAGGAGAA : 2940
NM_104068 : -----------------------TGAACTTCTTTTAAGAGTTCACCATAGAAATTTGGTGGGACTTGTGGGTTACTGTGATGATGGAGATAACTTGGCTTTGATTTATGAATACATGGCTAATGGAGATCTCAAGGAGAA : 1970
FJ708654  : -----------------------TGAACTTCTTTTAAGAGTTCACCATAGAAATTTGGTGGGACTTGTGGGTTACTGTGATGATGGAGATAACTTGGCTTTGATTTATGAATACATGGCTAATGGAGATCTCAAGGAGAA : 1946
                                                                                                                                                               
                     *      2960         *      2980         *      3000         *      3020         *      3040         *      3060         *      3080       
Genomic   : TATGTCAGGTAACATTTTAAATTTAGAGACTCTAATTTAATCGAATCCATATCTTTCTGCGGTTTAAAATATCCTAGTATAGGAAAACGTGGAGGCAATGTCCTTACCTGGGAAAACAGGATGCAAATAGCTGTAGAGGC : 3080
NM_104068 : TATGTCAGG--------------------------------------------------------------------------AAAACGTGGAGGCAATGTCCTTACCTGGGAAAACAGGATGCAAATAGCTGTAGAGGC : 2036
FJ708654  : TATGTCAGG--------------------------------------------------------------------------AAAACGTGGAGGCAATGTCCTTACCTGGGAAAACAGGATGCAAATAGCTGTAGAGGC : 2012
                                                                                                                                                               
                     *      3100         *      3120         *      3140         *      3160         *      3180         *      3200         *      3220       
Genomic   : TGCACAAGGTGAACAAAATTATGACTTGTTTTCTTATTACACAACAAACTGCGTTTGGTTATCTTAGAGAAAGATTGACATATACAATAACAACTTGTAACAGGATTGGAGTATCTTCACAATGGATGTACGCCTCCTAT : 3220
NM_104068 : TGCACAAGG-----------------------------------------------------------------------------------------------ATTGGAGTATCTTCACAATGGATGTACGCCTCCTAT : 2081
FJ708654  : TGCACAAGG-----------------------------------------------------------------------------------------------ATTGGAGTATCTTCACAATGGATGTACGCCTCCTAT : 2057
                                                                                                                                                               
                     *      3240         *      3260         *      3280         *      3300         *      3320         *      3340         *      3360       
Genomic   : GGTCCATAGAGATGTTAAAACTACCAACATCTTATTGAATGAGCGGTATGGAGCAAAACTAGCCGACTTTGGGCTCTCAAGATCTTTCCCAGTTGATGGCGAAAGTCATGTCTCGACAGTGGTTGCGGGCACACCTGGTT : 3360
NM_104068 : GGTCCATAGAGATGTTAAAACTACCAACATCTTATTGAATGAGCGGTATGGAGCAAAACTAGCCGACTTTGGGCTCTCAAGATCTTTCCCAGTTGATGGCGAAAGTCATGTCTCGACAGTGGTTGCGGGCACACCTGGTT : 2221
FJ708654  : GGTCCATAGAGATGTTAAAACTACCAACATCTTATTGAATGAGCGGTATGGAGCAAAACTAGCCGACTTTGGGCTCTCAAGATCTTTCCCAGTTGATGGCGAAAGTCATGTCTCGACAGTGGTTGCGGGCACACCTGGTT : 2197
                                                                                                                                                               
                     *      3380         *      3400         *      3420         *      3440         *      3460         *      3480         *      3500       
Genomic   : ACCTTGACCCTGAGTAAGTGAATCAACCAGTTATCATCACTCATGTTAGTTGTGTTTCTCAAATAAATATTCAATGATGTCAATGTAATATTTCTATTCTCTCTCAGGTACTACAGAACAAACTGGCTAAGCGAGAAGAG : 3500
NM_104068 : ACCTTGACCCTGAGTA----------------------------------------------------------------------------------------------CTACAGAACAAACTGGCTAAGCGAGAAGAG : 2267
FJ708654  : ACCTTGACCCTGAGTA----------------------------------------------------------------------------------------------CTACAGAACAAACTGGCTAAGCGAGAAGAG : 2243
                                                                                                                                                               
                     *      3520         *      3540         *      3560         *      3580         *      3600         *      3620         *      3640       
Genomic   : TGATGTGTACAGCTTCGGTGTAGTCCTATTAGAGATAGTCACAAACCAGCCTGTGACAGATAAAACAAGAGAGAGAACTCACATCAATGAATGGGTTGGGTCCATGCTCACTAAAGGGGACATCAAGAGCATCCTTGACC : 3640
NM_104068 : TGATGTGTACAGCTTCGGTGTAGTCCTATTAGAGATAGTCACAAACCAGCCTGTGACAGATAAAACAAGAGAGAGAACTCACATCAATGAATGGGTTGGGTCCATGCTCACTAAAGGGGACATCAAGAGCATCCTTGACC : 2407
FJ708654  : TGATGTGTACAGCTTCGGTGTAGTCCTATTAGAGATAGTCACAAACCAGCCTGTGACAGATAAAACAAGAGAGAGAACTCACATCAATGAATGGGTTGGGTCCATGCTCACTAAAGGGGACATCAAGAGCATCCTTGACC : 2383
                                                                                                                                                               
                     *      3660         *      3680         *      3700         *      3720         *      3740         *      3760         *      3780       
Genomic   : CAAAACTGATGGGGGACTATGACACAAACGGTGCCTGGAAGATTGTTGAGCTGGCTCTTGCATGTGTGAACCCGTCTTCAAACCGGAGACCAACAATGGCACACGTCGTGACTGAGCTAAACGAATGTGTGGCCTTAGAA : 3780
NM_104068 : CAAAACTGATGGGGGACTATGACACAAACGGTGCCTGGAAGATTGTTGAGCTGGCTCTTGCATGTGTGAACCCGTCTTCAAACCGGAGACCAACAATGGCACACGTCGTGACTGAGCTAAACGAATGTGTGGCCTTAGAA : 2547
FJ708654  : CAAAACTGATGGGGGACTATGACACAAACGGTGCCTGGAAGATTGTTGAGCTGGCTCTTGCATGTGTGAACCCGTCTTCAAACCGGAGACCAACAATGGCACACGTCGTGACTGAGCTAAACGAATGTGTGGCCTTAGAA : 2523
                                                                                                                
                     *      3800         *      3820         *      3840         *      3860         *          
Genomic   : AATGCAAGGCGACAAGGTCGTGAAGAGATGCACACAAGTGGTTATGTTGACTTTAGTCGCTCTTCTGCTTCCGAATTTTCCCCTGGAGCCAGA : 3873
NM_104068 : AATGCAAGGCGACAAGGTCGTGAAGAGATGCACACAAGTGGTTATGTTGACTTTAGTCGCTCTTCTGCTTCCGAATTTTCCCCTGGAGCCAGA : 2640
FJ708654  : AATGCAAGGCGACAAGGTCGTGAAGAGATGCACACAAGTGGTTATGTTGACTTTAGTCGCTCTTCTGCTTCCGAATTTTCCCCTGGAGCCAGA : 2616


At1g51890
                                                                                                                                                               
                     *        20         *        40         *        60         *        80         *       100         *       120         *       140       
Genomic   : ATGAGGTTTTTGTCTTTCTTGATCTTCGTTTTCGCAGTTCTTGGATTGGTTCAAGCTCAAGACCAATCAGGTTATTTACTCTGTTTTCATGAAATCTTGATGTTTTGTTTCCTTTTCAGATCAACAAAACCTCTTAAAAG :  140
NM_104069 : ATGAGGTTTTTGTCTTTCTTGATCTTCGTTTTCGCAGTTCTTGGATTGGTTCAAGCTCAAGACCAATCAGG--------------------------------------------------------------------- :   71
FJ708655  : ATGAGGTTTTTGTCTTTCTTGATCTTCGTTTTCGCAGTTCTTGGATTGGTTCAAGCTCAAGACCAATCAGG--------------------------------------------------------------------- :   71
                                                                                                                                                               
                     *       160         *       180         *       200         *       220         *       240         *       260         *       280       
Genomic   : TTTGTTTTTTTCGATCATAAACAGGATTCATAAGCTTAGATTGTGGTTTGGTGCCTACGGAAATTACTTATGTGGAAAAGTCGACGAATATAACATACAGATCAGACGCAACTTACATCGACAGTGGAGTTCCCGGGAAG :  280
NM_104069 : -------------------------ATTCATAAGCTTAGATTGTGGTTTGGTGCCTACGGAAATTACTTATGTGGAAAAGTCGACGAATATAACATACAGATCAGACGCAACTTACATCGACAGTGGAGTTCCCGGGAAG :  186
FJ708655  : -------------------------ATTCATAAGCTTAGATTGTGGTTTGGTGCCTACGGAAATTACTTATGTGGAAAAGTCGACGAATATAACATACAGATCAGACGCAACTTACATCGACAGTGGAGTTCCCGGGAAG :  186
                                                                                                                                                               
                     *       300         *       320         *       340         *       360         *       380         *       400         *       420       
Genomic   : ATCAATGAAGTGTACAGAACACAGTTTCAGCAACAAATTTGGGCCTTGAGAAGCTTCCCTGAGGGTCAAAGAAATTGTTACAACTTCAGTCTCACCGCAAAACGTAAGTATCTAATCAGAGGAACCTTTATCTATGGGAA :  420
NM_104069 : ATCAATGAAGTGTACAGAACACAGTTTCAGCAACAAATTTGGGCCTTGAGAAGCTTCCCTGAGGGTCAAAGAAATTGTTACAACTTCAGTCTCACCGCAAAACGTAAGTATCTAATCAGAGGAACCTTTATCTATGGGAA :  326
FJ708655  : ATCAATGAAGTGTACAGAACACAGTTTCAGCAACAAATTTGGGCCTTGAGAAGCTTCCCTGAGGGTCAAAGAAATTGTTACAACTTCAGTCTCACCGCAAAACGTAAGTATCTAATCAGAGGAACCTTTATCTATGGGAA :  326
                                                                                                                                                               
                     *       440         *       460         *       480         *       500         *       520         *       540         *       560       
Genomic   : TTATGACGGTTTGAATCAACTCCCGAGCTTTGATCTTTACATCGGTCCAAACAAATGGACCTCTGTTTCGATCCCCGGAGTGAGAAATGGTTCAGTCTCCGAGATGATCCATGTCTTAAGACAAGACCATCTTCAAATTT :  560
NM_104069 : TTATGACGGTTTGAATCAACTCCCGAGCTTTGATCTTTACATCGGTCCAAACAAATGGACCTCTGTTTCGATCCCCGGAGTGAGAAATGGTTCAGTCTCCGAGATGATCCATGTCTTAAGACAAGACCATCTTCAAATTT :  466
FJ708655  : TTATGACGGTTTGAATCAACTCCCGAGCTTTGATCTTTACATCGGTCCAAACAAATGGACCTCTGTTTCGATCCCCGGAGTGAGAAATGGTTCAGTCTCCGAGATGATCCATGTCTTAAGACAAGACCATCTTCAAATTT :  466
                                                                                                                                                               
                     *       580         *       600         *       620         *       640         *       660         *       680         *       700       
Genomic   : GTCTTGTGAAAACAGGAGAAACTACACCGTTTATTTCTTCATTGGAACTTCGTCCTTTGAACAATAATACATACGTCACAAAAAGTGGATCGCTTATTGTGGTCGCAAGACTTTACTTTTCACCCACTCCACCATTTCTC :  700
NM_104069 : GTCTTGTGAAAACAGGAGAAACTACACCGTTTATTTCTTCATTGGAACTTCGTCCTTTGAACAATAATACATACGTCACAAAAAGTGGATCGCTTATTGTGGTCGCAAGACTTTACTTTTCACCCACTCCACCATTTCTC :  606
FJ708655  : GTCTTGTGAAAACAGGAGAAACTACACCGTTTATTTCTTCATTGGAACTTCGTCCTTTGAACAATAATACATACGTCACAAAAAGTGGATCGCTTATTGTGGTCGCAAGACTTTACTTTTCACCCACTCCACCATTTCTC :  606
                                                                                                                                                               
                     *       720         *       740         *       760         *       780         *       800         *       820         *       840       
Genomic   : AGGTACAGAAATGAGCCAAAAAGGTTTTATTTTCATACTAGTTGTTTTGTTGTTTGTGTTAAATTATCAAAATGAGCCTTTCTCTCTAGGTATGATGAGGACGTCCATGACCGAATTTGGATTCCATTCTTAGATAACAA :  840
NM_104069 : AGGTA---------------------------------------------------------------------------------------TGATGAGGACGTCCATGACCGAATTTGGATTCCATTCTTAGATAACAA :  659
FJ708655  : AGGTA---------------------------------------------------------------------------------------TGATGAGGACGTCCATGACCGAATTTGGATTCCATTCTTAGATAACAA :  659
                                                                                                                                                               
                     *       860         *       880         *       900         *       920         *       940         *       960         *       980       
Genomic   : AAATTCCTTGTTAAGCACGGAACTCTCTGTTGATACAAGTAACTTCTACAATGTGCCTCAAACTGTAGCGAAAACTGCTGCTGTCCCTTTAAATGCTACTCAGCCTCTGAAAATAAATTGGAGTCTCGACGACATCACTT :  980
NM_104069 : AAATTCCTTGTTAAGCACGGAACTCTCTGTTGATACAAGTAACTTCTACAATGTGCCTCAAACTGTAGCGAAAACTGCTGCTGTCCCTTTAAATGCTACTCAGCCTCTGAAAATAAATTGGAGTCTCGACGACATCACTT :  799
FJ708655  : AAATTCCTTGTTAAGCACGGAACTCTCTGTTGATACAAGTAACTTCTACAATGTGCCTCAAACTGTAGCGAAAACTGCTGCTGTCCCTTTAAATGCTACTCAGCCTCTGAAAATAAATTGGAGTCTCGACGACATCACTT :  799
                                                                                                                                                               
                     *      1000         *      1020         *      1040         *      1060         *      1080         *      1100         *      1120       
Genomic   : CACAGTCATATATATACATGCATTTCGCTGAAATCGAGAATCTTGAAGCTAATGAGACCAGAGAATTCAATATTACTTACAATGGTGGCGAAAATTGGTTCTCCTATTTTAGACCTCCAAAGTTTCGTATAACAACTGTA : 1120
NM_104069 : CACAGTCATATATATACATGCATTTCGCTGAAATCGAGAATCTTGAAGCTAATGAGACCAGAGAATTCAATATTACTTACAATGGTGGCGAAAATTGGTTCTCCTATTTTAGACCTCCAAAGTTTCGTATAACAACTGTA :  939
FJ708655  : CACAGTCATATATATACATGCATTTCGCTGAAATCGAGAATCTTGAAGCTAATGAGACCAGAGAATTCAATATTACTTACAATGGTGGCGAAAATTGGTTCTCCTATTTTAGACCTCCAAAGTTTCGTATAACAACTGTA :  939
                                                                                                                                                               
                     *      1140         *      1160         *      1180         *      1200         *      1220         *      1240         *      1260       
Genomic   : TACAATCCAGCAGCTGTGAGTTCTCTAGATGGGAATTTCAACTTCACTTTCTCGATGACCGGTAACTCTACTCATCCTCCTCTTATCAACGGCCTTGAGATTTATCAAGTTCTAGAGCTTCCACAGCTTGATACATACCA : 1260
NM_104069 : TACAATCCAGCAGCTGTGAGTTCTCTAGATGGGAATTTCAACTTCACTTTCTCGATGACCGGTAACTCTACTCATCCTCCTCTTATCAACGGCCTTGAGATTTATCAAGTTCTAGAGCTTCCACAGCTTGATACATACCA : 1079
FJ708655  : TACAATCCAGCAGCTGTGAGTTCTCTAGATGGGAATTTCAACTTCACTTTCTCGATGACCGGTAACTCTACTCATCCTCCTCTTATCAACGGCCTTGAGATTTATCAAGTTCTAGAGCTTCCACAGCTTGATACATACCA : 1079
                                                                                                                                                               
                     *      1280         *      1300         *      1320         *      1340         *      1360         *      1380         *      1400       
Genomic   : AGATGAAGGTAAGTTAAGCATGTTCTTAAACCAATTCTTTTACCAAGAGTTCATGTCTTTGAAGTTTTTGAAATGTAATGATTGATCAGTTTCCGCTATGATGAATATCAAGACAATATATGGATTGAGCAAAAGGTCTA : 1400
NM_104069 : AGATGAAG---------------------------------------------------------------------------------TTTCCGCTATGATGAATATCAAGACAATATATGGATTGAGCAAAAGGTCTA : 1138
FJ708655  : AGATGAAG---------------------------------------------------------------------------------TTTCCGCTATGATGAATATCAAGACAATATATGGATTGAGCAAAAGGTCTA : 1138
                                                                                                                                                               
                     *      1420         *      1440         *      1460         *      1480         *      1500         *      1520         *      1540       
Genomic   : GCTGGCAAGGAGATCCATGTGCTCCTGAGTTATATAGATGGGAAGGTTTAAACTGTAGTTATCCAAACTTTGCGCCACCGCAGATCATATCCTTGTATGTTTTGCTTGTTAATCTCACAATTCTTTAGTTTCGAGTTTTT : 1540
NM_104069 : GCTGGCAAGGAGATCCATGTGCTCCTGAGTTATATAGATGGGAAG----------------------------------------------------------------------------------------------- : 1183
FJ708655  : GCTGGCAAGGAGATCCATGTGCTCCTGAGTTATATAGATGGGAAGGTTTAAACTGTAGTTATCCAAACTTTGCGCCACCGCAGATCATATCCTT---------------------------------------------- : 1232
                                                                                                                                                               
                     *      1560         *      1580         *      1600         *      1620         *      1640         *      1660         *      1680       
Genomic   : TTTCTCAAGTTCTAACTTTTGGACTCTCTATTAGGAACTTGAGTGGAAGCAATTTGAGTGGTACCATAACATCTGATATATCCAAGCTAACACATTTGAGAGAACTGTAAGAATCCGAAACTGTCACACACTAATAAACT : 1680
NM_104069 : ---------TTCTAACTTTTGGACTCTCTATTAGGAACTTGAGTGGAAGCAATTTGAGTGGTACCATAACATCTGATATATCCAAGCTAACACATTTGAGAGAACT---------------------------------- : 1280
FJ708655  : -------------------------------------------------------------------------------------------------------------------------------------------- :    -
                                                                                                                                                               
                     *      1700         *      1720         *      1740         *      1760         *      1780         *      1800         *      1820       
Genomic   : TAAAGTATATGATATGGTGTATTGTGTATAACAAATTTATTTTTTTCCTTTAATGCGCAGAGATTTATCAAACAATGACTTATCAGGAGATATTCCATTTGTTTTTTCTGATATGAAGAATTTGACACTCATGTGAGCTA : 1820
NM_104069 : ------------------------------------------------------------AGATTTATCAAACAATGACTTATCAGGAGATATTCCATTTGTTTTTTCTGATATGAAGAATTTGACACTCAT-------- : 1352
FJ708655  : -------------------------------------------------------------------------------------------------------------------------------------------- :    -
                                                                                                                                                               
                     *      1840         *      1860         *      1880         *      1900         *      1920         *      1940         *      1960       
Genomic   : CTAAATGACTAATATATTTATCTTGGTTCTTGGTCGCTCTGCTCTTTGTGTGCTGTTCTCAAAGATTCATTTTTGTCATTTGCAGAAACTTGAGTGGAAACAAGAATCTAAATCGCTCAGTTCCAGAGACTCTTCAGAAG : 1960
NM_104069 : -------------------------------------------------------------------------------------AAACTTGAGTGGAAACAAGAATCTAAATCGCTCAGTTCCAGAGACTCTTCAGAAG : 1407
FJ708655  : -------------------------------------------------------------------------------------AAACTTGAGTGGAAACAAGAATCTAAATCGCTCAGTTCCAGAGACTCTTCAGAAG : 1287
                                                                                                                                                               
                     *      1980         *      2000         *      2020         *      2040         *      2060         *      2080         *      2100       
Genomic   : AGGATAGATAACAAATCTTTAACACTAATGTAAGATTTTCAGTGGATTCAGTCTCAAGCTTTTCAAGTCACGGAAAGTATTTCAGAATCCTATGAAACAGTGTCTAACCTTCTATTCGATTCCACATCACCTTTAATGAT : 2100
NM_104069 : AGGATAGATAACAAATCTTTAACACTAAT--------------------------------------------------------------------------------------------------------------- : 1436
FJ708655  : AGGATAGATAACAAATCTTTAACACTAAT--------------------------------------------------------------------------------------------------------------- : 1316
                                                                                                                                                               
                     *      2120         *      2140         *      2160         *      2180         *      2200         *      2220         *      2240       
Genomic   : TGCAGTAGAGATGAAACCGGAAAAAATAGTACAAATGTAGTTGCTATCGCAGCATCAGTGGCTAGCGTGTTTGCTGTGCTAGTTATCTTGGCTATCGTTTTTGTCGTCATAAGGAAAAAACAGAGAACTAATGAAGGTAT : 2240
NM_104069 : -----TAGAGATGAAACCGGAAAAAATAGTACAAATGTAGTTGCTATCGCAGCATCAGTGGCTAGCGTGTTTGCTGTGCTAGTTATCTTGGCTATCGTTTTTGTCGTCATAAGGAAAAAACAGAGAACTAATGAAG---- : 1567
FJ708655  : -----TAGAGATGAAACCGGAAAAAATAGTACAAATGTAGTTGCTATCGCAGCATCAGTGGCTAGCGTGTTTGCTGTGCTAGTTATCTTGGCTATCGTTTTTGTCGTCATAAGGAAAAAACAGAGAACTAATGAAG---- : 1447
                                                                                                                                                               
                     *      2260         *      2280         *      2300         *      2320         *      2340         *      2360         *      2380       
Genomic   : GATCACTAGAATAGGTCTTGAAAATAATGAAAATGAAAGTTGTCAGATTTATACTCACTTCCTTTAGGTTTTACAGCTTCAGGACCCCGATCATTCACTACTGGCACGGTTAAGAGTGATGCAAGATCATCGAGTTCATC : 2380
NM_104069 : ----------------------------------------------------------------------------------GACCCCGATCATTCACTACTGGCACGGTTAAGAGTGATGCAAGATCATCGAGTTCATC : 1625
FJ708655  : ----------------------------------------------------------------------------CTTCAGGACCCCGATCATTCACTACTGGCACGGTTAAGAGTGATGCAAGATCATCGAGTTCATC : 1511
                                                                                                                                                               
   
                     *      2400         *      2420         *      2440         *      2460         *      2480         *      2500         *      2520       
Genomic   : AATCATAACAAAGGAACGCAAGTTCACTTATTCGGAGGTACTAAAGATGACTAAAAACTTTGAGAGAGTTCTTGGTAAAGGAGGGTTTGGAACAGTGTATCATGGTAACTTGGATGATACTCAAGTAGCTGTGAAAATGC : 2520
NM_104069 : AATCATAACAAAGGAACGCAAGTTCACTTATTCGGAGGTACTAAAGATGACTAAAAACTTTGAGAGAGTTCTTGGTAAAGGAGGGTTTGGAACAGTGTATCATGGTAACTTGGATGATACTCAAGTAGCTGTGAAAATGC : 1765
FJ708655  : AATCATAACAAAGGAACGCAAGTTCACTTATTCGGAGGTACTAAAGATGACTAAAAACTTTGAGAGAGTTCTTGGTAAAGGAGGGTTTGGAACAGTGTATCATGGTAACTTGGATGATACTCAAGTAGCTGTGAAAATGC : 1651
                                                                                                                                                               
                     *      2540         *      2560         *      2580         *      2600         *      2620         *      2640         *      2660       
Genomic   : TTTCTCATTCATCAGCTCAAGGTTATAAAGAGTTCAAAGCAGAGGTACTTTAACAAGAAATGGATATGTTTTTTTTTTTTTTAATAAGAAATGGATATGTTAGTCTTAATTGTTTTTAAAGTGCATTAGGTTCCTCAATC : 2660
NM_104069 : TTTCTCATTCATCAGCTCAAGGTTATAAAGAGTTCAAAGCAGAGGT---------------------------------------------------------------------------------------------- : 1811
FJ708655  : TTTCTCATTCATCAGCTCAAGGTTATAAAGAGTTCAAAGCAGAGGT---------------------------------------------------------------------------------------------- : 1697
                                                                                                                                                               
                     *      2680         *      2700         *      2720         *      2740         *      2760         *      2780         *      2800       
Genomic   : ATGAATGGTGATTTTGTTCTTTCTTAGGTTGAACTTCTTTTAAGAGTTCATCACAGACATTTGGTGGGACTTGTTGGTTACTGTGATGATGGAGACAACTTAGCTCTGATCTATGAATATATGGAAAAAGGAGACCTGAG : 2800
NM_104069 : -----------------------------TGAACTTCTTTTAAGAGTTCATCACAGACATTTGGTGGGACTTGTTGGTTACTGTGATGATGGAGACAACTTAGCTCTGATCTATGAATATATGGAAAAAGGAGACCTGAG : 1922
FJ708655  : -----------------------------TGAACTTCTTTTAAGAGTTCATCACAGACATTTGGTGGGACTTGTTGGTTACTGTGATGATGGAGACAACTTAGCTCTGATCTATGAATATATGGAAAAAGGAGACCTGAG : 1808
                                                                                                                                                               
                     *      2820         *      2840         *      2860         *      2880         *      2900         *      2920         *      2940       
Genomic   : GGAAAATATGTCAGGTAAGCACTTTAATTTAGTTGAATCCATCTCCTTAGACAGTTTGAAACATTCAAAGCCTGGTACAGGAAAACACAGTGTCAATGTCCTAAGCTGGGAAACAAGAATGCAAATAGCTGTAGAGGCAG : 2940
NM_104069 : GGAAAATATGTCAGGTAAGCACTTTAATTTAGTTGAATCCATCTCCTTAGACAGTTTGAAACATTCAAAGCCTGGTACAGGAAAACACAGTGTCAATGTCCTAAGCTGGGAAACAAGAATGCAAATAGCTGTAGAGGCAG : 2062
FJ708655  : GGAAAATATGTCAGG------------------------------------------------------------------AAAACACAGTGTCAATGTCCTAAGCTGGGAAACAAGAATGCAAATAGCTGTAGAGGCAG : 1882
                                                                                                                                                               
                     *      2960         *      2980         *      3000         *      3020         *      3040         *      3060         *      3080       
Genomic   : CACAAGGTGAACTAAATTATGACTTATATACTTTTCATTCACAACAATTTGTGTGGTAGTCTGAAAGCAAGATTGACATAAACAATAACTATTTGTAACAGGATTGGAGTATTTGCATAACGGATGTAGGCCTCCTATGG : 3080
NM_104069 : CACAAGG-----------------------------------------------------------------------------------------------ATTGGAGTATTTGCATAACGGATGTAGGCCTCCTATGG : 2107
FJ708655  : CACAAGG-----------------------------------------------------------------------------------------------ATTGGAGTATTTGCATAACGGATGTAGGCCTCCTATGG : 1927
                                                                                                                                                               
                     *      3100         *      3120         *      3140         *      3160         *      3180         *      3200         *      3220       
Genomic   : TACATAGAGATGTGAAACCAACCAACATTTTATTAAATGAGCGGTCTCAAGCAAAACTAGCCGACTTTGGGCTATCGAGATCTTTTCCTGTTGATGGTGAATCTCATGTCATGACAGTCGTTGCAGGAACACCTGGTTAC : 3220
NM_104069 : TACATAGAGATGTGAAACCAACCAACATTTTATTAAATGAGCGGTCTCAAGCAAAACTAGCCGACTTTGGGCTATCGAGATCTTTTCCTGTTGATGGTGAATCTCATGTCATGACAGTCGTTGCAGGAACACCTGGTTAC : 2247
FJ708655  : TACATAGAGATGTGAAACCAACCAACATTTTATTAAATGAGCGGTCTCAAGCAAAACTAGCCGACTTTGGGCTATCGAGATCTTTTCCTGTTGATGGTGAATCTCATGTCATGACAGTCGTTGCAGGAACACCTGGTTAC : 2067
                                                                                                                                                               
                     *      3240         *      3260         *      3280         *      3300         *      3320         *      3340         *      3360       
Genomic   : TTAGATCCTGAGTGAGTGAATCAATCTGTTTCTTGTACTGAAATAAGTTATGAAGAATTGGGAACTGTAACCTCTCTTTATGATACTCTTCTTTTCAGGTATTACAGAACAAACTGGCTAAGCGAGAAGAGTGATGTGTA : 3360
NM_104069 : TTAGATCCTGAGT---------------------------------------------------------------------------------------ATTACAGAACAAACTGGCTAAGCGAGAAGAGTGATGTGTA : 2300
FJ708655  : TTAGATCCTGAGT---------------------------------------------------------------------------------------ATTACAGAACAAACTGGCTAAGCGAGAAGAGTGATGTGTA : 2120
                                                                                                                                                               
                     *      3380         *      3400         *      3420         *      3440         *      3460         *      3480         *      3500       
Genomic   : CAGCTTTGGTGTAGTGCTTTTAGAGATAGTCACAAACCAGCCTGTGATGAATAAAAACCGAGAGAGACCTCATATCAATGAATGGGTTATGTTCATGCTTACCAATGGGGATATCAAGAGTATTGTCGACCCGAAACTGA : 3500
NM_104069 : CAGCTTTGGTGTAGTGCTTTTAGAGATAGTCACAAACCAGCCTGTGATGAATAAAAACCGAGAGAGACCTCATATCAATGAATGGGTTATGTTCATGCTTACCAATGGGGATATCAAGAGTATTGTCGACCCGAAACTGA : 2440
FJ708655  : CAGCTTTGGTGTAGTGCTTTTAGAGATAGTCACAAACCAGCCTGTGATGAATAAAAACCGAGAGAGACCTCATATCAATGAATGGGTTATGTTCATGCTTACCAATGGGGATATCAAGAGTATTGTCGACCCGAAACTGA : 2260
                                                                                                                                                               
                     *      3520         *      3540         *      3560         *      3580         *      3600         *      3620         *      3640       
Genomic   : ATGAAGACTATGACACAAACGGTGTGTGGAAGGTTGTAGAGTTGGCTTTAGCTTGTGTAAACCCGTCTTCAAGCCGTAGACCGACAATGCCACACGTGGTGATGGAGCTAAACGAATGTCTTGCTTTGGAAATAGAAAGG : 3640
NM_104069 : ATGAAGACTATGACACAAACGGTGTGTGGAAGGTTGTAGAGTTGGCTTTAGCTTGTGTAAACCCGTCTTCAAGCCGTAGACCGACAATGCCACACGTGGTGATGGAGCTAAACGAATGTCTTGCTTTGGAAATAGAAAGG : 2580
FJ708655  : ATGAAGACTATGACACAAACGGTGTGTGGAAGGTTGTAGAGTTGGCTTTAGCTTGTGTAAACCCGTCTTCAAGCCGTAGACCGACAATGCCACACGTGGTGATGGAGCTAAACGAATGTCTTGCTTTGGAAATAGAAAGG : 2400
                                                                                                       
                     *      3660         *      3680         *      3700         *      3720           
Genomic   : AAACAAGGTAGTCAAGCGACGTACATAAAGGAATCTGTTGAGTTTAGTCCATCTTCTGCTTCTGATTTTTCCCCTTTAGCTAGG : 3724
NM_104069 : AAACAAGGTAGTCAAGCGACGTACATAAAGGAATCTGTTGAGTTTAGTCCATCTTCTGCTTCTGATTTTTCCCCTTTAGCTAGG : 2664
FJ708655  : AAACAAGGTAGTCAAGCGACGTACATAAAGGAATCTGTTGAGTTTAGTCCATCTTCTGCTTCTGATTTTTCCCCTTTAGCTAGG : 2484

At1g53430
                                                                                                                                                               
                     *        20         *        40         *        60         *        80         *       100         *       120         *       140       
Genomic   : ATGGGTTTCATCTTCTCGACCGAGAAAGTTGTGTATGTTCTTCTTCTCATCTTCGTTTGCTTGGAAAATTTCGGATCAAATGCTCAACTTTTGCCTGAAGATGAAGGTTCGTTTCACTATTTTAAATCCACATCTCAACT :  140
NM_104221 : ATGGGTTTCATCTTCTCGACCGAGAAAGTTGTGTATGTTCTTCTTCTCATCTTCGTTTGCTTGGAAAATTTCGGATCAAATGCTCAACTTTTGCCTGAAGATGA------------------------------------ :  104
FJ708657  : ATGGGTTTCATCTTCTCGACCGAGAAAGTTGTGTATGTTCTTCTTCTCATCTTCGTTTGCTTGGAAAATTTCGGATCAAATGCTCAACTTTTGCCTGAAGATGA------------------------------------ :  104
                                                                                                                                                               
                     *       160         *       180         *       200         *       220         *       240         *       260         *       280       
Genomic   : AAATTTCTTCAATTTTATTTTCAACTCCTCAATTCTACTTAGTTTCGAAGCAAAATAATTTAGATGCTGCTTTACTAAAAAAGAAACTTTTAGAGAAAGAAAGTTATCCTTTAGCAAATTATCGAATTAACTGTATTTTT :  280
NM_104221 : -------------------------------------------------------------------------------------------------------------------------------------------- :    -
FJ708657  : -------------------------------------------------------------------------------------------------------------------------------------------- :    -
                                                                                                                                                               
                     *       300         *       320         *       340         *       360         *       380         *       400         *       420       
Genomic   : TGTTGTCCTTGTTCAAAAAATATATGTATATTAAATTCCACGGGAGATTTATGAAGTTGTCAAAAGGTTTTCCATTATTACCTTTTGGTATAAGCGTGATTTTTCTCTAAAAAGTCGCATTTCAAACCTAAGATGGGAAT :  420
NM_104221 : -------------------------------------------------------------------------------------------------------------------------------------------- :    -
FJ708657  : -------------------------------------------------------------------------------------------------------------------------------------------- :    -
                                                                                                                                                               
                     *       440         *       460         *       480         *       500         *       520         *       540         *       560       
Genomic   : TTTATTTGTGTGGTCATATTTGACAAATAAAAAGCTGGAAAATATTGGATTTATTCAAGTTCGAGGACCTACCTCAATAATTGAATGGTATTGGTCGTCGTAGCTGCACGATGCAAGAGTTAAAAAAATTAGGTACACAT :  560
NM_104221 : -------------------------------------------------------------------------------------------------------------------------------------------- :    -
FJ708657  : -------------------------------------------------------------------------------------------------------------------------------------------- :    -
                                                                                                                                                               
                     *       580         *       600         *       620         *       640         *       660         *       680         *       700       
Genomic   : AAATATTACTTGACTAAACGAATATACGCGTTTTCCGTTTTTTCATGTATAAGCAAGATGTTAATTTCATTTGTATCTCAAATTCTGAATGATTTATGCTATACTAGGTTAATATGTAAATGTGTGATTTACTTTAGAAT :  700
NM_104221 : -------------------------------------------------------------------------------------------------------------------------------------------- :    -
FJ708657  : -------------------------------------------------------------------------------------------------------------------------------------------- :    -
                                                                                                                                                               
                     *       720         *       740         *       760         *       780         *       800         *       820         *       840       
Genomic   : TTTGTTTTTCTAACATTTGGATAATGTATATTTTTTCTTAAGGATGTGTTAATAAACAAATAATTATATCTCAATATGGCAAGTTAGGTCAATGAGAGAGACGTTGGGATTTGAGATATTTATATTGGACTTTCCTCATT :  840
NM_104221 : -------------------------------------------------------------------------------------------------------------------------------------------- :    -
FJ708657  : -------------------------------------------------------------------------------------------------------------------------------------------- :    -
                                                                                                                                                               
                     *       860         *       880         *       900         *       920         *       940         *       960         *       980       
Genomic   : TATGCCAAAGTTTTGACTTCAAATGTTTGACCAATTTTGCACAGTTCAAACATTGCGAACGATCTTTAGAAAGCTTCAAAACCAAACAGTGAACATCGAAAGAACTTCTTGTTCGGACCAAAACTGGAACTTTGTTGTCG :  980
NM_104221 : ------------------------------------------AGTTCAAACATTGCGAACGATCTTTAGAAAGCTTCAAAACCAAACAGTGAACATCGAAAGAACTTCTTGTTCGGACCAAAACTGGAACTTTGTTGTCG :  202
FJ708657  : ------------------------------------------AGTTCAAACATTGCGAACGATCTTTAGAAAGCTTCAAAACCAAACAGTGAACATCGAAAGAACTTCTTGTTCGGACCAAAACTGGAACTTTGTTGTCG :  202
                                                                                                                                                               
                     *      1000         *      1020         *      1040         *      1060         *      1080         *      1100         *      1120       
Genomic   : AGTCAGCCTCCAACTCACCAACCAGTAACATTACCTGCGACTGTACTTTCAACGCCAGCTCAGTCTGTCGTGTCACAAACATGTAAGTAAAAGAAACTTCTCATCTCTGTGACTTTCATAACAAGAAACTGAGTTAGGGC : 1120
NM_104221 : AGTCAGCCTCCAACTCACCAACCAGTAACATTACCTGCGACTGTACTTTCAACGCCAGCTCAGTCTGTCGTGTCACAAACAT---------------------------------------------------------- :  284
FJ708657  : AGTCAGCCTCCAACTCACCAACCAGTAACATTACCTGCGACTGTACTTTCAACGCCAGCTCAGTCTGTCGTGTCACAAACAT---------------------------------------------------------- :  284
                                                                                                                                                               
                     *      1140         *      1160         *      1180         *      1200         *      1220         *      1240         *      1260       
Genomic   : TCTGAAAAAACTTTCTTTACTTCTTGATTTTCTTCAACAGACAGCTTAAAAGTTTCAGCTTGCCCGGAATATTCCCGCCTGAGTTCGGAAACCTCACGCGTCTTCGAGAGATGTATGCATTTTCGTTCTTGAGTTTCTGT : 1260
NM_104221 : ----------------------------------------ACAGCTTAAAAGTTTCAGCTTGCCCGGAATATTCCCGCCTGAGTTCGGAAACCTCACGCGTCTTCGAGAGAT---------------------------- :  356
FJ708657  : ----------------------------------------ACAGCTTAAAAGTTTCAGCTTGCCCGGAATATTCCCGCCTGAGTTCGGAAACCTCACGCGTCTTCGAGAGAT---------------------------- :  356
                                                                                                                                                               
                     *      1280         *      1300         *      1320         *      1340         *      1360         *      1380         *      1400       
Genomic   : CAGTGGATCATAGCAAAAATGTCTGAGGCTTGGAACTTAAGCAATCTCTGTTTTTGTTTTCTTACAGAGATCTTTCGCGGAACTTTCTCAATGGAACAATACCTACGACATTGTCTCAAATTCCACTTGAAATCTTGTAA : 1400
NM_104221 : -------------------------------------------------------------------AGATCTTTCGCGGAACTTTCTCAATGGAACAATACCTACGACATTGTCTCAAATTCCACTTGAAATCTTGT-- :  427
FJ708657  : -------------------------------------------------------------------AGATCTTTCGCGGAACTTTCTCAATGGAACAATACCTACGACATTGTCTCAAATTCCACTTGAAATCTTGT-- :  427
                                                                                                                                                               
                     *      1420         *      1440         *      1460         *      1480         *      1500         *      1520         *      1540       
Genomic   : CCATTCTCTGCAACACATACTTGTTTCTTGTGATGATTAAGTTTTAGTGATTCTCACGTTTTGTTTTGTTTTCTGCAACTAATAGGTCTGTAATCGGAAACCGTCTCTCAGGACCATTTCCTCCTCAACTTGGAGATATT : 1540
NM_104221 : ---------------------------------------------------------------------------------------CTGTAATCGGAAACCGTCTCTCAGGACCATTTCCTCCTCAACTTGGAGATATT :  480
FJ708657  : ---------------------------------------------------------------------------------------CTGTAATCGGAAACCGTCTCTCAGGACCATTTCCTCCTCAACTTGGAGATATT :  480
                                                                                                                                                               
                     *      1560         *      1580         *      1600         *      1620         *      1640         *      1660         *      1680       
Genomic   : ACTACACTTACTGATGTGTAATGTTTAAATATCTAATTTGTTTTTCATCAAACCAGAATTGACAAGATTCTAATATGTAAGAGACAAATTGCAGGAATTTGGAAACTAATTTATTCACAGGACCACTTCCTCGAAACCTA : 1680
NM_104221 : ACTACACTTACTGATGTG-----------------------------------------------------------------------------AATTTGGAAACTAATTTATTCACAGGACCACTTCCTCGAAACCTA :  543
FJ708657  : ACTACACTTACTGATGTG-----------------------------------------------------------------------------AATTTGGAAACTAATTTATTCACAGGACCACTTCCTCGAAACCTA :  543
                                                                                                                                                               
                     *      1700         *      1720         *      1740         *      1760         *      1780         *      1800         *      1820       
Genomic   : GGGAACTTAAGAAGCTTGAAAGAGTTGTAAGATCAGTTACTATATACTCTGTTTTGTCTTATATATTGTTGTTTCACTTTACTTAGTGATTTGGTCTATTTATATTACAGACTTCTCTCTGCAAACAACTTCACGGGTCA : 1820
NM_104221 : GGGAACTTAAGAAGCTTGAAAGAGTT------------------------------------------------------------------------------------ACTTCTCTCTGCAAACAACTTCACGGGTCA :  599
FJ708657  : GGGAACTTAAGAAGCTTGAAAGAGTT------------------------------------------------------------------------------------ACTTCTCTCTGCAAACAACTTCACGGGTCA :  599
                                                                                                                                                               
                     *      1840         *      1860         *      1880         *      1900         *      1920         *      1940         *      1960       
Genomic   : AATCCCTGAGTCCTTAAGTAATCTCAAGAATTTGACTGAATTGTAAGTAGAGATACTGTGAAGAATTCATCAATCTTTTGATTCTTTGGATTGATTCTATAAAAGCTTCATATGTTTCTTGCAGTCGGATTGATGGAAAC : 1960
NM_104221 : AATCCCTGAGTCCTTAAGTAATCTCAAGAATTTGACTGAATT----------------------------------------------------------------------------------TCGGATTGATGGAAAC :  657
FJ708657  : AATCCCTGAGTCCTTAAGTAATCTCAAGAATTTGACTGAATT----------------------------------------------------------------------------------TCGGATTGATGGAAAC :  657
                                                                                                                                                               
                     *      1980         *      2000         *      2020         *      2040         *      2060         *      2080         *      2100       
Genomic   : TCTCTATCTGGGAAAATACCTGATTTTATTGGAAACTGGACTCTGCTGGAGAGGCTGTGAGTAACCATCATTTCTCAAATAACTTCTCCTCAAAAACACTAAAAGTTGTAGTGACTAAATTGTTAATCTGCAGAGACCTT : 2100
NM_104221 : TCTCTATCTGGGAAAATACCTGATTTTATTGGAAACTGGACTCTGCTGGAGAGGCT-----------------------------------------------------------------------------AGACCTT :  720
FJ708657  : TCTCTATCTGGGAAAATACCTGATTTTATTGGAAACTGGACTCTGCTGGAGAGGCT-----------------------------------------------------------------------------AGACCTT :  720
                                                                                                                                                               
                     *      2120         *      2140         *      2160         *      2180         *      2200         *      2220         *      2240       
Genomic   : CAAGGCACATCAATGGAAGGTCCAATTCCACCTTCAATATCAAACTTGACGAACTTGACTGAATTGTAAGTATGAAACAAGTGTAACATACCATTTTCTGCATGCTCGTCCAAATTCTTGACCAAGCTCTATTTTACCAG : 2240
NM_104221 : CAAGGCACATCAATGGAAGGTCCAATTCCACCTTCAATATCAAACTTGACGAACTTGACTGAATTG-------------------------------------------------------------------------- :  786
FJ708657  : CAAGGCACATCAATGGAAGGTCCAATTCCACCTTCAATATCAAACTTGACGAACTTGACTGAATTG-------------------------------------------------------------------------- :  786
                                                                                                                                                               
                     *      2260         *      2280         *      2300         *      2320         *      2340         *      2360         *      2380       
Genomic   : GAGGATAACCGATTTGCGTGGACAAGCGGCCTTTTCTTTTCCGGACCTGCGAAATCTAATGAAAATGAAACGATTGTAAGTTTTAACATTGATTCAAGTTATATACTATGTTTCATCAAACAATATTTAACAGTCTGCTA : 2380
NM_104221 : -AGGATAACCGATTTGCGTGGACAAGCGGCCTTTTCTTTTCCGGACCTGCGAAATCTAATGAAAATGAAACGATTG---------------------------------------------------------------- :  861
FJ708657  : -AGGATAACCGATTTGCGTGGACAAGCGGCCTTTTCTTTTCCGGACCTGCGAAATCTAATGAAAATGAAACGATTG---------------------------------------------------------------- :  861
                
                                                                                                                                               
                                                                                                                                                               
                     *      2400         *      2420         *      2440         *      2460         *      2480         *      2500         *      2520       
Genomic   : ACTTCACTAGGGTACTAAGAAATTGTTTGATAAGGGGACCTATCCCGGAATACATTGGTTCCATGAGTGAGCTGAAGACACTGTAAGTCAAACTCTGCTGCTTAAAGGCTAATGGTTTGATTAAGATCTCACATTAGAGT : 2520
NM_104221 : -----------------------------------GGACCTATCCCGGAATACATTGGTTCCATGAGTGAGCTGAAGACACT---------------------------------------------------------- :  908
FJ708657  : -----------GTACTAAGAAATTGTTTGATAAGGGGACCTATCCCGGAATACATTGGTTCCATGAGTGAGCTGAAGACACT---------------------------------------------------------- :  932
                                                                                                                                                               
                     *      2540         *      2560         *      2580         *      2600         *      2620         *      2640         *      2660       
Genomic   : ATTTGTGTGTGTGTGTGCAGAGATTTAAGCTCAAACATGCTAACCGGTGTAATTCCTGACACATTTCGGAATCTCGATGCATTTAACTTTATGTAAGTTTCAAATTATCAGCATGGATGGCATTAACATGTACTCGAAAA : 2660
NM_104221 : --------------------AGATTTAAGCTCAAACATGCTAACCGGTGTAATTCCTGACACATTTCGGAATCTCGATGCATTTAACTTTATGT---------------------------------------------- :  982
FJ708657  : --------------------AGATTTAAGCTCAAACATGCTAACCGGTGTAATTCCTGACACATTTCGGAATCTCGATGCATTTAACTTTATGT---------------------------------------------- : 1006
                                                                                                                                                               
                     *      2680         *      2700         *      2720         *      2740         *      2760         *      2780         *      2800       
Genomic   : ACTGATAGTTATTTTTGTGGCTTTAGGTTTTTGAATAATAACTCATTGACTGGTCCAGTTCCTCAGTTCATCATCAACAGTAAAGAAAACCTGTAAGCAAAACTAACTAACCACCTCGAGGTTGGATATTCGGTTAACCA : 2800
NM_104221 : ----------------------------TTTTGAATAATAACTCATTGACTGGTCCAGTTCCTCAGTTCATCATCAACAGTAAAGAAAACCT------------------------------------------------ : 1046
FJ708657  : ----------------------------TTTTGAATAATAACTCATTGACTGGTCCAGTTCCTCAGTTCATCATCAACAGTAAAGAAAACCT------------------------------------------------ : 1070
                                                                                                                                                               
                     *      2820         *      2840         *      2860         *      2880         *      2900         *      2920         *      2940       
Genomic   : TCTGCGCTTATCGAGGTCATTAATTTTCTTGATTTGTTTTCAGAGATTTATCTGACAACAATTTCACTCAGCCACCTACTTTAAGCTGTAATCAGCTTGATGTGTAAGCATTCTTGTTATTTCATATTTTCCACCTGAAC : 2940
NM_104221 : -------------------------------------------AGATTTATCTGACAACAATTTCACTCAGCCACCTACTTTAAGCTGTAATCAGCTTGATGTG------------------------------------ : 1107
FJ708657  : -------------------------------------------AGATTTATCTGACAACAATTTCACTCAGCCACCTACTTTAAGCTGTAATCAGCTTGATGTG------------------------------------ : 1131
                                                                                                                                                               
                     *      2960         *      2980         *      3000         *      3020         *      3040         *      3060         *      3080       
Genomic   : CTTTTCTGTATGTTTTTCTAATTAGATTTATTGGTTAAAACATACAGGAACTTGATCTCCAGCTACCCTTCAGTAACCGATAACTCGTGAGTCTCCTTTTTTTCCCTCCTTTCGGTTCATAGAACCCAAAAAACCAAAGG : 3080
NM_104221 : ------------------------------------------------AACTTGATCTCCAGCTACCCTTCAGTAACCGATAACTC------------------------------------------------------ : 1145
FJ708657  : ------------------------------------------------AACTTGATCTCCAGCTACCCTTCAGTAACCGATAACTC------------------------------------------------------ : 1169
                                                                                                                                                               
                     *      3100         *      3120         *      3140         *      3160         *      3180         *      3200         *      3220       
Genomic   : TACTTACAAAAGAAAATATTTCTACTTGTGTATAGTGTTCAATGGTGCTTGAGAGAGGGTCTTCCATGTCCAGAAGATGCTAAACGTGAGTGTCCATCTTAAAGATTCAAACTCTTTGTGTGTGTACTATAAAGATTCAA : 3220
NM_104221 : -----------------------------------TGTTCAATGGTGCTTGAGAGAGGGTCTTCCATGTCCAGAAGATGCTAAAC------------------------------------------------------- : 1195
FJ708657  : -----------------------------------TGTTCAATGGTGCTTGAGAGAGGGTCTTCCATGTCCAGAAGATGCTAAAC------------------------------------------------------- : 1219
                                                                                                                                                               
                     *      3240         *      3260         *      3280         *      3300         *      3320         *      3340         *      3360       
Genomic   : ACTTTTCGATAAAGAATCCCTAACCAGTTACATTATTTATCCTGGGTTATCGCAGAGTCTTCCCTGTTCATCAACTGTGGAGGAAGCAGACTCAAGATTGGAAAAGACACTTATACAGATGACTTGAACAGTAGAGGACA : 3360
NM_104221 : -------------------------------------------------------AGTCTTCCCTGTTCATCAACTGTGGAGGAAGCAGACTCAAGATTGGAAAAGACACTTATACAGATGACTTGAACAGTAGAGGACA : 1280
FJ708657  : -------------------------------------------------------AGTCTTCCCTGTTCATCAACTGTGGAGGAAGCAGACTCAAGATTGGAAAAGACACTTATACAGATGACTTGAACAGTAGAGGACA : 1304
                                                                                                                                                               
                     *      3380         *      3400         *      3420         *      3440         *      3460         *      3480         *      3500       
Genomic   : ATCAACATTCTCTTCTGTCTCTGAAAGATGGGGATACAGTAGTTCTGGAGTTTGGTTAGGCAAGGAGGATGCTGGCTACTTAGCAACAGACAGATTTAACTTGATCAATGGATCAACTCCAGAGTATTACAAAACAGCCC : 3500
NM_104221 : ATCAACATTCTCTTCTGTCTCTGAAAGATGGGGATACAGTAGTTCTGGAGTTTGGTTAGGCAAGGAGGATGCTGGCTACTTAGCAACAGACAGATTTAACTTGATCAATGGATCAACTCCAGAGTATTACAAAACAGCCC : 1420
FJ708657  : ATCAACATTCTCTTCTGTCTCTGAAAGATGGGGATACAGTAGTTCTGGAGTTTGGTTAGGCAAGGAGGATGCTGGCTACTTAGCAACAGACAGATTTAACTTGATCAATGGATCAACTCCAGAGTATTACAAAACAGCCC : 1444
                                                                                                                                                               
                     *      3520         *      3540         *      3560         *      3580         *      3600         *      3620         *      3640       
Genomic   : GTCTCTCTCCACAGTCACTCAAGTACTATGGACTATGCTTGAGAAGAGGAAGTTACAAACTGCAGCTACATTTTGCAGAGATAATGTTCTCAAATGACCAGACTTTTAATAGCTTAGGGCGGCGAATATTCGACATTTAT : 3640
NM_104221 : GTCTCTCTCCACAGTCACTCAAGTACTATGGACTATGCTTGAGAAGAGGAAGTTACAAACTGCAGCTACATTTTGCAGAGATAATGTTCTCAAATGACCAGACTTTTAATAGCTTAGGGCGGCGAATATTCGACATTTAT : 1560
FJ708657  : GTCTCTCTCCACAGTCACTCAAGTACTATGGACTATGCTTGAGAAGAGGAAGTTACAAACTGCAGCTACATTTTGCAGAGATAATGTTCTCAAATGACCAGACTTTTAATAGCTTAGGGCGGCGAATATTCGACATTTAT : 1584
                                                                                                                                                               
                     *      3660         *      3680         *      3700         *      3720         *      3740         *      3760         *      3780       
Genomic   : GTCCAAGTGAGTTTTGCTGAACAAAGAATGTTTCTGTTTTCTTGGATCATTATGTTAAATTTCTCTTCTGCAGGGGAACTTGTTGGAGAGGGACTTTAACATAGCAGAGAGAGCAGGTGGAGTTGGTAAACCGTTCATAA : 3780
NM_104221 : GTCCAA-------------------------------------------------------------------GGGAACTTGTTGGAGAGGGACTTTAACATAGCAGAGAGAGCAGGTGGAGTTGGTAAACCGTTCATAA : 1633
FJ708657  : GTCCAA-------------------------------------------------------------------GGGAACTTGTTGGAGAGGGACTTTAACATAGCAGAGAGAGCAGGTGGAGTTGGTAAACCGTTCATAA : 1657
                                                                                                                                                               
                     *      3800         *      3820         *      3840         *      3860         *      3880         *      3900         *      3920       
Genomic   : GGCAAATTGATGGAGTTCAAGTGAATGGAAGTACGTTAGAGATTCATTTGCAGTGGACAGGGAAAGGCACAAACGTAATACCAACAAGAGGTGTTTACGGGCCTCTCATATCTGCCATAACCATTACACCAAGTGAGTTT : 3920
NM_104221 : GGCAAATTGATGGAGTTCAAGTGAATGGAAGTACGTTAGAGATTCATTTGCAGTGGACAGGGAAAGGCACAAACGTAATACCAACAAGAGGTGTTTACGGGCCTCTCATATCTGCCATAACCATTACACCAA-------- : 1765
FJ708657  : GGCAAATTGATGGAGTTCAAGTGAATGGAAGTACGTTAGAGATTCATTTGCAGTGGACAGGGAAAGGCACAAACGTAATACCAACAAGAGGTGTTTACGGGCCTCTCATATCTGCCATAACCATTACACCAA-------- : 1789
                                                                                                                                                               
                     *      3940         *      3960         *      3980         *      4000         *      4020         *      4040         *      4060       
Genomic   : TCTTTCATATATTTCTCAACTGTTTAAACCGATTTTGGGTGAGTAAGTGAATGTTCTCTCTGTTTCATTGCCTGTTGTTGTAGATTTCAAGGTTGATACCGGAAAACCATTGTCCAATGGAGCAGTTGCAGGCATTGTAA : 4060
NM_104221 : -----------------------------------------------------------------------------------ATTTCAAGGTTGATACCGGAAAACCATTGTCCAATGGAGCAGTTGCAGGCATTGTAA : 1822
FJ708657  : -----------------------------------------------------------------------------------ATTTCAAGGTTGATACCGGAAAACCATTGTCCAATGGAGCAGTTGCAGGCATTGTAA : 1846
                                                                                                                                                               
                     *      4080         *      4100         *      4120         *      4140         *      4160         *      4180         *      4200       
Genomic   : TCGCAGCGTGTGCGGTTTTCGGGTTGCTGGTACTTGTAATCTTGAGGCTTACAGGTTACTTAGGTGGAAAAGAAGTAGATGAGAATGGTAACAAAGAAAACATCATTCTTTTCAAGAAATGCTCTTGTCATTTTCAAGAT : 4200
NM_104221 : TCGCAGCGTGTGCGGTTTTCGGGTTGCTGGTACTTGTAATCTTGAGGCTTACAGGTTACTTAGGTGGAAAAGAAGTAGATGAGAATG----------------------------------------------------- : 1909
FJ708657  : TCGCAGCGTGTGCGGTTTTCGGGTTGCTGGTACTTGTAATCTTGAGGCTTACAGGTTACTTAGGTGGAAAAGAAGTAGATGAGAATG----------------------------------------------------- : 1933
                                                                                                                                                               
                     *      4220         *      4240         *      4260         *      4280         *      4300         *      4320         *      4340       
Genomic   : CTGATTGTTTTTTTTTCAAATCTTACTTTTTGGTTTTATCAGAAGAGCTTCGGGGACTTGATTTGCAGACAGGATCGTTCACATTGAAACAAATAAAACGTGCTACTAATAACTTTGATCCAGAAAACAAGATTGGTGAA : 4340
NM_104221 : ------------------------------------------AAGAGCTTCGGGGACTTGATTTGCAGACAGGATCGTTCACATTGAAACAAATAAAACGTGCTACTAATAACTTTGATCCAGAAAACAAGATTGGTGAA : 2007
FJ708657  : ------------------------------------------AAGAGCTTCGGGGACTTGATTTGCAGACAGGATCGTTCACATTGAAACAAATAAAACGTGCTACTAATAACTTTGATCCAGAAAACAAGATTGGTGAA : 2031
                                                                                                                                                               
                     *      4360         *      4380         *      4400         *      4420         *      4440         *      4460         *      4480       
Genomic   : GGAGGATTTGGACCGGTTTATAAGGTGATCATTGCTTTGGCTTTACACCTTCACAAGTTCTGTTAGATTGGATTATAAGTATTGATGTGCTTTGTTATTAGGGTGTTCTCGCTGATGGGATGACCATAGCGGTGAAGCAG : 4480
NM_104221 : GGAGGATTTGGACCGGTTTATAAGG-----------------------------------------------------------------------------GTGTTCTCGCTGATGGGATGACCATAGCGGTGAAGCAG : 2070
FJ708657  : GGAGGATTTGGACCGGTTTATAAGG-----------------------------------------------------------------------------GTGTTCTCGCTGATGGGATGACCATAGCGGTGAAGCAG : 2094
                                                                                                                                                               
                     *      4500         *      4520         *      4540         *      4560         *      4580         *      4600         *      4620       
Genomic   : CTTTCATCAAAATCTAAGCAAGGAAACCGAGAATTTGTGACTGAGATCGGTATGATATCTGCGCTGCAACACCCTAATCTTGTGAAACTTTATGGTTGTTGCATCGAAGGGAAAGAGCTCTTGCTTGTGTATGAGTACTT : 4620
NM_104221 : CTTTCATCAAAATCTAAGCAAGGAAACCGAGAATTTGTGACTGAGATCGGTATGATATCTGCGCTGCAACACCCTAATCTTGTGAAACTTTATGGTTGTTGCATCGAAGGGAAAGAGCTCTTGCTTGTGTATGAGTACTT : 2210
FJ708657  : CTTTCATCAAAATCTAAGCAAGGAAACCGAGAATTTGTGACTGAGATCGGTATGATATCTGCGCTGCAACACCCTAATCTTGTGAAACTTTATGGTTGTTGCATCGAAGGGAAAGAGCTCTTGCTTGTGTATGAGTACTT : 2234
                                                                                                                                                               
                     *      4640         *      4660         *      4680         *      4700         *      4720         *      4740         *      4760       
Genomic   : AGAGAACAACAGTCTCGCTCGCGCACTCTTTGGTTAGTTTTTTCTATCCATCTGGAAAAGATAGATTGAAGTGCTTGATCTTTAACCTGAAGTCACTTGTGGGTAACAACAGGCACAGAGAAACAGAGGCTTCACTTGGA : 4760
NM_104221 : AGAGAACAACAGTCTCGCTCGCGCACTCTTTGG--------------------------------------------------------------------------------CACAGAGAAACAGAGGCTTCACTTGGA : 2270
FJ708657  : AGAGAACAACAGTCTCGCTCGCGCACTCTTTGG--------------------------------------------------------------------------------CACAGAGAAACAGAGGCTTCACTTGGA : 2294


                                                                                                                                                               
                     *      4780         *      4800         *      4820         *      4840         *      4860         *      4880         *      4900       
Genomic   : TTGGTCAACAAGGAACAAGATTTGCATAGGGATTGCGAAAGGATTGGCCTATCTACACGAGGAATCAAGGCTGAAGATTGTTCATAGAGACATAAAAGCGACAAATGTGCTTCTTGATCTGTCTCTAAACGCTAAGATCT : 4900
NM_104221 : TTGGTCAACAAGGAACAAGATTTGCATAGGGATTGCGAAAGGATTGGCCTATCTACACGAGGAATCAAGGCTGAAGATTGTTCATAGAGACATAAAAGCGACAAATGTGCTTCTTGATCTGTCTCTAAACGCTAAGATCT : 2410
FJ708657  : TTGGTCAACAAGGAACAAGATTTGCATAGGGATTGCGAAAGGATTGGCCTATCTACACGAGGAATCAAGGCTGAAGATTGTTCATAGAGACATAAAAGCGACAAATGTGCTTCTTGATCTGTCTCTAAACGCTAAGATCT : 2434
                                                                                                                                                               
                     *      4920         *      4940         *      4960         *      4980         *      5000         *      5020         *      5040       
Genomic   : CTGATTTTGGTCTAGCTAAACTCAACGATGACGAGAATACACATATCAGCACAAGGATTGCAGGAACAATGTGAGTGTTCCTTACTTATCTTTTCTAAAATTCTTAGCGAGCGGTTAACACTTATCGACTTGATAAATCA : 5040
NM_104221 : CTGATTTTGGTCTAGCTAAACTCAACGATGACGAGAATACACATATCAGCACAAGGATTGCAGGAACAAT---------------------------------------------------------------------- : 2480
FJ708657  : CTGATTTTGGTCTAGCTAAACTCAACGATGACGAGAATACACATATCAGCACAAGGATTGCAGGAACAAT---------------------------------------------------------------------- : 2504
                                                                                                                                                               
                     *      5060         *      5080         *      5100         *      5120         *      5140         *      5160         *      5180       
Genomic   : TTGATGCAGAGGTTACATGGCTCCTGAGTATGCAATGAGAGGTTACTTGACAGACAAGGCAGATGTTTACAGCTTTGGTGTTGTCTGTTTAGAGATTGTTAGTGGAAAGAGCAACACAAATTACAGACCAAAGGAAGAGT : 5180
NM_104221 : ---------AGGTTACATGGCTCCTGAGTATGCAATGAGAGGTTACTTGACAGACAAGGCAGATGTTTACAGCTTTGGTGTTGTCTGTTTAGAGATTGTTAGTGGAAAGAGCAACACAAATTACAGACCAAAGGAAGAGT : 2611
FJ708657  : ---------AGGTTACATGGCTCCTGAGTATGCAATGAGAGGTTACTTGACAGACAAGGCAGATGTTTACAGCTTTGGTGTTGTCTGTTTAGAGATTGTTAGTGGAAAGAGCAACACAAATTACAGACCAAAGGAAGAGT : 2635
                                                                                                                                                               
                     *      5200         *      5220         *      5240         *      5260         *      5280         *      5300         *      5320       
Genomic   : TTGTTTACCTTCTGGATTGGGCATATGTCTTGCAAGAACAAGGTAGTCTTCTAGAACTCGTTGATCCGGATCTCGGCACAAGCTTTTCGAAGAAAGAAGCGATGAGGATGTTGAACATAGCATTACTCTGCACGAACCCA : 5320
NM_104221 : TTGTTTACCTTCTGGATTGGGCATATGTCTTGCAAGAACAAGGTAGTCTTCTAGAACTCGTTGATCCGGATCTCGGCACAAGCTTTTCGAAGAAAGAAGCGATGAGGATGTTGAACATAGCATTACTCTGCACGAACCCA : 2751
FJ708657  : TTGTTTACCTTCTGGATTGGGCATATGTCTTGCAAGAACAAGGTAGTCTTCTAGAACTCGTTGATCCGGATCTCGGCACAAGCTTTTCGAAGAAAGAAGCGATGAGGATGTTGAACATAGCATTACTCTGCACGAACCCA : 2775
                                                                                                                                                               
                     *      5340         *      5360         *      5380         *      5400         *      5420         *      5440         *      5460       
Genomic   : TCTCCAACATTGAGACCACCAATGTCATCTGTTGTAAGTATGTTAGAAGGAAAAATCAAAGTCCAACCACCATTGGTGAAACGTGAAGCAGATCCAAGTGGTTCAGCAGCAATGAGGTTTAAGGCCTTAGAGCTTTTGTC : 5460
NM_104221 : TCTCCAACATTGAGACCACCAATGTCATCTGTTGTAAGTATGTTAGAAGGAAAAATCAAAGTCCAACCACCATTGGTGAAACGTGAAGCAGATCCAAGTGGTTCAGCAGCAATGAGGTTTAAGGCCTTAGAGCTTTTGTC : 2891
FJ708657  : TCTCCAACATTGAGACCACCAATGTCATCTGTTGTAAGTATGTTAGAAGGAAAAATCAAAGTCCAACCACCATTGGTGAAACGTGAAGCAGATCCAAGTGGTTCAGCAGCAATGAGGTTTAAGGCCTTAGAGCTTTTGTC : 2915
                                                                                                                                                               
                     *      5480         *      5500         *      5520         *      5540         *      5560         *      5580         *      5600       
Genomic   : ACAAGACAGTGAGTCACAAGTCTCGACATATGCAAGAAACAGAGAGCAAGACATAAGCTCCTCATCGATGGACGGTCCTTGGGTAGATTCTTCCTTCTCTGAGCCTGGGAAAGATGTTAGCCTGCAACAGCAAGAAGAAG : 5600
NM_104221 : ACAAGACAGTGAGTCACAAGTCTCGACATATGCAAGAAACAGAGAGCAAGACATAAGCTCCTCATCGATGGACGGTCCTTGGGTAGATTCTTCCTTCTCTGAGCCTGGGAAAGATGTTAGCCTGCAACAGCAAGAAGAAG : 3031
FJ708657  : ACAAGACAGTGAGTCACAAGTCTCGACATATGCAAGAAACAGAGAGCAAGACATAAGCTCCTCATCGATGGACGGTCCTTGGGTAGATTCTTCCTTCTCTGAGCCTGGGAAAGATGTTAGCCTGCAACAGCAAGAAGAAG : 3055
                                                                              
                     *      5620         *      5640         *                
Genomic   : GACGTTCATCATCTTCGTCGAGGAAACTTTTAGATGATCTTACCGATGTGAAGATTGAG : 5659
NM_104221 : GACGTTCATCATCTTCGTCGAGGAAACTTTTAGATGATCTTACCGATGTGAAGATTGAG : 3090
FJ708657  : GACGTTCATCATCTTCGTCGAGGAAACTTTTAGATGATCTTACCGATGTGAAGATTGAG : 3114


At2g02780
                                                                                                                                                               
                     *        20         *        40         *        60         *        80         *       100         *       120         *       140       
Genomic   : ATGCAGATTTCTCTTCAAATCCATTTATCTTCCTTCACTTTTCTTCTTCTTATTTTCCTTCTTCCAGTACTCTCTGAGTCTCAGGTAGCTTCAAGTGAATCACAAACTCTTCTTGAAATCCAGAAGCAACTACAATATCC :  140
NM_126333 : ATGCAGATTTCTCTTCAAATCCATTTATCTTCCTTCACTTTTCTTCTTCTTATTTTCCTTCTTCCAGTACTCTCTGAGTCTCAGGTAGCTTCAAGTGAATCACAAACTCTTCTTGAAATCCAGAAGCAACTACAATATCC :  140
FJ708689  : ATGCAGATTTCTCTTCAAATCCATTTATCTTCCTTCACTTTTCTTCTTCTTATTTTCCTTCTTCCAGTACTCTCTGAGTCTCAGGTAGCTTCAAGTGAATCACAAACTCTTCTTGAAATCCAGAAGCAACTACAATATCC :  140
                                                                                                                                                               
                     *       160         *       180         *       200         *       220         *       240         *       260         *       280       
Genomic   : ACAGGTTCTTCAATCATGGACTGATACAACCAACTTCTGTCACATTCGTCCGTCTCCTTCCTTGAGAATCATCTGCCTCCATGGTCACGTAACAGAGTTAACCGTCACCGGGAACAGAACTAGTAAGCTCTCTGGAAGTT :  280
NM_126333 : ACAGGTTCTTCAATCATGGACTGATACAACCAACTTCTGTCACATTCGTCCGTCTCCTTCCTTGAGAATCATCTGCCTCCATGGTCACGTAACAGAGTTAACCGTCACCGGGAACAGAACTAGTAAGCTCTCTGGAAGTT :  280
FJ708689  : ACAGGTTCTTCAATCATGGACTGATACAACCAACTTCTGTCACATTCGTCCGTCTCCTTCCTTGAGAATCATCTGCCTCCATGGTCACGTAACAGAGTTAACCGTCACCGGGAACAGAACTAGTAAGCTCTCTGGAAGTT :  280
                                                                                                                                                               
                     *       300         *       320         *       340         *       360         *       380         *       400         *       420       
Genomic   : TTCATAAACTCTTCACTCTTCTTACACAACTATCGAGCTTAAAGACTTTGTCTCTTACTTCTCTCGGAATTTCTGGTTCTCTTTCTCCTAAGATCATCACTAAGCTATCACCGTCTCTTGAGTCTCTCAATCTTAGCTCT :  420
NM_126333 : TTCATAAACTCTTCACTCTTCTTACACAACTATCGAGCTTAAAGACTTTGTCTCTTACTTCTCTCGGAATTTCTGGTTCTCTTTCTCCTAAGATCATCACTAAGCTATCACCGTCTCTTGAGTCTCTCAATCTTAGCTCT :  420
FJ708689  : TTCATAAACTCTTCACTCTTCTTACACAACTATCGAGCTTAAAGACTTTGTCTCTTACTTCTCTCGGAATTTCTGGTTCTCTTTCTCCTAAGATCATCACTAAGCTATCACCGTCTCTTGAGTCTCTCAATCTTAGCTCT :  420
                                                                                                                                                               
                     *       440         *       460         *       480         *       500         *       520         *       540         *       560       
Genomic   : AATTTCATCTCTGGGAAGATTCCAGAAGAGATTGTGTCTTTGAAGAATCTGAAAAGCCTTGTTTTAAGAGACAATATGTTCTGGGGTTTTGTCTCTGATGATCTCAGAGGATTATCAAATCTTCAAGAGCTGGATTTGGG :  560
NM_126333 : AATTTCATCTCTGGGAAGATTCCAGAAGAGATTGTGTCTTTGAAGAATCTGAAAAGCCTTGTTTTAAGAGACAATATGTTCTGGGGTTTTGTCTCTGATGATCTCAGAGGATTATCAAATCTTCAAGAGCTGGATTTGGG :  560
FJ708689  : AATTTCATCTCTGGGAAGATTCCAGAAGAGATTGTGTCTTTGAAGAATCTGAAAAGCCTTGTTTTAAGAGACAATATGTTCTGGGGTTTTGTCTCTGATGATCTCAGAGGATTATCAAATCTTCAAGAGCTGGATTTGGG :  560
                                                                                                                                                               
                     *       580         *       600         *       620         *       640         *       660         *       680         *       700       
Genomic   : AGGTAATAAACTCGGTCCTGAAGTTCCTTCACTCCCAAGTAAGCTCACCACTGTTTCATTGAAGAACAACTCCTTTAGATCCAAGATTCCAGAACAGATCAAGAAGCTGAATAACCTTCAAAGTTTAGACCTTTCTTCCA :  700
NM_126333 : AGGTAATAAACTCGGTCCTGAAGTTCCTTCACTCCCAAGTAAGCTCACCACTGTTTCATTGAAGAACAACTCCTTTAGATCCAAGATTCCAGAACAGATCAAGAAGCTGAATAACCTTCAAAGTTTAGACCTTTCTTCCA :  700
FJ708689  : AGGTAATAAACTCGGTCCTGAAGTTCCTTCACTCCCAAGTAAGCTCACCACTGTTTCATTGAAGAACAACTCCTTTAGATCCAAGATTCCAGAACAGATCAAGAAGCTGAATAACCTTCAAAGTTTAGACCTTTCTTCCA :  700
                                                                                                                                                               
                     *       720         *       740         *       760         *       780         *       800         *       820         *       840       
Genomic   : ACGAATTCACCGGGTCGATTCCAGAGTTTCTGTTTTCGATTCCTTCTCTTCAAATTCTCAGTTTGGATCAGAATCTGTTAAGCGGGTCACTTCCAAATTCTTCTTGCACCTCCTCAAAGATTATAACTTTAGACGTGTCT :  840
NM_126333 : ACGAATTCACCGGGTCGATTCCAGAGTTTCTGTTTTCGATTCCTTCTCTTCAAATTCTCAGTTTGGATCAGAATCTGTTAAGCGGGTCACTTCCAAATTCTTCTTGCACCTCCTCAAAGATTATAACTTTAGACGTGTCT :  840
FJ708689  : ACGAATTCACCGGGTCGATTCCAGAGTTTCTGTTTTCGATTCCTTCTCTTCAAATTCTCAGTTTGGATCAGAATCTGTTAAGCGGGTCACTTCCAAATTCTTCTTGCACCTCCTCAAAGATTATAACTTTAGACGTGTCT :  840
                                                                                                                                                               
                     *       860         *       880         *       900         *       920         *       940         *       960         *       980       
Genomic   : CACAATCTTTTAACTGGAAAGCTTCCTTCTTGCTACTCTTCAAAGAGTTTCAGTAATCAGACAGTGCTTTTCTCATTCAACTGCTTGTCTTTGATTGGCACTCCTAACGCGAAGTATCAGCGTCCGCTTTCTTTCTGTCA :  980
NM_126333 : CACAATCTTTTAACTGGAAAGCTTCCTTCTTGCTACTCTTCAAAGAGTTTCAGTAATCAGACAGTGCTTTTCTCATTCAACTGCTTGTCTTTGATTGGCACTCCTAACGCGAAGTATCAGCGTCCGCTTTCTTTCTGTCA :  980
FJ708689  : CACAATCTTTTAACTGGAAAGCTTCCTTCTTGCTACTCTTCAAAGAGTTTCAGTAATCAGACAGTGCTTTTCTCATTCAACTGCTTGTCTTTGATTGGCACTCCTAACGCGAAGTATCAGCGTCCGCTTTCTTTCTGTCA :  980
                                                                                                                                                               
                     *      1000         *      1020         *      1040         *      1060         *      1080         *      1100         *      1120       
Genomic   : AAACCAAGCAAGCAAGGCAATAGCTGTGGAACCTATCCCCAAGGCTAAAGATAAAGATTCTGCAAGAATCAAACTCGGGTTAGTGATTTTGATAATCATCGGTGTGATCATCCTTGCAGCAATTTTGGTTCTGTTGGTTT : 1120
NM_126333 : AAACCAAGCAAGCAAGGCAATAGCTGTGGAACCTATCCCCAAGGCTAAAGATAAAGATTCTGCAAGAATCAAACTCGGGTTAGTGATTTTGATAATCATCGGTGTGATCATCCTTGCAGCAATTTTGGTTCTGTTGGTTT : 1120
FJ708689  : AAACCAAGCAAGCAAGGCAATAGCTGTGGAACCTATCCCCAAGGCTAAAGATAAAGATTCTGCAAGAATCAAACTCGGGTTAGTGATTTTGATAATCATCGGTGTGATCATCCTTGCAGCAATTTTGGTTCTGTTGGTTT : 1120
                                                                                                                                                               
                     *      1140         *      1160         *      1180         *      1200         *      1220         *      1240         *      1260       
Genomic   : TGATTGCCCTCAAAAGAAGAAGATCAAGATCAGAAGATGATCCTTTTGAAGTGAACAACAGCAACAATGAGAGACATGCCTCTGATAAAGTCTCTGTTTGCAGCACCACAACTGCCAGCTCTAAGTCATTACCAGATTCA : 1260
NM_126333 : TGATTGCCCTCAAAAGAAGAAGATCAAGATCAGAAGATGATCCTTTTGAAGTGAACAACAGCAACAATGAGAGACATGCCTCTGATAAAGTCTCTGTTTGCAGCACCACAACTGCCAGCTCTAAGTCATTACCAGATTCA : 1260
FJ708689  : TGATTGCCCTCAAAAGAAGAAGATCAAGATCAGAAGATGATCCTTTTGAAGTGAACAACAGCAACAATGAGAGACATGCCTCTGATAAAGTCTCTGTTTGCAGCACCACAACTGCCAGCTCTAAGTCATTACCAGATTCA : 1260
                                                                                                                                                               
                     *      1280         *      1300         *      1320         *      1340         *      1360         *      1380         *      1400       
Genomic   : AGTAAACAATGACTTATCTTATTAATACTTGGACACTTGAAACTGAGTCTTTCTCTAGCAAAAACCATTAACAGGATCTTTCTGAGTGTAGGACGTGTACCACAGACAATGAGATCTGCAGTGATTGGTTTGCCACCGTA : 1400
NM_126333 : A---------------------------------------------------------CAAAAACCATTAACAGGATCTTTCTGAGTGTAGGACGTGTACCACAGACAATGAGATCTGCAGTGATTGGTTTGCCACCGTA : 1343
FJ708689  : A------------------------------------------------------------------------------------------GACGTGTACCACAGACAATGAGATCTGCAGTGATTGGTTTGCCACCGTA : 1310
                                                                                                                                                               
                     *      1420         *      1440         *      1460         *      1480         *      1500         *      1520         *      1540       
Genomic   : CCGCGTTTTCTCCTTGGAGGAACTGGAAGAAGCGACTAACGACTTTGATGCAGCTAGCCTGTTCTGCGAACAGGTAAAAACAAAAACTTGACTTGAGACTCTGGCGACGAACCTAATAGATTACTTAATATATTTGGTAT : 1540
NM_126333 : CCGCGTTTTCTCCTTGGAGGAACTGGAAGAAGCGACTAACGACTTTGATGCAGCTAGCCTGTTCTGCGAACAG------------------------------------------------------------------- : 1416
FJ708689  : CCGCGTTTTCTCCTTGGAGGAACTGGAAGAAGCGACTAACGACTTTGATGCAGCTAGCCTGTTCTGCGAACAG------------------------------------------------------------------- : 1383
                                                                                                                                                               
                     *      1560         *      1580         *      1600         *      1620         *      1640         *      1660         *      1680       
Genomic   : TCGAACAGCTGTATAGAGGTTGTCTAAGAGAAGGCATACCAGTGACAGTGCGAGTTATCAAGCTGAAGCAGAAGAGCTTGCCACAAAGCTTAGCTCAACAGATGGAAGTTTTATCAAAGCTAAGGCATATGCATTTGGTT : 1680
NM_126333 : --------CTGTATAGAGGTTGTCTAAGAGAAGGCATACCAGTGACAGTGCGAGTTATCAAGCTGAAGCAGAAGAGCTTGCCACAAAGCTTAGCTCAACAGATGGAAGTTTTATCAAAGCTAAGGCATATGCATTTGGTT : 1548
FJ708689  : --------CTGTATAGAGGTTGTCTAAGAGAAGGCATACCAGTGACAGTGCGAGTTATCAAGCTGAAGCAGAAGAGCTTGCCACAAAGCTTAGCTCAACAGATGGAAGTTTTATCAAAGCTAAGGCATATGCATTTGGTT : 1515
                                                                                                                                                               
                     *      1700         *      1720         *      1740         *      1760         *      1780         *      1800         *      1820       
Genomic   : AGCGTTCTTGGACACTCCATTGCCAGTAACCAAGACCACAATCAACACGCTGGACACACCATCTTCATTGTTCAAGAATACATCTCTAGTGGATCATTGCGGGACTTTCTCACAAGTAATAATAACCTGAAAAAGATTAG : 1820
NM_126333 : AGCGTTCTTGGACACTCCATTGCCAGTAACCAAGACCACAATCAACACGCTGGACACACCATCTTCATTGTTCAAGAATACATCTCTAGTGGATCATTGCGGGACTTTCTCACAA------------------------- : 1663
FJ708689  : AGCGTTCTTGGACACTCCATTGCCAGTAACCAAGACCACAATCAACACGCTGGACACACCATCTTCATTGTTCAAGAATACATCTCTAGTGGATCATTGCGGGACTTTCTCACAA------------------------- : 1630
                                                                                                                                                               
                     *      1840         *      1860         *      1880         *      1900         *      1920         *      1940         *      1960       
Genomic   : AATATATATTCAGGTTTCCATAGACTCAAATGGTTATTCATTATGGGAATTTTTGTGATCAGACTGTAGGAAGAAAGAGGTACTGAAATGGCCTCAGAGAATGGCGATAGCAATAGGAGTCGCTCGAGGGATACAGTTCT : 1960
NM_126333 : --------------------------------------------------------------ACTGTAGGAAGAAAGAGGTACTGAAATGGCCTCAGAGAATGGCGATAGCAATAGGAGTCGCTCGAGGGATACAGTTCT : 1741
FJ708689  : --------------------------------------------------------------ACTGTAGGAAGAAAGAGGTACTGAAATGGCCTCAGAGAATGGCGATAGCAATAGGAGTCGCTCGAGGGATACAGTTCT : 1708
                                                                                                                                                               
                     *      1980         *      2000         *      2020         *      2040         *      2060         *      2080         *      2100       
Genomic   : TGCATATGGGAGTAGCACCAGGAATCTTTGGGAACAATTTGAAGATAGAGAACATCATGCTTGATGAAACACTCACAGTAAAAATCAGTGGCTATACTATTCCTTTACCATCCAAGGTATAAAACTTTGTTTTATTTCTT : 2100
NM_126333 : TGCATATGGGAGTAGCACCAGGAATCTTTGGGAACAATTTGAAGATAGAGAACATCATGCTTGATGAAACACTCACAGTAAAAATCAGTGGCTATACTATTCCTTTACCATCCAAGGT---------------------- : 1859
FJ708689  : TGCATATGGGAGTAGCACCAGGAATCTTTGGGAACAATTTGAAGATAGAGAACATCATGCTTGATGAAACACTCACAGTAAAAATCAGTGGCTATACTATTCCTTTACCATCCAAGGT---------------------- : 1826
                                                                                                                                                               
                     *      2120         *      2140         *      2160         *      2180         *      2200         *      2220         *      2240       
Genomic   : CTTCTATTAATTGAATCTAAGAAAATTTAATGGCATTTCTAGGTTGGAGAAGAGAGGCCTCAAGCCAAAAAACCTCGGAGGTTGTTGTTGTTTCTTAAGATATGATCAAATGTGTTCTCTTCTATCAAATTTTGAAACTT : 2240
NM_126333 : --------------------------------------------TGGAGAAGAGAGGCCTCAAGCCAAAAAACCTCGGAG------------------------------------------------------------ : 1895
FJ708689  : --------------------------------------------TGGAGAAGAGAGGCCTCAAGCCAAAAAACCTCGGAG------------------------------------------------------------ : 1862
                                                                                                                                                               
                     *      2260         *      2280         *      2300         *      2320         *      2340         *      2360         *      2380       
Genomic   : GTTAATGAATAATGTGCAGTAATGAAGATAGAGAGAAAGAAGATGTGTACCAGTTTGGAGTGATACTACTACAAATCATCACAGGGAAAGTAGTTGCTGCAGGATCTTCAGAGATGGGAAGTTTGAAGCTTCAGGTCTGT : 2380
NM_126333 : -------------------TAATGAAGATAGAGAGAAAGAAGATGTGTACCAGTTTGGAGTGATACTACTACAAATCATCACAGGGAAAGTAGTTGCTGCAGGATCTTCAGAGATGGGAAGTTTGAAGCTTCAG------ : 2010
FJ708689  : -------------------TAATGAAGATAGAGAGAAAGAAGATGTGTACCAGTTTGGAGTGATACTACTACAAATCATCACAGGGAAAGTAGTTGCTGCAGGATCTTCAGAGATGGGAAGTTTGAAGCTTCAG------ : 1977
                                                                                                                                                               
                
                     *      2400         *      2420         *      2440         *      2460         *      2480         *      2500         *      2520       
Genomic   : TCTGTTTCAGTCAGAACCTTTTCATCTTTAAGAATCCGACACGGCTTACAAATTTAGAACCTCAATGTTATTATTTCCTGCAGCTGGAGAACGGTTTGAGAGATGAACCATCAGTATTGAGCAGCTTAGCAGATCCATCT : 2520
NM_126333 : -----------------------------------------------------------------------------------CTGGAGAACGGTTTGAGAGATGAACCATCAGTATTGAGCAGCTTAGCAGATCCATCT : 2067
FJ708689  : -----------------------------------------------------------------------------------CTGGAGAACGGTTTGAGAGATGAACCATCAGTATTGAGCAGCTTAGCAGATCCATCT : 2034
                                                                                                                                                               
                     *      2540         *      2560         *      2580         *      2600         *      2620         *      2640         *      2660       
Genomic   : GTTAAAGGATCATATGCTTATGAGTCGTTGAGGACGACAGTAGAGTTTGCTATCAACTGTCTTTGCGAAGATCAGAGCAAGCGTCCTTCCATAGAAGACGTTGTGTGGAATCTACAGTATACAATTCAAGTGCAGCAAGG : 2660
NM_126333 : GTTAAAGGATCATATGCTTATGAGTCGTTGAGGACGACAGTAGAGTTTGCTATCAACTGTCTTTGCGAAGATCAGAGCAAGCGTCCTTCCATAGAAGACGTTGTGTGGAATCTACAGTATACAATTCAAGTGCAGCAAGG : 2207
FJ708689  : GTTAAAGGATCATATGCTTATGAGTCGTTGAGGACGACAGTAGAGTTTGCTATCAACTGTCTTTGCGAAGATCAGAGCAAGCGTCCTTCCATAGAAGACGTTGTGTGGAATCTACAGTATACAATTCAAGTGCAGCAAGG : 2174
                                                                       
                     *      2680         *      2700         *         
Genomic   : ATGGAGACCAAGCAGTGGGAATCATGAATCATCCATGAAAGCAATATATGAA : 2712
NM_126333 : ATGGAGACCAAGCAGTGGGAATCATGAATCATCCATGAAAGCAATATATGAA : 2259
FJ708689  : ATGGAGACCAAGCAGTGGGAATCATGAATCATCCATGAAAGCAATATATGAA : 2226


At3g21340
                                                                                                                                                               
                     *        20         *        40         *        60         *        80         *       100         *       120         *       140       
Genomic   : ATGGAGTACCATCCTCAAGCAATTAGGTTATGTGCGTTGATCTTCATCTCTTTCTATGCTCTTTTACACCTCGTTGAAGCACAAGACCAAAAAGGTATATACATATACACATTTCTTTGTTGTTTTTGATAATGTTTCGT :  140
NM_113029 : ATGGAGTACCATCCTCAAGCAATTAGGTTATGTGCGTTGATCTTCATCTCTTTCTATGCTCTTTTACACCTCGTTGAAGCACAAGACCAAAAAGG--------------------------------------------- :   95
FJ708725  : ATGGAGTACCATCCTCAAGCAATTAGGTTATGTGCGTTGATCTTCATCTCTTTCTATGCTCTTTTACACCTCGTTGAAGCACAAGACCAAAAAGG--------------------------------------------- :   95
                                                                                                                                                               
                     *       160         *       180         *       200         *       220         *       240         *       260         *       280       
Genomic   : CGAAATTATGTTCTTACTTAGTTTTCTGATACATTTTGTTCGCTTGTGTTACTAGGATTCATTAGTTTGGATTGCGGGTCATTGCCAAATGAGCCTCCTTACAACGATCCTTCAACCGGATTAACATACTCGACGGACGA :  280
NM_113029 : --------------------------------------------------------ATTCATTAGTTTGGATTGCGGGTCATTGCCAAATGAGCCTCCTTACAACGATCCTTCAACCGGATTAACATACTCGACGGACGA :  179
FJ708725  : --------------------------------------------------------ATTCATTAGTTTGGATTGCGGGTCATTGCCAAATGAGCCTCCTTACAACGATCCTTCAACCGGATTAACATACTCGACGGACGA :  179
                                                                                                                                                               
                     *       300         *       320         *       340         *       360         *       380         *       400         *       420       
Genomic   : TGGTTTCGTGCAGAGTGGCAAAACTGGAAGAATCCAGAAAGCGTTCGAGTCGATCTTCAGTAAACCGTCTTTGAAGCTTAGATACTTCCCGGACGGATTCCGAAACTGCTATACCTTGAATGTCACGCAAGACACAAACT :  420
NM_113029 : TGGTTTCGTGCAGAGTGGCAAAACTGGAAGAATCCAGAAAGCGTTCGAGTCGATCTTCAGTAAACCGTCTTTGAAGCTTAGATACTTCCCGGACGGATTCCGAAACTGCTATACCTTGAATGTCACGCAAGACACAAACT :  319
FJ708725  : TGGTTTCGTGCAGAGTGGCAAAACTGGAAGAATCCAGAAAGCGTTCGAGTCGATCTTCAGTAAACCGTCTTTGAAGCTTAGATACTTCCCGGACGGATTCCGAAACTGCTATACCTTGAATGTCACGCAAGACACAAACT :  319
                                                                                                                                                               
                     *       440         *       460         *       480         *       500         *       520         *       540         *       560       
Genomic   : ATCTGATCAAAGCTGTATTTGTGTATGGTAACTACGATGGTCTTAACAATCCCCCGAGTTTCGATCTTTACCTTGGTCCGAATCTATGGGTAACGGTTGATATGAATGGACGGACCAATGGTACTATCCAGGAGATTATC :  560
NM_113029 : ATCTGATCAAAGCTGTATTTGTGTATGGTAACTACGATGGTCTTAACAATCCCCCGAGTTTCGATCTTTACCTTGGTCCGAATCTATGGGTAACGGTTGATATGAATGGACGGACCAATGGTACTATCCAGGAGATTATC :  459
FJ708725  : ATCTGATCAAAGCTGTATTTGTGTATGGTAACTACGATGGTCTTAACAATCCCCCGAGTTTCGATCTTTACCTTGGTCCGAATCTATGGGTAACGGTTGATATGAATGGACGGACCAATGGTACTATCCAGGAGATTATC :  459
                                                                                                                                                               
                     *       580         *       600         *       620         *       640         *       660         *       680         *       700       
Genomic   : CACAAGACCATATCTAAGTCTCTCCAGGTCTGTCTTGTTAAGACAGGAACAAGCTCACCTATGATTAATACGTTAGAGCTACGACCACTTAAAAACAATACTTACAATACTCAGAGTGGCTCTCTGAAGTATTTCTTCCG :  700
NM_113029 : CACAAGACCATATCTAAGTCTCTCCAGGTCTGTCTTGTTAAGACAGGAACAAGCTCACCTATGATTAATACGTTAGAGCTACGACCACTTAAAAACAATACTTACAATACTCAGAGTGGCTCTCTGAAGTATTTCTTCCG :  599
FJ708725  : CACAAGACCATATCTAAGTCTCTCCAGGTCTGTCTTGTTAAGACAGGAACAAGCTCACCTATGATTAATACGTTAGAGCTACGACCACTTAAAAACAATACTTACAATACTCAGAGTGGCTCTCTGAAGTATTTCTTCCG :  599
                                                                                                                                                               
                     *       720         *       740         *       760         *       780         *       800         *       820         *       840       
Genomic   : ATATTATTTCAGCGGTTCAGGCCAAAACATACGGTATGAATCACGCCCTTTCACTTAATCATGAACAAAATTTGGTTTCTGGTTCTGTTTGATCCGTTTAGGATAAACTTTTTAACGAAGATTATTTGTTTTAAACCTTT :  840
NM_113029 : ATATTATTTCAGCGGTTCAGGCCAAAACATACGGTA-------------------------------------------------------------------------------------------------------- :  635
FJ708725  : ATATTATTTCAGCGGTTCAGGCCAAAACATACGGTA-------------------------------------------------------------------------------------------------------- :  635
                                                                                                                                                               
                     *       860         *       880         *       900         *       920         *       940         *       960         *       980       
Genomic   : AATAAATTTAGTGTATATATTGAAGTTTAAAGATTATAATTACTTATTAGGTTTTTGGTTGCACAAAACTCGGATTGGGTTCATTTGGTATAGACATTTTTCTAGCATCTTGGTTTATTAATATGGATTTTGATTTGTTT :  980
NM_113029 : -------------------------------------------------------------------------------------------------------------------------------------------- :    -
FJ708725  : -------------------------------------------------------------------------------------------------------------------------------------------- :    -
                                                                                                                                                               
                     *      1000         *      1020         *      1040         *      1060         *      1080         *      1100         *      1120       
Genomic   : CAGTAAGTTTGTTTTTAAGTAAAATGTTAGTCTTTAATACATGACAAAAATATTTTTGATAGGTACCCTGATGATGTCAATGATCGTAAATGGTATCCATTCTTTGATGCAAAAGAGTGGACAGAGTTAACAACCAATCT : 1120
NM_113029 : -----------------------------------------------------------------CCCTGATGATGTCAATGATCGTAAATGGTATCCATTCTTTGATGCAAAAGAGTGGACAGAGTTAACAACCAATCT :  710
FJ708725  : -----------------------------------------------------------------CCCTGATGATGTCAATGATCGTAAATGGTATCCATTCTTTGATGCAAAAGAGTGGACAGAGTTAACAACCAATCT :  710
                                                                                                                                                               
                     *      1140         *      1160         *      1180         *      1200         *      1220         *      1240         *      1260       
Genomic   : GAATATAAACAGTTCTAATGGTTATGCACCACCAGAAGTTGTGATGGCGTCAGCCTCAACGCCTATAAGTACTTTTGGAACATGGAACTTCTCATGGTTATTGCCATCTTCCACAACCCAATTTTATGTGTACATGCATT : 1260
NM_113029 : GAATATAAACAGTTCTAATGGTTATGCACCACCAGAAGTTGTGATGGCGTCAGCCTCAACGCCTATAAGTACTTTTGGAACATGGAACTTCTCATGGTTATTGCCATCTTCCACAACCCAATTTTATGTGTACATGCATT :  850
FJ708725  : GAATATAAACAGTTCTAATGGTTATGCACCACCAGAAGTTGTGATGGCGTCAGCCTCAACGCCTATAAGTACTTTTGGAACATGGAACTTCTCATGGTTATTGCCATCTTCCACAACCCAATTTTATGTGTACATGCATT :  850
                                                                                                                                                               
                     *      1280         *      1300         *      1320         *      1340         *      1360         *      1380         *      1400       
Genomic   : TTGCCGAGATTCAAACTCTACGGTCCCTCGATACCCGAGAATTCAAAGTGACGTTGAATGGAAAACTTGCTTATGAACGCTACAGCCCTAAAACGTTAGCCACCGAAACCATTTTCTATTCGACACCACAACAATGTGAA : 1400
NM_113029 : TTGCCGAGATTCAAACTCTACGGTCCCTCGATACCCGAGAATTCAAAGTGACGTTGAATGGAAAACTTGCTTATGAACGCTACAGCCCTAAAACGTTAGCCACCGAAACCATTTTCTATTCGACACCACAACAATGTGAA :  990
FJ708725  : TTGCCGAGATTCAAACTCTACGGTCCCTCGATACCCGAGAATTCAAAGTGACGTTGAATGGAAAACTTGCTTATGAACGCTACAGCCCTAAAACGTTAGCCACCGAAACCATTTTCTATTCGACACCACAACAATGTGAA :  990
                                                                                                                                                               
                     *      1420         *      1440         *      1460         *      1480         *      1500         *      1520         *      1540       
Genomic   : GATGGGACATGCCTCTTGGAGTTGACGAAAACACCTAAGTCTACTCTTCCTCCTCTCATGAACGCTCTTGAGGTTTTCACCGTGATCGATTTTCCACAGATGGAAACAAATCCAGATGATGGTAAGTCTATATGTTTCAA : 1540
NM_113029 : GATGGGACATGCCTCTTGGAGTTGACGAAAACACCTAAGTCTACTCTTCCTCCTCTCATGAACGCTCTTGAGGTTTTCACCGTGATCGATTTTCCACAGATGGAAACAAATCCAGATGATG------------------- : 1111
FJ708725  : GATGGGACATGCCTCTTGGAGTTGACGAAAACACCTAAGTCTACTCTTCCTCCTCTCATGAACGCTCTTGAGGTTTTCACCGTGATCGATTTTCCACAGATGGAAACAAATCCAGATGATG------------------- : 1111
                                                                                                                                                               
                     *      1560         *      1580         *      1600         *      1620         *      1640         *      1660         *      1680       
Genomic   : TCAATTTCCTCTATAAATATAATTTCATGGGTTGTTTTTTGATGCAACAATAACTTTATTCTAGTTGCTGCTATCAAGAGTATCCAAAGCACTTATGGATTAAGTAAAATCAGCTGGCAAGGAGATCCATGCGTTCCTAA : 1680
NM_113029 : ----------------------------------------------------------------TTGCTGCTATCAAGAGTATCCAAAGCACTTATGGATTAAGTAAAATCAGCTGGCAAGGAGATCCATGCGTTCCTAA : 1187
FJ708725  : ----------------------------------------------------------------TTGCTGCTATCAAGAGTATCCAAAGCACTTATGGATTAAGTAAAATCAGCTGGCAAGGAGATCCATGCGTTCCTAA : 1187
                                                                                                                                                               
                     *      1700         *      1720         *      1740         *      1760         *      1780         *      1800         *      1820       
Genomic   : ACAGTTTTTGTGGGAGGGTTTAAACTGCAATAATCTAGATAACTCCACGCCGCCTATTGTCACTTCCTTGTAAGATCAATCTTAAATTATTGTATTAGTTATGTCACATGATTTTATCATCTGTAAATGAAATGTGTGCA : 1820
NM_113029 : ACAGTTTTTGTGGGAGGGTTTAAACTGCAATAATCTAGATAACTCCACGCCGCCTATTGTCACTTCCTT----------------------------------------------------------------------- : 1256
FJ708725  : ACAGTTTTTGTGGGAGGGTTTAAACTGCAATAATCTAGATAACTCCACGCCGCCTATTGTCACTTCCTT----------------------------------------------------------------------- : 1256
                                                                                                                                                               
                     *      1840         *      1860         *      1880         *      1900         *      1920         *      1940         *      1960       
Genomic   : TGCATAAATATGTCTTTAGAAACTTATCGTCAAGTCATTTAACGGGGATCATCGCGCAAGGCATTCAGAATCTGACACACCTACAAGAACTGTAAGTATTTCGTATTTCAAAACTGAGCATAATGAAAACTATTTGTTCA : 1960
NM_113029 : -------------------AAACTTATCGTCAAGTCATTTAACGGGGATCATCGCGCAAGGCATTCAGAATCTGACACACCTACAAGAACT------------------------------------------------- : 1328
FJ708725  : -------------------AAACTTATCGTCAAGTCATTTAACGGGGATCATCGCGCAAGGCATTCAGAATCTGACACACCTACAAGAACT------------------------------------------------- : 1328
                                                                                                                                                               
                     *      1980         *      2000         *      2020         *      2040         *      2060         *      2080         *      2100       
Genomic   : CCTTAATTGTATCTTGTTTATAACATATTTTTGTACTTACAAATGCTTATGTAGAGACTTGTCAAATAACAATTTGACGGGAGGAATACCCGAATTTCTTGCTGACATAAAATCACTCTTAGTAATGTGAGTCGTTTTAA : 2100
NM_113029 : ------------------------------------------------------AGACTTGTCAAATAACAATTTGACGGGAGGAATACCCGAATTTCTTGCTGACATAAAATCACTCTTAGTAAT-------------- : 1400
FJ708725  : ------------------------------------------------------AGACTTGTCAAATAACAATTTGACGGGAGGAATACCCGAATTTCTTGCTGACATAAAATCACTCTTAGTAAT-------------- : 1400
                                                                                                                                                               
                     *      2120         *      2140         *      2160         *      2180         *      2200         *      2220         *      2240       
Genomic   : ACAAGACAATTTTTTATAATCTATTAGCCTCTTCGCACTACAAGATTGCCTATGTTTCTAACATTGTGTCTATCTTAACAGAAATTTAAGTGGGAACAATTTTAATGGCTCTATTCCTCAAATCCTTTTACAGAAGAAAG : 2240
NM_113029 : ---------------------------------------------------------------------------------AAATTTAAGTGGGAACAATTTTAATGGCTCTATTCCTCAAATCCTTTTACAGAAGAAAG : 1459
FJ708725  : ---------------------------------------------------------------------------------AAATTTAAGTGGGAACAATTTTAATGGCTCTATTCCTCAAATCCTTTTACAGAAGAAAG : 1459
                                                                                                                                                               
                     *      2260         *      2280         *      2300         *      2320         *      2340         *      2360         *      2380       
Genomic   : GACTAAAGCTAATGTAAGCTTTTCTCATATAATATTTTCATGACTATATAAAATTTGTGCTCAAGTCTCCTAAATGACTGGTAAATTTCTTTTTTTCCTCAACATTGGTAACTCATAGTCTTGAAGGAAACGCCAATCTG : 2380
NM_113029 : GACTAAAGCTAAT---------------------------------------------------------------------------------------------------------TCTTGAAGGAAACGCCAATCTG : 1494
FJ708725  : GACTAAAGCTAAT---------------------------------------------------------------------------------------------------------TCTTGAAGGAAACGCCAATCTG : 1494

                                                                                                                                                               
                     *      2400         *      2420         *      2440         *      2460         *      2480         *      2500         *      2520       
Genomic   : ATTTGTCCGGATGGATTATGTGTAAACAAAGCTGGCAATGGTGGTGCCAAGAAAATGAATGTTGTAATACCGATTGTTGCATCAGTTGCGTTTGTGGTTGTTCTTGGATCTGCATTGGCGTTCTTTTTTATTTTCAAAAA : 2520
NM_113029 : ATTTGTCCGGATGGATTATGTGTAAACAAAGCTGGCAATGGTGGTGCCAAGAAAATGAATGTTGTAATACCGATTGTTGCATCAGTTGCGTTTGTGGTTGTTCTTGGATCTGCATTGGCGTTCTTTTTTATTTTCAAAAA : 1634
FJ708725  : ATTTGTCCGGATGGATTATGTGTAAACAAAGCTGGCAATGGTGGTGCCAAGAAAATGAATGTTGTAATACCGATTGTTGCATCAGTTGCGTTTGTGGTTGTTCTTGGATCTGCATTGGCGTTCTTTTTTATTTTCAAAAA : 1634
                                                                                                                                                               
                     *      2540         *      2560         *      2580         *      2600         *      2620         *      2640         *      2660       
Genomic   : GAAAAAGACATCAAACAGTCAAGGTATACACAAAAAGAACCTTTAAATTGCCTTGAATACTGCAATACATATGTTCACTATGTATAAAAAGTCTCATTATACTGTTGCATTGTAACATTAGATTTAGGTCCATCTTCATA : 2660
NM_113029 : GAAAAAGACATCAAACAGTCAAG--------------------------------------------------------------------------------------------------------------------- : 1657
FJ708725  : GAAAAAGACATCAAACAGTCAAG--------------------------------------------------------------------------------------------------ATTTAGGTCCATCTTCATA : 1676
                                                                                                                                                               
                     *      2680         *      2700         *      2720         *      2740         *      2760         *      2780         *      2800       
Genomic   : TACTCAAGTATCAGAAGTTAGAACAATCAGATCTTCAGAGTCGGCAATAATGACTAAGAACAGAAGATTTACATATTCGGAGGTTGTAACAATGACAAATAACTTTGAAAGAGTTCTTGGTAAAGGAGGATTTGGAATGG : 2800
NM_113029 : --------------------------------------AGTCGGCAATAATGACTAAGAACAGAAGATTTACATATTCGGAGGTTGTAACAATGACAAATAACTTTGAAAGAGTTCTTGGTAAAGGAGGATTTGGAATGG : 1759
FJ708725  : TACTCAAGTATCAGAAGTTAGAACAATCAGATCTTCAGAGTCGGCAATAATGACTAAGAACAGAAGATTTACATATTCGGAGGTTGTAACAATGACAAATAACTTTGAAAGAGTTCTTGGTAAAGGAGGATTTGGAATGG : 1816
                                                                                                                                                               
                     *      2820         *      2840         *      2860         *      2880         *      2900         *      2920         *      2940       
Genomic   : TTTATCATGGAACTGTAAATAATACTGAACAAGTAGCCGTTAAAATGCTTTCACACTCATCTTCTCAAGGATATAAAGAATTCAAAGCAGAGGTAAGTTTTAGCAAGGTTATTAAGAAACGTTTGTAGAAACACTATCGT : 2940
NM_113029 : TTTATCATGGAACTGTAAATAATACTGAACAAGTAGCCGTTAAAATGCTTTCACACTCATCTTCTCAAGGATATAAAGAATTCAAAGCAGAGGT---------------------------------------------- : 1853
FJ708725  : TTTATCATGGAACTGTAAATAATACTGAACAAGTAGCCGTTAAAATGCTTTCACACTCATCTTCTCAAGGATATAAAGAATTCAAAGCAGAGGT---------------------------------------------- : 1910
                                                                                                                                                               
                     *      2960         *      2980         *      3000         *      3020         *      3040         *      3060         *      3080       
Genomic   : TTTATTAGAGTAACATGTCAAATTTTGATGTATTGCTCTTCCACTCCTCTAGGTGGAACTTCTTCTCAGAGTTCACCACAAAAATTTGGTTGGCCTCGTTGGATATTGTGATGAAGGAGAAAACTTGGCTCTTATCTACG : 3080
NM_113029 : ------------------------------------------------------GGAACTTCTTCTCAGAGTTCACCACAAAAATTTGGTTGGCCTCGTTGGATATTGTGATGAAGGAGAAAACTTGGCTCTTATCTACG : 1939
FJ708725  : ------------------------------------------------------GGAACTTCTTCTCAGAGTTCACCACAAAAATTTGGTTGGCCTCGTTGGATATTGTGATGAAGGAGAAAACTTGGCTCTTATCTACG : 1996
                                                                                                                                                               
                     *      3100         *      3120         *      3140         *      3160         *      3180         *      3200         *      3220       
Genomic   : AGTACATGGCTAACGGAGACTTGAGAGAACATATGTCAGGTAAGTATTTATAGTTGCTACAAATACATATTTGTTGTGGTGTTTTCACATATTTATATGTTGATTCAAATGTAGGAAAGCGAGGTGGATCTATTCTAAAT : 3220
NM_113029 : AGTACATGGCTAACGGAGACTTGAGAGAACATATGTC---------------------------------------------------------------------------AGGAAAGCGAGGTGGATCTATTCTAAAT : 2004
FJ708725  : AGTACATGGCTAACGGAGACTTGAGAGAACATATGTC---------------------------------------------------------------------------AGGAAAGCGAGGTGGATCTATTCTAAAT : 2061
                                                                                                                                                               
                     *      3240         *      3260         *      3280         *      3300         *      3320         *      3340         *      3360       
Genomic   : TGGGAAACTAGACTAAAAATAGTTGTCGAGTCTGCCCAAGGTTTGATATGAGTTTTAATATAATTTTTGCTTGTATCCTCTATTCATTTGCTTAACCCTTGATGAAAATGAAATTTGTTGCATAGGTTTGGAATACTTGC : 3360
NM_113029 : TGGGAAACTAGACTAAAAATAGTTGTCGAGTCTGCCCAAGGTTTG-------------------------------------------------------------------------------------GAATACTTGC : 2059
FJ708725  : TGGGAAACTAGACTAAAAATAGTTGTCGAGTCTGCCCAAGGTTTG-------------------------------------------------------------------------------------GAATACTTGC : 2116
                                                                                                                                                               
                     *      3380         *      3400         *      3420         *      3440         *      3460         *      3480         *      3500       
Genomic   : ATAATGGATGCAAACCACCAATGGTTCATAGGGATGTTAAAACCACAAATATATTGTTGAATGAACACCTCCATGCTAAGCTAGCTGATTTTGGGCTTTCGAGATCTTTTCCAATTGAAGGAGAAACTCATGTGTCAACA : 3500
NM_113029 : ATAATGGATGCAAACCACCAATGGTTCATAGGGATGTTAAAACCACAAATATATTGTTGAATGAACACCTCCATGCTAAGCTAGCTGATTTTGGGCTTTCGAGATCTTTTCCAATTGAAGGAGAAACTCATGTGTCAACA : 2199
FJ708725  : ATAATGGATGCAAACCACCAATGGTTCATAGGGATGTTAAAACCACAAATATATTGTTGAATGAACACCTCCATGCTAAGCTAGCTGATTTTGGGCTTTCGAGATCTTTTCCAATTGAAGGAGAAACTCATGTGTCAACA : 2256
                                                                                                                                                               
                     *      3520         *      3540         *      3560         *      3580         *      3600         *      3620         *      3640       
Genomic   : GTTGTTGCTGGAACTCCTGGATACCTTGATCCAGAGTAAGTTTCATAATTGTTGGGCAATGATTTTCTTTTAAATTTTATTTTAAGGGTAATTGCTAGTTTTCATCTATCATGAGTATCATCACTTGATAGGATATCATA : 3640
NM_113029 : GTTGTTGCTGGAACTCCTGGATACCTTGATCCAGA--------------------------------------------------------------------------------------------------------- : 2234
FJ708725  : GTTGTTGCTGGAACTCCTGGATACCTTGATCCAGA--------------------------------------------------------------------------------------------------------- : 2291
                                                                                                                                                               
                     *      3660         *      3680         *      3700         *      3720         *      3740         *      3760         *      3780       
Genomic   : TATATTTTTTATTTTTAATAGGATAAATATTGATTTTGCAGATATTACCGAACAAATTGGTTGAACGAGAAAAGTGATGTTTATAGCTTTGGAATTGTACTATTAGAGATCATCACAAACCAACTTGTGATCAATCAAAG : 3780
NM_113029 : -----------------------------------------ATATTACCGAACAAATTGGTTGAACGAGAAAAGTGATGTTTATAGCTTTGGAATTGTACTATTAGAGATCATCACAAACCAACTTGTGATCAATCAAAG : 2333
FJ708725  : -----------------------------------------ATATTACCGAACAAATTGGTTGAACGAGAAAAGTGATGTTTATAGCTTTGGAATTGTACTATTAGAGATCATCACAAACCAACTTGTGATCAATCAAAG : 2390
                                                                                                                                                               
                     *      3800         *      3820         *      3840         *      3860         *      3880         *      3900         *      3920       
Genomic   : TCGTGAAAAACCACATATAGCAGAATGGGTGGGGTTAATGCTTACAAAAGGAGACATTCAAAACATTATGGATCCAAAACTTTATGGTGATTATGACTCTGGTTCTGTCTGGAGAGCAGTTGAACTAGCAATGTCATGTC : 3920
NM_113029 : TCGTGAAAAACCACATATAGCAGAATGGGTGGGGTTAATGCTTACAAAAGGAGACATTCAAAACATTATGGATCCAAAACTTTATGGTGATTATGACTCTGGTTCTGTCTGGAGAGCAGTTGAACTAGCAATGTCATGTC : 2473
FJ708725  : TCGTGAAAAACCACATATAGCAGAATGGGTGGGGTTAATGCTTACAAAAGGAGACATTCAAAACATTATGGATCCAAAACTTTATGGTGATTATGACTCTGGTTCTGTCTGGAGAGCAGTTGAACTAGCAATGTCATGTC : 2530
                                                                                                                                                               
                     *      3940         *      3960         *      3980         *      4000         *      4020         *      4040         *      4060       
Genomic   : TAAATCCTTCTTCAGCTAGAAGACCAACAATGTCTCAAGTTGTTATCGAATTAAACGAATGTTTGTCATATGAAAACGCAAGAGGAGGAACGAGTCAAAACATGAACTCAGAGAGTTCAATAGAAGTCAGCATGAACTTT : 4060
NM_113029 : TAAATCCTTCTTCAGCTAGAAGACCAACAATGTCTCAAGTTGTTATCGAATTAAACGAATGTTTGTCATATGAAAACGCAAGAGGAGGAACGAGTCAAAACATGAACTCAGAGAGTTCAATAGAAGTCAGCATGAACTTT : 2613
FJ708725  : TAAATCCTTCTTCAGCTAGAAGACCAACAATGTCTCAAGTTGTTATCGAATTAAACGAATGTTTGTCATATGAAAACGCAAGAGGAGGAACGAGTCAAAACATGAACTCAGAGAGTTCAATAGAAGTCAGCATGAACTTT : 2670
                                              
                     *      4080              
Genomic   : GATATTGGAGCTACCCCTGATGCTCGT : 4087
NM_113029 : GATATTGGAGCTACCCCTGATGCTCGT : 2640
FJ708725  : GATATTGGAGCTACCCCTGATGCTCGT : 2697


At3g24660
                                                                                                                                                               
                     *        20         *        40         *        60         *        80         *       100         *       120         *       140       
Genomic   : ATGGGCATGGAAGCTTTGAGATTTCTTCATGTTATCTTCTTCTTTGTGCTAATTCTTCACTGTCATTGTGGAACATCTCTCTCTGGTTCTTCTGATGTGAAGCTTCTTTTAGGAAAAATCAAGTCTTCACTACAAGGAAA :  140
NM_113377 : ATGGGCATGGAAGCTTTGAGATTTCTTCATGTTATCTTCTTCTTTGTGCTAATTCTTCACTGTCATTGTGGAACATCTCTCTCTGGTTCTTCTGATGTGAAGCTTCTTTTAGGAAAAATCAAGTCTTCACTACAAGGAAA :  140
FJ708727  : ATGGGCATGGAAGCTTTGAGATTTCTTCATGTTATCTTCTTCTTTGTGCTAATTCTTCACTGTCATTGTGGAACATCTCTCTCTGGTTCTTCTGATGTGAAGCTTCTTTTAGGAAAAATCAAGTCTTCACTACAAGGAAA :  140
                                                                                                                                                               
                     *       160         *       180         *       200         *       220         *       240         *       260         *       280       
Genomic   : CAGTGAGAGCTTACTGTTGTCTTCTTGGAACTCCTCTGTTCCTGTTTGTCAATGGAGAGGTGTAAAATGGGTATTTTCAAATGGGTCTCCTCTTCAATGTAGTGACCTCTCTTCACCACAATGGACTAATACCTCTCTGT :  280
NM_113377 : CAGTGAGAGCTTACTGTTGTCTTCTTGGAACTCCTCTGTTCCTGTTTGTCAATGGAGAGGTGTAAAATGGGTATTTTCAAATGGGTCTCCTCTTCAATGTAGTGACCTCTCTTCACCACAATGGACTAATACCTCTCTGT :  280
FJ708727  : CAGTGAGAGCTTACTGTTGTCTTCTTGGAACTCCTCTGTTCCTGTTTGTCAATGGAGAGGTGTAAAATGGGTATTTTCAAATGGGTCTCCTCTTCAATGTAGTGACCTCTCTTCACCACAATGGACTAATACCTCTCTGT :  280
                                                                                                                                                               
                     *       300         *       320         *       340         *       360         *       380         *       400         *       420       
Genomic   : TCAACGACTCTTCTCTTCACCTTCTCTCTCTTCAGCTTCCTTCTGCTAATCTCACTGGTTCACTCCCTAGAGAGATTGGTGAGTTCTCTATGCTTCAAAGTGTGTTCCTCAACATCAATTCATTAAGTGGGTCAATCCCT :  420
NM_113377 : TCAACGACTCTTCTCTTCACCTTCTCTCTCTTCAGCTTCCTTCTGCTAATCTCACTGGTTCACTCCCTAGAGAGATTGGTGAGTTCTCTATGCTTCAAAGTGTGTTCCTCAACATCAATTCATTAAGTGGGTCAATCCCT :  420
FJ708727  : TCAACGACTCTTCTCTTCACCTTCTCTCTCTTCAGCTTCCTTCTGCTAATCTCACTGGTTCACTCCCTAGAGAGATTGGTGAGTTCTCTATGCTTCAAAGTGTGTTCCTCAACATCAATTCATTAAGTGGGTCAATCCCT :  420
                                                                                                                                                               
                     *       440         *       460         *       480         *       500         *       520         *       540         *       560       
Genomic   : CTTGAGCTTGGTTACACTTCTTCTCTCTCTGATGTTGATTTGAGTGGTAATGCCTTAGCTGGGGTTTTGCCTCCATCGATTTGGAACCTCTGTGATAAGCTTGTCTCTTTCAAGATTCATGGTAATAACTTGTCTGGGGT :  560
NM_113377 : CTTGAGCTTGGTTACACTTCTTCTCTCTCTGATGTTGATTTGAGTGGTAATGCCTTAGCTGGGGTTTTGCCTCCATCGATTTGGAACCTCTGTGATAAGCTTGTCTCTTTCAAGATTCATGGTAATAACTTGTCTGGGGT :  560
FJ708727  : CTTGAGCTTGGTTACACTTCTTCTCTCTCTGATGTTGATTTGAGTGGTAATGCCTTAGCTGGGGTTTTGCCTCCATCGATTTGGAACCTCTGTGATAAGCTTGTCTCTTTCAAGATTCATGGTAATAACTTGTCTGGGGT :  560
                                                                                                                                                               
                     *       580         *       600         *       620         *       640         *       660         *       680         *       700       
Genomic   : TTTGCCTGAGCCTGCTTTGCCAAATTCGACTTGTGGTAATCTCCAAGTTCTTGATTTGGGTGGTAATAAGTTCTCAGGTGAGTTTCCTGAGTTTATAACTAGGTTTAAAGGTGTGAAGTCACTTGATCTTTCAAGTAATG :  700
NM_113377 : TTTGCCTGAGCCTGCTTTGCCAAATTCGACTTGTGGTAATCTCCAAGTTCTTGATTTGGGTGGTAATAAGTTCTCAGGTGAGTTTCCTGAGTTTATAACTAGGTTTAAAGGTGTGAAGTCACTTGATCTTTCAAGTAATG :  700
FJ708727  : TTTGCCTGAGCCTGCTTTGCCAAATTCGACTTGTGGTAATCTCCAAGTTCTTGATTTGGGTGGTAATAAGTTCTCAG--------------------------------------------------------------- :  637
                                                                                                                                                               
                     *       720         *       740         *       760         *       780         *       800         *       820         *       840       
Genomic   : TCTTTGAAGGTCTTGTTCCTGAGGGTTTAGGTGTATTAGAGCTAGAAAGTCTCAATCTTTCTCATAATAACTTCAGTGGGATGTTGCCAGATTTTGGTGAGTCAAAGTTTGGAGCAGAATCTTTCGAAGGGAACAGTCCT :  840
NM_113377 : TCTTTGAAGGTCTTGTTCCTGAGGGTTTAGGTGTATTAGAGCTAGAAAGTCTCAATCTTTCTCATAATAACTTCAGTGGGATGTTGCCAGATTTTGGTGAGTCAAAGTTTGGAGCAGAATCTTTCGAAGGGAACAGTCCT :  840
FJ708727  : ------------------------------------------------------------------------------------------ATTTTGGTGAGTCAAAGTTTGGAGCAGAATCTTTCGAAGGGAACAGTCCT :  687
                                                                                                                                                               
                     *       860         *       880         *       900         *       920         *       940         *       960         *       980       
Genomic   : AGCCTTTGTGGTTTGCCTTTGAAGCCTTGTCTAGGCTCCTCTAGGTTAAGTCCAGGTGCTGTTGCTGGTCTGGTGATTGGTTTAATGTCTGGAGCTGTTGTTGTGGCCTCGTTGTTAATAGGGTATTTGCAGAACAAGAA :  980
NM_113377 : AGCCTTTGTGGTTTGCCTTTGAAGCCTTGTCTAGGCTCCTCTAGGTTAAGTCCAGGTGCTGTTGCTGGTCTGGTGATTGGTTTAATGTCTGGAGCTGTTGTTGTGGCCTCGTTGTTAATAGGGTATTTGCAGAACAAGAA :  980
FJ708727  : AGCCTTTGTGGTTTGCCTTTGAAGCCTTGTCTAGGCTCCTCTAGGTTAAGTCCAGGTGCTGTTGCTGGTCTGGTGATTGGTTTAATGTCTGGAGCTGTTGTTGTGGCCTCGTTGTTAATAGGGTATTTGCAGAACAAGAA :  827
                                                                                                                                                               
                     *      1000         *      1020         *      1040         *      1060         *      1080         *      1100         *      1120       
Genomic   : AAGAAAGAGTAGTATAGAGAGTGAAGATGATTTGGAAGAAGGTGATGAAGAAGATGAAATCGGTGAGAAAGAAGGCGGTGAAGGAAAGTTAGTTGTGTTTCAAGGTGGTGAGAATCTGACGTTGGATGATGTTTTGAATG : 1120
NM_113377 : AAGAAAGAGTAGTATAGAGAGTGAAGATGATTTGGAAGAAGGTGATGAAGAAGATGAAATCGGTGAGAAAGAAGGCGGTGAAGGAAAGTTAGTTGTGTTTCAAGGTGGTGAGAATCTGACGTTGGATGATGTTTTGAATG : 1120
FJ708727  : AAGAAAGAGTAGTATAGAGAGTGAAGATGATTTGGAAGAAGGTGATGAAGAAGATGAAATCGGTGAGAAAGAAGGCGGTGAAGGAAAGTTAGTTGTGTTTCAAGGTGGTGAGAATCTGACGTTGGATGATGTTTTGAATG :  967
                                                                                                                                                               
                     *      1140         *      1160         *      1180         *      1200         *      1220         *      1240         *      1260       
Genomic   : CTACTGGGCAAGTTATGGAGAAGACTAGCTATGGTACTGTCTATAAAGCTAAGCTTAGTGATGGAGGGAATATTGCATTGAGGCTATTGAGAGAAGGTACTTGCAAGGATAGAAGTTCTTGTCTGCCTGTTATAAGGCAG : 1260
NM_113377 : CTACTGGGCAAGTTATGGAGAAGACTAGCTATGGTACTGTCTATAAAGCTAAGCTTAGTGATGGAGGGAATATTGCATTGAGGCTATTGAGAGAAGGTACTTGCAAGGATAGAAGTTCTTGTCTGCCTGTTATAAGGCAG : 1260
FJ708727  : CTACTGGGCAAGTTATGGAGAAGACTAGCTATGGTACTGTCTATAAAGCTAAGCTTAGTGATGGAGGGAATATTGCATTGAGGCTATTGAGAGAAGGTACTTGCAAGGATAGAAGTTCTTGTCTGCCTGTTATAAGGCAG : 1107
                                                                                                                                                               
                     *      1280         *      1300         *      1320         *      1340         *      1360         *      1380         *      1400       
Genomic   : TTAGGACGCATTCGGCATGAGAATTTGGTTCCCTTGAGAGCTTTCTATCAAGGGAAGAGAGGAGAAAAGCTTCTCATCTATGACTATCTTCCCAACATAAGCTTACATGATTTGTTGCATGGTATTTTCTTTCATCATCA : 1400
NM_113377 : TTAGGACGCATTCGGCATGAGAATTTGGTTCCCTTGAGAGCTTTCTATCAAGGGAAGAGAGGAGAAAAGCTTCTCATCTATGACTATCTTCCCAACATAAGCTTACATGATTTGTTGCATG------------------- : 1381
FJ708727  : TTAGGACGCATTCGGCATGAGAATTTGGTTCCCTTGAGAGCTTTCTATCAAGGGAAGAGAGGAGAAAAGCTTCTCATCTATGACTATCTTCCCAACATAAGCTTACATGATTTGTTGCATG------------------- : 1228
                                                                                                                                                               
                     *      1420         *      1440         *      1460         *      1480         *      1500         *      1520         *      1540       
Genomic   : ATCTTTTTTGCCATGGATCTTTCTTGGTTGATTTTTGTTATTCATGTGTTGTTTGAATGATGTGCAGAAAGTAAACCTCGAAAGCCAGCTTTGAATTGGGCTAGGAGACACAAGATTGCACTTGGAATAGCGAGGGGACT : 1540
NM_113377 : -------------------------------------------------------------------AAAGTAAACCTCGAAAGCCAGCTTTGAATTGGGCTAGGAGACACAAGATTGCACTTGGAATAGCGAGGGGACT : 1454
FJ708727  : -------------------------------------------------------------------AAAGTAAACCTCGAAAGCCAGCTTTGAATTGGGCTAGGAGACACAAGATTGCACTTGGAATAGCGAGGGGACT : 1301
                                                                                                                                                               
                     *      1560         *      1580         *      1600         *      1620         *      1640         *      1660         *      1680       
Genomic   : TGCTTATCTTCATACTGGACAAGAAGTTCCTATCATCCATGGAAATATTAGATCAAAGAACGTGCTTGTGGACGACTTTTTCTTTGCAAGGCTAACTGAGTTTGGGCTTGACAAGATAATGGTACAGGCAGTAGCAGATG : 1680
NM_113377 : TGCTTATCTTCATACTGGACAAGAAGTTCCTATCATCCATGGAAATATTAGATCAAAGAACGTGCTTGTGGACGACTTTTTCTTTGCAAGGCTAACTGAGTTTGGGCTTGACAAGATAATGGTACAGGCAGTAGCAGATG : 1594
FJ708727  : TGCTTATCTTCATACTGGACAAGAAGTTCCTATCATCCATGGAAATATTAGATCAAAGAACGTGCTTGTGGACGACTTTTTCTTTGCAAGGCTAACTGAGTTTGGGCTTGACAAGATAATGGTACAGGCAGTAGCAGATG : 1441
                                                                                                                                                               
                     *      1700         *      1720         *      1740         *      1760         *      1780         *      1800         *      1820       
Genomic   : AGATTGTCTCGCAGGCGAAATCCGACGGGTACAAAGCACCTGAACTCCACAAGATGAAGAAATGCAATCCAAGGAGTGATGTTTACGCCTTTGGGATCCTTCTCTTGGAGATATTGATGGGTAAGAAACCAGGAAAGAGT : 1820
NM_113377 : AGATTGTCTCGCAGGCGAAATCCGACGGGTACAAAGCACCTGAACTCCACAAGATGAAGAAATGCAATCCAAGGAGTGATGTTTACGCCTTTGGGATCCTTCTCTTGGAGATATTGATGGGTAAGAAACCAGGAAAGAGT : 1734
FJ708727  : AGATTGTCTCGCAGGCGAAATCCGACGGGTACAAAGCACCTGAACTCCACAAGATGAAGAAATGCAATCCAAGGAGTGATGTTTACGCCTTTGGGATCCTTCTCTTGGAGATATTGATGGGTAAGAAACCAGGAAAGAGT : 1581
                                                                                                                                                               
                     *      1840         *      1860         *      1880         *      1900         *      1920         *      1940         *      1960       
Genomic   : GGAAGGAACGGTAATGAGTTTGTGGACTTGCCTTCTTTGGTTAAAGCTGCGGTGTTGGAAGAGACGACAATGGAGGTTTTCGACTTGGAGGCAATGAAAGGGATTAGGAGCCCAATGGAAGAAGGTTTGGTTCATGCATT : 1960
NM_113377 : GGAAGGAACGGTAATGAGTTTGTGGACTTGCCTTCTTTGGTTAAAGCTGCGGTGTTGGAAGAGACGACAATGGAGGTTTTCGACTTGGAGGCAATGAAAGGGATTAGGAGCCCAATGGAAGAAGGTTTGGTTCATGCATT : 1874
FJ708727  : GGAAGGAACGGTAATGAGTTTGTGGACTTGCCTTCTTTGGTTAAAGCTGCGGTGTTGGAAGAGACGACAATGGAGGTTTTCGACTTGGAGGCAATGAAAGGGATTAGGAGCCCAATGGAAGAAGGTTTGGTTCATGCATT : 1721
                                                                                                                                                               
                     *      1980         *      2000         *      2020         *      2040         *      2060         *      2080         *      2100       
Genomic   : GAAGCTAGCGATGGGATGTTGTGCTCCTGTTACAACAGTTAGACCCAGCATGGAAGAGGTTGTGAAGCAGTTGGAAGAGAACAGACCGAGGAATAGATCCGCATTGTACAGCCCAACCGAAACCAGGAGCGACGCCGAAA : 2100
NM_113377 : GAAGCTAGCGATGGGATGTTGTGCTCCTGTTACAACAGTTAGACCCAGCATGGAAGAGGTTGTGAAGCAGTTGGAAGAGAACAGACCGAGGAATAGATCCGCATTGTACAGCCCAACCGAAACCAGGAGCGACGCCGAAA : 2014
FJ708727  : GAAGCTAGCGATGGGATGTTGTGCTCCTGTTACAACAGTTAGACCCAGCATGGAAGAGGTTGTGAAGCAGTTGGAAGAGAACAGACCGAGGAATAGATCCGCATTGTACAGCCCAACCGAAACCAGGAGCGACGCCGAAA : 1861
                                                                                                                                                               
                           
Genomic   : CTCCATTT : 2108
NM_113377 : CTCCATTT : 2022
FJ708727  : CTCCATTT : 1869


At3g56100
                                                                                                                                                               
                     *        20         *        40         *        60         *        80         *       100         *       120         *       140       
Genomic   : ATGGAGTTTATCACCCAAAACCAAGCTATTACTTCTCTGAGTATGATCAACACCGACATTGATCAACCTAAGGCTTCACTTCGAAGCCGATTTCTCCTTCATCTAATCATATGCCTCCTCTTCTTCGTCCCTCCCTGCTC :  140
NM_115468 : ATGGAGTTTATCACCCAAAACCAAGCTATTACTTCTCTGAGTATGATCAACACCGACATTGATCAACCTAAGGCTTCACTTCGAAGCCGATTTCTCCTTCATCTAATCATATGCCTCCTCTTCTTCGTCCCTCCCTGCTC :  140
FJ708740  : ATGGAGTTTATCACCCAAAACCAAGCTATTACTTCTCTGAGTATGATCAACACCGACATTGATCAACCTAAGGCTTCACTTCGAAGCCGATTTCTCCTTCATCTAATCATATGCCTCCTCTTCTTCGTCCCTCCCTGCTC :  140
                                                                                                                                                               
                     *       160         *       180         *       200         *       220         *       240         *       260         *       280       
Genomic   : AAGCCAAGCCTGGGATGGTGTAGTGATAACACAAGCTGATTACCAAGGTCTTCAAGCAGTTAAACAAGAACTGATTGATCCAAGAGGCTTCTTGAGAAGCTGGAACGGTTCTGGATTCAGTGCTTGCTCTGGAGGCTGGG :  280
NM_115468 : AAGCCAAGCCTGGGATGGTGTAGTGATAACACAAGCTGATTACCAAGGTCTTCAAGCAGTTAAACAAGAACTGATTGATCCAAGAGGCTTCTTGAGAAGCTGGAACGGTTCTGGATTCAGTGCTTGCTCTGGAGGCTGGG :  280
FJ708740  : AAGCCAAGCCTGGGATGGTGTAGTGATAACACAAGCTGATTACCAAGGTCTTCAAGCAGTTAAACAAGAACTGATTGATCCAAGAGGCTTCTTGAGAAGCTGGAACGGTTCTGGATTCAGTGCTTGCTCTGGAGGCTGGG :  280
                                                                                                                                                               
                     *       300         *       320         *       340         *       360         *       380         *       400         *       420       
Genomic   : CTGGAATAAAATGTGCTCAAGGTCAAGTCATCGTCATTCAACTTCCATGGAAGAGTCTTGGAGGTAGAATCTCTGAGAAAATCGGACAGCTTCAAGCTCTTCGTAAGCTTAGTCTTCATGACAACAACCTTGGAGGTTCG :  420
NM_115468 : CTGGAATAAAATGTGCTCAAGGTCAAGTCATCGTCATTCAACTTCCATGGAAGAGTCTTGGAGGTAGAATCTCTGAGAAAATCGGACAGCTTCAAGCTCTTCGTAAGCTTAGTCTTCATGACAACAACCTTGGAGGTTCG :  420
FJ708740  : CTGGAATAAAATGTGCTCAAGGTCAAGTCATCGTCATTCAACTTCCATGGAAGAGTCTTGGAGGTAGAATCTCTGAGAAAATCGGACAGCTTCAAGCTCTTCGTAAGCTTAGTCTTCATGACAACAACCTTGGAGGTTCG :  420
                                                                                                                                                               
                     *       440         *       460         *       480         *       500         *       520         *       540         *       560       
Genomic   : ATTCCAATGTCATTGGGACTCATTCCTAATCTTAGAGGAGTTCAGCTTTTCAACAACCGTCTCACTGGCTCAATCCCTGCTTCTCTCGGTGTATCACATTTCCTTCAAACGCTTGATCTCAGCAATAACTTGCTCTCTGA :  560
NM_115468 : ATTCCAATGTCATTGGGACTCATTCCTAATCTTAGAGGAGTTCAGCTTTTCAACAACCGTCTCACTGGCTCAATCCCTGCTTCTCTCGGTGTATCACATTTCCTTCAAACGCTTGATCTCAGCAATAACTTGCTCTCTGA :  560
FJ708740  : ATTCCAATGTCATTGGGACTCATTCCTAATCTTAGAGGAGTTCAGCTTTTCAACAACCGTCTCACTGGCTCAATCCCTGCTTCTCTCGGTGTATCACATTTCCTTCAAACGCTTGATCTCAGCAATAACTTGCTCTCTGA :  560
                                                                                                                                                               
                     *       580         *       600         *       620         *       640         *       660         *       680         *       700       
Genomic   : GATTATTCCACCGAATCTTGCTGACTCTTCTAAGCTTCTTAGGCTTAATCTCAGCTTTAATTCACTGTCTGGTCAAATCCCAGTGAGTCTCTCTCGGTCTTCTTCCCTTCAGTTTCTTGCTCTTGACCATAACAACCTCT :  700
NM_115468 : GATTATTCCACCGAATCTTGCTGACTCTTCTAAGCTTCTTAGGCTTAATCTCAGCTTTAATTCACTGTCTGGTCAAATCCCAGTGAGTCTCTCTCGGTCTTCTTCCCTTCAGTTTCTTGCTCTTGACCATAACAACCTCT :  700
FJ708740  : GATTATTCCACCGAATCTTGCTGACTCTTCTAAGCTTCTTAGGCTTAATCTCAGCTTTAATTCACTGTCTGGTCAAATCCCAGTGAGTCTCTCTCGGTCTTCTTCCCTTCAGTTTCTTGCTCTTGACCATAACAACCTCT :  700
                                                                                                                                                               
                     *       720         *       740         *       760         *       780         *       800         *       820         *       840       
Genomic   : CTGGTCCAATCTTAGATACTTGGGGTAGTAAGTCTCTTAATCTCCGTGTCTTATCTCTTGATCACAACTCTCTCTCTGGTCCGTTCCCATTTTCACTCTGCAACTTAACTCAGCTGCAAGATTTTTTCCTTTAGTCATAA :  840
NM_115468 : CTGGTCCAATCTTAGATACTTGGGGTAGTAAG------------------------------------------------------------------------------------------------------------ :  732
FJ708740  : CTGGTCCAATCTTAGATACTTGGGGTAGTAAGTCTCTTAATCTCCGTGTCTTATCTCTTGATCACAACTCTCTCTCTGGTCCGTTCCCATTTTCACTCTGCAACTTAACTCAGCTGCAAGATTTTT-CCTTTAGTCATAA :  839
                                                                                                                                                               
                     *       860         *       880         *       900         *       920         *       940         *       960         *       980       
Genomic   : TAGGATCCGTGGAACCCTACCCTCTGAGCTAAGCAAACTCACTAAGCTTAGAAAAATGGATATTAGTGGTAACAGTGTTAGTGGCCATATCCCTGAAACCCTAGGGAACATTTCTTCTCTTATACATTTGGATTTGTCTC :  980
NM_115468 : ----ATCCGTGGAACCCTACCCTCTGAGCTAAGCAAACTCACTAAGCTTAGAAAAATGGATATTAGTGGTAACAGTGTTAGTGGCCATATCCCTGAAACCCTAGGGAACATTTCTTCTCTTATACATTTGGATTTGTCTC :  868
FJ708740  : TAGGATCCGTGGAACCCTACCCTCTGAGCTAAGCAAACTCACTAAGCTTAGAAAAATGGATATTAGTGGTAACAGTGTTAGTGGCCATATCCCTGAAACCCTAGGGAACATTTCTTCTCTTATACATTTGGATTTGTCTC :  979
                                                                                                                                                               
                     *      1000         *      1020         *      1040         *      1060         *      1080         *      1100         *      1120       
Genomic   : AAAACAAACTCACCGGAGAGATTCCTATTTCGATTTCTGACCTGGAAAGCCTGAACTTCTTCAATGTCTCTTATAACAACTTATCTGGTCCTGTTCCTACTCTCTTGTCCCAAAAGTTCAATTCCTCTTCTTTTGTCGGG : 1120
NM_115468 : AAAACAAACTCACCGGAGAGATTCCTATTTCGATTTCTGACCTGGAAAGCCTGAACTTCTTCAATGTCTCTTATAACAACTTATCTGGTCCTGTTCCTACTCTCTTGTCCCAAAAGTTCAATTCCTCTTCTTTTGTCGGG : 1008
FJ708740  : AAAACAAACTCACCGGAGAGATTCCTATTTCGATTTCTGACCTGGAAAGCCTGAACTTCTTCAATGTCTCTTATAACAACTTATCTGGTCCTGTTCCTACTCTCTTGTCCCAAAAGTTCAATTCCTCTTCTTTTGTCGGG : 1119
                                                                                                                                                               
                     *      1140         *      1160         *      1180         *      1200         *      1220         *      1240         *      1260       
Genomic   : AACTCACTGCTTTGCGGATACAGCGTTTCGACACCATGTCCTACTCTTCCTTCACCGTCTCCTGAAAAAGAAAGGAAACCATCCCACAGGAATCTGAGTACCAAAGACATCATCCTCATTGCGTCTGGAGCACTCCTCAT : 1260
NM_115468 : AACTCACTGCTTTGCGGATACAGCGTTTCGACACCATGTCCTACTCTTCCTTCACCGTCTCCTGAAAAAGAAAGGAAACCATCCCACAGGAATCTGAGTACCAAAGACATCATCCTCATTGCGTCTGGAGCACTCCTCAT : 1148
FJ708740  : AACTCACTGCTTTGCGGATACAGCGTTTCGACACCATGTCCTACTCTTCCTTCACCGTCTCCTGAAAAAGAAAGGAAACCATCCCACAGGAATCTGAGTACCAAAGACATCATCCTCATTGCGTCTGGAGCACTCCTCAT : 1259
                                                                                                                                                               
                     *      1280         *      1300         *      1320         *      1340         *      1360         *      1380         *      1400       
Genomic   : AGTTATGCTTATCCTTGTTTGTGTTCTATGTTGCTTGCTGAGGAAGAAAGCCAATGAAACCAAAGCCAAGGGCGGAGAGGCTGGACCTGGAGCTGTAGCCGCAAAGACTGAGAAAGGAGGGGAAGCTGAAGCTGGAGGTG : 1400
NM_115468 : AGTTATGCTTATCCTTGTTTGTGTTCTATGTTGCTTGCTGAGGAAGAAAGCCAATGAAACCAAAGCCAAGGGCGGAGAGGCTGGACCTGGAGCTGTAGCCGCAAAGACTGAGAAAGGAGGGGAAGCTGAAGCTGGAGGTG : 1288
FJ708740  : AGTTATGCTTATCCTTGTTTGTGTTCTATGTTGCTTGCTGAGGAAGAAAGCCAATGAAACCAAAGCCAAGGGCGGAGAGGCTGGACCTGGAGCTGTAGCCGCAAAGACTGAGAAAGGAGGGGAAGCTGAAGCTGGAGGTG : 1399
                                                                                                                                                               
                     *      1420         *      1440         *      1460         *      1480         *      1500         *      1520         *      1540       
Genomic   : AAACTGGAGGGAAGCTGGTTCATTTTGATGGACCAATGGCGTTTACTGCAGATGATCTTTTGTGTGCAACAGCAGAGATAATGGGGAAAAGCACTTATGGGACTGTGTACAAAGCTACACTTGAAGATGGAAGTCAAGTA : 1540
NM_115468 : AAACTGGAGGGAAGCTGGTTCATTTTGATGGACCAATGGCGTTTACTGCAGATGATCTTTTGTGTGCAACAGCAGAGATAATGGGGAAAAGCACTTATGGGACTGTGTACAAAGCTACACTTGAAGATGGAAGTCAAGTA : 1428
FJ708740  : AAACTGGAGGGAAGCTGGTTCATTTTGATGGACCAATGGCGTTTACTGCAGATGATCTTTTGTGTGCAACAGCAGAGATAATGGGGAAAAGCACTTATGGGACTGTGTACAAAGCTACACTTGAAGATGGAAGTCAAGTA : 1539
                                                                                                                                                               
                     *      1560         *      1580         *      1600         *      1620         *      1640         *      1660         *      1680       
Genomic   : GCAGTGAAGAGATTGAGAGAAA-GATCACCAAAAGTCAAAAAGAGTTTGAGAATGAGATTAACGTCTTGGGAAGAATCCGGCATCCGAATCTCCTTGCACTCAGAGCTTATTACTTAGGCCCTAAAGGAGAGAAGCTTGT : 1679
NM_115468 : GCAGTGAAGAGATTGAGAGAAA-GATCACCAAAAGTCAAAAAGAG-----------------------------------------------------------------------------------AGAGAAGCTTGT : 1484
FJ708740  : GCAGTGAAGAGATTGAGAGAAAAGATCACCAAAAGTCAAAAAGAGTTTGAGAATGAGATTAACGTCTTGGGAAGAATCCGGCATCCGAATCTCCTTGCACTCAGAGCTTATTACTTAGGCCCTAAAGGAGAGAAGCTTGT : 1679
                                                                                                                                                               
                     *      1700         *      1720         *      1740         *      1760         *      1780         *      1800         *      1820       
Genomic   : CGTCTTCGATTACATGTCTAGAGGAAGCCTTGCCACGTTTCTCCATGGTAAGTTTGATACTAAAGTCTTAAAGTTAAAGACAATTTTGCTTTACAAGTACCATTTTGTAACTCTGTTTTCATATTTGAAATTGCAGCAAG : 1819
NM_115468 : CGTCTTCGATTACATGTCTAGAGGAAGCCTTGCCACGTTTCTCCATG-----------------------------------------------------------------------------------------CAAG : 1535
FJ708740  : CGTCTTCGATTACATGTCTAGAGGAAGCCTTGCCACGTTTCTCCATG-----------------------------------------------------------------------------------------CAAG : 1730
                                                                                                                                                               
                     *      1840         *      1860         *      1880         *      1900         *      1920         *      1940         *      1960       
Genomic   : AGGACCAGATGTACATATCAATTGGCCTACAAGGATGAGCTTAATAAAGGGAATGGCTCGAGGCTTGTTCTACCTTCACACACACGCAAATATCATCCACGGAAACTTAACATCAAGCAATGTGCTTTTGGATGAGAACA : 1959
NM_115468 : AGGACCAGATGTACATATCAATTGGCCTACAAGGATGAGCTTAATAAAGGGAATGGCTCGAGGCTTGTTCTACCTTCACACACACGCAAATATCATCCACGGAAACTTAACATCAAGCAATGTGCTTTTGGATGAGAACA : 1675
FJ708740  : AGGACCAGATGTACATATCAATTGGCCTACAAGGATGAGCTTAATAAAGGGAATGGCTCGAGGCTTGTTCTACCTTCACACACACGCAAATATCATCCACGGAAACTTAACATCAAGCAATGTGCTTTTGGATGAGAACA : 1870
                                                                                                                                                               
                     *      1980         *      2000         *      2020         *      2040         *      2060         *      2080         *      2100       
Genomic   : TAACCGCGAAGATCTCAGATTACGGTCTCTCAAGGCTAATGACAGCAGCAGCAGGATCCAGCGTGATTGCAACAGCTGGTGCGTTAGGTTACAGAGCACCTGAGCTCTCTAAGCTGAAGAAAGCCAACACGAAAACCGAT : 2099
NM_115468 : TAACCGCGAAGATCTCAGATTACGGTCTCTCAAGGCTAATGACAGCAGCAGCAGGATCCAGCGTGATTGCAACAGCTGGTGCGTTAGGTTACAGAGCACCTGAGCTCTCTAAGCTGAAGAAAGCCAACACGAAAACCGAT : 1815
FJ708740  : TAACCGCGAAGATCTCAGATTACGGTCTCTCAAGGCTAATGACAGCAGCAGCAGGATCCAGCGTGATTGCAACAGCTGGTGCGTTAGGTTACAGAGCACCTGAGCTCTCTAAGCTGAAGAAAGCCAACACGAAAACCGAT : 2010
                                                                                                                                                               
                     *      2120         *      2140         *      2160         *      2180         *      2200         *      2220         *      2240       
Genomic   : GTGTACAGCCTCGGTGTGATCATATTGGAACTGTTGACTGGGAAATCTCCGAGTGAGGCCTTAAACGGTGTGGATTTGCCTCAATGGGTTGCTACTGCGGTTAAAGAAGAGTGGACTAATGAGGTTTTTGATTTGGAGCT : 2239
NM_115468 : GTGTACAGCCTCGGTGTGATCATATTGGAACTGTTGACTGGGAAATCTCCGAGTGAGGCCTTAAACGGTGTGGATTTGCCTCAATGGGTTGCTACTGCGGTTAAAGAAGAGTGGACTAATGAGGTTTTTGATTTGGAGCT : 1955
FJ708740  : GTGTACAGCCTCGGTGTGATCATATTGGAACTGTTGACTGGGAAATCTCCGAGTGAGGCCTTAAACGGTGTGGATTTGCCTCAATGGGTTGCTACTGCGGTTAAAGAAGAGTGGACTAATGAGGTTTTTGATTTGGAGCT : 2150
                                                                                                                                                               
                     *      2260         *      2280         *      2300         *      2320         *      2340         *      2360         *      2380       
Genomic   : GTTGAATGATGTGAACACAATGGGTGATGAGATTTTGAATACACTGAAACTGGCTTTACATTGTGTTGATGCTACACCATCAACAAGACCTGAAGCTCAACAAGTGATGACACAACTTGGAGAGATTAGACCGGAAGAGA : 2379
NM_115468 : GTTGAATGATGTGAACACAATGGGTGATGAGATTTTGAATACACTGAAACTGGCTTTACATTGTGTTGATGCTACACCATCAACAAGACCTGAAGCTCAACAAGTGATGACACAACTTGGAGAGATTAGACCGGAAGAGA : 2095
FJ708740  : GTTGAATGATGTGAACACAATGGGTGATGAGATTTTGAATACACTGAAACTGGCTTTACATTGTGTTGATGCTACACCATCAACAAGACCTGAAGCTCAACAAGTGATGACACAACTTGGAGAGATTAGACCGGAAGAGA : 2290
                                 
                                                                                                                              
                     *      2400         *      2420         *      2440         
Genomic   : CAACAGCAACAACATCAGAACCGTTGATTGATGTCCCTGAAGCTTCTGCTTCCACAAGTCAA : 2441
NM_115468 : CAACAGCAACAACATCAGAACCGTTGATTGATGTCCCTGAAGCTTCTGCTTCCACAAGTCAA : 2157
FJ708740  : CAACAGCAACAACATCAGAACCGTTGATTGATGTCCCTGAAGCTTCTGCTTCCACAAGTCAA : 2352


At4g20270
                                                                                                                                                               
                     *        20         *        40         *        60         *        80         *       100         *       120         *       140       
Genomic   : ATGGCAGACAAGATCTTCACTTTCTTCCTAATCTTGTCTTCGATCTCTCCTCTCTTATGCTCTTCTTTGATCTCACCTCTTAATCTCTCACTTATTAGACAAGCAAATGTCCTTATCTCTCTAAAGCAAAGTTTTGATTC :  140
NM_118146 : ATGGCAGACAAGATCTTCACTTTCTTCCTAATCTTGTCTTCGATCTCTCCTCTCTTATGCTCTTCTTTGATCTCACCTCTTAATCTCTCACTTATTAGACAAGCAAATGTCCTTATCTCTCTAAAGCAAAGTTTTGATTC :  140
FJ708747  : ATGGCAGACAAGATCTTCACTTTCTTCCTAATCTTGTCTTCGATCTCTCCTCTCTTATGCTCTTCTTTGATCTCACCTCTTAATCTCTCACTTATTAGACAAGCAAATGTCCTTATCTCTCTAAAGCAAAGTTTTGATTC :  140
                                                                                                                                                               
                     *       160         *       180         *       200         *       220         *       240         *       260         *       280       
Genomic   : CTATGATCCTTCTCTTGATTCATGGAACATTCCAAATTTCAACTCTCTATGTTCTTGGACTGGTGTTTCTTGTGACAACTTGAATCAGTCTATTACTCGTCTAGACCTATCTAATCTCAACATCTCCGGCACTATCTCTC :  280
NM_118146 : CTATGATCCTTCTCTTGATTCATGGAACATTCCAAATTTCAACTCTCTATGTTCTTGGACTGGTGTTTCTTGTGACAACTTGAATCAGTCTATTACTCGTCTAGACCTATCTAATCTCAACATCTCCGGCACTATCTCTC :  280
FJ708747  : CTATGATCCTTCTCTTGATTCATGGAACATTCCAAATTTCAACTCTCTATGTTCTTGGACTGGTGTTTCTTGTGACAACTTGAATCAGTCTATTACTCGTCTAGACCTATCTAATCTCAACATCTCCGGCACTATCTCTC :  280
                                                                                                                                                               
                     *       300         *       320         *       340         *       360         *       380         *       400         *       420       
Genomic   : CGGAAATATCTCGTCTTTCGCCGTCACTTGTTTTTCTTGACATTTCTTCTAACAGTTTCTCCGGTGAGCTTCCTAAAGAGATCTATGAGCTCTCAGGCCTCGAAGTGTTAAACATCTCTAGCAATGTTTTTGAAGGAGAG :  420
NM_118146 : CGGAAATATCTCGTCTTTCGCCGTCACTTGTTTTTCTTGACATTTCTTCTAACAGTTTCTCCGGTGAGCTTCCTAAAGAGATCTATGAGCTCTCAGGCCTCGAAGTGTTAAACATCTCTAGCAATGTTTTTGAAGGAGAG :  420
FJ708747  : CGGAAATATCTCGTCTTTCGCCGTCACTTGTTTTTCTTGACATTTCTTCTAACAGTTTCTCCGGTGAGCTTCCTAAAGAGATCTATGAGCTCTCAGGCCTCGAAGTGTTAAACATCTCTAGCAATGTTTTTGAAGGAGAG :  420
                                                                                                                                                               
                     *       440         *       460         *       480         *       500         *       520         *       540         *       560       
Genomic   : CTGGAGACACGTGGGTTCAGTCAAATGACTCAGCTTGTGACTCTTGACGCTTACGACAACAGCTTCAACGGATCACTTCCTCTGAGTCTAACCACACTCACTCGTCTCGAGCACTTAGATCTTGGAGGAAACTACTTCGA :  560
NM_118146 : CTGGAGACACGTGGGTTCAGTCAAATGACTCAGCTTGTGACTCTTGACGCTTACGACAACAGCTTCAACGGATCACTTCCTCTGAGTCTAACCACACTCACTCGTCTCGAGCACTTAGATCTTGGAGGAAACTACTTCGA :  560
FJ708747  : CTGGAGACACGTGGGTTCAGTCAAATGACTCAGCTTGTGACTCTTGACGCTTACGACAACAGCTTCAACGGATCACTTCCTCTGAGTCTAACCACACTCACTCGTCTCGAGCACTTAGATCTTGGAGGAAACTACTTCGA :  560
                                                                                                                                                               
                     *       580         *       600         *       620         *       640         *       660         *       680         *       700       
Genomic   : CGGTGAGATCCCTAGAAGCTATGGAAGTTTCTTGAGTCTCAAGTTTCTTTCTTTATCTGGTAATGATCTCCGTGGGAGAATCCCTAACGAGCTAGCGAACATCACGACTTTGGTACAGCTTTACTTAGGTTACTACAACG :  700
NM_118146 : CGGTGAGATCCCTAGAAGCTATGGAAGTTTCTTGAGTCTCAAGTTTCTTTCTTTATCTGGTAATGATCTCCGTGGGAGAATCCCTAACGAGCTAGCGAACATCACGACTTTGGTACAGCTTTACTTAGGTTACTACAACG :  700
FJ708747  : CGGTGAGATCCCTAGAAGCTATGGAAGTTTCTTGAGTCTCAAGTTTCTTTCTTTATCTGGTAATGATCTCCGTGGGAGAATCCCTAACGAGCTAGCGAACATCACGACTTTGGTACAGCTTTACTTAGGTTACTACAACG :  700
                                                                                                                                                               
                     *       720         *       740         *       760         *       780         *       800         *       820         *       840       
Genomic   : ATTACCGCGGTGGGATACCTGCAGATTTCGGGAGATTGATCAATCTTGTTCATTTGGATTTAGCTAATTGCAGCTTGAAAGGATCAATTCCTGCAGAATTGGGGAATCTCAAGAACTTGGAGGTTCTGTTTCTTCAGACC :  840
NM_118146 : ATTACCGCGGTGGGATACCTGCAGATTTCGGGAGATTGATCAATCTTGTTCATTTGGATTTAGCTAATTGCAGCTTGAAAGGATCAATTCCTGCAGAATTGGGGAATCTCAAGAACTTGGAGGTTCTGTTTCTTCAGACC :  840
FJ708747  : ATTACCGCGGTGGGATACCTGCAGATTTCGGGAGATTGATCAATCTTGTTCATTTGGATTTAGCTAATTGCAGCTTGAAAGGATCAATTCCTGCAGAATTGGGGAATCTCAAGAACTTGGAGGTTCTGTTTCTTCAGACC :  840
                                                                                                                                                               
                     *       860         *       880         *       900         *       920         *       940         *       960         *       980       
Genomic   : AATGAGCTTACAGGCTCTGTTCCTCGAGAGTTAGGGAACATGACAAGCCTCAAGACTCTTGATCTCTCCAACAACTTTCTTGAAGGAGAGATTCCTCTAGAGCTATCTGGACTTCAAAAGCTTCAGTTGTTTAACCTCTT :  980
NM_118146 : AATGAGCTTACAGGCTCTGTTCCTCGAGAGTTAGGGAACATGACAAGCCTCAAGACTCTTGATCTCTCCAACAACTTTCTTGAAGGAGAGATTCCTCTAGAGCTATCTGGACTTCAAAAGCTTCAGTTGTTTAACCTCTT :  980
FJ708747  : AATGAGCTTACAGGCTCTGTTCCTCGAGAGTTAGGGAACATGACAAGCCTCAAGACTCTTGATCTCTCCAACAACTTTCTTGAAGGAGAGATTCCTCTAGAGCTATCTGGACTTCAAAAGCTTCAGTTGTTTAACCTCTT :  980
                                                                                                                                                               
                     *      1000         *      1020         *      1040         *      1060         *      1080         *      1100         *      1120       
Genomic   : CTTCAACAGACTACACGGCGAGATCCCTGAGTTCGTATCTGAGCTTCCTGATCTGCAAATACTCAAGCTTTGGCACAACAATTTCACCGGAAAGATTCCTTCGAAACTCGGATCAAACGGGAACTTGATCGAGATCGATT : 1120
NM_118146 : CTTCAACAGACTACACGGCGAGATCCCTGAGTTCGTATCTGAGCTTCCTGATCTGCAAATACTCAAGCTTTGGCACAACAATTTCACCGGAAAGATTCCTTCGAAACTCGGATCAAACGGGAACTTGATCGAGATCGATT : 1120
FJ708747  : CTTCAACAGACTACACGGCGAGATCCCTGAGTTCGTATCTGAGCTTCCTGATCTGCAAATACTCAAGCTTTGGCACAACAATTTCACCGGAAAGATTCCTTCGAAACTCGGATCAAACGGGAACTTGATCGAGATCGATT : 1120
                                                                                                                                                               
                     *      1140         *      1160         *      1180         *      1200         *      1220         *      1240         *      1260       
Genomic   : TGTCTACCAATAAACTCACAGGTTTGATCCCTGAGTCACTCTGTTTCGGAAGAAGACTAAAGATTCTCATTCTCTTCAACAACTTCTTGTTCGGTCCTCTCCCTGAAGATCTTGGCCAATGTGAACCGCTATGGAGATTC : 1260
NM_118146 : TGTCTACCAATAAACTCACAGGTTTGATCCCTGAGTCACTCTGTTTCGGAAGAAGACTAAAGATTCTCATTCTCTTCAACAACTTCTTGTTCGGTCCTCTCCCTGAAGATCTTGGCCAATGTGAACCGCTATGGAGATTC : 1260
FJ708747  : TGTCTACCAATAAACTCACAG---------------------------------------------------------------------------------------ATCTTGGCCAATGTGAACCGCTATGGAGATTC : 1173
                                                                                                                                                               
                     *      1280         *      1300         *      1320         *      1340         *      1360         *      1380         *      1400       
Genomic   : CGTCTCGGACAGAACTTTCTGACAAGTAAGTTGCCAAAGGGTTTGATTTATTTGCCGAATCTTTCGCTTCTTGAGCTTCAAAACAACTTTTTGACTGGAGAAATCCCCGAAGAAGAGGCGGGAAATGCGCAGTTTTCGAG : 1400
NM_118146 : CGTCTCGGACAGAACTTTCTGACAAGTAAGTTGCCAAAGGGTTTGATTTATTTGCCGAATCTTTCGCTTCTTGAGCTTCAAAACAACTTTTTGACTGGAGAAATCCCCGAAGAAGAGGCGGGAAATGCGCAGTTTTCGAG : 1400
FJ708747  : CGTCTCGGACAGAACTTTCTGACAAGTAAGTTGCCAAAGGGTTTGATTTATTTGCCGAATCTTTCGCTTCTTGAGCTTCAAAACAACTTTTTGACTGGAGAAATCCCCGAAGAAGAGGCGGGAAATGCGCAGTTTTCGAG : 1313
                                                                                                                                                               
                     *      1420         *      1440         *      1460         *      1480         *      1500         *      1520         *      1540       
Genomic   : CCTTACTCAGATCAATCTGTCCAACAACAGGTTATCCGGACCGATTCCTGGTTCAATCAGAAACCTCAGAAGCCTTCAGATTCTTCTTCTCGGTGCAAACCGGTTATCGGGACAGATCCCTGGCGAAATCGGAAGTTTGA : 1540
NM_118146 : CCTTACTCAGATCAATCTGTCCAACAACAGGTTATCCGGACCGATTCCTGGTTCAATCAGAAACCTCAGAAGCCTTCAGATTCTTCTTCTCGGTGCAAACCGGTTATCGGGACAGATCCCTGGCGAAATCGGAAGTTTGA : 1540
FJ708747  : CCTTACTCAGATCAATCTGTCCAACAACAGGTTATCCGGACCGATTCCTGGTTCAATCAGAAACCTCAGAAGCCTTCAGATTCTTCTTCTCGGTGCAAACCGGTTATCGGGACAGATCCCTGGCGAAATCGGAAGTTTGA : 1453
                                                                                                                                                               
                     *      1560         *      1580         *      1600         *      1620         *      1640         *      1660         *      1680       
Genomic   : AGAGTCTTCTCAAGATTGACATGAGCAGAAACAACTTCTCAGGCAAGTTTCCTCCTGAGTTTGGTGATTGCATGTCACTCACATATTTAGATTTGAGTCACAACCAGATTTCCGGTCAGATTCCGGTTCAGATATCGCAG : 1680
NM_118146 : AGAGTCTTCTCAAGATTGACATGAGCAGAAACAACTTCTCAGGCAAGTTTCCTCCTGAGTTTGGTGATTGCATGTCACTCACATATTTAGATTTGAGTCACAACCAGATTTCCGGTCAGATTCCGGTTCAGATATCGCAG : 1680
FJ708747  : AGAGTCTTCTCAAGATTGACATGAGCAGAAACAACTTCTCAGGCAAGTTTCCTCCTGAGTTTGGTGATTGCATGTCACTCACATATTTAGATTTGAGTCACAACCAGATTTCCGGTCAGATTCCGGTTCAGATATCGCAG : 1593
                                                                                                                                                               
                     *      1700         *      1720         *      1740         *      1760         *      1780         *      1800         *      1820       
Genomic   : ATTCGGATTCTAAACTATCTGAATGTTTCTTGGAATTCCTTTAACCAAAGCCTTCCCAACGAACTCGGATACATGAAGAGTTTAACATCAGCAGATTTCTCACACAACAACTTCTCCGGTTCAGTACCAACTTCAGGGCA : 1820
NM_118146 : ATTCGGATTCTAAACTATCTGAATGTTTCTTGGAATTCCTTTAACCAAAGCCTTCCCAACGAACTCGGATACATGAAGAGTTTAACATCAGCAGATTTCTCACACAACAACTTCTCCGGTTCAGTACCAACTTCAGGGCA : 1820
FJ708747  : ATTCGGATTCTAAACTATCTGAATGTTTCTTGGAATTCCTTTAACCAAAGCCTTCCCAACGAACTCGGATACATGAAGAGTTTAACATCAGCAGATTTCTCACACAACAACTTCTCCGGTTCAGTACCAACTTCAGGGCA : 1733
                                                                                                                                                               
                     *      1840         *      1860         *      1880         *      1900         *      1920         *      1940         *      1960       
Genomic   : ATTCTCTTACTTCAACAACACGTCATTCCTTGGAAACCCTTTTCTCTGTGGATTTTCTTCAAACCCTTGCAACGGTTCCCAAAACCAATCTCAATCTCAGCTACTTAACCAGAACAACGCAAGATCCCGAGGTGAAATCT : 1960
NM_118146 : ATTCTCTTACTTCAACAACACGTCATTCCTTGGAAACCCTTTTCTCTGTGGATTTTCTTCAAACCCTTGCAACGGTTCCCAAAACCAATCTCAATCTCAGCTACTTAACCAGAACAACGCAAGATCCCGAGGTGAAATCT : 1960
FJ708747  : ATTCTCTTACTTCAACAACACGTCATTCCTTGGAAACCCTTTTCTCTGTGGATTTTCTTCAAACCCTTGCAACGGTTCCCAAAACCAATCTCAATCTCAGCTACTTAACCAGAACAACGCAAGATCCCGAGGTGAAATCT : 1873
                                                                                                                                                               
                     *      1980         *      2000         *      2020         *      2040         *      2060         *      2080         *      2100       
Genomic   : CCGCAAAATTCAAGTTGTTCTTCGGGTTAGGCCTACTAGGGTTTTTCTTGGTGTTCGTCGTTTTAGCTGTGGTCAAGAATAGGAGAATGAGAAAGAACAACCCGAATTTATGGAAGCTTATAGGGTTTCAGAAGCTCGGT : 2100
NM_118146 : CCGCAAAATTCAAGTTGTTCTTCGGGTTAGGCCTACTAGGGTTTTTCTTGGTGTTCGTCGTTTTAGCTGTGGTCAAGAATAGGAGAATGAGAAAGAACAACCCGAATTTATGGAAGCTTATAGGGTTTCAGAAGCTCGGT : 2100
FJ708747  : CCGCAAAATTCAAGTTGTTCTTCGGGTTAGGCCTACTAGGGTTTTTCTTGGTGTTCGTCGTTTTAGCTGTGGTCAAGAATAGGAGAATGAGAAAGAACAACCCGAATTTATGGAAGCTTATAGGGTTTCAGAAGCTCGGT : 2013
                                                                                                                                                               
                     *      2120         *      2140         *      2160         *      2180         *      2200         *      2220         *      2240       
Genomic   : TTCAGAAGCGAACACATATTAGAATGTGTTAAAGAGAACCATGTGATTGGGAAAGGCGGACGAGGGATTGTCTACAAAGGGGTAATGCCAAACGGAGAAGAAGTTGCAGTCAAGAAGCTCTTAACCATAACCAAAGGATC : 2240
NM_118146 : TTCAGAAGCGAACACATATTAGAATGTGTTAAAGAGAACCATGTGATTGGGAAAGGCGGACGAGGGATTGTCTACAAAGGGGTAATGCCAAACGGAGAAGAAGTTGCAGTCAAGAAGCTCTTAACCATAACCAAAGGATC : 2240
FJ708747  : TTCAGAAGCGAACACATATTAGAATGTGTTAAAGAGAACCATGTGATTGGGAAAGGCGGAGCAGGGATTGTCTACAAAGGGGTAATGCCAAACGGAGAAGAAGTTGCAGTCAAGAAGCTCTTAACCATAACCAAAGGATC : 2153
                                                                                                                                                               
                     *      2260         *      2280         *      2300         *      2320         *      2340         *      2360         *      2380       
Genomic   : ATCTCATGACAACGGTTTAGCCGCAGAGATTCAGACATTAGGTAGAATCAGACACAGAAACATAGTGAGATTGCTCGCTTTTTGTTCAAACAAAGACGTGAATCTCCTTGTTTACGAGTATATGCCTAATGGTAGCCTCG : 2380
NM_118146 : ATCTCATGACAACGGTTTAGCCGCAGAGATTCAGACATTAGGTAGAATCAGACACAGAAACATAGTGAGATTGCTCGCTTTTTGTTCAAACAAAGACGTGAATCTCCTTGTTTACGAGTATATGCCTAATGGTAGCCTCG : 2380
FJ708747  : ATCTCATGACAACGGTTTAGCCGCAGAGATTCAGACATTAGGTAGAATCAGACACAGAAACATAGTGAGATTGCTCGCTTTTTGTTCAAACAAAGACGTGAATCTCCTTGTTTACGAGTATATGCCTAATGGTAGCCTCG : 2293
     

                     *      2400         *      2420         *      2440         *      2460         *      2480         *      2500         *      2520       
Genomic   : GAGAAGTCTTGCACGGGAAAGCTGGAGTGTTTTTGAAATGGGAAACACGGTTGCAAATAGCGTTGGAAGCGGCTAAGGGGTTGTGTTATCTTCACCATGATTGCTCGCCACTTATAATCCACCGTGATGTGAAGTCAAAC : 2520
NM_118146 : GAGAAGTCTTGCACGGGAAAGCTGGAGTGTTTTTGAAATGGGAAACACGGTTGCAAATAGCGTTGGAAGCGGCTAAGGGGTTGTGTTATCTTCACCATGATTGCTCGCCACTTATAATCCACCGTGATGTGAAGTCAAAC : 2520
FJ708747  : GAGAAGTCTTGCACGGGAAAGCTGGAGTGTTTTTGAAATGGGAAACACGGTTGCAAATAGCGTTGGAAGCGGCTAAGGGGTTGTGTTATCTTCACCATGATTGCTCGCCACTTATAATCCACCGTGATGTGAAGTCAAAC : 2433
                                                                                                                                                               
                     *      2540         *      2560         *      2580         *      2600         *      2620         *      2640         *      2660       
Genomic   : AACATCTTGTTGGGTCCTGAGTTTGAAGCTCATGTTGCTGATTTTGGGCTTGCTAAGTTTATGATGCAAGACAATGGAGCTTCCGAGTGCATGTCCTCGATCGCTGGCTCGTACGGCTACATCGCTCCAGGTACATGACT : 2660
NM_118146 : AACATCTTGTTGGGTCCTGAGTTTGAAGCTCATGTTGCTGATTTTGGGCTTGCTAAGTTTATGATGCAAGACAATGGAGCTTCCGAGTGCATGTCCTCGATCGCTGGCTCGTACGGCTACATCGCTCCAG---------- : 2650
FJ708747  : AACATCTTGTTGGGTCCTGAGTTTGAAGCTCATGTTGCTGATTTTGGGCTTGCTAAGTTTATGATGCAAGACAATGGAGCTTCCGAGTGCATGTCCTCGATCGCTGGCTCGTACGGCTACATCGCTCCAG---------- : 2563
                                                                                                                                                               
                     *      2680         *      2700         *      2720         *      2740         *      2760         *      2780         *      2800       
Genomic   : GATTCCTAATACTACTTTCTTGTATCACACGTAACATTCAAACTTAGGATTTCAGTACTTCTAGTTAAAACGTATGATTGATCTTAGCCCATTTTATCCGTTTCTTGAATGCAGAATATGCATATACACTGAGAATAGAC : 2800
NM_118146 : ------------------------------------------------------------------------------------------------------------------AATATGCATATACACTGAGAATAGAC : 2676
FJ708747  : ------------------------------------------------------------------------------------------------------------------AATATGCATATACACTGAGAATAGAC : 2589
                                                                                                                                                               
                     *      2820         *      2840         *      2860         *      2880         *      2900         *      2920         *      2940       
Genomic   : GAGAAGAGCGATGTGTACAGCTTCGGAGTAGTGTTATTGGAGCTGATTACGGGTCGAAAACCAGTAGATAATTTTGGGGAAGAAGGGATAGACATTGTGCAATGGTCAAAGATCCAAACAAACTGTAACAGACAAGGTGT : 2940
NM_118146 : GAGAAGAGCGATGTGTACAGCTTCGGAGTAGTGTTATTGGAGCTGATTACGGGTCGAAAACCAGTAGATAATTTTGGGGAAGAAGGGATAGACATTGTGCAATGGTCAAAGATCCAAACAAACTGTAACAGACAAGGTGT : 2816
FJ708747  : GAGAAGAGCGATGTGTACAGCTTCGGAGTAGTGTTATTGGAGCTGATTACGGGTCGAAAACCAGTAGATAATTTTGGGGAAGAAGGGATAGACATTGTGCAATGGTCAAAGATCCAAACAAACTGTAACAGACAAGGTGT : 2729
                                                                                                                                                               
                     *      2960         *      2980         *      3000         *      3020         *      3040         *      3060         *      3080       
Genomic   : GGTGAAGATCATTGACCAGAGATTGAGCAATATTCCATTAGCAGAGGCCATGGAACTGTTCTTTGTGGCAATGCTATGTGTGCAAGAACATAGTGTTGAGAGACCGACCATGAGAGAGGTTGTCCAGATGATCTCTCAGG : 3080
NM_118146 : GGTGAAGATCATTGACCAGAGATTGAGCAATATTCCATTAGCAGAGGCCATGGAACTGTTCTTTGTGGCAATGCTATGTGTGCAAGAACATAGTGTTGAGAGACCGACCATGAGAGAGGTTGTCCAGATGATCTCTCAGG : 2956
FJ708747  : GGTGAAGATCATTGACCAGAGATTGAGCAATATTCCATTAGCAGAGGCCATGGAACTGTTCTTTGTGGCAATGCTATGTGTGCAAGAACATAGTGTTGAGAGACCGACCATGAGAGAGGTTGTCCAGATGATCTCTCAGG : 2869
                                       
                     *      3100       
Genomic   : CTAAACAGCCTAATACTTTC : 3100
NM_118146 : CTAAACAGCCTAATACTTTC : 2976
FJ708747  : CTAAACAGCCTAATACTTTC : 2889


At4g20940
                                                                                                                                                               
                     *        20         *        40         *        60         *        80         *       100         *       120         *       140       
Genomic   : ATGGGGCAACTTCCATCACAGGACATCATGGCATTGCTTGAATTCAAGAAAGGCATCAAACATGACCCTACAGGGTTTGTCCTTAATTCATGGAACGATGAGTCTATTGACTTCAATGGTTGTCCTTCTTCATGGAATGG :  140
NM_118212 : ATGGGGCAACTTCCATCACAGGACATCATGGCATTGCTTGAATTCAAGAAAGGCATCAAACATGACCCTACAGGGTTTGTCCTTAATTCATGGAACGATGAGTCTATTGACTTCAATGGTTGTCCTTCTTCATGGAATGG :  140
FJ708750  : ATGGGGCAACTTCCATCACAGGACATCATGGCATTGCTTGAATTCAAGAAAGGCATCAAACATGACCCTACAGGGTTTGTCCTTAATTCATGGAACGATGAGTCTATTGACTTCAATGGTTGTCCTTCTTCATGGAATGG :  140
                                                                                                                                                               
                     *       160         *       180         *       200         *       220         *       240         *       260         *       280       
Genomic   : TATTGTCTGCAATGGCGGTAATGTGGCTGGTGTTGTTTTGGATAATTTGGGTTTGACTGCTGATGCTGATTTCAGTCTGTTCTCCAACTTGACAAAGCTTGTCAAGCTATCTATGTCAAACAATTCCCTTTCTGGTGTGT :  280
NM_118212 : TATTGTCTGCAATGGCGGTAATGTGGCTGGTGTTGTTTTGGATAATTTGGGTTTGACTGCTGATGCTGATTTCAGTCTGTTCTCCAACTTGACAAAGCTTGTCAAGCTATCTATGTCAAACAATTCCCTTTCTGGTGTGT :  280
FJ708750  : TATTGTCTGCAATGGCGGTAATGTGGCTGGTGTTGTTTTGGATAATTTGGGTTTGACTGCTGATGCTGATTTCAGTCTGTTCTCCAACTTGACAAAGCTTGTCAAGCTATCTATGTCAAACAATTCCCTTTCTGGTGTGT :  280
                                                                                                                                                               
                     *       300         *       320         *       340         *       360         *       380         *       400         *       420       
Genomic   : TGCCAAATGATTTAGGCAGTTTCAAAAGCCTCCAGTTCCTGGATTTATCAGATAATCTGTTCTCTTCTTCATTGCCTAAAGAGATTGGTAGATCAGTAAGCTTGAGGAATCTTTCTTTATCTGGTAACAACTTTTCTGGT :  420
NM_118212 : TGCCAAATGATTTAGGCAGTTTCAAAAGCCTCCAGTTCCTGGATTTATCAGATAATCTGTTCTCTTCTTCATTGCCTAAAGAGATTGGTAGATCAGTAAGCTTGAGGAATCTTTCTTTATCTGGTAACAACTTTTCTGGT :  420
FJ708750  : TGCCAAATGATTTAGGCAGTTTCAAAAGCCTCCAGTTCCTGGATTTATCAGATAATCTGTTCTCTTCTTCATTGCCTAAAGAGATTGGTAGATCAGTAAGCTTGAGGAATCTTTCTTTATCTGGTAACAACTTTTCTGGT :  420
                                                                                                                                                               
                     *       440         *       460         *       480         *       500         *       520         *       540         *       560       
Genomic   : GAGATTCCAGAGTCTATGGGTGGTTTGATTTCACTTCAGTCGTTGGATATGAGTAGCAACTCCTTATCTGGACCTTTGCCAAAGTCCTTGACAAGGCTGAACGATCTGCTGTACTTGAATCTGTCTTCTAATGGATTCAC :  560
NM_118212 : GAGATTCCAGAGTCTATGGGTGGTTTGATTTCACTTCAGTCGTTGGATATGAGTAGCAACTCCTTATCTGGACCTTTGCCAAAGTCCTTGACAAGGCTGAACGATCTGCTGTACTTGAATCTGTCTTCTAATGGATTCAC :  560
FJ708750  : GAGATTCCAGAGTCTATGGGTGGTTTGATTTCACTTCAGTCGTTGGATATGAGTAGCAACTCCTTATCTGGACCTTTGCCAAAGTCCTTGACAAGGCTGAACGATCTGCTGTACTTGAATCTGTCTTCTAATGGATTCAC :  560
                                                                                                                                                               
                     *       580         *       600         *       620         *       640         *       660         *       680         *       700       
Genomic   : GGGGAAAATGCCAAGGGGCTTTGAGTTGATTTCAAGTCTTGAAGTGCTTGATTTGCATGGTAATTCAATTGATGGTAATCTTGATGGGGAGTTTTTCCTTTTAACGAATGCGAGCTATGTCGATATCAGTGGAAACAGAT :  700
NM_118212 : GGGGAAAATGCCAAGGGGCTTTGAGTTGATTTCAAGTCTTGAAGTGCTTGATTTGCATGGTAATTCAATTGATGGTAATCTTGATGGGGAGTTTTTCCTTTTAACGAATGCGAGCTATGTCGATATCAGTGGAAACAGAT :  700
FJ708750  : GGGGAAAATGCCAAGGGGCTTTGAGTTGATTTCAAGTCTTGAAGTGCTTGATTTGCATGGTAATTCAATTGATGGTAATCTTGATGGGGAGTTTTTCCTTTTAACGAATGCGAGCTATGTCGATATCAGTGGAAACAGAT :  700
                                                                                                                                                               
                     *       720         *       740         *       760         *       780         *       800         *       820         *       840       
Genomic   : TGGTGACGACGTCTGGGAAACTGTTACCTGGTGTTTCTGAGAGTATCAAGCACTTGAATCTCAGCCATAATCAGCTTGAAGGTTCATTGACTAGTGGGTTTCAGTTGTTTCAGAACTTAAAAGTCTTGGATCTTAGCTAC :  840
NM_118212 : TGGTGACGACGTCTGGGAAACTGTTACCTGGTGTTTCTGAGAGTATCAAGCACTTGAATCTCAGCCATAATCAGCTTGAAGGTTCATTGACTAGTGGGTTTCAGTTGTTTCAGAACTTAAAAGTCTTGGATCTTAGCTAC :  840
FJ708750  : TGGTGACGACGTCTGGGAAACTGTTACCTGGTGTTTCTGAGAGTATCAAGCACTTGAATCTCAGCCATAATCAGCTTGAAGGTTCATTGACTAGTGGGTTTCAGTTGTTTCAGAACTTAAAAGTCTTGGATCTTAGCTAC :  840
                                                                                                                                                               
                     *       860         *       880         *       900         *       920         *       940         *       960         *       980       
Genomic   : AACATGTTATCTGGAGAATTACCAGGTTTCAATTATGTCTATGATCTTGAAGTTCTCAAGCTTAGCAACAACAGATTCTCAGGTTCCCTTCCCAATAATTTGTTAAAAGGTGACTCTTTGCTTTTAACAACGCTGGATTT :  980
NM_118212 : AACATGTTATCTGGAGAATTACCAGGTTTCAATTATGTCTATGATCTTGAAGTTCTCAAGCTTAGCAACAACAGATTCTCAGGTTCCCTTCCCAATAATTTGTTAAAAGGTGACTCTTTGCTTTTAACAACGCTGGATTT :  980
FJ708750  : AACATGTTATCTGGAGAATTACCAGGTTTCAATTATGTCTATGATCTTGAAGTTCTCAAGCTTAGCAACAACAGATTCTCAGGTTCCCTTCCCAATAATTTGTTAAAAGGTGACTCTTTGCTTTTAACAACGCTGGATTT :  980
                                                                                                                                                               
                     *      1000         *      1020         *      1040         *      1060         *      1080         *      1100         *      1120       
Genomic   : AAGTGGCAACAATCTCTCAGGTATGCTCTTGGCTTTATCTTCTACATAGTGGAAATTGTTATATGTTAACGGTGGCACCAAGAAAACTAGAAATCTAATATTTTTTTATTGGTTTTCCTTTTGTGTTTCAGGGCCAGTAA : 1120
NM_118212 : AAGTGGCAACAATCTCTCAGG---------------------------------------------------------------------------------------------------------------GCCAGTAA : 1009
FJ708750  : AAGTGGCAACAATCTCTCAGG---------------------------------------------------------------------------------------------------------------GCCAGTAA : 1009
                                                                                                                                                               
                     *      1140         *      1160         *      1180         *      1200         *      1220         *      1240         *      1260       
Genomic   : GTTCTATCATGTCAACTACTCTTCACACCCTTGATCTTTCTTCAAATTCACTGACCGGGGAGCTTCCTCTCTTGACTGGGGGATGTGTTTTACTCGATCTCTCAAACAACCAGTTTGAAGGAAATCTGACAAGATGGTCA : 1260
NM_118212 : GTTCTATCATGTCAACTACTCTTCACACCCTTGATCTTTCTTCAAATTCACTGACCGGGGAGCTTCCTCTCTTGACTGGGGGATGTGTTTTACTCGATCTCTCAAACAACCAGTTTGAAGGAAATCTGACAAGATGGTCA : 1149
FJ708750  : GTTCTATCATGTCAACTACTCTTCACACCCTTGATCTTTCTTCAAATTCACTGACCGGGGAGCTTCCTCTCTTGACTGGGGGATGTGTTTTACTCGATCTCTCAAACAACCAGTTTGAAGGAAATCTGACAAGATGGTCA : 1149
                                                                                                                                                               
                     *      1280         *      1300         *      1320         *      1340         *      1360         *      1380         *      1400       
Genomic   : AAATGGGAGAATATAGAGTACCTTGATCTAAGCCAGAACCATTTCACTGGGTCGTTCCCAGACGCAACTCCCCAGCTTCTGCGAGCAAATCATCTTAATCTTTCCTACAATAAGCTCACAGGCTCACTTCCAGAACGGAT : 1400
NM_118212 : AAATGGGAGAATATAGAGTACCTTGATCTAAGCCAGAACCATTTCACTGGGTCGTTCCCAGACGCAACTCCCCAGCTTCTGCGAGCAAATCATCTTAATCTTTCCTACAATAAGCTCACAGGCTCACTTCCAGAACGGAT : 1289
FJ708750  : AAATGGGAGAATATAGAGTACCTTGATCTAAGCCAGAACCATTTCACTGGGTCGTTCCCAGACGCAACTCCCCAGCTTCTGCGAGCAAATCATCTTAATCTTTCCTACAATAAGCTCACAGGCTCACTTCCAGAACGGAT : 1289
                                                                                                                                                               
                     *      1420         *      1440         *      1460         *      1480         *      1500         *      1520         *      1540       
Genomic   : TCCAACTCACTATCCGAAGCTTCGGGTACTTGATATAAGTTCTAACAGCTTAGAAGGGCCAATTCCAGGTGCGTTACTATCCATGCCCACTTTGGAGGAAATCCACCTTCAAAACAATGGCATGACAGGTAACATCGGAC : 1540
NM_118212 : TCCAACTCACTATCCGAAGCTTCGGGTACTTGATATAAGTTCTAACAGCTTAGAAGGGCCAATTCCAGGTGCGTTACTATCCATGCCCACTTTGGAGGAAATCCACCTTCAAAACAATGGCATGACAGGTAACATCGGAC : 1429
FJ708750  : TCCAACTCACTATCCGAAGCTTCGGGTACTTGATATAAGTTCTAACAGCTTAGAAGGGCCAATTCCAGGTGCGTTACTATCCATGCCCACTTTGGAGGAAATCCACCTTCAAAACAATGGCATGACAGGTAACATCGGAC : 1429
                                                                                                                                                               
                     *      1560         *      1580         *      1600         *      1620         *      1640         *      1660         *      1680       
Genomic   : CCCTGCCTTCTTCTGGTTCCAGAATCCGTCTTCTTGATCTTTCTCACAACCGGTTTGACGGGGATCTTCCTGGTGTATTCGGGTCTTTAACCAATCTTCAAGTGCTGAATCTCGCAGCAAATAATTTGTCTGGCTCTTTG : 1680
NM_118212 : CCCTGCCTTCTTCTGGTTCCAGAATCCGTCTTCTTGATCTTTCTCACAACCGGTTTGACGGGGATCTTCCTGGTGTATTCGGGTCTTTAACCAATCTTCAAGTGCTGAATCTCGCAGCAAATAATTTGTCTGGCTCTTTG : 1569
FJ708750  : CCCTGCCTTCTTCTGGTTCCAGAATCCGTCTTCTTGATCTTTCTCACAACCGGTTTGACGGGGATCTTCCTGGTGTATTCGGGTCTTTAACCAATCTTCAAGTGCTGAATCTCGCAGCAAATAATTTGTCTGGCTCTTTG : 1569
                                                                                                                                                               
                     *      1700         *      1720         *      1740         *      1760         *      1780         *      1800         *      1820       
Genomic   : CCTAGCTCCATGAATGACATTGTCTCTCTAAGCTCATTAGACGTATCCCAGAATCATTTCACTGGACCACTCCCAAGCAACTTATCTAGCAACATTATGGCCTTCAACGTGTCATATAATGATCTATCAGGGACTGTGCC : 1820
NM_118212 : CCTAGCTCCATGAATGACATTGTCTCTCTAAGCTCATTAGACGTATCCCAGAATCATTTCACTGGACCACTCCCAAGCAACTTATCTAGCAACATTATGGCCTTCAACGTGTCATATAATGATCTATCAGGGACTGTGCC : 1709
FJ708750  : CCTAGCTCCATGAATGACATTGTCTCTCTAAGCTCATTAGACGTATCCCAGAATCATTTCACTGGACCACTCCCAAGCAACTTATCTAGCAACATTATGGCCTTCAACGTGTCATATAATGATCTATCAGGGACTGTGCC : 1709
                                                                                                                                                               
                     *      1840         *      1860         *      1880         *      1900         *      1920         *      1940         *      1960       
Genomic   : AGAGAATCTGAAGAACTTCCCTCCGCCTTCCTTTTATCCTGGAAACAGCAAGCTCGTCTTGCCTGCTGGGTCCCCTGGATCAAGTGCATCAGAAGCTTCCAAGAATAAATCCACGAACAAACTTGTTAAGGTTGTCATAA : 1960
NM_118212 : AGAGAATCTGAAGAACTTCCCTCCGCCTTCCTTTTATCCTGGAAACAGCAAGCTCGTCTTGCCTGCTGGGTCCCCTGGATCAAGTGCATCAGAAGCTTCCAAGAATAAATCCACGAACAAACTTGTTAAGGTTGTCATAA : 1849
FJ708750  : AGAGAATCTGAAGAACTTCCCTCCGCCTTCCTTTTATCCTGGAAACAGCAAGCTCGTCTTGCCTGCTGGGTCCCCTGGATCAAGTGCATCAGAAGCTTCCAAGAATAAATCCACGAACAAACTTGTTAAGGTTGTCATAA : 1849
                                                                                                                                                               
                     *      1980         *      2000         *      2020         *      2040         *      2060         *      2080         *      2100       
Genomic   : TAGTTTCTTGCGCTGTTGCTCTAATTATTCTCATCCTTGTGGCTATCCTCCTTTTCTGCATCTGCAAATCAAGAAGGCGCGAAGAACGTAGTATTACTGGTAAAGAAACTAATAGGCGGGCTCAAACTATCCCCTCAGGC : 2100
NM_118212 : TAGTTTCTTGCGCTGTTGCTCTAATTATTCTCATCCTTGTGGCTATCCTCCTTTTCTGCATCTGCAAATCAAGAAGGCGCGAAGAACGTAGTATTACTGGTAAAGAAACTAATAGGCGGGCTCAAACTATCCCCTCAGGC : 1989
FJ708750  : TAGTTTCTTGCGCTGTTGCTCTAATTATTCTCATCCTTGTGGCTATCCTCCTTTTCTGCATCTGCAAATCAAGAAGGCGCGAAGAACGTAGTATTACTGGTAAAGAAACTAATAGGCGGGCTCAAACTATCCCCTCAGGC : 1989
                                                                                                                                                               
                     *      2120         *      2140         *      2160         *      2180         *      2200         *      2220         *      2240       
Genomic   : AGCGGAGGTGGTATGGTTGTCTCTGCTGAGGATCTGGTTGCTTCAAGAAAAGGCTCATCATCCGAAATCCTCAGTCCAGATGAAAAACTAGCTGTTGCAACCGGCTTCTCTCCTTCTAAGACCAGCAATTTGTCTTGGTC : 2240
NM_118212 : AGCGGAGGTGGTATGGTTGTCTCTGCTGAGGATCTGGTTGCTTCAAGAAAAGGCTCATCATCCGAAATCCTCAGTCCAGATGAAAAACTAGCTGTTGCAACCGGCTTCTCTCCTTCTAAGACCAGCAATTTGTCTTGGTC : 2129
FJ708750  : AGCGGAGGTGGTATGGTTGTCTCTGCTGAGGATCTGGTTGCTTCAAGAAAAGGCTCATCATCCGAAATCCTCAGTCCAGATGAAAAACTAGCTGTTGCAACCGGCTTCTCTCCTTCTAAGACCAGCAATTTGTCTTGGTC : 2129
                                                                                                                                                               
                     *      2260         *      2280         *      2300         *      2320         *      2340         *      2360         *      2380       
Genomic   : ACCTGGCTCAGGAGATTCATTCCCAGCTGATCAGCAACTAGCACGGCTTGATGTGAGGTCACCAGATAGACTTGTGGGAGAGCTGCATTTTCTGGATGATTCAATTAAGTTGACTCCAGAGGAATTATCAAGGGCACCAG : 2380
NM_118212 : ACCTGGCTCAGGAGATTCATTCCCAGCTGATCAGCAACTAGCACGGCTTGATGTGAGGTCACCAGATAGACTTGTGGGAGAGCTGCATTTTCTGGATGATTCAATTAAGTTGACTCCAGAGGAATTATCAAGGGCACCAG : 2269
FJ708750  : ACCTGGCTCAGGAGATTCATTCCCAGCTGATCAGCAACTAGCACGGCTTGATGTGAGGTCACCAGATAGACTTGTGGGAGAGCTGCATTTTCTGGATGATTCAATTAAGTTGACTCCAGAGGAATTATCAAGGGCACCAG : 2269
                                                                                                                                                               
              
                     *      2400         *      2420         *      2440         *      2460         *      2480         *      2500         *      2520       
Genomic   : CTGAAGTTCTGGGAAGAAGTAGCCACGGCACTTCTTACAGGGCAACGCTGGATAATGGAGTGTTTCTAACAGTGAAATGGCTAAGAGAAGGAGTGGCAAAGCAGAGAAAGGAGTTTGCTAAAGAGGTGAAGAAATTTTCT : 2520
NM_118212 : CTGAAGTTCTGGGAAGAAGTAGCCACGGCACTTCTTACAGGGCAACGCTGGATAATGGAGTGTTTCTAACAGTGAAATGGCTAAGAGAAGGAGTGGCAAAGCAGAGAAAGGAGTTTGCTAAAGAGGTGAAGAAATTTTCT : 2409
FJ708750  : CTGAAGTTCTGGGAAGAAGTAGCCACGGCACTTCTTACAGGGCAACGCTGGATAATGGAGTGTTTCTAACAGTGAAATGGCTAAGAGAAGGAGTGGCAAAGCAGAGAAAGGAGTTTGCTAAAGAGGTGAAGAAATTTTCT : 2409
                                                                                                                                                               
                     *      2540         *      2560         *      2580         *      2600         *      2620         *      2640         *      2660       
Genomic   : AACATTAGACATCCTAATGTTGTGACTCTCCGAGGGTACTATTGGGGTCCAACGCAACACGAGAAGCTTATTCTTTCAGATTATATATCGCCTGGAAGCCTTGCCAGCTTCCTTTACGGTAATGCTCCTTTCAATAAACT : 2660
NM_118212 : AACATTAGACATCCTAATGTTGTGACTCTCCGAGG--------------------------------------------------------------------------------------------------------- : 2444
FJ708750  : AACATTAGACATCCTAATGTTGTGACTCTCCGAGGGTACTATTGGGGTCCAACGCAACACGAGAAGCTTATTCTTTCAGATTATATATCGCCTGGAAGCCTTGCCAGCTTCCTTTACG---------------------- : 2527
                                                                                                                                                               
                     *      2680         *      2700         *      2720         *      2740         *      2760         *      2780         *      2800       
Genomic   : TCTCTAAGTATTATATGTATGTGCAGTATTTTTCATCAAGCTTACGGTTCTTGTTTGATGACAGATCGACCGGGCAGGAAAGGCCCTCCCTTAGCCTGGACCCAACGGTTGAAAATCGCAGTTGATGTGGCACGTGGTCT : 2800
NM_118212 : -------------------------------------------------------------------------------------------------------------------------------------------- :    -
FJ708750  : ----------------------------------------------------------------ATCGACCGGGCAGGAAAGGCCCTCCCTTAGCCTGGACCCAACGGTTGAAAATCGCAGTTGATGTGGCACGTGGTCT : 2603
                                                                                                                                                               
                     *      2820         *      2840         *      2860         *      2880         *      2900         *      2920         *      2940       
Genomic   : AAACTACCTCCATTTCGACAGAGCTGTTCCACACGGTAACCTCAAGGCAACAAACATTCTCTTAGATGGAGCAGAGCTAAACGCGCGTGTGGCAGATTACTGCCTCCACCGTCTCATGACACAAGCAGGCACAGTCGAAC : 2940
NM_118212 : ---------------------AGCTGTTCCACACGGTAACCTCAAGGCAACAAACATTCTCTTAGATGGAGCAGAGCTAAACGCGCGTGTGGCAGATTACTGCCTCCACCGTCTCATGACACAAGCAGGCACAGTCGAAC : 2563
FJ708750  : AAACTACCTCCATTTCGACAGAGCTGTTCCACACGGTAACCTCAAGGCAACAAACATTCTCTTAGATGGAGCAGAGCTAAACGCGCGTGTGGCAGATTACTGCCTCCACCGTCTCATGACACAAGCAGGCACAGTCGAAC : 2743
                                                                                                                                                               
                     *      2960         *      2980         *      3000         *      3020         *      3040         *      3060         *      3080       
Genomic   : AGATTCTAGACGCAGGAATCCTCGGTTACCGAGCTCCCGAGCTAGCAGCTTCAAGAAAACCATTACCTTCTTTCAAATCAGATGTATATGCGTTTGGTGTGATACTTCTTGAGATCCTAACCGGAAGATGTGCAGGAGAT : 3080
NM_118212 : AGATTCTAGACGCAGGAATCCTCGGTTACCGAGCTCCCGAGCTAGCAGCTTCAAGAAAACCATTACCTTCTTTCAAATCAGATGTATATGCGTTTGGTGTGATACTTCTTGAGATCCTAACCGGAAGATGTGCAGGAGAT : 2703
FJ708750  : AGATTCTAGACGCAGGAATCCTCGGTTACCGAGCTCCCGAGCTAGCAGCTTCAAGAAAACCATTACCTTCTTTCAAATCAGATGTATATGCGTTTGGTGTGATACTTCTTGAGATCCTAACCGGAAGATGTGCAGGAGAT : 2883
                                                                                                                                                               
                     *      3100         *      3120         *      3140         *      3160         *      3180         *      3200         *      3220       
Genomic   : GTAATCACAGGTGAGCAAGAAGGTGTTGATCTAACCGATTGGGTTAGGTTACGTGTTGCAGAAGGGCGTGGTGCAGAATGCTTTGATTCGGTATTGACTCAGGAGATGGGAAGTGATCCGGTAACAGAAAAAGGCATGAA : 3220
NM_118212 : GTAATCACAGGTGAGCAAGAAGGTGTTGATCTAACCGATTGGGTTAGGTTACGTGTTGCAGAAGGGCGTGGTGCAGAATGCTTTGATTCGGTATTGACTCAGGAGATGGGAAGTGATCCGGTAACAGAAAAAGGCATGAA : 2843
FJ708750  : GTAATCACAGGTGAGCAAGAAGGTGTTGATCTAACCGATTGGGTTAGGTTACGTGTTGCAGAAGGGCGTGGTGCAGAATGCTTTGATTCGGTATTGACTCAGGAGATGGGAAGTGATCCGGTAACAGAAAAAGGCATGAA : 3023
                                                                                                           
                     *      3240         *      3260         *      3280         *      3300               
Genomic   : AGAAGTTCTTGGGATTGCTTTGAGGTGTATAAGATCAGTTTCTGAGAGACCTGGTATTAAGACCATTTACGAAGATCTTTCTTCTATT : 3308
NM_118212 : AGAAGTTCTTGGGATTGCTTTGAGGTGTATAAGATCAGTTTCTGAGAGACCTGGTATTAAGACCATTTACGAAGATCTTTCTTCTATT : 2931
FJ708750  : AGAAGTTCTTGGGATTGCTTTGAGGTGTATAAGATCAGTTTCTGAGAGACCTGGTATTAAGACCATTTACGAAGATCTTTCTTCTATT : 3111


At4g26540
                                                                                                                                                               
                     *        20         *        40         *        60         *        80         *       100         *       120         *       140       
Genomic   : ATGCCACCAAATATCTATAGACTCTCCTTCTTCTCATCCCTACTCTGTTTCTTCTTCATCCCCTGTTTCTCTCTCGACCAACAAGGTCAAGCTCTCTTGTCATGGAAGTCTCAACTGAACATCTCCGGCGACGCTTTTTC :  140
NM_118787 : ATGCCACCAAATATCTATAGACTCTCCTTCTTCTCATCCCTACTCTGTTTCTTCTTCATCCCCTGTTTCTCTCTCGACCAACAAGGTCAAGCTCTCTTGTCATGGAAGTCTCAACTGAACATCTCCGGCGACGCTTTTTC :  140
FJ708754  : ATGCCACCAAATATCTATAGACTCTCCTTCTTCTCATCCCTACTCTGTTTCTTCTTCATCCCCTGTTTCTCTCTCGACCAACAAGGTCAAGCTCTCTTGTCATGGAAGTCTCAACTGAACATCTCCGGCGACGCTTTTTC :  140
                                                                                                                                                               
                     *       160         *       180         *       200         *       220         *       240         *       260         *       280       
Genomic   : CTCCTGGCACGTCGCCGACACATCTCCCTGCAATTGGGTCGGCGTAAAATGTAACCGTAGAGGTGAAGTTTCGGAGATACAGCTCAAAGGTATGGACTTGCAAGGTTCTCTGCCGGTGACTAGTCTCCGGAGCCTCAAGT :  280
NM_118787 : CTCCTGGCACGTCGCCGACACATCTCCCTGCAATTGGGTCGGCGTAAAATGTAACCGTAGAGGTGAAGTTTCGGAGATACAGCTCAAAGGTATGGACTTGCAAGGTTCTCTGCCGGTGACTAGTCTCCGGAGCCTCAAGT :  280
FJ708754  : CTCCTGGCACGTCGCCGACACATCTCCCTGCAATTGGGTCGGCGTAAAATGTAACCGTAGAGGTGAAGTTTCGGAGATACAGCTCAAAGGTATGGACTTGCAAGGTTCTCTGCCGGTGACTAGTCTCCGGAGCCTCAAGT :  280
                                                                                                                                                               
                     *       300         *       320         *       340         *       360         *       380         *       400         *       420       
Genomic   : CTCTTACTTCCCTCACTTTATCTTCACTGAATCTCACCGGAGTAATCCCCAAGGAGATCGGAGACTTCACGGAGCTTGAGTTACTCGATTTGTCGGATAATTCTCTCTCCGGTGATATCCCTGTGGAAATCTTCAGGCTC :  420
NM_118787 : CTCTTACTTCCCTCACTTTATCTTCACTGAATCTCACCGGAGTAATCCCCAAGGAGATCGGAGACTTCACGGAGCTTGAGTTACTCGATTTGTCGGATAATTCTCTCTCCGGTGATATCCCTGTGGAAATCTTCAGGCTC :  420
FJ708754  : CTCTTACTTCCCTCACTTTATCTTCACTGAATCTCACCGGAGTAATCCCCAAGGAGATCGGAGACTTCACGGAGCTTGAGTTACTCGATTTGTCGGATAATTCTCTCTCCGGTGATATCCCTGTGGAAATCTTCAGGCTC :  420
                                                                                                                                                               
                     *       440         *       460         *       480         *       500         *       520         *       540         *       560       
Genomic   : AAGAAACTCAAGACTCTGTCTCTGAACACTAACAATCTAGAAGGTCATATTCCGATGGAGATTGGGAATCTTTCGGGTCTCGTAGAGCTTATGCTTTTCGACAACAAGCTATCCGGAGAGATCCCAAGGAGTATCGGAGA :  560
NM_118787 : AAGAAACTCAAGACTCTGTCTCTGAACACTAACAATCTAGAAGGTCATATTCCGATGGAGATTGGGAATCTTTCGGGTCTCGTAGAGCTTATGCTTTTCGACAACAAGCTATCCGGAGAGATCCCAAGGAGTATCGGAGA :  560
FJ708754  : AAGAAACTCAAGACTCTGTCTCTGAACACTAACAATCTAGAAGGTCATATTCCGATGGAGATTGGGAATCTTTCGGGTCTCGTAGAGCTTATGCTTTTCGACAACAAGCTATCCGGAGAGATCCCAAGGAGTATCGGAGA :  560
                                                                                                                                                               
                     *       580         *       600         *       620         *       640         *       660         *       680         *       700       
Genomic   : GCTCAAGAATCTACAAGTCTTACGTGCTGGTGGGAACAAGAATCTAAGAGGTGAGCTTCCTTGGGAGATAGGTAACTGCGAGAATCTTGTAATGCTTGGTCTCGCTGAGACTAGTCTCTCCGGGAAACTTCCGGCGTCGA :  700
NM_118787 : GCTCAAGAATCTACAAGTCTTACGTGCTGGTGGGAACAAGAATCTAAGAGGTGAGCTTCCTTGGGAGATAGGTAACTGCGAGAATCTTGTAATGCTTGGTCTCGCTGAGACTAGTCTCTCCGGGAAACTTCCGGCGTCGA :  700
FJ708754  : GCTCAAGAATCTACAAGTCTTACGTGCTGGTGGGAACAAGAATCTAAGAGGTGAGCTTCCTTGGGAGATAGGTAACTGCGAGAATCTTGTAATGCTTGGTCTCGCTGAGACTAGTCTCTCCGGGAAACTTCCGGCGTCGA :  700
                                                                                                                                                               
                     *       720         *       740         *       760         *       780         *       800         *       820         *       840       
Genomic   : TTGGAAACCTGAAACGTGTTCAGACAATAGCAATTTACACGTCACTCTTGTCTGGTCCAATTCCGGATGAGATTGGATACTGCACAGAGCTCCAAAACCTCTACTTGTATCAGAATTCAATCTCAGGATCGATCCCTACC :  840
NM_118787 : TTGGAAACCTGAAACGTGTTCAGACAATAGCAATTTACACGTCACTCTTGTCTGGTCCAATTCCGGATGAGATTGGATACTGCACAGAGCTCCAAAACCTCTACTTGTATCAGAATTCAATCTCAGGATCGATCCCTACC :  840
FJ708754  : TTGGAAACCTGAAACGTGTTCAGACAATAGCAATTTACACGTCACTCTTGTCTGGTCCAATTCCGGATGAGATTGGATACTGCACAGAGCTCCAAAACCTCTACTTGTATCAGAATTCAATCTCAGGATCGATCCCTACC :  840
                                                                                                                                                               
                     *       860         *       880         *       900         *       920         *       940         *       960         *       980       
Genomic   : ACCATTGGAGGTTTAAAGAAGCTGCAGAGTCTACTTTTATGGCAGAACAATCTCGTTGGGAAAATCCCAACCGAGCTTGGGAACTGTCCCGAGCTCTGGCTTATCGATTTTTCCGAGAATCTCCTCACCGGGACCATCCC :  980
NM_118787 : ACCATTGGAGGTTTAAAGAAGCTGCAGAGTCTACTTTTATGGCAGAACAATCTCGTTGGGAAAATCCCAACCGAGCTTGGGAACTGTCCCGAGCTCTGGCTTATCGATTTTTCCGAGAATCTCCTCACCGGGACCATCCC :  980
FJ708754  : ACCATTGGAGGTTTAAAGAAGCTGCAGAGTCTACTTTTATGGCAGAACAATCTCGTTGGGAAAATCCCAACCGAGCTTGGGAACTGTCCCGAGCTCTGGCTTATCGATTTTTCCGAGAATCTCCTCACCGGGACCATCCC :  980
                                                                                                                                                               
                     *      1000         *      1020         *      1040         *      1060         *      1080         *      1100         *      1120       
Genomic   : AAGGAGCTTCGGAAAACTTGAGAATCTACAAGAGCTTCAGCTGAGTGTAAACCAGATCTCAGGAACAATCCCTGAAGAGCTAACGAATTGCACGAAGCTGACGCATTTAGAGATCGACAATAATCTCATCACAGGAGAGA : 1120
NM_118787 : AAGGAGCTTCGGAAAACTTGAGAATCTACAAGAGCTTCAGCTGAGTGTAAACCAGATCTCAGGAACAATCCCTGAAGAGCTAACGAATTGCACGAAGCTGACGCATTTAGAGATCGACAATAATCTCATCACAGGAGAGA : 1120
FJ708754  : AAGGAGCTTCGGAAAACTTGAGAATCTACAAGAGCTTCAGCTGAGTGTAAACCAGATCTCAGGAACAATCCCTGAAGAGCTAACGAATTGCACGAAGCTGACGCATTTAGAGATCGACAATAATCTCATCACAGGAGAGA : 1120
                                                                                                                                                               
                     *      1140         *      1160         *      1180         *      1200         *      1220         *      1240         *      1260       
Genomic   : TTCCGTCATTGATGAGTAACCTAAGAAGCTTAACCATGTTCTTCGCATGGCAGAACAAGTTAACTGGAAACATCCCTCAAAGTCTCTCCCAGTGTCGTGAGCTTCAAGCCATCGATCTCTCTTACAACAGTCTCTCTGGC : 1260
NM_118787 : TTCCGTCATTGATGAGTAACCTAAGAAGCTTAACCATGTTCTTCGCATGGCAGAACAAGTTAACTGGAAACATCCCTCAAAGTCTCTCCCAGTGTCGTGAGCTTCAAGCCATCGATCTCTCTTACAACAGTCTCTCTGGC : 1260
FJ708754  : TTCCGTCATTGATGAGTAACCTAAGAAGCTTAACCATGTTCTTCGCATGGCAGAACAAGTTAACTGGAAACATCCCTCAAAGTCTCTCCCAGTGTCGTGAGCTTCAAGCCATCGATCTCTCTTACAACAGTCTCTCTGGC : 1260
                                                                                                                                                               
                     *      1280         *      1300         *      1320         *      1340         *      1360         *      1380         *      1400       
Genomic   : TCCATCCCAAAAGAGATATTCGGGTTACGAAACCTCACGAAGCTTCTTCTTCTCTCCAATGATTTGTCGGGTTTCATACCTCCGGATATTGGAAACTGTACGAATCTTTACCGGTTAAGACTCAACGGTAACAGACTCGC : 1400
NM_118787 : TCCATCCCAAAAGAGATATTCGGGTTACGAAACCTCACGAAGCTTCTTCTTCTCTCCAATGATTTGTCGGGTTTCATACCTCCGGATATTGGAAACTGTACGAATCTTTACCGGTTAAGACTCAACGGTAACAGACTCGC : 1400
FJ708754  : TCCATCCCAAAAGAGATATTCGGGTTACGAAACCTCACGAAGCTTCTTCTTCTCTCCAATGATTTGTCGGGTTTCATACCTCCGGATATTGGAAACTGTACGAATCTTTACCGGTTAAGACTCAACGGTAACAGACTCGC : 1400
                                                                                                                                                               
                     *      1420         *      1440         *      1460         *      1480         *      1500         *      1520         *      1540       
Genomic   : CGGAAGTATCC-GTCGGAAATCGGGAATCTGAAAAATCTCAACTTTGTTGATATAAGTGAGAACCGTCTCGTCGGGTCAATCC-TCCGGCGATCTCTGGTTGTGAAAGCCTCGAGTTTCTCGATCTTCACACGAATAGTC : 1538
NM_118787 : CGGAAGTATCC---CGGAAATCGGGAATCTGAAAAATCTCAACTTTGTTGATATAAGTGAGAACCGTCTCGTCGGGTCAATCC---CGGCGATCTCTGGTTGTGAAAGCCTCGAGTTTCTCGATCTTCACACGAATAGTC : 1534
FJ708754  : CGGAAGTATCCCGTCGGAAATCGGGAATCTGAAAAATCTCAACTTTGTTGATATAAGTGAGAACCGTCTCGTCGGGTCAATCCCTCCGGCGATCTCTGGTTGTGAAAGCCTCGAGTTTCTCGATCTTCACACGAATAGTC : 1540
                                                                                                                                                               
                     *      1560         *      1580         *      1600         *      1620         *      1640         *      1660         *      1680       
Genomic   : TCTCCGGTTCATTGCTCGGTACTACACTCCCAAAGAGCTTGAAGTTCATCGATTTCTCCGATAATGCTCTGTCCAGTACTCTGCCTCCGGGAATAGGATTGTTGACAGAGCTTACAAAGCTCAATCTCGCCAAGAACAGA : 1678
NM_118787 : TCTCCGGTTCATTGCTCGGTACTACACTCCCAAAGAGCTTGAAGTTCATCGATTTCTCCGATAATGCTCTGTCCAGTACTCTGCCTCCGGGAATAGGATTGTTGACAGAGCTTACAAAGCTCAATCTCGCCAAGAACAGA : 1674
FJ708754  : TCTCCGGTTCATTGCTCGGTACTACACTCCCAAAGAGCTTGAAGTTCATCGATTTCTCCGATAATGCTCTGTCCAGTACTCTGCCTCCGGGAATAGGATTGTTGACAGAGCTTACAAAGCTCAATCTCGCCAAGAACAGA : 1680
                                                                                                                                                               
                     *      1700         *      1720         *      1740         *      1760         *      1780         *      1800         *      1820       
Genomic   : TTATCCGGCGAAATCCCTAGAGAAATCTCCACTTGTCGGAGTCTACAGCTTCTCAATCTCGGCGAAAATGACTTTTCTGGTGAAATCCCTGACGAATTGGGTCAAATCCCGTCTCTGGCGATATCTCTTAATCTCAGCTG : 1818
NM_118787 : TTATCCGGCGAAATCCCTAGAGAAATCTCCACTTGTCGGAGTCTACAGCTTCTCAATCTCGGCGAAAATGACTTTTCTGGTGAAATCCCTGACGAATTGGGTCAAATCCCGTCTCTGGCGATATCTCTTAATCTCAGCTG : 1814
FJ708754  : TTATCCGGCGAAATCCCTAGAGAAATCTCCACTTGTCGGAGTCTACAGCTTCTCAATCTCGGCGAAAATGACTTTTCTGGTGAAATCCCTGACGAATTGGGTCAAATCCCGTCTCTGGCGATATCTCTTAATCTCAGCTG : 1820
                                                                                                                                                               
                     *      1840         *      1860         *      1880         *      1900         *      1920         *      1940         *      1960       
Genomic   : CAATAGGTTCGTCGGAGAAATCCCGTCTAGATTCTCCGACTTGAAAAACCTTGGAGTGCTCGACGTATCTCACAACCAACTCACCGGAAACTTAAACGTTTTAACGGATTTGCAAAACCTCGTCTCTCTCAACATCTCCT : 1958
NM_118787 : CAATAGGTTCGTCGGAGAAATCCCGTCTAGATTCTCCGACTTGAAAAACCTTGGAGTGCTCGACGTATCTCACAACCAACTCACCGGAAACTTAAACGTTTTAACGGATTTGCAAAACCTCGTCTCTCTCAACATCTCCT : 1954
FJ708754  : CAATAGGTTCGTCGGAGAAATCCCGTCTAGATTCTCCGACTTGAAAAACCTTGGAGTGCTCGACGTATCTCACAACCAACTCACCGGAAACTTAAACGTTTTAACGGATTTGCAAAACCTCGTCTCTCTCAACATCTCCT : 1960
                                                                                                                                                               
                     *      1980         *      2000         *      2020         *      2040         *      2060         *      2080         *      2100       
Genomic   : ACAACGATTTCTCCGGCGATTTACCCAACACGCCCTTTTTCCGGCGACTTCCACTTTCTGATCTCGCCTCAAACAGAGGGCTTTACATCTCGAACGCAATCTCGACCCGACCCGACCCGACGACCAGGAACAGCTCCGTC : 2098
NM_118787 : ACAACGATTTCTCCGGCGATTTACCCAACACGCCCTTTTTCCGGCGACTTCCACTTTCTGATCTCGCCTCAAACAGAGGGCTTTACATCTCGAACGCAATCTCGACCCGACCCGACCCGACGACCAGGAACAGCTCCGTC : 2094
FJ708754  : ACAACGATTTCTCCGGCGATTTACCCAACACGCCCTTTTTCCGGCGACTTCCACTTTCTGATCTCGCCTCAAACAGAGGGCTTTACATCTCGAACGCAATCTCGACCCGACCCGACCCGACGACCAGGAACAGCTCCGTC : 2100
                                                                                                                                                               
                     *      2120         *      2140         *      2160         *      2180         *      2200         *      2220         *      2240       
Genomic   : GTTAGATTGACTATTTTAATTCTCGTCGTCGTCACTGCCGTACTTGTTCTCATGGCGGTTTACACTCTAGTCCGGGCAAGAGCCGCCGGAAAACAACTCCTCGGAGAAGAAATAGATTCGTGGGAAGTGACGCTTTACCA : 2238
NM_118787 : GTTAGATTGACTATTTTAATTCTCGTCGTCGTCACTGCCGTACTTGTTCTCATGGCGGTTTACACTCTAGTCCGGGCAAGAGCCGCCGGAAAACAACTCCTCGGAGAAGAAATAGATTCGTGGGAAGTGACGCTTTACCA : 2234
FJ708754  : GTTAGATTGACTATTTTAATTCTCGTCGTCGTCACTGCCGTACTTGTTCTCATGGCGGTTTACACTCTAGTCCGGGCAAGAGCCGCCGGAAAACAACTCCTCGGAGAAGAAATAGATTCGTGGGAAGTGACGCTTTACCA : 2240
                                                                                                                                                               
                     *      2260         *      2280         *      2300         *      2320         *      2340         *      2360         *      2380       
Genomic   : AAAACTCGATTTTTCAATCGACGACATTGTTAAGAATCTGACGTCAGCAAACGTGATCGGAACCGGAAGCTCCGGTGTGGTTTACCGTATAACGATTCCCTCCGGCGAGTCACTCGCGGTGAAGAAAATGTGGTCAAAGG : 2378
NM_118787 : AAAACTCGATTTTTCAATCGACGACATTGTTAAGAATCTGACGTCAGCAAACGTGATCGGAACCGGAAGCTCCGGTGTGGTTTACCGTATAACGATTCCCTCCGGCGAGTCACTCGCGGTGAAGAAAATGTGGTCAAAGG : 2374
FJ708754  : AAAACTCGATTTTTCAATCGACGACATTGTTAAGAATCTGACGTCAGCAAACGTGATCGGAACCGGAAGCTCCGGTGTGGTTTACCGTATAACGATTCCCTCCGGCGAGTCACTCGCGGTGAAGAAAATGTGGTCAAAGG : 2380
              
                                                                                                                                                 
                     *      2400         *      2420         *      2440         *      2460         *      2480         *      2500         *      2520       
Genomic   : AAGAGAGTGGTGCGTTTAACTCCGAGATTAAAACTCTAGGATCGATTCGTCACAGAAACATCGTTCGTCTTCTTGGTTGGTGCTCTAATCGGAATCTGAAACTCTTGTTCTACGACTATCTCCCTAACGGTAGCTTGAGT : 2518
NM_118787 : AAGAGAGTGGTGCGTTTAACTCCGAGATTAAAACTCTAGGATCGATTCGTCACAGAAACATCGTTCGTCTTCTTGGTTGGTGCTCTAATCGGAATCTGAAACTCTTGTTCTACGACTATCTCCCTAACGGTAGCTTGAGT : 2514
FJ708754  : AAGAGAGTGGTGCGTTTAACTCCGAGATTAAAACTCTAGGATCGATTCGTCACAGAAACATCGTTCGTCTTCTTGGTTGGTGCTCTAATCGGAATCTGAAACTCTTGTTCTACGACTATCTCCCTAACGGTAGCTTGAGT : 2520
                                                                                                                                                               
                     *      2540         *      2560         *      2580         *      2600         *      2620         *      2640         *      2660       
Genomic   : TCTCGGCTTCACGGCGCCGGAAAAGGAGGATGTGTTGATTGGGAGGCGCGATACGACGTCGTTCTTGGTGTGGCCCATGCACTTGCTTATCTCCACCATGATTGTCTCCCCACAATTATACATGGTGACGTTAAAGCTAT : 2658
NM_118787 : TCTCGGCTTCACGGCGCCGGAAAAGGAGGATGTGTTGATTGGGAGGCGCGATACGACGTCGTTCTTGGTGTGGCCCATGCACTTGCTTATCTCCACCATGATTGTCTCCCCACAATTATACATGGTGACGTTAAAGCTAT : 2654
FJ708754  : TCTCGGCTTCACGGCGCCGGAAAAGGAGGATGTGTTGATTGGGAGGCGCGATACGACGTCGTTCTTGGTGTGGCCCATGCACTTGCTTATCTCCACCATGATTGTCTCCCCACAATTATACATGGTGACGTTAAAGCTAT : 2660
                                                                                                                                                               
                     *      2680         *      2700         *      2720         *      2740         *      2760         *      2780         *      2800       
Genomic   : GAATGTCTTATTGGGTCCTCACTTTGAACCCTACTTAGCTGATTTCGGTTTAGCTAGAACCATCTCCGGTTATCCAAATACCGGAATCGATTTAGCGAAACCCACCAATCGGCCTCCGATGGCTGGCTCCTTATGGTTAC : 2798
NM_118787 : GAATGTCTTATTGGGTCCTCACTTTGAACCCTACTTAGCTGATTTCGGTTTAGCTAGAACCATCTCCGGTTATCCAAATACCGGAATCGATTTAGCGAAACCCACCAATCGGCCTCCGATGGCTGGCTCCT-ATGGTTAC : 2793
FJ708754  : GAATGTCTTATTGGGTCCTCACTTTGAACCCTACTTAGCTGATTTCGGTTTAGCTAGAACCATCTCCGGTTATCCAAATACCGGAATCGATTTAGCGAAACCCACCAATCGGCCTCCGATGGCTGGCTCCT-ATGGTTAC : 2799
                                                                                                                                                               
                     *      2820         *      2840         *      2860         *      2880         *      2900         *      2920         *      2940       
Genomic   : ATGGCTCCAGGTAAGCTTGAGAAAGTACTTGTACACTTAACTAAGTTTGGAGTTTTGATTTTGATTTGTTTTGTTTGTTGGGTTTTACAGAACATGCTTCGATGCAACGTATTACCGAGAAGAGCGATGTTTATAGTTAC : 2938
NM_118787 : ATGGCTCCAG--------------------------------------------------------------------------------AACATGCTTCGATGCAACGTATTACCGAGAAGAGCGATGTTTATAGTTAC : 2853
FJ708754  : ATGGCTCCAG--------------------------------------------------------------------------------AACATGCTTCGATGCAACGTATTACCGAGAAGAGCGATGTTTATAGTTAC : 2859
                                                                                                                                                               
                     *      2960         *      2980         *      3000         *      3020         *      3040         *      3060         *      3080       
Genomic   : GGGGTGGTATTGTTAGAGGTTTTAACCGGAAAGCATCCGTTAGATCCGGATCTACCAGGAGGTGCACATTTGGTTAAGTGGGTGAGAGATCACTTGGCTGAGAAAAAAGATCCTAGCAGGCTTCTTGATCCGAGACTCGA : 3078
NM_118787 : GGGGTGGTATTGTTAGAGGTTTTAACCGGAAAGCATCCGTTAGATCCGGATCTACCAGGAGGTGCACATTTGGTTAAGTGGGTGAGAGATCACTTGGCTGAGAAAAAAGATCCTAGCAGGCTTCTTGATCCGAGACTCGA : 2993
FJ708754  : GGGGTGGTATTGTTAGAGGTTTTAACCGGAAAGCATCCGTTAGATCCGGATCTACCAGGAGGTGCACATTTGGTTAAGTGGGTGAGAGATCACTTGGCTGAGAAAAAAGATCCTAGCAGGCTTCTTGATCCGAGACTCGA : 2999
                                                                                                                                                               
                     *      3100         *      3120         *      3140         *      3160         *      3180         *      3200         *      3220       
Genomic   : TGGACGGACTGACTCGATAATGCATGAGATGCTTCAAACTCTTGCGGTTGCGTTCTTGTGTGTTAGCAACAAGGCAAACGAACGACCTTTAATGAAAGATGTTGTGGCAATGCTCACAGAAATAAGGCATATTGATGTTG : 3218
NM_118787 : TGGACGGACTGACTCGATAATGCATGAGATGCTTCAAACTCTTGCGGTTGCGTTCTTGTGTGTTAGCAACAAGGCAAACGAACGACCTTTAATGAAAGATGTTGTGGCAATGCTCACAGAAATAAGGCATATTGATGTTG : 3133
FJ708754  : TGGACGGACTGACTCGATAATGCATGAGATGCTTCAAACTCTTGCGGTTGCGTTCTTGTGTGTTAGCAACAAGGCAAACGAACGACCTTTAATGAAAGATGTTGTGGCAATGCTCACAGAAATAAGGCATATTGATGTTG : 3139
                                                                                                                                                         
                     *      3240         *      3260         *      3280         *      3300         *      3320         *      3340         *           
Genomic   : GAAGATCGGAGACAGAGAAGATCAAAGCTGGTGGTTGCGGAAGCAAAGAGCCACAACAATTTATGTCGAATGAGAAAATCATCAATTCTCACGGTTCGTCTAATTGTTCATTTGCGTTTTCGGATGACTCCGTT : 3352
NM_118787 : GAAGATCGGAGACAGAGAAGATCAAAGCTGGTGGTTGCGGAAGCAAAGAGCCACAACAATTTATGTCGAATGAGAAAATCATCAATTCTCACGGTTCGTCTAATTGTTCATTTGCGTTTTCGGATGACTCCGTT : 3267
FJ708754  : GAAGATCGGAGACAGAGAAGATCAAAGCTGGTGGTTGCGGAAGCAAAGAGCCACAACAATTTATGTCGAATGAGAAAATCATCAATTCTCACGGTTCGTCTAATTGTTCATTTGCGTTTTCGGATGACTCCGTT : 3273


At4g29180
                                                                                                                                                               
                     *        20         *        40         *        60         *        80         *       100         *       120         *       140       
Genomic   : ATGGGAGCTCACTCTGTTTTTCTCATCCTTTTTTCCGTCATTGCCATTGCCATTGTAGTACATGGCCAAGGCCAAGCAGGTTCGTGAGTCTCATTCTCGCTTATTTTCCTCTGTTAGAGTCTGTTTTGATTTGAAAATGA :  140
NM_119062 : ATGGGAGCTCACTCTGTTTTTCTCATCCTTTTTTCCGTCATTGCCATTGCCATTGTAGTACATGGCCAAGGCCAAG---------------------------------------------------------------- :   76
FJ708754  : ATGGGAGCTC-CTCTGTTTTTCTCATCCTTTTTTCCGTCATTGCCATTGCCATTGTAGTACATGGCCAAGGCCAAG---------------------------------------------------------------- :   76
                                                                                                                                                               
                     *       160         *       180         *       200         *       220         *       240         *       260         *       280       
Genomic   : GCAATCTTCAGACCAAAGATTTTTGAAGTTTTGATATATGTGGTTGCATTTTGGCCAAAACCGTGTTCTGATTATTGGTTTGACATGATAAGTGAGGTCCAAGTCTGTTTCTATGATTGAATGCTTTAGGTTCTAAAATT :  280
NM_119062 : -------------------------------------------------------------------------------------------------------------------------------------------- :    -
FJ708757  : -------------------------------------------------------------------------------------------------------------------------------------------- :    -
                                                                                                                                                               
                     *       300         *       320         *       340         *       360         *       380         *       400         *       420       
Genomic   : TAAACCTGCATTGGAATTTTGATGAAAATTTTCTCAACGTTGTAGAATTAATCAAAACCCTTACCTTCTTCTGGTCCAGGTTTCATCAGCATAGATTGTGGGTCACCCCCTAATATTAACTATGTAGACACTGATACTGG :  420
NM_119062 : ----------------------------------------------------------------------------CAGGTTTCATCAGCATAGATTGTGGGTCACCCCCTAATATTAACTATGTAGACACTGATACTGG :  140
FJ708757  : ----------------------------------------------------------------------------CAGGTTTCATCAGCATAGATTGTGGGTCACCCCCTAATATTAACTATGTAGACACTGATACTGG :  140
                                                                                                                                                               
                     *       440         *       460         *       480         *       500         *       520         *       540         *       560       
Genomic   : TATCAGTTACACCTGGGATGCACCTTTCATCAACGCTGGAGTAAATCTGAACGTCTCTGAAGAATACGGTTACCCGAAAAACCCGGTTTTGCCTTTCCCGCTTGCTGATGTAAGATCTTTTCCTCAAGGAAACAGGAACT :  560
NM_119062 : TATCAGTTACACCTGGGATGCACCTTTCATCAACGCTGGAGTAAATCTGAACGTCTCTGAAGAATACGGTTACCCGAAAAACCCGGTTTTGCCTTTCCCGCTTGCTGATGTAAGATCTTTTCCTCAAGGAAACAGGAACT :  280
FJ708757  : TATCAGTTACACCTGGGATGCACCTTTCATCAACGCTGGAGTAAATCTGAACGTCTCTGAAGAATACGGTTACCCGAAAAACCCGGTTTTGCCTTTCCCGCTTGCTGATGTAAGATCTTTTCCTCAAGGAAACAGGAACT :  280
                                                                                                                                                               
                     *       580         *       600         *       620         *       640         *       660         *       680         *       700       
Genomic   : GTTACACCTTAACCCCCTCCGATGGAAAAGGGAATCTTTATCTCATCAGAGCTTCGTTTATGTATGGAAACTACGATGGTAAAAATGCTTTACCTGAGTTTGATCTCTACGTAAACGTGAATTTCTGGACCTCGGTGAAA :  700
NM_119062 : GTTACACCTTAACCCCCTCCGATGGAAAAGGGAATCTTTATCTCATCAGAGCTTCGTTTATGTATGGAAACTACGATGGTAAAAATGCTTTACCTGAGTTTGATCTCTACGTAAACGTGAATTTCTGGACCTCGGTGAAA :  420
FJ708757  : GTTACACCTTAACCCCCTCCGATGGAAAAGGGAATCTTTATCTCATCAGAGCTTCGTTTATGTATGGAAACTACGATGGTAAAAATGCTTTACCTGAGTTTGATCTCTACGTAAACGTGAATTTCTGGACCTCGGTGAAA :  420
                                                                                                                                                               
                     *       720         *       740         *       760         *       780         *       800         *       820         *       840       
Genomic   : CTCAGAAATGCTTCTGAGAACGTCATCAAGGAGATCCTCAGCTTTGCAGAGTCAGATACAATATATGTTTGCCTTGTGAACAAAGGTAAAGGAACTCCTTTTATTTCAGCGTTGGAGCTTCGACCAATGAACAGCTCCAT :  840
NM_119062 : CTCAGAAATGCTTCTGAGAACGTCATCAAGGAGATCCTCAGCTTTGCAGAGTCAGATACAATATATGTTTGCCTTGTGAACAAAGGTAAAGGAACTCCTTTTATTTCAGCGTTGGAGCTTCGACCAATGAACAGCTCCAT :  560
FJ708757  : CTCAGAAATGCTTCTGAGAACGTCATCAAGGAGATCCTCAGCTTTGCAGAGTCAGATACAATATATGTTTGCCTTGTGAACAAAGGTAAAGGAACTCCTTTTATTTCAGCGTTGGAGCTTCGACCAATGAACAGCTCCAT :  560
                                                                                                                                                               
                     *       860         *       880         *       900         *       920         *       940         *       960         *       980       
Genomic   : CTATGGAACTGAGTTTGGAAGAAATGTCTCATTGGTACTCTATCAAAGATGGGATACAGGATATTTGAATGGAACAGGACGGTACCAAAAGGATACATATGATCGTATTTGGTCTCCGTACTCTCCAGTGTCTTGGAATA :  980
NM_119062 : CTATGGAACTGAGTTTGGAAGAAATGTCTCATTGGTACTCTATCAAAGATGGGATACAGGATATTTGAATGGAACAGGACGGTACCAAAAGGATACATATGATCGTATTTGGTCTCCGTACTCTCCAGTGTCTTGGAATA :  700
FJ708757  : CTATGGAACTGAGTTTGGAAGAAATGTCTCATTGGTACTCTATCAAAGATGGGATACAGGATATTTGAATGGAACAGGACGGTACCAAAAGGATACATATGATCGTATTTGGTCTCCGTACTCTCCAGTGTCTTGGAATA :  700
                                                                                                                                                               
                     *      1000         *      1020         *      1040         *      1060         *      1080         *      1100         *      1120       
Genomic   : CAACTATGACTACTGGGTATATCGATATTTTTCAGTCTGGTTATAGGCCACCAGATGAAGTTATTAAGACAGCTGCTTCGCCTAAAAGTGACGATGAACCATTGGAACTGTCTTGGACATCAAGCGATCCAGATACTCGG : 1120
NM_119062 : CAACTATGACTACTGGGTATATCGATATTTTTCAGTCTGGTTATAGGCCACCAGATGAAGTTATTAAGACAGCTGCTTCGCCTAAAAGTGACGATGAACCATTGGAACTGTCTTGGACATCAAGCGATCCAGATACTCGG :  840
FJ708757  : CAACTATGACTACTGGGTATATCGATATTTTTCAGTCTGGTTATAGGCCACCAGATGAAGTTATTAAGACAGCTGCTTCGCCTAAAAGTGACGATGAACCATTGGAACTGTCTTGGACATCAAGCGATCCAGATACTCGG :  840
                                                                                                                                                               
                     *      1140         *      1160         *      1180         *      1200         *      1220         *      1240         *      1260       
Genomic   : TTTTATGCGTACCTTTATTTTGCAGAACTTGAAAATCTCAAGAGGAATGAGAGCAGAGAAATCAAGATATTCTGGAATGGGTCGCCTGTTTCAGGAGCTTTTAACCCCTCTCCTGAGTATTCAATGACAGTGTCTAACTC : 1260
NM_119062 : TTTTATGCGTACCTTTATTTTGCAGAACTTGAAAATCTCAAGAGGAATGAGAGCAGAGAAATCAAGATATTCTGGAATGGGTCGCCTGTTTCAGGAGCTTTTAACCCCTCTCCTGAGTATTCAATGACAGTGTCTAACTC :  980
FJ708757  : TTTTATGCGTACCTTTATTTTGCAGAACTTGAAAATCTCAAGAGGAATGAGAGCAGAGAAATCAAGATATTCTGGAATGGGTCGCCTGTTTCAGGAGCTTTTAACCCCTCTCCTGAGTATTCAATGACAGTGTCTAACTC :  980
                                                                                                                                                               
                     *      1280         *      1300         *      1320         *      1340         *      1360         *      1380         *      1400       
Genomic   : ACGAGCTTTCACTGGTAAAGATCACTGGATTTCTGTTCAGAAGACCGCAGAGTCAACGCGTCCACCAATACTCAATGCTATTGAGATTTTTTCAGCACAATCTCTGGATGAATTTTACACCAGAATTGACGATGGTGTGT : 1400
NM_119062 : ACGAGCTTTCACTGGTAAAGATCACTGGATTTCTGTTCAGAAGACCGCAGAGTCAACGCGTCCACCAATACTCAATGCTATTGAGATTTTTTCAGCACAATCTCTGGATGAATTTTACACCAGAATTGACGATG------ : 1114
FJ708757  : ACGAGCTTTCACTGGTAAAGATCACTGGATTTCTGTTCAGAAGACCGCAGAGTCAACGCGTCCACCAATACTCAATGCTATTGAGATTTTTTCAGCACAATCTCTGGATGAATTTTACACCAGAATTGACGATG------ : 1114
                                                                                                                                                               
                     *      1420         *      1440         *      1460         *      1480         *      1500         *      1520         *      1540       
Genomic   : TCATCTCTACTTTGTTAGTCGTCTACGTATATTTTGTTCTGGTCTTTAATTTTCACTGACACTCACAGTTTTCCATTTTCTATTGGTAATGCAGTTCAAGCCATAGAAAGCATAAAATCAACGTATAAAGTGAATAAGAT : 1540
NM_119062 : ----------------------------------------------------------------------------------------------TTCAAGCCATAGAAAGCATAAAATCAACGTATAAAGTGAATAAGAT : 1160
FJ708757  : ----------------------------------------------------------------------------------------------TTCAAGCCATAGAAAGCATAAAATCAACGTATAAAGTGAATAAGAT : 1160
                                                                                                                                                               
                     *      1560         *      1580         *      1600         *      1620         *      1640         *      1660         *      1680       
Genomic   : TTGGACTGGTGATCCTTGCTCTCCCAGACTCTTCCCTTGGGAAGGTATTGGATGCAGCTACAACACTTCTAGTTATCAAATCAAGTCCCTGTAAGTCATTCATTCATTTCAACCTGAAAAAACTCATTGTGTAATTTCCC : 1680
NM_119062 : TTGGACTGGTGATCCTTGCTCTCCCAGACTCTTCCCTTGGGAAG------------------------------------------------------------------------------------------------ : 1204
FJ708757  : TTGGACTGGTGATCCTTGCTCTCCCAGACTCTTCCCTTGGGAAGGTATTGGATGCAGCTACAACACTTCTAGTTATCAAATCAAGTCCCT-------------------------------------------------- : 1250
                                                                                                                                                               
                     *      1700         *      1720         *      1740         *      1760         *      1780         *      1800         *      1820       
Genomic   : TCAAACTTAAGTTTTGCTGATGAGCTTGTTTCTGTATTTTGCTGCGAGAAGAAATCTTAGTTCAAGTGGATTACATGGACCAATAGCGTTCGCATTTAGAAATCTCTCCCTTCTTGAGTCATTGTAAGTATACAAACACA : 1820
NM_119062 : -----------TTTTGCTGATGAGCTTGTTTCTGTATTTTGCTGCGAGAAGAAATCTTAGTTCAAGTGGATTACATGGACCAATAGCGTTCGCATTTAGAAATCTCTCCCTTCTTGAGTCATTG---------------- : 1317
FJ708757  : ---------------------------------------------------AAATCTTAGTTCAAGTGGATTACATGGACCAATAGCGTTCGCATTTAGAAATCTCTCCCTTCTTGAGTCATTG---------------- : 1323
                                                                                                                                                               
                     *      1840         *      1860         *      1880         *      1900         *      1920         *      1940         *      1960       
Genomic   : TTGAAACTTGCCACCACTTTATTCACAGTAACTATAATAGTGGTGTGCATTGTTTTCCCAGGGATTTATCAAACAACAACCTCAAAGGAATTGTGCCGGAATTTTTGGCTGATCTAAAATATCTGAAGTCCCTGTGAGTT : 1960
NM_119062 : --------------------------------------------------------------GATTTATCAAACAACAACCTCAAAGGAATTGTGCCGGAATTTTTGGCTGATCTAAAATATCTGAAGTCCCTG------ : 1389
FJ708757  : --------------------------------------------------------------GATTTATCAAACAACAACCTCAAAGGAATTGTGCCGGAATTTTTGGCTGATCTAAAATATCTGAAGTCCCTG------ : 1395
                                                                                                                                                               
                     *      1980         *      2000         *      2020         *      2040         *      2060         *      2080         *      2100       
Genomic   : GAGCACATAGAGAACCTCGTTGAGTTTGTATTGATAGACAATCAAAATAGTATTTTGTCTCTGACATTAATTTGTGTCGCAGGAATTTGAAAGGAAATAACTTGACTGGGTTTATCCCAAGATCTCTTAGGAAAAGAGCA : 2100
NM_119062 : -----------------------------------------------------------------------------------AATTTGAAAGGAAATAACTTGACTGGGTTTATCCCAAGATCTCTTAGGAAAAGAGCA : 1446
FJ708757  : -----------------------------------------------------------------------------------AATTTGAAAGGAAATAACTTGACTGGGTTTATCCCAAGATCTCTTAGGAAAAGAGCA : 1452
                                                                                                                                                               
                     *      2120         *      2140         *      2160         *      2180         *      2200         *      2220         *      2240       
Genomic   : ACGGCTAATGGACTTGCACTCAGGTACACGGAACCATAGTCATTTTGCCGCGTCTCTACCATTTTCAAAAGCTTATTGATTTCCTCTTTGTCTCCAGTGTCGATGAACAAAACATTTGTCACTCACGTTCATGTCGGGAC : 2240
NM_119062 : ACGGCTAATGGACTTGCACTCAG--------------------------------------------------------------------------TGTCGATGAACAAAACATTTGTCACTCACGTTCATGTCGGGAC : 1512
FJ708757  : ACGGCTAATGGACTTGCACTCAG--------------------------------------------------------------------------TGTCGATGAACAAAACATTTGTCACTCACGTTCATGTCGGGAC : 1518
                                                                                                                                                               
                     *      2260         *      2280         *      2300         *      2320         *      2340         *      2360         *      2380       
Genomic   : GGGAACAGAATCATGGTGCCAATAGTTGTATCCACATTAGTGATTATTCTCATTGCCGCCTTGGCTATCATATGCATCATGCGAAGGGAAAGCAAAATAAGTAAGTACAGAGTGTCTTTTACTTAGATTTAAATTTAATA : 2380
NM_119062 : GGGAACAGAATCATGGTGCCAATAGTTGTATCCACATTAGTGATTATTCTCATTGCCGCCTTGGCTATCATATGCATCATGCGAAGGGAAAGCAAAATAA---------------------------------------- : 1612
FJ708757  : GGGAACAGAATCATGGTGCCAATAGTTGTATCCACATTAGTGATTATTCTCATTGCCGCCTTGGCTATCATATGCATCATGCGAAGGGAAAGCAAAATAA---------------------------------------- : 1618
                                                                                                                                                               
                    
                     *      2400         *      2420         *      2440         *      2460         *      2480         *      2500         *      2520       
Genomic   : AGGTAGAATTAATGCTGAAAGAAAACGAGTGTTTAATTTCCTAGTGTATTCAGGAGCATATTCAGGACCACTTCTGCCTTCGGGAAAGAGAAGGTTCACATATAGTGAAGTCTCTAGTATTACAAACAACTTCAATAAAG : 2520
NM_119062 : --------------------------------------------TGTATTCAGGAGCATATTCAGGACCACTTCTGCCTTCGGGAAAGAGAAGGTTCACATATAGTGAAGTCTCTAGTATTACAAACAACTTCAATAAAG : 1708
FJ708757  : --------------------------------------------TGTATTCAGGAGCATATTCAGGACCACTTCTGCCTTCGGGAAAGAGAAGGTTCACATATAGTGAAGTCTCTAGTATTACAAACAACTTCAATAAAG : 1714
                                                                                                                                                               
                     *      2540         *      2560         *      2580         *      2600         *      2620         *      2640         *      2660       
Genomic   : TGATTGGTAAAGGAGGGTTTGGTATTGTGTACCTTGGTTCCCTTGAAGATGGAACTGAAATTGCTGTAAAGATGATCAACGATTCTTCTTTTGGAAAATCTAAAGGATCTTCATCATCATCATCATCATCCCAGGTCTCC : 2660
NM_119062 : TGATTGGTAAAGGAGGGTTTGGTATTGTGTACCTTGGTTCCCTTGAAGATGGAACTGAAATTGCTGTAAAGATGATCAACGATTCTTCTTTTGGAAAATCTAAAGGATCTTCATCATCATCATCATCATCCCAGGTCTCC : 1848
FJ708757  : TGATTGGTAAAGGAGGGTTTGGTATTGTGTACCTTGGTTCCCTTGAAGATGGAACTGAAATTGCTGTAAAGATGATCAACGATTCTTCTTTTGGAAAATCTAAAGGATCTTCATCATCATCATCATCATCCCAGGTCTCC : 1854
                                                                                                                                                               
                     *      2680         *      2700         *      2720         *      2740         *      2760         *      2780         *      2800       
Genomic   : AAAGAATTCCAAGTCGAGGTAACGTAACACTAGAATCATTAATCCGATAAATTAGACAATGTTGTTTTATGGTTGCTGAAGTGTAAAATGTATGCAGGCGGAGCTTCTTTTGACAGTCCATCACCGGAACTTAGCTTCGT : 2800
NM_119062 : AAAGAATTCCAAGTCGAGG-------------------------------------------------------------------------------CGGAGCTTCTTTTGACAGTCCATCACCGGAACTTAGCTTCGT : 1909
FJ708757  : AAAGAATTCCAAGTCGAGG-------------------------------------------------------------------------------CGGAGCTTCTTTTGACAGTCCATCACCGGAACTTAGCTTCGT : 1915
                                                                                                                                                               
                     *      2820         *      2840         *      2860         *      2880         *      2900         *      2920         *      2940       
Genomic   : TTGTTGGATACTGCGATGATGGTCGCAGTATGGCTCTCATCTACGAGTACATGGCCAATGGCAACTTGCAAGATTATCTTTCAAGTATTCTTCATCTCTCTCGATCTCTCTATTGCCAATTTTCGCTGCCTTTGCGCTCA : 2940
NM_119062 : TTGTTGGATACTGCGATGATGGTCGCAGTATGGCTCTCATCTACGAGTACATGGCCAATGGCAACTTGCAAGATTATCTTTCAAGT------------------------------------------------------ : 1995
FJ708757  : TTGTTGGATACTGCGATGATGGTCGCAGTATGGCTCTCATCTACGAGTACATGGCCAATGGCAACTTGCAAGATTATCTTTCAAGT------------------------------------------------------ : 2001
                                                                                                                                                               
                     *      2960         *      2980         *      3000         *      3020         *      3040         *      3060         *      3080       
Genomic   : CGGATGTTTTGTTTACGGATATTCAGGTGAGAATGCAGAGGATCTTAGTTGGGAAAAGAGACTCCATATAGCCATAGACTCTGCACAAGGTTGGTCCCAGTTTTGACATCCTGAAATGTTCAAAAGCTAAGAGACATCAA : 3080
NM_119062 : ----------------------------GAGAATGCAGAGGATCTTAGTTGGGAAAAGAGACTCCATATAGCCATAGACTCTGCACAAGG-------------------------------------------------- : 2057
FJ708757  : ----------------------------GAGAATGCAGAGGATCTTAGTTGGGAAAAGAGACTCCATATAGCCATAGACTCTGCACAAGG-------------------------------------------------- : 2063
                                                                                                                                                               
                     *      3100         *      3120         *      3140         *      3160         *      3180         *      3200         *      3220       
Genomic   : ACCCGGTTTAGTCTGGTTGTAAATCCCCTTATTGATTTGTTTTCCTTGGCAGGGTTGGAGTATCTTCATCATGGCTGCAGGCCACCAATAGTACACAGAGACGTTAAAACAGCAAACATTCTTTTAAACGATAACTTGGA : 3220
NM_119062 : -----------------------------------------------------GTTGGAGTATCTTCATCATGGCTGCAGGCCACCAATAGTACACAGAGACGTTAAAACAGCAAACATTCTTTTAAACGATAACTTGGA : 2144
FJ708757  : -----------------------------------------------------GTTGGAGTATCTTCATCATGGCTGCAGGCCACCAATAGTACACAGAGACGTTAAAACAGCAAACATTCTTTTAAACGATAACTTGGA : 2150
                                                                                                                                                               
                     *      3240         *      3260         *      3280         *      3300         *      3320         *      3340         *      3360       
Genomic   : AGCCAAGATCGCTGATTTCGGGCTTTCTAAGGTCTTCCCTGAGGATGATCTCAGCCACGTTGTGACTGCTGTCATGGGCACTCCTGGCTATGTCGATCCTGAGTAAGTTTCCTTAACACATCACCACTTTTAATCTTTAC : 3360
NM_119062 : AGCCAAGATCGCTGATTTCGGGCTTTCTAAGGTCTTCCCTGAGGATGATCTCAGCCACGTTGTGACTGCTGTCATGGGCACTCCTGGCTATGTCGATCCTGAG------------------------------------- : 2247
FJ708757  : AGCCAAGATCGCTGATTTCTGGCTTTCTAAGGTCTTCCCTGAGGATGATCTCAGCCACGTTGTGACTGCTGTCATGGGCACTCCTGGCTATGTCGATCCTGAG------------------------------------- : 2253
                                                                                                                                                               
                     *      3380         *      3400         *      3420         *      3440         *      3460         *      3480         *      3500       
Genomic   : CATTTTGGTCATGTTGGTCTCATCATAAACTGGTTTCAAAATAGGTACTACAACACGTTTAAGCTAAACGAGAAGAGCGACGTGTACAGCTTTGGGATCGTCCTCCTTGAACTCATAACCGGCAAGAGATCCATAATGAA : 3500
NM_119062 : ---------------------------------------------TACTACAACACGTTTAAGCTAAACGAGAAGAGCGACGTGTACAGCTTTGGGATCGTCCTCCTTGAACTCATAACCGGCAAGAGATCCATAATGAA : 2342
FJ708757  : ---------------------------------------------TACTACAACACGTTTAAGCTAAACGAGAAGAGCGACGTGTACAGCTTTGGGATCGTCCTCCTTGAACTCATAACCGGCAAGAGATCCATAATGAA : 2348
                                                                                                                                                               
                     *      3520         *      3540         *      3560         *      3580         *      3600         *      3620         *      3640       
Genomic   : AACTGACGACGGAGAAAAAATGAACGTTGTTCACTACGTCGAGCCTTTCCTCAAAATGGGAGACATAGATGGTGTCGTGGATCCACGGCTTCACGGCGACTTCTCCTCAAACTCGGCTTGGAAATTTGTGGAAGTGGCAA : 3640
NM_119062 : AACTGACGACGGAGAAAAAATGAACGTTGTTCACTACGTCGAGCCTTTCCTCAAAATGGGAGACATAGATGGTGTCGTGGATCCACGGCTTCACGGCGACTTCTCCTCAAACTCGGCTTGGAAATTTGTGGAAGTGGCAA : 2482
FJ708757  : AACTGACGACGGAGAAAAAATGAACGTTGTTCACTACGTCGAGCCTTTCCTCAAAATGGGAGACATAGATGGTGTCGTGGATCCACGGCTTCACGGCGACTTCTCCTCAAACTCGGCTTGGAAATTTGTGGAAGTGGCAA : 2488
                                                                                                                                                               
                     *      3660         *      3680         *      3700         *      3720         *      3740         *      3760         *      3780       
Genomic   : TGTCTTGCGTCAGAGACAGAGGAACCAACAGACCAAACACAAACCAGATCGTGTCTGACTTGAAACAGTGCTTAGCTGCCGAGCTAGCTCGTGAGCCAAAAAGCAATCATGAGAAGAAGGAAGTAGTGAAAGAGAAGTAC : 3780
NM_119062 : TGTCTTGCGTCAGAGACAGAGGAACCAACAGACCAAACACAAACCAGATCGTGTCTGACTTGAAACAGTGCTTAGCTGCCGAGCTAGCTCGTGAGCCAAAAAGCAATCATGAGAAGAAGGAAGTAGTGAAAGAGAAGTAC : 2622
FJ708757  : TGTCTTGCGTCAGAGACAGAGGAACCAACAGACCAAACACAAACCAGATCGTGTCTGACTTGAAACAGTGCTTAGCTGCCGAGCTAGCTCGTGAGCCAAAAAGCAATCATGAGAAGAAGGAAGTAGTGAAAGAGAAGTAC : 2628
                                                                                                                                  
                     *      3800         *      3820         *      3840         *      3860         *      3880         *        
Genomic   : ACGAAGACCAAGTCGACGGTTCAGAATTATAGCAGCAATGAATATAATAGCTCATCTGGATCGGTTTCACTTTCTTTTGGAGATTACTCCACGTTTGGCCCAATGGCAAGG : 3891
NM_119062 : ACGAAGACCAAGTCGACGGTTCAGAATTATAGCAGCAATGAATATAATAGCTCATCTGGATCGGTTTCACTTTCTTTTGGAGATTACTCCACGTTTGGCCCAATGGCAAGG : 2733
FJ708757  : ACGAAGACCAAGTCGACGGTTCAGAATTATAGCAGCAATGAATATAATAGCTCATCTGGATCGGTTTCACTTTCTTTTGGAGATTACTCCACGTTTGGCCCAATGGCAAGG : 2739


At5g14210
                                                                                                                                                               
                     *        20         *        40         *        60         *        80         *       100         *       120         *       140       
Genomic   : ATGGAACAATTAAAGGTTTTACCTTTGCTCTTTCTTTCATGGGTTATGTTCTTACAAAGCACTCATCAACTGCCGAACTCTCAGACTCAGGTCCTGTATCAGCTCAGGAAGCATCTGGAATTCCCTAAAGCTCTGGAATC :  140
NM_121425 : ATGGAACAATTAAAGGTTTTACCTTTGCTCTTTCTTTCATGGGTTATGTTCTTACAAAGCACTCATCAACTGCCGAACTCTCAGACTCAGGTCCTGTATCAGCTCAGGAAGCATCTGGAATTCCCTAAAGCTCTGGAATC :  140
FJ708776  : ATGGAACAATTAAAGGTTTTACCTTTGCTCTTTCTTTCATGGGTTATGTTCTTACAAAGCACTCATCAACTGCCGAACTCTCAGACTCAGGTCCTGTATCAGCTCAGGAAGCATCTGGAATTCCCTAAAGCTCTGGAATC :  140
                                                                                                                                                               
                     *       160         *       180         *       200         *       220         *       240         *       260         *       280       
Genomic   : TTGGGGAAATTACTATGGAGACTTATGTCAAATCCCTGCAACTGCTCACATGAGCATCACCTGTCAAGGTAATTCCATCACGGAGCTTAAAGTCATGGGGGACAAGCTTTTTAAACCATTTGGTATGTTTGATGGGTCTT :  280
NM_121425 : TTGGGGAAATTACTATGGAGACTTATGTCAAATCCCTGCAACTGCTCACATGAGCATCACCTGTCAAGGTAATTCCATCACGGAGCTTAAAGTCATGGGGGACAAGCTTTTTAAACCATTTGGTATGTTTGATGGGTCTT :  280
FJ708776  : TTGGGGAAATTACTATGGAGACTTATGTCAAATCCCTGCAACTGCTCACATGAGCATCACCTGTCAAGGTAATTCCATCACGGAGCTTAAAGTCATGGGGGACAAGCTTTTTAAACCATTTGGTATGTTTGATGGGTCTT :  280
                                                                                                                                                               
                     *       300         *       320         *       340         *       360         *       380         *       400         *       420       
Genomic   : CACTTCCAAATCACACTCTGTCTGAAGCATTCATAATTGATTCTTTTGTTACCACATTGACAAGGCTTACAAGCTTGAGGGTTCTTAGCTTAGTGTCTCTAGGTATCTATGGTGAGTTCCCAGGGAAAATCCATCGGTTG :  420
NM_121425 : CACTTCCAAATCACACTCTGTCTGAAGCATTCATAATTGATTCTTTTGTTACCACATTGACAAGGCTTACAAGCTTGAGGGTTCTTAGCTTAGTGTCTCTAGGTATCTATGGTGAGTTCCCAGGGAAAATCCATCGGTTG :  420
FJ708776  : CACTTCCAAATCACACTCTGTCTGAAGCATTCATAATTGATTCTTTTGTTACCACATTGACAAGGCTTACAAGCTTGAGGGTTCTTAGCTTAGTGTCTCTAGGTATCTATGGTGAGTTCCCAGGGAAAATCCATCGGTTG :  420
                                                                                                                                                               
                     *       440         *       460         *       480         *       500         *       520         *       540         *       560       
Genomic   : AATTCTCTTGAGTACTTGGATTTGAGTTCAAATTTTCTGTTTGGCTCTGTCCCTCCTGATATTTCCAGATTGGTTATGCTTCAAAGTCTGATGCTTGATGGAAATTACTTCAACGGTAGTGTTCCAGACACGTTGGATTC :  560
NM_121425 : AATTCTCTTGAGTACTTGGATTTGAGTTCAAATTTTCTGTTTGGCTCTGTCCCTCCTGATATTTCCAGATTGGTTATGCTTCAAAGTCTGATGCTTGATGGAAATTACTTCAACGGTAGTGTTCCAGACACGTTGGATTC :  560
FJ708776  : AATTCTCTTGAGTACTTGGATTTGAGTTCAAATTTTCTGTTTGGCTCTGTCCCTCCTGATATTTCCAGATTGGTTATGCTTCAAAGTCTGATGCTTGATGGAAATTACTTCAACGGTAGTGTTCCAGACACGTTGGATTC :  560
                                                                                                                                                               
                     *       580         *       600         *       620         *       640         *       660         *       680         *       700       
Genomic   : CTTGACCAATCTTACTGTTCTTAGTTTAAAGAACAATCGGTTCAAAGGTCCATTTCCTTCTTCAATTTGCAGAATTGGAAGACTAACAAATCTTGCTCTATCACACAATGAGATTTCTGGTAAATTGCCTGATCTCAGCA :  700
NM_121425 : CTTGACCAATCTTACTGTTCTTAGTTTAAAGAACAATCGGTTCAAAGGTCCATTTCCTTCTTCAATTTGCAGAATTGGAAGACTAACAAATCTTGCTCTATCACACAATGAGATTTCTGGTAAATTGCCTGATCTCAGCA :  700
FJ708776  : CTTGACCAATCTTACTGTTCTTAGTTTAAAGAACAATCGGTTCAAAGGTCCATTTCCTTCTTCAATTTGCAGAATTGGAAGACTAACAAATCTTGCTCTATCACACAATGAGATTTCTGGTAAATTGCCTGATCTCAGCA :  700
                                                                                                                                                               
                     *       720         *       740         *       760         *       780         *       800         *       820         *       840       
Genomic   : AGTTAAGTCATCTGCATATGCTGGATCTGAGAGAAAACCACTTGGATTCTGAACTACCTGTCATGCCTATAAGATTAGTTACTGTTCTCCTGAGCAAGAACTCCTTCTCAGGCGAAATTCCAAGACGTTTTGGCGGTTTG :  840
NM_121425 : AGTTAAGTCATCTGCATATGCTGGATCTGAGAGAAAACCACTTGGATTCTGAACTACCTGTCATGCCTATAAGATTAGTTACTGTTCTCCTGAGCAAGAACTCCTTCTCAGGCGAAATTCCAAGACGTTTTGGCGGTTTG :  840
FJ708776  : AGTTAAGTCATCTGCATATGCTGGATCTGAGAGAAAACCACTTGGATTCTGAACTACCTGTCATGCCTATAAGATTAGTTACTGTTCTCCTGAGCAAGAACTCCTTCTCAGGCGAAATTCCAAGACGTTTTGGCGGTTTG :  840
                                                                                                                                                               
                     *       860         *       880         *       900         *       920         *       940         *       960         *       980       
Genomic   : TCTCAGCTTCAGCATCTTGACTTGTCGTTCAATCATCTGACTGGAACTCCATCTCGGTTCTTGTTCTCTTTGCCAAACATTAGTTACTTGGATTTGGCATCTAACAAGCTCAGTGGGAAGCTACCTCTTAATCTGACCTG :  980
NM_121425 : TCTCAGCTTCAGCATCTTGACTTGTCGTTCAATCATCTGACTGGAACTCCATCTCGGTTCTTGTTCTCTTTGCCAAACATTAGTTACTTGGATTTGGCATCTAACAAGCTCAGTGGGAAGCTACCTCTTAATCTGACCTG :  980
FJ708776  : TCTCAGCTTCAGCATCTTGACTTGTCGTTCAATCATCTGACTGGAACTCCATCTCGGTTCTTGTTCTCTTTGCCAAACATTAGTTACTTGGATTTGGCATCTAACAAGCTCAGTGGGAAGCTACCTCTTAATCTGACCTG :  980
                                                                                                                                                               
                     *      1000         *      1020         *      1040         *      1060         *      1080         *      1100         *      1120       
Genomic   : TGGAGGCAAACTCGGATTTGTGGATTTGTCGAATAACAGATTGATTGGGACTCCTCCCCGTTGCTTGGCAGGAGCTTCCGGCGAGCGAGTTGTTAAACTTGGTGGAAACTGCTTGTCCATAATTGGCAGTCATGATCAGC : 1120
NM_121425 : TGGAGGCAAACTCGGATTTGTGGATTTGTCGAATAACAGATTGATTGGGACTCCTCCCCGTTGCTTGGCAGGAGCTTCCGGCGAGCGAGTTGTTAAACTTGGTGGAAACTGCTTGTCCATAATTGGCAGTCATGATCAGC : 1120
FJ708776  : TGGAGGCAAACTCGGATTTGTGGATTTGTCGAATAACAGATTGATTGGGACTCCTCCCCGTTGCTTGGCAGGAGCTTCCGGCGAGCGAGTTGTTAAACTTGGTGGAAACTGCTTGTCCATAATTGGCAGTCATGATCAGC : 1120
                                                                                                                                                               
                     *      1140         *      1160         *      1180         *      1200         *      1220         *      1240         *      1260       
Genomic   : ATCAAGAATTCTTATGCGAAGAGGCTGAAACTGAGGGAAAACAGTTCCAAGGAAGAAAGGTTGGGATTTTGATTGCTGTCATTGGTGGAGCTGTTCTTGTTCTTGTGTTTTTTGTATTAGTTATCCTCCTCCTGTTATGC : 1260
NM_121425 : ATCAAGAATTCTTATGCGAAGAGGCTGAAACTGAGGGAAAACAGTTCCAAGGAAGAAAGGTTGGGATTTTGATTGCTGTCATTGGTGGAGCTGTTCTTGTTCTTGTGTTTTTTGTATTAGTTATCCTCCTCCTGTTATGC : 1260
FJ708776  : ATCAAGAATTCTTATGCGAAGAGGCTGAAACTGAGGGAAAACAGTTCCAAGGAAGAAAGGTTGGGATTTTGATTGCTGTCATTGGTGGAGCTGTTCTTGTTCTTGTGTTTTTTGTATTAGTTATCCTCCTCCTGTTATGC : 1260
                                                                                                                                                               
                     *      1280         *      1300         *      1320         *      1340         *      1360         *      1380         *      1400       
Genomic   : ACAAACCGCTGCTCTAGCTGTTGTTCAAGAGAAAAGTCAGTGCCGCAAACTCGGCTTAAGGTTGTGACAGATAACTCACACACCAGTCTCTCCTCTGAAGTTCTTGCTAGTGCAAGTGAGTAACTTTTGACTCAAAATGT : 1400
NM_121425 : ACAAACCGCTGCTCTAGCTGTTGTTCAAGAGAAAAGTCAGTGCCGCAAACTCGGCTTAAGGTTGTGACAGATAACTCACACACCAGTCTCTCCTCTGAAGTTCTTGCTAGTGCAAG------------------------ : 1376
FJ708776  : ACAAACCGCTGCTCTAGCTGTTGTTCAAGAGAAAAGTCAGTGCCGCAAACTCGGCTTAAGGTTGTGACAGATAACTCACACACCAGTCTCTCCTCTGAAGTTCTTGCTAGTGCAAG------------------------ : 1376
                                                                                                                                                               
                     *      1420         *      1440         *      1460         *      1480         *      1500         *      1520         *      1540       
Genomic   : AGACTATTCCTGCTAGGAATTGTCTTTCAGGGATTTAATATCCTTGTATGATTTCGTCTTGCAGGGTTAATCTCTCAAACAGCAAAGCTAGGTGCACAGGGTGTGCCCTCATGCCGGTCCTTTTCTTTTGAAGATTTAAA : 1540
NM_121425 : -----------------------------------------------------------------GTTAATCTCTCAAACAGCAAAGCTAGGTGCACAGGGTGTGCCCTCATGCCGGTCCTTTTCTTTTGAAGATTTAAA : 1451
FJ708776  : -----------------------------------------------------------------GTTAATCTCTCAAACAGCAAAGCTAGGTGCACAGGGTGTGCCCTCATGCCGGTCCTTTTCTTTTGAAGATTTAAA : 1451
                                                                                                                                                               
                     *      1560         *      1580         *      1600         *      1620         *      1640         *      1660         *      1680       
Genomic   : GGAAGCCACAGACGATTTTGATTCATCACGTTTCTTAGGTGAAGGCTCCCTTGGAAAGGTATGGTTTGTCTTCTGCAAGATTAATTCTTGTGATCTGCCTATCTGTCATTTGCTTTACTCTGCTTGTTTTGATCCTTTGC : 1680
NM_121425 : GGAAGCCACAGACGATTTTGATTCATCACGTTTCTTAGGTGAAGGCTCCCTTGGAAAG---------------------------------------------------------------------------------- : 1509
FJ708776  : GGAAGCCACAGACGATTTTGATTCATCACGTTTCTTAGGTGAAGGCTCCCTTGGAAAG---------------------------------------------------------------------------------- : 1509
                                                                                                                                                               
                     *      1700         *      1720         *      1740         *      1760         *      1780         *      1800         *      1820       
Genomic   : AGCTATACAGAGGAACACTGGAAAATGGAAGTTCCATAGCTATCAGATGTCTGGTTTTATCAAGGAAATTCTCCAGCCAGAGTATCAGAGGTCACTTAGACTGGATGGCAAAGCTCAACCATCCTCATCTCCTTGGCTTC : 1820
NM_121425 : --CTATACAGAGGAACACTGGAAAATGGAAGTTCCATAGCTATCAGATGTCTGGTTTTATCAAGGAAATTCTCCAGCCAGAGTATCAGAGGTCACTTAGACTGGATGGCAAAGCTCAACCATCCTCATCTCCTTGGCTTC : 1647
FJ708776  : --CTATACAGAGGAACACTGGAAAATGGAAGTTCCATAGCTATCAGATGTCTGGTTTTATCAAGGAAATTCTCCAGCCAGAGTATCAGAGGTCACTTAGACTGGATGGCAAAGCTCAACCATCCTCATCTCCTTGGCTTC : 1647
                                                                                                                                                               
                     *      1840         *      1860         *      1880         *      1900         *      1920         *      1940         *      1960       
Genomic   : TTGGGCCATTGTACGCAAACCAGCGGAGAACACGATCCTGTTGCAACCATACTCTACCTTGTATACGAGTATATGCCCAACGGGAGCTATCGCACACATCTATCAGGTCAGTCGTTTGGCATTTGTAGTAAACTAAATGT : 1960
NM_121425 : TTGGGCCATTGTACGCAAACCAGCGGAGAACACGATCCTGTTGCAACCATACTCTACCTTGTATACGAGTATATGCCCAACGGGAGCTATCGCACACATCTATCAG---------------------------------- : 1753
FJ708776  : TTGGGCCATTGTACGCAAACCAGCGGAGAACACGATCCTGTTGCAACCATACTCTACCTTGTATACGAGTATATGCCCAACGGGAGCTATCGCACACATCTATCAG---------------------------------- : 1753
                                                                                                                                                               
                     *      1980         *      2000         *      2020         *      2040         *      2060         *      2080         *      2100       
Genomic   : TATACTGAATCTCTCTGTACTTGGAAAATCGTTGTTGTTAGAATATTGTTTATTTGTAGAATCTTTCTCGGAGAAGATCCTGACATGGCCAGATCGGCTAGCAATTCTGATTGAGATAGCAAAGGCAGTTCATTTTCTTC : 2100
NM_121425 : -----------------------------------------------------------AATCTTTCTCGGAGAAGATCCTGACATGGCCAGATCGGCTAGCAATTCTGATTGAGATAGCAAAGGCAGTTCATTTTCTTC : 1834
FJ708776  : -----------------------------------------------------------AATCTTTCTCGGAGAAGATCCTGACATGGCCAGATCGGCTAGCAATTCTGATTGAGATAGCAAAGGCAGTTCATTTTCTTC : 1834
                                                                                                                                                               
                     *      2120         *      2140         *      2160         *      2180         *      2200         *      2220         *      2240       
Genomic   : ACACCGGTGTAATGCCTGGTTCATTCAACAATCAGCTTAAGACTAACAATATTTTGCTTGACGAACACAAGATTGCAAAGCTCAGTGACTATGGAGTCTCTGCCATCATTGAAGAGAACGAAAAGCTTGAGGTTTGCTCT : 2240
NM_121425 : ACACCGGTGTAATGCCTGGTTCATTCAACAATCAGCTTAAGACTAACAATATTTTGCTTGACGAACACAAGATTGCAAAGCTCAGTGACTATGGAGTCTCTGCCATCATTGAAGAGAACGAAAAGCTTGAG--------- : 1965
FJ708776  : ACACCGGTGTAATGCCTGGTTCATTCAACAATCAGCTTAAGACTAACAATATTTTGCTTGACGAACACAAGATTGCAAAGCTCAGTGACTATGGAGTCTCTGCCATCATTGAAGAGAACGAAAAGCTTGAG--------- : 1965
                                                                                                                                                               
                     *      2260         *      2280         *      2300         *      2320         *      2340         *      2360         *      2380       
Genomic   : ATTTACTCTCTTTCACTGTGTTTATCTTCATTTTGTATGATCTTCTTTGACACTAACTCAGGACTCTACTTTACTCCTGCTTCTACATTTTGCAGACAAAGTCAGAAACCCACAAGTCAAAGTATGCATCTTTTGTCCTT : 2380
NM_121425 : -------------------------------------------------------------GACTCTACTTTACTCCTGCTTCTACATTTTGCAGACAAAGTCAGAAACCCACAAGTCAAAGTATGCATCTTTTGTCCTT : 2044
FJ708776  : -----------------------------------------------------------------------------------------------ACAAAGTCAGAAACCCACAAGTCAAAG------------------ : 1992
                                                                                                                                                               
                                                                                                                                                          
                     *      2400         *      2420         *      2440         *      2460         *      2480         *      2500         *      2520       
Genomic   : GTATAGCAAAACTCCATGGCATTTATGAGAATCTAATCCAAACTTTGAGATTATGCAGGAAAAAGGCTAAAAGAGAGGACGATGTGTACAACTTCGGATTCATACTTCTGGAATCTCTGATAGGACCAGTGCCCACTACA : 2520
NM_121425 : GTATAGCAAAACTCCATGGCATTTATGAGAATCTAATCCAAACTTTGAGATTATGCAGGAAAAAGGCTAAAAGAGAGGACGATGTGTACAACTTCGGATTCATACTTCTGGAATCTCTGATAGGACCAGTGCCCACTACA : 2184
FJ708776  : -----------------------------------------------------------AAAAAGGCTAAAAGAGAGGACGATGTGTACAACTTCGGATTCATACTTCTGGAATCTCTGATAGGACCAGTGCCCACTACA : 2073
                                                                                                                                                               
                     *      2540         *      2560         *      2580         *      2600         *      2620         *      2640         *      2660       
Genomic   : AAAGGAGAGGCTTTTCTTCTCAACGAAATGGTAACAAAGAGAAAAAGCAAAAAAAAAAAGCATCATCATTGTTATTTGATTGAAACAAATTTGGTTAATAGCTGTTGCTGCAATGTTGTGTTTGCAGACATCATTTGGGA : 2660
NM_121425 : AAAGGAGAGGCTTTTCTTCTCAACGAAATG-------------------------------------------------------------------------------------------------ACATCATTTGGGA : 2227
FJ708776  : AAAGGAGAGGCTTTTCTTCTCAACGAAATG-------------------------------------------------------------------------------------------------ACATCATTTGGGA : 2116
                                                                                                                                                               
                     *      2680         *      2700         *      2720         *      2740         *      2760         *      2780         *      2800       
Genomic   : GCCAAGACGGTCGGCAGAAAATAGTAAGTCCAACAGTCCTAACAACAAGCTCGCAGGAGTCTTTATCGATTGCGATCTCAATCGCCAATAAATGTGTTTTGCTTGAACCTTCCGCAAGACCTTCCTTTGAAGATGTTCTC : 2800
NM_121425 : GCCAAGACGGTCGGCAGAAAATAGTAAGTCCAACAGTCCTAACAACAAGCTCGCAGGAGTCTTTATCGATTGCGATCTCAATCGCCAATAAATGTGTTTTGCTTGAACCTTCCGCAAGACCTTCCTTTGAAGATGTTCTC : 2367
FJ708776  : GCCAAGACGGTCGGCAGAAAATAGTAAGTCCAACAGTCCTAACAACAAGCTCGCAGGAGTCTTTATCGATTGCGATCTCAATCGCCAATAAATGTGTTTTGCTTGAACCTTCCGCAAGACCTTCCTTTGAAGATGTTCTC : 2256
                                                                                                                                                               
                     *      2820         *      2840         *      2860                
Genomic   : TGGAACTTACAATACGCAGCTCAAATGCAGTCTGCCGCAGACGCTGAACGCAAATCTGATACGTCGTCG : 2869
NM_121425 : TGGAACTTACAATACGCAGCTCAAATGCAGTCTGCCGCAGACGCTGAACGCAAATCTGATACGTCGTCG : 2436
FJ708776  : TGGAACTTACAATACGCAGCTCAAATGCAGTCTGCCGCAGACGCTGAACGCAAATCTGATACGTCGTCG : 2325


At5g35390
                                                                                                                                                               
                     *        20         *        40         *        60         *        80         *       100         *       120         *       140       
Genomic   : ATGCCTCCCATGCAGGCGCGTACCCTCAGCGTATACAACGTCATGGTACCACTAGTATGTCTCCTCCTTTTCTTCTCCACACCCACTCATGGTCTTTCAGATTCTGAGGCTATCCTCAAGTTCAAGGAATCTTTAGTTGT :  140
NM_122930 : ATGCCTCCCATGCAGGCGCGTACCCTCAGCGTATACAACGTCATGGTACCACTAGTATGTCTCCTCCTTTTCTTCTCCACACCCACTCATGGTCTTTCAGATTCTGAGGCTATCCTCAAGTTCAAGGAATCTTTAGTTGT :  140
FJ708784  : ATGCCTCCCATGCAGGCGCGTACCCTCAGCGTATACAACGTCATGGTACCACTAGTATGTCTCCTCCTTTTCTTCTCCACACCCACTCATGGTCTTTCAGATTCTGAGGCTATCCTCAAGTTCAAGGAATCTTTAGTTGT :  140
                                                                                                                                                               
                     *       160         *       180         *       200         *       220         *       240         *       260         *       280       
Genomic   : TGGGCAGGAAAACGCGTTGGCCTCATGGAATGCTAAAAGTCCTCCTTGCACTTGGTCTGGTGTCTTGTGCAATGGCGGCTCCGTCTGGAGACTGCAGATGGAGAACTTGGAGCTTTCTGGTTCTATAGACATCGAGGCAT :  280
NM_122930 : TGGGCAGGAAAACGCGTTGGCCTCATGGAATGCTAAAAGTCCTCCTTGCACTTGGTCTGGTGTCTTGTGCAATGGCGGCTCCGTCTGGAGACTGCAGATGGAGAACTTGGAGCTTTCTGGTTCTATAGACATCGAGGCAT :  280
FJ708784  : TGGGCAGGAAAACGCGTTGGCCTCATGGAATGCTAAAAGTCCTCCTTGCACTTGGTCTGGTGTCTTGTGCAATGGCGGCTCCGTCTGGAGACTGCAGATGGAGAACTTGGAGCTTTCTGGTTCTATAGACATCGAGGCAT :  280
                                                                                                                                                               
                     *       300         *       320         *       340         *       360         *       380         *       400         *       420       
Genomic   : TGTCGGGTTTGACATCCTTGAGGACTCTAAGCTTCATGAACAACAAGTTTGAAGGGCCATTCCCTGACTTCAAGAAACTCGCTGCTCTCAAGTCACTCTACCTGTCAAACAATCAATTCGGAGGAGATATACCAGGGGAT :  420
NM_122930 : TGTCGGGTTTGACATCCTTGAGGACTCTAAGCTTCATGAACAACAAGTTTGAAGGGCCATTCCCTGACTTCAAGAAACTCGCTGCTCTCAAGTCACTCTACCTGTCAAACAATCAATTCGGAGGAGATATACCAGGGGAT :  420
FJ708784  : TGTCGGGTTTGACATCCTTGAGGACTCTAAGCTTCATGAACAACAAGTTTGAAGGGCCATTCCCTGACTTCAAGAAACTCGCTGCTCTCAAGTCACTCTACCTGTCAAACAATCAATTCGGAGGAGATATACCAGGGGAT :  420
                                                                                                                                                               
                     *       440         *       460         *       480         *       500         *       520         *       540         *       560       
Genomic   : GCTTTTGAAGGTATGGGGTGGTTGAAGAAGGTCCATTTGGCACAAAACAAGTTTACCGGTCAAATTCCGTCATCTGTGGCCAAATTGCCCAAGCTTTTAGAACTGAGACTTGACGGGAACCAATTCACTGGAGAGATACC :  560
NM_122930 : GCTTTTGAAGGTATGGGGTGGTTGAAGAAGGTCCATTTGGCACAAAACAAGTTTACCGGTCAAATTCCGTCATCTGTGGCCAAATTGCCCAAGCTTTTAGAACTGAGACTTGACGGGAACCAATTCACTGGAGAGATACC :  560
FJ708784  : GCTTTTGAAGGTATGGGGTGGTTGAAGAAGGTCCATTTGGCACAAAACAAGTTTACCGGTCAAATTCCGTCATCTGTGGCCAAATTGCCCAAGCTTTTAGAACTGAGACTTGACGGGAACCAATTCACTGGAGAGATACC :  560
                                                                                                                                                               
                     *       580         *       600         *       620         *       640         *       660         *       680         *       700       
Genomic   : AGAGTTTGAACATCAGCTACACTTGTTAAACCTTTCAAACAATGCATTAACAGGTCCAATACCAGAGAGTCTCAGCATGACAGATCCAAAGGTGTTTGAAGGTAACAAAGGCTTATATGGAAAACCGCTGGAAACAGAAT :  700
NM_122930 : AGAGTTTGAACATCAGCTACACTTGTTAAACCTTTCAAACAATGCATTAACAGGTCCAATACCAGAGAGTCTCAGCATGACAGATCCAAAGGTGTTTGAAGGTAACAAAGGCTTATATGGAAAACCGCTGGAAACAGAAT :  700
FJ708784  : AGAGTTTGAACATCAGCTACACTTGTTAAACCTTTCAAACAATGCATTAACAGGTCCAATACCAGAGAGTCTCAGCATGACAGATCCAAAGGTGTTTGAAGGTAACAAAGGCTTATATGGAAAACCGCTGGAAACAGAAT :  700
                                                                                                                                                               
                     *       720         *       740         *       760         *       780         *       800         *       820         *       840       
Genomic   : GTGATTCTCCTTATATAGAACATCCTCCACAGTCTGAGGCACGACCCAAATCATCATCAAGGGGACCTCTCGTTATAACAGCAATAGTCGCGGCCTTGACAATACTCATAATCCTTGGAGTGATCTTCTTGCTTAACCGC :  840
NM_122930 : GTGATTCTCCTTATATAGAACATCCTCCACAGTCTGAGGCACGACCCAAATCATCATCAAGGGGACCTCTCGTTATAACAGCAATAGTCGCGGCCTTGACAATACTCATAATCCTTGGAGTGATCTTCTTGCTTAACCGC :  840
FJ708784  : GTGATTCTCCTTATATAGAACATCCTCCACAGTCTGAGGCACGACCCAAATCATCATCAAGGGGACCTCTCGTTATAACAGCAATAGTCGCGGCCTTGACAATACTCATAATCCTTGGAGTGATCTTCTTGCTTAACCGC :  840
                                                                                                                                                                                                                                                                                                                              
                     *       860         *       880         *       900         *       920         *       940         *       960         *       980       
Genomic   : AGCTATAAGAATAAAAAACCGCGTTTGGCGGTGGAAACCGGACCATCAAGTCTACAGAAGAAAACCGGCATCCGAGAAGCAGACCAAAGTCGTAGAGACAGAAAGAAAGCCGACCACCGGAAAGGCTCAGGCACTACTAA :  980
NM_122930 : AGCTATAAGAATAAAAAACCGCGTTTGGCGGTGGAAACCGGACCATCAAGTCTACAGAAGAAAACCGGCATCCGAGAAGCAGACCAAAGTCGTAGAGACAGAAAGAAAGCCGACCACCGGAAAGGCTCAGGCACTACTAA :  980
FJ708784  : AGCTATAAGAATAAAAAACCGCGTTTGGCGGTGGAAACCGGACCATCAAGTCTACAGAAGAAAACCGGCATCCGAGAAGCAGACCAAAGTCGTAGAGACAGAAAGAAAGCCGACCACCGGAAAGGCTCAGGCACTACTAA :  980
                                                                                                                                                               
                     *      1000         *      1020         *      1040         *      1060         *      1080         *      1100         *      1120       
Genomic   : GAGGATGGGTGCAGCAGCTGGAGTGGAGAACACAAAGCTTTCATTCTTGAGGGAAGATAGGGAAAAATTTGACCTGCAAGATCTGTTGAAGGCTTCGGCCGAGATACTTGGAAGTGGATGTTTTGGAGCATCTTATAAAG : 1120
NM_122930 : GAGGATGGGTGCAGCAGCTGGAGTGGAGAACACAAAGCTTTCATTCTTGAGGGAAGATAGGGAAAAATTTGACCTGCAAGATCTGTTGAAGGCTTCGGCCGAGATACTTGGAAGTGGATGTTTTGGAGCATCTTATAAAG : 1120
FJ708784  : GAGGATGGGTGCAGCAGCTGGAGTGGAGAACACAAAGCTTTCATTCTTGAGGGAAGATAGGGAAAAATTTGACCTGCAAGATCTGTTGAAGGCTTCGGCCGAGATACTTGGAAGTGGATGTTTTGGAGCATCTTATAAAG : 1120
                                                                                                                                                               
                     *      1140         *      1160         *      1180         *      1200         *      1220         *      1240         *      1260       
Genomic   : CAGTGCTATCAAGCGGACAAATGATGGTCGTGAAGAGGTTCAAGCAGATGAACAATGCAGGGAGGGATGAGTTTCAAGAGCACATGAAAAGATTAGGGAGGTTAATGCACCATAATCTGCTTTCCATTGTGGCTTATTAC : 1260
NM_122930 : CAGTGCTATCAAGCGGACAAATGATGGTCGTGAAGAGGTTCAAGCAGATGAACAATGCAGGGAGGGATGAGTTTCAAGAGCACATGAAAAGATTAGGGAGGTTAATGCACCATAATCTGCTTTCCATTGTGGCTTATTAC : 1260
FJ708784  : CAGTGCTATCAAGCGGACAAATGATGGTCGTGAAGAGGTTCAAGCAGATGAACAATGCAGGGAGGGATGAGTTTCAAGAGCACATGAAAAGATTAGGGAGGTTAATGCACCATAATCTGCTTTCCATTGTGGCTTATTAC : 1260
                                                                                                                                                               
                     *      1280         *      1300         *      1320         *      1340         *      1360         *      1380         *      1400       
Genomic   : TATAGGAAGGAGGAAAAGCTTTTAGTATGTGATTTTGCTGAGAGAGGAAGCTTGGCTATTAATCTTCATAGTAAGTTTTCGATATTTCTACACTTATAAGTCGTTAGATTTATCGATGCTTACGAGTCAAATGTTTGTAG : 1400
NM_122930 : TATAGGAAGGAGGAAAAGCTTTTAGTATGTGATTTTGCTGAGAGAGGAAGCTTGGCTATTAATCTTCATAG--------------------------------------------------------------------- : 1331
FJ708784  : TATAGGAAGGAGGAAAAGCTTTTAGTATGTGATTTTGCTGAGAGAGGAAGCTTGGCTATTAATCTTCATAG--------------------------------------------------------------------- : 1331
                                                                                                                                                               
                     *      1420         *      1440         *      1460         *      1480         *      1500         *      1520         *      1540       
Genomic   : GTAATCAATCGTTAGGAAAACCAAGTTTGGACTGGCCAACAAGATTGAAGATCGTGAAGGGAGTTGCAAAGGGGTTGTTCTATCTCCACCAAGACCTACCTAGCCTAATGGCTCCACATGGTCACCTCAAATCCTCCAAC : 1540
NM_122930 : ----------------AAAACCAAGTTTGGACTGGCCAACAAGATTGAAGATCGTGAAGGGAGTTGCAAAGGGGTTGTTCTATCTCCACCAAGACCTACCTAGCCTAATGGCTCCACATGGTCACCTCAAATCCTCCAAC : 1455
FJ708784  : -TAATCAATCGTTAGGAAAACCAAGTTTGGACTGGCCAACAAGATTGAAGATCGTGAAGGGAGTTGCAAAGGGGTTGTTCTATCTCCACCAAGACCTACCTAGCCTAATGGCTCCACATGGTCACCTCAAATCCTCCAAC : 1470
                                                                                                                                                               
                     *      1560         *      1580         *      1600         *      1620         *      1640         *      1660         *      1680       
Genomic   : GTTCTTCTCACCAAAACATTTGAACCACTTCTCACAGACTATGGATTAATTCCGTTGATCAACCAAGAGAAGGCACAAATGCACATGGCGGCCTATAGATCTCCAGAGTATTTGCAACATCGACGTATCACCAAGAAAAC : 1680
NM_122930 : GTTCTTCTCACCAAAACATTTGAACCACTTCTCACAGACTATGGATTAATTCCGTTGATCAACCAAGAGAAGGCACAAATGCACATGGCGGCCTATAGATCTCCAGAGTATTTGCAACATCGACGTATCACCAAGAAAAC : 1595
FJ708784  : GTTCTTCTCACCAAAACATTTGAACCACTTCTCACAGACTATGGATTAATTCCGTTGATCAACCAAGAGAAGGCACAAATGCACATGGCGGCCTATAGATCTCCAGAGTATTTGCAACATCGACGTATCACCAAGAAAAC : 1610
                                                                                                                                                               
                     *      1700         *      1720         *      1740         *      1760         *      1780         *      1800         *      1820       
Genomic   : CGATGTATGGGGGCTTGGCATACTTATCTTGGAGATCCTAACGGGAAAATTCCCGGCTAATTTCTCACAAAGTAGTGAAGAGGATTTAGCGAGTTGGGTGAACTCGGGTTTCCATGGGGTATGGGCACCAAGTTTGTTTG : 1820
NM_122930 : CGATGTATGGGGGCTTGGCATACTTATCTTGGAGATCCTAACGGGAAAATTCCCGGCTAATTTCTCACAAAGTAGTGAAGAGGATTTAGCGAGTTGGGTGAACTCGGGTTTCCATGGGGTATGGGCACCAAGTTTGTTTG : 1735
FJ708784  : CGATGTATGGGGGCTTGGCATACTTATCTTGGAGATCCTAACGGGAAAATTCCCGGCTAATTTCTCACAAAGTAGTGAAGAGGATTTAGCGAGTTGGGTGAACTCGGGTTTCCATGGGGTATGGGCACCAAGTTTGTTTG : 1750
                                                                                                                                                               
                     *      1840         *      1860         *      1880         *      1900         *      1920         *      1940         *      1960       
Genomic   : ATAAGGGTATGGGGAAGACAAGCCATTGTGAAGGACAGATTCTCAAACTCTTGACGATCGGATTGAACTGTTGTGAACCCGACGTGGAGAAAAGGTTGGACATAGGACAGGCTGTGGAGAAGATCGAAGAGTTGAAGGAG : 1960
NM_122930 : ATAAGGGTATGGGGAAGACAAGCCATTGTGAAGGACAGATTCTCAAACTCTTGACGATCGGATTGAACTGTTGTGAACCCGACGTGGAGAAAAGGTTGGACATAGGACAGGCTGTGGAGAAGATCGAAGAGTTGAAGGAG : 1875
FJ708784  : ATAAGGGTATGGGGAAGACAAGCCATTGTGAAGGACAGATTCTCAAACTCTTGACGATCGGATTGAACTGTTGTGAACCCGACGTGGAGAAAAGGTTGGACATAGGACAGGCTGTGGAGAAGATCGAAGAGTTGAAGGAG : 1890
                                                                                                                   
                     *      1980         *      2000         *      2020         *      2040         *             
Genomic   : CGAGAAGGGGATGATGACGACTTCTACTCAACGTATGTGAGTGAAACTGATGGTAGGTCGTCTAAAGGAGAGTCATGCGAGAGTATCAGCTTTGCA : 2056
NM_122930 : CGAGAAGGGGATGATGACGACTTCTACTCAACGTATGTGAGTGAAACTGATGGTAGGTCGTCTAAAGGAGAGTCATGCGAGAGTATCAGCTTTGCA : 1971
FJ708784  : CGAGAAGGGGATGATGACGACTTCTACTCAACGTATGTGAGTGAAACTGATGGTAGGTCGTCTAAAGGAGAGTCATGCGAGAGTATCAGCTTTGCA : 1986


At5g37450
                                                                                                                                                               
                     *        20         *        40         *        60         *        80         *       100         *       120         *       140       
Genomic   : ATGAAAGAGATGATGGGTGTAGTTGGAATCATTCTGGTTGTGTCTTCTTGTTGCTTGTCTTTGCTGGATGCCCAAGAGATTACTCATCCCACTGATGGTACGGTCCTTTAGATTTCTCTGAGCACCATCTATATGTGAAC :  140
NM_123104 : ATGAAAGAGATGATGGGTGTAGTTGGAATCATTCTGGTTGTGTCTTCTTGTTGCTTGTCTTTGCTGGATGCCCAAGAGATTACTCATCCCACTGATG------------------------------------------- :   97
FJ708785  : ATGAAAGAGATGATGGGTGTAGTTGGAATCATTCTGGTTGTGTCTTCTTGTTGCTTGTCTTTGCTGGATGCCCAAGAGATTACTCATCCCACTGATG------------------------------------------- :   97
                                                                                                                                                               
                     *       160         *       180         *       200         *       220         *       240         *       260         *       280       
Genomic   : AGTGATAAGTGATGCTAATGAGGGTGTTTTGTTGTTGCAGTTAGTGCTCTGCAGTATGTTCACCGCAAACTAAAGGACCCTCTGAATCATCTTCAAGACTGGAAAAAGACGGATCCATGCGCCTCTAACTGGACCGGTGT :  280
NM_123104 : ----------------------------------------TTAGTGCTCTGCAGTATGTTCACCGCAAACTAAAGGACCCTCTGAATCATCTTCAAGACTGGAAAAAGACGGATCCATGCGCCTCTAACTGGACCGGTGT :  197
FJ708785  : ----------------------------------------TTAGTGCTCTGCAGTATGTTCACCGCAAACTAAAGGACCCTCTGAATCATCTTCAAGACTGGAAAAAGACGGATCCATGCGCCTCTAACTGGACCGGTGT :  197
                                                                                                                                                               
                     *       300         *       320         *       340         *       360         *       380         *       400         *       420       
Genomic   : CATCTGCATCCCTGATCCTTCTGATGGCTTTCTTCATGTTAAAGAATTGTATGTTCGGACTTTAAAACTTTCTTCTATTGTTACTCAAAAGCAAACCTTAAGGATGTCATATGGTTTGATGAAATGGTGCAGGCGGTTGC :  420
NM_123104 : CATCTGCATCCCTGATCCTTCTGATGGCTTTCTTCATGTTAAAGAATTG------------------------------------------------------------------------------------------- :  246
FJ708785  : CATCTGCATCCCTGATCCTTCTGATGGCTTTCTTCATGTTAAAGAATTG------------------------------------------------------------------------------------CGGTTGC :  253
                                                                                                                                                               
                     *       440         *       460         *       480         *       500         *       520         *       540         *       560       
Genomic   : TAAACATGAACCTCACTGGACAACTGGCACCTGAACTCGGCTTACTATCAAATCTTACCATATTGTATGTTCTGTGTTTTGGTCTGTGCATGCTCTGGTTTTTTGAAATCAAACTATAGATATCAACGTCTCTCCTTTTT :  560
NM_123104 : -------------------------------------------------------------------------------------------------------------------------------------------- :    -
FJ708785  : TAAACATGAACCTCACTGGACAACTGGCACCTGAACTCGGCTTACTATCAAATCTTACCATATTG--------------------------------------------------------------------------- :  318
                                                                                                                                                               
                     *       580         *       600         *       620         *       640         *       660         *       680         *       700       
Genomic   : ATATTTTCTGATCACAGGAACTTCATGTGGAACGATCTCACCGGTCAGATTCCACCAGAACTAGGAAACTTAACTCATCTCATCTTCTTGTAAGTAGTCTTGATCATTGTTTCATCAATATTTCCTCTTCATGGATGCAT :  700
NM_123104 : -------------------------------------------------------------------------------------------------------------------------------------------- :    -
FJ708785  : ------------------AACTTCATGTGGAACGATCTCACCGGTCAGATTCCACCAGAACTAGGAAACTTAACTCATCTCATCTTCTTG-------------------------------------------------- :  390
                                                                                                                                                               
                     *       720         *       740         *       760         *       780         *       800         *       820         *       840       
Genomic   : AACTTAACAGATTCATGTTATCTTTTGTCAGGCTATTAAGTGGAAATCAACTGACAGGATCTTTGCCTCAAGAACTCGGTTCTCTTTCCAATCTCTTAATCCTTCAGATAGATTACAATGAAATTAGCGGAAAACTTCCA :  840
NM_123104 : --------------------------------CTATTAAGTGGAAATCAACTGACAGGATCTTTGCCTCAAGAACTCGGTTCTCTTTCCAATCTCTTAATCCTTCAGATAGATTACAATGAAATTAGCGGAAAACTTCCA :  354
FJ708785  : --------------------------------CTATTAAGTGGAAATCAACTGACAGGATCTTTGCCTCAAGAACTCGGTTCTCTTTCCAATCTCTTAATCCTTCAGATAGATTACAATGAAATTAGCGGAAAACTTCCA :  498
                                                                                                                                                               
                     *       860         *       880         *       900         *       920         *       940         *       960         *       980       
Genomic   : ACGTCCTTGGCAAATTTGAAAAAACTGAAGCATTTGTGAGATCTCCTTCTCTCCTACTTATCTTCTCTCTTCCTTTCAAACCCCTCTGACATAACTTTTACTTTCTTTGCTAGTCACATGAACAATAACTCAATCACGGG :  980
NM_123104 : ACGTCCTTGGCAAATTTGAAAAAACTGAAGCATTT------------------------------------------------------------------------------TCACATGAACAATAACTCAATCACGGG :  416
FJ708785  : ACGTCCTTGGCAAATTTGAAAAAACTGAAGCATTT------------------------------------------------------------------------------TCACATGAACAATAACTCAATCACGGG :  560
                                                                                                                                                               
                     *      1000         *      1020         *      1040         *      1060         *      1080         *      1100         *      1120       
Genomic   : TCAGATTCCGCCCGAGTACTCTACCTTAACCAATGTTCTGCACTTGTAAGTTTCTTGGTTACCTTTAAATCTGTTTATCTTATTCAAATCTGATCATCAAGGCTCAACTTGTGAAGCTTGATGGACAATAACAAGCTAAC : 1120
NM_123104 : TCAGATTCCGCCCGAGTACTCTACCTTAACCAATGTTCTGCACTT-----------------------------------------------------------------------CTTGATGGACAATAACAAGCTAAC :  485
FJ708785  : TCAGATTCCGCCCGAGTACTCTACCTTAACCAATGTTCTGCACTT-----------------------------------------------------------------------CTTGATGGACAATAACAAGCTAAC :  629
                                                                                                                                                               
                     *      1140         *      1160         *      1180         *      1200         *      1220         *      1240         *      1260       
Genomic   : TGGTAATCTTCCACCGGAGCTCGCTCAGATGCCAAGCTTGAGAATTCTGTAAGTGTCTTCAACTTCTTATCGAAGACGACTAACTTTCTTCCACATTCAAATTAGTTTGCTTCATAATTTACAGACAATTGGACGGTAGC : 1260
NM_123104 : TGGTAATCTTCCACCGGAGCTCGCTCAGATGCCAAGCTTGAGAATTCT----------------------------------------------------------------------------ACAATTGGACGGTAGC :  549
FJ708785  : TGGTAATCTTCCACCGGAGCTCGCTCAGATGCCAAGCTTGAGAATTCT----------------------------------------------------------------------------ACAATTGGACGGTAGC :  693
                                                                                                                                                               
                     *      1280         *      1300         *      1320         *      1340         *      1360         *      1380         *      1400       
Genomic   : AACTTTGATGGCACAGAGATCCCATCTTCTTACGGATCGATACCAAATCTCGTCAAATTGTAAGAAACTGAACATAGTAAAATCTTATGTTTTCCCTGTGCCGTACTGATTTTGTGTCTCACGCATTAACAGGAGTCTCA : 1400
NM_123104 : AACTTTGATGGCACAGAGATCCCATCTTCTTACGGATCGATACCAAATCTCGTCAAATTG-------------------------------------------------------------------------AGTCTCA :  616
FJ708785  : AACTTTGATGGCACAGAGATCCCATCTTCTTACGGATCGATACCAAATCTCGTCAAATTG-------------------------------------------------------------------------AGTCTCA :  760
                                                                                                                                                               
                     *      1420         *      1440         *      1460         *      1480         *      1500         *      1520         *      1540       
Genomic   : GAAACTGCAACCTAGAAGGACCAATTCCTGATTTGAGCAAATCACTGGTTCTCTATTACTTGTGAGTCACTTTAACAAGAAAAAAAACTTAACAATCCATGTTACATTAGTCTCTAAGGCTTTTTTACTCTTCATGCTTC : 1540
NM_123104 : GAAACTGCAACCTAGAAGGACCAATTCCTGATTTGAGCAAATCACTGGTTCTCTATTACTT------------------------------------------------------------------------------- :  677
FJ708785  : GAAACTGCAACCTAGAAGGACCAATTCCTGATTTGAGCAAATCACTGGTTCTCTATTACTT------------------------------------------------------------------------------- :  821
                                                                                                                                                               
                     *      1560         *      1580         *      1600         *      1620         *      1640         *      1660         *      1680       
Genomic   : AGAGATATTTCCTCCAACAAACTCACAGGAGAAATTCCCAAGAACAAGTTCTCTGCAAACATCACAACTATGTAAGTGCCATGGCCAAGAAATGGAATACAATTGATATGTCGTTACATTTTTGTTGACAAACATGTCTG : 1680
NM_123104 : --AGATATTTCCTCCAACAAACTCACAGGAGAAATTCCCAAGAACAAGTTCTCTGCAAACATCACAACTAT--------------------------------------------------------------------- :  746
FJ708785  : --AGATATTTCCTCCAACAAACTCACAGGAGAAATTCCCAAGAACAAGTTCTCTGCAAACATCACAACTAT--------------------------------------------------------------------- :  890
                                                                                                                                                               
                     *      1700         *      1720         *      1740         *      1760         *      1780         *      1800         *      1820       
Genomic   : CATTTGTTTATTGTATGCAGAAACTTGTACAACAATTTGCTCAGCGGATCCATTCCTTCTAATTTTTCAGGCCTTCCACGGTTACAGAGACTGTAATTAGACTCTCTTTTGCTCTTCTTTTTTTTAGAGAAAGAAAACAA : 1820
NM_123104 : --------------------AAACTTGTACAACAATTTGCTCAGCGGATCCATTCCTTCTAATTTTTCAGGCCTTCCACGGTTACAGAGACTG----------------------------------------------- :  819
FJ708785  : --------------------AAACTTGTACAACAATTTGCTCAGCGGATCCATTCCTTCTAATTTTTCAGGCCTTCCACGGTTACAGAGACTG----------------------------------------------- :  963
                                                                                                                                                               
                     *      1840         *      1860         *      1880         *      1900         *      1920         *      1940         *      1960       
Genomic   : AAAGACATAAGTTTTGTCTTAAATTTTCAGGCAAGTGCAGAACAACAACTTGAGTGGGGAGATTCCTGTGATATGGGAAAATAGGATCTTGAAGGCAGAGGAAAAACTTATCTTGTAAGACACGTCATATCTACAAAACA : 1960
NM_123104 : -------------------------------CAAGTGCAGAACAACAACTTGAGTGGGGAGATTCCTGTGATATGGGAAAATAGGATCTTGAAGGCAGAGGAAAAACTTATCTTG------------------------- :  903
FJ708785  : -------------------------------CAAGTGCAGAACAACAACTTGAGTGGGGAGATTCCTGTGATATGGGAAAATAGGATCTTGAAGGCAGAGGAAAAACTTATCTTG------------------------- : 1047
                                                                                                                                                               
                     *      1980         *      2000         *      2020         *      2040         *      2060         *      2080         *      2100       
Genomic   : CTAGAGAAAACTTAGTAATTGTTGATTGATTTTTGGTTGTAACAACTTGCAGGGACTTGAGGAACAACATGTTTTCAAATGTGTCAAGTGTTCTTCTAAACCCTCCCTCCAATGTCACAGTCAAGTAAGATTATGTTTTG : 2100
NM_123104 : -----------------------------------------------------GACTTGAGGAACAACATGTTTTCAAATGTGTCAAGTGTTCTTCTAAACCCTCCCTCCAATGTCACAGTCAAG--------------- :  975
FJ708785  : -----------------------------------------------------GACTTGAGGAACAACATGTTTTCAAATGTGTCAAGTGTTCTTCTAAACCCTCCCTCCAATGTCACAGTCAAG--------------- : 1119
                                                                                                                                                               
                     *      2120         *      2140         *      2160         *      2180         *      2200         *      2220         *      2240       
Genomic   : AAAAAGAACAAAAGATTAATAATTTGGAATGTTGGATAGAGAAATGAGAGAACTCTGTTTTGATGAGCAGGCTTTACGGAAACCCGGTTTGTGCAAATGTTAATGCGGGAAAGTTAGCTGATCTCTGTGGTATCTCAACG : 2240
NM_123104 : -----------------------------------------------------------------------CTTTACGGAAACCCGGTTTGTGCAAATGTTAATGCGGGAAAGTTAGCTGATCTCTGTGGTATCTCAACG : 1044
FJ708785  : -----------------------------------------------------------------------CTTTACGGAAACCCGGTTTGTGCAAATGTTAATGCGGGAAAGTTAGCTGATCTCTGTGGTATCTCAACG : 1188
                                                                                                                                                               
                     *      2260         *      2280         *      2300         *      2320         *      2340         *      2360         *      2380       
Genomic   : TTAGAAGTGGAATCTCCCGCAACTTCTTCGGAAACCATCTCGACGGGAGACTGCAAACGTCAGTCGTGCCCCGTAAGCGAAAACTATGACTACGTGATTGGGTCTCCAGTTGCATGTTTCTGCGCTGCACCACTTGGCAT : 2380
NM_123104 : TTAGAAGTGGAATCTCCCGCAACTTCTTCGGAAACCATCTCGACGGGAGACTGCAAACGTCAGTCGTGCCCCGTAAGCGAAAACTATGACTACGTGATTGGGTCTCCAGTTGCATGTTTCTGCGCTGCACCACTTGGCAT : 1184
FJ708785  : TTAGAAGTGGAATCTCCCGCAACTTCTTCGGAAACCATCTCGACGGGAGACTGCAAACGTCAGTCGTGCCCCGTAAGCGAAAACTATGACTACGTGATTGGGTCTCCAGTTGCATGTTTCTGCGCTGCACCACTTGGCAT : 1328


                     *      2400         *      2420         *      2440         *      2460         *      2480         *      2500         *      2520       
Genomic   : TGATCTTCGGCTTAGGAGTCCAAGTTTCTCGGACTTTCGACCTTACAAAGTCTCCTACATGCTCGATGTAGCATCCCCGAAAAATCTCGGGATAAACCCGTATCAGATATCTATTGATACATTTGCATGGCAGTCGGGTC : 2520
NM_123104 : TGATCTTCGGCTTAGGAGTCCAAGTTTCTCGGACTTTCGACCTTACAAAGTCTCCTACATGCTCGATGTAGCATCCCCGAAAAATCTCGGGATAAACCCGTATCAGATATCTATTGATACATTTGCATGGCAGTCGGGTC : 1324
FJ708785  : TGATCTTCGGCTTAGGAGTCCAAGTTTCTCGGACTTTCGACCTTACAAAGTCTCCTACATGCTCGATGTAGCATCCCCGAAAAATCTCGGGATAAACCCGTATCAGATATCTATTGATACATTTGCATGGCAGTCGGGTC : 1468
                                                                                                                                                               
                     *      2540         *      2560         *      2580         *      2600         *      2620         *      2640         *      2660       
Genomic   : CAAGGCTATTCATGAACATGAAGATTTTCCCTGAGTACAGTGAATTAAACAGTAAATTCAACAGCACAGAGGTTCAACGTATTGTGGACTTCTTTGCAACTTTCACTCTGAATACTGATGATTCTCTTGGCCCTTATGAG : 2660
NM_123104 : CAAGGCTATTCATGAACATGAAGATTTTCCCTGAGTACAGTGAATTAAACAGTAAATTCAACAGCACAGAGGTTCAACGTATTGTGGACTTCTTTGCAACTTTCACTCTGAATACTGATGATTCTCTTGGCCCTTATGAG : 1464
FJ708785  : CAAGGCTATTCATGAACATGAAGATTTTCCCTGAGTACAGTGAATTAAACAGTAAATTCAACAGCACAGAGGTTCAACGTATTGTGGACTTCTTTGCAACTTTCACTCTGAATACTGATGATTCTCTTGGCCCTTATGAG : 1608
                                                                                                                                                               
                     *      2680         *      2700         *      2720         *      2740         *      2760         *      2780         *      2800       
Genomic   : ATTATTAGTATCAACACTGGTGCTTACAAAGACGGTAATACACATATCTTCTACTCATCTTTGTGCATCAAAAGAGTATTTATTTATGTTACTCCTGTATATGAAGTTACTATCATATTCCCGAAAAAGTCGGGGATGAG : 2800
NM_123104 : ATTATTAGTATCAACACTGGTGCTTACAAAGACGGTAATACACATATCTTCTACTCATCTTTGTGCATCAAAAGAGTATTTATTTATGTTACTCCTGTATATGAAGTTACTATCATATTCCCGAAAAAGTCGGGGATGAG : 1604
FJ708785  : ATTATTAGTATCAACACTGGTGCTTACAAAGACG------------------------------------------------------------------------TTACTATCATATTCCCGAAAAAGTCGGGGATGAG : 1676
                                                                                                                                                               
                     *      2820         *      2840         *      2860         *      2880         *      2900         *      2920         *      2940       
Genomic   : CATAGGCGTCTCGGTTGGAATCATTATAGGAGCCATTGCTTTCTTCCTTGTATTGTCCTCTCTAGCATTGGTCTTCTTCATCAAGAGAAGCAAACGAAAGAGAAAAACTAGGGAAGTCGACATGGAACAAGAACACCCAC : 2940
NM_123104 : CATAGGCGTCTCGGTTGGAATCATTATAGGAGCCATTGCTTTCTTCCTTGTATTGTCCTCTCTAGCATTGGTCTTCTTCATCAAGAGAAGCAAACGAAAGAGAAAAACTAGGGAAGTCGACATGGAACAAGAACACCCAC : 1744
FJ708785  : CATAGGCGTCTCGGTTGGAATCATTATAGGAGCCATTGCTTTCTTCCTTGTATTGTCCTCTCTAGCATTGGTCTTCTTCATCAAGAGAAGCAAACGAAAGAGAAAAACTAGGGAAGTCGACATGGAACAAGAACACCCAC : 1816
                                                                                                                                                               
                     *      2960         *      2980         *      3000         *      3020         *      3040         *      3060         *      3080       
Genomic   : GTAAGTGGAAACTTAGTCAATTTCTCTGTTTTCCTTAAACGTATATCTAATGCTAATCTCCCATGTTTCTCCTTGGCCTAGTTCCAAAACCTCCAATGAACATGGAGAGCGTGAAGGGTTACAATTTCACGGAACTTGAT : 3080
NM_123104 : ---------------------------------------------------------------------------------TTCCAAAACCTCCAATGAACATGGAGAGCGTGAAGGGTTACAATTTCACGGAACTTGAT : 1803
FJ708785  : ---------------------------------------------------------------------------------TTCCAAAACCTCCAATGAACATGGAGAGCGTGAAGGGTTACAATTTCACGGAACTTGAT : 1875
                                                                                                                                                               
                     *      3100         *      3120         *      3140         *      3160         *      3180         *      3200         *      3220       
Genomic   : TCAGCAACAAGCAGTTTCAGTGATCTCTCTCAGATTGGTAGAGGTGGCTATGGGAAGGTCTACAAAGGCCATTTACCAGGTGGTCTGGTTGTAGCTGTCAAACGTGCAGAGCAAGGGTCTCTGCAGGGCCAAAAAGAGTT : 3220
NM_123104 : TCAGCAACAAGCAGTTTCAGTGATCTCTCTCAGATTGGTAGAGGTGGCTATGGGAAGGTCTACAAAGGCCATTTACCAGGTGGTCTGGTTGTAGCTGTCAAACGTGCAGAGCAAGGGTCTCTGCAGGGCCAAAAAGAGTT : 1943
FJ708785  : TCAGCAACAAGCAGTTTCAGTGATCTCTCTCAGATTGGTAGAGGTGGCTATGGGAAGGTCTACAAAGGCCATTTACCAGGTGGTCTGGTTGTAGCTGTCAAACGTGCAGAGCAAGGGTCTCTGCAGGGCCAAAAAGAGTT : 2015
                                                                                                                                                               
                     *      3240         *      3260         *      3280         *      3300         *      3320         *      3340         *      3360       
Genomic   : CTTCACCGAGATCGAGTTGCTTTCCCGGCTACATCACCGTAACCTTGTTTCTTTGCTTGGCTACTGTGATCAGAAAGGAGAACAGGTGAGTCACTTATTAACATTGTAAATTTCAAGATAGTCTTGAGTTGATTTGTTAT : 3360
NM_123104 : CTTCACCGAGATCGAGTTGCTTTCCCGGCTACATCACCGTAACCTTGTTTCTTTGCTTGGCTACTGTGATCAGAAAGGAGAACAG------------------------------------------------------- : 2028
FJ708785  : CTTCACCGAGATCGAGTTGCTTTCCCGGCTACATCACCGTAACCTTGTTTCTTTGCTTGGCTACTGTGATCAGAAAGGAGAACAG------------------------------------------------------- : 2100
                                                                                                                                                               
                     *      3380         *      3400         *      3420         *      3440         *      3460         *      3480         *      3500       
Genomic   : GCCTGATTTCAGATGTTGGTATATGAGTACATGCCCAACGGTTCTCTTCAGGACGCACTCTCTGGTAAGTTCCTACCTTGTCTTGTATATATAAAAGTGTTGTGGGAGGATTGAGATAGATGAATGTGAAACTGCAGCGA : 3500
NM_123104 : ------------ATGTTGGTATATGAGTACATGCCCAACGGTTCTCTTCAGGACGCACTCTCTG-------------------------------------------------------------------------CGA : 2083
FJ708785  : ------------ATGTTGGTATATGAGTACATGCCCAACGGTTCTCTTCAGGACGCACTCTCTG-------------------------------------------------------------------------CGA : 2155
                                                                                                                                                               
                     *      3520         *      3540         *      3560         *      3580         *      3600         *      3620         *      3640       
Genomic   : GGTTTAGACAACCACTGAGTTTGGCGCTGAGACTGAGAATAGCGCTCGGGTCTGCGAGAGGGATTCTGTATCTGCACACAGAAGCTGATCCACCAATCATCCATCGAGACATCAAACCAAGCAACATTCTTCTAGACAGC : 3640
NM_123104 : GGTTTAGACAACCACTGAGTTTGGCGCTGAGACTGAGAATAGCGCTCGGGTCTGCGAGAGGGATTCTGTATCTGCACACAGAAGCTGATCCACCAATCATCCATCGAGACATCAAACCAAGCAACATTCTTCTAGACAGC : 2223
FJ708785  : GGTTTAGACAACCACTGAGTTTGGCGCTGAGACTGAGAATAGCGCTCGGGTCTGCGAGAGGGATTCTGTATCTGCACACAGAAGCTGATCCACCAATCATCCATCGAGACATCAAACCAAGCAACATTCTTCTAGACAGC : 2295
                                                                                                                                                               
                     *      3660         *      3680         *      3700         *      3720         *      3740         *      3760         *      3780       
Genomic   : AAGATGAACCCTAAAGTTGCAGACTTTGGGATCTCAAAGCTCATAGCACTAGATGGTGGAGGAGTGCAAAGGGATCACGTAACTACCATCGTCAAAGGCACTCCTGTATGTTGCTTTAACATTCCTCTGTTTTAAATCCC : 3780
NM_123104 : AAGATGAACCCTAAAGTTGCAGACTTTGGGATCTCAAAGCTCATAGCACTAGATGGTGGAGGAGTGCAAAGGGATCACGTAACTACCATCGTCAAAGGCACTCCTG---------------------------------- : 2329
FJ708785  : AAGATGAACCCTAAAGTTGCAGACTTTGGGATCTCAAAGCTCATAGCACTAGATGGTGGAGGAGTGCAAAGGGATCACGTAACTACCATCGTCAAAGGCACTCCTG---------------------------------- : 2401
                                                                                                                                                               
                     *      3800         *      3820         *      3840         *      3860         *      3880         *      3900         *      3920       
Genomic   : TTAGGGAAGGTGACTAATTCCCGAAACGTATGGTACAGGGATATGTGGATCCAGAGTATTACTTGAGCCACAGGCTAACAGAAAAGAGTGACGTATACAGCCTCGGCATTGTCTTTCTCGAAATTCTCACAGGAATGCGC : 3920
NM_123104 : ---------------------------------------GATATGTGGATCCAGAGTATTACTTGAGCCACAGGCTAACAGAAAAGAGTGACGTATACAGCCTCGGCATTGTCTTTCTCGAAATTCTCACAGGAATGCGC : 2430
FJ708785  : ---------------------------------------GATATGTGGATCCAGAGTATTACTTGAGCCACAGGCTAACAGAAAAGAGTGACGTATACAGCCTCGGCATTGTCTTTCTCGAAATTCTCACAGGAATGCGC : 2502
                                                                                                                                                               
                     *      3940         *      3960         *      3980         *      4000         *      4020         *      4040         *      4060       
Genomic   : CCAATCTCGCACGGAAGAAACATCGTGCGGGAGGTGAATGAAGCATGCGATGCTGGGATGATGATGTCAGTGATAGACAGGAGCATGGGGCAGTACTCTGAGGAATGCGTCAAGAGATTCATGGAACTAGCCATCAGATG : 4060
NM_123104 : CCAATCTCGCACGGAAGAAACATCGTGCGGGAGGTGAATGAAGCATGCGATGCTGGGATGATGATGTCAGTGATAGACAGGAGCATGGGGCAGTACTCTGAGGAATGCGTCAAGAGATTCATGGAACTAGCCATCAGATG : 2570
FJ708785  : CCAATCTCGCACGGAAGAAACATCGTGCGGGAGGTGAATGAAGCATGCGATGCTGGGATGATGATGTCAGTGATAGACAGGAGCATGGGGCAGTACTCTGAGGAATGCGTCAAGAGATTCATGGAACTAGCCATCAGATG : 2642
                                                                                                                                                               
                     *      4080         *      4100         *      4120         *      4140         *      4160         *      4180         *      4200       
Genomic   : TTGTCAGGACAACCCAGAAGCGCGTCCGTGGATGCTGGAAATAGTGAGGGAGCTGGAGAATATCTATGGGCTGATTCCAAAGGAAGAGAAGCCATATTCAAGCCCTTCAGTCCAATCATCTGCTTCAGGAATGTCAGGCT : 4200
NM_123104 : TTGTCAGGACAACCCAGAAGCGCGTCCGTGGATGCTGGAAATAGTGAGGGAGCTGGAGAATATCTATGGGCTGATTCCAAAGGAAGAGAAGCCATATTCAAGCCCTTCAGTCCAATCATCTGCTTCAGGAATGTCAGGCT : 2710
FJ708785  : TTGTCAGGACAACCCAGAAGCGCGTCCGTGGATGCTGGAAATAGTGAGGGAGCTGGAGAATATCTATGGGCTGATTCCAAAGGAAGAGAAGCCATATTCAAGCCCTTCAGTCCAATCATCTGCTTCAGGAATGTCAGGCT : 2782
                                                                                                                                                               
                     *      4220         *      4240         *      4260         *      4280         *      4300         *      4320         *      4340       
Genomic   : TCGCTGTTGCTTCTCCAAGAAGCAGTTATACTACCTTCTCTGAATTCACAGCTAACCAACTCGTCAGCGGAGTCATTCCCTCCATCGCACCGCGC : 4295
NM_123104 : TCGCTGTTGCTTCTCCAAGAAGCAGTTATACTACCTTCTCTGAATTCACAGCTAACCAACTCGTCAGCGGAGTCATTCCCTCCATCGCACCGCGC : 2805
FJ708785  : TCGCTGTTGCTTCTCCAAGAAGCAGTTATACTACCTTCTCTGAATTCACAGCTAACCAACTCGTCAGCGGAGTCATTCCCTCCATCGCACCGCGC : 2877


At5g45840
                                                                                                                                                               
                     *        20         *        40         *        60         *        80         *       100         *       120         *       140       
Genomic   : ATGGGTTGTCGATGGAATCCAATTGGGTTCCAATTCTCTTGCTTCATGTTCTTGATCATTACTCTTCAATCTCGTTCTTCGTTGTCCCTCGAATCCGAAGGTGAACAACTTTGAAATCTCTATCATTGTGTTCTTTTGAA :  140
NM_123952 : ATGGGTTGTCGATGGAATCCAATTGGGTTCCAATTCTCTTGCTTCATGTTCTTGATCATTACTCTTCAATCTCGTTCTTCGTTGTCCCTCGAATCCGAAGG--------------------------------------- :  101
FJ708791  : ATGGGTTGTCGATGGAATCCAATTGGGTTCCAATTCTCTTGCTTCATGTTCTTGATCATTACTCTTCAATCTCGTTCTTCGTTGTCCCTCGAATCCGAAGG--------------------------------------- :  101
                                                                                                                                                               
                     *       160         *       180         *       200         *       220         *       240         *       260         *       280       
Genomic   : TGTTTTTGTTTAAAGGGTTTTTGAATTACGTATTATTGAATGTTTGTAGGATTTGTATTGTTGAAATTCCGGGCAAGAGTTGATTCTGATCCTCATGGAACTCTTGCAAATTGGAATGTTTCTGATCATGATCATTTCTG :  280
NM_123952 : --------------------------------------------------ATTTGTATTGTTGAAATTCCGGGCAAGAGTTGATTCTGATCCTCATGGAACTCTTGCAAATTGGAATGTTTCTGATCATGATCATTTCTG :  191
FJ708791  : --------------------------------------------------ATTTGTATTGTTGAAATTCCGGGCAAGAGTTGATTCTGATCCTCATGGAACTCTTGCAAATTGGAATGTTTCTGATCATGATCATTTCTG :  191
                                                                                                                                                               
                     *       300         *       320         *       340         *       360         *       380         *       400         *       420       
Genomic   : TTCTTGGTTTGGTGTTACGTGTGTCGACAATAAAGTGCAGATGCTGTAAGTAATGTTTTCTTTTGTTGTCTTTGTGATTCTTGTTGCAAGACCTAATGAGTGTATTGGTTTTGTTTTGTAGGAATCTTAGTGGTTGTTCT :  420
NM_123952 : TTCTTGGTTTGGTGTTACGTGTGTCGACAATAAAGTGCAGATGCTG----------------------------------------------------------------------------AATCTTAGTGGTTGTTCT :  255
FJ708791  : TTCTTGGTTTGGTGTTACGTGTGTCGACAATAAAGTGCAGATGCTG----------------------------------------------------------------------------AATCTTAGTGGTTGTTCT :  255
                                                                                                                                                               
                     *       440         *       460         *       480         *       500         *       520         *       540         *       560       
Genomic   : TTGGGAGGAACTTTAGCTCCGGAGCTTAGTCAATTGAGTGAATTAAGATCTCTGTAAGTTTGTTTCAACTAAAATTTTTGCTCTGTTTCTTGAAAGAATAAGAAAAACTAAAACCAATTCTTGTTTATTTTCTTGTAGAA :  560
NM_123952 : TTGGGAGGAACTTTAGCTCCGGAGCTTAGTCAATTGAGTGAATTAAGATCTCT-------------------------------------------------------------------------------------AA :  310
FJ708791  : TTGGGAGGAACTTTAGCTCCGGAGCTTAGTCAATTGAGTGAATTAAGATCTCT-------------------------------------------------------------------------------------AA :  310
                                                                                                                                                               
                     *       580         *       600         *       620         *       640         *       660         *       680         *       700       
Genomic   : TACTATCCAAGAACAAACTCTCTGGTGACATTCCAAACGAATTTGCGAGTTTCGCGAAATTAGAGTTCTTGGATTTGCGAGATAATAACTTAAACGGAGTAGTTCCACCCGAGCTAAATAAAGTGTTGACACCAGAAAAC :  700
NM_123952 : TACTATCCAAGAACAAACTCTCTGGTGACATTCCAAACGAATTTGCGAGTTTCGCGAAATTAGAGTTCTTGGATTTGCGAGATAATAACTTAAACGGAGTAGTTCCACCCGAGCTAAATAAAGTGTTGACACCAGAAAAC :  450
FJ708791  : TACTATCCAAGAACAAACTCTCTGGTGACATTCCAAACGAATTTGCGAGTTTCGCGAAATTAGAGTTCTTGGATTTGCGAGATAATAACTTAAACGGAGTAGTTCCACCCGAGCTAAATAAAGTGTTGACACCAGAAAAC :  450
                                                                                                                                                               
                     *       720         *       740         *       760         *       780         *       800         *       820         *       840       
Genomic   : TTGTGAGTTTTAAAGGCTTGTAGTAGTGCTTTTGATTTAATCCAAAACAATGTGTTGATACAAACTGTTCTGTTTTTTGGTGGATCCAGGTTGCTTTCTGGTAACAAATTTGCGGGTTTTATGACCGTAAAGTTCCTGAG :  840
NM_123952 : TTGT---------------------------------------------------------------------------------------TGCTTTCTGGTAACAAATTTGCGGGTTTTATGACCGTAAAGTTCCTGAG :  503
FJ708791  : TTGT---------------------------------------------------------------------------------------TGCTTTCTGGTAACAAATTTGCGGGTTTTATGACCGTAAAGTTCCTGAG :  503
                                                                                                                                                               
                     *       860         *       880         *       900         *       920         *       940         *       960         *       980       
Genomic   : ACTTCAATCGCTGTATAAAGTCCAGATGAACAAGAATCGAGAGCTATCTTCGGTTTCCGCTGATGTCCTCGACTGTGTCAATAGAAAACTTGGATACTGGTAATAAAAGTTTTAGTCCTTGGATGGAGAAAACTTTTCTT :  980
NM_123952 : ACTTCAATCGCTGTATAAAGTCCAGATGAACAAGAATCGAGAGCTATCTTCGGTTTCCGCTGATGTCCTCGACTGTGTCAATAGAAAACTTGGATACTG----------------------------------------- :  602
FJ708791  : ACTTCAATCGCTGTATAAAGTCCAGATGAACAAGAATCGAGAGCTATCTTCGGTTTCCGCTGATGTCCTCGACTGTGTCAATAGAAAACTTGGATACTG----------------------------------------- :  602
                                                                                                                                                               
                     *      1000         *      1020         *      1040         *      1060         *      1080         *      1100         *      1120       
Genomic   : TGAGTTAACTTGTTTTTAATTTGAATTTTTTTTACAGTGTTTCAAGGAGAAGTTTAATAACAAGAAACAAGGCAAAGGCATTCGTATTGCGGATTAGAGCAACTTCAAGACATTACATGAAAGCATTTTCTTTTATCTCT : 1120
NM_123952 : -------------------------------------------------------------------------------------------------------------------------------------------- :    -
FJ708791  : -------------------------------------TGTTTCAAGGAGAAGTTTAATAACAAGAAACAAGGCAAAGGCATTCGTATTGCGGATTAGAGCAACTTCAAGACATTACAT---------------------- :  683
                                                                                                                                                               
                     *      1140         *      1160         *      1180         *      1200         *      1220         *      1240         *      1260       
Genomic   : CAATACTTTTGGTCTGATTTGTATGTTTTAATCCTCATGTGTTACTGATTTTCTATGTTTTATGTATCTACAGGGTGCGAAGAGAATCTCATGGGAAGAATTATGTAGTGAATTATCACCCAAGTAAGGCTAAAAGATTA : 1260
NM_123952 : -------------------------------------------------------------------------GGTGCGAAGAGAATCTCATGGGAAGAATTATGTAGTGAATTATCACCCAAGT--------------- :  654
FJ708791  : -------------------------------------------------------------------------GGTGCGAAGAGAATCTCATGGGAAGAATTATGTAGTGAATTATCACCCAAGT--------------- :  735
                                                                                                                                                               
                     *      1280         *      1300         *      1320         *      1340         *      1360         *      1380         *      1400       
Genomic   : CTACTCCAAGCTCTGCAATTGGAAACACACAAAACTCATAACTGTCAATCTTATACCTTTGTTTTTTATGCAGGTGAGAACGAAACTAGTATCTTCAAAAGACGTGAGTTACTCGAGGAAACAAGCAATTTAGCGGCTAT : 1400
NM_123952 : ---------------------------------------------------------------------------GAGAACGAAACTAGTATCTTCAAAAGACGTGAGTTACTCGAGGAAACAAGCAATTTAGCGGCTAT :  719
FJ708791  : ---------------------------------------------------------------------------GAGAACGAAACTAGTATCTTCAAAAGACGTGAGTTACTCGAGGAAACAAGCAATTTAGCGGCTAT :  800
                                                                                                                                                               
                     *      1420         *      1440         *      1460         *      1480         *      1500         *      1520         *      1540       
Genomic   : GCCTGCGCCTGATACGCCCAGTCCTTCTCCTGAGATTATAACTATAGTGTTTCCTCGAAGCAGCGGGTCGTTTCCAGCATTAACTAATGCAAAGAAGAGAATACCTCCATTGATCCCTCCTTCTTCTCCTCCTCCTCTAC : 1540
NM_123952 : GCCTGCGCCTGATACGCCCAGTCCTTCTCCTGAGATTATAACTATAGTGTTTCCTCGAAGCAGCGGGTCGTTTCCAGCATTAACTAATGCAAAGAAGAGAATACCTCCATTGATCCCTCCTTCTTCTCCTCCTCCTCTAC :  859
FJ708791  : GCCTGCGCCTGATACGCCCAGTCCTTCTCCTGAGATTATAACTATAGTGTTTCCTCGAAGCAGCGGGTCGTTTCCAGCATTAACTAATGCAAAGAAGAGAATACCTCCATTGATCCCTCCTTCTTCTCCTCCTCCTCTAC :  940
                                                                                                                                                               
                     *      1560         *      1580         *      1600         *      1620         *      1640         *      1660         *      1680       
Genomic   : CTACCAACAACACCATCGCTAGTGATCCACCGAGGAAATTCGAAGAAAAATCGAAAGGGTTTAAGGACGTTTGGTTGTATGTTGTGATCGGTGTTGCTGCTTTCGTAGCGATGCTGATAATAGTAGCGGTTATATTCTTC : 1680
NM_123952 : CTACCAACAACACCATCGCTAGTGATCCACCGAGGAAATTCGAAGAAAAATCGAAAGGGTTTAAGGACGTTTGGTTGTATGTTGTGATCGGTGTTGCTGCTTTCGTAGCGATGCTGATAATAGTAGCGGTTATATTCTTC :  999
FJ708791  : CTACCAACAACACCATCGCTAGTGATCCACCGAGGAAATTCGAAGAAAAATCGAAAGGGTTTAAGGACGTTTGGTTGTATGTTGTGATCGGTGTTGCTGCTTTCGTAGCGATGCTGATAATAGTAGCGGTTATATTCTTC : 1080
                                                                                                                                                               
                     *      1700         *      1720         *      1740         *      1760         *      1780         *      1800         *      1820       
Genomic   : TTCCGGAAAAGAGCTGTGAAGAGTATAGGTCCATGGAAGACTGGTTTGAGTGGACAATTGCAGAAAGCTTTTGTTACTGGTAAAAATATTTGTTAAAACAAACTGATTTTTGCTCTGCTTTTCGGTTTAACATTAATGTG : 1820
NM_123952 : TTCCGGAAAAGAGCTGTGAAGAGTATAGGTCCATGGAAGACTGGTTTGAGTGGACAATTGCAGAAAGCTTTTGTTACTGGT----------------------------------------------------------- : 1080
FJ708791  : TTCCGGAAAAGAGCTGTGAAGAGTATAGGTCCATGGAAGACTGGTTTGAGTGGACAATTGCAGAAAGCTTTTGTTACTGGT----------------------------------------------------------- : 1161
                                                                                                                                                               
                     *      1840         *      1860         *      1880         *      1900         *      1920         *      1940         *      1960       
Genomic   : AGAAATGTTTTCAGGTGTACCTAAGCTAAACCGGTCTGAACTAGAAACAGCCTGTGAAGATTTCAGTAATATCATTGAAGCATTTGATGGTTACACTGTCTATAAAGGAACTTTGTCCAGTGGCGTTGAGATTGCGGTTG : 1960
NM_123952 : ----------------GTACCTAAGCTAAACCGGTCTGAACTAGAAACAGCCTGTGAAGATTTCAGTAATATCATTGAAGCATTTGATGGTTACACTGTCTATAAAGGAACTTTGTCCAGTGGCGTTGAGATTGCGGTTG : 1204
FJ708791  : ----------------GTACCTAAGCTAAACCGGTCTGAACTAGAAACAGCCTGTGAAGATTTCAGTAATATCATTGAAGCATTTGATGGTTACACTGTCTATAAAGGAACTTTGTCCAGTGGCGTTGAGATTGCGGTTG : 1285
                                                                                                                                                               
                     *      1980         *      2000         *      2020         *      2040         *      2060         *      2080         *      2100       
Genomic   : CTTCAACCGCCATTTTGGAAACTAGGGAATGGACAAGAGCTATGGAAATGACTTACCGCAGAAGGGTAATTAAAATAAACAGTTTTTCTTCGACCATTTTTACCGCAAAACTTGACTCATGTTTTGAGAGCTTATATTTG : 2100
NM_123952 : CTTCAACCGCCATTTTGGAAACTAGGGAATGGACAAGAGCTATGGAAATGACTTACCGCAGAAGG--------------------------------------------------------------------------- : 1269
FJ708791  : CTTCAACCGCCATTTTGGAAACTAGGGAATGGACAAGAGCTATGGAAATGACTTACCGCAGAAGG--------------------------------------------------------------------------- : 1350
                                                                                                                                                               
                     *      2120         *      2140         *      2160         *      2180         *      2200         *      2220         *      2240       
Genomic   : CAGATTGATACAATGTCAAGAGTCAACCATAAGAACTTTATTAATCTGATTGGTTATTGCGAAGAAGATGAACCGTTTAATAGGATGATGGTTTTCGAATATGCTCCAAATGGAACTCTTTTCGAACATTTGCATGGTTA : 2240
NM_123952 : ---ATTGATACAATGTCAAGAGTCAACCATAAGAACTTTATTAATCTGATTGGTTATTGCGAAGAAGATGAACCGTTTAATAGGATGATGGTTTTCGAATATGCTCCAAATGGAACTCTTTTCGAACATTTGCATG---- : 1402
FJ708791  : ---ATTGATACAATGTCAAGAGTCAACCATAAGAACTTTATTAATCTGATTGGTTATTGCGAAGAAGATGAACCGTTTAATAGGATGATGGTTTTCGAATATGCTCCAAATGGAACTCTTTTCGAACATTTGCATG---- : 1483
                                                                                                                                                               
                     *      2260         *      2280         *      2300         *      2320         *      2340         *      2360         *      2380       
Genomic   : GTAATATACATAAGTATGCCTCTACATCAAGATCTTGTTAGGCTTATTACGATTATCACCAACCAATCTATATCTTCTTGTTCAGATAAGGAAATGGAGCATCTTGATTGGAACGCGAGGACGAGGATAATAATGGGAAC : 2380
NM_123952 : -------------------------------------------------------------------------------------ATAAGGAAATGGAGCATCTTGATTGGAACGCGAGGACGAGGATAATAATGGGAAC : 1457
FJ708791  : -------------------------------------------------------------------------------------ATAAGGAAATGGAGCATCTTGATTGGAACGCGAGGACGAGGATAATAATGGGAAC : 1538
                    
                                                                                                                                           
                     *      2400         *      2420         *      2440         *      2460         *      2480         *      2500         *      2520       
Genomic   : TGCTTATTGTCTGCAATATATGCACGAGCTCAATCCTCCAATCTCACACACTAAACTTGTCTCATCAGCAATATACTTAACCGATGATTACGCAGCAAAGGTTCTTACTTTCTTGCTTAACAAGAAAACTGTTTTAGTAG : 2520
NM_123952 : TGCTTATTGTCTGCAATATATGCACGAGCTCAATCCTCCAATCTCACACACTAAACTTGTCTCATCAGCAATATACTTAACCGATGATTACGCAGCAAAGGT-------------------------------------- : 1559
FJ708791  : TGCTTATTGTCTGCAATATATGCACGAGCTCAATCCTCCAATCTCACACACTAAACTTGTCTCATCAGCAATATACTTAACCGATGATTACGCAGCAAAGGT-------------------------------------- : 1640
                                                                                                                                                               
                     *      2540         *      2560         *      2580         *      2600         *      2620         *      2640         *      2660       
Genomic   : TTATTTCTTTCTTGCTTAACAAGAAACTGGTGTTCTTGATTCTTAATACAAGACCTAGTGGTAAAATAATGCTTCGTTTTGCAGGTCGGAGAGGTTCCTTTCAGCGGACAAACCGGGAGTAAACCGAGAAAACCGATGAG : 2660
NM_123952 : --------------------------------------------------------------------------------------CGGAGAGGTTCCTTTCAGCGGACAAACCGGGAGTAAACCGAGAAAACCGATGAG : 1613
FJ708791  : --------------------------------------------------------------------------------------CGGAGAGGTTCCTTTCAGCGGACAAACCGGGAGTAAACCGAGAAAACCGATGAG : 1694
                                                                                                                                                               
                     *      2680         *      2700         *      2720         *      2740         *      2760         *      2780         *      2800       
Genomic   : TGGTGATTTAGACCAATCTTTATTGCCATTACCTCCTGAACCAGAGACTAATGTCTATAGCTTTGGAGTATTAATGCTTGAGATAATCTCTGGAAAGCTCTCAGATTCAGAAGAAGAAGGATCAATTCTAAAATGGGTAA : 2800
NM_123952 : TGGTGATTTAGACCAATCTTTATTGCCATTACCTCCTGAACCAGAGACTAATGTCTATAGCTTTGGAGTATTAATGCTTGAGATAATCTCTGGAAAGCTCTCAGATTCAGAAGAAGAAGGATCAATTCTAAAATGGG--- : 1750
FJ708791  : TGGTGATTTAGACCAATCTTTATTGCCATTACCTCCTGAACCAGAGACTAATGTCTATAGCTTTGGAGTATTAATGCTTGAGATAATCTCTGGAAAGCTCTCAGATTCAGAAGAAGAAGGATCAATTCTAAAATGGG--- : 1831
                                                                                                                                                               
                     *      2820         *      2840         *      2860         *      2880         *      2900         *      2920         *      2940       
Genomic   : AAAAAAACTCAAAAATCAAAACCAAATCACAATATTTCTTAAACTTATATTACTAACGTAATACATTTTGTGGATTTGTAATAGGCGTCAAAGTATCTGGAGAATGATAATTTGAGAGATATGATTGATCCTACACTAAC : 2940
NM_123952 : -------------------------------------------------------------------------------------CGTCAAAGTATCTGGAGAATGATAATTTGAGAGATATGATTGATCCTACACTAAC : 1805
FJ708791  : -------------------------------------------------------------------------------------CGTCAAAGTATCTGGAGAATGATAATTTGAGAGATATGATTGATCCTACACTAAC : 1886
                                                                                                                                                               
                     *      2960         *      2980         *      3000         *      3020         *      3040         *      3060         *      3080       
Genomic   : AACGTATAAAGAAGAAGAACTTGAAGCTATATGTGACGTGGCACGACATTGTCTGAAACTTGACGAAAGCCAAAGACCAAAAATGAAATATGTGGTTCAACAATTGAAAGAAGTAATAAACATATCTCAAGAACAAGCAA : 3080
NM_123952 : AACGTATAAAGAAGAAGAACTTGAAGCTATATGTGACGTGGCACGACATTGTCTGAAACTTGACGAAAGCCAAAGACCAAAAATGAAATATGTGGTTCAACAATTGAAAGAAGTAATAAACATATCTCAAGAACAAGCAA : 1945
FJ708791  : AACGTATAAAGAAGAAGAACTTGAAGCTATATGTGACGTGGCACGACATTGTCTGAAACTTGACGAAAGCCAAAGACCAAAAATGAAATATGTGGTTCAACAATTGAAAGAAGTAATAAACATATCTCAAGAACAAGCAA : 2026
                                                                                                                                                               
                     *      3100         *      3120         *                
Genomic   : CACCAAGACTCTCTCCTCTTTGGTGGGCAGAGCTTGAGATCTTATCCTCTGAAGCTACA : 3139
NM_123952 : CACCAAGACTCTCTCCTCTTTGGTGGGCAGAGCTTGAGATCTTATCCTCTGAAGCTACA : 2004
FJ708791  : CACCAAGACTCTCTCCTCTTTGGTGGGCAGAGCTTGAGATCTTATCCTCTGAAGCTACA : 2085


At5g59650
                                                                                                                                                               
                     *        20         *        40         *        60         *        80         *       100         *       120         *       140       
Genomic   : ATGGATAGTCCTTGTTGGCTTTTGCTGCTGCTATTAGGAGCTTTTGCCATTATTGGTTGTGTTCAAGCTCAGGATCAACAAGGTACCTAAATCTTAATGGTTTATATGTATATTACACTACTACTTCTTACTGATTCGAG :  140
NM_125357 : ATGGATAGTCCTTGTTGGCTTTTGCTGCTGCTATTAGGAGCTTTTGCCATTATTGGTTGTGTTCAAGCTCAGGATCAACAAG---------------------------------------------------------- :   82
FJ708806  : ATGGATAGTCCTTGTTGGCTTTTGCTGCTGCTATTAGGAGCTTTTGCCATTATTGGTTGTGTTCAAGCTCAGGATCAACAAG---------------------------------------------------------- :   82
                                                                                                                                                               
                     *       160         *       180         *       200         *       220         *       240         *       260         *       280       
Genomic   : TCTTAGTTATGTTTGTATCTGAGATGCTTTTGATTTTTCACTCTCTTAACAGAGTTTATCAGTTTGGATTGCGGGTTACCCATGACTGAACCGTCTTCTTATACTGAGTCAGTAACCGGACTACGGTTCTCGTCTGATGC :  280
NM_125357 : ----------------------------------------------------AGTTTATCAGTTTGGATTGCGGGTTACCCATGACTGAACCGTCTTCTTATACTGAGTCAGTAACCGGACTACGGTTCTCGTCTGATGC :  170
FJ708806  : ----------------------------------------------------AGTTTATCAGTTTGGATTGCGGGTTACCCATGACTGAACCGTCTTCTTATACTGAGTCAGTAACCGGACTACGGTTCTCGTCTGATGC :  170
                                                                                                                                                               
                     *       300         *       320         *       340         *       360         *       380         *       400         *       420       
Genomic   : TGAATTCATCCAGACTGGTGAAAGTGGTAAAATCCAGGCGAGTATGGAGAATGACTACCTTAAGCCGTACACGAGGCTGAGGTATTTTCCAGAGGAGAGAAGGAACTGCTATAGTTTGAGTGTTGACAAGAACAGGAAAT :  420
NM_125357 : TGAATTCATCCAGACTGGTGAAAGTGGTAAAATCCAGGCGAGTATGGAGAATGACTACCTTAAGCCGTACACGAGGCTGAGGTATTTTCCAGAGGAGAGAAGGAACTGCTATAGTTTGAGTGTTGACAAGAACAGGAAAT :  310
FJ708806  : TGAATTCATCCAGACTGGTGAAAGTGGTAAAATCCAGGCGAGTATGGAGAATGACTACCTTAAGCCGTACACGAGGCTGAGGTATTTTCCAGAGGAGAGAAGGAACTGCTATAGTTTGAGTGTTGACAAGAACAGGAAAT :  310
                                                                                                                                                               
                     *       440         *       460         *       480         *       500         *       520         *       540         *       560       
Genomic   : ATTTGATCAGGGCTAGGTTCATTTATGGGAATTACGATGGTCGTAACTCTAACCCGATATTTGAACTGCATCTAGGACCTAATCTGTGGGCAACTATAGATTTGCAAAAGTTTGTGAATGGTACAATGGAGGAGATCCTT :  560
NM_125357 : ATTTGATCAGGGCTAGGTTCATTTATGGGAATTACGATGGTCGTAACTCTAACCCGATATTTGAACTGCATCTAGGACCTAATCTGTGGGCAACTATAGATTTGCAAAAGTTTGTGAATGGTACAATGGAGGAGATCCTT :  450
FJ708806  : ATTTGATCAGGGCTAGGTTCATTTATGGGAATTACGATGGTCGTAACTCTAACCCGATATTTGAACTGCATCTAGGACCTAATCTGTGGGCAACTATAGATTTGCAAAAGTTTGTGAATGGTACAATGGAGGAGATCCTT :  450
                                                                                                                                                               
                     *       580         *       600         *       620         *       640         *       660         *       680         *       700       
Genomic   : CACACTCCAACATCAAACTCTTTGAACGTTTGTCTTGTTAAGACAGGGACAACTACACCCTTGATCTCAGCCTTGGAATTACGGCCATTAGGAAATAATTCTTATCTCACAGATGGTTCTTTGAATCTTTTCGTTCGGAT :  700
NM_125357 : CACACTCCAACATCAAACTCTTTGAACGTTTGTCTTGTTAAGACAGGGACAACTACACCCTTGATCTCAGCCTTGGAATTACGGCCATTAGGAAATAATTCTTATCTCACAGATGGTTCTTTGAATCTTTTCGTTCGGAT :  590
FJ708806  : CACACTCCAACATCAAACTCTTTGAACGTTTGTCTTGTTAAGACAGGGACAACTACACCCTTGATCTCAGCCTTGGAATTACGGCCATTAGGAAATAATTCTTATCTCACAGATGGTTCTTTGAATCTTTTCGTTCGGAT :  590
                                                                                                                                                               
                     *       720         *       740         *       760         *       780         *       800         *       820         *       840       
Genomic   : ATATCTCAACAAGACAGATGGCTTTCTTCGGTAAGTGTTTTTTTTTTGGTTAACAGTGATGGATTCTATATTCCTATACTCTTTATTTTTAACATTCCTATACTTATTCAAGGATAATACAACCTTTGTTCCACATAGGT :  840
NM_125357 : ATATCTCAACAAGACAGATGGCTTTCTTCGGTA----------------------------------------------------------------------------------------------------------- :  623
FJ708806  : ATATCTCAACAAGACAGATGGCTTTCTTCGGTA----------------------------------------------------------------------------------------------------------- :  623
                                                                                                                                                               
                     *       860         *       880         *       900         *       920         *       940         *       960         *       980       
Genomic   : ACCCGGATGATATTTATGATCGCAGATGGCATAATTACTTTATGGTGGATGATTGGACCCAAATTTTCACCACTCTCGAAGTGACCAATGATAACAATTATGAACCACCAAAAAAGGCTCTTGCAGCGGCTGCCACGCCT :  980
NM_125357 : -CCCGGATGATATTTATGATCGCAGATGGCATAATTACTTTATGGTGGATGATTGGACCCAAATTTTCACCACTCTCGAAGTGACCAATGATAACAATTATGAACCACCAAAAAAGGCTCTTGCAGCGGCTGCCACGCCT :  762
FJ708806  : -CCCGGATGATATTTATGATCGCAGATGGCATAATTACTTTATGGTGGATGATTGGACCCAAATTTTCACCACTCTCGAAGTGACCAATGATAACAATTATGAACCACCAAAAAAGGCTCTTGCAGCGGCTGCCACGCCT :  762
                                                                                                                                                               
                     *      1000         *      1020         *      1040         *      1060         *      1080         *      1100         *      1120       
Genomic   : TCTAATGCCAGCGCGCCATTGACAATCAGTTGGCCACCAGACAATCCTGGTGACCAATATTACTTGTACAGCCACTTCTCTGAGATACAAGATTTACAGACCAATGATACCAGAGAATTTGACATATTATGGGATGGAGC : 1120
NM_125357 : TCTAATGCCAGCGCGCCATTGACAATCAGTTGGCCACCAGACAATCCTGGTGACCAATATTACTTGTACAGCCACTTCTCTGAGATACAAGATTTACAGACCAATGATACCAGAGAATTTGACATATTATGGGATGGAGC :  902
FJ708806  : TCTAATGCCAGCGCGCCATTGACAATCAGTTGGCCACCAGACAATCCTGGTGACCAATATTACTTGTACAGCCACTTCTCTGAGATACAAGATTTACAGACCAATGATACCAGAGAATTTGACATATTATGGGATGGAGC :  902
                                                                                                                                                               
                     *      1140         *      1160         *      1180         *      1200         *      1220         *      1240         *      1260       
Genomic   : TGTTGTTGAAGAAGGTTTCATCCCTCCAAAGTTAGGGGTGACTACTATCCATAATCTTTCTCCAGTGACATGCAAAGGAGAAAATTGCATTTACCAGCTAATAAAAACCTCGAGGTCAACTCTTCCTTCTCTACTTAACG : 1260
NM_125357 : TGTTGTTGAAGAAGGTTTCATCCCTCCAAAGTTAGGGGTGACTACTATCCATAATCTTTCTCCAGTGACATGCAAAGGAGAAAATTGCATTTACCAGCTAATAAAAACCTCGAGGTCAACTCTTCCTTCTCTACTTAACG : 1042
FJ708806  : TGTTGTTGAAGAAGGTTTCATCCCTCCAAAGTTAGGGGTGACTACTATCCATAATCTTTCTCCAGTGACATGCAAAGGAGAAAATTGCATTTACCAGCTAATAAAAACCTCGAGGTCAACTCTTCCTTCTCTACTTAACG : 1042
                                                                                                                                                               
                     *      1280         *      1300         *      1320         *      1340         *      1360         *      1380         *      1400       
Genomic   : CTCTTGAAATCTACACAGTTATCCAATTTCCACGGTCGGAAACAAATGAAAATGATGGTATGTTACTTATCTTCATTGTTATGCAGATACAGTCTGTTTTTTTTTTTTAACTAGAGAAATCAACTTCACCTGCTAATTCT : 1400
NM_125357 : CTCTTGAAATCTACACAGTTATCCAATTTCCACG----------------------------------------------------------------------------------AAATCAACTTCACCTGCTAATTCT : 1100
FJ708806  : CTCTTGAAATCTACACAGTTATCCAATTTCCACGGTCGGAAACAAATGAAAATGAT------------------------------------------------------------------------------------ : 1098
[truncated: 55,003 more chars]
