# Supplementary material for: Genome-wide cloning and sequence analysis of leucine-rich repeat receptor-like protein kinase genes in Arabidopsis thaliana
Source: BMC Genomics. 2010 Jan 11;11:19. doi: 10.1186/1471-2164-11-19 (PMC2817689; doi:10.1186/1471-2164-11-19)
Supplement: Additional file 4 — Sequence alignments of isolated LRR-RLKs showing different coding sequences but not containing one continuous ORF. Corresponding genomic DNA sequences, predicted mRNA sequences, previously reported cDNA sequences (if available), and isolated cDNA sequences obtained from this report for each LRR-RLK were aligned. Sequences with differences are indicated with red boxes. [file 1471-2164-11-19-S4.RTF]

At1g06840
                                                                                                                                                               
                     *        20         *        40         *        60         *        80         *       100         *       120         *       140       
Genomic   : ATGGTTTTGACGGAAGAAGGTGGTGAAGTTATGGCGGCGGCGCAACGGAAACTTATGATGACGCTCTTCCGTCGGTCTCATCCATTACCTGAAATAGTCAAACTTAGAAAATGTCGTATTATCACTCTTCAATGCTCCCA :  140
NM_100561 : ATGGTTTTGACGGAAGAAGGTGGTGAAGTTATGGCGGCGGCGCAACGGAAACTTATGATGACGCTCTTCCGTCG------------------------------------------------------------------ :   74
FJ708626  : ATGGTTTTGACGGAAGAAGGTGGTGAAGTTATGGCGGCGGCGCAACGGAAACTTATGATGACGCTCTTCCGTCGGTCTCATCCATTACCTGAAATAGTCAAACTTAGAAAATGTCGTATTATCACTCTTCAATGCTCCCA :  140
                                                                                                                                                               
                     *       160         *       180         *       200         *       220         *       240         *       260         *       280       
Genomic   : CCCAATACCAACTACTTTCCTCTTTCTTTGTCTTGTGTTTCTTACTTGGTCATGGCCTTTTCCTTCTAAACTCTCTCTCTCTCGAGATTTCGTTTTTTCCTTGGGTCTCCTCTGTTTCCTCCTCTCTCGATATGTTTTCG :  280
NM_100561 : -------------------------------------------------------------------------------------------------------------------------------------------- :    -
FJ708626  : CCCAATACCAACTACTTTCCTCTTTCTTTGTCTTGTGTTTCTTACTTGGTCATGGCCTTTTCCTTCTAAACTCTCTCTCTCTCGAGATTTCGTTTTTTCCTTGGGTCTCCTCTGTTTCCTCCTCTCTCGATATGTTTTCG :  280
                                                                                                                                                               
                     *       300         *       320         *       340         *       360         *       380         *       400         *       420       
Genomic   : ACCCATCATGTCTCTCGTCTCCTCATACCTCTGCTCTTCTTCTTCCTCTTCTGTTGCTTTTCTTCTACTTTTGCTCAAGACGACATCACCAATCCCGTTGAAGGTTTCTTTTTTGTTGTTGTTGTTGTTAAAAGGCTTTG :  420
NM_100561 : -------------------------------------------------------------------------------------------------------------------------------------------- :    -
FJ708626  : ACCCATCATGTCTCTCGTCTCCTCATACCTCTGCTCTTCTTCTTCCTCTTCTGTTGCTTTTCTTCTACTTTTGCTCAAGACGACATCACCAATCCCGTTGAAG------------------------------------- :  383
                                                                                                                                                               
                     *       440         *       460         *       480         *       500         *       520         *       540         *       560       
Genomic   : TCTTTGGTGTTAGATCTTCAGACAATTTGTGTCTGTGACAATGGGTTTTCTTAGCTTTCGACTGATTTGGTACTGATCATTGATGTTTTTTTTGGTTTTAGTGAGGGCTTTGCGAGTGATCAAAGAAAGTTTGAACGATC :  560
NM_100561 : ---------------------------------------------------------------------------------------------------------GGCTTTGCGAGTGATCAAAGAAAGTTTGAACGATC :  109
FJ708626  : -----------------------------------------------------------------------------------------------------TGAGGGCTTTGCGAGTGATCAAAGAAAGTTTGAACGATC :  422
                                                                                                                                                               
                     *       580         *       600         *       620         *       640         *       660         *       680         *       700       
Genomic   : CTGTTCATAGATTGAGAAATTGGAAGCATGGAGACCCGTGCAATTCGAATTGGACTGGTGTTGTCTGCTTCAACTCCACTCTTGATGATGGTTATCTTCATGTCAGCGAATTGTATTGTCTTCTTCACTCTTGCTTTTTT :  700
NM_100561 : CTGTTCATAGATTGAGAAATTGGAAGCATGGAGACCCGTGCAATTCGAATTGGACTGGTGTTGTCTGCTTCAACTCCACTCTTGATGATGGTTATCTTCATGTCAGCGAATTG--------------------------- :  222
FJ708626  : CTGTTCATAGATTGAGAAATTGGAAGCATGGAGACCCGTGCAATTCGAATTGGACTGGTGTTGTCTGCTTCAACTCCACTCTTGATGATGGTTATCTTCATGTCAGCGAATTG--------------------------- :  535
                                                                                                                                                               
                     *       720         *       740         *       760         *       780         *       800         *       820         *       840       
Genomic   : CTTTTATTTGGTCTTCCTTGAGATTTGTTTGAGTTAGCTTTGTTTATACTTGTAGGCAATTGTTCAGTATGAATCTCTCAGGGAACTTGTCTCCGGAGCTTGGCCGGTTGTCTCGTCTTACTATCCTGTACGTGTTTTTA :  840
NM_100561 : --------------------------------------------------------CAATTGTTCAGTATGAATCTCTCAGGGAACTTGTCTCCGGAGCTTGGCCGGTTGTCTCGTCTTACTATCCTG------------ :  294
FJ708626  : --------------------------------------------------------CAATTGTTCAGTATGAATCTCTCAGGGAACTTGTCTCCGGAGCTTGGCCGGTTGTCTCGTCTTACTATCCTG------------ :  607
                                                                                                                                                               
                     *       860         *       880         *       900         *       920         *       940         *       960         *       980       
Genomic   : ACACGATATGTTGCTTGATCTTTTGTCATACTCAATGGTGAATAACTTTCTTTTTGTATTGTACAACTTCTTCTCTAATGCCAGGAGTTTCATGTGGAATAAGATCACCGGAAGTATACCAAAGGAAATTGGGAATATCA :  980
NM_100561 : -------------------------------------------------------------------------------------AGTTTCATGTGGAATAAGATCACCGGAAGTATACCAAAGGAAATTGGGAATATCA :  349
FJ708626  : -------------------------------------------------------------------------------------AGTTTCATGTGGAATAAGATCACCGGAAGTATACCAAAGGAAATTGGGAATATCA :  662
                                                                                                                                                               
                     *      1000         *      1020         *      1040         *      1060         *      1080         *      1100         *      1120       
Genomic   : AGTCCTTAGAACTCTTGTTAGTCCAGGACTTTGTTGTTTACTTCCCTGCTTTATTTGCTTTGGGATTGGCTGCTAACTTCACTTTTCCAGGCTCCTGAATGGAAATCTGTTAAATGGAAACTTACCAGAGGAGCTAGGGT : 1120
NM_100561 : AGTCCTTAGAACTCTT--------------------------------------------------------------------------GCTCCTGAATGGAAATCTGTTAAATGGAAACTTACCAGAGGAGCTAGGGT :  415
FJ708626  : AGTCCTTAGAACTCTT--------------------------------------------------------------------------GCTCCTGAATGGAAATCTGTTAAATGGAAACTTACCAGAGGAGCTAGGGT :  728
                                                                                                                                                               
                     *      1140         *      1160         *      1180         *      1200         *      1220         *      1240         *      1260       
Genomic   : TTCTTCCAAACTTGGACAGAATACAGATTGATGAAAACCGTATATCAGGACCACTTCCCAAATCTTTTGCAAACTTAAACAAAACGAAGCACTTGTGAGTTCTTTAATCTGGTGTCGAGTTGTACTTGGCTGTTTCAATA : 1260
NM_100561 : TTCTTCCAAACTTGGACAGAATACAGATTGATGAAAACCGTATATCAGGACCACTTCCCAAATCTTTTGCAAACTTAAACAAAACGAAGCACTT---------------------------------------------- :  509
FJ708626  : TTCTTCCAAACTTGGACAGAATACAGATTGATGAAAACCGTATATCAGGACCACTTCCCAAATCTTTTGCAAACTTAAACAAAACGAAGCACTT---------------------------------------------- :  822
                                                                                                                                                               
                     *      1280         *      1300         *      1320         *      1340         *      1360         *      1380         *      1400       
Genomic   : TGGTGATTCCTTCTCCCCTTGAATATGTTACATTTATTGCTTTTTTTTGGTCAATCTTATCTTTTTCCAGTCACATGAACAATAATTCTATTAGCGGGCAGATACCACCAGAGCTTGGAAGCCTACCATCCATTGTTCAC : 1400
NM_100561 : ----------------------------------------------------------------------TCACATGAACAATAATTCTATTAGCGGGCAGATACCACCAGAGCTTGGAAGCCTACCATCCATTGTTCAC :  579
FJ708626  : ----------------------------------------------------------------------TCACATGAACAATAATTCTATTAGCGGGCAGATACCACCAGAGCTTGGAAGCCTACCATCCATTGTTCAC :  892
                                                                                                                                                               
                     *      1420         *      1440         *      1460         *      1480         *      1500         *      1520         *      1540       
Genomic   : ATGTGAGTTGGTAGCAGTAGCAGGCTTAAATGATTTTACTTGAAAAATGCTATGTTTTTTACCTAAACTCAGTTTTCATGTCTAGAACATGCTACACACGCTTTACCTATTCAATGTTTATGAACATTTACTTTTTTTTT : 1540
NM_100561 : AT------------------------------------------------------------------------------------------------------------------------------------------ :  581
FJ708626  : AT------------------------------------------------------------------------------------------------------------------------------------------ :  894
                                                                                                                                                               
                     *      1560         *      1580         *      1600         *      1620         *      1640         *      1660         *      1680       
Genomic   : TCTTTTTTCTTACAGCCTTCTTGATAACAACAATTTATCAGGCTATCTTCCCCCTGAGTTATCAAACATGCCGCGTTTACTTATCCTGTAAGTTTATGTCACAGTTTCTGTTGATTTCCCTGGTCCTGTCACCATAAGGA : 1680
NM_100561 : ---------------CCTTCTTGATAACAACAATTTATCAGGCTATCTTCCCCCTGAGTTATCAAACATGCCGCGTTTACTTATCCT----------------------------------------------------- :  653
FJ708626  : ---------------CCTTCTTGATAACAACAATTTATCAGGCTATCTTCCCCCTGAGTTATCAAACATGCCGCGTTTACTTATCCT----------------------------------------------------- :  966
                                                                                                                                                               
                     *      1700         *      1720         *      1740         *      1760         *      1780         *      1800         *      1820       
Genomic   : CTGGTTACCCATGTGTTTCTTCTTTTCTTTTTTTTGACCTTTTTCCTCATGTTTATTTTGGCAGACAATTAGATAACAATCACTTTGACGGGACTACGATTCCACAATCTTATGGAAACATGTCTAAACTTTTGAAGATG : 1820
NM_100561 : ----------------------------------------------------------------ACAATTAGATAACAATCACTTTGACGGGACTACGATTCCACAATCTTATGGAAACATGTCTAAACTTTTGAAGAT- :  728
FJ708626  : ----------------------------------------------------------------ACAATTAGATAACAATCACTTTGACGGGACTACGATTCCACAATCTTATGGAAACATGTCTAAACTTTTGAAGAT- : 1041
                                                                                                                                                               
                     *      1840         *      1860         *      1880         *      1900         *      1920         *      1940         *      1960       
Genomic   : TAAGTGATAGTTTCATTATGTATCTGAAATCACTGATGTGTGAGGCTGGAAGGGCTGTCATCTTCTATTAATGCATTCAACAATTCAATACGAAAGTCATGCATTGGATTTGAATTAACCTGTGGGAATAGTAATCAGCC : 1960
NM_100561 : -------------------------------------------------------------------------------------------------------------------------------------------- :    -
FJ708626  : -------------------------------------------------------------------------------------------------------------------------------------------- :    -
                                                                                                                                                               
                     *      1980         *      2000         *      2020         *      2040         *      2060         *      2080         *      2100       
Genomic   : ATATTTGAGCTAATTAGGATAGTGCATTCTAAATGTAGAGATACTGGTTGTCGTTTTCTATTTTTTCCTGATCTTTTTGGGCATAAGATACATGTGTATGCTATGCTAACTTATGCATATTTGTCGTATGCCCACGGGCA : 2100
NM_100561 : -------------------------------------------------------------------------------------------------------------------------------------------- :    -
FJ708626  : -------------------------------------------------------------------------------------------------------------------------------------------- :    -
                                                                                                                                                               
                     *      2120         *      2140         *      2160         *      2180         *      2200         *      2220         *      2240       
Genomic   : GGAGTCTTAGGAACTGCAGCTTGCAAGGGCCAGTGCCTGATCTTAGCAGCATACCGAACCTTGGTTATTTGTAAGTCTTATAACTTATCTAGACGCTTCCTTATATAGTCGTGCAAGCCTTAGCGAAGGCGAATTAACCT : 2240
NM_100561 : -GAGTCTTAGGAACTGCAGCTTGCAAGGGCCAGTGCCTGATCTTAGCAGCATACCGAACCTTGGTTATTT---------------------------------------------------------------------- :  797
FJ708626  : -GAGTCTTAGGAACTGCAGCTTGCAAGGGCCAGTGCCTGATCTTAGCAGCATACCGAACCTTGGTTATTT---------------------------------------------------------------------- : 1110
                                                                                                                                                               
                     *      2260         *      2280         *      2300         *      2320         *      2340         *      2360         *      2380       
Genomic   : CATCTATTTCTGGTTGTCGGACATGAATTCCAGGGACCTAAGTCAAAATCAGTTAAATGGATCTATACCTGCAGGGAAGCTTTCTGATAGTATCACAACCATGTAAACTTTTAGCGCTTTTCATAATTCTGGCCTTTGAT : 2380
NM_100561 : ---------------------------------GGACCTAAGTCAAAATCAGTTAAATGGATCTATACCTGCAGGGAAGCTTTCTGATAGTATCACAACCAT-------------------------------------- :  866
FJ708626  : ---------------------------------GGACCTAAGTCAAAATCAGTTAAATGGATCTATACCTGCAGGGAAGCTTTCTGATAGTATCACAACCAT-------------------------------------- : 1179
        
                                                                                                                                                       
                     *      2400         *      2420         *      2440         *      2460         *      2480         *      2500         *      2520       
Genomic   : TTAATCCGGGCAATGTACTGGGATTAGTATTCTCTTTTTGGGGTTGGAAATGAAGTGCTTTTAACTTTGATATCTTCTGATGAAAATACTGCAGCGATCTATCCAATAACAGTCTAACTGGAACCATTCCAACAAATTTC : 2520
NM_100561 : ----------------------------------------------------------------------------------------------CGATCTATCCAATAACAGTCTAACTGGAACCATTCCAACAAATTTC :  912
FJ708626  : ----------------------------------------------------------------------------------------------CGATCTATCCAATAACAGTCTAACTGGAACCATTCCAACAAATTTC : 1225
                                                                                                                                                               
                     *      2540         *      2560         *      2580         *      2600         *      2620         *      2640         *      2660       
Genomic   : TCAGGCCTTCCACGGCTTCAAAAGCTGTAAGTCTGTTTGATTGGTGTAGCGAGTTTTGTTTGGTTTCTGAAATGCTGTTGTTCGTTCTATCTTGGATATGAAGATGGCTGATTGTTTGTTTTGTTGTCTACCTAATGTGA : 2660
NM_100561 : TCAGGCCTTCCACGGCTTCAAAAGCT------------------------------------------------------------------------------------------------------------------ :  938
FJ708626  : TCAGGCCTTCCACGGCTTCAAAAGCT------------------------------------------------------------------------------------------------------------------ : 1251
                                                                                                                                                               
                     *      2680         *      2700         *      2720         *      2740         *      2760         *      2780         *      2800       
Genomic   : CAGGTCGCTTGCAAACAATGCTCTGAGTGGTTCCATTCCCTCTAGAATATGGCAGGAAAGAGAGCTGAATTCAACCGAGAGTATTATCGTGTATGACTGTTACAAAAACAATCATATCTCCTATCCTGTTACCTAGAAGC : 2800
NM_100561 : ---GTCGCTTGCAAACAATGCTCTGAGTGGTTCCATTCCCTCTAGAATATGGCAGGAAAGAGAGCTGAATTCAACCGAGAGTATTATCGT-------------------------------------------------- : 1025
FJ708626  : ---GTCGCTTGCAAACAATGCTCTGAGTGGTTCCATTCCCTCTAGAATATGGCAGGAAAGAGAGCTGAATTCAACCGAGAGTATTATCGT-------------------------------------------------- : 1338
                                                                                                                                                               
                     *      2820         *      2840         *      2860         *      2880         *      2900         *      2920         *      2940       
Genomic   : ATGTTCCAACTTTCTTTATATTGTCTCTTGACAAACGTCAGTTCCTTTATGTTTTCAGGGATCTGCGGAACAATGGGTTTTCAAATATCTCTGGCAGATCCGATCTCCGTCCAAATGTGACCGTCTGGTTTGCTTCTGCT : 2940
NM_100561 : ----------------------------------------------------------GGATCTGCGGAACAATGGGTTTTCAAATATCTCTGGCAGATCCGATCTCCGTCCAAATGTGACCGTCTG------------- : 1094
FJ708626  : ----------------------------------------------------------GGATCTGCGGAACAATGGGTTTTCAAATATCTCTGGCAGATCCGATCTCCGTCCAAATGTGACCGTCTG------------- : 1407
                                                                                                                                                               
                     *      2960         *      2980         *      3000         *      3020         *      3040         *      3060         *      3080       
Genomic   : CTTTCCTATTTCTATTTATTATCTGCCCATACGTACTTATTTTTGCTTTGCATATCATTCTAGCTACTCAATGATGTATATAACTGCTTTTTGGATCTTGTTTGCTCATTCTGAAATGTATTAAACTCTTTTCCTCAGAT : 3080
NM_100561 : -------------------------------------------------------------------------------------------------------------------------------------------- :    -
FJ708626  : -------------------------------------------------------------------------------------------------------------------------------------------- :    -
                                                                                                                                                               
                     *      3100         *      3120         *      3140         *      3160         *      3180         *      3200         *      3220       
Genomic   : TAGTGTCCACTTTTCGGGTATCATTTAACAACGTTTGTTTTTTCAGGCTTCAGGGGAATCCGTTGTGCTCAGATGGAAATCTGCTTCGATTGTGTGGACCTATAACTGAGGAAGACATTAATCAGGGTTCAACCAATTCT : 3220
NM_100561 : ----------------------------------------------GCTTCAGGGGAATCCGTTGTGCTCAGATGGAAATCTGCTTCGATTGTGTGGACCTATAACTGAGGAAGACATTAATCAGGGTTCAACCAATTCT : 1188
FJ708626  : ----------------------------------------------GCTTCAGGGGAATCCGTTGTGCTCAGATGGAAATCTGCTTCGATTGTGTGGACCTATAACTGAGGAAGACATTAATCAGGGTTCAACCAATTCT : 1501
                                                                                                                                                               
                     *      3240         *      3260         *      3280         *      3300         *      3320         *      3340         *      3360       
Genomic   : AATACTACAATTTGTTCTGACTGCCCACCCCCTTATGAATTTTCACCAGAACCTCTTAGACGTTGCTTTTGTGCTGCTCCTCTGCTTGTTGGATATCGGTTGAAAAGTCCTGGTTTCTCGGACTTTGTTCCTTACAGATC : 3360
NM_100561 : AATACTACAATTTGTTCTGACTGCCCACCCCCTTATGAATTTTCACCAGAACCTCTTAGACGTTGCTTTTGTGCTGCTCCTCTGCTTGTTGGATATCGGTTGAAAAGTCCTGGTTTCTCGGACTTTGTTCCTTACAGATC : 1328
FJ708626  : AATACTACAATTTGTTCTGACTGCCCACCCCCTTATGAATTTTCACCAGAACCTCTTAGACGTTGCTTTTGTGCTGCTCCTCTGCTTGTTGGATATCGGTTGAAAAGTCCTGGTTTCTCGGACTTTGTTCCTTACAGATC : 1641
                                                                                                                                                               
                     *      3380         *      3400         *      3420         *      3440         *      3460         *      3480         *      3500       
Genomic   : CGAATTTGAGCAATATATCACCTCTGGTCTTAGTTTGAATCTGTATCAGCTACGCCTTGACTCATTCCAGTGGCAGAAAGGACCTAGACTTCGAATGTATTTGAAGTTCTTTCCTGTTTTTGGTTCAAATGCCAACAATT : 3500
NM_100561 : CGAATTTGAGCAATATATCACCTCTGGTCTTAGTTTGAATCTGTATCAGCTACGCCTTGACTCATTCCAGTGGCAGAAAGGACCTAGACTTCGAATGTATTTGAAGTTCTTTCCTGTTTTTGGTTCAAATGCCAACAATT : 1468
FJ708626  : CGAATTTGAGCAATATATCACCTCTGGTCTTAGTTTGAATCTGTATCAGCTACGCCTTGACTCATTCCAGTGGCAGAAAGGACCTAGACTTCGAATGTATTTGAAGTTCTTTCCTGTTTTTGGTTCAAATGCCAACAATT : 1781
                                                                                                                                                               
                     *      3520         *      3540         *      3560         *      3580         *      3600         *      3620         *      3640       
Genomic   : CTTTCATATTCAATCGTAGCGAGGTTCGGCGAATAAGGGGCATGTTCACTGGATGGAATATCCGAGACGAAGATCTCTTTGGTCCTTATGAGCTTATGAATTTCACATTGTTAGATGTCTACAGAGATGGTTAGTGGCAG : 3640
NM_100561 : CTTTCATATTCAATCGTAGCGAGGTTCGGCGAATAAGGGGCATGTTCACTGGATGGAATATCCGAGACGAAGATCTCTTTGGTCCTTATGAGCTTATGAATTTCACATTGTTAGATGTCTACAGAGATG----------- : 1597
FJ708626  : CTTTCATATTCAATCGTAGCGAGGTTCGGCGAATAAGGGGCATGTTCACTGGATGGAATATCCGAGACGAAGATCTCTTTGGTCCTTATGAGCTTATGAATTTCACATTGTTAGATGTCTACAGAGATG----------- : 1910
                                                                                                                                                               
                     *      3660         *      3680         *      3700         *      3720         *      3740         *      3760         *      3780       
Genomic   : AATATTCGTCCTCTCATATTTATTCAAAACAGAACGTGGTTAAAATACAAGTAAACGAGCTGATGTCTTTCTGCTACATTAAACATAAAAGTGTCATTTGTTTATCAGCGTCCCTGATAACAATAGATCCTGTCTTACCT : 3780
NM_100561 : -------------------------------------------------------------------------------------------------------------------------------------------- :    -
FJ708626  : -------------------------------------------------------------------------------------------------------------------------------------------- :    -
                                                                                                                                                               
                     *      3800         *      3820         *      3840         *      3860         *      3880         *      3900         *      3920       
Genomic   : TTTTGTTCTTACGAAGCTATAAAACTTTTGCATACATGGACATTGTTTTGACTGTCATGCTTTTTATGTTCAGTGTTTCCTTCAGCTTCGCCATCTGGTCTAAGTAATGGTGCAGTTGCGGGAATAGTTCTTGGTTCTGT : 3920
NM_100561 : -------------------------------------------------------------------------TGTTTCCTTCAGCTTCGCCATCTGGTCTAAGTAATGGTGCAGTTGCGGGAATAGTTCTTGGTTCTGT : 1664
FJ708626  : -------------------------------------------------------------------------TGTTTCCTTCAGCTTCGCCATCTGGTCTAAGTAATGGTGCAGTTGCGGGAATAGTTCTTGGTTCTGT : 1977
                                                                                                                                                               
                     *      3940         *      3960         *      3980         *      4000         *      4020         *      4040         *      4060       
Genomic   : TGCAGCTGCAGTAACGCTAACTGCTATCATTGCCCTTATCATTATGAGAAAACGTATGAGAGGATACAGTGCAGTTGCTAGAAGAAAGCGATGTAAGCATTTTCTTGTGGATGCCGATTTGTGCTCGAGTAAATGATTTA : 4060
NM_100561 : TGCAGCTGCAGTAACGCTAACTGCTATCATTGCCCTTATCATTATGAGAAAACGTATGAGAGGATACAGTGCAGTTGCTAGAAGAAAGCGAT------------------------------------------------ : 1756
FJ708626  : TGCAGCTGCAGTAACGCTAACTGCTATCATTGCCCTTATCATTATGAGAAAACGTATGAGAGGATACAGTGCAGTTGCTAGAAGAAAGCGAT------------------------------------------------ : 2069
                                                                                                                                                               
                     *      4080         *      4100         *      4120         *      4140         *      4160         *      4180         *      4200       
Genomic   : TCGGAATTGTACGTTTTTTTTGGTTTTTGGTGTTACAAGTTTGATTCATTCATGTGCAGCTTCCAAAGCTTCTTTGAAAATCGAAGGTGTGAAGAGCTTCACTTATGCTGAGTTGGCTCTGGCTACAGACAATTTTAATA : 4200
NM_100561 : -----------------------------------------------------------CTTCCAAAGCTTCTTTGAAAATCGAAGGTGTGAAGAGCTTCACTTATGCTGAGTTGGCTCTGGCTACAGACAATTTTAATA : 1837
FJ708626  : -----------------------------------------------------------CTTCCAAAGCTTCTTTGAAAATCGAAGGTGTGAAGAGCTTCACTTATGCTGAGTTGGCTCTGGCTACAGACAATTTTAATA : 2150
                                                                                                                                                               
                     *      4220         *      4240         *      4260         *      4280         *      4300         *      4320         *      4340       
Genomic   : GTTCCACTCAAATTGGGCAAGGGGGTTATGGAAAGGTATACAAAGGTACACTTGGGAGTGGAACGGTTGTGGCAATTAAAAGAGCACAAGAGGGATCATTGCAGGGTGAGAAGGAGTTCCTAACTGAAATTGAATTGTTA : 4340
NM_100561 : GTTCCACTCAAATTGGGCAAGGGGGTTATGGAAAGGTATACAAAGGTACACTTGGGAGTGGAACGGTTGTGGCAATTAAAAGAGCACAAGAGGGATCATTGCAGGGTGAGAAGGAGTTCCTAACTGAAATTGAATTGTTA : 1977
FJ708626  : GTTCCACTCAAATTGGGCAAGGGGGTTATGGAAAGGTATACAAAGGTACACTTGGGAGTGGAACGGTTGTGGCAATTAAAAGAGCACAAGAGGGATCATTGCAGGGTGAGAAGGAGTTCCTAACTGAAATTGAATTGTTA : 2290
                                                                                                                                                               
                     *      4360         *      4380         *      4400         *      4420         *      4440         *      4460         *      4480       
Genomic   : TCGAGATTGCATCACAGAAACCTTGTTTCGTTGCTTGGATTCTGTGATGAAGAAGGCGAACAGGTACTTTGTCCAAATTCCTCTTCTGGTTGATGTCCAAAACGTTTATATGTATCTCTTATTGCTTGTATTGTTAAGCG : 4480
NM_100561 : TCGAGATTGCATCACAGAAACCTTGTTTCGTTGCTTGGATTCTGTGATGAAGAAGGCGAACAG----------------------------------------------------------------------------- : 2040
FJ708626  : TCGAGATTGCATCACAGAAACCTTGTTTCGTTGCTTGGATTCTGTGATGAAGAAGGCGAACAG---------------------------------------------------------------------------CG : 2355
                                                                                                                                                               
                     *      4500         *      4520         *      4540         *      4560         *      4580         *      4600         *      4620       
Genomic   : TGCAGATGCTGGTTTATGAGTACATGGAAAATGGTACTTTGCGAGACAACATTTCTGGTATGTTTTACTTCTTCACTGAGTCATCTATAGTGGTGCTTGTTCTCTATGTGAATCAGCAAAGACTACCCACCGATGTTCAC : 4620
NM_100561 : -----ATGCTGGTTTATGAGTACATGGAAAATGGTACTTTGCGAGACAACATTTCTG----------------------------------------------------------------------------------- : 2092
FJ708626  : TGCAGATGCTGGTTTATGAGTACATGGAAAATGGTACTTTGCGAGACAACATTTCTG----------------------------------------------------------------------------------- : 2412
                                                                                                                                                               
                     *      4640         *      4660         *      4680         *      4700         *      4720         *      4740         *      4760       
Genomic   : TGTTCTTAATGTGAATTCTTTGGAGATGACTTATAAGCAGCATAGATGATATTGTGTGCTTTTAAGTTCGGCTCTATTCCTCGTGAATAACAATTTACATACAAATATGTGTTTTGGCAGTTAAATTAAAAGAGCCTCTA : 4760
NM_100561 : ------------------------------------------------------------------------------------------------------------------------TTAAATTAAAAGAGCCTCTA : 2112
FJ708626  : ------------------------------------------------------------------------------------------------------------------------TTAAATTAAAAGAGCCTCTA : 2432
                                                                                                                                                               
    

                                                                                                                                                           
                     *      4780         *      4800         *      4820         *      4840         *      4860         *      4880         *      4900       
Genomic   : GACTTTGCGATGAGACTACGGATTGCTTTAGGTTCAGCCAAGGGAATCTTGTATTTACACACGGAAGCTAATCCCCCGATATTTCATCGCGATATCAAAGCAAGCAACATATTGTTGGACTCCAGATTCACCGCAAAGGT : 4900
NM_100561 : GACTTTGCGATGAGACTACGGATTGCTTTAGGTTCAGCCAAGGGAATCTTGTATTTACACACGGAAGCTAATCCCCCGATATTTCATCGCGATATCAAAGCAAGCAACATATTGTTGGACTCCAGATTCACCGCAAAGGT : 2252
FJ708626  : GACTTTGCGATGAGACTACGGATTGCTTTAGGTTCAGCCAAGGGAATCTTGTATTTACACACGGAAGCTAATCCCCCGATATTTCATCGCGATATCAAAGCAAGCAACATATTGTTGGACTCCAGATTCACCGCAAAGGT : 2572
                                                                                                                                                               
                     *      4920         *      4940         *      4960         *      4980         *      5000         *      5020         *      5040       
Genomic   : TGCAGATTTTGGACTCTCAAGACTTGCCCCAGTACCTGATATGGAAGGCATCTCACCTCAGCACGTGTCTACTGTTGTAAAAGGGACTCCTGTAAGATCTCATTCCTGCATTGCTTCTCTAAAGTTTTTTTCTCAAACTC : 5040
NM_100561 : TGCAGATTTTGGACTCTCAAGACTTGCCCCAGTACCTGATATGGAAGGCATCTCACCTCAGCACGTGTCTACTGTTGTAAAAGGGACTCCT------------------------------------------------- : 2343
FJ708626  : TGCAGATTTTGGACTCTCAAGACTTGCCCCAGTACCTGATATGGAAGGCATCTCACCTCAGCACGTGTCTACTGTTGTAAAAGGGACTCCT------------------------------------------------- : 2663
                                                                                                                                                               
                     *      5060         *      5080         *      5100         *      5120         *      5140         *      5160         *      5180       
Genomic   : ATGACTGAGAGTTATTTGTCGACTTGCAGGGTTACCTTGACCCGGAATATTTCTTGACTCATCAATTGACGGACAAAAGTGATGTATATAGTCTAGGCGTAGTGTTGTTAGAGCTCTTTACTGGAATGCAGCCAATCACA : 5180
NM_100561 : -----------------------------GGTTACCTTGACCCGGAATATTTCTTGACTCATCAATTGACGGACAAAAGTGATGTATATAGTCTAGGCGTAGTGTTGTTAGAGCTCTTTACTGGAATGCAGCCAATCACA : 2454
FJ708626  : -----------------------------GGTTACCTTGACCCGGAATATTTCTTGACTCATCAATTGACGGACAAAAGTGATGTATATAGTCTAGGCGTAGTGTTGTTAGAGCTCTTTACTGGAATGCAGCCAATCACA : 2774
                                                                                                                                                               
                     *      5200         *      5220         *      5240         *      5260         *      5280         *      5300         *      5320       
Genomic   : CATGGCAAGAACATTGTGCGAGAGGTAATTCTGAGTAGATAATAAGAGCGTGTGTTTTCCTTTTAGGCGTTATATAAATTGTGTGGAATGTTTGTTTCTGTCAGATCAACATTGCCTACGAGTCTGGTTCGATATTATCA : 5320
NM_100561 : CATGGCAAGAACATTGTGCGAGAG--------------------------------------------------------------------------------ATCAACATTGCCTACGAGTCTGGTTCGATATTATCA : 2514
FJ708626  : CATGGCAAGAACATTGTGCGAGAG--------------------------------------------------------------------------------ATCAACATTGCCTACGAGTCTGGTTCGATATTATCA : 2834
                                                                                                                                                               
                     *      5340         *      5360         *      5380         *      5400         *      5420         *      5440         *      5460       
Genomic   : ACCGTGGATAAGAGAATGAGCTCAGTTCCAGACGAATGCCTCGAAAAGTTTGCAACTTTAGCGCTGCGATGCTGCAGAGAGGAGACAGATGCGAGGCCTTCAATGGCAGAAGTTGTAAGAGAACTAGAAATCATATGGGA : 5460
NM_100561 : ACCGTGGATAAGAGAATGAGCTCAGTTCCAGACGAATGCCTCGAAAAGTTTGCAACTTTAGCGCTGCGATGCTGCAGAGAGGAGACAGATGCGAGGCCTTCAATGGCAGAAGTTGTAAGAGAACTAGAAATCATATGGGA : 2654
FJ708626  : ACCGTGGATAAGAGAATGAGCTCAGTTCCAGACGAATGCCTCGAAAAGTTTGCAACTTTAGCGCTGCGATGCTGCAGAGAGGAGACAGATGCGAGGCCTTCAATGGCAGAAGTTGTAAGAGAACTAGAAATCATATGGGA : 2974
                                                                                                                                                               
                     *      5480         *      5500         *      5520         *      5540         *      5560         *      5580         *      5600       
Genomic   : ACTGATGCCGGAATCTCATGTAGCCAAGACAGCGGATCTCTCTGAGACAATGACTCATCCATCATCGTCATCGAATTCTTCAATCATGAAGCATCATTATACATCGATGGATGTCTCCGGCTCTGACCTCGTCAGTGGAG : 5600
NM_100561 : ACTGATGCCGGAATCTCATGTAGCCAAGACAGCGGATCTCTCTGAGACAATGACTCATCCATCATCGTCATCGAATTCTTCAATCATGAAGCATCATTATACATCGATGGATGTCTCCGGCTCTGACCTCGTCAGTGGAG : 2794
FJ708626  : ACTGATGCCGGAATCTCATGTAGCCAAGACAGCGGATCTCTCTGAGACAATGACTCATCCATCATCGTCATCGAATTCTTCAATCATGAAGCATCATTATACATCGATGGATGTCTCCGGCTCTGACCTCGTCAGTGGAG : 3114
                                                                                                                                                               
                     *      5620          
Genomic   : TTGCTCCCTCAGTTGCACCTAGA : 5623
NM_100561 : TTGCTCCCTCAGTTGCACCTAGA : 2817
FJ708626  : TTGCTCCCTCAGTTGCACCTAGA : 3137


At1g29730
                                                                                                                                                               
                     *        20         *        40         *        60         *        80         *       100         *       120         *       140       
Genomic   : ATGTCTGCAGCTTACAATCTCATGATTAAGTCGAAAAGTTGCCTTTTTAGCTCTTCTATTCTCTTTGTTGTTACACTTATCTACTTATTATGTACTGTATCTGCTTCTCCTTCTCTGCACCCAGATGAAGGTTTAGTAGT :  140
NM_102713 : ATGTCTGCAGCTTACAATCTCATGATTAAGTCGAAAAGTTGCCTTTTTAGCTCTTCTATTCTCTTTGTTGTTACACTTATCTACTTATTATGTACTGTATCTGCTTCTCCTTCTCTGCACCCAGATGAAG---------- :  130
FJ708641  : ATGTCTGCAGCTTACAATCTCATGATTAAGTCGAAAAGTTGCCTTTTTAGCTCTTCTATTCTCTTTGTTGTTACACTTATCTACTTATTATGTACTGTATCTGCTTCTCCTTCTCTGCACCCAGATGAAGGTTTAGTAGT :  140
                                                                                                                                                               
                     *       160         *       180         *       200         *       220         *       240         *       260         *       280       
Genomic   : TTCAATGCATTATATCATATATACATGTGCCTCTCTTTGTGAATTTGAACTAAACTTGATTTTGTTTCTGGTTTGCTGTCTTCGCCAGTGGAAGCGCTGAAGGATATCACTGAAACATTGGGTGTGAAGCACTTGAACCT :  280
NM_102713 : ----------------------------------------------------------------------------------------TGGAAGCGCTGAAGGATATCACTGAAACATTGGGTGTGAAGCACTTGAACCT :  182
FJ708641  : TTCAATGCATTATATCATATATACATGTGCCTCTCTTTGTGAATTTGAACTAAACTTGATTTTGTTTCTGGTTTGCTGTCTTCGCCAGTGGAAGCGCTGAAGGATATCACTGAAACATTGGGTGTGAAGCACTTGAACCT :  280
                                                                                                                                                               
                     *       300         *       320         *       340         *       360         *       380         *       400         *       420       
Genomic   : AAGTGAAGATCCATGTCTCACAAAGACTCTAGTGATATCTCAAGGTGTTCTCAAGGAAGGACAGAACAGCACAATCAGATGTGACTGTCATTTCAATAACTACAGCACTTGTCATATCAAACACTTGTAAGAAAAACCAT :  420
NM_102713 : AAGTGAAGATCCATGTCTCACAAAGACTCTAGTGATATCTCAAGGTGTTCTCAAGGAAGGACAGAACAGCACAATCAGATGTGACTGTCATTTCAATAACTACAGCACTTGTCATATCAAACACTT-------------- :  308
FJ708641  : AAGTGAAGATCCATGTCTCACAAAGACTCTAGTGATATCTCAAGGTGTTCTCAAGGAAGGACAGAACAGCACAATCAGATGTGACTGTCATTTCAATAACTACAGCACTTGTCATATCAAACACTT-------------- :  406
                                                                                                                                                               
                     *       440         *       460         *       480         *       500         *       520         *       540         *       560       
Genomic   : TCTGAAAAAGAAGAAACTAACCGTTTTTCTGCTTATTTCTCTTGTTTCTGACCAATTACTATGTACCTCATATCTAGTGTCCTCCAGAAATTCAATCTTCCAGGTAGACTTCCTCCAATGCTGTACAAGTTTCGGCATCT :  560
NM_102713 : -----------------------------------------------------------------------------TGTCCTCCAGAAATTCAATCTTCCAGGTAGACTTCCTCCAATGCTGTACAAGTTTCGGCATCT :  371
FJ708641  : -----------------------------------------------------------------------------TGTCCTCCAGAAATTCAATCTTCCAGGTAGACTTCCTCCAATGCTGTACAAGTTTCGGCATCT :  469
                                                                                                                                                               
                     *       580         *       600         *       620         *       640         *       660         *       680         *       700       
Genomic   : TGAATCGATGTGAGGAGGGTTTTCAGTGTTTCAACTACTTTCAATATCTACTGCTATCGTAGAAGAAACATAACAATGGCTGGAATTCTTTTGCTTGTTTTTGTAGTGACTTATACAATAATTACCTTTATGGCTCAATT :  700
NM_102713 : TGAATCGAT-------------------------------------------------------------------------------------------------TGACTTATACAATAATTACCTTTATGGCTCAATT :  414
FJ708641  : TGAATCGAT-------------------------------------------------------------------------------------------------TGACTTATACAATAATTACCTTTATGGCTCAATT :  512
                                                                                                                                                               
                     *       720         *       740         *       760         *       780         *       800         *       820         *       840       
Genomic   : CCTATGGAATGGGCCTCACTGCCTTACCTCAAATCTATGTAAGTCTTTGTTCTCAAGTTTGTTTATTGCTAGGAAAGTAAGAAAAGGTTCTGAAGTTACCTGATATATTTCTCCGCAGCTCTGTCTGTGCAAACCGCTTA :  840
NM_102713 : CCTATGGAATGGGCCTCACTGCCTTACCTCAAATCTAT--------------------------------------------------------------------------------CTCTGTCTGTGCAAACCGCTTA :  474
FJ708641  : CCTATGGAATGGGCCTCACTGCCTTACCTCAAATCTAT--------------------------------------------------------------------------------CTCTGTCTGTGCAAACCGCTTA :  572
                                                                                                                                                               
                     *       860         *       880         *       900         *       920         *       940         *       960         *       980       
Genomic   : TCAGGAGACATTCCCAAAGGATTGGGGAAGTTTATTAACCTCACCCTTTTGTATGTTCTTAACATTATTTTGTTGATCTTATGAGATTACATATAAAGCTTTTTGTCCTTTTTGAAAACTAAATCTTTCGTCTTTTTGTT :  980
NM_102713 : TCAGGAGACATTCCCAAAGGATTGGGGAAGTTTATTAACCTCACCCTTTT------------------------------------------------------------------------------------------ :  524
FJ708641  : TCAGGAGACATTCCCAAAGGATTGGGGAAGTTTATTAACCTCACCCTTTT------------------------------------------------------------------------------------------ :  622
                                                                                                                                                               
                     *      1000         *      1020         *      1040         *      1060         *      1080         *      1100         *      1120       
Genomic   : TGATTGCAGAGTTCTTGAAGCCAATCAGTTTTCGGGAACTATTCCTAAGGAACTAGGGAACCTAGTGAATCTACAAGGACTGTACGTAAGACCCTTTTAAAAATCACTTTTGAAGAGATCGAAACAAATTTATGACCCAG : 1120
NM_102713 : ---------AGTTCTTGAAGCCAATCAGTTTTCGGGAACTATTCCTAAGGAACTAGGGAACCTAGTGAATCTACAAGGACT----------------------------------------------------------- :  596
FJ708641  : ---------AGTTCTTGAAGCCAATCAGTTTTCGGGAACTATTCCTAAGGAACTAGGGAACCTAGTGAATCTACAAGGACT----------------------------------------------------------- :  694
                                                                                                                                                               
                     *      1140         *      1160         *      1180         *      1200         *      1220         *      1240         *      1260       
Genomic   : TGATTTTCTCAAACATGATCCATTTAATTTTGTTTTGCTGATTCCAGAGGACTCTCTTCCAACCAACTTGTCGGAGGCCTCCCCAAGACATTAGCAAAACTAACAAAGCTAACTAATCTGTAAGATCATCTTTCTCATCT : 1260
NM_102713 : -----------------------------------------------AGGACTCTCTTCCAACCAACTTGTCGGAGGCCTCCCCAAGACATTAGCAAAACTAACAAAGCTAACTAATCT--------------------- :  668
FJ708641  : -----------------------------------------------AGGACTCTCTTCCAACCAACTTGTCGGAGGCCTCCCCAAGACATTAGCAAAACTAACAAAGCTAACTAATCT--------------------- :  766
                                                                                                                                                               
                     *      1280         *      1300         *      1320         *      1340         *      1360         *      1380         *      1400       
Genomic   : GGATTACTAACAAAATATCAGCTTTAGAATGTGTTCATCATGCTGAATTGTTTTTCATTATATTGTTTACAGCCATTTAAGTGATAATCGCCTGAACGGATCGATTCCAGAGTTTATTGGGAAGTTACCAAAACTTCAAA : 1400
NM_102713 : ------------------------------------------------------------------------CCATTTAAGTGATAATCGCCTGAACGGATCGATTCCAGAGTTTATTGGGAAGTTACCAAAACTTCAAA :  736
FJ708641  : ------------------------------------------------------------------------CCATTTAAGTGATAATCGCCTGAACGGATCGATTCCAGAGTTTATTGGGAAGTTACCAAAACTTCAAA :  834
                                                                                                                                                               
                     *      1420         *      1440         *      1460         *      1480         *      1500         *      1520         *      1540       
Genomic   : GATTGTGAGTAAAGTATCTTTATTTTTCATTACACTTAAATAGTTAGTATTTGTACATTTCAATGTTTTCTGACTTGTGCTGATAACGTTGTATCAGAGAACTCTATGCGAGTGGCCTTAGAGGACCTATTCCAGACTCC : 1540
NM_102713 : GATT---------------------------------------------------------------------------------------------AGAACTCTATGCGAGTGGCCTTAGAGGACCTATTCCAGACTCC :  783
FJ708641  : GATT---------------------------------------------------------------------------------------------AGAACTCTATGCGAGTGGCCTTAGAGGACCTATTCCAGACTCC :  881
                                                                                                                                                               
                     *      1560         *      1580         *      1600         *      1620         *      1640         *      1660         *      1680       
Genomic   : ATTTTTCATCTGGAGAATTTGATCGACGTGTAAGTTTTTTATCCTCTTTAGATATTTGTTGAAGCAATAATTTTGTAAGTTGCAGAAGCCTATGTTCTTTTTTGAAACGAAATTGTGGCCTTTTCTTTCTATGCAGGAGA : 1680
NM_102713 : ATTTTTCATCTGGAGAATTTGATCGACGTG-----------------------------------------------------------------------------------------------------------AGA :  816
FJ708641  : ATTTTTCATCTGGAGAATTTGATCGACGTG-----------------------------------------------------------------------------------------------------------AGA :  914
                                                                                                                                                               
                     *      1700         *      1720         *      1740         *      1760         *      1780         *      1800         *      1820       
Genomic   : ATCAGTGACACGGTTGCAGGATTGGGACATGTTCCTCAGATAACTAGCACGAGCCTGAAATATTTGTATGTAATAGTACATTTTTATGTCTCAGGTCCAGATACGATCATATGGTCAATTACTTGTGTTTAGACTTTAGA : 1820
NM_102713 : ATCAGTGACACGGTTGCAGGATTGGGACATGTTCCTCAGATAACTAGCACGAGCCTGAAATATTTG-------------------------------------------------------------------------- :  882
FJ708641  : ATCAGTGACACGGTTGCAGGATTGGGACATGTTCCTCAGATAACTAGCACGAGCCTGAAATATTTG-------------------------------------------------------------------------- :  980
                                                                                                                                                               
                     *      1840         *      1860         *      1880         *      1900         *      1920         *      1940         *      1960       
Genomic   : CTGATGTTGATCATTTTCAGGGTTTTGAGAAACATAAACTTATCTGGACCAATTCCAACCAGTATCTGGGATCTGCCCAGTTTAATGACTCTGTAAGTCTCATGTTTTATCACCACGCATCTTGCTAAACACTGTCCAGT : 1960
NM_102713 : ---------------------GTTTTGAGAAACATAAACTTATCTGGACCAATTCCAACCAGTATCTGGGATCTGCCCAGTTTAATGACTCTG----------------------------------------------- :  954
FJ708641  : ---------------------GTTTTGAGAAACATAAACTTATCTGGACCAATTCCAACCAGTATCTGGGATCTGCCCAGTTTAATGACTCTG----------------------------------------------- : 1052
                                                                                                                                                               
                     *      1980         *      2000         *      2020         *      2040         *      2060         *      2080         *      2100       
Genomic   : CTCTTATAAGTTATAACGCCTTTTTTTTTGACAGGGATCTTTCCTTTAATAGGCTGACTGGAGAAATACCAGCATATGCAACTGCCCCAAAATACACGTAAGTCTTAAAGTGTCAGATGATCAAAAGGATTAGGTATGCT : 2100
NM_102713 : -----------------------------------GATCTTTCCTTTAATAGGCTGACTGGAGAAATACCAGCATATGCAACTGCCCCAAAATACAC------------------------------------------- : 1016
FJ708641  : -----------------------------------GATCTTTCCTTTAATAGGCTGACTGGAGAAATACCAGCATATGCAACTGCCCCAAAATACAC------------------------------------------- : 1114
                                                                                                                                                               
                     *      2120         *      2140         *      2160         *      2180         *      2200         *      2220         *      2240       
Genomic   : ATTAAAATTTACTATAGCTGTTGAACTCATCTCCTGGTTTCATTTTTCAGATATTTAGCTGGAAACATGTTGTCTGGAAAGGTTGAAACAGGAGCTTTCCTTACTGCAAGCACAAATATGTATGACCATTCTTATTTTAT : 2240
NM_102713 : --------------------------------------------------ATATTTAGCTGGAAACATGTTGTCTGGAAAGGTTGAAACAGGAGCTTTCCTTACTGCAAGCACAAATAT--------------------- : 1085
FJ708641  : --------------------------------------------------ATATTTAGCTGGAAACATGTTGTCTGGAAAGGTTGAAACAGGAGCTTTCCTTACTGCAAGCACAAATAT--------------------- : 1183                                                                                                                                                               
                                                                                                                                                               
                     *      2260         *      2280         *      2300         *      2320         *      2340         *      2360         *      2380       
Genomic   : AGCAGTCATATATGTCAAGTCATTTCTCGGTATTACACTAATCTCTCCTTTTACCAATATTCATATGTGCAGTGATCTTTCCTATAATAATTTCACGTGGTCTCCAATGTGCAAAGAGAGGAAGTATGTTTTTTCTGTTG : 2380
NM_102713 : ------------------------------------------------------------------------TGATCTTTCCTATAATAATTTCACGTGGTCTCCAATGTGCAAAGAGAGGAAG---------------- : 1137
FJ708641  : ------------------------------------------------------------------------TGATCTTTCCTATAATAATTTCACGTGGTCTCCAATGTGCAAAGAGAGGAAG---------------- : 1235
               
                                                                                                                                                               
                     *      2400         *      2420         *      2440         *      2460         *      2480         *      2500         *      2520       
Genomic   : TTAACTACCGCCTTTCATCTGCTTAAATTTTCTGGATACTTTTAACATAAGTTTCCATGCAGGAATATAAACACATATGAGAGCTCACACTCAAAAAACCGTCTGTAAGAGCATTTTGTCAATCTTATTAATCACTTGCT : 2520
NM_102713 : ---------------------------------------------------------------AATATAAACACATATGAGAGCTCACACTCAAAAAACCGTCT------------------------------------ : 1178
FJ708641  : ---------------------------------------------------------------AATATAAACACATATGAGAGCTCACACTCAAAAAACCGTCT------------------------------------ : 1276
                                                                                                                                                               
                     *      2540         *      2560         *      2580         *      2600         *      2620         *      2640         *      2660       
Genomic   : ACTTCGTGTACTGTTATTAACTCATTGTTCTGTATACAGAACCAGACTTCTTCCATGTTCCGCCATAAAGCAATGCCAAAATTGTAGGTTCTTTTGCTTTTTATTGATTTCCAAATACCCTTTTTCGAGTTTCTGCATAT : 2660
NM_102713 : ---------------------------------------AACCAGACTTCTTCCATGTTCCGCCATAAAGCAATGCCAAAATT--------------------------------------------------------- : 1222
FJ708641  : ---------------------------------------AACCAGACTTCTTCCATGTTCCGCCATAAAGCAATGCCAAAATT--------------------------------------------------------- : 1320
                                                                                                                                                               
                     *      2680         *      2700         *      2720         *      2740         *      2760         *      2780         *      2800       
Genomic   : GAACTGGAAATTGTTTACTTTCCACAGATAGTAGATCGCTGCATATAAACTGTGGGGGACCTGATGTAACCATCGAAAACTCTCGAGGAAGGTTTCTGTATGAAGGTGATAACTATGGACTTACCGGATCAGCTACGAAC : 2800
NM_102713 : ---------------------------ATAGTAGATCGCTGCATATAAACTGTGGGGGACCTGATGTAACCATCGAAAACTCTCGAGGAAGGTTTCTGTATGAAGGTGATAACTATGGACTTACCGGATCAGCTACGAAC : 1335
FJ708641  : ---------------------------ATAGTAGATCGCTGCATATAAACTGTGGGGGACCTGATGTAACCATCGAAAACTCTCGAGGAAGGTTTCTGTATGAAGGTGATAACTATGGACTTACCGGATCAGCTACGAAC : 1433
                                                                                                                                                               
                     *      2820         *      2840         *      2860         *      2880         *      2900         *      2920         *      2940       
Genomic   : TATTATAGGAAAAATTGGGGATACAGCAATACTGGTGACTTTATGGATGATGCAATAACAGAAGATACATACACAGTTTCATCAGAATCTGCAGTTTCAGCAAAATATCCAGACCTTTACCAGAATGCTCGGCGCTCTCC : 2940
NM_102713 : TATTATAGGAAAAATTGGGGATACAGCAATACTGGTGACTTTATGGATGATGCAATAACAGAAGATACATACACAGTTTCATCAGAATCTGCAGTTTCAGCAAAATATCCAGACCTTTACCAGAATGCTCGGCGCTCTCC : 1475
FJ708641  : TATTATAGGAAAAATTGGGGATACAGCAATACTGGTGACTTTATGGATGATGCAATAACAGAAGATACATACACAGTTTCATCAGAATCTGCAGTTTCAGCAAAATATCCAGACCTTTACCAGAATGCTCGGCGCTCTCC : 1573
                                                                                                                                                               
                     *      2960         *      2980         *      3000         *      3020         *      3040         *      3060         *      3080       
Genomic   : CCTCAGTTTGGCTTACTATGCATTTTGCTTCGAAAATGGAAGCTACAATGTGAAACTCCATTTTGCAGAGATTCAGTTCTCAGATGTGGAACCATACACTAAACTAGCGAAACGGGTTTTTAACATTTACATTCAGGTAC : 3080
NM_102713 : CCTCAGTTTGGCTTACTATGCATTTTGCTTCGAAAATGGAAGCTACAATGTGAAACTCCATTTTGCAGAGATTCAGTTCTCAGATGTGGAACCATACACTAAACTAGCGAAACGGGTTTTTAACATTTACATTCAGG--- : 1612
FJ708641  : CCTCAGTTTGGCTTACTATGCATTTTGCTTCGAAAATGGAAGCTACAATGTGAAACTCCATTTTGCAGAGATTCAGTTCTCAGATGTGGAACCATACACTAAACTAGCGAAACGGGTTTTTAACATTTACATTCAGG--- : 1710
                                                                                                                                                               
                     *      3100         *      3120         *      3140         *      3160         *      3180         *      3200         *      3220       
Genomic   : ATATAGCCTTTTGCCGCAGATTCTTTAAGAACCCCTTTTCAGGGGTTTTAGCCGATTCTTGAAGTTTGAAATTTAATATATGTTCAGGGGAAGTTGATTTGGGAGGATTTCAGCATTAGAGAGGAGGCTAATGGAACTCA : 3220
NM_102713 : ----------------------------------------------------------------------------------------GGAAGTTGATTTGGGAGGATTTCAGCATTAGAGAGGAGGCTAATGGAACTCA : 1664
FJ708641  : ----------------------------------------------------------------------------------------GGAAGTTGATTTGGGAGGATTTCAGCATTAGAGAGGAGGCTAATGGAACTCA : 1762
                                                                                                                                                               
                     *      3240         *      3260         *      3280         *      3300         *      3320         *      3340         *      3360       
Genomic   : CAAAGAAGTTATAAGAGAAGTAAACACGACCGTGACTGATAACACTTTAGAGATACGGCTTTACTGGGCAGGGAAAGGCACAATGATCATTCCTCAAAGAGGGTACTATGGCTCTCTTATCTCTGCAGTCTCAGTTTGTC : 3360
NM_102713 : CAAAGAAGTTATAAGAGAAGTAAACACGACCGTGACTGATAACACTTTAGAGATACGGCTTTACTGGGCAGGGAAAGGCACAATGATCATTCCTCAAAGAGGGTACTATGGCTCTCTTATCTCTGCAGTCTCAGTTTGTC : 1804
FJ708641  : CAAAGAAGTTATAAGAGAAGTAAACACGACCGTGACTGATAACACTTTAGAGATACGGCTTTACTGGGCAGGGAAAGGCACAATGATCATTCCTCAAAGAGGGTACTATGGCTCTCTTATCTCTGCAGTCTCAGTTTGTC : 1902
                                                                                                                                                               
                     *      3380         *      3400         *      3420         *      3440         *      3460         *      3480         *      3500       
Genomic   : CCAGTAAGTAAAATTCTGTGGAGGTAAACATGAAGTTTCCCTCAGAAATTTTTATCCTAATTGTTTACTTGTTTTTGTTTTACCAGGTTCAGAATCTGAATGCGGTGGTATGAAGAAGAAAATCTCAAGTCTTTGAGTCC : 3500
NM_102713 : CCAGT-----------------------------------------------------------------------------------TCAGAATCTGAATGCGGTGGTATGAAGAAGAAAATCTCAA------------ : 1849
FJ708641  : CCAGT-----------------------------------------------------------------------------------TCAGAATCTGAATGCGGTGGTATGAAGAAGAAAATCTCAAGTCTTTGAGTCC : 1959
                                                                                                                                                               
                     *      3520         *      3540         *      3560         *      3580         *      3600         *      3620         *      3640       
Genomic   : TTTTGTTCCTGCTAAATCTTGAATATGGATTAATCTTTTTCTCTTTGTTTTCTTTTGTCCGCCTTCAGTTCCAGTGCAATTTCTTCCGGTAAAAAAAGATCATAAACCAACAAAATATCCTCTAATTCTTGGCTTAACAC : 3640
NM_102713 : -------------------------------------------------------------------------------------------------------------------------------------------- :    -
FJ708641  : TTTTGTTCCTGCTAAATCTTGAATATGGATTAATCTTTTTCTCTTTGTTTTCTTTTGTCCGCCTTCAGTTCCAGTGCAATTTCTTCCGGTAAAAAAAGATCATAAACCAACAAAATATCCTCTAATTCTTGGCTTAACAC : 2099
                                                                                                                                                               
                     *      3660         *      3680         *      3700         *      3720         *      3740         *      3760         *      3780       
Genomic   : TCTCTCTTGCTTTCTTGCTCCTGGCCGCATTCTGTTGGAGAAAGTGTGTTAGCAATGCAAATGCAGCAGAACGAGGTATTGAATTCGCTACTTAAAAACCATGTAAAGTTCTGTTTTTTTTTTTGAAAATAAAGAGCTTA : 3780
NM_102713 : --------------------------------------------------------------------------------------------------------------------------------------AGCTTA : 1855
FJ708641  : TCTCTCTTGCTTTCTTGCTCCTGGCCGCATTCTGTTGGAGAAAGTGTGTTAGCAATGCAAATGCAGCAGAACGAGGT--------------------------------------------------------------- : 2176
                                                                                                                                                               
                     *      3800         *      3820         *      3840         *      3860         *      3880         *      3900         *      3920       
Genomic   : AGGGACCTGATCTAAGAACAGGTTCCTTCAGTTTGAGGCAACTAAAAGTCGCGACCAACGATTTTGATCCCTTAAACAAGATTGGAGAAGGCGGCTTTGGATCTGTTTATAAGGTACAAAGGAGTCTTCAAGCTCTAGAA : 3920
NM_102713 : AGGGACCTGATCTAAGAACAGGTTCCTTCAGTTTGAGGCAACTAAAAGTCGCGACCAACGATTTTGATCCCTTAAACAAGATTGGAGAAGGCGGCTTTGGATCTGTTTATAAGG-------------------------- : 1969
FJ708641  : -----------------------TCCTTCAGTTTGAGGCAACTAAAAGTCGCGACCAACGATTTTGATCCCTTAAACAAGATTGGAGAAGGCGGCTTTGGATCTGTTTATAAGG-------------------------- : 2267
                                                                                                                                                               
                     *      3940         *      3960         *      3980         *      4000         *      4020         *      4040         *      4060       
Genomic   : ATCAATGTTAGTGTTCTTAAAAACAGGTGACGGTAAAGCCAAAAATTTGACAATACAGGGGCGATTACCAGATGGAACATTGATTGCGGTAAAGAAGCTATCTTCCAAATCACATCAAGGTAACAAAGAGTTTGTAAACG : 4060
NM_102713 : -----------------------------------------------------------GGCGATTACCAGATGGAACATTGATTGCGGTAAAGAAGCTATCTTCCAAATCACATCAAGGTAACAAAGAGTTTGTAAACG : 2050
FJ708641  : -----------------------------------------------------------GGCGATTACCAGATGGAACATTGATTGCGGTAAAGAAGCTATCTTCCAAATCACATCAAGGTAACAAAGAGTTTGTAAACG : 2348
                                                                                                                                                               
                     *      4080         *      4100         *      4120         *      4140         *      4160         *      4180         *      4200       
Genomic   : AGATCGGTATGATCGCTTGCCTGCAGCACCCGAACCTTGTGAAGCTTTATGGATGCTGTGTTGAGAAAAACCAGCTGCTTCTTGTCTATGAGTACTTGGAGAACAATTGTCTTTCTGATGCATTATTTGGTAAATTTATC : 4200
NM_102713 : AGATCGGTATGATCGCTTGCCTGCAGCACCCGAACCTTGTGAAGCTTTATGGATGCTGTGTTGAGAAAAACCAGCTGCTTCTTGTCTATGAGTACTTGGAGAACAATTGTCTTTCTGATGCATTATTTG----------- : 2179
FJ708641  : AGATCGGTATGATCGCTTGCCTGCAGCACCCGAACCTTGTGAAGCTTTATGGATGCTGTGTTGAGAAAAACCAGCTGCTTCTTGTCTATGAGTACTTGGAGAACAATTGTCTTTCTGATGCATTATTTG----------- : 2477
                                                                                                                                                               
                     *      4220         *      4240         *      4260         *      4280         *      4300         *      4320         *      4340       
Genomic   : TGATAGATGATTGGACCAAATCCTTTGACTGCATTCTCTTCTGTCTTGAGATTGAATATTTGCTTGTAAACTGTGGGAGCAGGAAGAAGCTGTCTCAAACTAGAATGGGGAACAAGACACAAAATATGCTTGGGAATCGC : 4340
NM_102713 : -------------------------------------------------------------------------------CAGGAAGAAGCTGTCTCAAACTAGAATGGGGAACAAGACACAAAATATGCTTGGGAATCGC : 2240
FJ708641  : ----------------------------------------------------------------------------------GAAGAAGCTGTCTCAAACTAGAATGGGGAACAAGACACAAAATATGCTTGGGAATCGC : 2535
                                                                                                                                                               
                     *      4360         *      4380         *      4400         *      4420         *      4440         *      4460         *      4480       
Genomic   : GAGAGGGCTTGCTTTCCTCCATGAAGATTCAGCAGTTAAGATCATTCACCGAGACATTAAAGGGACAAATGTTCTACTTGACAAGGATCTGAACTCAAAGATATCAGATTTCGGGTTGGCCAGGCTCCATGAAGACAATC : 4480
NM_102713 : GAGAGGGCTTGCTTTCCTCCATGAAGATTCAGCAGTTAAGATCATTCACCGAGACATTAAAGGGACAAATGTTCTACTTGACAAGGATCTGAACTCAAAGATATCAGATTTCGGGTTGGCCAGGCTCCATGAAGACAATC : 2380
FJ708641  : GAGAGGGCTTGCTTTCCTCCATGAAGATTCAGCAGTTAAGATCATTCACCGAGACATTAAAGGGACAAATGTTCTACTTGACAAGGATCTGAACTCAAAGATATCAGATTTCGGGTTGGCCAGGCTCCATGAAGACAATC : 2675
                                                                                                                                                               
                     *      4500         *      4520         *      4540         *      4560         *      4580         *      4600         *      4620       
Genomic   : AGAGTCACATTACCACCAGAGTTGCAGGAACAATGTTAGTAGTATATTCAGTCGTACAGATGAGTTCAGCATAAGATGATGTTTCAATAGCAGTTATTGTTATTTTTGTTGCAGAGGATATATGGCTCCTGAATATGCAA : 4620
NM_102713 : AGAGTCACATTACCACCAGAGTTGCAGGAACAAT--------------------------------------------------------------------------------AGGATATATGGCTCCTGAATATGCAA : 2440
FJ708641  : AGAGTCACATTACCACCAGAGTTGCAGGAACAAT--------------------------------------------------------------------------------AGGATATATGGCTCCTGAATATGCAA : 2735
                                                                                                                                                               
                     *      4640         *      4660         *      4680         *      4700         *      4720         *      4740         *      4760       
Genomic   : TGAGGGGTCACCTAACAGAAAAGGCAGATGTTTACAGCTTCGGTGTGGTGGCAATGGAGATTGTTAGCGGAAAGAGCAACGCGAAATACACGCCAGATGACGAATGTTGTGTTGGCCTTCTCGATTGGGTACTGATTCAG : 4760
NM_102713 : TGAGGGGTCACCTAACAGAAAAGGCAGATGTTTACAGCTTCGGTGTGGTGGCAATGGAGATTGTTAGCGGAAAGAGCAACGCGAAATACACGCCAGATGACGAATGTTGTGTTGGCCTTCTCGATTGGG----------- : 2569
FJ708641  : TGAGGGGTCACCTAACAGAAAAGGCAGATGTTTACAGCTTCGGTGTGGTGGCAATGGAGATTGTTAGCGGAAAGAGCAACGCGAAATACACGCCAGATGACGAATGTTGTGTTGGCCTTCTCGATTGGG----------- : 2864
                                                                                                                                                               
   

                                                                                                                                                            
                     *      4780         *      4800         *      4820         *      4840         *      4860         *      4880         *      4900       
Genomic   : TCTTTTTCTCCACAAGAATTCTGCTTTCTCTCAAATAAAAAGGTTCTTAGTTTTCAATTTCAGGCATTTGTGTTGCAAAAGAAAGGGGATATCGCGGAAATTCTGGATCCGAGGCTGGAAGGAATGTTCGATGTGATGGA : 4900
NM_102713 : ----------------------------------------------------------------CATTTGTGTTGCAAAAGAAAGGGGATATCGCGGAAATTCTGGATCCGAGGCTGGAAGGAATGTTCGATGTGATGGA : 2645
FJ708641  : ----------------------------------------------------------------CATTTGTGTTGCAAAAGAAAGGGGATATCGCGGAAATTCTGGATCCGAGGCTGGAAGGAATGTTCGATGTGATGGA : 2940
                                                                                                                                                               
                     *      4920         *      4940         *      4960         *      4980         *      5000         *      5020         *      5040       
Genomic   : GGCAGAGAGAATGATAAAGGTTTCACTTTTATGCGCCAACAAGTCTTCGACATTACGGCCAAACATGTCGCAAGTTGTGAAGATGCTTGAAGGGGAGACTGAGATAGAACAGATCATATCAGACCCTGGAGTGTATAGTG : 5040
NM_102713 : GGCAGAGAGAATGATAAAGGTTTCACTTTTATGCGCCAACAAGTCTTCGACATTACGGCCAAACATGTCGCAAGTTGTGAAGATGCTTGAAGGGGAGACTGAGATAGAACAGATCATATCAGACCCTGGAGTGTATAGTG : 2785
FJ708641  : GGCAGAGAGAATGATAAAGGTTTCACTTTTATGCGCCAACAAGTCTTCGACATTACGGCCAAACATGTCGCAAGTTGTGAAGATGCTTGAAGGGGAGACTGAGATAGAACAGATCATATCAGACCCTGGAGTGTATAGTG : 3080
                                                                                                                                                               
                     *      5060         *      5080         *      5100         *      5120         *      5140         *      5160         
Genomic   : ATAACTTGCACTTCAAGCCATCGTCTTTGTCGTCTGATTATATCTTGTCGATTCCTTCATCATCTGAATCTGCTTATGATCTGTACCCACTCAGCCCCGAGTCCATTGTCTTTACTATACAG : 5162
NM_102713 : ATAACTTGCACTTCAAGCCATCGTCTTTGTCGTCTGATTATATCTTGTCGATTCCTTCATCATCTGAATCTGCTTATGATCTGTACCCACTCAGCCCCGAGTCCATTGTCTTTACTATACAG : 2907
FJ708641  : ATAACTTGCACTTCAAGCCATCGTCTTTGTCGTCTGATTATATCTTGTCGATTCCTTCATCATCTGAATCTGCTTATGATCTGTACCCACTCAGCCCCGAGTCCATTGTCTTTACTATACAG : 3202


At1g35710
                                                                                                                                                               
                     *        20         *        40         *        60         *        80         *       100         *       120         *       140       
Genomic   : ATGGGTTTTGCAGAGAAGAATCTTTATGATTTTCGTTTTCTTTTGTTCATTTCCATTATTTTGAGCTGTTCCATTTCAGCTTCTGCAACTATCGCAGAAGCAAATGCTCTTCTGAAATGGAAATCCACTTTCACGAACTC :  140
NM_103273 : ATGGGTTTTGCAGAGAAGAATCTTTATGATTTTCGTTTTCTTTTGTTCATTTCCATTATTTTGAGCTGTTCCATTTCAGCTTCTGCAACTATCGCAGAAGCAAATGCTCTTCTGAAATGGAAATCCACTTTCACGAACTC :  140
FJ708647  : ATGGGTTTTGCGGAGAAGAATCTTTATGATTTTCGTTTTCTTTTGTTCATTTCCATTATTTTGAGCTGTTCCATTTCAGCTTCTGCAACTATCGCAGAAGCAAATGCTCTTCTGAAATGGAAATCCACTTTCACGAACTC :  140
                                                                                                                                                               
                     *       160         *       180         *       200         *       220         *       240         *       260         *       280       
Genomic   : ATCAAAACTGTCTTCATGGGTCCATGATGCAAACACCAATACTAGCTTTTCCTGCACCAGTTGGTATGGTGTTTCTTGCAACTCACGAGGGAGTATCGAAGAGTTAAACCTCACTAACACTGGTATAGAAGGTACTTTCC :  280
NM_103273 : ATCAAAACTGTCTTCATGGGTCCATGATGCAAACACCAATACTAGCTTTTCCTGCACCAGTTGGTATGGTGTTTCTTGCAACTCACGAGGGAGTATCGAAGAGTTAAACCTCACTAACACTGGTATAGAAGGTACTTTCC :  280
FJ708647  : ATCAAAACTGTCTTCATGGGTCCATGATGCAAACACCAATACTAGCTTTTCCTGCACCAGTTGGTATGGTGTTTCTTGCAACTCACGAGGGAGTATCGAAGAGTTAAACCTCACTAACACTGGTATAGAAGGTACTTTCC :  280
                                                                                                                                                               
                     *       300         *       320         *       340         *       360         *       380         *       400         *       420       
Genomic   : AAGATTTCCCCTTCATTTCTCTCTCAAACCTTGCTTACGTAGATCTTAGCATGAACCTTTTATCTGGAACCATCCCTCCTCAATTCGGAAACCTCTCTAAACTCATCTACTTTGACCTCTCCACAAATCACTTAACCGGA :  420
NM_103273 : AAGATTTCCCCTTCATTTCTCTCTCAAACCTTGCTTACGTAGATCTTAGCATGAACCTTTTATCTGGAACCATCCCTCCTCAATTCGGAAACCTCTCTAAACTCATCTACTTTGACCTCTCCACAAATCACTTAACCGGA :  420
FJ708647  : AAGATTTCCCCTTCATTTCTCTCTCAAACCTTGCTTACGTAGATCTTAGCATGAACCTTTTATCTGGAACCATCCCTCCTCAATTCGGAAACCTCTCTAAACTCATCTACTTTGACCTCTCCACAAATCACTTAACCGGA :  420
                                                                                                                                                               
                     *       440         *       460         *       480         *       500         *       520         *       540         *       560       
Genomic   : GAAATCTCTCCTTCCTTAGGAAATCTCAAGAACTTGACGGTACTTTATCTTCACCAAAACTATCTAACTAGTGTCATTCCCTCGGAACTAGGCAACATGGAATCCATGACAGATTTAGCGCTAAGTCAGAACAAACTTAC :  560
NM_103273 : GAAATCTCTCCTTCCTTAGGAAATCTCAAGAACTTGACGGTACTTTATCTTCACCAAAACTATCTAACTAGTGTCATTCCCTCGGAACTAGGCAACATGGAATCCATGACAGATTTAGCGCTAAGTCAGAACAAACTTAC :  560
FJ708647  : GAAATCTCTCCTTCCTTAGGAAATCTCAAGAACTTGACGGTACTTTATCTTCACCAAAACTATCTAACTAGTGTCATTCCCTCGGAACTAGGCAACATGGAATCCATGACAGATTTAGCGCTAAGTCAGAACAAACTTAC :  560
                                                                                                                                                               
                     *       580         *       600         *       620         *       640         *       660         *       680         *       700       
Genomic   : CGGTTCAATTCCTTCTTCCTTAGGAAATCTCAAGAACTTGATGGTTCTTTATCTTTACGAGAACTATCTAACTGGTGTCATTCCCCCGGAACTAGGTAACATGGAATCCATGACAGATTTAGCTTTAAGTCAGAACAAAC :  700
NM_103273 : CGGTTCAATTCCTTCTTCCTTAGGAAATCTCAAGAACTTGATGGTTCTTTATCTTTACGAGAACTATCTAACTGGTGTCATTCCCCCGGAACTAGGTAACATGGAATCCATGACAGATTTAGCTTTAAGTCAGAACAAAC :  700
FJ708647  : CGGTTCAATTCCTTCTTCCTTAGGAAATCTCAAGAACTTGATGGTTCTTTATCTTTACGAGAACTATCTAACTGGTGTCATTCCCCCGGAACTAGGTAACATGGAATCCATGACAGATTTAGCTTTAAGTCAGAACAAAC :  700
                                                                                                                                                               
                     *       720         *       740         *       760         *       780         *       800         *       820         *       840       
Genomic   : TTACCGGTTCAATTCCTTCTACCTTAGGAAATCTCAAGAACTTGATGGTTCTTTATCTTTACGAGAACTATCTAACTGGTGTCATTCCCCCAGAAATAGGCAACATGGAATCCATGACAAATTTAGCGCTAAGTCAGAAC :  840
NM_103273 : TTACCGGTTCAATTCCTTCTACCTTAGGAAATCTCAAGAACTTGATGGTTCTTTATCTTTACGAGAACTATCTAACTGGTGTCATTCCCCCAGAAATAGGCAACATGGAATCCATGACAAATTTAGCGCTAAGTCAGAAC :  840
FJ708647  : TTACCGGTTCAATTCCTTCTACCTTAGGAAATCTCAAGAACTTGATGGTTCTTTATCTTTACGAGAACTATCTAACTGGTGTCATTCCCCCAGAAATAGGCAACATGGAATCCATGACAAATTTAGCGCTAAGTCAGAAC :  840
                                                                                                                                                               
                     *       860         *       880         *       900         *       920         *       940         *       960         *       980       
Genomic   : AAACTTACTGGTTCAATTCCTTCTTCCTTAGGTAATCTCAAGAACTTGACGCTTCTTTCTCTTTTCCAGAACTATCTAACTGGTGGCATTCCCCCGAAACTAGGCAACATAGAATCCATGATCGATTTAGAGTTGAGTAA :  980
NM_103273 : AAACTTACTGGTTCAATTCCTTCTTCCTTAGGTAATCTCAAGAACTTGACGCTTCTTTCTCTTTTCCAGAACTATCTAACTGGTGGCATTCCCCCGAAACTAGGCAACATAGAATCCATGATCGATTTAGAGTTGAGTAA :  980
FJ708647  : AAACTTACTGGTTCAATTCCTTCTTCCTTAGGTAATCTCAAGAACTTGACGCTTCTTTCTCTTTTCCAGAACTATCTAACTGGTGGCATTCCCCCGAAACTAGGCAACATAGAATCCATGATCGATTTAGAGTTGAGTAA :  980
                                                                                                                                                               
                     *      1000         *      1020         *      1040         *      1060         *      1080         *      1100         *      1120       
Genomic   : CAACAAACTTACCGGTTCAATTCCTTCTTCCTTAGGAAATCTCAAGAATTTGACTATTCTTTATCTTTACGAGAACTATTTAACTGGTGTCATTCCCCCGGAACTAGGCAACATGGAATCGATGATTGATTTACAGTTGA : 1120
NM_103273 : CAACAAACTTACCGGTTCAATTCCTTCTTCCTTAGGAAATCTCAAGAATTTGACTATTCTTTATCTTTACGAGAACTATTTAACTGGTGTCATTCCCCCGGAACTAGGCAACATGGAATCGATGATTGATTTACAGTTGA : 1120
FJ708647  : CAACAAACTTACCGGTTCAATTCCTTCTTCCTTAGGAAATCTCAAGAATTTGACTATTCTTTATCTTTACGAGAACTATTTAACTGGTGTCATTCCCCCGGAACTAGGCAACATGGAATCGATGATTGATTTACAGTTGA : 1120
                                                                                                                                                               
                     *      1140         *      1160         *      1180         *      1200         *      1220         *      1240         *      1260       
Genomic   : ATAACAACAAACTTACCGGTTCAATTCCTTCTTCCTTCGGAAATCTCAAGAACTTGACGTATCTTTATCTTTACCTGAATTATTTAACTGGTGTCATTCCCCAGGAGCTAGGCAACATGGAATCCATGATCAATTTAGAT : 1260
NM_103273 : ATAACAACAAACTTACCGGTTCAATTCCTTCTTCCTTCGGAAATCTCAAGAACTTGACGTATCTTTATCTTTACCTGAATTATTTAACTGGTGTCATTCCCCAGGAGCTAGGCAACATGGAATCCATGATCAATTTAGAT : 1260
FJ708647  : ATAACAACAAACTTACCGGTTCAATTCCTTCTTCCTTCGGAAATCTCAAGAACTTGACGTATCTTTATCTTTACCTGAATTATTTAACTGGTGTCATTCCCCAGGAGCTAGGCAACATGGAATCCATGATCAATTTAGAT : 1260
                                                                                                                                                               
                     *      1280         *      1300         *      1320         *      1340         *      1360         *      1380         *      1400       
Genomic   : TTAAGTCAGAACAAACTTACAGGTTCTGTTCCTGATTCCTTTGGTAACTTTACCAAGCTGGAAAGTTTGTACCTTCGTGTTAACCACCTCTCTGGTGCGATCCCGCCAGGAGTAGCAAACTCTTCACATCTAACTACCTT : 1400
NM_103273 : TTAAGTCAGAACAAACTTACAGGTTCTGTTCCTGATTCCTTTGGTAACTTTACCAAGCTGGAAAGTTTGTACCTTCGTGTTAACCACCTCTCTGGTGCGATCCCGCCAGGAGTAGCAAACTCTTCACATCTAACTACCTT : 1400
FJ708647  : TTAAGTCAGAACAAACTTACAGGTTCTGTTCCTGATTCCTTTGGTAACTTTACCAAGCTGGAAAGTTTGTACCTTCGTGTTAACCACCTCTCTGGTGCGATCCCGCCAGGAGTAGCAAACTCTTCACATCTAACTACCTT : 1400
                                                                                                                                                               
                     *      1420         *      1440         *      1460         *      1480         *      1500         *      1520         *      1540       
Genomic   : GATACTTGACACCAACAACTTCACTGGTTTCTTTCCTGAAACTGTTTGCAAAGGTAGGAAGCTTCAAAATATCTCATTGGATTACAATCACCTCGAAGGTCCTATCCCGAAAAGCTTGAGAGATTGCAAGAGCCTGATCA : 1540
NM_103273 : GATACTTGACACCAACAACTTCACTGGTTTCTTTCCTGAAACTGTTTGCAAAGGTAGGAAGCTTCAAAATATCTCATTGGATTACAATCACCTCGAAGGTCCTATCCCGAAAAGCTTGAGAGATTGCAAGAGCCTGATCA : 1540
FJ708647  : GATACTTGACACCAACAACTTCACTGGTTTCTTTCCTGAAACTGTTTGCAAAGGTAGGAAGCTTCAAAATATCTCATTGGATTACAATCACCTCGAAGGTCCTATCCCGAAAAGCTTGAGAGATTGCAAGAGCCTGATCA : 1540
                                                                                                                                                               
                     *      1560         *      1580         *      1600         *      1620         *      1640         *      1660         *      1680       
Genomic   : GAGCAAGATTTCTAGGAAACAAATTCACTGGCGATATTTTTGAAGCTTTTGGGATTTACCCAGACCTTAACTTCATTGATTTCAGCCACAACAAATTCCACGGTGAGATTTCTAGCAACTGGGAGAAGAGTCCAAAGCTA : 1680
NM_103273 : GAGCAAGATTTCTAGGAAACAAATTCACTGGCGATATTTTTGAAGCTTTTGGGATTTACCCAGACCTTAACTTCATTGATTTCAGCCACAACAAATTCCACGGTGAGATTTCTAGCAACTGGGAGAAGAGTCCAAAGCTA : 1680
FJ708647  : GAGCAAGATTTCTAGGAAACAAATTCACTGGCGATATTTTTGAAGCTTTTGGGATTTACCCAGACCTTAACTTCATTGATTTCAGCCACAACAAATTCCACGGTGAGATTTCTAGCAACTGGGAGAAGAGTCCAAAGCTA : 1680
                                                                                                                                                               
                     *      1700         *      1720         *      1740         *      1760         *      1780         *      1800         *      1820       
Genomic   : GGTGCCTTGATCATGTCAAACAACAACATCACGGGTGCTATCCCAACAGAGATTTGGAACATGACACAACTAGTTGAGCTGGATCTATCTACCAACAACCTCTTTGGTGAACTTCCAGAAGCAATTGGAAATCTCACGAA : 1820
NM_103273 : GGTGCCTTGATCATGTCAAACAACAACATCACGGGTGCTATCCCAACAGAGATTTGGAACATGACACAACTAGTTGAGCTGGATCTATCTACCAACAACCTCTTTGGTGAACTTCCAGAAGCAATTGGAAATCTCACGAA : 1820
FJ708647  : GGTGCCTTGATCATGTCAAACAACAACATCACGGGTGCTATCCCAACAGAGATTTGGAACATGACACAACTAGTTGAGCTGGATCTATCTACCAACAACCTCTTTGGTGAACTTCCAGAAGCAATTGGAAATCTCACGAA : 1820
                                                                                                                                                               
                     *      1840         *      1860         *      1880         *      1900         *      1920         *      1940         *      1960       
Genomic   : TTTGTCGAGGCTACGACTTAATGGGAATCAGTTATCTGGAAGAGTTCCTGCAGGTTTAAGTTTCTTAACCAATCTTGAGTCTCTTGACTTATCCTCAAACAATTTCAGCTCAGAGATCCCACAAACCTTTGACTCCTTTT : 1960
NM_103273 : TTTGTCGAGGCTACGACTTAATGGGAATCAGTTATCTGGAAGAGTTCCTGCAGGTTTAAGTTTCTTAACCAATCTTGAGTCTCTTGACTTATCCTCAAACAATTTCAGCTCAGAGATCCCACAAACCTTTGACTCCTTTT : 1960
FJ708647  : TTTGTCGAGGCTACGACTTAATGGGAATCAGTTATCTGGAAGAGTTCCTGCAGGTTTAAGTTTCTTAACCAATCTTGAGTCTCTTGACTTATCCTCAAACAATTTCAGCTCAGAGATCCCACAAACCTTTGACTCCTTTT : 1960
                                                                                                                                                               
                     *      1980         *      2000         *      2020         *      2040         *      2060         *      2080         *      2100       
Genomic   : TAAAGCTTCATGACATGAACCTGAGCAGAAACAAATTCGACGGAAGCATTCCCCGACTATCAAAGCTGACTCAGTTAACGCAACTTGATCTCAGCCACAACCAGCTCGACGGAGAAATCCCATCACAACTAAGCTCCCTG : 2100
NM_103273 : TAAAGCTTCATGACATGAACCTGAGCAGAAACAAATTCGACGGAAGCATTCCCCGACTATCAAAGCTGACTCAGTTAACGCAACTTGATCTCAGCCACAACCAGCTCGACGGAGAAATCCCATCACAACTAAGCTCCCTG : 2100
FJ708647  : TAAAGCTTCATGACATGAACCTGAGCAGAAACAAATTCGACGGAAGCATTCCCCGACTATCAAAGCTGACTCAGTTAACGCAACTTGATCTCAGCCACAACCAGCTCGACGGAGAAATCCCATCACAACTAAGCTCCCTG : 2100
                                                                                                                                                               
                     *      2120         *      2140         *      2160         *      2180         *      2200         *      2220         *      2240       
Genomic   : CAAAGCCTCGACAAGCTCGACCTCTCACACAACAATCTCTCAGGTCTCATTCCAACAACTTTCGAGGGCATGATAGCGCTGACGAACGTCGATATATCAAACAATAAACTCGAAGGTCCGCTTCCAGATACTCCAACGTT : 2240
NM_103273 : CAAAGCCTCGACAAGCTCGACCTCTCACACAACAATCTCTCAGGTCTCATTCCAACAACTTTCGAGGGCATGATAGCGCTGACGAACGTCGATATATCAAACAATAAACTCGAAGGTCCGCTTCCAGATACTCCAACGTT : 2240
FJ708647  : CAAAGCCTCGACAAGCTCGACCTCTCATACAACAATCTCTCAGGTCTCATTCCAACAACTTTCGAGGGCATGATAGCGCTGACGAACGTCGATATATCAAACAATAAACTCGAAGGTCCGCTTCCAGATACTCCAACGTT : 2240
                                                                                                                                                               
                     *      2260         *      2280         *      2300         *      2320         *      2340         *      2360         *      2380       
Genomic   : TCGAAAAGCAACAGCAGATGCATTGGAGGAAAACATAGGCTTATGCAGTAATATTCCTAAACAAAGATTGAAACCATGCCGTGAGTTGAAGAAACCGAAGAAAAATGGCAACCTTGTTGTGTGGATATTAGTGCCAATCC : 2380
NM_103273 : TCGAAAAGCAACAGCAGATGCATTGGAGGAAAACATAGGCTTATGCAGTAATATTCCTAAACAAAGATTGAAACCATGCCGTGAGTTGAAGAAACCGAAGAAAAATGGCAACCTTGTTGTGTGGATATTAGTGCCAATCC : 2380
FJ708647  : TCGAAAAGCAACAGCAGATGCATTGGAGGAAAACATAGGCTTATGCAGTAATATTCCTAAACAAAGATTGAAACCATGCCGTGAGTTGAAGAAACCGAAGAAAAATGGCAACCTTGTTGTGTGGATATTAGTGCCAATCC : 2380
                                                                                                                                                               

                     *      2400         *      2420         *      2440         *      2460         *      2480         *      2500         *      2520       
Genomic   : TAGGAGTACTCGTCATTCTCTCTATATGTGCAAACACATTCACCTACTGCATTCGGAAACGAAAACTACAAAATGGAAGAAACACAGATCCTGAAACAGGAGAGAATATGTCCATATTCAGCGTTGATGGCAAATTCAAA : 2520
NM_103273 : TAGGAGTACTCGTCATTCTCTCTATATGTGCAAACACATTCACCTACTGCATTCGGAAACGAAAACTACAAAATGGAAGAAACACAGATCCTGAAACAGGAGAGAATATGTCCATATTCAGCGTTGATGGCAAATTCAAA : 2520
FJ708647  : TAGGAGTACTCGTCATTCTCTCTATATGTGCAAACACATTCACCTACTGCATTCGGAAACGAAAACTACAAAATGGAAGAAACACAGATCCT------------------------------------------------ : 2472
                                                                                                                                                               
                     *      2540         *      2560         *      2580         *      2600         *      2620         *      2640         *      2660       
Genomic   : TACCAAGATATCATCGAATCAACAAACGAATTCGATCCAACACACCTCATCGGAACCGGAGGATACAGCAAAGTCTACAGAGCAAACCTCCAAGATACAATCATAGCCGTTAAACGACTACATGACACAATAGACGAAGA : 2660
NM_103273 : TACCAAGATATCATCGAATCAACAAACGAATTCGATCCAACACACCTCATCGGAACCGGAGGATACAGCAAAGTCTACAGAGCAAACCTCCAAGATACAATCATAGCCGTTAAACGACTACATGACACAATAGACGAAGA : 2660
FJ708647  : -----------------------------------------------CATCGGAACCGGAGGATACAGCAAAGTCTACAGAGCAAACCTCCAAGATACAATCATAGCCGTTAAACGACTACATGACACAATAGACGAAGA : 2565
                                                                                                                                                               
                     *      2680         *      2700         *      2720         *      2740         *      2760         *      2780         *      2800       
Genomic   : GATATCAAAGCCAGTTGTGAAGCAAGAGTTCCTAAACGAGGTAAAAGCATTAACAGAGATCCGTCACCGCAACGTTGTGAAGCTCTTCGGCTTCTGCTCCCACCGCCGCCACACATTTCTGATCTACGAGTACATGGAAA : 2800
NM_103273 : GATATCAAAGCCAGTTGTGAAGCAAGAGTTCCTAAACGAGGTAAAAGCATTAACAGAGATCCGTCACCGCAACGTTGTGAAGCTCTTCGGCTTCTGCTCCCACCGCCGCCACACATTTCTGATCTACGAGTACATGGAAA : 2800
FJ708647  : GATATCAAAGCCAGTTGTGAAGCAAGAGTTCCTAAACGAGGTAAAAGCATTAACAGAGATCCGTCACCGCAACGTTGTGAAGCTCTTCGGCTTCTGCTCCCACCGCCGCCACACATTTCTGATCTACGAGTACATGGAAA : 2705
                                                                                                                                                               
                     *      2820         *      2840         *      2860         *      2880         *      2900         *      2920         *      2940       
Genomic   : AAGGAAGTTTAAACAAACTCTTAGCCAACGACGAAGAAGCTAAGCGACTCACTTGGACCAAAAGGATCAACGTCGTGAAAGGTGTGGCTCACGCTTTATCGTATATGCACCATGACCGAATAACACCGATCGTTCACCGT : 2940
NM_103273 : AAGGAAGTTTAAACAAACTCTTAGCCAACGACGAAGAAGCTAAGCGACTCACTTGGACCAAAAGGATCAACGTCGTGAAAGGTGTGGCTCACGCTTTATCGTATATGCACCATGACCGAATAACACCGATCGTTCACCGT : 2940
FJ708647  : AAGGAAGTTTAAACAAACTCTTAGCCAACGACGAAGAAGCTAAGCGACTCACTTGGACCAAAAGGATCAACGTCGTGAAAGGTGTGGCTCACGCTTTATCGTATATGCACCATGACCGAATAACACCGATCGTTCACCGT : 2845
                                                                                                                                                               
                     *      2960         *      2980         *      3000         *      3020         *      3040         *      3060         *      3080       
Genomic   : GATATTAGCAGCGGAAACATCCTTCTCGATAACGATTACACAGCTAAGATCTCCGATTTTGGTACAGCAAAACTTCTAAAGACGGATTCATCAAACTGGTCCGCCGTTGCTGGAACCTACGGCTACGTTGCACCAGGTAA : 3080
NM_103273 : GATATTAGCAGCGGAAACATCCTTCTCGATAACGATTACACAGCTAAGATCTCCGATTTTGGTACAGCAAAACTTCTAAAGACGGATTCATCAAACTGGTCCGCCGTTGCTGGAACCTACGGCTACGTTGCACCAG---- : 3076
FJ708647  : GATATTAGCAGCGGAAACATCCTTCTCGATAACGATTACACAGCTAAGATCTCCGATTTTGGTACAGCAAAACTTCTAAAGACGGATTCATCAAACTGGTCCGCCGTTGCTGGAACCTACGGCTACGTTGCACCAGGTAA : 2985
                                                                                                                                                               
                     *      3100         *      3120         *      3140         *      3160         *      3180         *      3200         *      3220       
Genomic   : ATCTAAATCCCTAAATCTGATTTTGAGCTTATGAAATTTAAATCACTAAATTCGAAATTGAACTTGATTTTTCGCAACAGAGTTTGCTTACACGATGAAGGTGACGGAGAAATGCGATGTGTATAGCTTCGGGGTTTTGA : 3220
NM_103273 : --------------------------------------------------------------------------------AGTTTGCTTACACGATGAAGGTGACGGAGAAATGCGATGTGTATAGCTTCGGGGTTTTGA : 3136
FJ708647  : ATCTAAATCCCTAAATCTGATTTTGAGCTTATGAAATTTAAATCACTAAATTCGAAATTGAACTTGATTTTTCGCAACAGAGTTTGCTTACACGATGAAGGTGACGGAGAAATGCGATGTGTATAGCTTCGGGGTTTTGA : 3125
                                                                                                                                                               
                     *      3240         *      3260         *      3280         *      3300         *      3320         *      3340         *      3360       
Genomic   : TACTCGAATTGATAATTGGGAAACATCCGGGAGATTTGGTATCGAGTTTGTCTTCGTCTCCAGGAGAAGCTCTGTCGCTAAGAAGCATTTCCGATGAACGTGTATTAGAACCACGGGGACAAAATAGAGAGAAGCTCTTG : 3360
NM_103273 : TACTCGAATTGATAATTGGGAAACATCCGGGAGATTTGGTATCGAGTTTGTCTTCGTCTCCAGGAGAAGCTCTGTCGCTAAGAAGCATTTCCGATGAACGTGTATTAGAACCACGGGGACAAAATAGAGAGAAGCTCTTG : 3276
FJ708647  : TACTCGAATTGATAATTGGGAAACATCCGGGAGATTTGGTATCGAGTTTGTCTTCGTCTCCAGGAGAAGCTCTGTCGCTAAGAAGCATTTCCGATGAACGTGTATTAGAACCACGGGGACAAAATAGAGAGAAGCTCTTG : 3265
                                                                                                                                                               
                     *      3380         *      3400         *      3420         *      3440           
Genomic   : AAGATGGTGGAAATGGCTTTATTGTGTTTACAAGCAAATCCAGAATCTCGGCCAACGATGTTGTCAATCTCCACTACATTTTCT : 3444
NM_103273 : AAGATGGTGGAAATGGCTTTATTGTGTTTACAAGCAAATCCAGAATCTCGGCCAACGATGTTGTCAATCTCCACTACATTTTCT : 3360
FJ708647  : AAGATGGTGGAAATGGCTTTATTGTGTTTACAAGCAAATCCAGAATCTCGGCCAACGATGTTGTCAATCTCCACTACATTTTCT : 3349


At1g51860
                                                                                                                                                               
                     *        20         *        40         *        60         *        80         *       100         *       120         *       140       
Genomic   : ATGAAATCTCTTCACTGGTTTTTGCATCTTTTGATCATAGCTTTTACCGTTTTGAGATCAGTGGAAGCTCAAAATCAAGCAGGTTCTTAAAACATGATGATGTTTTGATTGCTCTGTTTTGGTCACAATTAGATGTTTTT :  140
NM_104066 : ATGAAATCTCTTCACTGGTTTTTGCATCTTTTGATCATAGCTTTTACCGTTTTGAGATCAGTGGAAGCTCAAAATCAAG------------------------------------------------------------- :   79
FJ708653  : ATGAAATCTCTTCACTGGTTTTTGCATCTTTTGATCATAGCTTTTACCGTTTTGAGATCAGTGGAAGCTCAAAATCAAG------------------------------------------------------------- :   79
                                                                                                                                                               
                     *       160         *       180         *       200         *       220         *       240         *       260         *       280       
Genomic   : AAGGGTTTCCTTTGATGATTATATATCTTGTTTTGATCATGTACAGGATTTATTAGTTTGGATTGTGGGTTGGTGCCTAAGGAAACTACTTATACAGAGAAGTCGACGAATATAACATACAAATCAGACGTGGATTACAT :  280
NM_104066 : -------------------------------------------CAGGATTTATTAGTTTGGATTGTGGGTTGGTGCCTAAGGAAACTACTTATACAGAGAAGTCGACGAATATAACATACAAATCAGACGTGGATTACAT :  176
FJ708653  : -------------------------------------------CAGGATTTATTAGTTTGGATTGTGGGTTGGTGCCTAAGGAAACTACTTATACAGAGAAGTCGACGAATATAACATACAAATCAGACGTGGATTACAT :  176
                                                                                                                                                               
                     *       300         *       320         *       340         *       360         *       380         *       400         *       420       
Genomic   : CGACAGTGGATTGGTCGGAAAGATCAATGATGCATACAAAACTCAGTTTCAGCAACAGGTTTGGGCCGTGAGAAGCTTCCCTGTAGGTCAAAGAAACTGTTACAATGTCAACCTCACGGCAAACAACAAATATTTGATCA :  420
NM_104066 : CGACAGTGGATTGGTCGGAAAGATCAATGATGCATACAAAACTCAGTTTCAGCAACAGGTTTGGGCCGTGAGAAGCTTCCCTGTAGGTCAAAGAAACTGTTACAATGTCAACCTCACGGCAAACAACAAATATTTGATCA :  316
FJ708653  : CGACAGTGGATTGGTCGGAAAGATCAATGATGCATACAAAACTCAGTTTCAGCAACAGGTTTGGGCCGTGAGAAGCTTCCCTGTAGGTCAAAGAAACTGTTACAATGTCAACCTCACGGCAAACAACAAATATTTGATCA :  316
                                                                                                                                                               
                     *       440         *       460         *       480         *       500         *       520         *       540         *       560       
Genomic   : GAGGAACCTTTGTGTATGGGAATTATGATGGCCTGAATCAGTTCCCAAGTTTTGATCTTCATATCGGTCCTAACAAATGGTCGTCTGTTAAAATACTAGGAGTAACAAATACTTCTATGCATGAGATAATCCATGTCGTA :  560
NM_104066 : GAGGAACCTTTGTGTATGGGAATTATGATGGCCTGAATCAGTTCCCAAGTTTTGATCTTCATATCGGTCCTAACAAATGGTCGTCTGTTAAAATACTAGGAGTAACAAATACTTCTATGCATGAGATAATCCATGTCGTA :  456
FJ708653  : GAGGAACCTTTGTGTATGGGAATTATGATGGCCTGAATCAGTTCCCAAGTTTTGATCTTCATATCGGTCCTAACAAATGGTCGTCTGTTAAAATACTAGGAGTAACAAATACTTCTATGCATGAGATAATCCATGTCGTA :  456
                                                                                                                                                               
                     *       580         *       600         *       620         *       640         *       660         *       680         *       700       
Genomic   : CCACAAGATAGTCTTGAAGTTTGTCTTGTTAAGACTGGACCGACGACACCATTCATTTCGTCGCTGGAGGTTCGTCCATTGAACAATGAAAGTTATCTCACGCAAAGTGGATCATTGATGTTGTTCGCCAGAGTATACTT :  700
NM_104066 : CCACAAGATAGTCTTGAAGTTTGTCTTGTTAAGACTGGACCGACGACACCATTCATTTCGTCGCTGGAGGTTCGTCCATTGAACAATGAAAGTTATCTCACGCAAAGTGGATCATTGATGTTGTTCGCCAGAGTATACTT :  596
FJ708653  : CCACAAGATAGTCTTGAAGTTTGTCTTGTTAAGACTGGACCGACGACACCATTCATTTCGTCGCTGGAGGTTCGTCCATTGAACAATGAAAGTTATCTCACGCAAAGTGGATCATTGATGTTGTTCGCCAGAGTATACTT :  596
                                                                                                                                                               
                     *       720         *       740         *       760         *       780         *       800         *       820         *       840       
Genomic   : TCCATCCAGTTCATCATCTTTCATTAGGTAATATTTGTAGGCAACGAAGAGTTTTTTACTTCTTGTCCTTTGTGTGTGTTATAAGTTAACTAAAAGATCATCTCTTTAGGTATGATGAGGACATACATGACCGTGTTTGG :  840
NM_104066 : TCCATCCAGTTCATCATCTTTCATTAG----------------------------------------------------------------------------------GTATGATGAGGACATACATGACCGTGTTTGG :  654
FJ708653  : TCCATCCAGTTCATCATCTTTCATTAG----------------------------------------------------------------------------------------------------------------- :  623
                                                                                                                                                               
                     *       860         *       880         *       900         *       920         *       940         *       960         *       980       
Genomic   : AATTCATTCACAGATGATGAAACCGTCTGGATAAGTACAGACCTCCCGATCGATACAAGTAACTCCTATGACATGCCTCAATCCGTGATGAAGACAGCTGCTGTCCCTAAAAATGCTAGTGAGCCATGGCTCTTATGGTG :  980
NM_104066 : AATTCATTCACAGATGATGAAACCGTCTGGATAAGTACAGACCTCCCGATCGATACAAGTAACTCCTATGACATGCCTCAATCCGTGATGAAGACAGCTGCTGTCCCTAAAAATGCTAGTGAGCCATGGCTCTTATGGTG :  794
FJ708653  : -------------ATGATGAAACCGTCTGGATAAGTACAGACCTCCCGATCGATACAAGTAACTCCTATGACATGCCTCAATCCGTGATGAAGACAGCTGCTGTCCCTAAAAATGCTAGTGAGCCATGGCTCTTATGGTG :  750
                                                                                                                                                               
                     *      1000         *      1020         *      1040         *      1060         *      1080         *      1100         *      1120       
Genomic   : GACTCTTGATGAGAACACCGCACAATCATATGTATATATGCATTTCGCCGAAGTCCAGAATCTTACAGCAAATGAAACCAGAGAATTCAACATTACTTACAATGGTGGTCTACGTTGGTTCAGCTATTTGAGGCCTCCTA : 1120
NM_104066 : GACTCTTGATGAGAACACCGCACAATCATATGTATATATGCATTTCGCCGAAGTCCAGAATCTTACAGCAAATGAAACCAGAGAATTCAACATTACTTACAATGGTGGTCTACGTTGGTTCAGCTATTTGAGGCCTCCTA :  934
FJ708653  : GACTCTTGATGAGAACACCGCACAATCATATGTATATATGCATTTCGCCGAAGTCCAGAATCTTACAGCAAATGAAACCAGAGAATTCAACATTACTTACAATGGTGGTCTACGTTGGTTCAGCTATTTGAGGCCTCCTA :  890
                                                                                                                                                               
                     *      1140         *      1160         *      1180         *      1200         *      1220         *      1240         *      1260       
Genomic   : ATCTCAGCATTTCAACGATCTTTAATCCAAGGGCAGTGAGTTCTTCAAATGGGATATTTAATTTCACATTCGCAATGACGGGTAACTCAACTCTGCCTCCCCTTCTCAACGCCCTCGAGATTTATACAGTCGTAGACATT : 1260
NM_104066 : ATCTCAGCATTTCAACGATCTTTAATCCAAGGGCAGTGAGTTCTTCAAATGGGATATTTAATTTCACATTCGCAATGACGGGTAACTCAACTCTGCCTCCCCTTCTCAACGCCCTCGAGATTTATACAGTCGTAGACATT : 1074
FJ708653  : ATCTCAGCATTTCAACGATCTTTAATCCAAGGGCAGTGAGTTCTTCAAATGGGATATTTAATTTCACATTCGCAATGACGGGTAACTCAACTCTGCCTCCCCTTCTCAACGCCCTCGAGATTTATACAGTCGTAGACATT : 1030
                                                                                                                                                               
                     *      1280         *      1300         *      1320         *      1340         *      1360         *      1380         *      1400       
Genomic   : CTACAGCTAGAGACAAACAAAGATGAAGGCAAGTGTGGTCTTTCTTTAAAAGAAATAGTACTGTCTTTTTAAAACTTTTCTTTTTGGTTTTTGTGAATAGTTTTCAATGTGATGATCAGTTTCTGCTATGATGAACATCA : 1400
NM_104066 : CTACAGCTAGAGACAAACAAAGATGAAG-------------------------------------------------------------------------------------------TTTCTGCTATGATGAACATCA : 1123
FJ708653  : CTACAGCTAGAGACAAACAAAGATGAAG-------------------------------------------------------------------------------------------TTTCTGCTATGATGAACATCA : 1079
                                                                                                                                                               
                     *      1420         *      1440         *      1460         *      1480         *      1500         *      1520         *      1540       
Genomic   : AAGAAACATATGGTTTAAGCAAAAAGATAAGCTGGCAAGGAGATCCGTGTGCTCCTCAGCTTTATCGGTGGGAAGGTTTAAATTGTAGTTATCCGGACTCCGAGGGATCAAGAATCATATCCTTGTATGTTACGTATTCT : 1540
NM_104066 : AAGAAACATATGGTTTAAGCAAAAAGATAAGCTGGCAAGGAGATCCGTGTGCTCCTCAGCTTTATCGGTGGGAAGGTTTAAATTGTAGTTATCCGGACTCCGAGGGATCAAGAATCATATCCTTG--------------- : 1248
FJ708653  : AAGAAACATATGGTTTAAGCAAAAAGATAAGCTGGCAAGGAGATCCGTGTGCTCCTCAGCTTTATCGGTGGGAAGGTTTAAATTGTAGTTATCCGGACTCCGAGGGATCAAGAATCATATCCTTG--------------- : 1204
                                                                                                                                                               
                     *      1560         *      1580         *      1600         *      1620         *      1640         *      1660         *      1680       
Genomic   : TTATCACAAGGTTCTCTAGTTTTGGTTCTTTCACAAAATTCTAACAATTATGGATACTTTTCTTGTTTCACAAACTTTAGGAACTTGAATGGGAGCGAGTTGACAGGTTCTATAACATCTGACATATCCAAGCTAACACT : 1680
NM_104066 : ---------------------------------------------------------------------------------AACTTGAATGGGAGCGAGTTGACAGGTTCTATAACATCTGACATATCCAAGCTAACACT : 1307
FJ708653  : ---------------------------------------------------------------------------------AACTTGAATGGGAGCGAGTTGACAGGTTCTATAACATCTGACATATCCAAGCTAACACT : 1263
                                                                                                                                                               
                     *      1700         *      1720         *      1740         *      1760         *      1780         *      1800         *      1820       
Genomic   : GTTGACAGTATTGTAAGCATCCGAACTGCAACACCGATAAACTTAGTACGCTTTATTTCTATGGGATTCTGTAATATAATAGTTCTGCTAAAACTTTGGTTTCTCTGTTTAATGAACAGAGATTTATCAAATAATGATTT : 1820
NM_104066 : GTTGACAGTATT-----------------------------------------------------------------------------------------------------------AGATTTATCAAATAATGATTT : 1340
FJ708653  : GTTGACAGTATT-----------------------------------------------------------------------------------------------------------AGATTTATCAAATAATGATTT : 1296
                                                                                                                                                               
                     *      1840         *      1860         *      1880         *      1900         *      1920         *      1940         *      1960       
Genomic   : ATCAGGAGATATTCCAACATTTTTTGCTGAGATGAAGTCGTTGAAACTCATGTGAGTTTCTTTTCACATACTCTCTCAGTCTTTCACTGCACTTATTGTTTTGCTTTGAAAGATTTAGTCTTTTCATTCACAGAAACTTA : 1960
NM_104066 : ATCAGGAGATATTCCAACATTTTTTGCTGAGATGAAGTCGTTGAAACTCAT----------------------------------------------------------------------------------AAACTTA : 1398
FJ708653  : ATCAGGAGATATTCCAACATTTTTTGCTGAGATGAAGTCGTTGAAACTCAT----------------------------------------------------------------------------------AAACTTA : 1354
                                                                                                                                                               
                     *      1980         *      2000         *      2020         *      2040         *      2060         *      2080         *      2100       
Genomic   : AGTGGAAACCCGAACCTTAATCTCACAGCAATTCCAGACTCTCTTCAGCAAAGGGTAAACAGCAAATCTTTAACACTAATGTAAGATGACCACACTCCTCTGAACAGTCCTTATAAGTTATATGTAGTGCTACAATAACT : 2100
NM_104066 : AGTGGAAACCCGAACCTTAATCTCACAGCAATTCCAGACTCTCTTCAGCAAAGGGTAAACAGCAAATCTTTAACACTAAT------------------------------------------------------------ : 1478
FJ708653  : AGTGGAAACCCGAACCTTAATCTCACAGCAATTCCAGACTCTCTTCAGCAAAGGGTAAACAGCAAATCTTTAACACTAAT------------------------------------------------------------ : 1434
                                                                                                                                                               
                     *      2120         *      2140         *      2160         *      2180         *      2200         *      2220         *      2240       
Genomic   : CTATTCTAATCTCTTTTTTTGTTTTCTTTTCTGTTTTCCATCATTTTTAATGATTACAGTTTGGGTGAAAACCTGACTCTGACTCCCAAAAAAGAGAGTAAAAAGGTTCCCATGGTTGCTATCGCAGCGTCAGTGGCTGG : 2240
NM_104066 : -----------------------------------------------------------TTTGGGTGAAAACCTGACTCTGACTCCCAAAAAAGAGAGTAAAAAGGTTCCCATGGTTGCTATCGCAGCGTCAGTGGCTGG : 1559
FJ708653  : -----------------------------------------------------------TTTGGGTGAAAACCTGACTCTGACTCCCAAAAAAGAGAGTAAAAAGGTTCCCATGGTTGCTATCGCAGCGTCAGTGGCTGG : 1515
                                                                                                                                                               
                     *      2260         *      2280         *      2300         *      2320         *      2340         *      2360         *      2380       
Genomic   : CGTGTTCGCTCTGCTCGTTATCTTAGCCATATTTTTTGTCATTAAAAGGAAAAATGTGAAAGCTCATAAGTGTATGAATCTCCACCAGGTCTTGATATTAGTGAAAAATGAAAACTTTTGCTCTTTCATTTCACATGTTT : 2380
NM_104066 : CGTGTTCGCTCTGCTCGTTATCTTAGCCATATTTTTTGTCATTAAAAGGAAAAATGTGAAAGCTCATAAGT--------------------------------------------------------------------- : 1630
FJ708653  : CGTGTTCGCTCTGCTCGTTATCTTAGCCATATTTTTTGTCATTAAAAGGAAAAATGTGAAAGCTCATAAGT--------------------------------------------------------------------- : 1586


                     *      2400         *      2420         *      2440         *      2460         *      2480         *      2500         *      2520       
Genomic   : TTATCTTATTTTACAGCTCCAGGACCACCCCCATTAGTCACTCCCGGTATAGTTAAAAGTGAGACAAGATCATCCAATCCATCAATCATAACAAGGGAACGCAAGATCACGTATCCAGAGGTACTGAAGATGACTAATAA : 2520
NM_104066 : ----------------CTCCAGGACCACCCCCATTAGTCACTCCCGGTATAGTTAAAAGTGAGACAAGATCATCCAATCCATCAATCATAACAAGGGAACGCAAGATCACGTATCCAGAGGTACTGAAGATGACTAATAA : 1754
FJ708653  : ----------------CTCCAGGACCACCCCCATTAGTCACTCCCGGTATAGTTAAAAGTGAGACAAGATCATCCAATCCATCAATCATAACAAGGGAACGCAAGATCACGTATCCAGAGGTACTGAAGATGACTAATAA : 1710
                                                                                                                                                               
                     *      2540         *      2560         *      2580         *      2600         *      2620         *      2640         *      2660       
Genomic   : CTTCGAGAGAGTTCTTGGCAAAGGAGGCTTTGGAACAGTGTATCATGGAAACTTGGATGGTGCTGAAGTGGCAGTGAAAATGCTTTCTCATTCATCAGCTCAAGGTTATAAAGAGTTCAAAGCAGAGGTACTTCAAGAAA : 2660
NM_104066 : CTTCGAGAGAGTTCTTGGCAAAGGAGGCTTTGGAACAGTGTATCATGGAAACTTGGATGGTGCTGAAGTGGCAGTGAAAATGCTTTCTCATTCATCAGCTCAAGGTTATAAAGAGTTCAAAGCAGAGGT----------- : 1883
FJ708653  : CTTCGAGAGAGTTCTTGGCAAAGGAGGCTTTGGAACAGTGTATCATGGAAACTTGGATGGTGCTGAAGTGGCAGTGAAAATGCTTTCTCATTCATCAGCTCAAGGTTATAAAGAGTTCAAAGCAGAGGT----------- : 1839
                                                                                                                                                               
                     *      2680         *      2700         *      2720         *      2740         *      2760         *      2780         *      2800       
Genomic   : AAATGGTTTTGTTAATCCTTTTTTTTAAAATATTACTTTCTCAGTTATGAAGTTGTCATGGGACACTTTGATATCTTTCGTAGGTTGAACTTCTTTTAAGAGTTCACCATAGACATTTGGTGGGACTTGTGGGTTACTGT : 2800
NM_104066 : -------------------------------------------------------------------------------------TGAACTTCTTTTAAGAGTTCACCATAGACATTTGGTGGGACTTGTGGGTTACTGT : 1938
FJ708653  : -------------------------------------------------------------------------------------TGAACTTCTTTTAAGAGTTCACCATAGACATTTGGTGGGACTTGTGGGTTACTGT : 1894
                                                                                                                                                               
                     *      2820         *      2840         *      2860         *      2880         *      2900         *      2920         *      2940       
Genomic   : GATGATGGAGACAACTTGGCTCTGATTTATGAATATATGGCAAATGGAGACCTGAGGGAGAATATGTCAGGTAACATTTTTAATTTAGAGACTGATCTGAGTTTTTTTGCCAAAAAAAAGAGACTCTGATATAGTCGAAT : 2940
NM_104066 : GATGATGGAGACAACTTGGCTCTGATTTATGAATATATGGCAAATGGAGACCTGAGGGAGAATATGTCAGG--------------------------------------------------------------------- : 2009
FJ708653  : GATGATGGAGACAACTTGGCTCTGATTTATGAATATATGGCAAATGGAGACCTGAGGGAGAATATGTCAGG--------------------------------------------------------------------- : 1965
                                                                                                                                                               
                     *      2960         *      2980         *      3000         *      3020         *      3040         *      3060         *      3080       
Genomic   : CTTCCGTGGTTTAAAAATAAAATAGGAAAACGTGGAGGCAATGTCCTTACCTGGGAAAACAGGATGCAAATAGCTGTAGAGGCTGCACAAGGTGAACAAAGTTTTGACTTATTCTCTCTACCTTTGTTTGACTCATTACC : 3080
NM_104066 : --------------------------AAAACGTGGAGGCAATGTCCTTACCTGGGAAAACAGGATGCAAATAGCTGTAGAGGCTGCACAAGG------------------------------------------------ : 2075
FJ708653  : --------------------------AAAACGTGGAGGCAATGTCCTTACCTGGGAAAACAGGATGCAAATAGCTGTAGAGGCTGCACAAGG------------------------------------------------ : 2031
                                                                                                                                                               
                     *      3100         *      3120         *      3140         *      3160         *      3180         *      3200         *      3220       
Genomic   : AAAAAATATTGTTTCTTGATGTTCATTTTATGATGTATGAAAGCAAACTCTACATAAGCAAACACATTTTGTGACAGGGCTGGAGTATCTGCACAATGGATGTAGGCCTCCTATGGTACATAGAGATGTTAAAACTACTA : 3220
NM_104066 : ------------------------------------------------------------------------------GCTGGAGTATCTGCACAATGGATGTAGGCCTCCTATGGTACATAGAGATGTTAAAACTACTA : 2137
FJ708653  : ------------------------------------------------------------------------------GCTGGAGTATCTGCACAATGGATGTAGGCCTCCTATGGTACATAGAGATGTTAAAACTACTA : 2093
                                                                                                                                                               
                     *      3240         *      3260         *      3280         *      3300         *      3320         *      3340         *      3360       
Genomic   : ACATTTTATTGAATGAGCGGTGTGGAGCAAAACTAGCCGACTTTGGGCTCTCGAGATCTTTCCCAATCGATGGCGAATGTCATGTTTCGACAGTGGTTGCGGGTACACCTGGTTACCTAGACCCGGAGTGAGTGAGCTCA : 3360
NM_104066 : ACATTTTATTGAATGAGCGGTGTGGAGCAAAACTAGCCGACTTTGGGCTCTCGAGATCTTTCCCAATCGATGGCGAATGTCATGTTTCGACAGTGGTTGCGGGTACACCTGGTTACCTAGACCCGGAGT----------- : 2266
FJ708653  : ACATTTTATTGAATGAGCGGTGTGGAGCAAAACTAGCCGACTTTGGGCTCTCGAGATCTTTCCCAATCGATGGCGAATGTCATGTTTCGACAGTGGTTGCGGGTACACCTGGTTACCTAGACCCGGAGT----------- : 2222
                                                                                                                                                               
                     *      3380         *      3400         *      3420         *      3440         *      3460         *      3480         *      3500       
Genomic   : ATCAAATTTTAAGAGTTGTGTTGTTGAAGCCAAATACTTATATGAAATTTATATTATTCATCTTATATTATTTTCAGGTACTACAGAACAAACTGGCTAAGCGAGAAGAGTGACGTGTACAGCTTCGGTGTAGTGCTATT : 3500
NM_104066 : -------------------------------------------------------------------------------ACTACAGAACAAACTGGCTAAGCGAGAAGAGTGACGTGTACAGCTTCGGTGTAGTGCTATT : 2327
FJ708653  : -------------------------------------------------------------------------------ACTACAGAACAAACTGGCTAAGCGAGAAGAGTGACGTGTACAGCTTCGGTGTAGTGCTATT : 2283
                                                                                                                                                               
                     *      3520         *      3540         *      3560         *      3580         *      3600         *      3620         *      3640       
Genomic   : AGAGATAGTCACAAACCAGCCTGTGATAGATAAAACCCGGGAGAGACCTCACATCAATGACTGGGTTGGGTTCATGCTCACTAAAGGAGACATCAAGAGCATCGTTGACCCGAAACTGATGGGGGACTATGATACAAACG : 3640
NM_104066 : AGAGATAGTCACAAACCAGCCTGTGATAGATAAAACCCGGGAGAGACCTCACATCAATGACTGGGTTGGGTTCATGCTCACTAAAGGAGACATCAAGAGCATCGTTGACCCGAAACTGATGGGGGACTATGATACAAACG : 2467
FJ708653  : AGAGATAGTCACAAACCAGCCTGTGATAGATAAAACCCGGGAGAGACCTCACATCAATGACTGGGTTGGGTTCATGCTCACTAAAGGAGACATCAAGAGCATCGTTGACCCGAAACTGATGGGGGACTATGATACAAACG : 2423
                                                                                                                                                               
                     *      3660         *      3680         *      3700         *      3720         *      3740         *      3760         *      3780       
Genomic   : GTGCATGGAAGATTGTGGAGCTAGCTCTGGCCTGTGTGAACCCATCTTCGAACCGGAGACCAACAATGGCACACGTTGTGATGGAGCTAAACGACTGTGTGGCCTTAGAAAATGCAAGGCGGCAAGGTAGTGAAGAGATG : 3780
NM_104066 : GTGCATGGAAGATTGTGGAGCTAGCTCTGGCCTGTGTGAACCCATCTTCGAACCGGAGACCAACAATGGCACACGTTGTGATGGAGCTAAACGACTGTGTGGCCTTAGAAAATGCAAGGCGGCAAGGTAGTGAAGAGATG : 2607
FJ708653  : GTGCATGGAAGATTGTGGAGCTAGCTCTGGCCTGTGTGAACCCATCTTCGAACCGGAGACCAACAATGGCACACGTTGTGATGGAGCTAAACGACTGTGTGGCCTTAGAAAATGCAAGGCGGCAAGGTAGTGAAGAGATG : 2563
                                                                                                                                                               
                     *      3800         *      3820         *      3840          
Genomic   : TACTCAATGGGTTCTGTTGACTATAGTCTCTCTTCTACTTCTGACTTTGCTCCTGGAGCCAGA : 3843
NM_104066 : TACTCAATGGGTTCTGTTGACTATAGTCTCTCTTCTACTTCTGACTTTGCTCCTGGAGCCAGA : 2670
FJ708653  : TACTCAATGGGTTCTGTTGACTATAGTCTCTCTTCTACTTCTGACTTTGCTCCTGGAGCCAGA : 2626


At1g53420
                                                                                                                                                               
                     *        20         *        40         *        60         *        80         *       100         *       120         *       140       
Genomic   : ATGTCGTTAAATCGGTTTCTCTTCACTTCCTTCTCGTTCTTTCTCTTCTTCATCGTCCATTTCGCATCGTCTGCAACTTTGCCAACACAAGAAGGTAGATATATGCATATATAATATGCTTTTAATATTCGTGTAATTTG :  140
NM_104220 : ATGTCGTTAAATCGGTTTCTCTTCACTTCCTTCTCGTTCTTTCTCTTCTTCATCGTCCATTTCGCATCGTCTGCAACTTTGCCAACACAAGAAGG--------------------------------------------- :   95
FJ708656  : ATGTCGTTAAATCGGTTTCTCTTCACTTCCTTCTCGTTCTTTCTCTTCTTCATCGTCCATTTCGCATCGTCTGCAACTTTGCCAACACAAGAAGG--------------------------------------------- :   95
                                                                                                                                                               
                     *       160         *       180         *       200         *       220         *       240         *       260         *       280       
Genomic   : TATCATTTCTATGATCTGTGTGTGGAATTCTTTGTAGGGGAGGCTTTTAAAGTTGTACTAACCACATTGAAAAAGACGAATATTGATTTAAATGTGGATCCATGCGAAGTCTCATCTACTGGAAATGAATGGTCAACCAT :  280
NM_104220 : --------------------------------------GGAGGCTTTTAAAGTTGTACTAACCACATTGAAAAAGACGAATATTGATTTAAATGTGGATCCATGCGAAGTCTCATCTACTGGAAATGAATGGTCAACCAT :  197
FJ708656  : --------------------------------------GGAGGCTTTTAAAGTTGTACTAACCACATTGAAAAAGACGAATATTGATTTAAATGTGGATCCATGCGAAGTCTCATCTACTGGAAATGAATGGTCAACCAT :  197
                                                                                                                                                               
                     *       300         *       320         *       340         *       360         *       380         *       400         *       420       
Genomic   : CAGCAGGTGGAAAGACCTAGTAACTTGTGACTGCTCCTTCGTTAATGGAACCATTTGCCACATTACCAAAATGTTCGTCTCCCTTAATTTTATTTTATAAAATCTATTTGGTTTTGTTGAGTCAATTTTGTGATTTTTTT :  420
NM_104220 : CAGCAG-------------------------------------------------------------------------------------------------------------------------------------- :  203
FJ708656  : CAGCAGGTGGAAAGACCTAGTAACTTGTGACTGCTCCTTCGTTAATGGAACCATTTGCCACATTACCAAAAT-------------------------------------------------------------------- :  269
                                                                                                                                                               
                     *       440         *       460         *       480         *       500         *       520         *       540         *       560       
Genomic   : TGGTTGATATTTGACACAGAAATTTGAAGAGAGAAAATCTTCAAGGGTCTCTTCCAAAAGAGTTAGTGGGACTTCCTTTACTGCAAGAGATGTAAGCAAGAACCCAAAGAATCGATCGACGAAATTACAAAGTTGCATCT :  560
NM_104220 : -------------------AAATTTGAAGAGAGAAAATCTTCAAGGGTCTCTTCCAAAAGAGTTAGTGGGACTTCCTTTACTGCAAGAGAT------------------------------------------------- :  275
FJ708656  : -------------------AAATTTGAAGAGAGAAAATCTTCAAGGGTCTCTTCCAAAAGAGTTAGTGGGACTTCCTTTACTGCAAGAGAT------------------------------------------------- :  341
                                                                                                                                                               
                     *       580         *       600         *       620         *       640         *       660         *       680         *       700       
Genomic   : TTAAAAATGATATGAAGTAGTGTCATTCTTTTTGTTGTAGTGATCTCTCAAGAAACTATCTCAATGGTTCCATCCCTCCTGAATGGGGAGTCTTGCCACTTGTCAACATGTACATACACATATCTCCATAGCTGCTCATA :  700
NM_104220 : ----------------------------------------TGATCTCTCAAGAAACTATCTCAATGGTTCCATCCCTCCTGAATGGGGAGTCTTGCCACTTGTCAACAT------------------------------- :  344
FJ708656  : ----------------------------------------TGATCTCTCAAGAAACTATCTCAATGGTTCCATCCCTCCTGAATGGGGAGTCTTGCCACTTGTCAACAT------------------------------- :  410
                                                                                                                                                               
                     *       720         *       740         *       760         *       780         *       800         *       820         *       840       
Genomic   : CCATTTCAATATATATTGGACTTACTTTGTTGATGAAATGCAGTTGGCTTCTCGGAAACCGATTAACAGGTCCAATCCCTAAGGAGTTTGGAAACATTACAACTCTTACTAGCCTGTGAGTTCAACTAATTTTCAATCTA :  840
NM_104220 : -------------------------------------------TTGGCTTCTCGGAAACCGATTAACAGGTCCAATCCCTAAGGAGTTTGGAAACATTACAACTCTTACTAGCCT------------------------- :  416
FJ708656  : -------------------------------------------TTGGCTTCTCGGAAACCGATTAACAGGTCCAATCCCTAAGGAGTTTGGAAACATTACAACTCTTACTAGCCT------------------------- :  482
                                                                                                                                                               
                     *       860         *       880         *       900         *       920         *       940         *       960         *       980       
Genomic   : TTATAACCAATGTTTTCAGATAGTTTATGTTTAGAAGTAACTTAATCAAAAAAACTTATGATTTTAGTGTCTTGGAAGCCAATCAACTTTCAGGAGAATTACCTCTTGAGCTAGGGAATCTACCAAACATTCAACAAATG :  980
NM_104220 : -------------------------------------------------------------------TGTCTTGGAAGCCAATCAACTTTCAGGAGAATTACCTCTTGAGCTAGGGAATCTACCAAACATTCAACAAATG :  489
FJ708656  : -------------------------------------------------------------------TGTCTTGGAAGCCAATCAACTTTCAGGAGAATTACCTCTTGAGCTAGGGAATCTACCAAACATTCAACAAATG :  555
                                                                                                                                                               
                     *      1000         *      1020         *      1040         *      1060         *      1080         *      1100         *      1120       
Genomic   : TAAGATTATTCGTACGAGTTTAAGTTTAGTTTTTGTATTGTTGAAACTTGAAACCTCGATTTGTCATTGTTCAGGATTCTTAGCTCGAATAACTTTAATGGTGAAATTCCATCTACATTTGCAAAACTAACCACCTTGAG : 1120
NM_104220 : ---------------------------------------------------------------------------ATTCTTAGCTCGAATAACTTTAATGGTGAAATTCCATCTACATTTGCAAAACTAACCACCTTGAG :  554
FJ708656  : TAAGATTATTCGTACGAGTTTAAGTTTAGTTTTTGTATTGTTGAAACTTGAAACCTCGATTTGTCATTGTTCAGGATTCTTAGCTCGAATAACTTTAATGGTGAAATTCCATCTACATTTGCAAAACTAACCACCTTGAG :  695
                                                                                                                                                               
                     *      1140         *      1160         *      1180         *      1200         *      1220         *      1240         *      1260       
Genomic   : AGATTTGTAAGTATTCTTCTTTGATTCATAGAGAATTTTTTTTTTATTTGCTACTATATATATGCAATTCTGATATTTTTTGCTAATGCATATAATTTTGTACATAGTCGTGTAAGTGACAACCAGTTGTCAGGAACAAT : 1260
NM_104220 : AGATTT-----------------------------------------------------------------------------------------------------TCGTGTAAGTGACAACCAGTTGTCAGGAACAAT :  593
FJ708656  : AGATTT-----------------------------------------------------------------------------------------------------TCGTGTAAGTGACAACCAGTTGTCAGGAACAAT :  734
                                                                                                                                                               
                     *      1280         *      1300         *      1320         *      1340         *      1360         *      1380         *      1400       
Genomic   : TCCAGATTTCATCCAAAAATGGACTAAGCTTGAGCGACTGTAAGATCTTTGTTTACGGTTTGAGATTGTTACTTCCTTTAAACATTCTTGATTTAAGTAATTATAATGTTATGGTTACCTCAGGTTTATTCAAGCAAGTG : 1400
NM_104220 : TCCAGATTTCATCCAAAAATGGACTAAGCTTGAGCGACTGT------------------------------------------------------------------------------------TTATTCAAGCAAGTG :  649
FJ708656  : TCCAGATTTCATCCAAAAATGGACTAAGCTTGAGCGACTGT------------------------------------------------------------------------------------TTATTCAAGCAAGTG :  790
                                                                                                                                                               
                     *      1420         *      1440         *      1460         *      1480         *      1500         *      1520         *      1540       
Genomic   : GTTTAGTTGGACCAATTCCCATTGCCATTGCTTCTCTCGTAGAGTTAAAAGACTTGTATGTATAACTAACTACATTGGCGTCATGTTAGTTGTTAACTTTTCTTCAATGTATATTGACTTTATTATTCTTGTAAATGCAT : 1540
NM_104220 : GTTTAGTTGGACCAATTCCCATTGCCATTGCTTCTCTCGTAGAGTTAAAAGACTTG------------------------------------------------------------------------------------ :  705
FJ708656  : GTTTAGTTGGACCAATTCCCATTGCCATTGCTTCTCTCGTAGAGTTAAAAGACTTG------------------------------------------------------------------------------------ :  846
                                                                                                                                                               
                     *      1560         *      1580         *      1600         *      1620         *      1640         *      1660         *      1680       
Genomic   : TTCTTAGGAGAATCAGCGATTTGAATGGACCCGAATCTCCATTTCCACAGTTAAGGAACATAAAAAAGATGGAGACATTGTGAGTCTTATAGATAAAACTACATTTGGAACCTTTTTGGACGTTGAAAGCTAATTCACAA : 1680
NM_104220 : --------AGAATCAGCGATTTGAATGGACCCGAATCTCCATTTCCACAGTTAAGGAACATAAAAAAGATGGAGACATT------------------------------------------------------------- :  776
FJ708656  : --------AGAATCAGCGATTTGAATGGACCCGAATCTCCATTTCCACAGTTAAGGAACATAAAAAAGATGGAGACATT------------------------------------------------------------- :  917
                                                                                                                                                               
                     *      1700         *      1720         *      1740         *      1760         *      1780         *      1800         *      1820       
Genomic   : GCTCTTTTTTTTTCTTGTGACATTGTAGAATTCTTAGGAACTGCAATCTCACAGGAGACTTACCTGATTATCTTGGAAAGATTACCTCCTTCAAATTCTTGTTAGTATATTTACAAGTTTATAGTCAATCTCTACTAGCT : 1820
NM_104220 : ----------------------------AATTCTTAGGAACTGCAATCTCACAGGAGACTTACCTGATTATCTTGGAAAGATTACCTCCTTCAAATTCTT---------------------------------------- :  848
FJ708656  : ----------------------------AATTCTTAGGAACTGCAATCTCACAGGAGACTTACCTGATTATCTTGGAAAGATTACCTCCTTCAAATTCTT---------------------------------------- :  989
                                                                                                                                                               
                     *      1840         *      1860         *      1880         *      1900         *      1920         *      1940         *      1960       
Genomic   : AGGATGAAATCAAAACTAAGCTAACTTTGTAATAATCTTTTTGTAGAGATCTTAGCTTCAATAAATTAAGTGGAGCCATCCCTAACACTTATATTAATCTTAGAGACGGAGGTTACATGTATGAAATCATTCTCTTCTTT : 1960
NM_104220 : ----------------------------------------------AGATCTTAGCTTCAATAAATTAAGTGGAGCCATCCCTAACACTTATATTAATCTTAGAGACGGAGGTTACAT---------------------- :  920
FJ708656  : ----------------------------------------------AGATCTTAGCTTCAATAAATTAAGTGGAGCCATCCCTAACACTTATATTAATCTTAGAGACGGAGGTTACAT---------------------- : 1061
                                                                                                                                                               
                     *      1980         *      2000         *      2020         *      2040         *      2060         *      2080         *      2100       
Genomic   : CTTATTTTTGAGAAATTGCTGAGAATTTTGTTAGACTTGTTTCTTTCTTACAGATATTTTACAGGAAACATGTTAAACGGGTCGGTTCCAGATTGGATGGTAAATAAAGGATATAAAATGTGAGTTTTAAAACTTTTTTG : 2100
NM_104220 : -----------------------------------------------------ATATTTTACAGGAAACATGTTAAACGGGTCGGTTCCAGATTGGATGGTAAATAAAGGATATAAAAT--------------------- :  986
FJ708656  : -----------------------------------------------------ATATTTTACAGGAAACATGTTAAACGGGTCGGTTCCAGATTGGATGGTAAATAAAGGATATAAAAT--------------------- : 1127
                                                                                                                                                               
                     *      2120         *      2140         *      2160         *      2180         *      2200         *      2220         *      2240       
Genomic   : TAGGGTGCATTACGAAAAAAATTATATGTAATTACTTCATAGATTATTAACATGTTTCTTCGGTTACTGGATTATATTTTGTAGTGATCTTAGTTACAACAATTTCTCAGTAGATCCAACCAATGCAGTATGCAAGTACA : 2240
NM_104220 : ------------------------------------------------------------------------------------TGATCTTAGTTACAACAATTTCTCAGTAGATCCAACCAATGCAGTATGCAAGTACA : 1042
FJ708656  : ------------------------------------------------------------------------------------TGATCTTAGTTACAACAATTTCTCAGTAGATCCAACCAATGCAGTATGCAAGTACA : 1183
                                                                                                                                                               
                     *      2260         *      2280         *      2300         *      2320         *      2340         *      2360         *      2380       
Genomic   : ATAATGTGTAAGAAAACATTACACCTTGGAAACTCTCATGGCTATTTGTTTTTCGTAATTTTAAGAGACTAATTAAAAGATTAACAAATGTTTTGTTTGTTATAGACTTTCATGCATGAGAAACTACCAATGTCCTAAAA : 2380
NM_104220 : ATAATGT--------------------------------------------------------------------------------------------------ACTTTCATGCATGAGAAACTACCAATGTCCTAAAA : 1084
FJ708656  : ATAATGT--------------------------------------------------------------------------------------------------ACTTTCATGCATGAGAAACTACCAATGTCCTAAAA : 1225
                                                                                                                                                               
                                                                                                                                                               
                     *      2400         *      2420         *      2440         *      2460         *      2480         *      2500         *      2520       
Genomic   : GTAAGTTTGTATACGCATTAATGAAGAGAATGAAATTTTAGTCGGTGCATGTGTTAGTCTGATTTATGCTCTATTTCTTGTGTTATAGCTTTCAATGCTCTTCATATAAACTGTGGTGGGGATGAAATGTCTATCAATGG : 2520
NM_104220 : ----------------------------------------------------------------------------------------CTTTCAATGCTCTTCATATAAACTGTGGTGGGGATGAAATGTCTATCAATGG : 1136
FJ708656  : ----------------------------------------------------------------------------------------CTTTCAATGCTCTTCATATAAACTGTGGTGGGGATGAAATGTCTATCAATGG : 1277
                                                                                                                                                               
                     *      2540         *      2560         *      2580         *      2600         *      2620         *      2640         *      2660       
Genomic   : GACAATATATGAATCTGATAAGTACGATAGACTGGAGAGTTGGTACGAAAGTCGAAATGGATGGTTCTCAAACAACGTAGGAGTCTTCGTAGACGATAAACATGTTCCCGAGAGAGTAACCATCGAGTCTAACTCATCCG : 2660
NM_104220 : GACAATATATGAATCTGATAAGTACGATAGACTGGAGAGTTGGTACGAAAGTCGAAATGGATGGTTCTCAAACAACGTAGGAGTCTTCGTAGACGATAAACATGTTCCCGAGAGAGTAACCATCGAGTCTAACTCATCCG : 1276
FJ708656  : GACAATATATGAATCTGATAAGTACGATAGACTGGAGAGTTGGTACGAAAGTCGAAATGGATGGTTCTCAAACAACGTAGGAGTCTTCGTAGACGATAAACATGTTCCCGAGAGAGTAACCATCGAGTCTAACTCATCCG : 1417
                                                                                                                                                               
                     *      2680         *      2700         *      2720         *      2740         *      2760         *      2780         *      2800       
Genomic   : AGCTCAATGTGGTTGATTTTGGTCTGTATACGCAAGCTCGCATATCAGCTATCTCACTCACTTACTATGCATTGTGTTTAGAAAATGGAAATTACAACGTTAATCTCCATTTCGCCGAGATTATGTTCAATGGTAACAAC : 2800
NM_104220 : AGCTCAATGTGGTTGATTTTGGTCTGTATACGCAAGCTCGCATATCAGCTATCTCACTCACTTACTATGCATTGTGTTTAGAAAATGGAAATTACAACGTTAATCTCCATTTCGCCGAGATTATGTTCAATGGTAACAAC : 1416
FJ708656  : AGCTCAATGTGGTTGATTTTGGTCTGTATACGCAAGCTCGCATATCAGCTATCTCACTCACTTACTATGCATTGTGTTTAGAAAATGGAAATTACAACGTTAATCTCCATTTCGCCGAGATTATGTTCAATGGTAACAAC : 1557
                                                                                                                                                               
                     *      2820         *      2840         *      2860         *      2880         *      2900         *      2920         *      2940       
Genomic   : AATTATCAAAGCTTGGGTAGACGGTTTTTCGACATATACATTCAGGTAACTAGTAGGACAAAAATATATATTAAATGTCTAAAAAGCTTGGTCACATAATGAATTTTGATGAATCTTTGGTATAATAATGTAGAGGAAGC : 2940
NM_104220 : AATTATCAAAGCTTGGGTAGACGGTTTTTCGACATATACATTCAG----------------------------------------------------------------------------------------AGGAAGC : 1468
FJ708656  : AATTATCAAAGCTTGGGTAGACGGTTTTTCGACATATACATTCAG----------------------------------------------------------------------------------------AGGAAGC : 1609
                                                                                                                                                               
                     *      2960         *      2980         *      3000         *      3020         *      3040         *      3060         *      3080       
Genomic   : TCGAGGTTAAAGATTTCAATATCGCCAAGGAGGCAAAAGATGTTGGAAACGTTGTTATTAAGACATTTCCGGTCGAGATAAAAGATGGAAAGTTGGAGATACGATTGTATTGGGCCGGGAGAGGAACTACAGTCATTCCC : 3080
NM_104220 : TCGAGGTTAAAGATTTCAATATCGCCAAGGAGGCAAAAGATGTTGGAAACGTTGTTATTAAGACATTTCCGGTCGAGATAAAAGATGGAAAGTTGGAGATACGATTGTATTGGGCCGGGAGAGGAACTACAGTCATTCCC : 1608
FJ708656  : TCGAGGTTAAAGATTTCAATATCGCCAAGGAGGCAAAAGATGTTGGAAACGTTGTTATTAAGACATTTCCGGTCGAGATAAAAGATGGAAAGTTGGAGATACGATTGTATTGGGCCGGGAGAGGAACTACAGTCATTCCC : 1749
                                                                                                                                                               
                     *      3100         *      3120         *      3140         *      3160         *      3180         *      3200         *      3220       
Genomic   : AAAGAACGTGTTTATGGTCCTCTCATATCAGCTATATCAGTGGATTCAAGTAAACAATGTCACTTTTTTTTGGTATTTTTCACATTAGAGTTGATTTATTATCTCATTTCTAACGAAAATTTAATACGTTGATGAAAATA : 3220
NM_104220 : AAAGAACGTGTTTATGGTCCTCTCATATCAGCTATATCAGTGGATTCAAGT----------------------------------------------------------------------------------------- : 1659
FJ708656  : AAAGAACGTGTTTATGGTCCTCTCATATCAGCTATATCAGTGGATTCAAGT----------------------------------------------------------------------------------------- : 1800
                                                                                                                                                               
                     *      3240         *      3260         *      3280         *      3300         *      3320         *      3340         *      3360       
Genomic   : GGTGTTAATCCATCTCCTAGAAATGGTATGAGTACTGGTACTCTCCATACATTGGTAGTGATCTTATCCATTTTCATTGTGTTTCTCGTATTTGGAACTCTATGGAAAAAAGGTTACTTGAGATCGAAAAGCCAAATGGA : 3360
NM_104220 : ---GTTAATCCATCTCCTAGAAATGGTATGAGTACTGGTACTCTCCATACATTGGTAGTGATCTTATCCATTTTCATTGTGTTTCTCGTATTTGGAACTCTATGGAAAAAAGGTTACTTGAGATCGAAAAGCCAAATGGA : 1796
FJ708656  : ---GTTAATCCATCTCCTAGAAATGGTATGAGTACTGGTACTCTCCATACATTGGTAGTGATCTTATCCATTTTCATTGTGTTTCTCGTATTTGGAACTCTATGGAAAAAAGGTTACTTGAGATCGAAAAGCCAAATGGA : 1937
                                                                                                                                                               
                     *      3380         *      3400         *      3420         *      3440         *      3460         *      3480         *      3500       
Genomic   : AAAAGGTACTCATCACAAAGAATACTAATAAGTTATGATATTTCTAAGTGATTTTGTGTTTCTTGGTATAATTGTTAGAGCTTTGTTCTTCTCTCAGATTTCAAAAGTTTGGAACTCATGATTGCTTCTTTCTCGTTGAG : 3500
NM_104220 : AAAAG--------------------------------------------------------------------------------------------ATTTCAAAAGTTTGGAACTCATGATTGCTTCTTTCTCGTTGAG : 1844
FJ708656  : AAAAG--------------------------------------------------------------------------------------------ATTTCAAAAGTTTGGAACTCATGATTGCTTCTTTCTCGTTGAG : 1985
                                                                                                                                                               
                     *      3520         *      3540         *      3560         *      3580         *      3600         *      3620         *      3640       
Genomic   : GCAAATCAAAATCGCTACAAACAACTTTGATTCTGCAAATAGGATTGGAGAAGGCGGCTTTGGTCCTGTATACAAGGTAAGAAGAGAAAGAAAGATCACAACTAAGAACAATATATATGTGTATGGATGGATCAATTCTC : 3640
NM_104220 : GCAAATCAAAATCGCTACAAACAACTTTGATTCTGCAAATAGGATTGGAGAAGGCGGCTTTGGTCCTGTATACAAGG--------------------------------------------------------------- : 1921
FJ708656  : GCAAATCAAAATCGCTACAAACAACTTTGATTCTGCAAATAGGATTGGAGAAGGCGGCTTTGGTCCTGTATACAAGG--------------------------------------------------------------- : 2062
                                                                                                                                                               
                     *      3660         *      3680         *      3700         *      3720         *      3740         *      3760         *      3780       
Genomic   : AAAAGTTTGTTGTCTTTAATATGGTTACAGGGAAAGTTATTTGATGGAACAATAATCGCAGTGAAACAACTTTCGACAGGATCAAAACAAGGGAACCGCGAGTTCTTGAACGAGATTGGCATGATTTCAGCTCTGCACCA : 3780
NM_104220 : -------------------------------GAAAGTTATTTGATGGAACAATAATCGCAGTGAAACAACTTTCGACAGGATCAAAACAAGGGAACCGCGAGTTCTTGAACGAGATTGGCATGATTTCAGCTCTGCACCA : 2030
FJ708656  : -------------------------------GAAAGTTATTTGATGGAACAATAATCGCAGTGAAACAACTTTCGACAGGATCAAAACAAGGGAACCGCGAGTTCTTGAACGAGATTGGCATGATTTCAGCTCTGCACCA : 2171
                                                                                                                                                               
                     *      3800         *      3820         *      3840         *      3860         *      3880         *      3900         *      3920       
Genomic   : CCCTAATCTGGTTAAACTATATGGATGTTGCGTTGAAGGAGGCCAACTTTTATTAGTCTATGAGTTTGTAGAAAACAACAGTCTCGCTCGAGCATTGTTTGGTTAGCAACACTCTAGCTCCTTAAATAAACCGTCAAAAC : 3920
NM_104220 : CCCTAATCTGGTTAAACTATATGGATGTTGCGTTGAAGGAGGCCAACTTTTATTAGTCTATGAGTTTGTAGAAAACAACAGTCTCGCTCGAGCATTGTTTGGT------------------------------------- : 2133
FJ708656  : CCCTAATCTGGTTAAACTATATGGATGTTGCGTTGAAGGAGGCCAACTTTTATTAGTCTATGAGTTTGTAGAAAACAACAGTCTCGCTCGAGCATTGTTTGGT------------------------------------- : 2274
                                                                                                                                                               
                     *      3940         *      3960         *      3980         *      4000         *      4020         *      4040         *      4060       
Genomic   : TGAATTTAGTTATGATTTGTTTTCCCTTTCTTGATAAAGGTCCTCAAGAAACTCAGTTGAGATTGGACTGGCCTACAAGACGGAAGATCTGTATCGGAGTGGCGAGAGGGCTAGCATATCTACACGAAGAATCGAGGCTA : 4060
NM_104220 : -----------------------------------------CCTCAAGAAACTCAGTTGAGATTGGACTGGCCTACAAGACGGAAGATCTGTATCGGAGTGGCGAGAGGGCTAGCATATCTACACGAAGAATCGAGGCTA : 2232
FJ708656  : -----------------------------------------CCTCAAGAAACTCAGTTGAGATTGGACTGGCCTACAAGACGGAAGATCTGTATCGGAGTGGCGAGAGGGCTAGCATATCTACACGAAGAATCGAGGCTA : 2373
                                                                                                                                                               
                     *      4080         *      4100         *      4120         *      4140         *      4160         *      4180         *      4200       
Genomic   : AAGATTGTACACAGAGACATCAAAGCCACTAATGTGTTGCTAGACAAACAACTGAATCCGAAAATATCAGACTTTGGTCTTGCCAAGCTTGATGAAGAGGACAGCACTCACATTAGCACTCGAATCGCTGGAACATTGTA : 4200
NM_104220 : AAGATTGTACACAGAGACATCAAAGCCACTAATGTGTTGCTAGACAAACAACTGAATCCGAAAATATCAGACTTTGGTCTTGCCAAGCTTGATGAAGAGGACAGCACTCACATTAGCACTCGAATCGCTGGAACATT--- : 2369
FJ708656  : AAGATTGTACACAGAGACATCAAAGCCACTAATGTGTTGCTAGACAAACAACTGAATCCGAAAATATCAGACTTTGGTCTTGCCAAGCTTGATGAAGAGGACAGCACTCACATTAGCACTCGAATCGCTGGAACATT--- : 2510
                                                                                                                                                               
                     *      4220         *      4240         *      4260         *      4280         *      4300         *      4320         *      4340       
Genomic   : AGTCACAAAATCAATATTCAATGATAAAAGTTTATATGTCATGTGAGATTTGCTTATAAGTTATAGCACATCATCTTTTTGCAACATTTATGCAGTGGTTACATGGCTCCGGAGTACGCCATGAGAGGCCATTTGACAGA : 4340
NM_104220 : -----------------------------------------------------------------------------------------------TGGTTACATGGCTCCGGAGTACGCCATGAGAGGCCATTTGACAGA : 2414
FJ708656  : -----------------------------------------------------------------------------------------------TGGTTACATGGCTCCGGAGTACGCCATGAGAGGCCATTTGACAGA : 2555
                                                                                                                                                               
                     *      4360         *      4380         *      4400         *      4420         *      4440         *      4460         *      4480       
Genomic   : TAAAGCTGACGTTTACAGCTTTGGTATAGTAGCTCTAGAGATTGTTCATGGAAGAAGCAATAAAATAGAACGATCCAAAAACAATACCTTCTATCTTATTGACTGGGTAATGATCTTTTTGATTGATTACATTTCACAGA : 4480
NM_104220 : TAAAGCTGACGTTTACAGCTTTGGTATAGTAGCTCTAGAGATTGTTCATGGAAGAAGCAATAAAATAGAACGATCCAAAAACAATACCTTCTATCTTATTGACTGGGT-------------------------------- : 2522
FJ708656  : TAAAGCTGACGTTTACAGCTTTGGTATAGTAGCTCTAGAGATTGTTCATGGAAGAAGCAATAAAATAGAACGATCCAAAAACAATACCTTCTATCTTATTGACTGGGT-------------------------------- : 2663
                                                                                                                                                               
                     *      4500         *      4520         *      4540         *      4560         *      4580         *      4600         *      4620       
Genomic   : ACTATTGAACAAAAGATCTCAACTATATTTTGTTCTTGTTTCCCTAGGTGGAGGTTTTGAGAGAGAAGAATAATCTGTTGGAACTGGTGGATCCAAGACTAGGATCAGAATATAACAGAGAAGAAGCAATGACCATGATC : 4620
NM_104220 : -------------------------------------------------GGAGGTTTTGAGAGAGAAGAATAATCTGTTGGAACTGGTGGATCCAAGACTAGGATCAGAATATAACAGAGAAGAAGCAATGACCATGATC : 2613
FJ708656  : -------------------------------------------------GGAGGTTTTGAGAGAGAAGAATAATCTGTTGGAACTGGTGGATCCAAGACTAGGATCAGAATATAACAGAGAAGAAGCAATGACCATGATC : 2754
                                                                                                                                                               
                     *      4640         *      4660         *      4680         *      4700         *      4720         *      4740         *      4760       
Genomic   : CAAATTGCGATAATGTGTACAAGCTCAGAACCTTGTGAGAGACCGTCAATGTCAGAAGTGGTGAAGATGTTAGAAGGTAAGAAGATGGTGGAGGTGGAGAAACTTGAAGAAGCTTCAGTCCATAGAGAAACAAAGAGACT : 4760
NM_104220 : CAAATTGCGATAATGTGTACAAGCTCAGAACCTTGTGAGAGACCGTCAATGTCAGAAGTGGTGAAGATGTTAGAAGGTAAGAAGATGGTGGAGGTGGAGAAACTTGAAGAAGCTTCAGTCCATAGAGAAACAAAGAGACT : 2753
FJ708656  : CAAATTGCGATAATGTGTACAAGCTCAGAACCTTGTGAGAGACCGTCAATGTCAGAAGTGGTGAAGATGTTAGAAGGTAAGAAGATGGTGGAGGTGGAGAAACTTGAAGAAGCTTCAGTCCATAGAGAAACAAAGAGACT : 2894


                                                                                                                             
                     *      4780         *      4800         *      4820         *      4840         *      4860             
Genomic   : TGAAAACATGAACACGATGAAGAAGTACTACGAAATGATAGGGCAAGAGATAAGTACAAGCATGAGCATGATCATGAGTGATCGTAGTGAATCATCAGCAGATCAT : 4866
NM_104220 : TGAAAACATGAACACGATGAAGAAGTACTACGAAATGATAGGGCAAGAGATAAGTACAAGCATGAGCATGATCATGAGTGATCGTAGTGAATCATCAGCAGATCAT : 2859
FJ708656  : TGAAAACATGAACACGATGAAGAAGTACTACGAAATGATAGGGCAAGAGATAAGTACAAGCATGAGCATGATCATGAGTGATCGTAGTGAATCATCAGCAGATCAT : 3000


At1g53440                                                                                                                             
                                                                                                                                                               
                     *        20         *        40         *        60         *        80         *       100         *       120         *       140       
Genomic   : ATGGGTTTCTTTTTCTCGACCCGGAAAGGTCTTCTTCTCATCATCTTCATTTGCTTGGATATTTTCGGATCAAATGCTCAACTTTTGCCAGAAGATGAAGGTTTGTATTACCTTCCTTTTTCTTATATTTACATTGCAAT :  140
NM_104222 : ATGGGTTTCTTTTTCTCGACCCGGAAAGGTCTTCTTCTCATCATCTTCATTTGCTTGGATATTTTCGGATCAAATGCTCAACTTTTGCCAGAAGATGAAG---------------------------------------- :  100
FJ708658  : ATGGGTTTCTTTTTCTCGACCCGGAAAGGTCTTCTTCTCATCATCTTCATTTGCTTGGATATTTTCGGATCAAATGCTCAACTTTTGCCAGAAGATGAAG---------------------------------------- :  100
                                                                                                                                                               
                     *       160         *       180         *       200         *       220         *       240         *       260         *       280       
Genomic   : TAACTTTCTTCAATTTTACTCTAATTTCTAAGAAAATTAATTCTATGCTTCCTTCTTGATTTAAACAAAAAGAAAACTTAATCGAAAGAAAGTTATCAGTTGTTAAAAGAAAAATTATATTTTTTGGTGTGGATATTGGT :  280
NM_104222 : -------------------------------------------------------------------------------------------------------------------------------------------- :    -
FJ708658  : -------------------------------------------------------------------------------------------------------------------------------------------- :    -
                                                                                                                                                               
                     *       300         *       320         *       340         *       360         *       380         *       400         *       420       
Genomic   : CGTCATCAATAAATTTGTTGAATGGTATGGGTCATCATGAATCATAAGATCCATAAAATTATCTAATCTATTGTAGGTCAATTTGTAAATGCTTGACTTATTTAAAGATTTGCTAGTTCTAGTATATCATACATAATTTA :  420
NM_104222 : -------------------------------------------------------------------------------------------------------------------------------------------- :    -
FJ708658  : -------------------------------------------------------------------------------------------------------------------------------------------- :    -
                                                                                                                                                               
                     *       440         *       460         *       480         *       500         *       520         *       540         *       560       
Genomic   : CACATTCCGATATTATATAATCTTTTTACAATAAAAAAATTGATTGGTAACACAAAATTCACTACTAATTTTGGTATCCGGTAGAAGAAGCGGTAAATGATATCAAAATAATTAAAAGTGATAAACCGTAGTAAGAGAAT :  560
NM_104222 : -------------------------------------------------------------------------------------------------------------------------------------------- :    -
FJ708658  : -------------------------------------------------------------------------------------------------------------------------------------------- :    -
                                                                                                                                                               
                     *       580         *       600         *       620         *       640         *       660         *       680         *       700       
Genomic   : CCAATAGAAAAGAAAAAAGAAATGATGTTTTTGGTACTATTTTGATGATTAAAACAGTAAGCCCGTGTCTTCTTATTAATAATTGTATTTCAATACATGTGCAATTTGGCCAGTTAGGTCAAAAATTTTACTTAAAAGAT :  700
NM_104222 : -------------------------------------------------------------------------------------------------------------------------------------------- :    -
FJ708658  : -------------------------------------------------------------------------------------------------------------------------------------------- :    -
                                                                                                                                                               
                     *       720         *       740         *       760         *       780         *       800         *       820         *       840       
Genomic   : CGATAGAGACGTTGAGACAGAAATGTTTTTTATTGTATAGTATTTATCTCTCATTCTTATTCATTTTGTTGGACTTTCATTATTTATTATATCAAAGTTCTTACTTTAATTCTCAACCGACAAATTGTTTGACCAATTTT :  840
NM_104222 : -------------------------------------------------------------------------------------------------------------------------------------------- :    -
FJ708658  : -------------------------------------------------------------------------------------------------------------------------------------------- :    -
                                                                                                                                                               
                     *       860         *       880         *       900         *       920         *       940         *       960         *       980       
Genomic   : GCACAGTTCAAACATTGCGAACGATCTTTAGAAAGCTTCAAAACCAAACAGTGAACATCGAAAGAACTTCTTGTTTGGACAGAAAATGGAACTTTGTTGCCGAGTCAACCTCCAAGCTACCAACCAGTAACATTACCTGC :  980
NM_104222 : ------TTCAAACATTGCGAACGATCTTTAGAAAGCTTCAAAACCAAACAGTGAACATCGAAAGAACTTCTTGTTTGGACAGAAAATGGAACTTTGTTGCCGAGTCAACCTCCAAGCTACCAACCAGTAACATTACCTGC :  234
FJ708658  : ----------------------------------------------------------------------------------AAAATGGAACTTTGTTGCCGAGTCAACCTCCAAGCTACCAACCAGTAACATTACCTGC :  158
                                                                                                                                                               
                     *      1000         *      1020         *      1040         *      1060         *      1080         *      1100         *      1120       
Genomic   : GACTGTACTTTCAACGCCAGCTCAGTCTGTCGTGTCACAAACATGTAAGTATTCACATCTCTGTAACTTTCATAACAAGAAACAGAGTTAGGGTTCTGAAATTTCTTTATTTTCATCAACAGACAGCTTAGAGGTTTCAA : 1120
NM_104222 : GACTGTACTTTCAACGCCAGCTCAGTCTGTCGTGTCACAAACAT------------------------------------------------------------------------------ACAGCTTAGAGGTTTCAA :  296
FJ708658  : GACTGTACTTTCAACGCCAGCTCAGTCTGTCGTGTCACAAACAT------------------------------------------------------------------------------ACAGCTTAGAGGTTTCAA :  220
                                                                                                                                                               
                     *      1140         *      1160         *      1180         *      1200         *      1220         *      1240         *      1260       
Genomic   : TTTGCGAGGAATTATACCGCCTGAATTCGGGAACCTCACACGTCTTACAGAGATGTACGAACCATTTTCCATAAAAGGATCTTTGTCTCTTGATATTTTGTCAATGGATTTTACCAAAATGTGAGGCTTGGAACTTAAGC : 1260
NM_104222 : TTTGCGAGGAATTATACCGCCTGAATTCGGGAACCTCACACGTCTTACAGAGAT-------------------------------------------------------------------------------------- :  350
FJ708658  : TTTGCGAGGAATTATACCGCCTGAATTCGGGAACCTCACACGTCTTACAGAGAT-------------------------------------------------------------------------------------- :  274
                                                                                                                                                               
                     *      1280         *      1300         *      1320         *      1340         *      1360         *      1380         *      1400       
Genomic   : AATCTTTGTTTGTGGTTTTTTCTTACAGAGATCTTGTGCTGAACTTTCTCAGTGGAACAATACCTACAACATTGTCTCAAATTCCGCTTGAAATCTTGTAACCATTCTCTCTAATACATGTATGGTTCATTGTTTCTGGT : 1400
NM_104222 : ----------------------------AGATCTTGTGCTGAACTTTCTCAGTGGAACAATACCTACAACATTGTCTCAAATTCCGCTTGAAATCTTG------------------------------------------ :  420
FJ708658  : ----------------------------AGATCTTGTGCTGAACTTTCTCAGTGGAACAATACCTACAACATTGTCTCAAATTCCGCTTGAAATCTTG------------------------------------------ :  344
                                                                                                                                                               
                     *      1420         *      1440         *      1460         *      1480         *      1500         *      1520         *      1540       
Genomic   : GATGATAATAAAGTTATACTGATTCTTGGTTTTGTTTTGGTTTCTACAACTAACAGGGCTGTAACCGGAAACCGACTCTCTGGACCATTTCCTCCTCAGCTCGGACAGATTACTACACTTACTGATGTGTAATGTTACAT : 1540
NM_104222 : ---------------------------------------------------------GCTGTAACCGGAAACCGACTCTCTGGACCATTTCCTCCTCAGCTCGGACAGATTACTACACTTACTGATGTG----------- :  492
FJ708658  : ---------------------------------------------------------GCTGTAACCGGAAACCGACTCTCTGGACCATTTCCTCCTCAGCTCGGACAGATTACTACACTTACTGATGTG----------- :  416
                                                                                                                                                               
                     *      1560         *      1580         *      1600         *      1620         *      1640         *      1660         *      1680       
Genomic   : ATCTAATCTTATTTTCTTCAAACCAGAATTGACGATATTCTAATATGGAATTTCTTTTTTGCAGGATTATGGAAAGTAATTTATTCACAGGACAACTTCCTCCAAACCTAGGGAACTTAAGAAGCTTGAAAAGATTGTAA : 1680
NM_104222 : -----------------------------------------------------------------ATTATGGAAAGTAATTTATTCACAGGACAACTTCCTCCAAACCTAGGGAACTTAAGAAGCTTGAAAAGATTG--- :  564
FJ708658  : -----------------------------------------------------------------ATTATGGAAAGTAATTTATTCACAGGACAACTTCCTCCAAACCTAGGGAACTTAAGAAGCTTGAAAAGATTG--- :  488
                                                                                                                                                               
                     *      1700         *      1720         *      1740         *      1760         *      1780         *      1800         *      1820       
Genomic   : AATCCTTTACTATATATTTTGGTTTTTATCATATACATTGTTATTGTAGTTTTTCTTAGTGCATCATATGGTGTATTTATGATTACAGGCTAATCTCTTCAAATAACATCACGGGTCGGATCCCTGAGTCCTTGAGCAAT : 1820
NM_104222 : -----------------------------------------------------------------------------------------CTAATCTCTTCAAATAACATCACGGGTCGGATCCCTGAGTCCTTGAGCAAT :  615
FJ708658  : -----------------------------------------------------------------------------------------CTAATCTCTTCAAATAACATCACGGGTCGGATCCCCGAGTCCTTGAGCAAT :  539
                                                                                                                                                               
                     *      1840         *      1860         *      1880         *      1900         *      1920         *      1940         *      1960       
Genomic   : CTCAAGAATTTGACTAATTTGTAAGTAGAGATACTGTGAAGAATTTGATTCTCTGGAATGATTCTAAAAGCTTCATATGTATGTTTCTTGCAGTCGGATTGATGGAAACTCTCTATCTGGGAAGATACCTGATTTTATTG : 1960
NM_104222 : CTCAAGAATTTGACTAATTT-------------------------------------------------------------------------TCGGATTGATGGAAACTCTCTATCTGGGAAGATACCTGATTTTATTG :  682
FJ708658  : CTCAAGAATTTGACTAATTT-------------------------------------------------------------------------TCGGATTGATGGAAACTCTCTATCTGGGAAGATACCTGATTTTATTG :  606
                                                                                                                                                               
                     *      1980         *      2000         *      2020         *      2040         *      2060         *      2080         *      2100       
Genomic   : GAAACTGGACTCGGCTCGTTAGGCTGTGAGCTTACTTCTTCTCAAGAAAAAAGAAAACTGTTGGTAAAGACTGAATTGTTGTTAATTTGCAGAGACCTCCAAGGCACATCAATGGAAGGTCCTATTCCAGCTTCGATTTC : 2100
NM_104222 : GAAACTGGACTCGGCTCGTTAGGCT-------------------------------------------------------------------AGACCTCCAAGGCACATCAATGGAAGGTCCTATTCCAGCTTCGATTTC :  755
FJ708658  : GAAACTGGACTCGGCTCGTTAGGCT-------------------------------------------------------------------AGACCTCCAAGGCACATCAATGGAAGGTCCTATTCCAGCTTCGATTTC :  679
                                                                                                                                                               
                     *      2120         *      2140         *      2160         *      2180         *      2200         *      2220         *      2240       
Genomic   : AAACTTGAAAAACCTGACTGAATTGTAAGCAACCCGTACTTAACATTTAACATCTTTTCCAAACTTTTGAGCTTAAAAAATCTCTCTCTGTTTATCAGGAGGATAACAGATTTGCGTGGACCAACTTCTCCTTTTCCAGA : 2240
NM_104222 : AAACTTGAAAAACCTGACTGAATTG--------------------------------------------------------------------------AGGATAACAGATTTGCGTGGACCAACTTCTCCTTTTCCAGA :  821
FJ708658  : AAACTTGAAAAACCTGACTGAATTG--------------------------------------------------------------------------AGGATAACAGATTTGCGTGGACCAACTTCTCCTTTTCCAGA :  745
                                                                                                                                                               
                     *      2260         *      2280         *      2300         *      2320         *      2340         *      2360         *      2380       
Genomic   : CCTTCAAAATATGACGAATATGGAAAGATTGTAAGTTTTTAAGTTTTAACTTTGACTTGAAGTTAAAGAAACTCTGTTTCCATCAAACCAACATTTGAATATCTTCTTTGCTTATTATGAACTTCATCAGGGTACTAAGA : 2380
NM_104222 : CCTTCAAAATATGACGAATATGGAAAGATTG----------------------------------------------------------------------------------------------------GTACTAAGA :  861
FJ708658  : CCTTCAAAATATGACGAATATGGAAAGATTG----------------------------------------------------------------------------------------------------GTACTAAGA :  785
                                                                                                                                                               
                          
                     *      2400         *      2420         *      2440         *      2460         *      2480         *      2500         *      2520       
Genomic   : AACTGTTTGATAAGGGAACCTATACCGGAGTACATCGGTACTTCCATGACTATGTTGAAGTTACTGTAAGTCAAAATTCTCTTGCTAGTTCTTACTGCCTAAAGGCTAAATTTTTTGATCAAGTTCTCACATTAAAGTAT : 2520
NM_104222 : AACTGTTTGATAAGGGAACCTATACCGGAGTACATCGGTACTTCCATGACTATGTTGAAGTTACT--------------------------------------------------------------------------- :  926
FJ708658  : AACTGTTTGATAAGGGAACCTATACCGGAGTACATCGGTACTTCCATGACTATGTTGAAGTTACT--------------------------------------------------------------------------- :  850
                                                                                                                                                               
                     *      2540         *      2560         *      2580         *      2600         *      2620         *      2640         *      2660       
Genomic   : GTATGTGTGTTCAGAGATTTAAGCTCAAACATGTTAAATGGTACAATTCCAGACACATTTCGGAGTCTGAACGCATTCAACTTTATGTAAGTTTATCAATATAGATGATATTTTTAACCGTGACTTGAAAACTGATTCTT : 2660
NM_104222 : --------------AGATTTAAGCTCAAACATGTTAAATGGTACAATTCCAGACACATTTCGGAGTCTGAACGCATTCAACTTTATGTA--------------------------------------------------- : 1001
FJ708658  : --------------AGATTTAAGCTCAAACATGTTAAATGGTACAATTCCAGACACATTTCGGAGTCTGAACGCATTCAACTTTATGTA--------------------------------------------------- :  925
                                                                                                                                                               
                     *      2680         *      2700         *      2720         *      2740         *      2760         *      2780         *      2800       
Genomic   : TTCTTGCTTCTTTAGGTATCTGAATAATAACTCATTGACTGGTCCAGTTCCTCAGTTCATTCTTGATAGTAAACAAAACATGTAAGCAAACTAACTAACTACCTACCTCGAGGTTGAATATTCGGTATCATCAAGGTCAT : 2800
NM_104222 : ------------------TCTGAATAATAACTCATTGACTGGTCCAGTTCCTCAGTTCATTCTTGATAGTAAACAAAACAT----------------------------------------------------------- : 1064
FJ708658  : ------------------TCTGAATAATAACTCATTGACTGGTCCAGTTCCTCAGTTCATTCTTGATAGTAAACAAAACAT----------------------------------------------------------- :  988
                                                                                                                                                               
                     *      2820         *      2840         *      2860         *      2880         *      2900         *      2920         *      2940       
Genomic   : TAATTTCTTGATCTGTTTTCAGAGATTTATCTTACAACAATTTCACTCAGCCACCTACTTTAAGCTGTAATCAGCTTGATGTGTAAGCATTCCTTGTTGTTTCATGTCAGTGATTTTCAGCTTATCTTGTTTACATGTTT : 2940
NM_104222 : ----------------------AGATTTATCTTACAACAATTTCACTCAGCCACCTACTTTAAGCTGTAATCAGCTTGATGT---------------------------------------------------------- : 1124
FJ708658  : ----------------------AGATTTATCTTACAACAATTTCACTCAGCCACCTACTTTAAGCTGTAATCAGCTTGATGT---------------------------------------------------------- : 1048
                                                                                                                                                               
                     *      2960         *      2980         *      3000         *      3020         *      3040         *      3060         *      3080       
Genomic   : TTCTAATCAGCATTTTTGGTTAAAACTTTCAGTAACTTGATCTCCAGCTACCCCTCAGTAACCAATAACTCGTGAGTCTCTTTTCCCTTTCCTTTCGGTTCCCGTAAAACCAAAGATTACTTACAAAGAAATAATATCTA : 3080
NM_104222 : --------------------------------TAACTTGATCTCCAGCTACCCCTCAGTAACCAATAACTC--------------------------------------------------------------------- : 1163
FJ708658  : --------------------------------TAACTTGATCTCCAGCTACCCCTCAGTAACCAATAACTC--------------------------------------------------------------------- : 1087
                                                                                                                                                               
                     *      3100         *      3120         *      3140         *      3160         *      3180         *      3200         *      3220       
Genomic   : CTTGTGTAGTGTCCAATGGTGCTTAAGAAAGGATCTTCCGTGTCCTGGAGACGCACACCGTAAGTGGAAAATTCCAACTCTCACATCAAGATTCAAAACATTTCTTGAAAAAAAGAAGCCCTTATAAAGTTTCTTGCAGA : 3220
NM_104222 : ---------TGTCCAATGGTGCTTAAGAAAGGATCTTCCGTGTCCTGGAGACGCACACC--------------------------------------------------------------------------------A : 1214
FJ708658  : ---------TGTCCAATGGTGCTTAAGAAAGGATCTTCCGTGTCCTGGAGACGCACACC--------------------------------------------------------------------------------A : 1138
                                                                                                                                                               
                     *      3240         *      3260         *      3280         *      3300         *      3320         *      3340         *      3360       
Genomic   : TTCTTCCTTGTTCATTAACTGTGGAGGAAACCGACTCAAGGTTGATAAAGACGAGTATGCGGATGACTTAAACAAAAGAGGAGCATCAACATTCTCTTCTGTCTCTGAAAGATGGGGATACAGTAGTTCTGGAGCTTGGT : 3360
NM_104222 : TTCTTCCTTGTTCATTAACTGTGGAGGAAACCGACTCAAGGTTGATAAAGACGAGTATGCGGATGACTTAAACAAAAGAGGAGCATCAACATTCTCTTCTGTCTCTGAAAGATGGGGATACAGTAGTTCTGGAGCTTGGT : 1354
FJ708658  : TTCTTCCTTGTTCATTAACTGTGGAGGAAACCGACTCAAGGTTGATAAAGACGAGTATGCGGATGACTTAAACAAAAGAGGAGCATCAACATTCTCTTCTGTCTCTGAAAGATGGGGATACAGTAGTTCTGGAGCTTGGT : 1278
                                                                                                                                                               
                     *      3380         *      3400         *      3420         *      3440         *      3460         *      3480         *      3500       
Genomic   : TAGGCAATGACGGTGCCACTTACTTAGCAACAGATACGTTTAACTTGATCAATGAATCAACTCCAGAGTATTACAAAACAGCCCGTCTCGCTTCACAATCACTCAAGTACTATGGACTATGCATGAGAAGAGGAAGTTAC : 3500
NM_104222 : TAGGCAATGACGGTGCCACTTACTTAGCAACAGATACGTTTAACTTGATCAATGAATCAACTCCAGAGTATTACAAAACAGCCCGTCTCGCTTCACAATCACTCAAGTACTATGGACTATGCATGAGAAGAGGAAGTTAC : 1494
FJ708658  : TAGGCAATGACGGTGCCACTTACTTAGCAACAGATACGTTTAACTTGATCAATGAATCAACTCCAGAGTATTACAAAACAGCCCGTCTCGCTTCACAATCACTCAAGTACTATGGACTATGCATGAGAAGAGGAAGTTAC : 1418
                                                                                                                                                               
                     *      3520         *      3540         *      3560         *      3580         *      3600         *      3620         *      3640       
Genomic   : AAAGTTCAGCTCTATTTTGCAGAGATAATGTTCTCAAATGATCAGACTTATAGTAGCTTAGGGCGGCGACTATTCGACATTTATGTTCAAGTGAGTTTTGCAGAACAAACAAGAAAGTTTCGGTTTTCCTATATCACTAA : 3640
NM_104222 : AAAGTTCAGCTCTATTTTGCAGAGATAATGTTCTCAAATGATCAGACTTATAGTAGCTTAGGGCGGCGACTATTCGACATTTATGTTCAAG------------------------------------------------- : 1585
FJ708658  : AAAGTTCAGCTCTATTTTGCAGAGATAATGTTCTCAAATGATCAGACTTATAGTAGCTTAGGGCGGCGACTATTCGACATTTATGTTCAAG------------------------------------------------- : 1509
                                                                                                                                                               
                     *      3660         *      3680         *      3700         *      3720         *      3740         *      3760         *      3780       
Genomic   : GTAAATTTGATTTTGGATTCTCTCGTGCAGGGGATTTTGTTGGAGAGGGACTTTAACATAGCACAGAGAGCAGGTGGAGTTGGTAAACCGTTCCTAAGGCAAGTTGATGAAGTTCAAGTGAATGGAAGTACGTTGGAGAT : 3780
NM_104222 : -------------------------------GGATTTTGTTGGAGAGGGACTTTAACATAGCACAGAGAGCAGGTGGAGTTGGTAAACCGTTCCTAAGGCAAGTTGATGAAGTTCAAGTGAATGGAAGTACGTTGGAGAT : 1694
FJ708658  : -------------------------------GGATTTTGTTGGAGAGGGACTTTAACATAGCACAGAGAGCAGGTGGAGTTGGTAAACCGTTCCTAAGGCAAGTTGATGAAGTTCAAGTGAATGGAAGTACGTTGGAGAT : 1618
                                                                                                                                                               
                     *      3800         *      3820         *      3840         *      3860         *      3880         *      3900         *      3920       
Genomic   : TCATTTGAAGTGGACAGGAAAAGGCACAAACGTAATACCAACAAGAGGTGTTTACGGGCCTCTCATATCCGCTATAACCGTCACACCGAGTGAGTTTTCCGTCCAATCTTTCTCAACTGTTCAAAATAATTTTGGACTGA : 3920
NM_104222 : TCATTTGAAGTGGACAGGAAAAGGCACAAACGTAATACCAACAAGAGGTGTTTACGGGCCTCTCATATCCGCTATAACCGTCACACCGA--------------------------------------------------- : 1783
FJ708658  : TCATTTGAAGTGGACAGGAAAAGGCACAAACGTAATACCAACAAGAGGTGTTTACGGGCCTCTCATATCCGCTATAACCGTCACACCGA--------------------------------------------------- : 1707
                                                                                                                                                               
                     *      3940         *      3960         *      3980         *      4000         *      4020         *      4040         *      4060       
Genomic   : GTGGATGAATGAATGATCTTTATTGCCTGCTGTTATAGATTTCAAGGTTGATACCGGAAAACCATTGTCCAATGGAGTAGTTGCAGGCATTGTAATCGCAGCGTGTGTAGCTTTCGGGTTGCTGGTACTTGTAATCCTCA : 4060
NM_104222 : --------------------------------------ATTTCAAGGTTGATACCGGAAAACCATTGTCCAATGGAGTAGTTGCAGGCATTGTAATCGCAGCGTGTGTAGCTTTCGGGTTGCTGGTACTTGTAATCCTCA : 1885
FJ708658  : --------------------------------------ATTTCAAGGTTGATACCGGAAAACCATTGTCCAATGGAGTAGTTGCAGGCATTGTAATCGCAGCGTGTGTAGCTTTCGGGTTGCTGGTACTTGTAATCCTCA : 1809
                                                                                                                                                               
                     *      4080         *      4100         *      4120         *      4140         *      4160         *      4180         *      4200       
Genomic   : GGCTAACAGGTTACTTAGGTGGAAAAGAAGTGGATGAAAATGGTAACAAAGAAAACATCATTCTCTTGTTCAAAAATGCTCTTTGTCATTTTCAAGACCTGATTGTTTCTTACTTTCTTGTTTGATCAGAAGAGCTTCGG : 4200
NM_104222 : GGCTAACAGGTTACTTAGGTGGAAAAGAAGTGGATGAAAATG---------------------------------------------------------------------------------------AAGAGCTTCGG : 1938
FJ708658  : GGCTAACAGGTTACTTAGGTGGAAAAGAAGTGGATGAAAATG---------------------------------------------------------------------------------------AAGAGCTTCGG : 1862
                                                                                                                                                               
                     *      4220         *      4240         *      4260         *      4280         *      4300         *      4320         *      4340       
Genomic   : GGACTCGATTTGCAGACAGGATCGTTCACATTGAAGCAAATCAAACGTGCTACTAACAACTTTGATCCAGAAAACAAGATTGGAGAAGGAGGATTTGGACCGGTTTATAAGGTGATGATTGCTTTGGCTTTACACCTTCA : 4340
NM_104222 : GGACTCGATTTGCAGACAGGATCGTTCACATTGAAGCAAATCAAACGTGCTACTAACAACTTTGATCCAGAAAACAAGATTGGAGAAGGAGGATTTGGACCGGTTTATAAGG---------------------------- : 2050
FJ708658  : GGACTCGATTTGCAGACAGGATCGTTCACATTGAAGCAAATCAAACGTGCTACTAACAACTTTGATCCAGAAAACAAGATTGGAGAAGGAGGATTTGGACCGGTTTATAAGG---------------------------- : 1974
                                                                                                                                                               
                     *      4360         *      4380         *      4400         *      4420         *      4440         *      4460         *      4480       
Genomic   : TAAGTTCTGTAAGATTGGATTATAAGTATTGATGTGTTTTTGTTATTAGGGTGTTCTCGCTGATGGGATGACCATAGCGGTGAAGCAGCTTTCATCGAAATCTAAGCAAGGAAACCGAGAATTTGTGACTGAGATCGGTA : 4480
NM_104222 : --------------------------------------------------GTGTTCTCGCTGATGGGATGACCATAGCGGTGAAGCAGCTTTCATCGAAATCTAAGCAAGGAAACCGAGAATTTGTGACTGAGATCGGTA : 2140
FJ708658  : --------------------------------------------------GTGTTCTCGCTGATGGGATGACCATAGCGGTGAAGCAGCTTTCATCGAAATCTAAGCAAGGAAACCGAGAATTTGTGACTGAGATCGGTA : 2064
                                                                                                                                                               
                     *      4500         *      4520         *      4540         *      4560         *      4580         *      4600         *      4620       
Genomic   : TGATATCTGCGTTGCAACACCCTAATCTTGTGAAACTTTATGGTTGTTGCATCGAAGGGAAAGAGCTTTTGCTTGTGTATGAATACTTAGAGAACAACAGTCTCGCTCGCGCACTCTTTGGTTAGTTTTTTCTATCCATC : 4620
NM_104222 : TGATATCTGCGTTGCAACACCCTAATCTTGTGAAACTTTATGGTTGTTGCATCGAAGGGAAAGAGCTTTTGCTTGTGTATGAATACTTAGAGAACAACAGTCTCGCTCGCGCACTCTTTGG------------------- : 2261
FJ708658  : TGATATCTGCGTTGCAACACCCTAATCTTGTGAAACTTTATGGTTGTTGCATCGAAGGGAAAGAGCTTTTGCTTGTGTATGAATACTTAGAGAACAACAGTCTCGCTCGCGCACTCTTTGG------------------- : 2185
                                                                                                                                                               
                     *      4640         *      4660         *      4680         *      4700         *      4720         *      4740         *      4760       
Genomic   : TAAGATAGGCAGGCCGAAGTTCTTGATCTTCAAGCTAAAGTCTCTTGGATTTAACAGGCACAGAGAAACAAAGACTTCACTTGGATTGGTCCACGAGGAACAAGGTATGCATAGGGATTGCGAAAGGATTGGCTTATCTA : 4760
NM_104222 : ----------------------------------------------------------CACAGAGAAACAAAGACTTCACTTGGATTGGTCCACGAGGAACAAGGTATGCATAGGGATTGCGAAAGGATTGGCTTATCTA : 2343
FJ708658  : ----------------------------------------------------------CACAGAGAAACAAAGACTTCACTTGGATTGGTCCACGAGGAACAAGGTATGCATAGGGATTGCGAAAGGATTGGCTTATCTA : 2267


                                                                                                                                                               
                     *      4780         *      4800         *      4820         *      4840         *      4860         *      4880         *      4900       
Genomic   : CACGAGGAATCAAGGCTGAAGATTGTGCACAGAGACATTAAAGCGACAAATGTGCTTCTTGATCTGTCTCTAAATGCTAAGATCTCTGATTTTGGTCTAGCGAAACTCGATGAAGAAGAGAATACACATATCAGCACAAG : 4900
NM_104222 : CACGAGGAATCAAGGCTGAAGATTGTGCACAGAGACATTAAAGCGACAAATGTGCTTCTTGATCTGTCTCTAAATGCTAAGATCTCTGATTTTGGTCTAGCGAAACTCGATGAAGAAGAGAATACACATATCAGCACAAG : 2483
FJ708658  : CACGAGGAATCAAGGCTGAAGATTGTGCACAGAGACATTAAAGCGACAAATGTGCTTCTTGATCTGTCTCTAAATGCTAAGATCTCTGATTTTGGTCTAGCGAAACTCGATGAAGAAGAGAATACACATATCAGCACAAG : 2407
                                                                                                                                                               
                     *      4920         *      4940         *      4960         *      4980         *      5000         *      5020         *      5040       
Genomic   : GATTGCAGGAACAATGTGAGTGTTCATTACTTATCTTTTGTAATATTCATCGTGAATGGTTAACACTTACCGACTTAATATTTCATTGATGCAGAGGTTACATGGCTCCAGAGTATGCAATGAGAGGTTACTTAACAGAC : 5040
NM_104222 : GATTGCAGGAACAAT-------------------------------------------------------------------------------AGGTTACATGGCTCCAGAGTATGCAATGAGAGGTTACTTAACAGAC : 2544
FJ708658  : GATTGCAGGAACAAT-------------------------------------------------------------------------------AGGTTACATGGCTCCAGAGTATGCAATGAGAGGTTACTTAACAGAC : 2468
                                                                                                                                                               
                     *      5060         *      5080         *      5100         *      5120         *      5140         *      5160         *      5180       
Genomic   : AAAGCAGATGTTTATAGCTTCGGTGTAGTCTGTTTAGAGATTGTTAGCGGAAAGAGCAACACAAATTACAGACCAAAAGAAGAGTTTATTTACCTTCTTGACTGGGCATATGTCTTGCAAGAACAAGGGAGTCTTCTAGA : 5180
NM_104222 : AAAGCAGATGTTTATAGCTTCGGTGTAGTCTGTTTAGAGATTGTTAGCGGAAAGAGCAACACAAATTACAGACCAAAAGAAGAGTTTATTTACCTTCTTGACTGGGCATATGTCTTGCAAGAACAAGGGAGTCTTCTAGA : 2684
FJ708658  : AAAGCAGATGTTTATAGCTTCGGTGTAGTCTGTTTAGAGATTGTTAGCGGAAAGAGCAACACAAATTACAGACCAAAAGAAGAGTTTATTTACCTTCTTGACTGGGCATATGTCTTGCAAGAACAAGGGAGTCTTCTAGA : 2608
                                                                                                                                                               
                     *      5200         *      5220         *      5240         *      5260         *      5280         *      5300         *      5320       
Genomic   : ACTCGTGGATCCAGATCTCGGTACAAGCTTTTCGAAGAAAGAAGCAATGAGGATGTTGAACATAGCTTTACTCTGTACAAACCCATCTCCAACATTGAGACCACCGATGTCATCTGTTGTAAGTATGCTACAAGGAAAAA : 5320
NM_104222 : ACTCGTGGATCCAGATCTCGGTACAAGCTTTTCGAAGAAAGAAGCAATGAGGATGTTGAACATAGCTTTACTCTGTACAAACCCATCTCCAACATTGAGACCACCGATGTCATCTGTTGTAAGTATGCTACAAGGAAAAA : 2824
FJ708658  : ACTCGTGGATCCAGATCTCGGTACAAGCTTTTCGAAGAAAGAAGCAATGAGGATGTTGAACATAGCTTTACTCTGTACAAACCCATCTCCAACATTGAGACCACCGATGTCATCTGTTGTAAGTATGCTACAAGGAAAAA : 2748
                                                                                                                                                               
                     *      5340         *      5360         *      5380         *      5400         *      5420         *      5440         *      5460       
Genomic   : TCAAAGTCCAACCACCACTGGTGAAACGTGAAGCTGATCCAAGTGGTTCAGCCGCAATGAGGTTTAAGGCCTTAGAGCATTTGTCACAAGACAGCGAGTCACAAGTCTCAACCTATACAAGAAACAAAGAACACAAAAGC : 5460
NM_104222 : TCAAAGTCCAACCACCACTGGTGAAACGTGAAGCTGATCCAAGTGGTTCAGCCGCAATGAGGTTTAAGGCCTTAGAGCATTTGTCACAAGACAGCGAGTCACAAGTCTCAACCTATACAAGAAACAAAGAACACAAAAGC : 2964
FJ708658  : TCAAAGTCCAACCACCACTGGTGAAACGTGAAGCTGATCCAAGTGGTTCAGCCGCAATGAGGTTTAAGGCCTTAGAGCATTTGTCACAAGACAGCGAGTCACAAGTCTCAACCTATACAAGAAACAAAGAACACAAAAGC : 2888
                                                                                                                                                               
                     *      5480         *      5500         *      5520         *      5540         *      5560         *      5580         *      5600       
Genomic   : TCCTCCTCGATGGACGGTCCTTGGGTCGATTCTTCCTTCTCTGATCCGAGCAAAGATGTTAGCCTATTACAGCAAGAAGAAGGAAATTCATCATCTTCGTCGAGGAGACTGTTAGATGATCTTACTGATGTGGAGATTGA : 5600
NM_104222 : TCCTCCTCGATGGACGGTCCTTGGGTCGATTCTTCCTTCTCTGATCCGAGCAAAGATGTTAGCCTATTACAGCAAGAAGAAGGAAATTCATCATCTTCGTCGAGGAGACTGTTAGATGATCTTACTGATGTGGAGATTGA : 3104
FJ708658  : TCCTCCTCGATGGACGGTCCTTGGGTCGATTCTTCCTTCTCTGATCCGAGCAAAGATGTTAGCCTATTACAGCAAGAAGAAGGAAATTCATCATCTTCGTCGAGGAGACTGTTAGATGATCTTACTGATGTGGAGATTGA : 3028
                    
                    
Genomic   : G : 5601
NM_104222 : G : 3105
FJ708658  : G : 3029
                    

At1g56120
                                                                                                                                                               
                     *        20         *        40         *        60         *        80         *       100         *       120         *       140       
Genomic   : ATGGTTCGAGCCCAAAACCGAACCAGACCCACTACTCATCCCGACGATGGTCTGTCTTTTCAACACTTGACAAAAGTTTAGTCACCAATTATCGACTGGATTTGTAGTTTGTAATAGCTAGCTTCTTCTTTGTAATAAAC :  140
NM_104490 : ATGGTTCGAGCCCAAAACCGAACCAGACCCACTACTCATCCCGACGATG------------------------------------------------------------------------------------------- :   49
FJ708661  : ATGGTTCGAGCCCAAAACCGAACCAGACCCACTACTCATCCCGACGATG------------------------------------------------------------------------------------------- :   49
                                                                                                                                                               
                     *       160         *       180         *       200         *       220         *       240         *       260         *       280       
Genomic   : CCCACTAACGAAAAAACAAATAAAATGCATCTTGTCAATAGAATGAGTTTCTTAGTTCAGTTAATATATAGTTTCAATTAATTAATACTAGTTGTTTAGTTAAGCGTAATAGATTAAGGGGTGCTAATAATAATTTGACC :  280
NM_104490 : -------------------------------------------------------------------------------------------------------------------------------------------- :    -
FJ708661  : -------------------------------------------------------------------------------------------------------------------------------------------- :    -
                                                                                                                                                               
                     *       300         *       320         *       340         *       360         *       380         *       400         *       420       
Genomic   : GTTGTGAATTATGTTGGATAAAAACAGCGCGAGCTTTGAACTCCATCTTCGCGGCTTGGAAGATTCGGGCGCCGAGGGAATGGAACATCAGCGGCGAACTTTGCTCCGGCGTCGCCATCGACGCCAGTGTACTCGACTCA :  420
NM_104490 : ---------------------------CGCGAGCTTTGAACTCCATCTTCGCGGCTTGGAAGATTCGGGCGCCGAGGGAATGGAACATCAGCGGCGAACTTTGCTCCGGCGTCGCCATCGACGCCAGTGTACTCGACTCA :  162
FJ708661  : ---------------------------CGCGAGCTTTGAACTCCATCTTCGCGGCTTGGAAGATTCGGGCGCCGAGGGAATGGAACATCAGCGGCGAACTTTGCTCCGGCGTCGCCATCGACGCCAGTGTACTCGACTCA :  162
                                                                                                                                                               
                     *       440         *       460         *       480         *       500         *       520         *       540         *       560       
Genomic   : AACCATGCCTACAATCCTCTCATCAAATGCGACTGTAGTTTCCAAAACTCCACAATCTGCCGCATAAATAACATGTTACACCTCTTTCCCACTTTTTATGCACTCCATATGTTCGACGAATTCTCCCACTGAGTCTCAAT :  560
NM_104490 : AACCATGCCTACAATCCTCTCATCAAATGCGACTGTAGTTTCCAAAACTCCACAATCTGCCGCATAAATAACAT------------------------------------------------------------------ :  236
FJ708661  : AACCATGCCTACAATCCTCTCATCAAATGCGACTGTAGTTTCCAAAACTCCACAATCTGCCGCATAAATAACAT------------------------------------------------------------------ :  236
                                                                                                                                                               
                     *       580         *       600         *       620         *       640         *       660         *       680         *       700       
Genomic   : TTTGTTTGTTGTTGTCTCAGAAAGGTTTATGCGATAGATGTTGTAGGACCTATACCTCCAGAGCTCTGGACCTTAACATACCTCACCAATCTGTATAGTTCCATACAACCCACTTACCCACTTTTTATATTCACAAGTAT :  700
NM_104490 : --------------------AAAGGTTTATGCGATAGATGTTGTAGGACCTATACCTCCAGAGCTCTGGACCTTAACATACCTCACCAATCTG----------------------------------------------- :  309
FJ708661  : --------------------AAAGGTTTATGCGATAGATGTTGTAGGACCTATACCTCCAGAGCTCTGGACCTTAACATACCTCACCAATCTG----------------------------------------------- :  309
                                                                                                                                                               
                     *       720         *       740         *       760         *       780         *       800         *       820         *       840       
Genomic   : ATATATGTTGAGACTCTTAACTGATCCAATGGTCATCAAAAACACACATCTTTCAGGAACCTAGGTCAAAATTATCTCACAGGCTCACTGTCTCCTGCTATTGGGAATTTGACTCGGATGCAGTGGATGTAAAAGCCTCT :  840
NM_104490 : -----------------------------------------------------------------------------------CTCACTGTCTCCTGCTATTGGGAATTTGACTCGGATGCAGTGGATGTAAAAGCCTCT :  366
FJ708661  : ---------------------------------------------------------AACCTAGGTCAAAATTATCTCACAGGCTCACTGTCTCCTGCTATTGGGAATTTGACTCGGATGCAGTGGATG----------- :  381
                                                                                                                                                               
                     *       860         *       880         *       900         *       920         *       940         *       960         *       980       
Genomic   : GAAACCTTGTTTCTTTTTCTTTTTTGATAATTTTATACAAGTTCAATTAACAATTGTTTGTCTCCCATGCTCAAATGTAGGACTTTTGGAATCAACGCGTTGTCTGGCCCTATTCCTAAGGAAATCGGTTTGCTTACGGA :  980
NM_104490 : GAAACCTTG------------------------------------------------------------------------ACTTTTGGAATCAACGCGTTGTCTGGCCCTATTCCTAAGGAAATCGGTTTGCTTACGGA :  434
FJ708661  : ---------------------------------------------------------------------------------ACTTTTGGAATCAACGCGTTGTCTGGCCCTATTCCTAAGGAAATCGGTTTGCTTACGGA :  440
                                                                                                                                                               
                     *      1000         *      1020         *      1040         *      1060         *      1080         *      1100         *      1120       
Genomic   : TTTAAGATTACTGTAAGCTTTGGCTGCTTCCTCATTATCTATATTTGAACATGTCTTCACTCTGCTTATGGAGAGGATTTGATGGTTCTTTATAGCTATATATATTTTCTGCAGTGGTATTAGTTCAAATAACTTTTCCG : 1120
NM_104490 : TTTAAGATTACT------------------------------------------------------------------------------------------------------TGGTATTAGTTCAAATAACTTTTCCG :  472
FJ708661  : TTTAAGATTACT------------------------------------------------------------------------------------------------------TGGTATTAGTTCAAATAACTTTTCCG :  478
                                                                                                                                                               
                     *      1140         *      1160         *      1180         *      1200         *      1220         *      1240         *      1260       
Genomic   : GTTCCTTACCAGCTGAGATTGGGAGTTGTACAAAACTACAGCAAATGTGAGTCTATCTGTCAAATCCATAAGTGCTTATGTTATTGACTAGTTATAATTTAGATATGAAACCTTAACGTCTGAAACTCTTTAGTGTTCGT : 1260
NM_104490 : GTTCCTTACCAGCTGAGATTGGGAGTTGTACAAAACTACAGCAAATGT-------------------------------------------------------------------------------------------- :  520
FJ708661  : GTTCCTTACCAGCTGAGATTGGGAGTTGTACAAAACTACAGCAAATGT-------------------------------------------------------------------------------------------- :  526
                                                                                                                                                               
                     *      1280         *      1300         *      1320         *      1340         *      1360         *      1380         *      1400       
Genomic   : CGACCACAAAAGTCTTGTGCATCTGTTATTCTTAAAAATTTCTTACATCTATTACAGGTACATCGATAGTTCTGGACTCAGCGGAGGAATACCTTTATCATTTGCTAATTTTGTGGAGTTGGAAGTCGCGTAAGATATAA : 1400
NM_104490 : -----------------------------------------------------------ACATCGATAGTTCTGGACTCAGCGGAGGAATACCTTTATCATTTGCTAATTTTGTGGAGTTGGAAGTCGC----------- :  590
FJ708661  : -----------------------------------------------------------ACATCGATAGTTCTGGACTCAGCGGAGGAATACCTTTATCATTTGCTAATTTTGTGGAGTTGGAAGTCGC----------- :  596
                                                                                                                                                               
                     *      1420         *      1440         *      1460         *      1480         *      1500         *      1520         *      1540       
Genomic   : TTTTTATAATCTACTTACTACTTTACATTTTAGTTTTTTGCGTTGGGGTGATTGTTTCCTTAATGTTGTACCAGTTGGATTATGGATGTGGAACTTACAGGTCGGATACCAGACTTTATAGGATTCTGGACCAAACTTAC : 1540
NM_104490 : --------------------------------------------------------------------------TTGGATTATGGATGTGGAACTTACAGGTCGGATACCAGACTTTATAGGATTCTGGACCAAACTTAC :  656
FJ708661  : --------------------------------------------------------------------------TTGGATTATGGATGTGGAACTTACAGGTCGGATACCAGACTTTATAGGATTCTGGACCAAACTTAC :  662
                                                                                                                                                               
                     *      1560         *      1580         *      1600         *      1620         *      1640         *      1660         *      1680       
Genomic   : TACCTTGTAAGGAGATTTCATTTATACGGAGTAATATAGTGGAATAATTCTTCATTGTATCTAAATTGGGAATATACGACTGGTACAATTTACTGCAGGAGGATCCTCGGAACTGGTTTGAGCGGCCCAATACCGTCGTC : 1680
NM_104490 : TACCTTG--------------------------------------------------------------------------------------------AGGATCCTCGGAACTGGTTTGAGCGGCCCAATACCGTCGTC :  704
FJ708661  : TACCTTG--------------------------------------------------------------------------------------------AGGATCCTCGGAACTGGTTTGAGCGGCCCAATACCGTCGTC :  710
                                                                                                                                                               
                     *      1700         *      1720         *      1740         *      1760         *      1780         *      1800         *      1820       
Genomic   : ATTTTCCAACTTAATTGCTTTGACAGAACTGTATGTGTTAAATTGTGTCAGAATCATTATTGTTTACTGTTTCTCTATCTTTCCTTTTGTCCACATATTCATATTCTATATGTAGGAGGCTTGGTGATATATCCAATGGA : 1820
NM_104490 : ATTTTCCAACTTAATTGCTTTGACAGAACTG-------------------------------------------------------------------------------------AGGCTTGGTGATATATCCAATGGA :  759
FJ708661  : ATTTTCCAACTTAATTGCTTTGACAGAACTG-------------------------------------------------------------------------------------AGGCTTGGTGATATATCCAATGGA :  765
                                                                                                                                                               
                     *      1840         *      1860         *      1880         *      1900         *      1920         *      1940         *      1960       
Genomic   : AGCTCTTCTCTTGATTTCATCAAAGACATGAAATCTCTAAGTGTATTGTAAGGCAGCTTTGATTCAACTCCTCCTCCAATTTATGATCAGATTAATGTACTGTTGAGCTGTTTCTATCTCCTCCTGTATTTAGCAATTAT : 1960
NM_104490 : AGCTCTTCTCTTGATTTCATCAAAGACATGAAATCTCTAAGTGTATT--------------------------------------------------------------------------------------------- :  806
FJ708661  : AGCTCTTCTCTTGATTTCATCAAAGACATGAAATCTCTAAGTGTATT--------------------------------------------------------------------------------------------- :  812
                                                                                                                                                               
                     *      1980         *      2000         *      2020         *      2040         *      2060         *      2080         *      2100       
Genomic   : GATATTTGTTGCAGAGTATTGAGGAACAACAATCTCACTGGGACAATACCGTCTACTATTGGAGGATACACAAGTTTGCAACAAGTGTAAGTACATATGTCAGTGTTTTTCTTCACATGCTCTGAAAGCTGTTTCAGGGT : 2100
NM_104490 : --------------AGTATTGAGGAACAACAATCTCACTGGGACAATACCGTCTACTATTGGAGGATACACAAGTTTGCAACAAGT------------------------------------------------------ :  878
FJ708661  : --------------AGTATTGAGGAACAACAATCTCACTGGGACAATACCGTCTACTATTGGAGGATACACAAGTTTGCAACAAGT------------------------------------------------------ :  884
                                                                                                                                                               
                     *      2120         *      2140         *      2160         *      2180         *      2200         *      2220         *      2240       
Genomic   : CAAAAAGAGTGAGATGATTTGTGAAAGTCACAGAAAGAACACGAAATGATCTTAAACTGCTGATAATATAATTTCGTATACTTCCTCATTCATTAGAAAACAGAAATTGTTTCATGTTTTACCGTTTAATTCATGTTTGT : 2240
NM_104490 : -------------------------------------------------------------------------------------------------------------------------------------------- :    -
FJ708661  : -------------------------------------------------------------------------------------------------------------------------------------------- :    -
                                                                                                                                                               
                     *      2260         *      2280         *      2300         *      2320         *      2340         *      2360         *      2380       
Genomic   : CTTCCAGTGATTTGAGCTTCAACAAATTACATGGACCAATTCCGGCTTCACTTTTCAACTTAAGTCGACTTACTCACTTGTAAGATACTAAGATGATATGTTTTCTGCTTCTTTTGGGTCTTACTATATAACTACACGGA : 2380
NM_104490 : -------TGATTTGAGCTTCAACAAATTACATGGACCAATTCCGGCTTCACTTTTCAACTTAAGTCGACTTACTCACTTGT----------------------------------------------------------- :  952
FJ708661  : -------TGATTTGAGCTTCAACAAATTACATGGACCAATTCCGGCTTCACTTTTCAACTTAAGTCGACTTACTCACTTGT----------------------------------------------------------- :  958
                                                                                                                                                               
    
                     *      2400         *      2420         *      2440         *      2460         *      2480         *      2500         *      2520       
Genomic   : TCATTTTATAAAAAAAAATCGTTTGTCTTTTCATATTAGGTTTCTGGGAAACAACACGTTGAATGGATCCTTGCCCACTCTAAAGGGGCAGTCTCTGAGCAATTTGTGAGTAACGAAATCCTTTATAATTTTTCCATCTT : 2520
NM_104490 : -----------------------------------------TTCTGGGAAACAACACGTTGAATGGATCCTTGCCCACTCTAAAGGGGCAGTCTCTGAGCAATTT----------------------------------- : 1016
FJ708661  : -----------------------------------------TTCTGGGAAACAACACGTTGAATGGATCCTTGCCCACTCTAAAGGGGCAGTCTCTGAGCAATTTGTGAGTAACGAAATCCTTTATAATTTTTCCATCTT : 1057
                                                                                                                                                               
                     *      2540         *      2560         *      2580         *      2600         *      2620         *      2640         *      2660       
Genomic   : TAAAAGCCACAACGTTTCTTGGACGGTTTGTTGACAGATCCTCCTAATATGGCTCGTACACTATTTACGGGGCACTTATTCAAGTTGAATAAGGTTCCACTCCTGGTGACACAGATAACTTGCCAAGATATTAAGAAACT : 2660
NM_104490 : -------------------------------------------------------------------------------------------------------------------------------------------- :    -
FJ708661  : TAAAAGCCACAACGTTTCTTGGACGGTTTGTTGACAGATCCTCCTAATATGGCTCGTACACTATTTACGGGGCACTTATTCAAGTTGAATAAG----------------------------------------------- : 1150
                                                                                                                                                               
                     *      2680         *      2700         *      2720         *      2740         *      2760         *      2780         *      2800       
Genomic   : GTTTTGGACATATAATATAGTCGGTAGATCCTAACTGTATTTTTCTTTTATGCTGACAGAGATGTGTCGTACAATGATTTGTCTGGAAGTCTTCCTTCATGGGTCAGCTTACCAGATTTGAAGCTGTAAGTAAAGAAAAA : 2800
NM_104490 : -----------------------------------------------------------AGATGTGTCGTACAATGATTTGTCTGGAAGTCTTCCTTCATGGGTCAGCTTACCAGATTTGAAGCT--------------- : 1082
FJ708661  : -----------------------------------------------------------AGATGTGTCGTACAATGATTTGTCTGGAAGTCTTCCTTCATGGGTCAGCTTACCAGATTTGAAGCT--------------- : 1216
                                                                                                                                                               
                     *      2820         *      2840         *      2860         *      2880         *      2900         *      2920         *      2940       
Genomic   : CATTATATCTCAACTTATCTTCAGAGCCTTTTTAAAATTTCAGAATTTTTCCCACATGCATATTCATTAGGCCTCTTCATATGCAGCATGAGACTTCCTCTTTGTCGTAAAAGTTTATTATAATCACTTGTTAGGGAATA : 2940
NM_104490 : -------------------------------------------------------------------------------------------------------------------------------------------- :    -
FJ708661  : -------------------------------------------------------------------------------------------------------------------------------------------- :    -
                                                                                                                                                               
                     *      2960         *      2980         *      3000         *      3020         *      3040         *      3060         *      3080       
Genomic   : ATAAACAGAAGCGTTTGTTATATGTGCAGCAATCTAGTTGCTAACAACTTCACACTGGAAGGTCTTGACAACAGGTGAGTTTTTGTATATATTTCTATTCTTTTCCTACAAAAGGATGCAAGCATAAACGATTTATCATT : 3080
NM_104490 : -----------------------------CAATCTAGTTGCTAACAACTTCACACTGGAAGGTCTTGACAACAGG----------------------------------------------------------------- : 1128
FJ708661  : -----------------------------CAATCTAGTTGCTAACAACTTCACACTGGAAGGTCTTGACAACAGG----------------------------------------------------------------- : 1262
                                                                                                                                                               
                     *      3100         *      3120         *      3140         *      3160         *      3180         *      3200         *      3220       
Genomic   : TCTTTCTTAAATCTTTTTTTGTTGTTAAAAGGGTTTTATCAGGACTACATTGCCTGCAGAAGAACTTCCCCTGCAATCGAGGCGAAGGAATCTGTAAGTGTAACAAAAAGATACCCTCACAAATTATGTTCTCCATGTTG : 3220
NM_104490 : --------------------------------GTTTTATCAGGACTACATTGCCTGCAGAAGAACTTCCCCTGCAATCGAGGCGAAGGAATCTGTAAGTGTAACAAAAAGATACCCTCACAAATTATGTTCTCCATGTTG : 1236
FJ708661  : --------------------------------GTTTTATCAGGACTACATTGCCTGCAGAAGAACTTCCCCTGCAATCGAGGCGAAGGAATCT----------------------------------------------- : 1323
                                                                                                                                                               
                     *      3240         *      3260         *      3280         *      3300         *      3320         *      3340         *      3360       
Genomic   : AATGTGCAAACATATGGACAGGGCTCCAATGTCTTATTGGAAAAAAATTGTTTTAAGACTTTCGCTATTGACTGGTGTCTAGATTATAACTTTTCGATCAACTGCGGAGGCCCAGAGATAAGGTCTGTTAGTGGAGCACT : 3360
NM_104490 : AATGTGCAAACATATGGACAGGGCTCCAATGTCTTATTGGAAAAAAATTGTTTTAAGACTTTCGCTATTGACTGGTGTCTAGATTATAACTTTTCGATCAACTGCGGAGGCCCAGAGATAAGGTCTGTTAGTGGAGCACT : 1376
FJ708661  : ----------------------------------------------------------------------------------ATTATAACTTTTCGATCAACTGCGGAGGCCCAGAGATAAGGTCTGTTAGTGGAGCACT : 1381
                                                                                                                                                               
                     *      3380         *      3400         *      3420         *      3440         *      3460         *      3480         *      3500       
Genomic   : ATTTGAAAAGGAGGACGCGGATCTTGGACCAGCTTCGTTTGTTGTGAGTGCTGCAAAGAGATGGGCAGCCAGTAGTGTAGGAAATTTTGCTGGCAGTAGCAACAATATATACATAGCTACTTCACTAGCACAATTTATCA : 3500
NM_104490 : ATTTGAAAAGGAGGACGCGGATCTTGGACCAGCTTCGTTTGTTGTGAGTGCTGCAAAGAGATGGGCAGCCAGTAGTGTAGGAAATTTTGCTGGCAGTAGCAACAATATATACATAGCTACTTCACTAGCACAATTTATCA : 1516
FJ708661  : ATTTGAAAAGGAGGACGCGGATCTTGGACCAGCTTCGTTTGTTGTGAGTGCTGCAAAGAGATGGGCAGCCAGTAGTGTAGGAAATTTTGCTGGCAGTAGCAACAATATATACATAGCTACTTCACTAGCACAATTTATCA : 1521
                                                                                                                                                               
                     *      3520         *      3540         *      3560         *      3580         *      3600         *      3620         *      3640       
Genomic   : ACACTATGGACTCAGAGCTTTTTCAGTCAGCACGACTTTCTGCATCTTCCCTAAGGTATTATGGGTTGGGGCTAGAAAATGGAGGGTACACCGTAACACTTCAGTTTGCTGAAGTACAAATTGAAGGTTCTAACTCTTGG : 3640
NM_104490 : ACACTATGGACTCAGAGCTTTTTCAGTCAGCACGACTTTCTGCATCTTCCCTAAGGTATTATGGGTTGGGGCTAGAAAATGGAGGGTACACCGTAACACTTCAGTTTGCTGAAGTACAAATTGAAGGTTCTAACTCTTGG : 1656
FJ708661  : ACACTATGGACTCAGAGCTTTTTCAGTCAGCACGACTTTCTGCATCTTCCCTAAGGTATTATGGGTTGGGGCTAGAAAATGGAGGGTACACCGTAACACTTCAGTTTGCTGAAGTACAAATTGAAGGTTCTAACTCTTGG : 1661
                                                                                                                                                               
                     *      3660         *      3680         *      3700         *      3720         *      3740         *      3760         *      3780       
Genomic   : AAAGGTATTGGAAGACGACGTTTTAACATTTATGTCCAGGTCTGCACCACTTGATCGAGCTTTCCGGTTCACAAATGTTACTTTTTGGTTCTGATGAATTCTAAGATCTAGCTCCATTTTTCCAGGGAAGACTTGTTGAA : 3780
NM_104490 : AAAGGTATTGGAAGACGACGTTTTAACATTTATGTCCAGG--------------------------------------------------------------------------------------GAAGACTTGTTGAA : 1710
FJ708661  : AAAGGTATTGGAAGACGACGTTTTAACATTTATGTCCAGG--------------------------------------------------------------------------------------GAAGACTTGTTGAA : 1715
                                                                                                                                                               
                     *      3800         *      3820         *      3840         *      3860         *      3880         *      3900         *      3920       
Genomic   : AAGGATTTTGATATACGCAGAACAGCTGGTGGCTCTTCGGTTCGAGCAGTTCAGAGAGAATATAAAACAAATGTATCAGAAAATCATCTCGAAGTCCATCTTTTCTGGGCTGGAAAAGGAACATGTTGTATTCCTATCCA : 3920
NM_104490 : AAGGATTTTGATATACGCAGAACAGCTGGTGGCTCTTCGGTTCGAGCAGTTCAGAGAGAATATAAAACAAATGTATCAGAAAATCATCTCGAAGTCCATCTTTTCTGGGCTGGAAAAGGAACATGTTGTATTCCTATCCA : 1850
FJ708661  : AAGGATTTTGATATACGCAGAACAGCTGGTGGCTCTTCGGTTCGAGCAGTTCAGAGAGAATATAAAACAAATGTATCAGAAAATCATCTCGAAGTCCATCTTTTCTGGGCTGGAAAAGGAACATGTTGTATTCCTATCCA : 1855
                                                                                                                                                               
                     *      3940         *      3960         *      3980         *      4000         *      4020         *      4040         *      4060       
Genomic   : AGGGGCTTATGGGCCATTAATAGCGGCCGTCAGTGCGACACCAGGTAACACAAACGGTTGATATATTTTTTTTCCTCTGATTTTCACTATCTCCCGATACTAGTTAGTGCCGTGTCACATATTCTTGTTCAGATTTCACA : 4060
NM_104490 : AGGGGCTTATGGGCCATTAATAGCGGCCGTCAGTGCGACACCAG----------------------------------------------------------------------------------------ATTTCACA : 1902
FJ708661  : AGGGGCTTATGGGCCATTAATAGCGGCCGTCAGTGCGACACCAGGTAACACAAACGGTTGATATATTTTTTTTCCTCTGATTTTCACTATCTCCCGATACTAGTTAGTGCCGTGTCACATATTCTTGTTCAGATTTCACA : 1995
                                                                                                                                                               
                     *      4080         *      4100         *      4120         *      4140         *      4160         *      4180         *      4200       
Genomic   : CCAACTGTGGCTAATAGGCCACCATCAAAGGGAAAGAGCAGGACTGGTACTATTGTGGGTGTCATTGTTGGTGTTGGACTTTTGAGCATCTTTGCGGGTGTGGTTATCCTCGTCATTCGAAAAAGAAGAAAACCGTACAC : 4200
NM_104490 : CCAACTGTGGCTAATAGGCCACCATCAAAGGGAAAGAGCAGGACTGGTACTATTGTGGGTGTCATTGTTGGTGTTGGACTTTTGAGCATCTTTGCGGGTGTGGTTATCCTCGTCATTCGAAAAAGAAGAAAACCGTACAC : 2042
FJ708661  : CCAACTGTGGCTAATAGGCCACCATCAAAGGGAAAGAGCAGGACTGGTACTATTGTGGGTGTCATTGTTGGTGTTGGACTTTTGAGCATCTTTGCGGGTGTGGTTATCCTCGTCATTCGAAAAAGAAGAAAACCGTACAC : 2135
                                                                                                                                                               
                     *      4220         *      4240         *      4260         *      4280         *      4300         *      4320         *      4340       
Genomic   : AGATGATGAAGGTACAGATGTAATAAGAACATAAACAAGCCCTTGAGCTGAATAACTTTAGCTCTCTGATCAAAATTAAAGTTGACTATCATCTGTTAAATTGGTTGATTTTAAGTAGTGAATTTTTTATGGCAGAGATA : 4340
NM_104490 : AGATGATGAAG----------------------------------------------------------------------------------------------------------------------------AGATA : 2058
FJ708661  : AGATGATGAAG----------------------------------------------------------------------------------------------------------------------------AGATA : 2151
                                                                                                                                                               
                     *      4360         *      4380         *      4400         *      4420         *      4440         *      4460         *      4480       
Genomic   : CTTAGTATGGACGTAAAGCCTTACACCTTTACTTACTCGGAACTTAAGAATGCAACACAAGATTTTGATCTCTCAAACAAGCTTGGAGAGGGAGGGTTTGGGGCTGTTTATAAGGTAAGTCCCTTACTGAATCGAACAAA : 4480
NM_104490 : CTTAGTATGGACGTAAAGCCTTACACCTTTACTTACTCGGAACTTAAGAATGCAACACAAGATTTTGATCTCTCAAACAAGCTTGGAGAGGGAGGGTTTGGGGCTGTTTATAAGG------------------------- : 2173
FJ708661  : CTTAGTATGGACGTAAAGCCTTACACCTTTACTTACTCGGAACTTAAGAATGCAACACAAGATTTTGATCTCTCAAACAAGCTTGGAGAGGGAGGGTTTGGGGCTGTTTATAAGG------------------------- : 2266
                                                                                                                                                               
                     *      4500         *      4520         *      4540         *      4560         *      4580         *      4600         *      4620       
Genomic   : ATAGCTGTATATTTTTCTGAGATACTAGGAAAGAAGGGTACATGTAATACCTTTTAATACGTTTTGTTTTGAATAACACGTTCAACAGGGAAACCTCAATGATGGAAGAGAGGTGGCGGTGAAGCAGTTGTCAATTGGAT : 4620
NM_104490 : -----------------------------------------------------------------------------------------GAAACCTCAATGATGGAAGAGAGGTGGCGGTGAAGCAGTTGTCAATTGGAT : 2224
FJ708661  : -----------------------------------------------------------------------------------------GAAACCTCAATGATGGAAGAGAGGTGGCGGTGAAGCAGTTGTCAATTGGAT : 2317
                                                                                                                                                               
                     *      4640         *      4660         *      4680         *      4700         *      4720         *      4740         *      4760       
Genomic   : CACGGCAAGGGAAGGGACAATTTGTTGCAGAAATTATTGCAATTTCTTCAGTTCTACATCGCAACCTAGTAAAACTTTACGGGTGCTGCTTTGAAGGAGATCATCGTTTGCTCGTATATGAGTATCTCCCTAATGGAAGT : 4760
NM_104490 : CACGGCAAGGGAAGGGACAATTTGTTGCAGAAATTATTGCAATTTCTTCAGTTCTACATCGCAACCTAGTAAAACTTTACGGGTGCTGCTTTGAAGGAGATCATCGTTTGCTCGTATATGAGTATCTCCCTAATGGAAGT : 2364
FJ708661  : CACGGCAAGGGAAGGGACAATTTGTTGCAGAAATTATTGCAATTTCTTCAGTTCTACATCGCAACCTAGTAAAACTTTACGGGTGCTGCTTTGAAGGAGATCATCGTTTGCTCGTATATGAGTATCTCCCTAATGGAAGT : 2457


                                                                                                                                                               
                     *      4780         *      4800         *      4820         *      4840         *      4860         *      4880         *      4900       
Genomic   : CTCGATCAGGCATTATTTGGTAATGAACAGAAACATGACTTACCTATCTCTGCTTCTGCCAAGCTAAAGTAATCATTATGCCATTGGGTTGGATGTTACAGGGGATAAGAGTTTGCATCTTGATTGGTCAACCCGTTATG : 4900
NM_104490 : CTCGATCAGGCATTATTTGG----------------------------------------------------------------------------------GGATAAGAGTTTGCATCTTGATTGGTCAACCCGTTATG : 2422
FJ708661  : CTCGATCAGGCATTATTTGG----------------------------------------------------------------------------------GGATAAGAGTTTGCATCTTGATTGGTCAACCCGTTATG : 2515
                                                                                                                                                               
                     *      4920         *      4940         *      4960         *      4980         *      5000         *      5020         *      5040       
Genomic   : AGATATGTCTGGGAGTAGCCAGAGGTCTAGTTTATCTCCACGAGGAAGCAAGTGTTCGCATAATACACAGAGATGTGAAGGCCAGCAACATTTTACTGGACTCTGAACTGGTCCCAAAAGTTTCTGATTTTGGGCTTGCA : 5040
NM_104490 : AGATATGTCTGGGAGTAGCCAGAGGTCTAGTTTATCTCCACGAGGAAGCAAGTGTTCGCATAATACACAGAGATGTGAAGGCCAGCAACATTTTACTGGACTCTGAACTGGTCCCAAAAGTTTCTGATTTTGGGCTTGCA : 2562
FJ708661  : AGATATGTCTGGGAGTAGCCAGAGGTCTAGTTTATCTCCACGAGGAAGCAAGTGTTCGCATAATACACAGAGATGTGAAGGCCAGCAACATTTTACTGGACTCTGAACTGGTCCCAAAAGTTTCTGATTTTGGGCTTGCA : 2655
                                                                                                                                                               
                     *      5060         *      5080         *      5100         *      5120         *      5140         *      5160         *      5180       
Genomic   : AAACTATATGATGACAAGAAAACCCATATTAGTACCAGAGTGGCAGGGACGATGTAAGCAAAATCCATAGACCCTGACATTTTGTTTGCAACACAATTATGGCTTTATAGCTCATGTTAGTTGTATCAATGCAGTGGGTA : 5180
NM_104490 : AAACTATATGATGACAAGAAAACCCATATTAGTACCAGAGTGGCAGGGACGAT---------------------------------------------------------------------------------TGGGTA : 2621
FJ708661  : AAACTATATGATGACAAGAAAACCCATATTAGTACCAGAGTGGCAGGGACGAT---------------------------------------------------------------------------------TGGGTA : 2714
                                                                                                                                                               
                     *      5200         *      5220         *      5240         *      5260         *      5280         *      5300         *      5320       
Genomic   : TCTTGCGCCAGAGTATGCCATGCGTGGACATCTAACAGAGAAAACAGATGTGTATGCCTTTGGTGTTGTGGCTCTTGAGCTAGTGAGTGGAAGGAAAAACTCTGATGAGAACCTGGAAGAGGGAAAAAAATATCTTCTTG : 5320
NM_104490 : TCTTGCGCCAGAGTATGCCATGCGTGGACATCTAACAGAGAAAACAGATGTGTATGCCTTTGGTGTTGTGGCTCTTGAGCTAGTGAGTGGAAGGAAAAACTCTGATGAGAACCTGGAAGAGGGAAAAAAATATCTTCTTG : 2761
FJ708661  : TCTTGCGCCAGAGTATGCCATGCGTGGACATCTAACAGAGAAAACAGATGTGTATGCCTTTGGTGTTGTGGCTCTTGAGCTAGTGAGTGGAAGGAAAAACTCTGATGAGAACCTGGAAGAGGGAAAAAAATATCTTCTTG : 2854
                                                                                                                                                               
                     *      5340         *      5360         *      5380         *      5400         *      5420         *      5440         *      5460       
Genomic   : AATGGGTACGTCTATATTTGTCATATAGGCGAAGTTGAAGATGGTGTGATAAATGACAAAAGCCGATAATCCTTTTGTATTTCTTGTATACAGGCATGGAATCTACACGAGAAAAACCGTGACGTTGAACTAATAGATGA : 5460
NM_104490 : AATGGG----------------------------------------------------------------------------------------CATGGAATCTACACGAGAAAAACCGTGACGTTGAACTAATAGATGA : 2813
FJ708661  : AATGGG----------------------------------------------------------------------------------------CATGGAATCTACACGAGAAAAACCGTGACGTTGAACTAATAGATGA : 2906
                                                                                                                                                               
                     *      5480         *      5500         *      5520         *      5540         *      5560         *      5580         *      5600       
Genomic   : TGAGCTAAGTGAATACAACATGGAAGAAGTGAAACGCATGATTGGCATTGCTCTGCTTTGCACTCAGTCATCTTATGCGTTGAGACCACCAATGTCACGAGTAGTAGCCATGTTGTCAGGAGATGCTGAGGTCAATGATG : 5600
NM_104490 : TGAGCTAAGTGAATACAACATGGAAGAAGTGAAACGCATGATTGGCATTGCTCTGCTTTGCACTCAGTCATCTTATGCGTTGAGACCACCAATGTCACGAGTAGTAGCCATGTTGTCAGGAGATGCTGAGGTCAATGATG : 2953
FJ708661  : TGAGCTAAGTGAATACAACATGGAAGAAGTGAAACGCATGATTGGCATTGCTCTGCTTTGCACTCAGTCATCTTATGCGTTGAGACCACCAATGTCACGAGTAGTAGCCATGTTGTCAGGAGATGCTGAGGTCAATGATG : 3046
                                                                                                                                                               
                     *      5620         *      5640         *      5660         *      5680         *      5700         *      5720         *      5740       
Genomic   : CCACCTCTAAGCCAGGCTACCTAACCGACTGTACATTTGATGACACCACAAGCTCCTCATTCAGCAACTTTCAAACGAAAGACACTAGCTTCTCGACGAGCTTTATAGCGCCTGGCCCGGAGATGCCACTTAGAGACGGC : 5740
NM_104490 : CCACCTCTAAGCCAGGCTACCTAACCGACTGTACATTTGATGACACCACAAGCTCCTCATTCAGCAACTTTCAAACGAAAGACACTAGCTTCTCGACGAGCTTTATAGCGCCTGGCCCGGAGATGCCACTTAGAGACGGC : 3093
FJ708661  : CCACCTCTAAGCCAGGCTACCTAACCGACTGTACATTTGATGACACCACAAGCTCCTCATTCAGCAACTTTCAAACGAAAGACACTAGCTTCTCGACGAGCTTTATAGCGCCTGGCCCGGAGATGCCACTTAGAGACGGC : 3186
                                                             
                     *      5760         *      5780         
Genomic   : GAATCGAAGCCAATGGTTGGATTCAAGATCAAGGAGGGAAGA : 5782
NM_104490 : GAATCGAAGCCAATGGTTGGATTCAAGATCAAGGAGGGAAGA : 3135
FJ708661  : GAATCGAAGCCAATGGTTGGATTCAAGATCAAGGAGGGAAGA : 3228


At1g56130
                                                                                                                                                               
                     *        20         *        40         *        60         *        80         *       100         *       120         *       140       
Genomic   : ATGACCAGGATACGGAGGTCTCCGTGTCTACTACTCCTCATTATCTGGTTCATGTGTATTGCCGGTTCGGTTCAGGTGGTTCAATCTCAAAACCAAACCGGAGCCACTACTCATCCCGACGAAGGTTTGTCTTTAATCTA :  140
NM_104491 : ATGACCAGGATACGGAGGTCTCCGTGTCTACTACTCCTCATTATCTGGTTCATGTGTATTGCCGGTTCGGTTCAGGTGGTTCAATCTCAAAACCAAACCGGAGCCACTACTCATCCCGACGAAG---------------- :  124
FJ708662  : ATGACCAGGATACGGAGGTCTCCGTGTCTACTACTCCTCATTATCTGGTTCATGTGTATTGCCGGTTCGGTTCAGGTGGTTCAATCTCAAAACCAAACCGGAGCCACTACTCATCCCGACGAAG---------------- :  124
                                                                                                                                                               
                     *       160         *       180         *       200         *       220         *       240         *       260         *       280       
Genomic   : ACACTTACAAAGAGTTCAATCACCAATAATCAATTGGATTGTATAGATTATAGTCTTATACTAGTTATTCGGATCCAAATTGATTAGAAACAAAAAATACACCATGAATTGTATCCACAAATTCCAAAATAATAAGATAC :  280
NM_104491 : -------------------------------------------------------------------------------------------------------------------------------------------- :    -
FJ708662  : -------------------------------------------------------------------------------------------------------------------------------------------- :    -
                                                                                                                                                               
                     *       300         *       320         *       340         *       360         *       380         *       400         *       420       
Genomic   : TTACCGGTTAGTTTCTGTTAATAATTGACCGGTTATATTAATTTGACCGTTGTGATTGATTGTGGATACAACAGCGCGAGCTTTGAACTCAATTTTCGCGGCTTGGAAGATCCAGGCGCCGAGAGAATGGAACATCAGCG :  420
NM_104491 : --------------------------------------------------------------------------CGCGAGCTTTGAACTCAATTTTCGCGGCTTGGAAGATCCAGGCGCCGAGAGAATGGAACATCAGCG :  190
FJ708662  : --------------------------------------------------------------------------CGCGAGCTTTGAACTCAATTTTCGCGGCTTGGAAGATCCAGGCGCCGAGAGAATGGAACATCAGCG :  190
                                                                                                                                                               
                     *       440         *       460         *       480         *       500         *       520         *       540         *       560       
Genomic   : GCGAACTTTGCTCCGGCGCCGCTATCGACGCCAGTGTACTCGACAGCAACCCTGCCTACAATCCTCTCATCAAATGCGACTGTAGTTTCCAAAACTCCACAATCTGCCGCATCACTAACATGTACGCCCTTTTTGTGCAC :  560
NM_104491 : GCGAACTTTGCTCCGGCGCCGCTATCGACGCCAGTGTACTCGACAGCAACCCTGCCTACAATCCTCTCATCAAATGCGACTGTAGTTTCCAAAACTCCACAATCTGCCGCATCACTAACAT------------------- :  311
FJ708662  : GCGAACTTTGCTCCGGCGCCGCTATCGACGCCAGTGTACTCGACAGCAACCCTGCCTACAATCCTCTCATCAAATGCGACTGTAGTTTCCAAAACTCCACAATCTGCCGCATCACTAACAT------------------- :  311
                                                                                                                                                               
                     *       580         *       600         *       620         *       640         *       660         *       680         *       700       
Genomic   : CCCATGTGTTCGACGTATTCCCCCACTGGGACTTGACTTTTCTTTATACAAATCATTTTTAATTTCATGTGGTAACTATGCTTCAGCTATGTCACGTCTCAAATTCTTCTCCTTGTTGTTGCCACAGCAAGGTTTATGCG :  700
NM_104491 : -------------------------------------------------------------------------------------------------------------------------------CAAGGTTTATGCG :  324
FJ708662  : -------------------------------------------------------------------------------------------------------------------------------CAAGGTTTATGCG :  324                                                                                                                                                               
                                                                                                                                                               
                     *       720         *       740         *       760         *       780         *       800         *       820         *       840       
Genomic   : ATAGATGTTGTAGGACCTATCCCTCCAGAGCTCTGGACTTTAACATACCTCACCAATCTGTATGGTTTTTACTATTACTTGATGATTATATAGATTAAGCTACAAGAAAAAGAGCACAAATTTTCACAAATCAAAATCAC :  840
NM_104491 : ATAGATGTTGTAGGACCTATCCCTCCAGAGCTCTGGACTTTAACATACCTCACCAATCTG-------------------------------------------------------------------------------- :  384
FJ708662  : ATAGATGTTGTAGGACCTATCCCTCCAGAGCTCTGGACTTTAACATACCTCACCAATCTG-------------------------------------------------------------------------------- :  384
                                                                                                                                                               
                     *       860         *       880         *       900         *       920         *       940         *       960         *       980       
Genomic   : TTCTTTCAGGAACTTGGGTCAAAATGTTCTCACTGGCTCACTTCCTCCTGCAATTGGAAATCTGACTCGAATGCAATGGATGTATGTCTCTGACAACTTCTTTGTCTTATTGTTGTCGTGATGATTTTCTACAACAATTT :  980
NM_104491 : ----------AACTTGGGTCAAAATGTTCTCACTGGCTCACTTCCTCCTGCAATTGGAAATCTGACTCGAATGCAATGGATG---------------------------------------------------------- :  456
FJ708662  : ----------AACTTGGGTCAAAATGTTCTCACTGGCTCACTTCCTCCTGCAATTGGAAATCTGACTCGAATGCAATGGATG---------------------------------------------------------- :  456
                                                                                                                                                               
                     *      1000         *      1020         *      1040         *      1060         *      1080         *      1100         *      1120       
Genomic   : CTTGTCTCTCACGCTTAACTGTTTTTAATATGTAGGACTTTTGGGATCAATGCGTTGTCTGGCCCTGTTCCTAAGGAAATTGGTTTGCTTACAGATTTAAGATTACTGTAAGCTTATGGATGCTTCTTCATTGTTTTAAT : 1120
NM_104491 : ------------------------------------ACTTTTGGGATCAATGCGTTGTCTGGCCCTGTTCCTAAGGAAATTGGTTTGCTTACAGATTTAAGATTACT--------------------------------- :  527
FJ708662  : ------------------------------------ACTTTTGGGATCAATGCGTTGTCTGGCCCTGTTCCTAAGGAAATTGGTTTGCTTACAGATTTAAGATTACT--------------------------------- :  527
                                                                                                                                                               
                     *      1140         *      1160         *      1180         *      1200         *      1220         *      1240         *      1260       
Genomic   : ATAATTGTCTCTTCACAATGTTTCTGAAAGATCTTGACGGTTCCTTATACATATGTATATCTTTTTGCAGTGGTATTAGTTCAAATAACTTTTCCGGTTCTATACCAGATGAGATTGGGAGATGTACAAAACTACAACAG : 1260
NM_104491 : ----------------------------------------------------------------------TGGTATTAGTTCAAATAACTTTTCCGGTTCTATACCAGATGAGATTGGGAGATGTACAAAACTACAACAG :  597
FJ708662  : ----------------------------------------------------------------------TGGTATTAGTTCAAATAACTTTTCCGGTTCTATACCAGATGAGATTGGGAGATGTACAAAACTACAACAG :  597
                                                                                                                                                               
                     *      1280         *      1300         *      1320         *      1340         *      1360         *      1380         *      1400       
Genomic   : ATGTGAGTCTATCTGTTAAATCGGCAAGCGTTTTATGTATTTATTTCCTGGTTATAACTTAGATAGGAAATCTTAACCTCAGTAATCATCTAGTGTTTACCGACCACAACATTCTTGTGTAATTACCTTTTTAAAGAATT : 1400
NM_104491 : ATGT---------------------------------------------------------------------------------------------------------------------------------------- :  601
FJ708662  : ATGT---------------------------------------------------------------------------------------------------------------------------------------- :  601
                                                                                                                                                               
                     *      1420         *      1440         *      1460         *      1480         *      1500         *      1520         *      1540       
Genomic   : TCTTCCATCTTTTGAAGGTACATAGATAGTTCTGGACTCAGCGGGAGAATACCTTTATCATTTGCTAACCTCGTGCAGCTGGAACAAGCGTAAGATATACTGAATTCGAGGATAAGCTTTATTGTTGGGTTTGTACCATC : 1540
NM_104491 : -------------------ACATAGATAGTTCTGGACTCAGCGGGAGAATACCTTTATCATTTGCTAACCTCGTGCAGCTGGAACAAGC--------------------------------------------------- :  671
FJ708662  : -------------------ACATAGATAGTTCTGGACTCAGCGGGAGAATACCTTTATCATTTGCTAACCTCGTGCAGCTGGAACAAGC--------------------------------------------------- :  671
                                                                                                                                                               
                     *      1560         *      1580         *      1600         *      1620         *      1640         *      1660         *      1680       
Genomic   : TATTTATTACTTTACGTTTTAATCTCAATGTTGTACCAGTTGGATCGCGGATCTGGAAGTTACAGATCAGATACCAGACTTTATAGGAGATTGGACCAAACTTACTACCTTGTAAGAAGATTTTCATTTTTACGGGACTT : 1680
NM_104491 : ---------------------------------------TTGGATCGCGGATCTGGAAGTTACAGATCAGATACCAGACTTTATAGGAGATTGGACCAAACTTACTACCTTG---------------------------- :  744
FJ708662  : ---------------------------------------TTGGATCGCGGATCTGGAAGTTACAGATCAGATACCAGACTTTATAGGAGATTGGACCAAACTTACTACCTTG---------------------------- :  744
                                                                                                                                                               
                     *      1700         *      1720         *      1740         *      1760         *      1780         *      1800         *      1820       
Genomic   : ATATTGGATTTGTTACATTCATCTTGTTTTAATATAGTCTGTATTAATTTTCCTCTTAGTAGAACACTCTTCTTTATGTTTTAATTTGGGATTCAACGATTGGTACATGTTTTACTGCAGGAGAATTATTGGAACTGGTC : 1820
NM_104491 : -------------------------------------------------------------------------------------------------------------------------AGAATTATTGGAACTGGTC :  763
FJ708662  : -------------------------------------------------------------------------------------------------------------------------AGAATTATTGGAACTGGTC :  763
                                                                                                                                                               
                     *      1840         *      1860         *      1880         *      1900         *      1920         *      1940         *      1960       
Genomic   : TGAGTGGTCCGATACCTTCGTCATTTTCCAACTTGACTTCGTTGACAGAATTGTATGTGTTGCATTGGTATCAGAATCATGATTATTTACTGTTACTTTCTCTTTCCCTTTCCTCACATATTCATATTCAATATGTAGGA : 1960
NM_104491 : TGAGTGGTCCGATACCTTCGTCATTTTCCAACTTGACTTCGTTGACAGAATTG--------------------------------------------------------------------------------------A :  817
FJ708662  : TGAGTGGTCCGATACCTTCGTCATTTTCCAACTTGACTTCGTTGACAGAATTG--------------------------------------------------------------------------------------A :  817
                                                                                                                                                               
                     *      1980         *      2000         *      2020         *      2040         *      2060         *      2080         *      2100       
Genomic   : GGCTTGGTGATATATCCAGTGGAAGCTCTTCTCTTGATTTCATCAAAGACATGAAATCTCTTAGTGTATTGTAAGGCAGCTTTGATTCAACTTCCGCTCCAATTTATGATCAGATTTTTGTAGTGTTGAACTGTTTCTCT : 2100
NM_104491 : GGCTTGGTGATATATCCAGTGGAAGCTCTTCTCTTGATTTCATCAAAGACATGAAATCTCTTAGTGTATT---------------------------------------------------------------------- :  887
FJ708662  : GGCTTGGTGATATATCCAGTGGAAGCTCTTCTCTTGATTTCATCAAAGACATGAAATCTCTTAGTGTATT---------------------------------------------------------------------- :  887
                                                                                                                                                               
                     *      2120         *      2140         *      2160         *      2180         *      2200         *      2220         *      2240       
Genomic   : CTCCTCCGTGTATTTAACATTTATGATATATGTCGCAGAGTTTTGAGGAACAACAATCTCACTGGGACAATACCGTCTACTATTGGAGAACACTCAAGTTTGCGACAAGTGTAAGTACATATGTCAATCTGTTTCTTCAA : 2240
NM_104491 : --------------------------------------AGTTTTGAGGAACAACAATCTCACTGGGACAATACCGTCTACTATTGGAGAACACTCAAGTTTGCGACAAGT------------------------------ :  959
FJ708662  : --------------------------------------AGTTTTGAGGAACAACAATCTCACTGGGACAATACCGTCTACTATTGGAGAACACTCAAGTTTGCGACAAGT------------------------------ :  959

                                                                                                                                                               
                     *      2260         *      2280         *      2300         *      2320         *      2340         *      2360         *      2380       
Genomic   : ATGCTCTGAAAGCTGTTTCAGGGTCTTTCCCTGTAAAAAATGTGAGATCATCACTGTCAAAAGTCACCGTGAGAATACGAAATCATCCCATATTGATGATTTGTAAAAGTCCCCGAGAAAATACAAAACAATCTTAAATT : 2380
NM_104491 : -------------------------------------------------------------------------------------------------------------------------------------------- :    -
FJ708662  : -------------------------------------------------------------------------------------------------------------------------------------------- :    -
                                                                                                                                                               
                     *      2400         *      2420         *      2440         *      2460         *      2480         *      2500         *      2520       
Genomic   : GTTGTTAATTGCTGATCGTCTAAACAGGAATGGTTTAATGTTATAACATGTAATGCATGTCTGTTAGCAAACAAGTGTAAGTAACTCGTGTCTGGTTTCCAGTGATTTAAGCTTCAACAAACTACATGGACCAATTCCGG : 2520
NM_104491 : ------------------------------------------------------------------------------------------------------TGATTTAAGCTTCAACAAACTACATGGACCAATTCCGG :  997
FJ708662  : ------------------------------------------------------------------------------------------------------TGATTTAAGCTTCAACAAACTACATGGACCAATTCCGG :  997
                                                                                                                                                               
                     *      2540         *      2560         *      2580         *      2600         *      2620         *      2640         *      2660       
Genomic   : CTTCACTTTTCAACTTAAGTCAGCTTACTCACTTGTAAGATGATGTGTTTTCTGCTTCTCTTGGGTCTTAATAAGTTAATACACAACTACATGGGTAGTATTTTTATAAAAATTGTTGTCTTTTCATATTAGGTTTCTGG : 2660
NM_104491 : CTTCACTTTTCAACTTAAGTCAGCTTACTCACTTGT--------------------------------------------------------------------------------------------------TTCTGG : 1039
FJ708662  : CTTCACTTTTCAACTTAAGTCAGCTTACTCACTTGT--------------------------------------------------------------------------------------------------TTCTGG : 1039
                                                                                                                                                               
                     *      2680         *      2700         *      2720         *      2740         *      2760         *      2780         *      2800       
Genomic   : GAAACAACACGTTGAATGGCTCCTTTCCCACTCAAAAGACGCAGTCTCTGAGAAATGTGTGAGTAACAAAATTTTTTATAATTGTTTCATCCTGATGTAGACTCAAGTTTCATACTGACGTTGAAATAGGTTCCACTCTT : 2800
NM_104491 : GAAACAACACGTTGAATGGCTCCTTTCCCACTCAAAAGACGCAGTCTCTGAGAAATGT---------------------------------------------------------------------------------- : 1097
FJ708662  : GAAACAACACGTTGAATGGCTCCTTTCCCACTCAAAAGACGCAGTCTCTGAGAAATGT---------------------------------------------------------------------------------- : 1097
                                                                                                                                                               
                     *      2820         *      2840         *      2860         *      2880         *      2900         *      2920         *      2940       
Genomic   : GGTTACGCGGATAACTTCGCCAGATAATCAGGAAACAATTTTGGGACATATTACATATTATGTGTATTCCTAATTATTGTTCTTTTATGCTCACAGAGATGTGTCGTACAATGATTTGTCTGGAAGTCTTCCTTCATGGG : 2940
NM_104491 : ------------------------------------------------------------------------------------------------AGATGTGTCGTACAATGATTTGTCTGGAAGTCTTCCTTCATGGG : 1141
FJ708662  : ------------------------------------------------------------------------------------------------AGATGTGTCGTACAATGATTTGTCTGGAAGTCTTCCTTCATGGG : 1141

                     *      2960         *      2980         *      3000         *      3020         *      3040         *      3060         *      3080       
Genomic   : TCAGCTTACCAAGCTTGAAACTGTAAGTAAACAAAACACCATATCCAACCTATATTCACAGAAAATTTCTCATATGTATATTCACTAGGCTTCTTCATATCCAGCATGATAGTTATTATAATCTGTATTAAACAAAACTG : 3080
NM_104491 : TCAGCTTACCAAGCTTGAAACT---------------------------------------------------------------------------------------------------------------------- : 1163
FJ708662  : TCAGCTTACCAAGCTTGAAACT---------------------------------------------------------------------------------------------------------------------- : 1163

                     *      3100         *      3120         *      3140         *      3160         *      3180         *      3200         *      3220       
Genomic   : TATGATATTTCTGCAGCAACCTAGTTGCTAACAACTTCACATTAGAAGGTCTTGACAACAGGTGATTATCTTAAATATGTTCTTGTTGGTGATTGAAACGCCTATATGTTTCTCTTTTCTTTCTTTTTATTTTCCTCCAA : 3220
NM_104491 : ----------------CAACCTAGTTGCTAACAACTTCACATTAGAAGGTCTTGACAACAGG------------------------------------------------------------------------------ : 1209
FJ708662  : ----------------CAACCTAGTTGCTAACAACTTCACATTAGAAGGTCTTGACAACAGG------------------------------------------------------------------------------ : 1209

                     *      3240         *      3260         *      3280         *      3300         *      3320         *      3340         *      3360       
Genomic   : AAGGATGCAAGATTTAATGAATCTTTCTTTAATCATTTTTCTTTTGTTAAAAGGGTTTTACCAGGACTGAACTGCCTGCAGAAAAACTTCCCCTGCAATCGAGGCAAAGGAATCTGTAAGTGTAACAATGAGATATCCTC : 3360
NM_104491 : ------------------------------------------------------GTTTTACCAGGACTGAACTGCCTGCAGAAAAACTTCCCCTGCAATCGAGGCAAAGGAATCT------------------------- : 1270
FJ708662  : ------------------------------------------------------GTTTTACCAGGACTGAACTGCCTGCAGAAAAACTTCCCCTGCAATCGAGGCAAAGGAATCT------------------------- : 1270
                                                                                                                                                               
                     *      3380         *      3400         *      3420         *      3440         *      3460         *      3480         *      3500       
Genomic   : ATAACTGTTATTCTCCATGTGTACTGAGCAAACTCATGGGTAGTTGGAAATGTTTTTGTTTAATTTCGCTATTGATTGGTGTCTAGATTCTGACTTTTCAATCAACTGCGGAGGCCCAGAGAAAAGATCTGTAACTGGAG : 3500
NM_104491 : --------------------------------------------------------------------------------------ATTCTGACTTTTCAATCAACTGCGGAGGCCCAGAGAAAAGATCTGTAACTGGAG : 1324
FJ708662  : --------------------------------------------------------------------------------------ATTCTGACTTTTCAATCAACTGCGGAGGCCCAGAGAAAAGATCTGTAACTGGAG : 1324
                                                                                                                                                               
                     *      3520         *      3540         *      3560         *      3580         *      3600         *      3620         *      3640       
Genomic   : CACTATTTGAGAGGGAGGATGAGGATTTTGGACCAGCTTCATTTTTCGTGAGTGCTGGTCAGAGATGGGCAGCTAGTAGTGTAGGACTTTTTGCCGGAAGTAGCAACAATATATACATAGCTACTTCACAATCACAATTT : 3640
NM_104491 : CACTATTTGAGAGGGAGGATGAGGATTTTGGACCAGCTTCATTTTTCGTGAGTGCTGGTCAGAGATGGGCAGCTAGTAGTGTAGGACTTTTTGCCGGAAGTAGCAACAATATATACATAGCTACTTCACAATCACAATTT : 1464
FJ708662  : CACTATTTGAGAGGGAGGATGAGGATTTTGGACCAGCTTCATTTTTCGTGAGTGCTGGTCAGAGATGGGCAGCTAGTAGTGTAGGACTTTTTGCCGGAAGTAGCAACAATATATACATAGCTACTTCACAATCACAATTT : 1464
                                                                                                                                                               
                     *      3660         *      3680         *      3700         *      3720         *      3740         *      3760         *      3780       
Genomic   : GTCAACACTTTGGACTCAGAGCTATTTCAATCAGCAAGACTTTCTGCATCTTCCGTAAGGTATTATGGGTTGGGGCTAGAAAATGGAGGATATACAGTAACCCTTCAGTTTGCTGAAATACAAATTCTAGGTTCTACTTC : 3780
NM_104491 : GTCAACACTTTGGACTCAGAGCTATTTCAATCAGCAAGACTTTCTGCATCTTCCGTAAGGTATTATGGGTTGGGGCTAGAAAATGGAGGATATACAGTAACCCTTCAGTTTGCTGAAATACAAATTCTAGGTTCTACTTC : 1604
FJ708662  : GTCAACACTTTGGACTCAGAGCTATTTCAATCAGCAAGACTTTCTGCATCTTCCGTAAGGT-----------------------------------------------------------------------TCTACTTC : 1533
                                                                                                                                                               
                     *      3800         *      3820         *      3840         *      3860         *      3880         *      3900         *      3920       
Genomic   : TACCACTTGGAAAGGTTTGGGACGACGACGTTTTGACATTTATGTCCAGGTCTGCACTACTTGATCAAGCTTTCCGCTTTACAAGTTAACTATTTGGTGTTATCCATTTCCAAATTTGGTGATGGATTCTGACATGTAGC : 3920
NM_104491 : TACCACTTGGAAAGGTTTGGGACGACGACGTTTTGACATTTATGTCCAGG------------------------------------------------------------------------------------------ : 1654
FJ708662  : TACCACTTGGAAAGGTTTGGGACGACGACGTTTTGACATTTATGTCCAGG------------------------------------------------------------------------------------------ : 1583
                                                                                                                                                               
                     *      3940         *      3960         *      3980         *      4000         *      4020         *      4040         *      4060       
Genomic   : TCCGTTTTTCCAGGGAAGACTTGTTGAAAAGGATTTTGATGTACGTAGAACAGCTGGTGACTCTACTGTTAGAGCAGTTCAGAGAGTATATAAGGCAAATGTATCAGAAAATCACCTCGAAGTTCATCTTTTCTGGGCTG : 4060
NM_104491 : --------------GAAGACTTGTTGAAAAGGATTTTGATGTACGTAGAACAGCTGGTGACTCTACTGTTAGAGCAGTTCAGAGAGTATATAAGGCAAATGTATCAGAAAATCACCTCGAAGTTCATCTTTTCTGGGCTG : 1780
FJ708662  : --------------GAAGACTTGTTGAAAAGGATTTTGATGTACGTAGAACAGCTGGTGACTCTACTGTTAGAGCAGTTCAGAGAGTATATAAGGCAAATGTATCAGAAAATCACCTCGAAGTTCATCTTTTCTGGGCTG : 1709
                                                                                                                                                               
                     *      4080         *      4100         *      4120         *      4140         *      4160         *      4180         *      4200       
Genomic   : GGAAAGGAACATGTTGTATACCTATCCAAGGGGCTTATGGACCATTAATATCAGCAGTCAGTGCAACACCAGGTAACACAAATGGCGAATTCTTTCTTCTCCCTAATTTTCACTATCTTTTGATGCTACTTAGTGCCTAA : 4200
NM_104491 : GGAAAGGAACATGTTGTATACCTATCCAAGGGGCTTATGGACCATTAATATCAGCAGTCAGTGCAACACCAG-------------------------------------------------------------------- : 1852
FJ708662  : GGAAAGGAACATGTTGTATACCTATCCAAGGGGCTTATGGACCATTAATATCAGCAGTCAGTGCAACACCAG-------------------------------------------------------------------- : 1781
                                                                                                                                                               
                     *      4220         *      4240         *      4260         *      4280         *      4300         *      4320         *      4340       
Genomic   : GAGATGGTGTAACATATTCATGTTCAGATTTCACACCAACTGTGGCGAATAAGCCACCATCAAAAGGAAAGAACAGGACTGGTACTATTGTGGGCGTCATTGTTGGCGTAGGACTTTTGAGCATCCTTGCGGGTGTGGTC : 4340
NM_104491 : ---------------------------ATTTCACACCAACTGTGGCGAATAAGCCACCATCAAAAGGAAAGAACAGGACTGGTACTATTGTGGGCGTCATTGTTGGCGTAGGACTTTTGAGCATCCTTGCGGGTGTGGTC : 1965
FJ708662  : ---------------------------ATTTCACACCAACTGTGGCGAATAAGCCACCATCAAAAGGAAAGAACAGGACTGGTACTATTGTGGGCGTCATTGTTGGCGTAGGACTTTTGAGCATCCTTGCGGGTGTGGTC : 1894
                                                                                                                                                               
                     *      4360         *      4380         *      4400         *      4420         *      4440         *      4460         *      4480       
Genomic   : ATGTTTACTATCCGAAAAAGAAGAAAACGGTACACAGATGATGAAGGTATGTAATCAGAATACAAACAAGCCCTTGAGCTGAAGAATTTTGGCCCTAAAATTAGACCTGACTATCATTTTTTAAATTGGTTAATTTATAA : 4480
NM_104491 : ATGTTTACTATCCGAAAAAGAAGAAAACGGTACACAGATGATGAAG---------------------------------------------------------------------------------------------- : 2011
FJ708662  : ATGTTTACTATCCGAAAAAGAAGAAAACGGTACACAGATGATGAAG---------------------------------------------------------------------------------------------- : 1940
                                                                                                                                                               
                     *      4500         *      4520         *      4540         *      4560         *      4580         *      4600         *      4620       
Genomic   : CCGAATCGTTTTTTGCAGAGCTACTTGGTATGGACGTAAAGCCTTACATCTTTACTTATTCGGAACTTAAAAGTGCAACTCAAGATTTTGATCCCTCAAACAAGCTTGGAGAGGGGGGATTTGGGCCTGTTTATAAGGTA : 4620
NM_104491 : ------------------AGCTACTTGGTATGGACGTAAAGCCTTACATCTTTACTTATTCGGAACTTAAAAGTGCAACTCAAGATTTTGATCCCTCAAACAAGCTTGGAGAGGGGGGATTTGGGCCTGTTTATAAGG-- : 2131
FJ708662  : ------------------AGCTACTTGGTATGGACGTAAAGCCTTACATCTTTACTTATTCGGAACTTAAAAGTGCAACTCAAGATTTTGATCCCTCAAACAAGCTTGGAGAGGGGGGATTTGGGCCTGTTTATAAGG-- : 2060
                                                                                                                                                               
                     *      4640         *      4660         *      4680         *      4700         *      4720         *      4740         *      4760       
Genomic   : AGGTACCCATACCGAAATGGACGACATATCTGATATCTGTATATTCTTAGATATACTAGGAAAGAAGAGTGTCTGTTTATACGTTTTGTTTTGAAAAACACATTCAATAGGGAAACCTTAATGATGGAAGAGTGGTCGCG : 4760
NM_104491 : ---------------------------------------------------------------------------------------------------------------GAAACCTTAATGATGGAAGAGTGGTCGCG : 2160
FJ708662  : ---------------------------------------------------------------------------------------------------------------GAAACCTTAATGATGGAAGAGTGGTCGCG : 2089
  

                                                                                                                                                             
                                                                                                                                                               
                     *      4780         *      4800         *      4820         *      4840         *      4860         *      4880         *      4900       
Genomic   : GTGAAGCTATTGTCGGTGGGATCCCGTCAAGGGAAGGGACAATTTGTTGCAGAAATTGTAGCAATTTCTTCAGTTCTACATCGCAACCTAGTAAAACTTTATGGGTGCTGCTTTGAAGGAGAGCATCGTATGCTCGTATA : 4900
NM_104491 : GTGAAGCTATTGTCGGTGGGATCCCGTCAAGGGAAGGGACAATTTGTTGCAGAAATTGTAGCAATTTCTTCAGTTCTACATCGCAACCTAGTAAAACTTTATGGGTGCTGCTTTGAAGGAGAGCATCGTATGCTCGTATA : 2300
FJ708662  : GTGAAGCTATTGTCGGTGGGATCCCGTCAAGGGAAGGGACAATTTGTTGCAGAAATTGTAGCAATTTCTTCAGTTCTACATCGCGACCTAGTAAAACTTTATGGGTGCTGCTTTGAAGGAGAGCATCGTATGCTCGTATA : 2229
                                                                                                                                                               
                     *      4920         *      4940         *      4960         *      4980         *      5000         *      5020         *      5040       
Genomic   : TGAGTATCTACCTAATGGAAGTCTTGATCAGGCGCTATTTGGTAATGAACAGAAACATGACTTATCTATCACTGCTTCTGCGAACCTTAAGTACTCATTTATGCCCTTGGTTTGCATGTTTCAGGGGATAAGACCTTACA : 5040
NM_104491 : TGAGTATCTACCTAATGGAAGTCTTGATCAGGCGCTATTTG-----------------------------------------------------------------------------------GGGATAAGACCTTACA : 2357
FJ708662  : TGAGTATCTACCTAATGGAAGTCTTGATCAGGCGCTATTTG-----------------------------------------------------------------------------------GGGATAAGACCTTACA : 2286
                                                                                                                                                               
                     *      5060         *      5080         *      5100         *      5120         *      5140         *      5160         *      5180       
Genomic   : TCTTGATTGGTCAACCCGTTATGAGATATGCCTGGGAGTAGCCAGAGGTCTAGTTTATCTCCACGAGGAGGCGAGTGTTCGCATAGTACACAGGGATGTGAAGGCCAGTAACATTTTGCTCGACTCTAGACTGGTCCCAC : 5180
NM_104491 : TCTTGATTGGTCAACCCGTTATGAGATATGCCTGGGAGTAGCCAGAGGTCTAGTTTATCTCCACGAGGAGGCGAGTGTTCGCATAGTACACAGGGATGTGAAGGCCAGTAACATTTTGCTCGACTCTAGACTGGTCCCAC : 2497
FJ708662  : TCTTGATTGGTCAACCCGTTATGAGATATGCCTGGGAGTAGCCAGAGGTCTAGTTTATCTCCACGAGGAGGCGAGTGTTCGCATAGTACACAGGGATGTGAAGGCCAGTAACATTTTGCTCGACTCTAGACTGGTCCCAC : 2426
                                                                                                                                                               
                     *      5200         *      5220         *      5240         *      5260         *      5280         *      5300         *      5320       
Genomic   : AAATTTCTGATTTTGGACTTGCAAAACTGTACGATGACAAGAAAACCCACATAAGTACCCGAGTGGCAGGGACGATGTAAGTAATCCACAAACTCTGACCTTTGGTTTGCATCACAATTACAAATTAGTGAGACATGTTA : 5320
NM_104491 : AAATTTCTGATTTTGGACTTGCAAAACTGTACGATGACAAGAAAACCCACATAAGTACCCGAGTGGCAGGGACGAT---------------------------------------------------------------- : 2573
FJ708662  : AAATTTCTGATTTTGGACTTGCAAAACTGTACGATGACAAGAAAACCCACATAAGTACCCGAGTGGCAGGGACGAT---------------------------------------------------------------- : 2502
                                                                                                                                                               
                     *      5340         *      5360         *      5380         *      5400         *      5420         *      5440         *      5460       
Genomic   : GTGATGTTGCTGTATCAATGCAGTGGGTATCTTGCGCCAGAGTATGCCATGCGTGGACATCTAACAGAGAAAACGGATGTGTATGCCTTTGGTGTTGTGGCTCTTGAGCTAGTGAGTGGAAGGCCAAACTCTGATGAGAA : 5460
NM_104491 : -----------------------TGGGTATCTTGCGCCAGAGTATGCCATGCGTGGACATCTAACAGAGAAAACGGATGTGTATGCCTTTGGTGTTGTGGCTCTTGAGCTAGTGAGTGGAAGGCCAAACTCTGATGAGAA : 2690
FJ708662  : -----------------------TGGGTATCTTGCGCCAGAGTATGCCATGCGTGGACATCTAACAGAGAAAACGGATGTGTATGCCTTTGGTGTTGTGGCTCTTGAGCTAGTGAGTGGAAGGCCAAACTCTGATGAGAA : 2619
                                                                                                                                                               
                     *      5480         *      5500         *      5520         *      5540         *      5560         *      5580         *      5600       
Genomic   : TCTGGAGGAGGAAAAAAAATATCTTCTTGAATGGGTACGTTCTTGCTTATTATGATTGATTGTAAATGGATGGAGTTTAGAATGGCAAAAGCCAATAATACTTGTGTATTTTCTGTATACAGGCATGGAATTTACACGAG : 5600
NM_104491 : TCTGGAGGAGGAAAAAAAATATCTTCTTGAATGG----------------------------------------------------------------------------------------GCATGGAATTTACACGAG : 2742
FJ708662  : TCTGGAGGAGGAAAAAAAATATCTTCTTGAATGG----------------------------------------------------------------------------------------GCATGGAATTTACACGAG : 2671
                                                                                                                                                               
                     *      5620         *      5640         *      5660         *      5680         *      5700         *      5720         *      5740       
Genomic   : AAAAGCCGTGACATTGAACTAATCGATGATAAGCTGACTGATTTCAACATGGAAGAAGCAAAGCGCATGATTGGCATTGCTCTGTTGTGCACACAGACATCTCATGCCTTGAGGCCACCAATGTCACGAGTGGTGGCCAT : 5740
NM_104491 : AAAAGCCGTGACATTGAACTAATCGATGATAAGCTGACTGATTTCAACATGGAAGAAGCAAAGCGCATGATTGGCATTGCTCTGTTGTGCACACAGACATCTCATGCCTTGAGGCCACCAATGTCACGAGTGGTGGCCAT : 2882
FJ708662  : AAAAGCCGTGACATTGAACTAATCGATGATAAGCTGACTGATTTCAACATGGAAGAAGCAAAGCGCATGATTGGCATTGCTCTGTTGTGCACACAGACATCTCATGCCTTGAGGCCACCAATGTCACGAGTGGTGGCCAT : 2811
                                                                                                                                                               
                     *      5760         *      5780         *      5800         *      5820         *      5840         *      5860         *      5880       
Genomic   : GCTTTCAGGAGATGTTGAGATCGGTGATGTCACTTCTAAGCCAGGCTACGTAAGCGACTGGAGATTTGATGACACCACAGGCTCATCTCTCAGCGGATTTCAAATCAAAGACACAACAGGCTATTCCATGAGCCTTGTGG : 5880
NM_104491 : GCTTTCAGGAGATGTTGAGATCGGTGATGTCACTTCTAAGCCAGGCTACGTAAGCGACTGGAGATTTGATGACACCACAGGCTCATCTCTCAGCGGATTTCAAATCAAAGACACAACAGGCTATTCCATGAGCCTTGTGG : 3022
FJ708662  : GCTTTCAGGAGATGTTGAGATCGGTGATGTCACTTCTAAGCCAGGCTACGTAAGCGACTGGAGATTTGATGACACCACAGGCTCATCTCTCAGCGGATTTCAAATCAAAGACACAACAGGCTATTCCATGAGCCTTGTGG : 2951
                                                                                                                                                               
                     *      5900         *      5920         *      5940         *           
Genomic   : CGCCTGGCTCCGAGATATCACCCAGAGACAGCGACTTTAAGCCAATGCTTGGATCCAAGATCAATGAGGGAAGA : 5954
NM_104491 : CGCCTGGCTCCGAGATATCACCCAGAGACAGCGACTTTAAGCCAATGCTTGGATCCAAGATCAATGAGGGAAGA : 3096
FJ708662  : CGCCTGGCTCCGAGATATCACCCAGAGACAGCGACTTTAAGCCAATGCTTGGATCCAAGATCAATGAGGGAAGA : 3025


At1g56140
                                                                                                                                                               
                     *        20         *        40         *        60         *        80         *       100         *       120         *       140       
Genomic   : ATGCTCAGGCTATGGCGGTATCTGTGTCTACTCCTCACTGTCTGGTTCTTGTGTAATTTCGGTCCGGTTTACGTGGTTCGAGCTCAAAATCGAACAGGAGCCACCACACATCCCGACGAAGGTCTGTCTTCTTCTTCTTT :  140
NM_104492 : ATGCTCAGGCTATGGCGGTATCTGTGTCTACTCCTCACTGTCTGGTTCTTGTGTAATTTCGGTCCGGTTTACGTGGTTCGAGCTCAAAATCGAACAGGAGCCACCACACATCCCGACGAAG------------------- :  121
BT011697  : ATGCTCAGGCTATGGCGGTATCTGTGTCTACTCCTCACTGTCTGGTTCTTGTGTAATTTCGGTCCGGTTTACGTGGTTCGAGCTCAAAATCGAACAGGAGCCACCACACATCCCGACGAAG------------------- :  121
FJ708663  : ATGCTCAGGCTATGGCGGTATCTGTGTCTACTCCTCACTGTCTGGTTCTTGTGTAATTTCGGTCCGGTTTACGTGGTTCGAGCTCAAAATCGAACAGGAGCCACCACACATCCCGACGAAG------------------- :  121
                                                                                                                                                               
                     *       160         *       180         *       200         *       220         *       240         *       260         *       280       
Genomic   : GAACAACTTGACAAGTTCAAATTCAATCGATCGGATTGAAGACTGTGTAGCTAGGTCTTAAGCTACTCTTCTGATCCTAATTGATAAAACCGATGTTTACATTTATAATATTTTTTTATTTTATCCTAAGAAGTTTACCG :  280
NM_104492 : -------------------------------------------------------------------------------------------------------------------------------------------- :    -
BT011697  : -------------------------------------------------------------------------------------------------------------------------------------------- :    -
FJ708663  : -------------------------------------------------------------------------------------------------------------------------------------------- :    -
                                                                                                                                                               
                     *       300         *       320         *       340         *       360         *       380         *       400         *       420       
Genomic   : GTTTAGTTTCAATTGACATTTTTTGTTTAGTTAATCGTATTTTGTTAAGAGCTAATAATAATTGACTGTTGTAAATAAAAACAGCGCTAGCTTTGAACTCAATTTTCGCGGCTTGGAGGATTCGGGCGCCGAGGGAATGG :  420
NM_104492 : ------------------------------------------------------------------------------------CGCTAGCTTTGAACTCAATTTTCGCGGCTTGGAGGATTCGGGCGCCGAGGGAATGG :  177
BT011697  : ------------------------------------------------------------------------------------CGCTAGCTTTGAACTCAATTTTCGCGGCTTGGAGGATTCGGGCGCCGAGGGAATGG :  177
FJ708663  : ------------------------------------------------------------------------------------CGCTAGCTTTGAACTCAATTTTCGCGGCTTGGAGGATTCGGGCGCCGAGGGAATGG :  177
                                                                                                                                                               
                     *       440         *       460         *       480         *       500         *       520         *       540         *       560       
Genomic   : AACATCAGCGGCGAACTTTGCTCCGGCGCCGCTATCGACGCCAGTGTTCTCGACTCAAACCCTGCCTACAATCCTCTCATCAAATGCGACTGCAGTTTCGAAAACTCCACAATCTGCCGCATTACCAACATGTAGGGCCT :  560
NM_104492 : AACATCAGCGGCGAACTTTGCTCCGGCGCCGCTATCGACGCCAGTGTTCTCGACTCAAACCCTGCCTACAATCCTCTCATCAAATGCGACTGCAGTTTCGAAAACTCCACAATCTGCCGCATTACCAACAT--------- :  308
BT011697  : AACATCAGCGGCGAACTTTGCTCCGGCGCCGCTATCGACGCCAGTGTTCTCGACTCAAACCCTGCCTACAATCCTCTCATCAAATGCGACTGCAGTTTCGAAAACTCCACAATCTGCCGCATTACCAACAT--------- :  308
FJ708663  : AACATCAGCGGCGAACTTTGCTCCGGCGCCGCTATCGACGCCAGTGTTCTCGACTCAAACCCTGCCTACAATCCTCTCATCAAATGCGACTGCAGTTTCGAAAACTCCACAATCTGCCGCATTACCAACAT--------- :  308
                                                                                                                                                               
                     *       580         *       600         *       620         *       640         *       660         *       680         *       700       
Genomic   : CTTTCCCCCTTTTTTCTGCACCCCATTTGTTCGACGTATTCTCCCACTGGGAATTGACTTTTGTGGGGGAAACGAATGGTTTTCAATTTCATGTCTCAAATTCTTCTCCTCATTTTTGGATGTTGTTGATGTTTTCACAG :  700
NM_104492 : -------------------------------------------------------------------------------------------------------------------------------------------- :    -
BT011697  : -------------------------------------------------------------------------------------------------------------------------------------------- :    -
FJ708663  : -------------------------------------------------------------------------------------------------------------------------------------------- :    -
                                                                                                                                                               
                     *       720         *       740         *       760         *       780         *       800         *       820         *       840       
Genomic   : CAAGGTTTATGCGATGGAAGTTGTAGGATCTATACCTCAACAACTCTGGACCTTGGAATACCTGACAAATCTGTATGGTTTTACTATCACTTGATGATTATACAATTTCAGCTACAAAAGGAGCACACATTTTCACAATT :  840
NM_104492 : CAAGGTTTATGCGATGGAAGTTGTAGGATCTATACCTCAACAACTCTGGACCTTGGAATACCTGACAAATCTG------------------------------------------------------------------- :  381
BT011697  : CAAGGTTTATGCGATGGAAGTTGTAGGATCTATACCTCAACAACTCTGGACCTTGGAATACCTGACAAATCTG------------------------------------------------------------------- :  381
FJ708663  : CAAGGTTTATGCGATGGAAGTTGTAGGATCTATACCTCAACAACTCTGGACCTTGGAATACCTGACAAATCTG------------------------------------------------------------------- :  381
                                                                                                                                                               
                     *       860         *       880         *       900         *       920         *       940         *       960         *       980       
Genomic   : TAAAATCACTTCTTTCAGGAACTTGGGTCAAAATGTTCTCACTGGCTCACTTCCTCCTGCACTTGGAAATTTGACTCGAATGCGATGGATGTATGTCTCTGACACCTTCTTTTTTTCTTATTGTTTGTGGTGAATGTCTA :  980
NM_104492 : -------------------AACTTGGGTCAAAATGTTCTCACTGGCTCACTTCCTCCTGCACTTGGAAATTTGACTCGAATGCGATGGATG------------------------------------------------- :  453
BT011697  : -------------------AACTTGGGTCAAAATGTTCTCACTGGCTCACTTCCTCCTGCACTTGGAAATTTGACTCGAATGCGATGGATG------------------------------------------------- :  453
FJ708663  : -------------------AACTTGGGTCAAAATGTTCTCACTGGCTCACTTCCTCCTGCACTTGGAAATTTGACTCGAATGCGATGGATG------------------------------------------------- :  453
                                                                                                                                                               
                     *      1000         *      1020         *      1040         *      1060         *      1080         *      1100         *      1120       
Genomic   : CACCTTTCATTGATAATGCACCCTTATAGGAACATTTCTTGTTTCTCACTCTCAATTGTTTATCACGTAGGACTTTTGGGATCAATGCGTTGTCTGGCCCTATTCCTAAAGAAATCGGTTTGCTTACAGATTTAAGATTA : 1120
NM_104492 : -----------------------------------------------------------------------ACTTTTGGGATCAATGCGTTGTCTGGCCCTATTCCTAAAGAAATCGGTTTGCTTACAGATTTAAGATTA :  522
BT011697  : -----------------------------------------------------------------------ACTTTTGGGATCAATGCGTTGTCTGGCCCTATTCCTAAAGAAATCGGTTTGCTTACAGATTT------- :  515
FJ708663  : -----------------------------------------------------------------------ACTTTTGGGATCAATGCGTTGTCTGGCCCTATTCCTAAAGAAATCGGTTTGCTTACAGATTTAAGATTA :  522
                                                                                                                                                               
                     *      1140         *      1160         *      1180         *      1200         *      1220         *      1240         *      1260       
Genomic   : CTGTAAGCTTAGGCTGCTTCTTCATTGTTTAAATATGATAGTGTCTTCACATTGCTTCTGGAGGATTCTGACGGTTCCTTATACACTATATATATTTCTGCAGTAGTATTAGTTCAAATAACTTTTCGGGTTCTATACCA : 1260
NM_104492 : CT-----------------------------------------------------------------------------------------------------TAGTATTAGTTCAAATAACTTTTCGGGTTCTATACCA :  561
BT011697  : -------------------------------------------------------------------------------------------------------------------------------------------- :    -
FJ708663  : CT-----------------------------------------------------------------------------------------------------TAGTATTAGTTCAAATAACTTTTCGGGTTCTATACCA :  561
                                                                                                                                                               
                     *      1280         *      1300         *      1320         *      1340         *      1360         *      1380         *      1400       
Genomic   : GATGAGATTGGGAGATGTACAAAACTACAACAGATGTGAGTTTTCTGTCAATCTATAAGTGTTTATGTCTTTATATCCTTGGTTATAATTTAGATATGATTAAATATTAACCTTTGAAATCATTTAGCGATCACCGACCT : 1400
NM_104492 : GATGAGATTGGGAGATGTACAAAACTACAACAGAT--------------------------------------------------------------------------------------------------------- :  596
BT011697  : -------------------------------------------------------------------------------------------------------------------------------------------- :    -
FJ708663  : GATGAGATTGGGAGATGTACAAAACTACAACAGAT--------------------------------------------------------------------------------------------------------- :  596
                                                                                                                                                               
                     *      1420         *      1440         *      1460         *      1480         *      1500         *      1520         *      1540       
Genomic   : CAAAATTCTAGCGCAGTTGATGACTGATGGGAAATTTCTTCCATCTATTGCAGATACATAGATAGTTCGGGACTCAGCGGGGGATTACCTGTATCATTTGCTAATCTTGTGGAGCTGGAACAAGCGTAAGACATATTGAA : 1540
NM_104492 : -----------------------------------------------------ATACATAGATAGTTCGGGACTCAGCGGGGGATTACCTGTATCATTTGCTAATCTTGTGGAGCTGGAACAAGC--------------- :  668
BT011697  : -------------------------------------------------------------------------------------------------------------------------------------------- :    -
FJ708663  : -----------------------------------------------------ATACATAGATAGTTCGGGACTCAGCGGGGGATTACCTGTATCATTTGCTAATCTTGTGGAGCTGGAACAAGC--------------- :  668
                                                                                                                                                               
                     *      1560         *      1580         *      1600         *      1620         *      1640         *      1660         *      1680       
Genomic   : GTCGAATATAAGCTTTATTGTTTAACTTTAGTTGCATCTTATTTTTGTAATCTATTTATTACTTTACAATTTAGTCTTTTTTGCCGTGATGTGATTGTTTCCTTAATGTGGTACCAGTTGGATTGCGGATATGGAACTTA : 1680
NM_104492 : ---------------------------------------------------------------------------------------------------------------------TTGGATTGCGGATATGGAACTTA :  691
BT011697  : -------------------------------------------------------------------------------------------------------------------------------------------- :    -
FJ708663  : ---------------------------------------------------------------------------------------------------------------------TTGGATTGCGGATATGGAACTTA :  691
                                                                                                                                                               
                     *      1700         *      1720         *      1740         *      1760         *      1780         *      1800         *      1820       
Genomic   : CAGGTCAGATACCAGACTTTATAGGAGATTGGACCAAACTTACTACCTTGTAAGAAGTTTTCATTTTTACGGTTATATAATAGGCTGGTTCCATTGATCTTTTTTTTTAACCAGTCTTTTTTACATTTAAATTTGGGATT : 1820
NM_104492 : CAGGTCAGATACCAGACTTTATAGGAGATTGGACCAAACTTACTACCTTG------------------------------------------------------------------------------------------ :  741
BT011697  : -------------------------------------------------------------------------------------------------------------------------------------------- :    -
FJ708663  : CAGGTCAGATACCAGACTTTATAGGAGATTGGACCAAACTTACTACCTTG------------------------------------------------------------------------------------------ :  741
                                                                                                                                                               
                     *      1840         *      1860         *      1880         *      1900         *      1920         *      1940         *      1960       
Genomic   : TAACGGCTGGTACGTGTTTTACTGCAGGAGAATTCTTGGAACTGGTTTGAGTGGTCCGATACCGGCGTCGTTTTCAAACTTAACTTCTTTGACAGAGCTGTATGTGTTGCATTGTTGTCATAATCATTATCGTTTTACTG : 1960
NM_104492 : ----------------------------AGAATTCTTGGAACTGGTTTGAGTGGTCCGATACCGGCGTCGTTTTCAAACTTAACTTCTTTGACAGAGCTG---------------------------------------- :  813
BT011697  : -------------------------------------------------------------------------------------------------------------------------------------------- :    -
FJ708663  : ----------------------------AGAATTCTTGGAACTGGTTTGAGTGGTCCGATACCGGCGTCGTTTTCAAACTTAACTTCTTTGACAGAGCTG---------------------------------------- :  813


                                                                                                                                                               
                     *      1980         *      2000         *      2020         *      2040         *      2060         *      2080         *      2100       
Genomic   : TTGCTCTTCCTTTCCTTTTCTCCACATATTCATATACTATATGTAGGAGGCTTGGTGATATATCCAATGGAAATTCTTCTCTTGAATTCATCAAAGACATGAAATCTCTAAGTATATTGTAAGGCAGTTTCAACTTGAAC : 2100
NM_104492 : --------------------------------------------------CTTGGTGATATATCCAATGGAAATTCTTCTCTTGAATTCATCAAAGACATGAAATCTCTAAGTATATT---------------------- :  881
BT011697  : -------------------------------------------------------------------------------------------------------------------------------------------- :    -
FJ708663  : -----------------------------------------------AGGCTTGGTGAGATATCCAATGGAAATTCTTCTCTTGAATTCATCAAAGACATGAAATCTCTAAGTATATT---------------------- :  884
                                                                                                                                                               
                     *      2120         *      2140         *      2160         *      2180         *      2200         *      2220         *      2240       
Genomic   : TTCTAGGAATTGATCTGGTTCTCTCCTCCCTGTATTTAACATTTGTAATATATGTGGCAGAGTATTGAGGAACAATAATCTCACTGGGACAATACCGTCTAATATTGGAGAATATTCAAGTTTGCGACAACTGTACGTAT : 2240
NM_104492 : ------------------------------------------------------------AGTATTGAGGAACAATAATCTCACTGGGACAATACCGTCTAATATTGGAGAATATTCAAGTTTGCGACAACT-------- :  953
BT011697  : -------------------------------------------------------------------------------------------------------------------------------------------- :    -
FJ708663  : ------------------------------------------------------------AGTATTGAGGAACAATAATCTCACTGGGACAATACCGTCTAATATTGGAGAATATTCAAGTTTGCGACAACT-------- :  956
                                                                                                                                                               
                     *      2260         *      2280         *      2300         *      2320         *      2340         *      2360         *      2380       
Genomic   : ATATGTCAATTTGTTTCTTCAAATGCTCTAGCAGCTTTTTCAGGGGTTTTTTGTTTGTAAAAAGAGTGAGATGATTTGTGAAAGTCACGAGAAATTACGAATTGATCCTATATTTCTGATTATATAATTTCTTATTTTTC : 2380
NM_104492 : -------------------------------------------------------------------------------------------------------------------------------------------- :    -
BT011697  : -------------------------------------------------------------------------------------------------------------------------------------------- :    -
FJ708663  : -------------------------------------------------------------------------------------------------------------------------------------------- :    -
                                                                                                                                                               
                     *      2400         *      2420         *      2440         *      2460         *      2480         *      2500         *      2520       
Genomic   : CTCATTTATTGCTTAACAGGAATGGTTTGACGTTTTACCATGTAATTCATGTCTGTTTTCCAGTGATTTGAGCTTCAACAAACTACATGGAACAATTCCGGCTTCACTTTTCAACTTAAGACAGCTTACTCACTTGTAAG : 2520
NM_104492 : ---------------------------------------------------------------TGATTTGAGCTTCAACAAACTACATGGAACAATTCCGGCTTCACTTTTCAACTTAAGACAGCTTACTCACTTGT--- : 1027
BT011697  : -------------------------------------------------------------------------------------------------------------------------------------------- :    -
FJ708663  : ---------------------------------------------------------------TGATTTGAGCTTCAACAAACTACATGGAACAATTCCGGCTTCACTTTTCAACTTAAGACAGCTTACTCACTTGT--- : 1030
                                                                                                                                                               
                     *      2540         *      2560         *      2580         *      2600         *      2620         *      2640         *      2660       
Genomic   : ATGATATGTTTTCTACTTTTGGATCTTTATGTACAGCGACCCGGATTATTGTGAAAAAATAACTGTTGTCTTCATATTAGGTTTCTGGGGAACAACACGTTGAATGGCTCATTGCCCACTCAAAAGGGGCAGTCTCTGAG : 2660
NM_104492 : ----------------------------------------------------------------------------------TTCTGGGGAACAACACGTTGAATGGCTCATTGCCCACTCAAAAGGGGCAGTCTCTGAG : 1085
BT011697  : -------------------------------------------------------------------------------------------------------------------------------------------- :    -
FJ708663  : ----------------------------------------------------------------------------------TTCTGGGGAACAACACGTTGAATGGCTCATTGCCCACTCAAAAGGGGCAGTCTCTGAG : 1088
                                                                                                                                                               
                     *      2680         *      2700         *      2720         *      2740         *      2760         *      2780         *      2800       
Genomic   : CAATGTGTAAGTAACAAAATCCTTTATAATTTTTCCATCTTTAAAAGCCATGTCCCCTAGTTTCTTGGACGGTTGTTAACTGATATATACTCAAGTTTCGTACACTTTTTACTCTGCATTTATTCAAGTCGAATAAGAAT : 2800
NM_104492 : CAATGT-------------------------------------------------------------------------------------------------------------------------------------- : 1091
BT011697  : -------------------------------------------------------------------------------------------------------------------------------------------- :    -
FJ708663  : CAATGT-------------------------------------------------------------------------------------------------------------------------------------- : 1094
                                                                                                                                                               
                     *      2820         *      2840         *      2860         *      2880         *      2900         *      2920         *      2940       
Genomic   : TCCACTCTTGGTGACACAAATGACATGCCAAGATAATTAGGAAACTATTTTGGTAACATATTCTGGCGATCCTAATTAGTGTTCTTCTATGCTTACAGAGATGTGTCATACAATGATTTGTCTGGAAGTCTTCCGTCATG : 2940
NM_104492 : --------------------------------------------------------------------------------------------------AGATGTGTCATACAATGATTTGTCTGGAAGTCTTCCGTCATG : 1133
BT011697  : -------------------------------------------------------------------------------------------------------------------------------------------- :    -
FJ708663  : --------------------------------------------------------------------------------------------------AGATGTGTCATACAATGATTTGTCTGGAAGTCTTCCGTCATG : 1136
                                                                                                                                                               
                     *      2960         *      2980         *      3000         *      3020         *      3040         *      3060         *      3080       
Genomic   : GGTCAGTTTACCAAACTTGAATCTGTAAGTAAACAAAACACTATATCACGACTTATATTAACAGTGTTTTACATTCAGAAATTTCCCCACATGTATATTCATTAGACTTCTTCATATCCAGCATGAAGCTTCCTTTTTGT : 3080
NM_104492 : GGTCAGTTTACCAAACTTGAATCT-------------------------------------------------------------------------------------------------------------------- : 1157
BT011697  : -------------------------------------------------------------------------------------------------------------------------------------------- :    -
FJ708663  : GGTCAGTTTACCAAACTTGAATCT-------------------------------------------------------------------------------------------------------------------- : 1160
                                                                                                                                                               
                     *      3100         *      3120         *      3140         *      3160         *      3180         *      3200         *      3220       
Genomic   : ATGAAATTATTTATTATAATCATGTTAGAGAGAATAATATTAAACTAAACTGTATGTTCTTTGTGCAGCAACTTAGTTGCTAACAACTTCACATTGGAAGGTCTTGACAACAGGTGACTTTTTAATTAATGTTCTTGTTA : 3220
NM_104492 : --------------------------------------------------------------------CAACTTAGTTGCTAACAACTTCACATTGGAAGGTCTTGACAACAG--------------------------- : 1202
BT011697  : -------------------------------------------------------------------------------------------------------------------------------------------- :    -
FJ708663  : --------------------------------------------------------------------CAACTTAGTTGCTAACAACTTCACATTGGAAGGTCTTGACAACAG--------------------------- : 1205
                                                                                                                                                               
                     *      3240         *      3260         *      3280         *      3300         *      3320         *      3340         *      3360       
Genomic   : GGGCTTGAAATTGCTTTCCCTTTGTTTCTCTTTTCTCTTTTTTATTTGTTGCTTGCAAATAGTTTAATCATATGTTTAATTTCTTTCTGTAAAAAGGGTTTTATCAGGACTGAACTGCCTGCAGAAGAACTTCCCATGCA : 3360
NM_104492 : ------------------------------------------------------------------------------------------------GGTTTTATCAGGACTGAACTGCCTGCAGAAGAACTTCCCATGCA : 1246
BT011697  : -------------------------------------------------------------------------------------------------------------------------------------------- :    -
FJ708663  : ------------------------------------------------------------------------------------------------GGTTTTATCAGGACTGAACTGCCTGCAGAAGAACTTCCCATGCA : 1249
                                                                                                                                                               
                     *      3380         *      3400         *      3420         *      3440         *      3460         *      3480         *      3500       
Genomic   : ATCGAGGGAAAGGAATATGTAAGTGTAACAGCAATATCCGCTTCTTGAGCAAATGTTGGAAAAAAATCCGCTAAATTTTTTCTCTTTACACTGGTGTCTAGATTCTGACTTCTCAATCAACTGCGGAGGCCCAGAGATAA : 3500
NM_104492 : ATCGAGGGAAAGGAATAT-----------------------------------------------------------------------------------ATTCTGACTTCTCAATCAACTGCGGAGGCCCAGAGATAA : 1303
BT011697  : -------------------------------------------------------------------------------------------------------------------------------------------- :    -
FJ708663  : ATCGAGGGAAAGGAATAT-----------------------------------------------------------------------------------ATTCTGACTTCTCAATCAACTGCGGAGGCCCAGAGATAA : 1306
                                                                                                                                                               
                     *      3520         *      3540         *      3560         *      3580         *      3600         *      3620         *      3640       
Genomic   : GATCTGTAACTGAAGCAGTATTTGAGAGGGAGGACGAGGATCTTGGACCAGCTTCATTTGTCGTGAGTGCTGGTCAGAGATGGGCAGCCAGTAGTGTAGGACTTTTTGCCGGAAGTAGCAACAATATATACATATCTACT : 3640
NM_104492 : GATCTGTAACTGAAGCAGTATTTGAGAGGGAGGACGAGGATCTTGGACCAGCTTCATTTGTCGTGAGTGCTGGTCAGAGATGGGCAGCCAGTAGTGTAGGACTTTTTGCCGGAAGTAGCAACAATATATACATATCTACT : 1443
BT011697  : -------------------------------------------------------------------------------------------------------------------------------------------- :    -
FJ708663  : GATCTGTAACTGAAGCAGTATTTGAGAGGGAGGACGAGGATCTTGGACCAGCTTCATTTGTCGTGAGTGCTGGTCAGAGATGGGCAGCCAGTAGTGTAGGACTTTTTGCCGGAAGTAGCAACAATATATACATATCTACT : 1446
                                                                                                                                                               
                     *      3660         *      3680         *      3700         *      3720         *      3740         *      3760         *      3780       
Genomic   : TCACAATCACAATTTGTCAACACTTTGGACTCAGAGCTATTTCAGTCAGCAAGACTTTCTGCATCTTCCCTAAGGTATTATGGGTTGGGTCTAGAAAATGGAGGCTATACCGTCACACTTCAGTTTGCTGAAATACAAAT : 3780
NM_104492 : TCACAATCACAATTTGTCAACACTTTGGACTCAGAGCTATTTCAGTCAGCAAGACTTTCTGCATCTTCCCTAAGGTATTATGGGTTGGGTCTAGAAAATGGAGGCTATACCGTCACACTTCAGTTTGCTGAAATACAAAT : 1583
BT011697  : -------------------------------------------------------------------------------------------------------------------------------------------- :    -
FJ708663  : TCACAATCACAATTTGTCAACACTTTGGACTCAGAGCTATTTCAGTCAGCAAGACTTTCTGCATCTTCCCTAAG------------------------------------------------------------------ : 1520
                                                                                                                                                               
                     *      3800         *      3820         *      3840         *      3860         *      3880         *      3900         *      3920       
Genomic   : TCTAGGTTCTACTTCCAACACTTGGAGAGGTTTAGGAAGACGACGTTTTGACATTTATGTCCAGGTCTGCACTACTTGACCGAGCTTTCTGCTTTACAAATTAAATTTTGGTGTAATTATCTGCTTCCGACTTTTGATTC : 3920
NM_104492 : TCTAGGTTCTACTTCCAACACTTGGAGAGGTTTAGGAAGACGACGTTTTGACATTTATGTCCAG---------------------------------------------------------------------------- : 1647
BT011697  : -------------------------------------------------------------------------------------------------------------------------------------------- :    -
FJ708663  : ------TTCTACTTCCAACACTTGGAGAGGTTTAGGAAGACGACGTTTTGACATTTATGTCCAG---------------------------------------------------------------------------- : 1578
                                                                                                                                                               


                     *      3940         *      3960         *      3980         *      4000         *      4020         *      4040         *      4060       
Genomic   : TGATGAATTCTAAGACTAGCTCACTTTTTCCAGGGAAGACTTGTTGAAAAGGACTTCGATGTACGCAGAACAGCTGGTGACTCCACTGTTCGAGCAGTTCAGAGAGAATATAAAGCAAATGTATCACAAAATCATCTCGA : 4060
NM_104492 : ---------------------------------GGAAGACTTGTTGAAAAGGACTTCGATGTACGCAGAACAGCTGGTGACTCCACTGTTCGAGCAGTTCAGAGAGAATATAAAGCAAATGTATCACAAAATCATCTCGA : 1754
BT011697  : -------------------------------------------------------------------------------------------------------------------------------------------- :    -
FJ708663  : --------------------------------------ACTTGTTGAAAAGGACTTCGATGTACGCAGAACAGCTGGTGACTCCACTGTTCGAGCAGTTCAGAGAGAATATAAAGCAAATGTATCACAAAATCATCTCGA : 1680
                                                                                                                                                               
                     *      4080         *      4100         *      4120         *      4140         *      4160         *      4180         *      4200       
Genomic   : AATTCATCTTTTCTGGGCTGGAAAAGGAACATGCTGTATTCCTATCCAAGGGGCTTATGGGCCATTAATATCGGCCGTCGGTGCAACACCAGGTAAATAAATGGTGAATTCTTCTTGTTTTACACCATCTCCTGATGCTA : 4200
NM_104492 : AATTCATCTTTTCTGGGCTGGAAAAGGAACATGCTGTATTCCTATCCAAGGGGCTTATGGGCCATTAATATCGGCCGTCGGTGCAACACCAG------------------------------------------------ : 1846
BT011697  : -------------------------------------------------------------------------------------------------------------------------------------------- :    -
FJ708663  : AATTCATCTTTTCTGGGCTGGAAAAGGAACATGCTGTATTCCTATCCAAGGGGCTTATGGGCCATTAATATCGGCCGTCGGTGCAACACCAG------------------------------------------------ : 1772
                                                                                                                                                               
                     *      4220         *      4240         *      4260         *      4280         *      4300         *      4320         *      4340       
Genomic   : GTTAGTGCTTAAGTGTTACATATTTCTGTTCAGATTTCACACCAACTGTGGGTAATAGGCCACCATCAAAGGGAAAGAGCATGACTGGTACTATTGTGGGTGTCATTGTTGGCGTTGGACTTTTGAGCATCATTTCTGGT : 4340
NM_104492 : ---------------------------------ATTTCACACCAACTGTGGGTAATAGGCCACCATCAAAGGGAAAGAGCATGACTGGTACTATTGTGGGTGTCATTGTTGGCGTTGGACTTTTGAGCATCATTTCTGGT : 1953
BT011697  : -------------------------------------------------------------------------------------------------------------------------------------------- :    -
FJ708663  : ---------------------------------ATTTCACACCAACTGTGGGTAATAGGCCACCATCAAAGGGAAAGAGCATGACTGGTACTATTGTGGGTGTCATTGTTGGCGTTGGACTTTTGAGCATCATTTCTGGT : 1879
                                                                                                                                                               
                     *      4360         *      4380         *      4400         *      4420         *      4440         *      4460         *      4480       
Genomic   : GTGGTTATCTTTATCATCCGAAAAAGAAGAAAGCGGTACACAGATGATGAAGGTATAGATGTAAAAAGCTCTTGAACTGTAGAATTTTGGCCCTCTGATTAAAAATTAGTTATATATCATCTCTCATATTGGTCGATTTT : 4480
NM_104492 : GTGGTTATCTTTATCATCCGAAAAAGAAGAAAGCGGTACACAGATGATGAAG---------------------------------------------------------------------------------------- : 2005
BT011697  : -------------------------------------------------------------------------------------------------------------------------------------------- :    -
FJ708663  : GTGGTTATCTTTATCATCCGAAAAAGAAGAAAGCGGTACACAGATGATGAAG---------------------------------------------------------------------------------------- : 1931
                                                                                                                                                               
                     *      4500         *      4520         *      4540         *      4560         *      4580         *      4600         *      4620       
Genomic   : AAGAACCGAATTGTTTCTGGCAGAGATACTTAGTATGGACGTTAAGCCTTACACCTTCACTTACTCGGAACTTAAAAGTGCAACTCAAGATTTTGATCCCTCAAACAAGCTTGGAGAGGGAGGATTTGGCCCTGTTTATA : 4620
NM_104492 : -----------------------AGATACTTAGTATGGACGTTAAGCCTTACACCTTCACTTACTCGGAACTTAAAAGTGCAACTCAAGATTTTGATCCCTCAAACAAGCTTGGAGAGGGAGGATTTGGCCCTGTTTATA : 2122
BT011697  : -------------------------------------------------------------------------------------------------------------------------------------------- :    -
FJ708663  : ---AACCGAATTGTTTCTGGCAGAGATACTTAGTATGGACGTTAAGCCTTACACCTTCACTTACTCGGAACTTAAAAGTGCAACTCAAGATTTTGATCCCTCAAACAAGCTTGGAGAGGGAGGATTTGGCCCTGTTTATA : 2068
                                                                                                                                                               
                     *      4640         *      4660         *      4680         *      4700         *      4720         *      4740         *      4760       
Genomic   : AGGTAAGTCCCTACTGAAATTTGTACATATTTGAGATATTAGGAAAGAAGAGTATATGTGTACTATCTTTTGTTTTGATATTACCTTTTGTTTTGACAATATCATTTATTGAATAATGCATTCAACAGGGAAAACTCAAT : 4760
NM_104492 : AGG------------------------------------------------------------------------------------------------------------------------------GAAAACTCAAT : 2136
BT011697  : -------------------------------------------------------------------------------------------------------------------------------------------- :    -
FJ708663  : AGG------------------------------------------------------------------------------------------------------------------------------GAAAACTCAAT : 2082
                                                                                                                                                               
                     *      4780         *      4800         *      4820         *      4840         *      4860         *      4880         *      4900       
Genomic   : GATGGAAGAGAGGTAGCAGTGAAGCTATTGTCGGTGGGATCCCGACAAGGGAAGGGACAATTTGTTGCAGAAATTGTAGCAATTTCTGCAGTCCAACATCGCAACTTAGTAAAACTTTATGGGTGCTGCTATGAAGGAGA : 4900
NM_104492 : GATGGAAGAGAGGTAGCAGTGAAGCTATTGTCGGTGGGATCCCGACAAGGGAAGGGACAATTTGTTGCAGAAATTGTAGCAATTTCTGCAGTCCAACATCGCAACTTAGTAAAACTTTATGGGTGCTGCTATGAAGGAGA : 2276
BT011697  : -------------------------------------------------------------------------------------------------------------------------------------------- :    -
FJ708663  : GATGGAAGAGAGGTAGCAGTGAAGCTATTGTCGGTGGGATCCCGACAAGGGAAGGGACAATTTGTTGCAGAAATTGTAGCAATTTCTGCAGTCCAACATCGCAACTTAGTAAAACTTTATGGGTGCTGCTATGAAGGAGA : 2222
                                                                                                                                                               
                     *      4920         *      4940         *      4960         *      4980         *      5000         *      5020         *      5040       
Genomic   : GCATCGTTTGCTTGTATATGAATACCTTCCTAACGGAAGTCTTGATCAGGCTCTATTTGGTACGCACAGAAGCATGTTTATCGATCCATGCTTCTGCCAGGCTAAAATCACTCATTATGTACATGTCATTGGTTTGAATG : 5040
NM_104492 : GCATCGTTTGCTTGTATATGAATACCTTCCTAACGGAAGTCTTGATCAGGCTCTATTTGG-------------------------------------------------------------------------------- : 2336
BT011697  : -------------------------------------------------------------------------------------------------------------------------------------------- :    -
FJ708663  : GCATCGTTTGCTTGTATATGAATACCTTCCTAACGGAAGTCTTGATCAGGCTCTATTTGG-------------------------------------------------------------------------------- : 2282
                                                                                                                                                               
                     *      5060         *      5080         *      5100         *      5120         *      5140         *      5160         *      5180       
Genomic   : TTGCAGGGGAAAAGACTTTGCATCTTGATTGGTCAACCCGTTATGAGATATGCCTGGGAGTAGCCAGAGGTCTAGTCTATCTCCATGAGGAGGCAAGGCTTCGCATAGTACACAGGGATGTGAAGGCCAGCAACATTTTG : 5180
NM_104492 : -------GGAAAAGACTTTGCATCTTGATTGGTCAACCCGTTATGAGATATGCCTGGGAGTAGCCAGAGGTCTAGTCTATCTCCATGAGGAGGCAAGGCTTCGCATAGTACACAGGGATGTGAAGGCCAGCAACATTTTG : 2469
BT011697  : -------------------------------------------------------------------------------------------------------------------------------------------- :    -
FJ708663  : -------GGAAAAGACTTTGCATCTTGATTGGTCAACCCGTTATGAGATATGCCTGGGAGTAGCCAGAGGTCTAGTCTATCTCCATGAGGAGGCAAGGCTTCGCATAGTACACAGGGATGTGAAGGCCAGCAACATTTTG : 2415
                                                                                                                                                               
                     *      5200         *      5220         *      5240         *      5260         *      5280         *      5300         *      5320       
Genomic   : CTTGACTCTAAATTAGTACCAAAAGTTTCTGATTTTGGGCTTGCAAAACTGTACGATGACAAGAAAACCCACATAAGTACCCGGGTTGCAGGGACAATGTAAGTAACTCCCAACCCTGACCTCTGGTTTGCAACACAACT : 5320
NM_104492 : CTTGACTCTAAATTAGTACCAAAAGTTTCTGATTTTGGGCTTGCAAAACTGTACGATGACAAGAAAACCCACATAAGTACCCGGGTTGCAGGGACAAT------------------------------------------ : 2567
BT011697  : -------------------------------------------------------------------------------------------------------------------------------------------- :    -
FJ708663  : CTTGACTCTAAATTAGTACCAAAAGTTTCTGATTTTGGGCTTGCAAAACTGTACGATGACAAGAAAACCCACATAAGTACCCGGGTTGCAGGGACAAT------------------------------------------ : 2513
                                                                                                                                                               
                     *      5340         *      5360         *      5380         *      5400         *      5420         *      5440         *      5460       
Genomic   : ATGGATTTATAACACATGTTGGTGATTTTACTGTATCAATGCAGTGGGTATCTTGCGCCAGAGTATGCCATGCGTGGACATCTAACAGAGAAAACGGATGTATATGCCTTTGGTGTTGTGGCTCTTGAGCTAGTGAGTGG : 5460
NM_104492 : --------------------------------------------TGGGTATCTTGCGCCAGAGTATGCCATGCGTGGACATCTAACAGAGAAAACGGATGTATATGCCTTTGGTGTTGTGGCTCTTGAGCTAGTGAGTGG : 2663
BT011697  : -------------------------------------------------------------------------------------------------------------------------------------------- :    -
FJ708663  : --------------------------------------------TGGGTATCTTGCGCCAGAGTATGCCATGCGTGGACATCTAACAGAGAAAACGGATGTATATGCCTTTGGTGTTGTGGCTCTTGAGCTAGTGAGTGG : 2609
                                                                                                                                                               
                     *      5480         *      5500         *      5520         *      5540         *      5560         *      5580         *      5600       
Genomic   : AAGGCCAAACTCTGATGAGAACTTGGAGGATGAAAAAAGATATCTTCTTGAATGGGTACGTCCATACTTCAACACTTTTTGATAATGAACACAACTTCAATGTATGTGACCATGAGAGGCTAATCTGGGTTATGCCCTTT : 5600
NM_104492 : AAGGCCAAACTCTGATGAGAACTTGGAGGATGAAAAAAGATATCTTCTTGAATGGG------------------------------------------------------------------------------------ : 2719
BT011697  : -------------------------------------------------------------------------------------------------------------------------------------------- :    -
FJ708663  : AAGGCCAAACTCTGATGAGAACTTGGAGGATGAAAAAAGATATCTTCTTGAATGGG------------------------------------------------------------------------------------ : 2665
                                                                                                                                                               
                     *      5620         *      5640         *      5660         *      5680         *      5700         *      5720         *      5740       
Genomic   : AGCAAAAGCTGATATTGATTGTGTAACTTGTTCACAGGCATGGAATCTACACGAGAAAGGCCGTGAAGTTGAACTTATTGATCATCAGCTGACTGAATTCAACATGGAAGAAGGGAAACGCATGATTGGCATAGCCCTGC : 5740
NM_104492 : --------------------------------------CATGGAATCTACACGAGAAAGGCCGTGAAGTTGAACTTATTGATCATCAGCTGACTGAATTCAACATGGAAGAAGGGAAACGCATGATTGGCATAGCCCTGC : 2821
BT011697  : -------------------------------------------------------------------------------------------------------------------------------------------- :    -
FJ708663  : --------------------------------------CATGGAATCTACACGAGAAAGGCCGTGAAGTTGAACTTATTGATCATCAGCTGACTGAATTCAACATGGAAGAAGGGAAACGCATGATTGGCATAGCCCTGC : 2767
                                                                                                                                                               
                     *      5760         *      5780         *      5800         *      5820         *      5840         *      5860         *      5880       
Genomic   : TGTGTACACAAACATCTCATGCCTTGAGACCACCAATGTCACGAGTGGTGGCCATGCTTTCAGGAGATGTTGAGGTCAGTGATGTCACTTCAAAGCCAGGCTACCTAACCGACTGGAGATTTGATGACACCACAGCCTCC : 5880
NM_104492 : TGTGTACACAAACATCTCATGCCTTGAGACCACCAATGTCACGAGTGGTGGCCATGCTTTCAGGAGATGTTGAGGTCAGTGATGTCACTTCAAAGCCAGGCTACCTAACCGACTGGAGATTTGATGACACCACAGCCTCC : 2961
BT011697  : --------------------------------------------------------------------------------------------------------------------------GATGACACCACAGCCTCC :  533
FJ708663  : TGTGTACACAAACATCTCATGCCTTGAGACCACCAATGTCACGAGTGGTGGCCATGCTTTCAGGAGATGTTGAGGTCAGTGATGTCACTTCAAAGCCAGGCTACCTAACCGACTGGAGATTTGATGACACCACAGCCTCC : 2907                                                                                                                                                          
                     *      5900         *      5920         *      5940         *      5960         *      5980         *      6000         *            
Genomic   : TCAATCAGCGGCTTTCCATTAAGAAACACACAGGCTTCTGAATCCTTCACGAGCTTTGTGGCGCCTAGATCCGAGATATCACCGAGAAACAACGACGCTAGGCCAATGCTTGGAGCCCAGATGAATGAGGGAAGA : 6015
NM_104492 : TCAATCAGCGGCTTTCCATTAAGAAACACACAGGCTTCTGAATCCTTCACGAGCTTTGTGGCGCCTAGATCCGAGATATCACCGAGAAACAACGACGCTAGGCCAATGCTTGGAGCCCAGATGAATGAGGGAAGA : 3096
BT011697  : TCAATCAGCGGCTTTCCATTAAGAAACACACAGGCTTCTGAATCCTTCACGAGCTTTGTGGCGCCTAGATCCGAGATATCACCGAGAAACAACGACGCTAGGCCAATGCTTGGAGCCCAGATGAATGAGGGAAGA :  668
FJ708663  : TCAATCAGCGGCTTTCCATTAAGAAACACACAGGCTTCTGAATCCTTCACGAGCTTTGTGGCGCCTAGATCCGAGATATCACCGAGAAACAACGACGCTAGGCCAATGCTTGGAGCCCAGATGAATGAGGGAAGA : 3042

At2g28970
                                                                                                                                                               
                     *        20         *        40         *        60         *        80         *       100         *       120         *       140       
Genomic   : ATGATGAGCCATCTTTTGTTGGCCATAATTGGAACCTTTGCCGTTATAGTTGGCGCCCAAAAACAAGAAGGTAATAATTTATAAACTCTTTAGTAGATATTCACCGAGGATCATCCATCCTTTAAATTGATATATCTTCT :  140
NM_128456 : ATGATGAGCCATCTTTTGTTGGCCATAATTGGAACCTTTGCCGTTATAGTTGGCGCCCAAAAACAAGAAG---------------------------------------------------------------------- :   70
FJ708705  : ATGATGAGCCATCTTTTGTTGGCCATAATTGGAACCTTTGCCGTTATAGTTGGCGCCCAAAAACAAGAAG---------------------------------------------------------------------- :   70
                                                                                                                                                               
                     *       160         *       180         *       200         *       220         *       240         *       260         *       280       
Genomic   : ATATATAGTTTTTTTTTAAACTTCGTCGATCCTTTTTATGCTTGAACAGGTTTCATCAGTTTGGATTGCGGATTTCCTATTGAGGAATCTCCTTATAGTGATCCATCTACTGGATTAACATTCACATCAGATTCAACATT :  280
NM_128456 : -------------------------------------------------GTTTCATCAGTTTGGATTGCGGATTTCCTATTGAGGAATCTCCTTATAGTGATCCATCTACTGGATTAACATTCACATCAGATTCAACATT :  161
FJ708705  : -------------------------------------------------GTTTCATCAGTTTGGATTGCGGATTTCCTATTGAGGAATCTCCTTATAGTGATCCATCTACTGGATTAACATTCACATCAGATTCAACATT :  161
                                                                                                                                                               
                     *       300         *       320         *       340         *       360         *       380         *       400         *       420       
Genomic   : TATCCAGACTGGAGAAAGTGGTAGAGTCGATAAAGAGCTCAACAAAATATTCCGAAAACCATATTTGACTCTAAGATACTTTCCGGAAGGAAAGCGTAATTGTTAGTCTGAATGTCACTCGTGGCACAAATTATCTTATA :  420
NM_128456 : TATCCAGACTGGAGAAAGTGGTAGAGTCGATAAAGAGCTCAACAAAATATTCCGAAAACCATATTTGACTCTAAGATACTTTCCGGAAGGAAAGCGTAATTG-------------------------------------- :  263
FJ708705  : TATCCAGACTGGAGAAAGTGGTAGAGTCGATAAAGAGCTCAACAAAATATTCCGAAAACCATATTTGACTCTAAGATACTTTCCGGAAGGAAAGCGTAATTGTTAGTCTGAATGTCACTCGTGGCACAAATTATCTTATA :  301
                                                                                                                                                               
                     *       440         *       460         *       480         *       500         *       520         *       540         *       560       
Genomic   : TTGGTTACCTTCATATATGGAAATTATGATGGTCTTAATATTTATCCAAACTTTGATCTCTATCTTGGTCCTGATAAGTGGGCAAGAATAGATATGGAAGGAAGACAAAATGGTACATTCGAGGAGATCATACACAGAGT :  560
NM_128456 : -------------------------------------------------------------------------------------------------------------------------------------------- :    -
FJ708705  : TTGGTTACCTTCATATATGGAAATTATGATGGTCTTAATATTTATCCAAACTTTGATCTCTATCTTGGTCCTGATAAGTGGGCAAGAATAGATATGGAAGGAAGACAAAATGGTACATTCGAGGAGATCATACACAGAGT :  441
                                                                                                                                                               
                     *       580         *       600         *       620         *       640         *       660         *       680         *       700       
Genomic   : GAGGTCAAGCTCTTTGGATATTTGTCTTGTTAAAACAGGACCAACCTCGCCAATAATATCATCTATAGAACTACGACCAATGAGAAATGATACTTATCTTACGCATTCAGGCTCACTGAGGAACTCTTTCCGGGTTCATT :  700
NM_128456 : ---------------------------------------------------------------------------------------------------------------CTCACTGAGGAACTCTTTCCGGGTTCATT :  292
FJ708705  : GAGGTCAAGCTCTTTGGATATTTGTCTTGTTAAAACAGGACCAACCTCGCCAATAATATCATCTATAGAACTACGACCAATGAGAAATGATACTTATCTTACGCATTCAGGCTCACTGAGGAACTCTTTCCGGGTTCATT :  581
                                                                                                                                                               
                     *       720         *       740         *       760         *       780         *       800         *       820         *       840       
Genomic   : GCAGCACTTCTGATAGCGAAATAAGGTAATTAGTTGACTTTGTTTAAAACAATAAGTGGGAGTTTTCTGTCATTAATCATACATGGCATCAATTTTCTTATACATAAATTATTGTACTTTTGGTTAGTATATAAACATCG :  840
NM_128456 : GCAGCACTTCTGATAGCGAAATAAGGTA---------------------------------------------------------------------------------------------------------------- :  320
FJ708705  : GCAGCACTTCTGATAGCGAAATAAGGTA---------------------------------------------------------------------------------------------------------------- :  609
                                                                                                                                                               
                     *       860         *       880         *       900         *       920         *       940         *       960         *       980       
Genomic   : TTATTCATGATTAACTTGGATAAAAAAAAACAAGTTGCAACTATCACCGCACGACATGTTTTGTTTTAATCAATTTTCTACATTTTTTTTTTTGTTATTTATTATCAAACTATCATATCGTTATACAATTTTTTTAAACA :  980
NM_128456 : -------------------------------------------------------------------------------------------------------------------------------------------- :    -
FJ708705  : -------------------------------------------------------------------------------------------------------------------------------------------- :    -
                                                                                                                                                               
                     *      1000         *      1020         *      1040         *      1060         *      1080         *      1100         *      1120       
Genomic   : TGTAAACAAAAATGATTCGTGTATGAAATAAATGTAGTTTGCATTGAGATTAGATTTGTGTGTGTCAGCTATTTTTTTTCTATTTAAATCGGTTTTAACAAATAACAAGGATTGATTTGCTTAATTATGCTGCCTAATGC : 1120
NM_128456 : -------------------------------------------------------------------------------------------------------------------------------------------- :    -
FJ708705  : -------------------------------------------------------------------------------------------------------------------------------------------- :    -
                                                                                                                                                               
                     *      1140         *      1160         *      1180         *      1200         *      1220         *      1240         *      1260       
Genomic   : AATTTATTTGCAGGTATGATGATGACTCCTATGATCGTGTCTGGTATCCATTCTTCAGCTCTTCGTTTAGCTATATAACGACTAGTCTCAATATAAACAATTCAGATACATTTGAGATACCAAAAGCTGCACTCAAAAGT : 1260
NM_128456 : ----------------TGATGATGACTCCTATGATCGTGTCTGGTATCCATTCTTCAGCTCTTCGTTTAGCTATATAACGACTAGTCTCAATATAAACAATTCAGATACATTTGAGATACCAAAAGCTGCACTCAAAAGT :  444
FJ708705  : ----------------TGATGATGACTCCTATGATCGTGTCTGGTATCCATTCTTCAGCTCTTCGTTTAGCTATATAACGACTAGTCTCAATATAAACAATTCAGATACATTTGAGATACCAAAAGCTGCACTCAAAAGT :  733
                                                                                                                                                               
                     *      1280         *      1300         *      1320         *      1340         *      1360         *      1380         *      1400       
Genomic   : GCTGCCACGCCTAAAAACGCTAGTGCACCGCTAATCATAACTTGGAAACCAAGACCCTCTAACGCTGAAGTTTACTTCTATCTTCATTTCGCTGAGATACAAACCCTTGCAGCCAACGAGACGAGGGAATTCGACATTGT : 1400
NM_128456 : GCTGCCACGCCTAAAAACGCTAGTGCACCGCTAATCATAACTTGGAAACCAAGACCCTCTAACGCTGAAGTTTACTTCTATCTTCATTTCGCTGAGATACAAACCCTTGCAGCCAACGAGACGAGGGAATTCGACATTGT :  584
FJ708705  : GCTGCCACGCCTAAAAACGCTAGTGCACCGCTAATCATAACTTGGAAACCAAGACCCTCTAACGCTGAAGTTTACTTCTATCTTCATTTCGCTGAGATACAAACCCTTGCAGCCAACGAGACGAGGGAATTCGACATTGT :  873
                                                                                                                                                               
                     *      1420         *      1440         *      1460         *      1480         *      1500         *      1520         *      1540       
Genomic   : TTTCAAAGGAAACTTTAATTATTCAGCTTTTAGTCCTACCAAGTTAGAGCTACTTACATTTTTCACAAGTGGACCAGTGCAATGTGATTCAGATGGTTGCAATTTACAGCTCGTAAGAACTCCGAATTCAACCCTTCCAC : 1540
NM_128456 : TTTCAAAGGAAACTTTAATTATTCAGCTTTTAGTCCTACCAAGTTAGAGCTACTTACATTTTTCACAAGTGGACCAGTGCAATGTGATTCAGATGGTTGCAATTTACAGCTCGTAAGAACTCCGAATTCAACCCTTCCAC :  724
FJ708705  : TTTCAAAGGAAACTTTAATTATTCAGCTTTTAGTCCTACCAAGTTAGAGCTACTTACATTTTTCACAAGTGGACCAGTGCAATGTGATTCAGATGGTTGCAATTTACAGCTCGTAAGAACTCCGAATTCAACCCTTCCAC : 1013
                                                                                                                                                               
                     *      1560         *      1580         *      1600         *      1620         *      1640         *      1660         *      1680       
Genomic   : CTTTGATCAACGCTCTTGAGGCTTACACTATTATCGAATTCCCACAGTTGGAAACAAGCCTAAGTGATGGTACCTCACTCAATCTTTCACTTATTACCACATAATTGTTTTTGGCTTTCCATATATGCTATTGATTTCTA : 1680
NM_128456 : CTTTGATCAACGCTCTTGAGGCTTACACTATTATCGAATTCCCACAGTTGGAAACAAGCCTAAGTGATG----------------------------------------------------------------------- :  793
FJ708705  : CTTTGATCAACGCTCTTGAGGCTTACACTATTATCGAATTCCCACAGTTGGAAACAAGCCTAAGTGATGGTACCTCACTCAATCTTTCACTTATTACCACATAATTGTTTTTGGCTTTCCATATATGCTATTGATTTCTA : 1153
                                                                                                                                                               
                     *      1700         *      1720         *      1740         *      1760         *      1780         *      1800         *      1820       
Genomic   : TTGTTTATGATAGTTAATGCTATCAAAAACATTAAAGCTACGTACCGGCTAAGTAAAACTAGTTGGCAAGGAGATCCATGTCTCCCTCAAGAATTATCCTGGGAAAATCTTAGATGCAGTTACACAAATAGTTCTACCCC : 1820
NM_128456 : -------------TTAATGCTATCAAAAACATTAAAGCTACGTACCGGCTAAGTAAAACTAGTTGGCAAGGAGATCCATGTCTCCCTCAAGAATTATCCTGGGAAAATCTTAGATGCAGTTACACAAATAGTTCTACCCC :  920
FJ708705  : TTGTTTATGATAGTTAATGCTATCAAAAACATTAAAGCTACGTACCGGCTAAGTAAAACTAGTTGGCAAGGAGATCCATGTCTCCCTCAAGAATTATCCTGGGAAAATCTTAGATGCAGTTACACAAATAGTTCTACCCC : 1293
                                                                                                                                                               
                     *      1840         *      1860         *      1880         *      1900         *      1920         *      1940         *      1960       
Genomic   : ACCAAAAATCATTTCATTGTAAGGGCATTACCAAACATAGTTCCTATTTTTAAAACAAATTTAACCAAATATATTTGTTAAGTGTACATTTTATTTTATTTGTTGCATAATTAGAAACTTGTCAGCAAGTGGATTAACTG : 1960
NM_128456 : ACCAAAAATCATTTCATT------------------------------------------------------------------------------------------------AAACTTGTCAGCAAGTGGATTAACTG :  964
FJ708705  : ACCAAAAATCATTTCATT------------------------------------------------------------------------------------------------AAACTTGTCAGCAAGTGGATTAACTG : 1337
                                                                                                                                                               
                     *      1980         *      2000         *      2020         *      2040         *      2060         *      2080         *      2100       
Genomic   : GGAGTCTACCCTCAGTTTTTCAAAATCTAACGCAGATACAAGAGCTGTAAGTAGTACTTCTATTGTTGACTCATCAAACCAAGAATGATTTGTTCGGACGTGACATGTAAGCGAACCATATGTATTTATATATTGGCGTG : 2100
NM_128456 : GGAGTCTACCCTCAGTTTTTCAAAATCTAACGCAGATACAAGAGCTG--------------------------------------------------------------------------------------------- : 1011
FJ708705  : GGAGTCTACCCTCAGTTTTTCAAAATCTAACGCAGATACAAGAGCTG--------------------------------------------------------------------------------------------- : 1384
                                                                                                                                                               
                     *      2120         *      2140         *      2160         *      2180         *      2200         *      2220         *      2240       
Genomic   : CAGGGACTTATCTAACAACAGTTTGACTGGACTTGTGCCTTCTTTTCTGGCCAACATTAAATCATTATCTTTGTTGTAAGTTTTCTAGAAAGTAGATTGAACATTTTGCTTACGTTTTCGTTGTTGTTGGATTTAGCTAT : 2240
NM_128456 : ----GACTTATCTAACAACAGTTTGACTGGACTTGTGCCTTCTTTTCTGGCCAACATTAAATCATTATCTTTGTT----------------------------------------------------------------- : 1082
FJ708705  : ----GACTTATCTAACAACAGTTTGACTGGACTTGTGCCTTCTTTTCTGGCCAACATTAAATCATTATCTTTGTT----------------------------------------------------------------- : 1455
                                                                                                                                                               
                     *      2260         *      2280         *      2300         *      2320         *      2340         *      2360         *      2380       
Genomic   : ATATATGATGTTTTTTTTTTCTTTTTCTTAACAGAGACTTGAGTGGGAACAATTTTACCGGTTCAGTTCCACAAACTCTTCTTGATAGAGAAAAGGAAGGACTTGTCTTGAAGTAAGATTATTTTCCCCCCATTGTTTAT : 2380
NM_128456 : ----------------------------------AGACTTGAGTGGGAACAATTTTACCGGTTCAGTTCCACAAACTCTTCTTGATAGAGAAAAGGAAGGACTTGTCTTGAA---------------------------- : 1160
FJ708705  : ----------------------------------AGACTTGAGTGGGAACAATTTTACCGGTTCAGTTCCACAAACTCTTCTTGATAGAGAAAAGGAAGGACTTGTCTTGAA---------------------------- : 1533
       
                                                                                                                                                        
                     *      2400         *      2420         *      2440         *      2460         *      2480         *      2500         *      2520       
Genomic   : CAACTAAACATAAATGGTAGTACTTGTATAAAAATTAGATTGCTTATATATGCAAGATCTGGTCGTAGGTGTACTTGAAAGTTCAGATATGTTCCATCCACATCTTTATGGTACATTAATTTAACAATCTTGGTATCACA : 2520
NM_128456 : -------------------------------------------------------------------------------------------------------------------------------------------- :    -
FJ708705  : -------------------------------------------------------------------------------------------------------------------------------------------- :    -
                                                                                                                                                               
                     *      2540         *      2560         *      2580         *      2600         *      2620         *      2640         *      2660       
Genomic   : TGTTCCTTGTAGACTTGAAGGAAATCCAGAGCTATGCAAGTTTAGCTCATGCAATCCAAAAAAGAAAAAGGGATTATTGGTACCGGTTATTGCATCAATTTCCTCTGTGCTTATTGTAATAGTGGTTGTGGCTCTCTTTT : 2660
NM_128456 : ------------ACTTGAAGGAAATCCAGAGCTATGCAAGTTTAGCTCATGCAATCCAAAAAAGAAAAAGGGATTATTGGTACCGGTTATTGCATCAATTTCCTCTGTGCTTATTGTAATAGTGGTTGTGGCTCTCTTTT : 1288
FJ708705  : ------------ACTTGAAGGAAATCCAGAGCTATGCAAGTTTAGCTCATGCAATCCAAAAAAGAAAAAGGGATTATTGGTACCGGTTATTGCATCAATTTCCTCTGTGCTTATTGTAATAGTGGTTGTGGCTCTCTTTT : 1661
                                                                                                                                                               
                     *      2680         *      2700         *      2720         *      2740         *      2760         *      2780         *      2800       
Genomic   : TTGTTCTCCGAAAGAAGAAGATGCCTTCAGGTGAGTCTCGAGGCAAATAACCATTAGAGATGTATTATAACATCAATGTATATAAATCAATCATTGTACTTTGTAGATGCACAGGCTCCACCAAGTTTGCCCGTAGAAGA : 2800
NM_128456 : TTGTTCTCCGAAAGAAGAAGATGCCTTCAG----------------------------------------------------------------------------ATGCACAGGCTCCACCAAGTTTGCCCGTAGAAGA : 1352
FJ708705  : TTGTTCTCCGAAAGAAGAAGATGCCTTCAG----------------------------------------------------------------------------ATGCACAGGCTCCACCAAGTTTGCCCGTAGAAGA : 1725
                                                                                                                                                               
                     *      2820         *      2840         *      2860         *      2880         *      2900         *      2920         *      2940       
Genomic   : TGTTGGACAAGCTAAACATTCAGAATCATCATTCGTCTCGAAAAAGATAAGGTTTGCTTATTTCGAGGTTCAAGAGATGACAAATAACTTTCAGAGAGTTCTTGGTGAAGGAGGCTTTGGAGTCGTTTATCATGGTTGTG : 2940
NM_128456 : TGTTGGACAAGCTAAACATTCAGAATCATCATTCGTCTCGAAAAAGATAAGGTTTGCTTATTTCGAGGTTCAAGAGATGACAAATAACTTTCAGAGAGTTCTTGGTGAAGGAGGCTTTGGAGTCGTTTATCATGGTTGTG : 1492
FJ708705  : TGTTGGACAAGCTAAACATTCAGAATCATCATTCGTCTCGAAAAAGATAAGGTTTGCTTATTTCGAGGTTCAAGAGATGACAAATAACTTTCAGAGAGTTCTTGGTGAAGGAGGCTTTGGAGTCGTTTATCATGGTTGTG : 1865
                                                                                                                                                               
                     *      2960         *      2980         *      3000         *      3020         *      3040         *      3060         *      3080       
Genomic   : TTAATGGTACCCAACAAGTAGCTGTTAAATTGCTCTCTCAATCATCTTCCCAAGGCTATAAACATTTCAAGGCAGAGGTTAGATTTTGATCCGGTGATTGTTGTTGGAATATCCATTTATTATTTACAAGTGCTTTCTAT : 3080
NM_128456 : TTAATGGTACCCAACAAGTAGCTGTTAAATTGCTCTCTCAATCATCTTCCCAAGGCTATAAACATTTCAAGGCAGAGGT------------------------------------------------------------- : 1571
FJ708705  : TTAATGGTACCCAACAAGTAGCTGTTAAATTGCTCTCTCAATCATCTTCCCAAGGCTATAAACATTTCAAGGCAGAGGT------------------------------------------------------------- : 1944
                                                                                                                                                               
                     *      3100         *      3120         *      3140         *      3160         *      3180         *      3200         *      3220       
Genomic   : CTTAAAGAGTATTAGCTAATGGGTGGTTCGTTAGGTGGAACTTCTTATGAGAGTACACCATAAAAATTTGGTGAGTCTTGTTGGTTATTGTGATGAAGGAGACCATTTGGCCCTCATCTACGAGTACATGCCTAATGGAG : 3220
NM_128456 : ------------------------------------GGAACTTCTTATGAGAGTACACCATAAAAATTTGGTGAGTCTTGTTGGTTATTGTGATGAAGGAGACCATTTGGCCCTCATCTACGAGTACATGCCTAATGGAG : 1675
FJ708705  : ------------------------------------GGAACTTCTTATGAGAGTACACCATAAAAATTTGGTGAGTCTTGTTGGTTATTGTGATGAAGGAGACCATTTGGCCCTCATCTACGAGTACATGCCTAATGGAG : 2048
                                                                                                                                                               
                     *      3240         *      3260         *      3280         *      3300         *      3320         *      3340         *      3360       
Genomic   : ACTTAAAACAACATTTATCAGGTATAAGAATATCTTTACCAATTTTTGTTAAAAATAAGAGTTTTGTTTGAAAAAGATCAGAACATTAATTATCTTAATTTTTTGTTGGTTTGTTACAGGAAAGCGTGGTGGATTTGTCT : 3360
NM_128456 : ACTTAAAACAACATTTATCAGG--------------------------------------------------------------------------------------------------AAAGCGTGGTGGATTTGTCT : 1717
FJ708705  : ACTTAAAACAACATTTATCAGG--------------------------------------------------------------------------------------------------AAAGCGTGGTGGATTTGTCT : 2090
                                                                                                                                                               
                     *      3380         *      3400         *      3420         *      3440         *      3460         *      3480         *      3500       
Genomic   : TAAGCTGGGAAAGTAGACTAAGAGTAGCTGTCGATGCAGCACTAGGTACACACTAATTTACTGATAACAATTTAAGACAATTATATAGTTCAGATTGTAACTTTCAATAGTATTAGATTTGCCTCTAGGAATCAGCAAGA : 3500
NM_128456 : TAAGCTGGGAAAGTAGACTAAGAGTAGCTGTCGATGCAGCACTAGGT--------------------------------------------------------------------------------------------- : 1764
FJ708705  : TAAGCTGGGAAAGTAGACTAAGAGTAGCTGTCGATGCAGCACTAGGT--------------------------------------------------------------------------------------------- : 2137
                                                                                                                                                               
                     *      3520         *      3540         *      3560         *      3580         *      3600         *      3620         *      3640       
Genomic   : ACCATATAATATAAAATAATAATTCGGCACTAAAAATTTGTTATGTAGGTTTGGAGTACTTACACACTGGATGCAAACCACCAATGGTTCACAGAGATATAAAAAGTACAAACATACTTTTGGATGAACGTTTCCAAGCC : 3640
NM_128456 : --------------------------------------------------TTGGAGTACTTACACACTGGATGCAAACCACCAATGGTTCACAGAGATATAAAAAGTACAAACATACTTTTGGATGAACGTTTCCAAGCC : 1854
FJ708705  : --------------------------------------------------TTGGAGTACTTACACACTGGATGCAAACCACCAATGGTTCACAGAGATATAAAAAGTACAAACATACTTTTGGATGAACGTTTCCAAGCC : 2227
                                                                                                                                                               
                     *      3660         *      3680         *      3700         *      3720         *      3740         *      3760         *      3780       
Genomic   : AAATTAGCCGATTTTGGGCTTTCGAGATCTTTTCCTACCGAAAACGAAACACATGTTTCAACTGTTGTGGCTGGAACTCCTGGTTATCTTGATCCCGAGTGAGTATTTTCTTCATATTGTATGTTAGTCCATAAACATGC : 3780
NM_128456 : AAATTAGCCGATTTTGGGCTTTCGAGATCTTTTCCTACCGAAAACGAAACACATGTTTCAACTGTTGTGGCTGGAACTCCTGGTTATCTTGATCCCGAGT---------------------------------------- : 1954
FJ708705  : AAATTAGCCGATTTTGGGCTTTCGAGATCTTTTCTTACCGAAAACGAAACACATGTTTCAACTGTTGTGGCTGGAACTCCTGGTTATCTTGATCCCGAGT---------------------------------------- : 2327
                                                                                                                                                               
                     *      3800         *      3820         *      3840         *      3860         *      3880         *      3900         *      3920       
Genomic   : ATCATACCTTTTAATAGTTACTAGCCATAAACCACCTTCAATGTTTTGTTTTGTAGGTATTATCAAACAAATTGGTTGACAGAGAAAAGTGATGTTTACAGTTTCGGAATTGTACTATTGGAGATCATCACAAACCGGCC : 3920
NM_128456 : ----------------------------------------------------------ATTATCAAACAAATTGGTTGACAGAGAAAAGTGATGTTTACAGTTTCGGAATTGTACTATTGGAGATCATCACAAACCGGCC : 2036
FJ708705  : ----------------------------------------------------------ATTATCAAACAAATTGGTTGACAGAGAAAAGTGATGTTTACAGTTTCGGAATTGTACTATTGGAGATCATCACAAACCGGCC : 2409
                                                                                                                                                               
                     *      3940         *      3960         *      3980         *      4000         *      4020         *      4040         *      4060       
Genomic   : TATAATTCAGCAATCTCGTGAAAAGCCTCACCTAGTAGAATGGGTTGGATTTATTGTAAGAACGGGAGATATTGGAAATATTGTTGATCCAAACCTTCACGGAGCTTACGACGTCGGTTCTGTCTGGAAGGCTATTGAAC : 4060
NM_128456 : TATAATTCAGCAATCTCGTGAAAAGCCTCACCTAGTAGAATGGGTTGGATTTATTGTAAGAACGGGAGATATTGGAAATATTGTTGATCCAAACCTTCACGGAGCTTACGACGTCGGTTCTGTCTGGAAGGCTATTGAAC : 2176
FJ708705  : TATAATTCAGCAATCTCGTGAAAAGCCTCACCTAGTAGAATGGGTTGGATTTATTGTAAGAACGGGAGATATTGGAAATATTGTTGATCCAAACCTTCACGGAGCTTACGACGTCGGTTCTGTCTGGAAGGCTATTGAAC : 2549
                                                                                                                                                               
                     *      4080         *      4100         *      4120         *      4140         *      4160         *      4180         *      4200       
Genomic   : TAGCTATGTCATGTGTGAATATTTCTTCGGCAAGAAGACCAAGCATGTCTCAAGTTGTTAGCGATCTTAAAGAGTGTGTGATATCTGAAAATTCAAGGACAGGAGAGAGTCGAGAAATGAACTCAATGAGTTCCATCGAA : 4200
NM_128456 : TAGCTATGTCATGTGTGAATATTTCTTCGGCAAGAAGACCAAGCATGTCTCAAGTTGTTAGCGATCTTAAAGAGTGTGTGATATCTGAAAATTCAAGGACAGGAGAGAGTCGAGAAATGAACTCAATGAGTTCCATCGAA : 2316
FJ708705  : TAGCTATGTCATGTGTGAATATTTCTTCGGCAAGAAGACCAAGCATGTCTCAAGTTGTTAGCGATCTTAAAGAGTGTGTGATATCTGAAAATTCAAGGACAGGAGAGAGTCGAGAAATGAACTCAATGAGTTCCATCGAA : 2689
                                                             
                     *      4220         *      4240         
Genomic   : TTCAGCATGGGAATTGACACCGAGGTGATCCCTAAAGCACGC : 4242
NM_128456 : TTCAGCATGGGAATTGACACCGAGGTGATCCCTAAAGCACGC : 2358
FJ708705  : TTCAGCATGGGAATTGACACCGAGGTGATCCCTAAAGCACGC : 2731


At3g46370
                                                                                                                                                               
                     *        20         *        40         *        60         *        80         *       100         *       120         *       140       
Genomic   : ATGCGAAACTGTTACAATCTTAGTGTGCATAAAGAAACAAAGTATTTGATTAGGGTTACAAGTAACTATGGAAATTACGATGGTCGTAACGAACCTCCTAGATTTGATTTGTACTTAGGCCCTAACTTCTGGGTAACAAT :  140
NM_114504 : ATGCGAAACTGTTACAATCTTAGTGTGCATAAAGAAACAAAGTATTTGATTAGGGTTACAAGTAACTATGGAAATTACGATGGTCGTAACGAACCTCCTAGATTTGATTTGTACTTAGGCCCTAACTTCTGGGTAACAAT :  140
FJ708733  : ATGCGAAACTGTTACAATCTTAGTGTGCATAAAGAAACAAAGTATTTGATTAGGGTTACAAGTAACTATGGAAATTACGATGGTCGTAACGAACCTCCTAGATTTGATTTGTACTTAGGCCCTAACTTCTGGGTAACAAT :  140
                                                                                                                                                               
                     *       160         *       180         *       200         *       220         *       240         *       260         *       280       
Genomic   : AGATTTGGGGAAGCATGTAAATGGTGATACATGGAAGGAGATTATTCATATCCCAAAGTCAAACTCTCTAGATGTGTGTCTTATCAAAACAGGTACAACAACGCCAATTATATCGACCTTGGAACTAAGGTCTCTACCAA :  280
NM_114504 : AGATTTGGGGAAGCATGTAAATGGTGATACATGGAAGGAGATTATTCATATCCCAAAGTCAAACTCTCTAGATGTGTGTCTTATCAAAACAGGTACAACAACGCCAATTATATCGACCTTGGAACTAAGGTCTCTACCAA :  280
FJ708733  : AGATTTGGGGAAGCATGTAAATGGTGATACATGGAAGGAGATTATTCATATCCCAAAGTCAAACTCTCTAGATGTGTGTCTTATCAAAACAGGTACAACAACGCCAATTATATCGACCTTGGAACTAAGGTCTCTACCAA :  280
                                                                                                                                                               
                     *       300         *       320         *       340         *       360         *       380         *       400         *       420       
Genomic   : AGTATAGTTACAACGCAATATCAGGTTCTTTGAAGTCGACACTGAGGGCATTCCTAAGCGAATCAACAGAAGTTATAAGGTACGTCACCATTAAATTGATATTAAGGTTTTTTGACTCGTTCCATCGTCCATTGTTAATA :  420
NM_114504 : AGTATAGTTACAACGCAATATCAGGTTCTTTGAAGTCGACACTGAGGGCATTCCTAAGCGAATCAACAGAAGTTATAAG------------------------------------------------------------- :  359
FJ708733  : AGTATAGTTACAACGCAATATCAGGTTCTTTGAAGTCGACACTGAGGGCATTCCTAAGCGAATCAACAGAAGTTATAAG------------------------------------------------------------- :  359
                                                                                                                                                               
                     *       440         *       460         *       480         *       500         *       520         *       540         *       560       
Genomic   : ATACAAGATTGTGTTGAAGCTCAGGTACCCAAATGATTTCTACGATCGGATGTGGGTTCCACATTTTGAGACGGAATGGAAGCAAATTTCTACCAATCTCAAAGTGAACAGCTCCAATGGTTATCTTCTGCCACAGGATG :  560
NM_114504 : ------------------------GTACCCAAATGATTTCTACGATCGGATGTGGGTTCCACATTTTGAGACGGAATGGAAGCAAATTTCTACCAATCTCAAAGTGAACAGCTCCAATGGTTATCTTCTGCCACAGGATG :  475
FJ708733  : ------------------------GTACCCAAATGATTTCTACGATCGGATGTGGGTTCCACATTTTGAGACGGAATGGAAGCAAATTTCTACCAATCTCAAAGTGAACAGCTCCAATGGTTATCTTCTGCCACAGGATG :  475
                                                                                                                                                               
                     *       580         *       600         *       620         *       640         *       660         *       680         *       700       
Genomic   : TGCTCATGACCGCCGCAATACCTGTGAATACTAGTGCACGATTGAGTTTTACCGAGAATCTTGAGTTCCCTCATGATGAACTTTACCTGTACTTCCACTTCTCTGAGGTCCAAGTCTTACAGGCCAACCAGAGTAGAGAG :  700
NM_114504 : TGCTCATGACCGCCGCAATACCTGTGAATACTAGTGCACGATTGAGTTTTACCGAGAATCTTGAGTTCCCTCATGATGAACTTTACCTGTACTTCCACTTCTCTGAGGTCCAAGTCTTACAGGCCAACCAGAGTAGAGAG :  615
FJ708733  : TGCTCATGACCGCCGCAATACCTGTGAATACTAGTGCACGATTGAGTTTTACCGAGAATCTTGAGTTCCCTCATGATGAACTTTACCTGTACTTCCACTTCTCTGAGGTCCAAGTCTTACAGGCCAACCAGAGTAGAGAG :  615
                                                                                                                                                               
                     *       720         *       740         *       760         *       780         *       800         *       820         *       840       
Genomic   : TTCAGCATTTTGTGGAACGGAATGGTTATTTACCCAGATTTTATCCCTGACTATCTTGGTGCCGCCACTGTGTACAACCCGTCACCATCTCTTTGTGAAGTAGGGAAATGCTTATTAGAGCTTGAAAGAACTCAGAAATC :  840
NM_114504 : TTCAGCATTTTGTGGAACGGAATGGTTATTTACCCAGATTTTATCCCTGACTATCTTGGTGCCGCCACTGTGTACAACCCGTCACCATCTCTTTGTGAAGTAGGGAAATGCTTATTAGAGCTTGAAAGAACTCAGAAATC :  755
FJ708733  : TTCAGCATTTTGTGGAACGGAATGGTTATTTACCCAGATTTTATCCCTGACTATCTTGGTGCCGCCACTGTGTACAACCCGTCACCATCTCTTTGTGAAGTAGGGAAATGCTTATTAGAGCTTGAAAGAACTCAGAAATC :  755
                                                                                                                                                               
                     *       860         *       880         *       900         *       920         *       940         *       960         *       980       
Genomic   : AACTCTCCCGCCTCTCCTTAACGCCATTGAAGTTTTCACAGTGATGAATTTTCCACAGTCTGAGACAAACGATGATGATGGTACTTTTTTTCTTCTTCTTAATGGTAGTTAAGTATTGTATGGGGAATGTGGTCTAAATC :  980
NM_114504 : AACTCTCCCGCCTCTCCTTAACGCCATTGAAGTTTTCACAGTGATGAATTTTCCACAGTCTGAGACAAACGATGATGATG------------------------------------------------------------ :  835
FJ708733  : AACTCTCCCGCCTCTCCTTAACGCCATTGAAGTTTTCACAGTGATGAATTTTCCACAGTCTGAGACAAACGATGATGATG------------------------------------------------------------ :  835
                                                                                                                                                               
                     *      1000         *      1020         *      1040         *      1060         *      1080         *      1100         *      1120       
Genomic   : CAATTAACCTGTTTGTCGGGAAATTTTATGACAGTAATTGCTATAACAAAGATCAAAGACACACACAGATTGAATAGAACCTCCTGGCAAGGAGATCCATGCGTCCCTCAACTGTTTTCGTGGGCCGGTCTAAGTTGCAT : 1120
NM_114504 : ----------------------------------TAATTGCTATAACAAAGATCAAAGACACACACAGATTGAATAGAACCTCCTGGCAAGGAGATCCATGCGTCCCTCAACTGTTTTCGTGGGCCGGTCTAAGTTGCAT :  941
FJ708733  : ----------------------------------TAATTGCTATAACAAAGATCAAAGACACACACAGATTGAATAGAACCTCCTGGCAAGGAGATCCATGCGTCCCTCAACTGTTTTCGTGGGCCGGTCTAAGTTGCAT :  941
                                                                                                                                                               
                     *      1140         *      1160         *      1180         *      1200         *      1220         *      1240         *      1260       
Genomic   : TGACACGAATGTATCTACCCCACCAAGAATCATTTCCCTGTAAGACCTCTATAACACAATCACTCTTTAGACTTGTGAGAAAAACTTGTCTAACTAAACTTATACTTGTTTATTTTTCTTCTTGTTTGGACTTATACACA : 1260
NM_114504 : TGACACGAATGTATCTACCCCACCAAGAATCATTTCCCT----------------------------------------------------------------------------------------------------- :  980
FJ708733  : TGACACGAATGTATCTACCCCACCAAGAATCATTTCCCT----------------------------------------------------------------------------------------------------- :  980
                                                                                                                                                               
                     *      1280         *      1300         *      1320         *      1340         *      1360         *      1380         *      1400       
Genomic   : GAAACTTGTCTTCAAGTGGGTTAACAGGAAACATAGCAACTGGAATTCAAAATCTGACCAAACTGCAAAAGCTGTAAGTATGACCATTTAAAGAAAAATGTTATATACGTCCGTTACCTTTCTTATTGATTTCTTACAAA : 1400
NM_114504 : -AAACTTGTCTTCAAGTGGGTTAACAGGAAACATAGCAACTGGAATTCAAAATCTGACCAAACTGCAAAAGCTG------------------------------------------------------------------ : 1053
FJ708733  : -AAACTTGTCTTCAAGTGGGTTAACAGGAAACATAGCAACTGGAATTCAAAATCTGACCAAACTGCAAAAGCTG------------------------------------------------------------------ : 1053
                                                                                                                                                               
                     *      1420         *      1440         *      1460         *      1480         *      1500         *      1520         *      1540       
Genomic   : CTTCAGGGACTTGTCAAATAACAATTTGACAGGAGTAGTGCCCGAATTTCTAGCTAACATGAAATCGTTGCTGTTCATGTAAGTTTCCCAAAAGGTTTTGGTGGTTTTTTTCCCATGGATCATCTTCACCTAAGTATGTA : 1540
NM_114504 : -------GACTTGTCAAATAACAATTTGACAGGAGTAGTGCCCGAATTTCTAGCTAACATGAAATCGTTGCTGTTCAT-------------------------------------------------------------- : 1124
FJ708733  : -------GACTTGTCAAATAACAATTTGACAGGAGTAGTGCCCGAATTTCTAGCTAACATGAAATCGTTGCTGTTCAT-------------------------------------------------------------- : 1124
                                                                                                                                                               
                     *      1560         *      1580         *      1600         *      1620         *      1640         *      1660         *      1680       
Genomic   : GATTTCTCTGGTTTTAACAGAGACTTGAGGAAGAACAAACTGAATGGTTCAATACCAAAGACTCTACTTGATAGAAAAAAGAAAGGACTACAATTATTGTAAGAAGGAAATTCATCCACGCACATTTTAATTCAAGATCC : 1680
NM_114504 : --------------------AGACTTGAGGAAGAACAAACTGAATGGTTCAATACCAAAGACTCTACTTGATAGAAAAAAGAAAGGACTACAATTATT------------------------------------------ : 1202
FJ708733  : --------------------AGACTTGAGGAAGAACAAACTGAATGGTTCAATACCAAAGACTCTACTTGATAGAAAAAAGAAAGGACTACAATTATT------------------------------------------ : 1202
                                                                                                                                                               
                     *      1700         *      1720         *      1740         *      1760         *      1780         *      1800         *      1820       
Genomic   : ATTGCCTTGTTAAATTCATCAAAACACTAAAAGATCGATATATTATTTTTGTAGTGTTGATGGCGATGATGATAAGGGCGATGATAATAAGTGTTTATCCGGTTCATGTGTCCCAAAAATGAAATTCCCATTGATGATTG : 1820
NM_114504 : ------------------------------------------------------TGTTGATGGCGATGATGATAAGGGCGATGATAATAAGTGTTTATCCGGTTCATGTGTCCCAAAAATGAAATTCCCATTGATGATTG : 1288
FJ708733  : ------------------------------------------------------TGTTGATGGCGATGATGATAAGGGCGATGATAATAAGTGTTTATCCGGTTCATGTGTCCCAAAAATGAAATTCCCATTGATGATTG : 1288
                                                                                                                                                               
                     *      1840         *      1860         *      1880         *      1900         *      1920         *      1940         *      1960       
Genomic   : TTGCATTAGCGGTTTCTGCGGTTGTGGTCATTGCAGTGGTAATGATTCTCATTTTTCTGTTCAGAAAGAAAAAGAAATCAAGTTTAGGTATTTGCATAGACTCACCAAATATTAACTAACAAACTATACAATATAAATTA : 1960
NM_114504 : TTGCATTAGCGGTTTCTGCGGTTGTGGTCATTGCAGTGGTAATGATTCTCATTTTTCTGTTCAGAAAGAAAAAGAAATCAAGTTTAGGTA-------------------------------------------------- : 1378
FJ708733  : TTGCATTAGCGGTTTCTGCGGTTGTGGTCATTGCAGTGGTA--------------------------------------------------------------------------------------------------- : 1329
                                                                                                                                                               
                     *      1980         *      2000         *      2020         *      2040         *      2060         *      2080         *      2100       
Genomic   : AAATGTCACTGATATTGTTGTTTCTCTACATATATCTATCCCCTTTTCTTGTTATGCCACAAATAGGTATTACGTCAGCTGCTATATCTGAAGAATCGATTGAGACGAAAAGGAGAAGGTTTACCTATTCTGAAGTTGTG : 2100
NM_114504 : ---------------------------------------------------------------------TTACGTCAGCTGCTATATCTGAAGAATCGATTGAGACGAAAAGGAGAAGGTTTACCTATTCTGAAGTTGTG : 1449
FJ708733  : ---------------------------------------------------------------------TTACGTCAGCTGCTATATCTGAAGAATCGATTGAGACGAAAAGGAGAAGGTTTACCTATTCTGAAGTTGTG : 1400
                                                                                                                                                               
                     *      2120         *      2140         *      2160         *      2180         *      2200         *      2220         *      2240       
Genomic   : GAAATGACAAAAAACTTCCAAAAAACTCTAGGTGAAGGAGGGTTCGGAACCGTGTATTATGGTAATTTGAATGGTTCAGAGCAAGTAGCGGTTAAAGTACTCTCGCAATCATCATCACAGGGCTATAAACACTTCAAGGC : 2240
NM_114504 : GAAATGACAAAAAACTTCCAAAAAACTCTAGGTGAAGGAGGGTTCGGAACCGTGTATTATGGTAATTTGAATGGTTCAGAGCAAGTAGCGGTTAAAGTACTCTCGCAATCATCATCACAGGGCTATAAACACTTCAAGGC : 1589
FJ708733  : GAAATGACAAAAAACTTCCAAAAAACTCTAGGTGAAGGAGGGTTCGGAACCGTGTATTATGGTAATTTGAATGGTTCAGAGCAAGTAGCGGTTAAAGTACTCTCGCAATCATCATCACAGGGCTATAAACACTTCAAGGC : 1540
                                                                                                                                                               
                     *      2260         *      2280         *      2300         *      2320         *      2340         *      2360         *      2380       
Genomic   : AGAGGTATGATATGCCATAAATCGTCTATAGAATCGCACAAAATTTAAAAGAAATCATGATCTATATAGATATATATTTAGCATCACGTTAAGCAAACATATTTTGTCATTGTTGGTCTATAGGTCGAACTTCTTTTAAG : 2380
NM_114504 : AGAGGT-----------------------------------------------------------------------------------------------------------------------CGAACTTCTTTTAAG : 1610
FJ708733  : AGAGGT-----------------------------------------------------------------------------------------------------------------------CGAACTTCTTTTAAG : 1561
                                                                                                                                                               

                     *      2400         *      2420         *      2440         *      2460         *      2480         *      2500         *      2520       
Genomic   : AGTTCACCACATAAATTTGGTGAGTCTTGTTGGATATTGCGATGAAAGAAATCACTTGGCCCTCATCTACGAATGCATGTCCAATGGAGACTTAAAAGATCATTTGTCAGGTGAAAGATGTATATATGCAAATATAAATA : 2520
NM_114504 : AGTTCACCACATAAATTTGGTGAGTCTTGTTGGATATTGCGATGAAAGAAATCACTTGGCCCTCATCTACGAATGCATGTCCAATGGAGACTTAAAAGATCATTTGTCAGG----------------------------- : 1721
FJ708733  : AGTTCACCACATAAATTTGGTGAGTCTTGTTGGATATTGCGATGAAAGAAATCACTTGGCCCTCATCTACGAATGCATGTCCAATGGAGACTTAAAAGATCATTTGTCAGG----------------------------- : 1672
                                                                                                                                                               
                     *      2540         *      2560         *      2580         *      2600         *      2620         *      2640         *      2660       
Genomic   : TATATATATATATATATATATATATATATATATATATATATTATATAAAATTCATAGGACGGTTAATTTTGACAATCTAATACAGGAAAAAAGGGTAACGCTGTTTTGAAATGGAGTACTAGACTACGAATAGCTGTCGA : 2660
NM_114504 : --------------------------------------------------------------------------------------AAAAAAGGGTAACGCTGTTTTGAAATGGAGTACTAGACTACGAATAGCTGTCGA : 1775
FJ708733  : --------------------------------------------------------------------------------------AAAAAAGGGTAACGCTGTTTTGAAATGGAGTACTAGACTACGAATAGCTGTCGA : 1726
                                                                                                                                                               
                     *      2680         *      2700         *      2720         *      2740         *      2760         *      2780         *      2800       
Genomic   : TGCTGCGCTAGGTTCGTACTAACTCAAAACAGTTCCAGTGACTTGTCATTTTATATCAATTAGTTACTAATTGTATGCATTTGATACATCTGTTACAAACTTCTCATGATGAATTATTTCCATCAATTTTTACTCACTGG : 2800
NM_114504 : TGCTGCGCTAGG-------------------------------------------------------------------------------------------------------------------------------- : 1787
FJ708733  : TGCTGCGCTAGG-------------------------------------------------------------------------------------------------------------------------------- : 1738
                                                                                                                                                               
                     *      2820         *      2840         *      2860         *      2880         *      2900         *      2920         *      2940       
Genomic   : AATTTCTGACATGATCAACTAACAACATGAATGAGTCTCCTGCACAGGATTGGAATACTTGCATTATGGATGTCGACCATCGATAGTGCATAGAGATGTCAAAAGTACCAATATACTGCTGGATGATCAGCTCATGGCCA : 2940
NM_114504 : ------------------------------------------------ATTGGAATACTTGCATTATGGATGTCGACCATCGATAGTGCATAGAGATGTCAAAAGTACCAATATACTGCTGGATGATCAGCTCATGGCCA : 1879
FJ708733  : ------------------------------------------------ATTGGAATACTTGCATTATGGATGTCGACCATCGATAGTGCATAGAGATGTCAAAAGTACCAATATACTGCTGGATGATCAGCTCATGGCCA : 1830
                                                                                                                                                               
                     *      2960         *      2980         *      3000         *      3020         *      3040         *      3060         *      3080       
Genomic   : AAATTGCTGATTTTGGGCTTTCAAGATCATTCAAACTCGGAGAAGAATCACAGGCTTCCACGGTTGTTGCTGGTACTCTTGGATACCTTGATCCCGAGTAAGTACTATTAGTTCACTCCCATGATAAATATGTGTGTAAT : 3080
NM_114504 : AAATTGCTGATTTTGGGCTTTCAAGATCATTCAAACTCGGAGAAGAATCACAGGCTTCCACGGTTGTTGCTGGTACTCTTGGATACCTTGATCCCGA------------------------------------------- : 1976
FJ708733  : AAATTGCTGATTTTGGGCTTTCAAGATCATTCAAACTCGGAGAAGAATCACAGGCTTCCACGGTTGTTGCTGGTACTCTTGGATACCTTGATCCCGA------------------------------------------- : 1927
                                                                                                                                                               
                     *      3100         *      3120         *      3140         *      3160         *      3180         *      3200         *      3220       
Genomic   : TTATGTCTACATTCATCTTCATCGTTTATATAGAGGTATACATATAAATCCTACTTCATCTCTTTGTTTTCCAGATATTATAGAACATGTCGGTTGGCTGAGATGAGTGATGTATACAGTTTCGGTATTTTACTACTAGA : 3220
NM_114504 : --------------------------------------------------------------------------ATATTATAGAACATGTCGGTTGGCTGAGATGAGTGATGTATACAGTTTCGGTATTTTACTACTAGA : 2042
FJ708733  : --------------------------------------------------------------------------ATATTATAGAACATGTCGGTTGGCTGAGATGAGTGATGTATACAGTTTCGGTATTTTACTACTAGA : 1993
                                                                                                                                                               
                     *      3240         *      3260         *      3280         *      3300         *      3320         *      3340         *      3360       
Genomic   : GATAATCACAAACCAAAATGTGATTGACCATGCCCGCGAAAAGGCTCACATTACAGAATGGGTAGGACTTGTGCTTAAGGGAGGAGATGTTACTAGGATTGTTGATCCTAACCTTGACGGTGAATATAACTCTCGTTCAG : 3360
NM_114504 : GATAATCACAAACCAAAATGTGATTGACCATGCCCGCGAAAAGGCTCACATTACAGAATGGGTAGGACTTGTGCTTAAGGGAGGAGATGTTACTAGGATTGTTGATCCTAACCTTGACGGTGAATATAACTCTCGTTCAG : 2182
FJ708733  : GATAATCACAAACCAAAATGTGATTGACCATGCCCGCGAAAAGGCTCACATTACAGAATGGGTAGGACTTGTGCTTAAGGGAGGAGATGTTACTAGGATTGTTGATCCTAACCTTGACGGTGAATATAACTCTCGTTCAG : 2133
                                                                                                                                                               
                     *      3380         *      3400         *      3420         *      3440         *      3460         *      3480         *      3500       
Genomic   : TTTGGAGAGCTCTTGAATTGGCTATGTCATGCGCCAACCCTTCTTCAGAACATCGACCAATCATGTCCCAGGTTGTTATCGATCTAAAAGAGTGTTTAAATACTGAAAACTCAATGAAAATAAAGAAAAATGACACGGAC : 3500
NM_114504 : TTTGGAGAGCTCTTGAATTGGCTATGTCATGCGCCAACCCTTCTTCAGAACATCGACCAATCATGTCCCAGGTTGTTATCGATCTAAAAGAGTGTTTAAATACTGAAAACTCAATGAAAATAAAGAAAAATGACACGGAC : 2322
FJ708733  : TTTGGAGAGCTCTTGAATTGGCTATGTCATGCGCCAACCCTTCTTCAGAACATCGACCAATCATGTCCCAGGTTGTTATCGATCTAAAAGAGTGTTTAAATACTGAAAACTCAATGAAAATAAAGAAAAATGACACGGAC : 2273
                                                                            
                     *      3520         *      3540         *              
Genomic   : AATGATGGTTCTCTGGAACTGAGCTCGAGTGATACTGAGGCGGTCCCTTGTGCAAGG : 3557
NM_114504 : AATGATGGTTCTCTGGAACTGAGCTCGAGTGATACTGAGGCGGTCCCTTGTGCAAGG : 2379
FJ708733  : AATGATGGTTCTCTGGAACTGAGCTCGAGTGATACTGAGGCGGTCCCTTGTGCAAGG : 2330


At4g29990
                                                                                                                                                               
                     *        20         *        40         *        60         *        80         *       100         *       120         *       140       
Genomic   : ATGACACGTCTACGCCTTCTTTCATGGATTTCAATCACGAGCTGTGTTTGTCTCGTTTTTGCTCAAGACCAATCAGGTAAATTGGTACTCAGATGTCTCTGCTTTTCTAAACACTTCTATATGTTGGTTATTGCTAAGGT :  140
NM_119145 : ATGACACGTCTACGCCTTCTTTCATGGATTTCAATCACGAGCTGTGTTTGTCTCGTTTTTGCTCAAGACCAATCAGGT-------------------------------------------------------------- :   78
X97774    : ATGACACGTCTACGCCTTCTTTCATGGATTTCAATCACGAGCTGTGTTTGTCTCGTTTTTGCTCAAGACCAATCAGGT-------------------------------------------------------------- :   78
FJ708759  : ATGACACGTCTACGCCTTCTTTCATGGATTTCAATCACGAGCTGTGTTTGTCTCGTTTTTGCTCAAGACCAATCAGGT-------------------------------------------------------------- :   78
                                                                                                                                                               
                     *       160         *       180         *       200         *       220         *       240         *       260         *       280       
Genomic   : TTGTGATTTCAATACAGGTTTTATTAGTATCGATTGTGGGATACCAGATGATTCAAGCTACACTGATGAGAAAACAAACATGAAATACGTTTCGGATTTGGGTTTTGTTGAGTCTGGAACAAGTCATAGCATAGTCTCTG :  280
NM_119145 : -------------------TTTATTAGTATCGATTGTGGGATACCAGATGATTCAAGCTACACTGATGAGAAAACAAACATGAAATACGTTTCGGATTTGGGTTTTGTTGAGTCTGGAACAAGTCATAGCATAGTCTCTG :  199
X97774    : -------------------TTTATTAGTATCGATTGTGGGATACCAGATGATTCAAGCTACACTGATGAGAAAACAAACATGAAATACGTTTCGGATTTGGGTTTTGTTGAGTCTGGAACAAGTCATAGCATAGTCTCTG :  199
FJ708759  : -------------------TTTATTAGTATCGATTGTGGGATACCAGATGATTCAAGCTACACTGATGAGAAAACAAACATGAAATACGTTTCGGATTTGGGTTTTGTTGAGTCTGGAACAAGTCATAGCATAGTCTCTG :  199
                                                                                                                                                               
                     *       300         *       320         *       340         *       360         *       380         *       400         *       420       
Genomic   : ACCTTCAAACAACTTCTCTTGAAAGGCAGTTCCAAAACGTAAGAAGCTTCCCTGAAGGTAAGAGAAACTGTTACGACATAAGGCCTCAGCAAGGGAAAGGTTTCAAGTATCTGATTAGAACTCGTTTTATGTACGGGAAC :  420
NM_119145 : ACCTTCAAACAACTTCTCTTGAAAGGCAGTTCCAAAACGTAAGAAGCTTCCCTGAAGGTAAGAGAAACTGTTACGACATAAGGCCTCAGCAAGGGAAAGGTTTCAAGTATCTGATTAGAACTCGTTTTATGTACGGGAAC :  339
X97774    : ACCTTCAAACAACTTCTCTTGAAAGGCAGTTCCAAAACGTAAGAAGCTTCCCTGAAGGTAAGAGAAACTGTTACGACATAAGGCCTCAGCAAGGGAAAGGTTTCAAGTATCTGATTAGAACTCGTTTTATGTACGGGAAC :  339
FJ708759  : ACCTTCAAACAACTTCTCTTGAAAGGCAGTTCCAAAACGTAAGAAGCTTCCCTGAAG----------------------------------------------------------------------------------- :  256
                                                                                                                                                               
                     *       440         *       460         *       480         *       500         *       520         *       540         *       560       
Genomic   : TATGATGGTTTTAGTAAAACACCTGAGTTTGATCTCTATATTGGAGCCAATCTTTGGGAATCTGTTGTTCTTATCAATGAAACGGCTATAATGACCAAAGAAATCATTTATACACCTCCATCAGACCATATTCATGTGTG :  560
NM_119145 : TATGATGGTTTTAGTAAAACACCTGAGTTTGATCTCTATATTGGAGCCAATCTTTGGGAATCTGTTGTTCTTATCAATGAAACGGCTATAATGACCAAAGAAATCATTTATACACCTCCATCAGACCATATTCATGTGTG :  479
X97774    : TATGATGGTTTTAGTAAAACACCTGAGTTTGATCTCTATATTGGAGCCAATCTTTGGGAATCTGTTGTTCTTATCAATGAAACGGCTATAATGACCAAAGAAATCATTTATACACCTCCATCAGACCATATTCATGTGTG :  479
FJ708759  : -------------------------------------------------------------------------------------------------------------------------------------------- :    -
                                                                                                                                                               
                     *       580         *       600         *       620         *       640         *       660         *       680         *       700       
Genomic   : TCTTGTTGATAAAAACAGAGGAACTCCTTTTCTCTCTGTCTTGGAAATAAGATTTTTGAAGAACGATACATATGACACTCCTTATGAAGCGCTTATGCTTGGTCGAAGATGGGATTTCGGCACAGCGACCAATCTTCAAA :  700
NM_119145 : TCTTGTTGATAAAAACAGAGGAACTCCTTTTCTCTCTGTCTTGGAAATAAGATTTTTGAAGAACGATACATATGACACTCCTTATGAAGCGCTTATGCTTGGTCGAAGATGGGATTTCGGCACAGCGACCAATCTTCAAA :  619
X97774    : TCTTGTTGATAAAAACAGAGGAACTCCTTTTCTCTCTGTCTTGGAAATAAGATTTTTGAAGAACGATACATATGACACTCCTTATGAAGCGCTTATGCTTGGTCGAAGATGGGATTTCGGCACAGCGACCAATCTTCAAA :  619
FJ708759  : ------------------AGGAACTCCTTTTCTCTCTGTCTTGGAAATAAGATTTTTGAAGAACGATACATATGACACTCCTTATGAAGCGCTTATGCTTGGTCGAAGATGGGATTTCGGCACAGCGACCAATCTTCAAA :  378
                                                                                                                                                               
                     *       720         *       740         *       760         *       780         *       800         *       820         *       840       
Genomic   : TCAGGTGAAAACATGTCTTATTCTTTTGTTGTTATATTCTCCCTGCTAAATGTAATCGTTTTGTAGGTACAAAGATGATTTCTATGATCGCATATGGATGCCTTATAAGTCTCCGTACCAGAAAACTTTGAATACATCCC :  840
NM_119145 : TCAGGT--------------------------------------------------------------ACAAAGATGATTTCTATGATCGCATATGGATGCCTTATAAGTCTCCGTACCAGAAAACTTTGAATACATCCC :  697
X97774    : TCAGGT--------------------------------------------------------------ACAAAGATGATTTCTATGATCGCATATGGATGCCTTATAAGTCTCCGTACCAGAAAACTTTGAATACATCCC :  697
FJ708759  : TCAGGT--------------------------------------------------------------ACAAAGATGATTTCTATGATCGCATATGGATGCCTTATAAGTCTCCGTACCAGAAAACTTTGAATACATCCC :  456
                                                                                                                                                               
                     *       860         *       880         *       900         *       920         *       940         *       960         *       980       
Genomic   : TCACCATCGATGAAACTAACCACAACGGTTTCAGACCTGCTAGTATAGTCATGAGATCTGCGATAGCACCTGGAAACGAAAGCAATCCCTTGAAGTTTAACTGGGCACCAGATGATCCGAGGTCGAAGTTTTATATATAC :  980
NM_119145 : TCACCATCGATGAAACTAACCACAACGGTTTCAGACCTGCTAGTATAGTCATGAGATCTGCGATAGCACCTGGAAACGAAAGCAATCCCTTGAAGTTTAACTGGGCACCAGATGATCCGAGGTCGAAGTTTTATATATAC :  837
X97774    : TCACCATCGATGAAACTAACCACAACGGTTTCAGACCTGCTAGTATAGTCATGAGATCTGCGATAGCACCTGGAAACGAAAGCAATCCCTTGAAGTTTAACTGGGCACCAGATGATCCGAGGTCGAAGTTTTATATATAC :  837
FJ708759  : TCACCATCGATGAAACTAACCACAACGGTTTCAGACCTGCTAGTATAGTCATGAGATCTGCGATAGCACCTGGAAACGAAAGCAATCCCTTGAAGTTTAACTGGGCACCAGATGATCCGAGGTCGAAGTTTTATATATAC :  596
                                                                                                                                                               
                     *      1000         *      1020         *      1040         *      1060         *      1080         *      1100         *      1120       
Genomic   : ATGCACTTCGCTGAAGTTCGAGAGCTACAAAGAAACGAGACACGAGAATTCGATATATACATAAATGACGTTATACTTGCCGAGAATTTCCGTCCTTTTTATTTGTTTACAGACACACGCTCCACTGTAGATCCAGTTGG : 1120
NM_119145 : ATGCACTTCGCTGAAGTTCGAGAGCTACAAAGAAACGAGACACGAGAATTCGATATATACATAAATGACGTTATACTTGCCGAGAATTTCCGTCCTTTTTATTTGTTTACAGACACACGCTCCACTGTAGATCCAGTTGG :  977
X97774    : ATGCACTTCGCTGAAGTTCGAGAGCTACAAAGAAACGAGACACGAGAATTCGATATATACATAAATGACGTTATACTTGCCGAGAATTTCCGTCCTTTTTATTTGTTTACAGACACACGCTCCACTGTAGATCCAGTTGG :  977
FJ708759  : ATGCACTTCGCTGAAGTTCGAGAGCTACAAAGAAACGAGACACGAGAATTCGATATATACATAAATGACGTTATACTTGCCGAGAATTTCCGTCCTTTTTATTTGTTTACAGACACACGCTCCACTGTAGATCCAGTTGG :  736
                                                                                                                                                               
                     *      1140         *      1160         *      1180         *      1200         *      1220         *      1240         *      1260       
Genomic   : CAGAAAAATGAATGAAATTGTCCTTCAGAGAACTGGTGTATCGACCCTTCCACCGATCATCAACGCTATAGAGATCTATCAAATCAACGAGTTCCTTCAGTTACCAACAGATCAACAGGATGGTAAGGCTTTCTGACTCA : 1260
NM_119145 : CAGAAAAATGAATGAAATTGTCCTTCAGAGAACTGGTGTATCGACCCTTCCACCGATCATCAACGCTATAGAGATCTATCAAATCAACGAGTTCCTTCAGTTACCAACAGATCAACAGGATG------------------ : 1099
X97774    : CAGAAAAATGAATGAAATTGTCCTTCAGAGAACTGGTGTATCGACCCTTCCACCGATCATCAACGCTATAGAGATCTATCAAATCAACGAGTTCCTTCAGTTACCAACAGATCAACAGGATG------------------ : 1099
FJ708759  : CAGAAAAATGAATGAAATTGTCCTTCAGAGAACTGGTGTATCGACCCTTCCACCGATCATCAACGCTATAGAGATCTATCAAATCAACGAGTTCCTTCAGTTACCAACAGATCAACGGGATG------------------ :  858
                                                                                                                                                               
                     *      1280         *      1300         *      1320         *      1340         *      1360         *      1380         *      1400       
Genomic   : AAAGATTTCCTCTTCTGTACTTTATTTGATTTTGTTTTATCTTGTCTTAGTTGATGCCATGACGAAGATTAAGTTCAAGTATAGAGTAAAGAAGAACTGGCAAGGAGACCCATGTGTTCCAGTGGACAATTCTTGGGAAG : 1400
NM_119145 : --------------------------------------------------TTGATGCCATGACGAAGATTAAGTTCAAGTATAGAGTAAAGAAGAACTGGCAAGGAGACCCATGTGTTCCAGTGGACAATTCTTGGGAAG : 1189
X97774    : --------------------------------------------------TTGATGCCATGACGAAGATTAAGTTCAAGTATAGAGTAAAGAAGAACTGGCAAGGAGACCCATGTGTTCCAGTGGACAATTCTTGGGAAG : 1189
FJ708759  : --------------------------------------------------TTGATGCCATGACGAAGATTAAGTTCAAGTATAGAGTAAAGAAGAACTGGCAAGGAGACCCATGTGTTCCAGTGGACAATTCTTGGGAAG :  948
                                                                                                                                                               
                     *      1420         *      1440         *      1460         *      1480         *      1500         *      1520         *      1540       
Genomic   : GTCTTGAATGTCTCCATAGTGATAATAATACTTCTCCAAAATCCATTGCTCTGTAAGAATTTTTTCAGTTCACAACTGCTCTGCTTTTCTCTGTTCTGCTCTTGCTGTTACATGTATCTCAAACAACGTTGTGATTCGCA : 1540
NM_119145 : GTCTTGAATGTCTCCATAGTGATAATAATACTTCTCCAAAATCCATTGCTCTG--------------------------------------------------------------------------------------- : 1242
X97774    : GTCTTGAATGTCTCCATAGTGATAATAATACTTCTCCAAGATCCATTGCTCTG--------------------------------------------------------------------------------------- : 1242
FJ708759  : GTCTTGAATGTCTCCATAGTGATAATAATACTTCTCCAAAATCCATTGCTCTG--------------------------------------------------------------------------------------- : 1001
                                                                                                                                                               
                     *      1560         *      1580         *      1600         *      1620         *      1640         *      1660         *      1680       
Genomic   : GGAACTTATCCTCCAGTGGACTGACTGGCCAGATTGATCCAGCTTTCGCCAACCTAACATCAATAAATAAATTGTCAGTGTTTCTAATCTCTTTGTAATCAACATAAGTGCACTCAATGGTATTTAAACTTTTGACTGAC : 1680
NM_119145 : --AACTTATCCTCCAGTGGACTGACTGGCCAGATTGATCCAGCTTTCGCCAACCTAACATCAATAAATAAATT------------------------------------------------------------------- : 1313
X97774    : --AACTTATCCTCCAGTGGACTGACTGGCCAGATTGATCCAGCTTTCGCCAACCTAACATCAATAAATAAATT------------------------------------------------------------------- : 1313
FJ708759  : --AACTTATCCTCCAGTGGACTGACTGGCCAGATTGATCCAGCTTTCGCCAACCTAACATCAATAAATAAATT------------------------------------------------------------------- : 1072
                                                                                                                                                               
                     *      1700         *      1720         *      1740         *      1760         *      1780         *      1800         *      1820       
Genomic   : ACAAGGAAATTATCATTACAGAGACTTATCAAACAACAGCTTAACAGGGAAAGTACCCGATTTCCTCGCCAGTTTACCAAACTTGACTGAATTGTGAGTCGTGCATACTATAGTGGTGAGATTTATAAACCAGTAGCAGT : 1820
NM_119145 : ---------------------AGACTTATCAAACAACAGCTTAACAGGGAAAGTACCCGATTTCCTCGCCAGTTTACCAAACTTGACTGAATT----------------------------------------------- : 1385
X97774    : ---------------------AGACTTATCAAACAACAGCTTAACAGGGAAAGTACCCGATTTCCTCGCCAGTTTACCAAACTTGACTGAATT----------------------------------------------- : 1385
FJ708759  : ---------------------AGACTTATCAAACAACAGCTTAACAGGGAAAGTACCCGATTTCCTCGCCAGTTTACCAAACTTGACTGAATT----------------------------------------------- : 1144
                                                                                                                                                               
                     *      1840         *      1860         *      1880         *      1900         *      1920         *      1940         *      1960       
Genomic   : TTATTACATGTGAAATTTATACAAGGTTCTGTGCTTATACTTGTAGAAACCTGGAAGGAAACAAGTTAACTGGTTCGATTCCAGCTAAACTGTTGGAGAAATCAAAAGATGGTTCTCTTTCGCTTAGGTATGCATCTCAA : 1960
NM_119145 : ----------------------------------------------AAACCTGGAAGGAAACAAGTTAACTGGTTCGATTCCAGCTAAACTGTTGGAGAAATCAAAAGATGGTTCTCTTTCGCTTAG------------- : 1466
X97774    : ----------------------------------------------AAACCTGGAAGGAAACAAGTTAACTGGTTCGATTCCAGCTAAACTGTTGGAGAAATCAAAAGATGGTTCTCTTTCGCTTAG------------- : 1466
FJ708759  : ----------------------------------------------AAACCTGGAAGGAAACAAGTTAACTGGTTCGATTCCAGCTAAACTGTTGGAGAAATCAAAAGATGGTTCTCTTTCGCTTAG------------- : 1225
                                                                                                                                                               
    

                     *      1980         *      2000         *      2020         *      2040         *      2060         *      2080         *      2100       
Genomic   : TGAGCTCTGAAACATAATGTGTGCATTTTCATCAATTTAGTTTCGTATTGCAATCTAGAAAGGATGGCCTTGCTTGTTGCTATGTGAGGTAGTTTGAACCGTTTCTATTGCGAAAGATACAGACTGAAGTTTCTATTTTG : 2100
NM_119145 : -------------------------------------------------------------------------------------------------------------------------------------------- :    -
X97774    : -------------------------------------------------------------------------------------------------------------------------------------------- :    -
FJ708759  : -------------------------------------------------------------------------------------------------------------------------------------------- :    -
                                                                                                                                                               
                     *      2120         *      2140         *      2160         *      2180         *      2200         *      2220         *      2240       
Genomic   : TAAACTTTAGATTTGGTGGAAACCCGGACCTTTGTCAGTCTCCCTCATGTCAAACAACAACGAAGAAGAAAATTGGTTACATCGTCCCAGTAGTAGCATCACTCGCAGGATTGCTCATTGTTTTGACCGCATTAGCTTTG : 2240
NM_119145 : ----------ATTTGGTGGAAACCCGGACCTTTGTCAGTCTCCCTCATGTCAAACAACAACGAAGAAGAAAATTGGTTACATCGTCCCAGTAGTAGCATCACTCGCAGGATTGCTCATTGTTTTGACCGCATTAGCTTTG : 1596
X97774    : ----------ATTTGGTGGAAACCCGGACCTTTGTCAGTCTCCCTCATGTCAAACAACAACGAAGAAGAAAATTGGTTACATCGTCCCAGTAGTAGCATCACTCGCAGGATTGCTCATTGTTTTGACCGCATTAGCTTTG : 1596
FJ708759  : ----------ATTTGGTGGAAACCCGGACCTTTGTCAGTCTCCCTCATGTCAAACAACAACGAAGAAGAAAATTGGTTACATCGTCCCAGTAGTAGCATCACTCGCAGGATTGCTCATTGTTTTGACCGCATTAGCTTTG : 1355
                                                                                                                                                               
                     *      2260         *      2280         *      2300         *      2320         *      2340         *      2360         *      2380       
Genomic   : ATCTGGCATTTTAAAAAACGATCACGAAGAGGTACAATCTCAAACAAACCATTAGGTGTCAACACCGGACCATTGGACACAGCCAAAAGATACTTTATATACTCAGAAGTTGTGAATATCACAAACAACTTCGAGAGAGT : 2380
NM_119145 : ATCTGGCATTTTAAAAAACGATCACGAAGAGGTACAATCTCAAACAAACCATTAGGTGTCAACACCGGACCATTGGACACAGCCAAAAGATACTTTATATACTCAGAAGTTGTGAATATCACAAACAACTTCGAGAGAGT : 1736
X97774    : ATCTGGCATTTTAAAAAACGATCACGAAGAGGTACAATCTCAAACAAACCATTAGGTGTCAACACCGGACCATTGGACACAGCCAAAAGATACTTTATATACTCAGAAGTTGTGAATATCACAAACAACTTCGAGAGAGT : 1736
FJ708759  : ATCTGGCATTTTAAAAAACGATCACGAAGAGGTACAATCTCAAACAAACCATTAGGTGTCAACACCGGACCATTGGACACAGCCAAAAGATACTTTATATACTCAGAAGTTGTGAATATCACAAACAACTTCGAGAGAGT : 1495
                                                                                                                                                               
                     *      2400         *      2420         *      2440         *      2460         *      2480         *      2500         *      2520       
Genomic   : TCTTGGTAAAGGAGGTTTTGGTAAAGTATACCATGGTTTCTTGAATGGAGATCAAGTAGCTGTGAAGATACTTTCCGAAGAGTCAACTCAAGGTTACAAAGAGTTTCGAGCAGAGGTCATTCTCTAAATTACTTGTTAAG : 2520
NM_119145 : TCTTGGTAAAGGAGGTTTTGGTAAAGTATACCATGGTTTCTTGAATGGAGATCAAGTAGCTGTGAAGATACTTTCCGAAGAGTCAACTCAAGGTTACAAAGAGTTTCGAGCAGAGGT----------------------- : 1853
X97774    : TCTTGGTAAAGGAGGTTTTGGTAAAGTATACCATGGTTTCTTGAATGGAGATCAAGTAGCTGTGAAGATACTTTCCGAAGAGTCAACTCAAGGTTACAAAGAGTTTCGAGCAGAGGT----------------------- : 1853
FJ708759  : TCTTGGTAAAGGAGGTTTTGGTAAAGTATACCATGGTTTCTTGAATGGAGATCAAGTAGCTGTGAAGATACTTTCCGAAGAGTCAACTCAAGGTTACAAAGAGTTTCGAGCAGAGGT----------------------- : 1612
                                                                                                                                                               
                     *      2540         *      2560         *      2580         *      2600         *      2620         *      2640         *      2660       
Genomic   : ACATCATTTCTCTGTAATTCCTAATAATGTTTTGCATCCAAAGTCCAAACTAATCTATATTCTCTCAGGTTGAGCTTCTGATGAGAGTCCATCACACAAATTTGACATCTCTTATCGGATACTGCAATGAAGATAACCAC : 2660
NM_119145 : ----------------------------------------------------------------------TGAGCTTCTGATGAGAGTCCATCACACAAATTTGACATCTCTTATCGGATACTGCAATGAAGATAACCAC : 1923
X97774    : ----------------------------------------------------------------------TGAGCTTCTGATGAGAGTCCATCACACAAATTTGACATCTCTTATCGGATACTGCAATGAAGATAACCAC : 1923
FJ708759  : ----------------------------------------------------------------------TGAGCTTCTGATGAGAGTCCATCACACAAATTTGACATCTCTTATCGGATACTGCAATGAAGATAACCAC : 1682
                                                                                                                                                               
                     *      2680         *      2700         *      2720         *      2740         *      2760         *      2780         *      2800       
Genomic   : ATGGCACTTATCTACGAGTACATGGCTAATGGAAACTTAGGAGACTATTTGTCAGGTTAGTCTTTAGATAATAAACTCACAGCCCTTTTGTATTAACAGAAGCAACTAAACTAATTCAGTTTTTTTTTTTTTTTTTCCTT : 2800
NM_119145 : ATGGCACTTATCTACGAGTACATGGCTAATGGAAACTTAGGAGACTATTTGTCAGG------------------------------------------------------------------------------------ : 1979
X97774    : ATGGCACTTATCTACGAGTACATGGCTAATGGAAACTTAGGAGACTATTTGTCAGG------------------------------------------------------------------------------------ : 1979
FJ708759  : ATGGCACTTATCTACGAGTACATGGCTAATGGAAACTTAGGAGACTATTTGTCAGG------------------------------------------------------------------------------------ : 1738
                                                                                                                                                               
                     *      2820         *      2840         *      2860         *      2880         *      2900         *      2920         *      2940       
Genomic   : ATTGTAAAGGAAAAAGTTCCTTAATCTTGAGCTGGGAAGAGAGATTACAGATATCATTGGATGCAGCACAAGGTTTGTAAAATCAAAAAACACAAAATTAGTTCTTTTTAAAAATTTACGCAATCTGAATGAGAATTTTC : 2940
NM_119145 : ----------AAAAAGTTCCTTAATCTTGAGCTGGGAAGAGAGATTACAGATATCATTGGATGCAGCACAAGG------------------------------------------------------------------- : 2042
X97774    : ----------AAAAAGTTCCTTAATCTTGAGCTGGGAAGAGAGATTACAGATATCATTGGATGCAGCACAAGG------------------------------------------------------------------- : 2042
FJ708759  : ----------AAAAAGTTCCTTAATCTTGAGCTGGGAAGAGAGATTACAGATATCATTGGATGCAGCACAAGG------------------------------------------------------------------- : 1801
                                                                                                                                                               
                     *      2960         *      2980         *      3000         *      3020         *      3040         *      3060         *      3080       
Genomic   : AAATGATTAAGGCCTCGAGTATCTCCATTACGGTTGCAAGCCTCCCATAGTTCACAGAGATGTGAAGCCGGCAAATATCTTACTGAACGAGAATCTTCAAGCCAAGATAGCTGACTTTGGTTTATCTAGAAGCTTCCCAG : 3080
NM_119145 : ------------CCTCGAGTATCTCCATTACGGTTGCAAGCCTCCCATAGTTCACAGAGATGTGAAGCCGGCAAATATCTTACTGAACGAGAATCTTCAAGCCAAGATAGCTGACTTTGGTTTATCTAGAAGCTTCCCAG : 2170
X97774    : ------------CCTCGAGTATCTCCATTACGGTTGCAAGCCTCCCATAGTTCACAGAGATGTGAAGCCGGCAAATATCTTACTGAACGAGAATCTTCAAGCCAAGATAGCTGACTTTGGTTTATCTAGAAGCTTCCCAG : 2170
FJ708759  : ------------CCTCGAGTATCTCCATTACGGTTGCAAGCCTCCCATAGTTCACAGAGATGTGAAGCCGGCAAATATCTTACTGAACGAGAATCTTCAAGCCAAGATAGCTGACTTTGGTTTATCTAGAAGCTTCCCAG : 1929
                                                                                                                                                               
                     *      3100         *      3120         *      3140         *      3160         *      3180         *      3200         *      3220       
Genomic   : TCGAAGGTAGTAGTCAAGTCTCCACCGTTGTCGCTGGAACCATCGGTTACTTAGACCCTGAGTAATGATAATCTTTCTCTTAGAATCTAAATTCGTGATTCCGGCTAGATGTATAATTAACCAGATGTGTTCAAATCCTT : 3220
NM_119145 : TCGAAGGTAGTAGTCAAGTCTCCACCGTTGTCGCTGGAACCATCGGTTACTTAGACCCTGAGTA---------------------------------------------------------------------------- : 2234
X97774    : TCGAAGGTAGTAGTCAAGTCTCCACCGTTGTCGCTGGAACCATCGGTTACTTAGACCCTGAGTA---------------------------------------------------------------------------- : 2234
FJ708759  : TCGAAGGTAGTAGTCAAGTCTCCACCGTTGTCGCTGGAACCATCGGTTACTTAGACCCTGAGTA---------------------------------------------------------------------------- : 1993
                                                                                                                                                               
                     *      3240         *      3260         *      3280         *      3300         *      3320         *      3340         *      3360       
Genomic   : TTATTGCCTAAATTCAGGTATTACGCAACGCGGCAAATGAACGAGAAGAGTGATGTTTACAGCTTCGGGGTGGTTCTTCTTGAAGTAATAACAGGAAAACCGGCAATTTGGCATTCAAGAACAGAGAGTGTGCATCTAAG : 3360
NM_119145 : --------------------TTACGCAACGCGGCAAATGAACGAGAAGAGTGATGTTTACAGCTTCGGGGTGGTTCTTCTTGAAGTAATAACAGGAAAACCGGCAATTTGGCATTCAAGAACAGAGAGTGTGCATCTAAG : 2354
X97774    : --------------------TTACGCAACGCGGCAAATGAACGAGAAGAGTGATGTTTACAGCTTCGGGGTGGTTCTTCTTGAAGTAATAACAGGAAAACCGGCAATTTGGCATTCAAGAACAGAGAGTGTGCATCTAAG : 2354
FJ708759  : --------------------TTACGCAACGCGGCAAATGAACGAGAAGAGTGATGTTTACAGCTTCGGGGTGGTTCTTCTTGAAGTAATAACAGGAAAACCGGCAATTTGGCATTCAAGAACAGAGAGTGTGCATCTAAG : 2113
                                                                                                                                                               
                     *      3380         *      3400         *      3420         *      3440         *      3460         *      3480         *      3500       
Genomic   : TGATCAGGTTGGTTCAATGTTGGCGAATGGAGACATAAAAGGTATTGTGGATCAACGTCTGGGAGATAGATTTGAGGTTGGCTCGGCTTGGAAGATCACTGAACTAGCATTGGCTTGTGCGTCTGAGAGTTCAGAACAAA : 3500
NM_119145 : TGATCAGGTTGGTTCAATGTTGGCGAATGGAGACATAAAAGGTATTGTGGATCAACGTCTGGGAGATAGATTTGAGGTTGGCTCGGCTTGGAAGATCACTGAACTAGCATTGGCTTGTGCGTCTGAGAGTTCAGAACAAA : 2494
X97774    : TGATCAGGTTGGTTCAATGTTGGCGAATGGAGACATAAAAGGTATTGTGGATCAACGTCTGGGAGATAGATTTGAGGTTGGCTCGGCTTGGAAGATCACTGAACTAGCATTGGCTTGTGCGTCTGAGAGTTCAGAACAAA : 2494
FJ708759  : TGATCAGGTTGGTTCAATGTTGGCGAATGGAGACATAAAAGGTATTGTGGATCAACGTCTGGGAGATAGATTTGAGGTTGGCTCGGCTTGGAAGATCACTGAACTAGCATTGGCTTGTGCGTCTGAGAGTTCAGAACAAA : 2253
                                                                                                                                                         
                     *      3520         *      3540         *      3560         *      3580         *      3600         *      3620         *           
Genomic   : GACCGACAATGAGCCAAGTCGTTATGGAGCTGAAACAGAGTATCTTTGGGAGAGTGAACAATCGAAGTGACCATAAGGACCCTGTGAGAATGGTTACGATGAACCTCGATACTGAAATGGTTCCTCGGGCGAGG : 3634
NM_119145 : GACCGACAATGAGCCAAGTCGTTATGGAGCTGAAACAGAGTATCTTTGGGAGAGTGAACAATCGAAGTGACCATAAGGACCCTGTGAGAATGGTTACGATGAACCTCGATACTGAAATGGTTCCTCGGGCGAGG : 2628
X97774    : GACCGACAATGAGCCAAGTCGTTATGGAGCTGAAACAGAGTATCTTTGGGAGAGTGAACAATCGAAGTGACCATAAGGACCCTGTGAGAATGGTTACGATGAACCTCGATACTGAAATGGTTCCTCGGGCGAGG : 2628
FJ708759  : GACCGACAATGAGCCAAGTCGTTATGGAGCTGAAACAGAGTATCTTTGGGAGAGTGAACAATCGAAGTGACCATAAGGACCCTGTGAGAATGGTTACGATGAACCTCGATACTGAAATGGTTCCTCGGGCGAGG : 2387


At5g07150
                                                                                                                                                               
                     *        20         *        40         *        60         *        80         *       100         *       120         *       140       
Genomic   : ATGAGTTCCGATCAACGGTGGAGATTGCTCCGACCAGCATTCTTCATCTTCTTCTTCCTCTTCTTTCTTCCTCACAATCTCACCTTTGGTCTCTGTTTCAACACCGAAGGTACTTACTTTTGTTTCATCTTTCTTTCTGA :  140
NM_120797 : ATGAGTTCCGATCAACGGTGGAGATTGCTCCGACCAGCATTCTTCATCTTCTTCTTCCTCTTCTTTCTTCCTCACAATCTCACCTTTGGTCTCTGTTTCAACACCGAAG------------------------------- :  109
FJ708771  : ATGAGTTCCGATCAACGGTGGAGATTGCTCCGACCAGCATTCTTCATCTTCTTCTTCCTCTTCTTTCTTCCTCACAATCTCACCTTTGGTCTCTGTTTCAACACCGAAGGTACTTACTTTTGTTTCATCTTTCTTTCTGA :  140
                                                                                                                                                               
                     *       160         *       180         *       200         *       220         *       240         *       260         *       280       
Genomic   : GTCTATTCGACTATTGATTGATGGATTAACTCTTGTGTGTTTTGCAGCATTGGCCTTGATGAAATTCAAAGAGAGAATAGAGATAGACCCATTTGGAGCTCTGGTTAATTGGGGAGAGCTTTCTCATTGTTCTTGGTCTG :  280
NM_120797 : -----------------------------------------------CATTGGCCTTGATGAAATTCAAAGAGAGAATAGAGATAGACCCATTTGGAGCTCTGGTTAATTGGGGAGAGCTTTCTCATTGTTCTTGGTCTG :  202
FJ708771  : GTCTATTCGACTATTGATTGATGGATTAACTCTTGTGTGTTTTGCAGCATTGGCCTTGATGAAATTCAAAGAGAGAATAGAGATAGACCCATTTGGAGCTCTGGTTAATTGGGGAGAGCTTTCTCATTGTTCTTGGTCTG :  280
                                                                                                                                                               
                     *       300         *       320         *       340         *       360         *       380         *       400         *       420       
Genomic   : GTGTTGTCTGTTCACATGATGGAAGAGTTGTCATCTTGTAAGTCATATCCGAGTCCTTAACATAAGAATAAGAAGTTTCTCTAAAATTTCAGTTTTGTTATAAATTTGATGTCTTATAATGTAGAAATCTAAGAGATCTC :  420
NM_120797 : GTGTTGTCTGTTCACATGATGGAAGAGTTGTCATCTT---------------------------------------------------------------------------------------AAATCTAAGAGATCTC :  255
FJ708771  : GTGTTGTCTGTTCACATGATGGAAGAGTTGTCATCTTGTAAGTCATATCCGAGTCCT----------------------------------------------------------------------------------- :  337
                                                                                                                                                               
                     *       440         *       460         *       480         *       500         *       520         *       540         *       560       
Genomic   : TCCCTACAAGGAACACTTGCACCTGAACTAGGAAACCTAACTCATTTGAAATCTCTGTAAGTAACCAACGTTTTCCCCAACACTCCTCAAACTATACACTAGATAGATAGACCTCTACAACACCCCTTGTTCTTGTTGCA :  560
NM_120797 : TCCCTACAAGGAACACTTGCACCTGAACTAGGAAACCTAACTCATTTGAAATCTCT------------------------------------------------------------------------------------ :  311
FJ708771  : -------------------------------------------------------------------------------------------------------------------------------------------- :    -
                                                                                                                                                               
                     *       580         *       600         *       620         *       640         *       660         *       680         *       700       
Genomic   : GTATTCTCAGAAACAATTCATTTTCCGGGAAAGTACCTGAAGAGGTAACAGAATTGCAGGAGCTTGAGATCTTGGATTTGTGTGACAACAACTTTGGCCAACCATTTCCCTTCGCTCGCAGGCTGCTTCAGATATCTCCC :  700
NM_120797 : -TATTCTCAGAAACAATTCATTTTCCGGGAAAGTACCTGAAGAGGTAACAGAATTGCAGGAGCTTGAGATCTTGGATTTGTGTGACAACAACTTTGGCCAACCATTTCCCTTCGCTCGCAGGCTGCTTCAGATATCTCCC :  450
FJ708771  : --------------------------------------GAAGAGGTAACAGAATTGCAGGAGCTTGAGATCTTGGATTTGTGTGACAACAACTTTGGCCAACCATTTCCCTTCGCTCGCAGGCTGCTTCAGATATCTCCC :  439
                                                                                                                                                               
                     *       720         *       740         *       760         *       780         *       800         *       820         *       840       
Genomic   : CCTCCTTCTCAACCTTCTCCTCCATCGCCTATGGAGGAAGTCCCCATTGATTTTCCTTTCTTCTTTGCCCCTCCTCCGCAGAATATAGGTGCAAGCCCTCCTACTGAAACCCAAGTTATCCCAAACCCGTCTCCAGTTCC :  840
NM_120797 : CCTCCTTCTCAACCTTCTCCTCCATCGCCTATGGAGGAAGTCCCCATTGATTTTCCTTTCTTCTTTGCCCCTCCTCCGCAGAATATAGGTGCAAGCCCTCCTACTGAAACCCAAGTTATCCCAAACCCGTCTCCAGTTCC :  590
FJ708771  : CCTCCTTCTCAACCTTCTCCTCCATCGCCTATGGAGGAAGTCCCCATTGATTTTCCTTTCTTCTTTGCCCCTCCTCCGCAGAATATAGGTGCAAGCCCTCCTACTGAAACCCAAGTTATCCCAAACCCGTCTCCAGTTCC :  579
                                                                                                                                                               
                     *       860         *       880         *       900         *       920         *       940         *       960         *       980       
Genomic   : CCCTCCACCAGCTCAGCCTCCACCAGCTCAGACTCCACCACCTCAGCTTTCTGAAGTGCCTCATGCTGTCAACAAAAAGAAATCTCATAAGAGTAAGATGTACATAATAGTCGGAGTGCTAGTAGGTGTATTAGGCGTCA :  980
NM_120797 : CCCTCCACCAGCTCAGCCTCCACCAGCTCAGACTCCACCACCTCAGCTTTCTGAAGTGCCTCATGCTGTCAACAAAAAGAAATCTCATAAGAGTAAGATGTACATAATAGTCGGAGTGCTAGTAGGTGTATTAGGCGTCA :  730
FJ708771  : CCCTCCACCAGCTCAGCCTCCACCAGCTCAGACTCCACCACCTCAGCTTTCTGAAGTGCCTCATGCTGTCAACAAAAAGAAATCTCATAAGAGTAAGATGTACATAATAGTCGGAGTGCTAGTAGGTGTATTAGGCGTCA :  719
                                                                                                                                                               
                     *      1000         *      1020         *      1040         *      1060         *      1080         *      1100         *      1120       
Genomic   : TGGCCGCATTGGTGGCCTTCTTTTTTCTCTGGAACCAAAAGGTAAAATTGATAAAGCCATGGGGGGAAACTGGGAGCAGTGGCCAGCTTCAAGATGTTGTTACTACAGGTATCATCACTGTTATGTTTACTCTCTAGTTC : 1120
NM_120797 : TGGCCGCATTGGTGGCCTTCTTTTTTCTCTGGAACCAAAAGGTAAAATTGATAAAGCCATGGGGGGAAACTGGGAGCAGTGGCCAGCTTCAAGATGTTGTTACTACAGGT------------------------------ :  840
FJ708771  : TGGCCGCATTGGTGGCCTTCTTTTTTCTCTGGAACCAAAAGGTAAAATTGATAAAGCCATGGGGGGAAACTGGGAGCAGTGGCCAGCTTCAAGATGTTGTTACTACAGGTATCATCACTGTTATGTTTACTCTCTAGTTC :  859
                                                                                                                                                               
                     *      1140         *      1160         *      1180         *      1200         *      1220         *      1240         *      1260       
Genomic   : AATAACATAGCAGAGATTGGTGAAAGTGAGTTTAGTGTATTTATTATGACTTATGAAGGTGTTCCCAAGCTGAAGCTAGCAGAACTAGAAACTGCCTGTGAAGATTTCAGTAACATCATAGGCTCCACATCCTCAGACGC : 1260
NM_120797 : ------------------------------------------------------------GTTCCCAAGCTGAAGCTAGCAGAACTAGAAACTGCCTGTGAAGATTTCAGTAACATCATAGGCTCCACATCCTCAGACGC :  920
FJ708771  : AATAACATAGCAGAGATTGGTGAAAGTGAGTTTAGTGTATTTATTATGACTTATGAAGGTGTTCCCAAGCTGAAGCTAGCAGAACTAGAAACTGCCTGTGAAGATTTCAGTAACATCATAGGCTCCACATCCTCAGACGC :  999
                                                                                                                                                               
                     *      1280         *      1300         *      1320         *      1340         *      1360         *      1380         *      1400       
Genomic   : CACCATTTACAAAGGAACTCTCTCCACCGGCTCTGAAATTGCCGTTCTAGCAGTTGCATCTGGATCCCTCCAAGACTGGTCAGAGGATCACGAAACACAGTTCCAAGAAAAGGTAACATCAGAAACCTCTTTCTGCATTC : 1400
NM_120797 : CACCATTTACAAAGGAACTCTCTCCACCGGCTCTGAAATTGCCGTTCTAGCAGTTGCATCTGGATCCCTCCAAGACTGGTCAGAGGATCACGAAACACAGTTCCAAGAAAAG---------------------------- : 1032
FJ708771  : CACCATTTACAAAGGAACTCTCTCCACCGGCTCTGAAATTGCCGTTCTAGCAGTTGCATCTGGATCCCTCCAAGACTGGTCAGAGGATCACGAAACACAGTTCCAAGAAAAGGTAACATCAGAAACCTCTTTCTGCATTC : 1139
                                                                                                                                                               
                     *      1420         *      1440         *      1460         *      1480         *      1500         *      1520         *      1540       
Genomic   : TTTCTCCTCTTCTTTAAAACACATACAAATCATGCAATGCAGATACAGAGGTTATCTCAAGTGAACCACAAGAACTTCCTCAATGTAATCGGATATTGCCATGAAGACGAACCATTCAACCGAATGCTAGTTTTCGAATA : 1540
NM_120797 : ------------------------------------------------AGGTTATCTCAAGTGAACCACAAGAACTTCCTCAATGTAATCGGATATTGCCATGAAGACGAACCATTCAACCGAATGCTAGTTTTCGAATA : 1124
FJ708771  : TTTCTCCTCTTCTTTAAAACACATACAAATCATGCAATGCAGATACAGAGGTTATCTCAAGTGAACCACAAGAACTTCCTCAATGTAATCGGATATTGCCATGAAGACGAACCATTCAACCGAATGCTAGTTTTCGAATA : 1279
                                                                                                                                                               
                     *      1560         *      1580         *      1600         *      1620         *      1640         *      1660         *      1680       
Genomic   : CGCTCCTAATGGATCCCTCTTCGAGCATCTGCACGGTGAGTAGTAGTAATGCCACTTCTTCCAACAGACTCAGTCCCTCCCATCTATTTTTTAAACTTGTAACTGTAATATTATGTTGCAGACCAAGACGCAGAGCATTT : 1680
NM_120797 : CGCTCCTAATGGATCCCTCTTCGAGCATCTGCACG--------------------------------------------------------------------------------------ACCAAGACGCAGAGCATTT : 1178
FJ708771  : CGCTCCTAATGGATCCCTCTTCGAGCATCTGCACGGTGAGTAGTAGTAATGCCACTTCTTCCAACAGACTCAGTCCCTCCCATCTATTTTTTAAACTTGTAACTGTAATATTATGTTGCAGACCAAGACGCAGAGCATTT : 1419
                                                                                                                                                               
                     *      1700         *      1720         *      1740         *      1760         *      1780         *      1800         *      1820       
Genomic   : GGACTGGCCAATGAGACTGAGAATCGTAATGGGAATAGCTTACTGTATGGAACATATGCACAATCTAAACCCTAAACCCATCTCTCACACCAACCTCAACTCTTCTTCAGTCTACTTGGCAACAGATTACGCAGCCAAAG : 1820
NM_120797 : GGACTGGCCAATGAGACTGAGAATCGTAATGGGAATAGCTTACTGTATGGAACATATGCACAATCTAAACCCTAAACCCATCTCTCACACCAACCTCAACTCTTCTTCAGTCTACTTGGCAACAGATTACGCAGCCAAAG : 1318
FJ708771  : GGACTGGCCAATGAGACTGAGAATCGTAATGGGAATAGCTTACTGTATGGAACATATGCACAATCTAAACCCTAAACCCATCTCTCACACCAACCTCAACTCTTCTTCAGTCTACTTGGCAACAGATTACGCAGCCAAAG : 1559
                                                                                                                                                               
                     *      1840         *      1860         *      1880         *      1900         *      1920         *      1940         *      1960       
Genomic   : TCTCAGACTTTACGTTCCTCAGCTCCACACCACTTGACCCCATGACCAACGTATCCAGCTTTGGCGCCCTTCTACAAGAGATCATCACCGGAAAGATCCCGGATCCAGATTCTCTGCTCCAGGAAGAAACCAAACCCGTT : 1960
NM_120797 : TCTCAGACTTTACGTTCCTCAGCTCCACACCACTTGACCCCATGACCAACGTATCCAGCTTTGGCGCCCTTCTACAAGAGATCATCACCGGAAAGATCCCGGATCCAGATTCTCTGCTCCAGGAAGAAACCAAACCCGTT : 1458
FJ708771  : TCTCAGACTTTACGTTCCTCAGCTCCACACCACTTGACCCCATGACCAACGTATCCAGCTTTGGCGCCCTTCTACAAGAGATCATCACCGGAAAGATCCCGGATCCAGATTCTCTGCTCCAGGAAGAAACCAAACCCGTT : 1699
                                                                                                                                                               
                     *      1980         *      2000         *      2020         *      2040         *      2060         *      2080         *      2100       
Genomic   : GCAGACCCGACTCTGAAAAGCTTTCAAGAGGAGGTCATGGAGAGAGTGTGGGAAGTGGTTAAAGAGTGTTTGAGTCAGAAGGTGGAAATGAAGGAAGTGGTGGTTAAGCTGAGAGAGATCACCGGAATAACGCCGGAAGC : 2100
NM_120797 : GCAGACCCGACTCTGAAAAGCTTTCAAGAGGAGGTCATGGAGAGAGTGTGGGAAGTGGTTAAAGAGTGTTTGAGTCAGAAGGTGGAAATGAAGGAAGTGGTGGTTAAGCTGAGAGAGATCACCGGAATAACGCCGGAAGC : 1598
FJ708771  : GCAGACCCGACTCTGAAAAGCTTTCAAGAGGAGGTCATGGAGAGAGTGTGGGAAGTGGTTAAAGAGTGTTTGAGTCAGAAGGTGGAAATGAAGGAAGTGGTGGTTAAGCTGAGAGAGATCACCGGAATAACGCCGGAAGC : 1839
                                                                                
                     *      2120         *      2140         *      2160        
Genomic   : AGCGTTGCCTAGTCGGTCTCCGGCGTGGTGGGCGGAGCTGGAGATTATATCCACCGAAATG : 2161
NM_120797 : AGCGTTGCCTAGTCGGTCTCCGGCGTGGTGGGCGGAGCTGGAGATTATATCCACCGAAATG : 1659
FJ708771  : AGCGTTGCCTAGTCGGTCTCCGGCGTGGTGGGCGGAGCTGGAGATTATATCCACCGAAATG : 1900


At5g44700
                                                                                                                                                               
                     *        20         *        40         *        60         *        80         *       100         *       120         *       140       
Genomic   : ATGCAGCAAAACTCTGTTCTTCTTGCTCTGTTTTTCCTCTGTTTCTCATCCGGGTTGGGTTCGGGTCAACCGGGTCAAAGAGACGATCTTCAAACTCTTCTCGAACTGAAGAACTCTTTTATCACAAACCCAAAAGAAGA :  140
NM_123837 : ATGCAGCAAAACTCTGTTCTTCTTGCTCTGTTTTTCCTCTGTTTCTCATCCGGGTTGGGTTCGGGTCAACCGGGTCAAAGAGACGATCTTCAAACTCTTCTCGAACTGAAGAACTCTTTTATCACAAACCCAAAAGAAGA :  140
FJ708788  : ATGCAGCAAAACTCTGTTCTTCTTGCTCTGTTTTTCCTCTGTTTCTCATCCGGGTTGGGTTCGGGTCAACCGGGTCAAAGAGACGATCTTCAAACTCTTCTCGAACTGAAGAACTCTTTTATCACAAACCCAAAAGAAGA :  140
                                                                                                                                                               
                     *       160         *       180         *       200         *       220         *       240         *       260         *       280       
Genomic   : AGACGTTCTCCGAGATTGGAATTCCGGTAGTCCCAGTTACTGCAACTGGACCGGCGTCACATGCGGTGGTCGTGAAATCATCGGTTTAAATCTCTCCGGTTTAGGTTTAACCGGTTCAATTTCTCCTTCGATCGGCCGGT :  280
NM_123837 : AGACGTTCTCCGAGATTGGAATTCCGGTAGTCCCAGTTACTGCAACTGGACCGGCGTCACATGCGGTGGTCGTGAAATCATCGGTTTAAATCTCTCCGGTTTAGGTTTAACCGGTTCAATTTCTCCTTCGATCGGCCGGT :  280
FJ708788  : AGACGTTCTCCGAGATTGGAATTCCGGTAGTCCCAGTTACTGCAACTGGACCGGCGTCACATGCGGTGGTCGTGAAATCATCGGTTTAAATCTCTCCGGTTTAGGTTTAACCGGTTCAATTTCTCCTTCGATCGGCCGGT :  280
                                                                                                                                                               
                     *       300         *       320         *       340         *       360         *       380         *       400         *       420       
Genomic   : TTAACAACCTAATCCACATCGATTTGTCTTCGAACCGTCTTGTCGGTCCCATCCCAACGACTCTCTCTAACCTCTCCTCTTCATTGGAATCGTTGCATCTCTTCTCTAACCTACTCAGTGGCGATATACCGAGTCAACTC :  420
NM_123837 : TTAACAACCTAATCCACATCGATTTGTCTTCGAACCGTCTTGTCGGTCCCATCCCAACGACTCTCTCTAACCTCTCCTCTTCATTGGAATCGTTGCATCTCTTCTCTAACCTACTCAGTGGCGATATACCGAGTCAACTC :  420
FJ708788  : TTAACAACCTAATCCACATCGATTTGTCTTCGAACCGTCTTGTCGGTCCCATCCCAACGACTCTCTCTAACCTCTCCTCTTCATTGGAATCGTTGCATCTCTTCTCTAACCTACTCAGTGGCGATATACCGAGTCAACTC :  420
                                                                                                                                                               
                     *       440         *       460         *       480         *       500         *       520         *       540         *       560       
Genomic   : GGCTCACTTGTGAATCTCAAATCGTTAAAACTCGGAGACAACGAACTCAACGGAACAATCCCGGAGACGTTTGGGAACCTCGTCAACCTCCAGATGCTTGCTTTGGCTTCGTGTAGACTCACCGGTCTTATACCGAGTCG :  560
NM_123837 : GGCTCACTTGTGAATCTCAAATCGTTAAAACTCGGAGACAACGAACTCAACGGAACAATCCCGGAGACGTTTGGGAACCTCGTCAACCTCCAGATGCTTGCTTTGGCTTCGTGTAGACTCACCGGTCTTATACCGAGTCG :  560
FJ708788  : GGCTCACTTGTGAATCTCAAATCGTTAAAACTCGGAGACAACGAACTCAACGGAACAATCCCGGAGACGTTTGGGAACCTCGTCAACCTCCAGA---------------------------------------------- :  514
                                                                                                                                                               
                     *       580         *       600         *       620         *       640         *       660         *       680         *       700       
Genomic   : ATTCGGTAGACTCGTTCAGCTCCAGACTTTGATACTACAAGACAACGAACTCGAAGGACCGATTCCGGCGGAAATCGGAAACTGCACCAGCCTCGCTTTGTTTGCCGCGGCGTTTAATCGTCTCAACGGTTCGTTACCGG :  700
NM_123837 : ATTCGGTAGACTCGTTCAGCTCCAGACTTTGATACTACAAGACAACGAACTCGAAGGACCGATTCCGGCGGAAATCGGAAACTGCACCAGCCTCGCTTTGTTTGCCGCGGCGTTTAATCGTCTCAACGGTTCGTTACCGG :  700
FJ708788  : -------------------------------------------------------------------------------------------------------------------------------------------- :    -
                                                                                                                                                               
                     *       720         *       740         *       760         *       780         *       800         *       820         *       840       
Genomic   : CGGAGCTGAATCGACTTAAGAACCTCCAGACGCTGAATTTGGGAGATAACAGTTTCTCCGGTGAGATACCGAGTCAACTCGGTGATTTGGTTAGTATCCAATACCTTAATTTGATTGGTAATCAGTTACAGGGTTTGATT :  840
NM_123837 : CGGAGCTGAATCGACTTAAGAACCTCCAGACGCTGAATTTGGGAGATAACAGTTTCTCCGGTGAGATACCGAGTCAACTCGGTGATTTGGTTAGTATCCAATACCTTAATTTGATTGGTAATCAGTTACAGGGTTTGATT :  840
FJ708788  : -------------------------------------------------------------------------------------------------------------------------------------------- :    -
                                                                                                                                                               
                     *       860         *       880         *       900         *       920         *       940         *       960         *       980       
Genomic   : CCAAAGAGATTAACAGAATTAGCGAATCTTCAAACACTTGATTTGTCTTCTAATAATCTCACCGGAGTGATACACGAAGAGTTCTGGAGAATGAATCAGCTTGAGTTCTTGGTATTAGCGAAAAATCGTCTCTCTGGTTC :  980
NM_123837 : CCAAAGAGATTAACAGAATTAGCGAATCTTCAAACACTTGATTTGTCTTCTAATAATCTCACCGGAGTGATACACGAAGAGTTCTGGAGAATGAATCAGCTTGAGTTCTTGGTATTAGCGAAAAATCGTCTCTCTGGTTC :  980
FJ708788  : -------------------------------------------------------------------------------------------------------------------------------------------- :    -
                                                                                                                                                               
                     *      1000         *      1020         *      1040         *      1060         *      1080         *      1100         *      1120       
Genomic   : GTTACCAAAGACTATATGTTCTAACAACACAAGCTTGAAGCAACTGTTTCTGTCTGAAACTCAGCTTTCCGGCGAAATTCCAGCGGAAATCAGCAACTGTCAGTCATTAAAATTGCTTGATTTGTCGAACAATACGCTTA : 1120
NM_123837 : GTTACCAAAGACTATATGTTCTAACAACACAAGCTTGAAGCAACTGTTTCTGTCTGAAACTCAGCTTTCCGGCGAAATTCCAGCGGAAATCAGCAACTGTCAGTCATTAAAATTGCTTGATTTGTCGAACAATACGCTTA : 1120
FJ708788  : -------------------------------------------------------------------------------------------------------------------------------------------- :    -
                                                                                                                                                               
                     *      1140         *      1160         *      1180         *      1200         *      1220         *      1240         *      1260       
Genomic   : CAGGTCAAATCCCGGATTCGTTGTTTCAGCTCGTCGAACTCACGAATCTTTATCTTAACAACAATAGCTTGGAGGGTACGTTATCTTCATCAATATCAAACCTCACGAATCTACAAGAGTTTACTCTCTATCACAATAAC : 1260
NM_123837 : CAGGTCAAATCCCGGATTCGTTGTTTCAGCTCGTCGAACTCACGAATCTTTATCTTAACAACAATAGCTTGGAGGGTACGTTATCTTCATCAATATCAAACCTCACGAATCTACAAGAGTTTACTCTCTATCACAATAAC : 1260
FJ708788  : -------------------------------------------------------------------------------------------------------------------------------------------- :    -
                                                                                                                                                               
                     *      1280         *      1300         *      1320         *      1340         *      1360         *      1380         *      1400       
Genomic   : TTGGAAGGCAAGGTACCTAAAGAAATCGGATTTCTTGGTAAATTGGAGATTATGTATCTGTATGAGAATCGGTTTTCGGGTGAAATGCCGGTTGAGATTGGGAATTGCACGAGACTGCAGGAGATTGATTGGTACGGGAA : 1400
NM_123837 : TTGGAAGGCAAGGTACCTAAAGAAATCGGATTTCTTGGTAAATTGGAGATTATGTATCTGTATGAGAATCGGTTTTCGGGTGAAATGCCGGTTGAGATTGGGAATTGCACGAGACTGCAGGAGATTGATTGGTACGGGAA : 1400
FJ708788  : -------------------------------------------------------------------------------------------------------------------------------------------- :    -                                                                                                                                                               
                                                                                                                                                               
                     *      1420         *      1440         *      1460         *      1480         *      1500         *      1520         *      1540       
Genomic   : TCGTTTAAGTGGAGAGATTCCTTCTTCAATTGGAAGATTGAAAGATCTTACTCGTCTTCACTTGAGAGAGAACGAGCTTGTAGGTAACATTCCCGCCAGTTTAGGTAACTGTCATCAAATGACGGTTATCGATTTAGCGG : 1540
NM_123837 : TCGTTTAAGTGGAGAGATTCCTTCTTCAATTGGAAGATTGAAAGATCTTACTCGTCTTCACTTGAGAGAGAACGAGCTTGTAGGTAACATTCCCGCCAGTTTAGGTAACTGTCATCAAATGACGGTTATCGATTTAGCGG : 1540
FJ708788  : -------------------------------------------------------------------------------------------------------------------------------------------- :    -
                                                                                                                                                               
                     *      1560         *      1580         *      1600         *      1620         *      1640         *      1660         *      1680       
Genomic   : ATAACCAGCTCTCGGGTTCAATTCCTTCCTCGTTCGGGTTCTTAACGGCATTAGAACTGTTTATGATCTACAACAATTCTCTTCAAGGAAATCTTCCGGATTCACTTATCAACCTCAAGAACCTCACAAGAATCAATTTT : 1680
NM_123837 : ATAACCAGCTCTCGGGTTCAATTCCTTCCTCGTTCGGGTTCTTAACGGCATTAGAACTGTTTATGATCTACAACAATTCTCTTCAAGGAAATCTTCCGGATTCACTTATCAACCTCAAGAACCTCACAAGAATCAATTTT : 1680
FJ708788  : -------------------------------------------------------------------------------------------------------------------------------------------- :    -
                                                                                                                                                               
                     *      1700         *      1720         *      1740         *      1760         *      1780         *      1800         *      1820       
Genomic   : TCAAGCAACAAGTTCAATGGTTCGATAAGTCCATTGTGCGGTTCAAGCTCGTATCTCTCGTTTGATGTCACGGAAAACGGATTTGAGGGAGATATACCTCTTGAGCTAGGTAAGTCTACGAATCTTGATCGGTTAAGGCT : 1820
NM_123837 : TCAAGCAACAAGTTCAATGGTTCGATAAGTCCATTGTGCGGTTCAAGCTCGTATCTCTCGTTTGATGTCACGGAAAACGGATTTGAGGGAGATATACCTCTTGAGCTAGGTAAGTCTACGAATCTTGATCGGTTAAGGCT : 1820
FJ708788  : -------------------------------------------------------------------------------------------------------------------------------------------- :    -
                                                                                                                                                               
                     *      1840         *      1860         *      1880         *      1900         *      1920         *      1940         *      1960       
Genomic   : GGGGAAGAATCAGTTCACAGGAAGAATCCCTCGGACATTTGGGAAGATCAGTGAGCTTTCTTTATTGGATATTTCAAGAAACTCTCTCTCAGGAATCATACCGGTGGAGCTCGGTTTGTGCAAGAAGCTGACACACATTG : 1960
NM_123837 : GGGGAAGAATCAGTTCACAGGAAGAATCCCTCGGACATTTGGGAAGATCAGTGAGCTTTCTTTATTGGATATTTCAAGAAACTCTCTCTCAGGAATCATACCGGTGGAGCTCGGTTTGTGCAAGAAGCTGACACACATTG : 1960
FJ708788  : -------------------------------------------------------------------------------------------------------------------------------------------- :    -
                                                                                                                                                               
                     *      1980         *      2000         *      2020         *      2040         *      2060         *      2080         *      2100       
Genomic   : ATCTCAACAATAACTATCTTTCAGGGGTGATACCTACATGGCTTGGAAAGCTTCCATTGTTGGGAGAGCTAAAGCTTTCTTCTAATAAGTTTGTTGGGTCTCTCCCTACTGAGATTTTCAGCTTAACCAATATTCTTACG : 2100
NM_123837 : ATCTCAACAATAACTATCTTTCAGGGGTGATACCTACATGGCTTGGAAAGCTTCCATTGTTGGGAGAGCTAAAGCTTTCTTCTAATAAGTTTGTTGGGTCTCTCCCTACTGAGATTTTCAGCTTAACCAATATTCTTACG : 2100
FJ708788  : -------------------------------------------------------------------------------------------------------------------------------------------- :    -
                                                                                                                                                               
                     *      2120         *      2140         *      2160         *      2180         *      2200         *      2220         *      2240       
Genomic   : CTATTTCTTGATGGTAACTCACTCAACGGCTCGATCCCGCAAGAGATTGGTAACTTGCAAGCTCTCAATGCGTTGAACCTCGAAGAGAATCAGCTTTCAGGTCCTCTGCCTTCTACAATAGGGAAACTGAGCAAGCTTTT : 2240
NM_123837 : CTATTTCTTGATGGTAACTCACTCAACGGCTCGATCCCGCAAGAGATTGGTAACTTGCAAGCTCTCAATGCGTTGAACCTCGAAGAGAATCAGCTTTCAGGTCCTCTGCCTTCTACAATAGGGAAACTGAGCAAGCTTTT : 2240
FJ708788  : -------------------------------------------------------------------------------------------------------------------------------------------- :    -
                                                                                                                                                               
                     *      2260         *      2280         *      2300         *      2320         *      2340         *      2360         *      2380       
Genomic   : CGAGCTTCGGTTATCGAGAAACGCTTTAACCGGAGAGATTCCTGTTGAGATTGGACAGCTGCAAGATCTTCAAAGTGCTTTAGATCTCAGCTACAACAACTTTACCGGACGTATACCGTCTACGATTTCGACGTTACCTA : 2380
NM_123837 : CGAGCTTCGGTTATCGAGAAACGCTTTAACCGGAGAGATTCCTGTTGAGATTGGACAGCTGCAAGATCTTCAAAGTGCTTTAGATCTCAGCTACAACAACTTTACCGGACGTATACCGTCTACGATTTCGACGTTACCTA : 2380
FJ708788  : -------------------------------------------------------------------------------------------------------------------------------------------- :    -
                                                                                                                                                               
    
                     *      2400         *      2420         *      2440         *      2460         *      2480         *      2500         *      2520       
Genomic   : AGCTTGAATCTCTTGATCTGTCTCACAATCAGCTTGTAGGAGAAGTTCCTGGCCAAATCGGTGACATGAAAAGCTTGGGATATCTTAACCTCTCTTACAACAACCTTGAGGGAAAGTTAAAGAAACAGTTCTCTAGATGG : 2520
NM_123837 : AGCTTGAATCTCTTGATCTGTCTCACAATCAGCTTGTAGGAGAAGTTCCTGGCCAAATCGGTGACATGAAAAGCTTGGGATATCTTAACCTCTCTTACAACAACCTTGAGGGAAAGTTAAAGAAACAGTTCTCTAGATGG : 2520
FJ708788  : -------------------------------------------------------------------------------------------------------------------------------------------- :    -
                                                                                                                                                               
                                                                                                                                                               
                     *      2540         *      2560         *      2580         *      2600         *      2620         *      2640         *      2660       
Genomic   : CAAGCTGATGCGTTTGTAGGCAATGCAGGTCTTTGTGGAAGCCCTCTTAGTCATTGCAACAGAGCTGGATCAAAGAATCAACGAAGTCTTAGTCCGAAAACAGTGGTTATAATCTCTGCGATTTCATCATTAGCAGCGAT : 2660
NM_123837 : CAAGCTGATGCGTTTGTAGGCAATGCAGGTCTTTGTGGAAGCCCTCTTAGTCATTGCAACAGAGCTGGATCAAAGAATCAACGAAGTCTTAGTCCGAAAACAGTGGTTATAATCTCTGCGATTTCATCATTAGCAGCGAT : 2660
FJ708788  : -------------------------------------------------------------------------------------------------------------------------------------------- :    -
                                                                                                                                                               
                                                                                                                                                               
                     *      2680         *      2700         *      2720         *      2740         *      2760         *      2780         *      2800       
Genomic   : TGCTTTGATGGTACTTGTTATCATCCTCTTCTTCAAGCAAAACCATGATCTTTTCAAGAAAGTGCGAGGCGGAAACAGCGCATTTTCATCGAACTCTTCTTCTTCACAAGCTCCTCTATTTAGTAATGGAGGTGCAAAGT : 2800
NM_123837 : TGCTTTGATGGTACTTGTTATCATCCTCTTCTTCAAGCAAAACCATGATCTTTTCAAGAAAGTGCGAGGCGGAAACAGCGCATTTTCATCGAACTCTTCTTCTTCACAAGCTCCTCTATTTAGTAATGGAGGTGCAAAGT : 2800
FJ708788  : -------------------------------------------------------------------------------------------------------------------------------------------- :    -
                                                                                                                                                               
                                                                                                                                                               
                     *      2820         *      2840         *      2860         *      2880         *      2900         *      2920         *      2940       
Genomic   : CAGATATAAAGTGGGATGACATAATGGAAGCTACACATTACCTTAACGAAGAGTTCATGATTGGATCAGGAGGGTCAGGAAAAGTTTACAAAGCGGAGTTAAAGAACGGCGAGACGATAGCTGTGAAGAAGATTCTTTGG : 2940
NM_123837 : CAGATATAAAGTGGGATGACATAATGGAAGCTACACATTACCTTAACGAAGAGTTCATGATTGGATCAGGAGGGTCAGGAAAAGTTTACAAAGCGGAGTTAAAGAACGGCGAGACGATAGCTGTGAAGAAGATTCTTTGG : 2940
FJ708788  : -------------------------------------------------------------------------------------------------------------------------------------------- :    -
                                                                                                                                                               
                                                                                                                                                               
                     *      2960         *      2980         *      3000         *      3020         *      3040         *      3060         *      3080       
Genomic   : AAAGATGATTTGATGTCAAACAAGAGCTTTAACAGAGAAGTTAAGACACTTGGAACAATCAGACATAGACATTTGGTTAAGCTAATGGGTTATTGTAGCAGTAAAGCAGATGGTTTGAATCTGTTGATCTACGAGTATAT : 3080
NM_123837 : AAAGATGATTTGATGTCAAACAAGAGCTTTAACAGAGAAGTTAAGACACTTGGAACAATCAGACATAGACATTTGGTTAAGCTAATGGGTTATTGTAGCAGTAAAGCAGATGGTTTGAATCTGTTGATCTACGAGTATAT : 3080
FJ708788  : -------------------------------------------------------------------------------------------------------------------------------------------- :    -
                                                                                                                                                               
                                                                                                                                                               
                     *      3100         *      3120         *      3140         *      3160         *      3180         *      3200         *      3220       
Genomic   : GGCGAATGGAAGCGTTTGGGATTGGCTTCATGCCAACGAGAATACGAAGAAAAAGGAGGTTCTTGGTTGGGAAACAAGATTGAAAATAGCACTTGGGTTGGCTCAAGGAGTGGAGTATCTTCATTATGACTGTGTTCCTC : 3220
NM_123837 : GGCGAATGGAAGCGTTTGGGATTGGCTTCATGCCAACGAGAATACGAAGAAAAAGGAGGTTCTTGGTTGGGAAACAAGATTGAAAATAGCACTTGGGTTGGCTCAAGGAGTGGAGTATCTTCATTATGACTGTGTTCCTC : 3220
FJ708788  : -------------------------------------------------------------------------------------------------------------------------------------------- :    -
                                                                                                                                                               
                                                                                                                                                               
                     *      3240         *      3260         *      3280         *      3300         *      3320         *      3340         *      3360       
Genomic   : CGATTGTTCATCGCGATATTAAGTCTAGTAATGTGCTTCTTGATTCCAACATAGAAGCACATTTAGGAGATTTCGGACTCGCCAAGATCCTAACCGGGAATTATGACACCAACACAGAATCAAACACTATGTTTGCAGGC : 3360
NM_123837 : CGATTGTTCATCGCGATATTAAGTCTAGTAATGTGCTTCTTGATTCCAACATAGAAGCACATTTAGGAGATTTCGGACTCGCCAAGATCCTAACCGGGAATTATGACACCAACACAGAATCAAACACTATGTTTGCAGGC : 3360
FJ708788  : -------------------------------------------------------------------------------------------------------------------------------------------- :    -
                                                                                                                                                               
                                                                                                                                                               
                     *      3380         *      3400         *      3420         *      3440         *      3460         *      3480         *      3500       
Genomic   : TCTTATGGCTACATCGCGCCAGGTACGGTACATACATGTCTTTCTCGGGTTTCTTAACTACGATTCGACTATTAAACCTAACGTGTTGGATACCGTTCTTTTTTCGCAGAGTATGCGTACTCGTTGAAGGCGACTGAGAA : 3500
NM_123837 : TCTTATGGCTACATCGCGCCAG---------------------------------------------------------------------------------------AGTATGCGTACTCGTTGAAGGCGACTGAGAA : 3413
FJ708788  : --------------------------------------------------------------------------------------------------------------GTATGCGTACTCGTTGAAGGCGACTGAGAA :  544
                                                                                                                                                               
                                                                                                                                                               
                     *      3520         *      3540         *      3560         *      3580         *      3600         *      3620         *      3640       
Genomic   : GAGCGATGTTTACAGTATGGGGATAGTGTTGATGGAGATTGTGACTGGTAAAATGCCAACCGAAGCAATGTTTGATGAAGAGACGGATATGGTGAGATGGGTAGAGACAGTTCTTGATACACCTCCGGGTTCTGAAGCAA : 3640
NM_123837 : GAGCGATGTTTACAGTATGGGGATAGTGTTGATGGAGATTGTGACTGGTAAAATGCCAACCGAAGCAATGTTTGATGAAGAGACGGATATGGTGAGATGGGTAGAGACAGTTCTTGATACACCTCCGGGTTCTGAAGCAA : 3553
FJ708788  : GAGCGATGTTTACAGTATGGGGATAGTGTTGATGGAGATTGTGACTGGTAAAATGCCAACCGAAGCAATGTTTGATGAAGAGACGGATATGGTGAGATGGGTAGAGACAGTTCTTGATACACCTCCGGGTTCTGAAGCAA :  684
                                                                                                                                                               
                                                                                                                                                               
                     *      3660         *      3680         *      3700         *      3720         *      3740         *      3760         *      3780       
Genomic   : GAGAGAAGCTGATTGATTCAGAGCTTAAATCGCTGTTACCTTGCGAAGAAGAAGCAGCTTATCAGGTTCTTGAAATAGCACTTCAGTGCACAAAAAGTTATCCTCAAGAGAGACCTTCTTCAAGACAAGCTAGTGAATAT : 3780
NM_123837 : GAGAGAAGCTGATTGATTCAGAGCTTAAATCGCTGTTACCTTGCGAAGAAGAAGCAGCTTATCAGGTTCTTGAAATAGCACTTCAGTGCACAAAAAGTTATCCTCAAGAGAGACCTTCTTCAAGACAAGCTAGTGAATAT : 3693
FJ708788  : GAGAGAAGCTGATTGATTCAGAGCTTAAATCGCTGTTACCTTGCGAAGAAGAAGCAGCTTATCAGGTTCTTGAAATAGCACTTCAGTGCACAAAAAGTTATCCTCAAGAGAGACCTTCTTCAAGACAAGCTAGTGAATAT :  824
                                                                                                                                                               
                                                                                  
                     *      3800         *      3820         *      3840          
Genomic   : CTTCTTAATGTCTTCAACAATAGAGCTGCCAGTTACAGGGAGATGCAAACTGATACCGATAAA : 3843
NM_123837 : CTTCTTAATGTCTTCAACAATAGAGCTGCCAGTTACAGGGAGATGCAAACTGATACCGATAAA : 3756
FJ708788  : CTTCTTAATGTCTTCAACAATAGAGCTGCCAGTTACAGGGAGATGCAAACTGATACCGATAAA :  887
